# Supplementary material for: Did mpox knowledge, attitudes and beliefs affect intended behaviour in the general population and men who are gay, bisexual and who have sex with men? An online cross-sectional survey in the UK
Source: BMJ Open. 2023 Oct 12;13(10):e070882. doi: 10.1136/bmjopen-2022-070882 (PMC10583036; doi:10.1136/bmjopen-2022-070882)
Supplement: Supplementary data [file bmjopen-2022-070882supp001.pdf]

Supplementary materials 1. Full survey materials and top-line results for general population and GBMSM samples separately

For categorical data, n (%) are shown. Percentages are column percentages. Where totals do not add up to 100%, it is due to rounding errors.

For continuous data, n (where different to total sample), mean (M), standard deviation (SD), and range are shown.

Total *n*s are: General population, n=3050; Savanta GBMSM, n=247; Grindr, n=831; Meta [Facebook and Instagram], n=1036.

Screening questions

ASK ALL
Postcode What is your full postcode?

We will only use your postcode to allocate you to groups based on your location (e.g. region). Your full postcode will not be passed on to the research team.

Type your answer below
OPEN END

OPEN TEXT ENTRY
Prefer not to say

New screen

ASK ALL
Gender What is your gender?
SINGLE CODE

	General population	Savanta GBMSM	Grindr	Meta
Male (including trans man)	1282 (42.0)	247 (100.0)	827 (99.5)	1030 (99.4)
Female (including trans woman)	1729 (56.7)	0 (0.0)	0 (0.0)	0 (0.0)
Non-binary	17 (0.6)	0 (0.0)	0 (0.0)	0 (0.0)
Other, please state	15 (0.5)	0 (0.0)	4 (0.5)	6 (0.6)
Prefer not to say	7 (0.2)	0 (0.0)	0 (0.0)	0 (0.0)

Smith LE, *et al. BMJ Open* 2023; 13:e070882. doi: 10.1136/bmjopen-2022-070882

** For main analyses, where possible, text answers from "other, please state" were used to assign participants to a group. Therefore, numbers presented in the main manuscript may differ slightly.*

New screen

ASK ALL
SexBirth Is your gender the same as your gender assigned at birth?
SINGLE CODE

	General population	Savanta GBMSM	Grindr	Meta
Yes	3022 (99.1)	238 (96.4)	828 (99.6)	1013 (97.8)
No	22 (0.7)	9 (3.6)	2 (0.2)	18 (1.7)
Prefer not to say	6 (0.2)	0 (0.0)	1 (0.1)	5 (0.5)

New screen

ASK ALL
Age How old are you (in years)?
Type your answer below
NUMERIC - OPEN END

	General population	Savanta GBMSM	Grindr	Meta
OPEN TEXT ENTRY – number [cap at 110]	M=48.6, SD=17.4, range 18 to 98.	M=47.1, SD=16.5, range 18 to 77.	M=44.2, SD=12.5, range 18 to 82.	M=47.6, SD=11.9, range 18 to 79.

SCREEN OUT if under 18 years.

New screen

ASK ALL
Sexuality Which of the following best describes your sexual orientation?
SINGLE CODE, RANDOMISE

	General population	Savanta GBMSM	Grindr	Meta
Straight or heterosexual	2795 (91.6)	0 (0.0)	0 (0.0)	0 (0.0)
Gay or lesbian	78 (2.6)	230 (93.1)	706 (85.0)	967 (93.3)
Bisexual	135 (4.4)	16 (6.5)	118 (14.2)	55 (5.3)
Other sexual orientation, please state [ANCHOR]	18 (0.6)	1 (0.4)	7 (0.8)	14 (1.4)
Prefer not to say	24 (0.8)	0 (0.0)	0 (0.0)	0 (0.0)

[ANCHOR]				

** For main analyses, where possible, text answers from "other, please state" were used to assign participants to a group. Therefore, numbers presented in the main manuscript may differ slightly.*

New screen

ASK ALL
SEG Which of the following best describes the profession of the chief income earner in your household?
SINGLE CODE

	General population	Savanta GBMSM	Grindr	Meta
**High managerial, administrative or professional** e.g. doctor, lawyer, medium / large company director (50+ people)	193 (6.3)	28 (11.3)	144 (17.3)	253 (24.4)
**Intermediate managerial, administrative or professional** e.g. teacher, manager, accountant	608 (19.9)	57 (23.1)	308 (37.1)	381 (36.8)
**Supervisor, administrative or professional** e.g. police officer, nurse, secretary, self-employed	628 (20.6)	51 (20.6)	175 (21.1)	195 (18.8)
**Skilled manual worker** e.g. mechanic, plumber, electrician, lorry driver, train driver	395 (13.0)	17 (6.9)	42 (5.1)	18 (1.7)
**Semi-skilled or unskilled manual worker** e.g. waiter, factory worker, receptionist, labourer	328 (10.8)	18 (7.3)	45 (5.4)	31 (3.0)
**House-wife / house-husband**	76 (2.5)	3 (1.2)	4 (0.5)	0.0 (0.0)
**Unemployed**	176 (5.8)	24 (9.7)	27 (3.2)	26 (2.5)
**Student**	52 (1.7)	5 (2.0)	30 (3.6)	32 (3.1)
**Retired on a state pension**	215 (7.0)	9 (3.6)	15 (1.8)	13 (1.3)
**Retired on a private pension**	379 (12.4)	35 (14.2)	41 (4.9)	87 (8.4)

Smith LE, *et al. BMJ Open* 2023; 13:e070882. doi: 10.1136/bmjopen-2022-070882

New screen

ASK ALL

QC This is a quality control question.

What colour is grass usually?

SINGLE CODE, RANDOMISE ORDER

	General population	Savanta GBMSM	Grindr	Meta
Purple	0 (0.0)	0 (0.0)	0 (0.0)	0 (0.0)
Pink	0 (0.0)	0 (0.0)	0 (0.0)	0 (0.0)
Orange	0 (0.0)	0 (0.0)	0 (0.0)	0 (0.0)
Green	3050 (100.0)	247 (100.0)	831 (100.0)	1036 (100.0)
White	0 (0.0)	0 (0.0)	0 (0.0)	0 (0.0)
Black	0 (0.0)	0 (0.0)	0 (0.0)	0 (0.0)

SCREEN OUT if do not select "green".

Beliefs and attitudes

New screen

There is an infection called "monkeypox" that has affected some people in the UK. We are interested to know your personal opinion about monkeypox, based on what you currently know.

For each question, please select the answer that reflects your opinion about monkeypox. Do not worry if you do not know what the best answer might be or if you are not at all familiar with monkeypox. We only ask that you try to give your answer based on what you think you know or what you would honestly decide to do in the situations described. If you are really not sure, please answer "don't know".

New screen

Q1. Before today, how much have you seen or heard about monkeypox?

	General population	Savanta GBMSM	Grindr	Meta
I have not seen or heard anything	117 (3.8)	6 (2.4)	7 (0.8)	2 (0.2)
I have seen or heard a little	2164 (71.0)	136 (55.1)	385 (46.3)	287 (27.7)
I have seen or heard a lot	759 (24.9)	103 (41.7)	439 (52.8)	747 (72.1)
Don't know	10 (0.3)	2 (0.8)	0 (0.0)	0 (0.0)

New screen

ASK ALL

Q2. Overall, how worried are you about monkeypox?

SINGLE CODE

	General population	Savanta GBMSM	Grindr	Meta
Not at all worried	635 (20.8)	40 (16.2)	77 (9.3)	47 (4.5)
Not very worried	1763 (57.8)	125 (50.6)	469 (56.4)	500 (48.3)
Very worried	452 (14.8)	61 (24.7)	234 (28.2)	410 (39.6)
Extremely worried	129 (4.2)	13 (5.3)	38 (4.6)	72 (6.9)
Don't know	71 (2.3)	8 (3.2)	13 (1.6)	7 (0.7)

New screen

ASK ALL

Q3. How much risk do you think monkeypox currently poses to:

Please select one option for each answer

RANDOMISE order of presentation of statements

People in the UK?

	General population	Savanta GBMSM	Grindr	Meta
No risk at all	106 (3.5)	9 (3.6)	11 (1.3)	3 (0.3)
Low risk	1537 (50.4)	102 (41.3)	357 (43.0)	427 (41.2)
Medium risk	1058 (34.7)	97 (39.3)	337 (40.6)	426 (41.1)
High risk	186 (6.1)	28 (11.3)	92 (11.1)	153 (14.8)
Very high risk	75 (2.5)	6 (2.4)	17 (2.0)	22 (2.1)
Don't know	88 (2.9)	5 (2.0)	17 (2.0)	5 (0.5)

You personally?

	General population	Savanta GBMSM	Grindr	Meta
No risk at all	700 (23.0)	48 (19.4)	42 (5.1)	44 (4.2)
Low risk	1619 (53.1)	96 (38.9)	291 (35.0)	266 (25.7)
Medium risk	441 (14.5)	71 (28.7)	303 (36.5)	332 (32.0)
High risk	136 (4.5)	15 (6.1)	148 (17.8)	299 (28.9)
Very high risk	58 (1.9)	13 (5.3)	37 (4.5)	93 (9.0)
Don't know	96 (3.1)	4 (1.6)	10 (1.2)	2 (0.2)

New screen

ASK ALL

Q4. How much, if at all, have you seen or heard about monkeypox? From:

The news (TV, online news websites or apps, printed newspapers, or radio)

	General population	Savanta GBMSM	Grindr	Meta

Not at all	210 (6.9)	14 (5.7)	61 (7.3)	54 (5.2)
A little	2125 (69.7)	149 (60.3)	533 (64.1)	652 (62.9)
A lot	693 (22.7)	82 (33.2)	235 (28.3)	330 (31.9)
Don't know	22 (0.7)	2 (0.8)	2 (0.2)	0 (0.0)

Official websites or helplines (e.g. NHS, GOV.UK), or an NHS GP practice, clinic or hospital

	General population	Savanta GBMSM	Grindr	Meta
Not at all	1547 (50.7)	98 (39.7)	198 (23.8)	188 (18.1)
A little	1113 (36.5)	97 (39.3)	450 (54.2)	601 (58.0)
A lot	316 (10.4)	48 (19.4)	169 (20.3)	241 (23.3)
Don't know	74 (2.4)	4 (1.6)	14 (1.7)	6 (0.6)

Speaking to friends, family, colleagues or other people you know (in person, by phone, text, WhatsApp, email, or in other ways)

	General population	Savanta GBMSM	Grindr	Meta
Not at all	1469 (48.2)	111 (44.9)	263 (31.6)	218 (21.0)
A little	1235 (40.5)	87 (35.2)	388 (46.7)	466 (45.0)
A lot	300 (9.8)	44 (17.8)	175 (21.1)	350 (33.8)
Don't know	46 (1.5)	5 (2.0)	5 (0.6)	2 (0.2)

Workers in community groups, community or faith leaders, charities, or volunteers who help improve the health and wellbeing of others

	General population	Savanta GBMSM	Grindr	Meta
Not at all	2111 (69.2)	165 (66.8)	553 (66.5)	633 (61.1)
A little	633 (20.8)	53 (21.5)	162 (19.5)	253 (24.4)
A lot	220 (7.2)	25 (10.1)	58 (7.0)	113 (10.9)
Don't know	86 (2.8)	4 (1.6)	58 (7.0)	37 (3.6)

Other places, including blogs, social media sites (e.g. Facebook, Twitter, Instagram), online communities or other websites

	General population	Savanta GBMSM	Grindr	Meta
Not at all	1426 (46.8)	100 (40.5)	214 (25.8)	138 (13.3)
A little	1215 (39.8)	100 (40.5)	427 (51.4)	534 (51.5)
A lot	357 (11.7)	43 (17.4)	179 (21.5)	361 (34.8)
Don't know	52 (1.7)	4 (1.6)	11 (1.3)	3 (0.3)

New screen

Q4_other. Please state any other places that you have seen or heard about monkeypox from.
Open ended, text entry
None button

New screen

For each question, please select the answer that reflects your opinion about monkeypox. Do not worry if you do not know what the best answer might be or if you are not at all familiar with monkeypox. We only ask that you try to give your answer based on what you think you know. If you are really not sure, please answer "don't know".

New screen

ASK ALL
Q5. How much do you agree or disagree with the following statements:

RANDOMISE statements
I have a good idea of how people catch monkeypox

	General population	Savanta GBMSM	Grindr	Meta
Strongly disagree	252 (8.2)	14 (5.6)	27 (3.2)	15 (1.4)
Disagree	727 (23.8)	35 (14.1)	46 (5.5)	31 (2.9)
Neither agree nor disagree	590 (19.3)	32 (12.9)	82 (9.8)	57 (5.5)
Agree	1159 (38.0)	121 (48.9)	468 (56.3)	559 (53.9)
Strongly agree	217 (7.1)	41 (16.5)	196 (23.5)	371 (35.8)
Don't know	105 (3.4)	4 (1.6)	12 (1.4)	3 (0.2)

I know what the main symptoms of monkeypox are

	General population	Savanta GBMSM	Grindr	Meta
Strongly disagree	263 (8.6)	16 (6.5)	38 (4.6)	17 (1.6)
Disagree	820 (26.9)	44 (17.8)	100 (12.0)	60 (5.8)
Neither agree nor disagree	590 (19.3)	41 (16.6)	106 (12.8)	108 (10.4)
Agree	1113 (36.5)	120 (48.6)	493 (59.3)	642 (62.0)
Strongly agree	152 (5.0)	23 (9.3)	80 (9.6)	204 (19.7)
Don't know	112 (3.7)	3 (1.2)	14 (1.7)	5 (0.5)

It would be easy for me to tell if someone I meet has monkeypox

	General population	Savanta GBMSM	Grindr	Meta
Strongly disagree	485 (15.9)	33 (13.4)	149 (17.9)	205 (19.8)
Disagree	1123 (36.8)	71 (28.7)	369 (44.4)	498 (48.1)
Neither agree nor disagree	663 (21.7)	64 (25.9)	181 (21.8)	209 (20.2)
Agree	504 (16.5)	54 (21.9)	88 (10.6)	92 (8.9)
Strongly agree	119 (3.9)	14 (5.7)	15 (1.8)	15 (1.4)
Don't know	156 (5.1)	11 (4.5)	29 (3.5)	17 (1.6)

New screen

ASK ALL

Q6. How much do you agree or disagree with the following statements:

RANDOMISE statements

In the near future, it is likely that some of the people I come into physical contact with (touch) will have monkeypox

	General population	Savanta GBMSM	Grindr	Meta
Strongly disagree	275 (9.0)	13 (5.3)	23 (2.8)	23 (2.2)
Disagree	743 (24.4)	56 (22.7)	144 (17.3)	145 (14.0)
Neither agree nor disagree	912 (29.9)	65 (26.3)	237 (28.5)	238 (23.0)
Agree	632 (20.7)	73 (29.6)	297 (35.7)	470 (45.4)
Strongly agree	112 (3.7)	12 (4.9)	43 (5.2)	118 (11.4)
Don't know	376 (12.3)	28 (11.3)	87 (10.5)	42 (4.1)

If I come into physical contact with (touch) someone who has monkeypox, it is likely that I will catch it

	General population	Savanta GBMSM	Grindr	Meta
Strongly disagree	108 (3.5)	5 (2.0)	20 (2.4)	9 (0.9)
Disagree	467 (15.3)	24 (9.7)	93 (11.2)	145 (14.0)
Neither agree nor disagree	814 (26.7)	59 (23.9)	167 (20.1)	207 (20.0)
Agree	944 (31.0)	99 (40.1)	376 (45.2)	497 (48.0)
Strongly agree	207 (6.8)	25 (10.1)	98 (11.8)	135 (13.0)
Don't know	510 (16.7)	35 (14.2)	77 (9.3)	43 (4.2)

Monkeypox would be a serious illness for me

	General population	Savanta GBMSM	Grindr	Meta
Strongly disagree	224 (7.3)	16 (6.5)	30 (3.6)	26 (2.5)
Disagree	546 (17.9)	35 (14.2)	144 (17.3)	160 (15.4)
Neither agree nor disagree	744 (24.4)	58 (23.5)	182 (21.9)	220 (21.2)
Agree	787 (25.8)	85 (34.4)	290 (34.9)	413 (39.9)
Strongly agree	274 (9.0)	28 (11.3)	116 (14.0)	166 (16.0)
Don't know	475 (15.6)	25 (10.1)	69 (8.3)	51 (4.9)

People who catch monkeypox usually make a full recovery, even if they do not receive any treatment

	General population	Savanta GBMSM	Grindr	Meta
Strongly disagree	52 (1.7)	0 (0.0)	11 (1.3)	6 (0.6)
Disagree	264 (8.7)	25 (10.1)	77 (9.3)	62 (6.0)
Neither agree nor disagree	844 (27.7)	56 (22.7)	117 (14.1)	144 (13.9)
Agree	977 (32.0)	96 (38.9)	383 (46.1)	530 (51.2)

Strongly agree	228 (7.5)	29 (11.7)	102 (12.3)	210 (20.3)
Don't know	685 (22.5)	41 (16.6)	141 (17.0)	84 (8.1)

My personal behaviour has an impact on how monkeypox spreads

	General population	Savanta GBMSM	Grindr	Meta
Strongly disagree	498 (16.3)	24 (9.7)	53 (6.4)	28 (2.7)
Disagree	581 (19.0)	31 (12.6)	78 (9.4)	71 (6.9)
Neither agree nor disagree	572 (18.8)	40 (16.2)	91 (11.0)	67 (6.5)
Agree	834 (27.3)	95 (38.5)	384 (46.2)	552 (53.3)
Strongly agree	278 (9.1)	45 (18.2)	191 (23.0)	312 (30.1)
Don't know	287 (9.4)	12 (4.9)	34 (4.1)	6 (0.6)

My life has been negatively affected by changes made in response to the monkeypox outbreak

	General population	Savanta GBMSM	Grindr	Meta
Strongly disagree	1181 (38.7)	90 (36.4)	167 (20.1)	160 (15.4)
Disagree	981 (32.2)	75 (30.4)	289 (34.8)	300 (29.0)
Neither agree nor disagree	420 (13.8)	37 (15.0)	168 (20.2)	217 (20.9)
Agree	246 (8.1)	24 (9.7)	144 (17.3)	263 (25.4)
Strongly agree	115 (3.8)	12 (4.9)	42 (5.1)	88 (8.5)
Don't know	107 (3.5)	9 (3.6)	21 (2.5)	8 (0.8)

New screen

Below are some claims that some people have made about monkeypox. For each one, please say whether you strongly disagree, disagree, neither agree nor disagree, agree, or strongly agree.

ASK ALL

Q7. How much do you agree or disagree with the following claims:

SINGLE CODE

RANDOMISE statements

The risks of monkeypox are being exaggerated

	General population	Savanta GBMSM	Grindr	Meta
Strongly disagree	170 (5.6)	23 (9.3)	99 (11.9)	175 (16.9)
Disagree	822 (27.0)	87 (35.2)	326 (39.2)	505 (48.7)
Neither agree nor disagree	998 (32.7)	65 (26.3)	221 (26.6)	214 (20.7)
Agree	529 (17.3)	39 (15.8)	89 (10.7)	89 (8.6)
Strongly agree	184 (6.0)	11 (4.5)	34 (4.1)	19 (1.8)
Don't know	347 (11.4)	22 (8.9)	62 (7.5)	34 (3.3)

Because of the monkeypox outbreak, it is best to avoid physical contact with (touch) men who are gay, bisexual or have sex with men

	General population	Savanta GBMSM	Grindr	Meta
Strongly disagree	422 (13.8)	50 (20.2)	237 (28.5)	294 (28.4)
Disagree	707 (23.2)	64 (25.9)	267 (32.1)	357 (34.5)
Neither agree nor disagree	667 (21.9)	44 (17.8)	147 (17.7)	172 (16.6)
Agree	611 (20.0)	51 (20.6)	132 (15.9)	161 (15.5)
Strongly agree	305 (10.0)	22 (8.9)	22 (2.6)	37 (3.6)
Don’t know	338 (11.1)	16 (6.5)	26 (3.1)	15 (1.4)

Because of the monkeypox outbreak, it is best to avoid physical contact with (touch) people from Africa

	General population	Savanta GBMSM	Grindr	Meta
Strongly disagree	462 (15.1)	52 (21.1)	327 (39.4)	431 (41.6)
Disagree	845 (27.7)	69 (27.9)	273 (32.9)	399 (38.5)
Neither agree nor disagree	768 (25.2)	57 (23.1)	118 (14.2)	127 (12.3)
Agree	454 (14.9)	40 (16.2)	59 (7.1)	43 (4.2)
Strongly agree	175 (5.7)	10 (4.0)	14 (1.7)	11 (1.1)
Don’t know	346 (11.3)	19 (7.7)	40 (4.8)	25 (2.4)

Monkeypox is only a risk to men who are gay, bisexual or have sex with men

	General population	Savanta GBMSM	Grindr	Meta
Strongly disagree	536 (17.6)	77 (31.2)	375 (45.1)	431 (41.6)
Disagree	909 (29.8)	75 (30.4)	302 (36.3)	449 (43.3)
Neither agree nor disagree	586 (19.2)	35 (14.2)	56 (6.7)	63 (6.1)
Agree	521 (17.1)	30 (12.1)	61 (7.3)	65 (6.3)
Strongly agree	161 (5.3)	15 (6.1)	14 (1.7)	21 (2.0)
Don’t know	337 (11.0)	15 (6.1)	23 (2.8)	7 (0.7)

New screen

ASK ALL
Q8. What do you think the main symptoms of monkeypox are?
Please select up to 4
MULTICODE – MAX 4
RANDOMISE

	General population	Savanta GBMSM	Grindr	Meta
High temperature / fever	1081 (35.4)	105 (42.5)	445 (53.5)	632 (61.0)

Shivering / chills	501 (16.4)	35 (14.2)	151 (18.2)	232 (22.4)
Muscle aches	542 (17.8)	47 (19.0)	249 (30.0)	339 (32.7)
Pain in your arms, legs or joints	385 (12.6)	45 (18.2)	117 (14.1)	170 (16.4)
Backache	104 (3.4)	10 (4.0)	19 (2.3)	23 (2.2)
Headache	426 (14.0)	39 (15.8)	146 (17.6)	181 (17.5)
Exhaustion	320 (10.5)	37 (15)	169 (20.3)	226 (21.8)
Swollen glands	496 (16.3)	68 (27.5)	256 (30.8)	446 (43.1)
Unexplained rash with blisters	1688 (55.3)	147 (59.5)	647 (77.9)	872 (84.2)
Unexplained rash without blisters	632 (20.7)	65 (26.3)	198 (23.8)	257 (24.8)
Runny or blocked nose	127 (4.2)	5 (2.0)	16 (1.9)	10 (1.0)
Dizziness	160 (5.2)	12 (4.9)	23 (2.8)	18 (1.7)
Sore throat	281 (9.2)	30 (12.1)	72 (8.7)	88 (8.5)
Nausea / feeling sick, or vomiting	388 (12.7)	46 (18.6)	68 (8.2)	77 (7.4)
Diarrhoea	182 (6)	17 (6.9)	31 (3.7)	32 (3.1)
Other, please state [ANCHOR]	5 (0.2)	0 (0.0)	9 (1.1)	12 (1.2)
Don't know [ANCHOR, EXCLUSIVE]	562 (18.4)	23 (9.3)	48 (5.8)	25 (2.4)

†Participants could select up to four answers to this question, therefore column totals add to more than 100%.

New screen

ASK ALL

Q9. You can catch monkeypox if:

Please select one option for each answer

RANDOMISE statements

You touch the rash of a person who has monkeypox

	General population	Savanta GBMSM	Grindr	Meta
Strongly disagree	68 (2.2)	3 (1.2)	11 (1.3)	6 (0.6)
Disagree	176 (5.8)	10 (4.0)	23 (2.8)	15 (1.4)
Neither agree nor disagree	516 (16.9)	28 (11.3)	48 (5.8)	38 (3.7)
Agree	1326 (43.5)	112 (45.3)	394 (47.4)	424 (40.9)
Strongly agree	394 (12.9)	65 (26.3)	293 (35.3)	519 (50.1)
Don’t know	570 (18.7)	29 (11.7)	62 (7.5)	34 (3.3)

You are coughed or sneezed on by a person who has monkeypox

	General	Savanta GBMSM	Grindr	Meta

	population			
Strongly disagree	82 (2.7)	17 (6.9)	50 (6.0)	82 (7.9)
Disagree	356 (11.7)	39 (15.8)	191 (23.0)	253 (24.4)
Neither agree nor disagree	634 (20.8)	41 (16.6)	127 (15.3)	160 (15.4)
Agree	1024 (33.6)	87 (35.2)	251 (30.2)	344 (33.2)
Strongly agree	273 (9.0)	22 (8.9)	74 (8.9)	82 (7.9)
Don't know	681 (22.3)	41 (16.6)	138 (16.6)	115 (11.1)

You touch a person who has monkeypox, even if they do not have a rash

	General population	Savanta GBMSM	Grindr	Meta
Strongly disagree	91 (3.0)	4 (1.6)	26 (3.1)	20 (1.9)
Disagree	419 (13.7)	28 (11.3)	139 (16.7)	189 (18.2)
Neither agree nor disagree	634 (20.8)	61 (24.7)	125 (15.0)	198 (19.1)
Agree	932 (30.6)	91 (36.8)	337 (40.6)	410 (39.6)
Strongly agree	237 (7.8)	24 (9.7)	73 (8.8)	95 (9.2)
Don't know	737 (24.2)	39 (15.8)	131 (15.8)	124 (12)

You have sex with someone who has monkeypox

	General population	Savanta GBMSM	Grindr	Meta
Strongly disagree	72 (2.4)	5 (2.0)	12 (1.4)	10 (1.0)
Disagree	99 (3.2)	4 (1.6)	5 (0.6)	7 (0.7)
Neither agree nor disagree	359 (11.8)	17 (6.9)	24 (2.9)	16 (1.5)
Agree	1293 (42.4)	120 (48.6)	387 (46.6)	441 (42.6)
Strongly agree	802 (26.3)	89 (36.0)	377 (45.4)	551 (53.2)
Don't know	425 (13.9)	12 (4.9)	26 (3.1)	11 (1.1)

You come within 1 metre of someone who has monkeypox

	General population	Savanta GBMSM	Grindr	Meta
Strongly disagree	357 (11.7)	59 (23.9)	269 (32.4)	407 (39.3)
Disagree	972 (31.9)	93 (37.7)	379 (45.6)	438 (42.3)
Neither agree nor disagree	598 (19.6)	29 (11.7)	72 (8.7)	84 (8.1)
Agree	358 (11.7)	34 (13.8)	39 (4.7)	50 (4.8)
Strongly agree	131 (4.3)	4 (1.6)	10 (1.2)	11 (1.1)
Don't know	634 (20.8)	28 (11.3)	62 (7.5)	46 (4.4)

You touch something (e.g. towels, bedding or clothing) that has been touched by a person who has monkeypox

	General population	Savanta GBMSM	Grindr	Meta
Strongly disagree	87 (2.9)	7 (2.8)	21 (2.5)	18 (1.7)

Disagree	414 (13.6)	30 (12.1)	85 (10.2)	72 (6.9)
Neither agree nor disagree	653 (21.4)	46 (18.6)	100 (12.0)	89 (8.6)
Agree	961 (31.5)	96 (38.9)	379 (45.6)	585 (56.5)
Strongly agree	248 (8.1)	35 (14.2)	142 (17.1)	204 (19.7)
Don't know	687 (22.5)	33 (13.4)	104 (12.5)	68 (6.6)

You touch a pet animal that has monkeypox

	General population	Savanta GBMSM	Grindr	Meta
Strongly disagree	130 (4.3)	14 (5.7)	61 (7.3)	67 (6.5)
Disagree	469 (15.4)	43 (17.4)	140 (16.8)	151 (14.6)
Neither agree nor disagree	693 (22.7)	57 (23.1)	159 (19.1)	208 (20.1)
Agree	740 (24.3)	62 (25.1)	219 (26.4)	341 (32.9)
Strongly agree	193 (6.3)	24 (9.7)	46 (5.5)	82 (7.9)
Don't know	825 (27.0)	47 (19)	206 (24.8)	187 (18.1)

Intended behaviour

Motivational messaging

New screen

[For Savanta gen pop sample, randomise people to four groups. Ppts should see EITHER group A OR group B OR group C OR group D.
For Savanta GBMSM, Grindr, Meta samples, randomise people to two groups. Ppts should see EITHER group E OR group F.]

[Savanta gen pop] GROUP A (risk + perceived necessity/efficacy): Monkeypox causes mild illness for most people, but it can be very severe and can even kill some people. Pets may also be able to catch monkeypox. People who have recently been in contact with someone who might have monkeypox and get an unexplained rash should get tested quickly. If they test positive, they should avoid close contact with others to stop monkeypox from spreading further.

[Savanta gen pop] GROUP B (risk + perceived benefits): Monkeypox causes mild illness for most people, but it can be very severe and can even kill some people. Pets may also be able to catch monkeypox. People who test positive for monkeypox can take action to protect their friends, family, and pets from infection.

[Savanta gen pop] GROUP C (risk + perceived costs): Monkeypox causes mild illness for most people, but it can be very severe and can even kill some people. Pets may also be able to catch monkeypox. Imagine that people who have to self-isolate because they test positive for monkeypox will be provided with the practical and financial support that they need to do that.

Smith LE, *et al. BMJ Open* 2023; 13:e070882. doi: 10.1136/bmjopen-2022-070882

[Savanta gen pop] GROUP D (control message): Monkeypox is a rare infection that is mainly found in parts of west or central Africa. Recently, there has been an outbreak of monkeypox in other countries. Since early May, more than 40,000 monkeypox cases have been reported in over 80 countries where the virus is not normally seen, including the UK.

[Savanta GBMSM / Grindr / Meta] GROUP E (all messages): Monkeypox causes mild illness for most people, but it can be very severe and can even kill some people. Pets may also be able to catch monkeypox.

People who have recently been in contact with someone who might have monkeypox and get an unexplained rash should get tested quickly. If they test positive, they should avoid close contact with others to stop monkeypox from spreading further. People who test positive for monkeypox can take action to protect their friends, family, and pets from infection. Imagine that people who have to self-isolate because they test positive for monkeypox will be provided with the practical and financial support that they need to do that.

[Savanta GBMSM / Grindr / Meta] GROUP F (control message): Monkeypox is a rare infection that is mainly found in parts of west or central Africa. Recently, there has been an outbreak of monkeypox in other countries.

There is a new outbreak of monkeypox in the UK. Since early May, more than 40,000 monkeypox cases have been reported in over 80 countries where the virus is not normally seen, including the UK. The current outbreak is the first time that the virus has been passed from person to person in the UK, and so the UK Health Security Agency (UKHSA) is working with partners to put in place guidance and measures that will prevent the spread of infection.

New screen

Behaviours

Self-isolation

People who have monkeypox are being asked to self-isolate for 21 days (3 weeks). They are also being asked to tell public health officials about other people they have been in contact with.

Self-isolation means staying at home and not going to work, school or public areas. It means avoiding close contact with people you live with, spending time in separate rooms as much as possible, and not having visitors to your home.

[Randomise order of Q10 and Q11]

New screen

ASK ALL

Q10. Imagine you are contacted by public health officials after testing and told that **you have monkeypox**. They tell you that you need to self-isolate for 21 days.

Realistically, would you self-isolate for 21 days?
SINGLE CODE
Answer Options

	General population	Savanta GBMSM	Grindr	Meta
Definitely would not	106 (3.5)	10 (4.0)	56 (6.7)	41 (4.0)
Probably would not	283 (9.3)	24 (9.7)	116 (14.0)	144 (13.9)
Not sure	367 (12.0)	19 (7.7)	74 (8.9)	90 (8.7)
Probably would	799 (26.2)	76 (30.8)	260 (31.3)	327 (31.6)
Definitely would	1495 (49.0)	118 (47.8)	325 (39.1)	434 (41.9)

New screen

ASK ALL
Q11. Imagine you are contacted by public health officials and told that **you have come into high-risk contact with someone who has monkeypox**. They tell you that you need to self-isolate for 21 days.

Realistically, would you self-isolate for 21 days?
SINGLE CODE
Answer Options

	General population	Savanta GBMSM	Grindr	Meta
Definitely would not	126 (4.1)	15 (6.1)	78 (9.4)	70 (6.8)
Probably would not	341 (11.2)	38 (15.4)	169 (20.3)	249 (24.0)
Not sure	483 (15.8)	24 (9.7)	124 (14.9)	172 (16.6)
Probably would	907 (29.7)	77 (31.2)	261 (31.4)	322 (31.1)
Definitely would	1193 (39.1)	93 (37.7)	199 (23.9)	223 (21.5)

New screen

ASK ALL
Q12. What would stop you from being able to self-isolate for 21 days?

Please select all that apply
MULTI CODE, RANDOMISE ORDER
Answer Options

	General population	Savanta GBMSM	Grindr	Meta
I would need to go out to get food, medicines, or other essentials	992 (32.5)	119 (48.2)	477 (57.4)	604 (58.3)
I would need to go out to work	542 (17.8)	52 (21.1)	309 (37.2)	323 (31.2)
I would need to go out to provide help or care to friends or relatives	238 (7.8)	22 (8.9)	53 (6.4)	72 (6.9)

I would need to go out as part of looking after my children (e.g. to take them to school, or a playground)	396 (13.0)	9 (3.6)	21 (2.5)	15 (1.4)
I would need to go out for a walk or some other exercise	765 (25.1)	72 (29.1)	388 (46.7)	550 (53.1)
My home is too small	192 (6.3)	17 (6.9)	103 (12.4)	109 (10.5)
I would be too bored or lonely	344 (11.3)	39 (15.8)	170 (20.5)	242 (23.4)
I don't think it's necessary for me to self-isolate	207 (6.8)	12 (4.9)	54 (6.5)	85 (8.2)
Other, please state	40 (1.3)	3 (1.2)	40 (4.8)	52 (5.0)
Nothing, I would stay at home for 21 days [EXCLUSIVE]	1166 (38.2)	76 (30.8)	153 (18.4)	185 (17.9)

†Participants could select more than one item for this question, therefore column totals add to more than 100%.

New screen

Now we would like you to think about a different scenario.

Help seeking

New screen

Imagine that tomorrow morning **you develop an unexplained rash with blisters**. You also learn that **you have come into contact with someone who might have monkeypox**.

ASK ALL
Q13 If **you** got these symptoms, which of these things would you do?

RANDOMISE order

Wait a day or two to see if they get better or clear up on their own

	General population	Savanta GBMSM	Grindr	Meta
Definitely would not	344 (11.3)	27 (10.9)	130 (15.6)	195 (18.8)
Probably would not	546 (17.9)	47 (19.0)	175 (21.1)	248 (23.9)
Not sure	809 (26.5)	58 (23.5)	145 (17.4)	217 (20.9)
Probably would	1080 (35.4)	92 (37.2)	323 (38.9)	326 (31.5)

Definitely would	271 (8.9)	23 (9.3)	58 (7.0)	50 (4.8)

Try to book an appointment with a GP

	General population	Savanta GBMSM	Grindr	Meta
Definitely would not	307 (10.1)	28 (11.3)	154 (18.5)	183 (17.7)
Probably would not	409 (13.4)	48 (19.4)	167 (20.1)	295 (28.5)
Not sure	586 (19.2)	40 (16.2)	125 (15.0)	148 (14.3)
Probably would	1032 (33.8)	86 (34.8)	221 (26.6)	222 (21.4)
Definitely would	716 (23.5)	45 (18.2)	164 (19.7)	188 (18.1)

Visit a Pharmacist/Chemist for advice

	General population	Savanta GBMSM	Grindr	Meta
Definitely would not	634 (20.8)	49 (19.8)	284 (34.2)	393 (37.9)
Probably would not	749 (24.6)	69 (27.9)	329 (39.6)	438 (42.3)
Not sure	727 (23.8)	64 (25.9)	97 (11.7)	125 (12.1)
Probably would	701 (23.0)	48 (19.4)	88 (10.6)	63 (6.1)
Definitely would	239 (7.8)	17 (6.9)	33 (4.0)	17 (1.6)

Go to A&E or visit another NHS service such as a walk-in centre or minor injuries unit

	General population	Savanta GBMSM	Grindr	Meta
Definitely would not	776 (25.4)	60 (24.3)	309 (37.2)	436 (42.1)
Probably would not	841 (27.6)	66 (26.7)	274 (33.0)	365 (35.2)
Not sure	682 (22.4)	54 (21.9)	110 (13.2)	113 (10.9)
Probably would	519 (17.0)	52 (21.1)	93 (11.2)	87 (8.4)
Definitely would	232 (7.6)	15 (6.1)	45 (5.4)	35 (3.4)

Call NHS 111 or 999 / ambulance service

	General population	Savanta GBMSM	Grindr	Meta
Definitely would not	246 (8.1)	22 (8.9)	145 (17.4)	185 (17.9)
Probably would not	358 (11.7)	30 (12.1)	153 (18.4)	233 (22.5)
Not sure	556 (18.2)	36 (14.6)	108 (13.0)	118 (11.4)
Probably would	1084 (35.5)	95 (38.5)	243 (29.2)	283 (27.3)
Definitely would	806 (26.4)	64 (25.9)	182 (21.9)	217 (20.9)

Visit a walk-in sexual health service

	General population	Savanta GBMSM	Grindr	Meta
Definitely would not	906 (29.7)	50 (20.2)	164 (19.7)	207 (20.0)

Smith LE, *et al. BMJ Open* 2023; 13:e070882. doi: 10.1136/bmjopen-2022-070882

Probably would not	783 (25.7)	56 (22.7)	192 (23.1)	203 (19.6)
Not sure	727 (23.8)	51 (20.6)	125 (15.0)	130 (12.5)
Probably would	454 (14.9)	65 (26.3)	205 (24.7)	262 (25.3)
Definitely would	180 (5.9)	25 (10.1)	145 (17.4)	234 (22.6)

Call a sexual health service [fix below 6]

	General population	Savanta GBMSM	Grindr	Meta
Definitely would not	692 (22.7)	27 (10.9)	33 (4.0)	25 (2.4)
Probably would not	690 (22.6)	38 (15.4)	68 (8.2)	57 (5.5)
Not sure	697 (22.9)	35 (14.2)	60 (7.2)	60 (5.8)
Probably would	630 (20.7)	87 (35.2)	278 (33.5)	320 (30.9)
Definitely would	341 (11.2)	60 (24.3)	392 (47.2)	574 (55.4)

Let people you have recently been in close contact with know that you have symptoms

	General population	Savanta GBMSM	Grindr	Meta
Definitely would not	81 (2.7)	8 (3.2)	34 (4.1)	32 (3.1)
Probably would not	220 (7.2)	20 (8.1)	75 (9.0)	90 (8.7)
Not sure	495 (16.2)	47 (19.0)	152 (18.3)	177 (17.1)
Probably would	1185 (38.9)	96 (38.9)	298 (35.9)	396 (38.2)
Definitely would	1069 (35)	76 (30.8)	272 (32.7)	341 (32.9)

Search for information from official websites (e.g. NHS, GOV.UK)

	General population	Savanta GBMSM	Grindr	Meta
Definitely would not	73 (2.4)	4 (1.6)	13 (1.6)	9 (0.9)
Probably would not	122 (4.0)	10 (4.0)	26 (3.1)	23 (2.2)
Not sure	325 (10.7)	22 (8.9)	30 (3.6)	24 (2.3)
Probably would	1165 (38.2)	91 (36.8)	281 (33.8)	366 (35.3)
Definitely would	1365 (44.8)	120 (48.6)	481 (57.9)	614 (59.3)

Search for information from other places, including blogs, social media sites (e.g. Facebook, Twitter, Instagram), online communities or other websites [fix below 9]

	General population	Savanta GBMSM	Grindr	Meta
Definitely would not	533 (17.5)	49 (19.8)	144 (17.3)	135 (13.0)
Probably would not	647 (21.2)	54 (21.9)	231 (27.8)	277 (26.7)
Not sure	647 (21.2)	39 (15.8)	113 (13.6)	155 (15.0)
Probably would	789 (25.9)	70 (28.3)	206 (24.8)	301 (29.1)
Definitely would	434 (14.2)	35 (14.2)	137 (16.5)	168 (16.2)

Smith LE, *et al. BMJ Open* 2023; 13:e070882. doi: 10.1136/bmjopen-2022-070882

New screen

Imagine that tomorrow morning **you develop an unexplained rash with blisters**. You also learn that **you have come into contact with someone who might have monkeypox**.

Q13_other. Please state any other things you would do.
Open ended text
Nothing button

Contact behaviour

New screen

Imagine that tomorrow morning **you develop an unexplained rash with blisters**. You also learn that **you have come into contact with someone who might have monkeypox**.

ASK ALL
Q14 If **you** got these symptoms, in the following 21 days, realistically how much, if at all, would you:

RANDOMISE order

Come into skin-to-skin contact with other people

	General population	Savanta GBMSM	Grindr	Meta
I would completely stop doing this	1883 (61.7)	153 (61.9)	648 (78)	858 (82.8)
Less than normal	412 (13.5)	41 (16.6)	127 (15.3)	135 (13.0)
Same as normal	249 (8.2)	21 (8.5)	15 (1.8)	12 (1.2)
More than normal	107 (3.5)	8 (3.2)	3 (0.4)	1 (0.1)
Not applicable, I wouldn't do this anyway	280 (9.2)	16 (6.5)	22 (2.6)	21 (2.0)
Don't know	109 (3.6)	7 (2.8)	15 (1.8)	9 (0.9)
Prefer not to say	10 (0.3)	1 (0.4)	1 (0.1)	0 (0.0)

Have sexual contact (from kissing to intercourse) with other people

	General population	Savanta GBMSM	Grindr	Meta
I would completely stop doing this	1923 (63.0)	171 (69.2)	721 (86.8)	922 (89.0)
Less than normal	238 (7.8)	20 (8.1)	54 (6.5)	61 (5.9)
Same as normal	216 (7.1)	15 (6.1)	14 (1.7)	7 (0.7)
More than normal	113 (3.7)	10 (4.0)	1 (0.1)	1 (0.1)
Not applicable, I wouldn't do this anyway	451 (14.8)	24 (9.7)	27 (3.2)	30 (2.9)
Don't know	93 (3.0)	6 (2.4)	13 (1.6)	13 (1.3)
Prefer not to say	16 (0.5)	1 (0.4)	1 (0.1)	2 (0.2)

Smith LE, *et al. BMJ Open* 2023; 13:e070882. doi: 10.1136/bmjopen-2022-070882

Have sex without using a condom

	General population	Savanta GBMSM	Grindr	Meta
I would completely stop doing this	1729 (56.7)	155 (62.8)	623 (75)	788 (76.1)
Less than normal	146 (4.8)	17 (6.9)	33 (4.0)	37 (3.6)
Same as normal	235 (7.7)	15 (6.1)	27 (3.2)	29 (2.8)
More than normal	124 (4.1)	11 (4.5)	3 (0.4)	2 (0.2)
Not applicable, I wouldn't do this anyway	680 (22.3)	42 (17.0)	123 (14.8)	161 (15.5)
Don't know	111 (3.6)	7 (2.8)	17 (2.0)	14 (1.4)
Prefer not to say	25 (0.8)	0 (0.0)	5 (0.6)	5 (0.5)

Share bedding, towels, or clothes with other people

	General population	Savanta GBMSM	Grindr	Meta
I would completely stop doing this	1839 (60.3)	147 (59.5)	604 (72.7)	807 (77.9)
Less than normal	286 (9.4)	23 (9.3)	62 (7.5)	96 (9.3)
Same as normal	279 (9.1)	21 (8.5)	24 (2.9)	19 (1.8)
More than normal	124 (4.1)	12 (4.9)	2 (0.2)	1 (0.1)
Not applicable, I wouldn't do this anyway	415 (13.6)	41 (16.6)	126 (15.2)	104 (10.0)
Don't know	98 (3.2)	3 (1.2)	12 (1.4)	8 (0.8)
Prefer not to say	9 (0.3)	0 (0.0)	1 (0.1)	1 (0.1)

Go to a crowded place

	General population	Savanta GBMSM	Grindr	Meta
I would completely stop doing this	1872 (61.4)	141 (57.1)	552 (66.4)	663 (64.0)
Less than normal	418 (13.7)	50 (20.2)	183 (22)	297 (28.7)
Same as normal	248 (8.1)	23 (9.3)	57 (6.9)	39 (3.8)
More than normal	123 (4.0)	9 (3.6)	1 (0.1)	1 (0.1)
Not applicable, I wouldn't do this anyway	275 (9.0)	16 (6.5)	25 (3.0)	23 (2.2)
Don't know	103 (3.4)	8 (3.2)	13 (1.6)	12 (1.2)
Prefer not to say	11 (0.4)	0 (0.0)	0 (0.0)	1 (0.1)

Help or provide care for a vulnerable person

	General population	Savanta GBMSM	Grindr	Meta
I would completely stop doing this	1674 (54.9)	131 (53.0)	516 (62.1)	686 (66.2)
Less than normal	304 (10.0)	16 (6.5)	81 (9.7)	81 (7.8)
Same as normal	255 (8.4)	24 (9.7)	23 (2.8)	24 (2.3)
More than normal	160 (5.2)	14 (5.7)	8 (1.0)	6 (0.6)
Not applicable, I wouldn't do this anyway	513 (16.8)	50 (20.2)	181 (21.8)	217 (20.9)

Smith LE, *et al. BMJ Open* 2023; 13:e070882. doi: 10.1136/bmjopen-2022-070882

Don't know	134 (4.4)	11 (4.5)	22 (2.6)	20 (1.9)
Prefer not to say	10 (0.3)	1 (0.4)	0 (0.0)	2 (0.2)

Go to a public place where you may come into physical contact with (touch) someone else

	General population	Savanta GBMSM	Grindr	Meta
I would completely stop doing this	1818 (59.6)	146 (59.1)	525 (63.2)	653 (63.0)
Less than normal	469 (15.4)	51 (20.6)	236 (28.4)	318 (30.7)
Same as normal	249 (8.2)	20 (8.1)	33 (4.0)	24 (2.3)
More than normal	143 (4.7)	9 (3.6)	2 (0.2)	2 (0.2)
Not applicable, I wouldn't do this anyway	259 (8.5)	11 (4.5)	24 (2.9)	24 (2.3)
Don't know	104 (3.4)	9 (3.6)	10 (1.2)	14 (1.4)
Prefer not to say	8 (0.3)	1 (0.4)	1 (0.1)	1 (0.1)

New screen

Imagine that tomorrow morning **you develop an unexplained rash with blisters**. You also learn that **you have come into contact with someone who might have monkeypox**.

Q15 How many days after the start of these symptoms would you:

Please give an approximate number if you are unsure. If you would not do this, please put 0.
Type your answer below

			General population	Savanta GBMSM	Grindr	Meta
Have sexual contact (from kissing to intercourse) with someone else	OPEN TEXT ENTRY – number [cap at 100]	ASK ONLY to those who answer 1-4 for similar question above	N=2021, M=15.7, SD=15.3, range 0 to 100.	N=190, M=16.2, SD=14.4, range 0 to 100.	N=746, M=20.4, SD=15.8, range 0 to 100.	N=947, M=20.0, SD=12.3, range 0 to 100.
Prefer not to say			469 (18.8)	26 (12.0)	44 (5.6)	44 (4.4)
Have sex without using a condom	OPEN TEXT ENTRY – number [cap at 100]	ASK ONLY to those who answer 1-4 for similar question above	N=1807, M=15.7, SD=16.8, range 0 to 100.	N=175, M=16.8, SD=18.9, range 0 to 100.	N=649, M=21.2, SD=19.5, range 0 to 100.	N=821, M=21.3, SD=18.0, range 0 to 100.
Prefer not to say			427 (19.1)	23 (11.6)	37 (5.4)	35 (4.1)

Contact sharing

Smith LE, *et al. BMJ Open* 2023; 13:e070882. doi: 10.1136/bmjopen-2022-070882

New screen

People with suspected or confirmed monkeypox are being asked by public health officials to share contact details of people they have been in close contact with, so that they can be offered appropriate care and advice. Details of contacts are kept completely confidential.

ASK ALL

Q16 If **you** were asked for this information, would you try to share all the contact details of every …

RANDOMISE statements

Person who had been in your home (household members and visitors) in the last seven days

	General population	Savanta GBMSM	Grindr	Meta
Definitely would not	135 (4.4)	14 (5.7)	51 (6.1)	56 (5.4)
Probably would not	168 (5.5)	19 (7.7)	101 (12.2)	118 (11.4)
Not sure	392 (12.9)	30 (12.1)	97 (11.7)	120 (11.6)
Probably would	758 (24.9)	60 (24.3)	247 (29.7)	325 (31.4)
Definitely would	1552 (50.9)	120 (48.6)	331 (39.8)	414 (40.0)
Prefer not to say	45 (1.5)	4 (1.6)	4 (0.5)	3 (0.3)

Person you had sexual contact with (from kissing to intercourse) in the last seven days

	General population	Savanta GBMSM	Grindr	Meta
Definitely would not	169 (5.5)	17 (6.9)	49 (5.9)	54 (5.2)
Probably would not	162 (5.3)	16 (6.5)	59 (7.1)	67 (6.5)
Not sure	366 (12.0)	27 (10.9)	59 (7.1)	83 (8.0)
Probably would	629 (20.6)	56 (22.7)	236 (28.4)	282 (27.2)
Definitely would	1671 (54.8)	127 (51.4)	420 (50.5)	545 (52.6)
Prefer not to say	53 (1.7)	4 (1.6)	8 (1.0)	5 (0.5)

Place you had sexual contact with someone (from kissing to intercourse) in the last seven days

	General population	Savanta GBMSM	Grindr	Meta
Definitely would not	165 (5.4)	17 (6.9)	51 (6.1)	52 (5)
Probably would not	188 (6.2)	18 (7.3)	63 (7.6)	72 (6.9)
Not sure	415 (13.6)	28 (11.3)	72 (8.7)	91 (8.8)
Probably would	655 (21.5)	64 (25.9)	261 (31.4)	306 (29.5)
Definitely would	1559 (51.1)	116 (47)	375 (45.1)	510 (49.2)
Prefer not to say	68 (2.2)	4 (1.6)	9 (1.1)	5 (0.5)

Person you had skin-to-skin contact with (including hugging or kissing) in the last seven days

	General	Savanta	Grindr	Meta

Smith LE, *et al. BMJ Open* 2023; 13:e070882. doi: 10.1136/bmjopen-2022-070882

	population	GBMSM		
Definitely would not	140 (4.6)	15 (6.1)	45 (5.4)	53 (5.1)
Probably would not	183 (6.0)	16 (6.5)	74 (8.9)	83 (8.0)
Not sure	352 (11.5)	25 (10.1)	74 (8.9)	116 (11.2)
Probably would	710 (23.3)	65 (26.3)	261 (31.4)	309 (29.8)
Definitely would	1621 (53.1)	122 (49.4)	373 (44.9)	472 (45.6)
Prefer not to say	44 (1.4)	4 (1.6)	4 (0.5)	3 (0.3)

Person you had shared bedding, towels, or clothes with in the last seven days

	General population	Savanta GBMSM	Grindr	Meta
Definitely would not	148 (4.9)	15 (6.1)	48 (5.8)	47 (4.5)
Probably would not	180 (5.9)	16 (6.5)	57 (6.9)	70 (6.8)
Not sure	352 (11.5)	26 (10.5)	69 (8.3)	81 (7.8)
Probably would	746 (24.5)	65 (26.3)	236 (28.4)	282 (27.2)
Definitely would	1574 (51.6)	122 (49.4)	415 (49.9)	551 (53.2)
Prefer not to say	50 (1.6)	3 (1.2)	6 (0.7)	5 (0.5)

Attitudes and beliefs

New screen

ASK ALL

Q17 How much do you agree or disagree with the following statements:

RANDOMISE statements

I wouldn’t want to know the results of a monkeypox test

	General population	Savanta GBMSM	Grindr	Meta
Strongly disagree	1057 (34.7)	110 (44.5)	498 (59.9)	690 (66.6)
Disagree	963 (31.6)	63 (25.5)	235 (28.3)	243 (23.5)
Neither agree nor disagree	365 (12.0)	23 (9.3)	29 (3.5)	27 (2.6)
Agree	353 (11.6)	23 (9.3)	27 (3.2)	22 (2.1)
Strongly agree	240 (7.9)	23 (9.3)	35 (4.2)	51 (4.9)
Don’t know	72 (2.4)	5 (2)	7 (0.8)	3 (0.3)

I would be worried what my friends or family would think about me if they thought I had monkeypox

	General population	Savanta GBMSM	Grindr	Meta
Strongly disagree	428 (14.0)	35 (14.2)	83 (10.0)	125 (12.1)
Disagree	624 (20.5)	43 (17.4)	114 (13.7)	147 (14.2)
Neither agree nor disagree	627 (20.6)	42 (17.0)	99 (11.9)	104 (10.0)

Agree	865 (28.4)	73 (29.6)	271 (32.6)	325 (31.4)
Strongly agree	402 (13.2)	52 (21.1)	263 (31.6)	333 (32.1)
Don't know	104 (3.4)	2 (0.8)	1 (0.1)	2 (0.2)

I would be worried about how colleagues / my employer would react if they thought I had monkeypox

	General population	Savanta GBMSM	Grindr	Meta
Strongly disagree	366 (12)	31 (12.6)	79 (9.5)	99 (9.6)
Disagree	508 (16.7)	40 (16.2)	95 (11.4)	112 (10.8)
Neither agree nor disagree	649 (21.3)	37 (15.0)	82 (9.9)	109 (10.5)
Agree	914 (30.0)	67 (27.1)	249 (30.0)	309 (29.8)
Strongly agree	462 (15.1)	64 (25.9)	316 (38.0)	391 (37.7)
Don't know	151 (5.0)	8 (3.2)	10 (1.2)	16 (1.5)

I don't want to have a monkeypox test result on my medical record

	General population	Savanta GBMSM	Grindr	Meta
Strongly disagree	541 (17.7)	50 (20.2)	161 (19.4)	229 (22.1)
Disagree	801 (26.3)	63 (25.5)	193 (23.2)	260 (25.1)
Neither agree nor disagree	837 (27.4)	58 (23.5)	183 (22.0)	205 (19.8)
Agree	494 (16.2)	42 (17.0)	142 (17.1)	151 (14.6)
Strongly agree	257 (8.4)	28 (11.3)	142 (17.1)	171 (16.5)
Don't know	120 (3.9)	6 (2.4)	10 (1.2)	20 (1.9)

An effective way to prevent the spread of monkeypox is for people who have symptoms to contact healthcare services

	General population	Savanta GBMSM	Grindr	Meta
Strongly disagree	67 (2.2)	7 (2.8)	16 (1.9)	19 (1.8)
Disagree	131 (4.3)	9 (3.6)	25 (3.0)	19 (1.8)
Neither agree nor disagree	459 (15.0)	24 (9.7)	58 (7.0)	64 (6.2)
Agree	1185 (38.9)	88 (35.6)	311 (37.4)	378 (36.5)
Strongly agree	1105 (36.2)	113 (45.7)	408 (49.1)	549 (53)
Don't know	103 (3.4)	6 (2.4)	13 (1.6)	7 (0.7)

I would be willing to contact a sexual health clinic if I thought I had monkeypox symptoms or had come into contact with someone who had monkeypox

	General population	Savanta GBMSM	Grindr	Meta
Strongly disagree	149 (4.9)	7 (2.8)	21 (2.5)	20 (1.9)
Disagree	291 (9.5)	20 (8.1)	24 (2.9)	17 (1.6)
Neither agree nor disagree	590 (19.3)	28 (11.3)	36 (4.3)	39 (3.8)
Agree	1047 (34.3)	73 (29.6)	260 (31.3)	247 (23.8)

Strongly agree	800 (26.2)	110 (44.5)	481 (57.9)	709 (68.4)
Don't know	173 (5.7)	9 (3.6)	9 (1.1)	4 (0.4)

New screen

ASK ALL

Q18 How much do you agree or disagree with the following statements:

RANDOMISE statements

If I had monkeypox symptoms, I wouldn't want to tell anyone as I don't want to self-isolate

	General population	Savanta GBMSM	Grindr	Meta
Strongly disagree	1055 (34.6)	79 (32.0)	291 (35.0)	374 (36.1)
Disagree	949 (31.1)	74 (30.0)	322 (38.7)	414 (40.0)
Neither agree nor disagree	431 (14.1)	33 (13.4)	98 (11.8)	119 (11.5)
Agree	365 (12.0)	32 (13.0)	72 (8.7)	78 (7.5)
Strongly agree	174 (5.7)	21 (8.5)	36 (4.3)	35 (3.4)
Don't know	76 (2.5)	8 (3.2)	12 (1.4)	16 (1.5)

If I had monkeypox symptoms, I wouldn't want to tell anyone so that others don't have to self-isolate

	General population	Savanta GBMSM	Grindr	Meta
Strongly disagree	909 (29.8)	71 (28.7)	260 (31.3)	305 (29.4)
Disagree	991 (32.5)	72 (29.1)	319 (38.4)	407 (39.3)
Neither agree nor disagree	468 (15.3)	37 (15.0)	122 (14.7)	143 (13.8)
Agree	396 (13)	35 (14.2)	86 (10.3)	114 (11.0)
Strongly agree	207 (6.8)	25 (10.1)	33 (4.0)	41 (4.0)
Don't know	79 (2.6)	7 (2.8)	11 (1.3)	26 (2.5)

Most people would self-isolate if they were told to

	General population	Savanta GBMSM	Grindr	Meta
Strongly disagree	132 (4.3)	14 (5.7)	84 (10.1)	90 (8.7)
Disagree	561 (18.4)	42 (17.0)	233 (28.0)	338 (32.6)
Neither agree nor disagree	634 (20.8)	51 (20.6)	180 (21.7)	222 (21.4)
Agree	1127 (37.0)	93 (37.7)	239 (28.8)	311 (30.0)
Strongly agree	461 (15.1)	32 (13.0)	51 (6.1)	36 (3.5)
Don't know	135 (4.4)	15 (6.1)	44 (5.3)	39 (3.8)

I have the support I need to self-isolate for 21 days

	General population	Savanta GBMSM	Grindr	Meta
Strongly disagree	173 (5.7)	26 (10.5)	142 (17.1)	132 (12.7)

Disagree	348 (11.4)	28 (11.3)	185 (22.3)	245 (23.6)
Neither agree nor disagree	458 (15.0)	41 (16.6)	129 (15.5)	127 (12.3)
Agree	1062 (34.8)	80 (32.4)	226 (27.2)	352 (34.0)
Strongly agree	942 (30.9)	66 (26.7)	133 (16.0)	168 (16.2)
Don't know	67 (2.2)	6 (2.4)	16 (1.9)	12 (1.2)

An effective way to prevent the spread of monkeypox is for people who have tested positive to self-isolate

	General population	Savanta GBMSM	Grindr	Meta
Strongly disagree	51 (1.7)	2 (0.8)	17 (2.0)	21 (2.0)
Disagree	107 (3.5)	13 (5.3)	16 (1.9)	34 (3.3)
Neither agree nor disagree	361 (11.8)	24 (9.7)	45 (5.4)	64 (6.2)
Agree	1018 (33.4)	68 (27.5)	319 (38.4)	362 (34.9)
Strongly agree	1424 (46.7)	132 (53.4)	416 (50.1)	543 (52.4)
Don't know	89 (2.9)	8 (3.2)	18 (2.2)	12 (1.2)

New screen

ASK ALL
Q19 How much do you agree or disagree with the following statements:

If I had to self-isolate because I had tested positive for monkeypox…

RANDOMISE statements

…I would lose touch with my friends and relatives

	General population	Savanta GBMSM	Grindr	Meta
Strongly disagree	567 (18.6)	44 (17.8)	151 (18.2)	180 (17.4)
Disagree	1039 (34.1)	78 (31.6)	320 (38.5)	408 (39.4)
Neither agree nor disagree	614 (20.1)	47 (19.0)	148 (17.8)	180 (17.4)
Agree	539 (17.7)	52 (21.1)	134 (16.1)	200 (19.3)
Strongly agree	225 (7.4)	19 (7.7)	61 (7.3)	65 (6.3)
Don't know	66 (2.2)	7 (2.8)	17 (2)	3 (0.3)

…it would have a severe impact on my family's wellbeing

	General population	Savanta GBMSM	Grindr	Meta
Strongly disagree	420 (13.8)	48 (19.4)	152 (18.3)	181 (17.5)
Disagree	740 (24.3)	58 (23.5)	279 (33.6)	350 (33.8)
Neither agree nor disagree	705 (23.1)	51 (20.6)	159 (19.1)	198 (19.1)
Agree	763 (25.0)	56 (22.7)	148 (17.8)	195 (18.8)

Strongly agree	338 (11.1)	21 (8.5)	81 (9.7)	99 (9.6)
Don't know	84 (2.8)	13 (5.3)	12 (1.4)	13 (1.3)

…it would have a negative impact on how much money I have

	General population	Savanta GBMSM	Grindr	Meta
Strongly disagree	498 (16.3)	52 (21.1)	132 (15.9)	184 (17.8)
Disagree	791 (25.9)	62 (25.1)	231 (27.8)	357 (34.5)
Neither agree nor disagree	566 (18.6)	27 (10.9)	99 (11.9)	153 (14.8)
Agree	694 (22.8)	56 (22.7)	162 (19.5)	169 (16.3)
Strongly agree	414 (13.6)	43 (17.4)	196 (23.6)	165 (15.9)
Don't know	87 (2.9)	7 (2.8)	11 (1.3)	8 (0.8)

…it would have a negative impact on my work

	General population	Savanta GBMSM	Grindr	Meta
Strongly disagree	545 (17.9)	52 (21.1)	76 (9.1)	98 (9.5)
Disagree	664 (21.8)	38 (15.4)	136 (16.4)	191 (18.4)
Neither agree nor disagree	622 (20.4)	49 (19.8)	100 (12)	136 (13.1)
Agree	697 (22.9)	55 (22.3)	260 (31.3)	325 (31.4)
Strongly agree	405 (13.3)	46 (18.6)	251 (30.2)	264 (25.5)
Don't know	117 (3.8)	7 (2.8)	8 (1.0)	22 (2.1)

…I would miss out on events and activities that I want to attend

	General population	Savanta GBMSM	Grindr	Meta
Strongly disagree	340 (11.1)	32 (13.0)	77 (9.3)	46 (4.4)
Disagree	471 (15.4)	33 (13.4)	106 (12.8)	107 (10.3)
Neither agree nor disagree	607 (19.9)	37 (15.0)	97 (11.7)	117 (11.3)
Agree	1097 (36.0)	84 (34.0)	354 (42.6)	457 (44.1)
Strongly agree	460 (15.1)	55 (22.3)	185 (22.3)	306 (29.5)
Don't know	75 (2.5)	6 (2.4)	12 (1.4)	3 (0.3)

Vaccination

New screen

People who have come into significant contact with someone who has tested positive for monkeypox are being asked to have a smallpox vaccine to reduce the risk of getting seriously ill. As monkeypox is caused by a virus similar to the one that causes smallpox, vaccines designed for smallpox are considered effective in preventing or reducing the severity of monkeypox.

Smith LE, *et al. BMJ Open* 2023; 13:e070882. doi: 10.1136/bmjopen-2022-070882

Like all medicines, this vaccine can cause side effects, but not everyone gets them. The most common side effects are pain and itching at the injection site. Most side effects are mild and clear fully without any treatment within 7 days.

New screen

For each question, please select the answer that reflects your opinion. Do not worry if you do not know what the best answer might be or if you are not at all familiar with monkeypox or vaccination. We only ask that you try to give your answer based on what you think you know or what you would honestly decide to do in the situations described. If you are really not sure, please answer "don't know".

New screen

ASK ALL
Q20 How much do you agree or disagree with the following statements:

RANDOMISE statements

In general, vaccination is a good thing

	General population	Savanta GBMSM	Grindr	Meta
Strongly disagree	65 (2.1)	9 (3.6)	15 (1.8)	6 (0.6)
Disagree	119 (3.9)	4 (1.6)	5 (0.6)	3 (0.3)
Neither agree nor disagree	361 (11.8)	21 (8.5)	33 (4.0)	15 (1.4)
Agree	1019 (33.4)	59 (23.9)	189 (22.7)	142 (13.7)
Strongly agree	1400 (45.9)	148 (59.9)	584 (70.3)	869 (83.9)
Don't know	86 (2.8)	6 (2.4)	5 (0.6)	1 (0.1)

Most people like me will get a smallpox vaccination if advised

	General population	Savanta GBMSM	Grindr	Meta
Strongly disagree	82 (2.7)	5 (2.0)	24 (2.9)	15 (1.4)
Disagree	248 (8.1)	14 (5.7)	41 (4.9)	43 (4.2)
Neither agree nor disagree	533 (17.5)	31 (12.6)	93 (11.2)	122 (11.8)
Agree	1270 (41.6)	111 (44.9)	404 (48.6)	470 (45.4)
Strongly agree	717 (23.5)	69 (27.9)	223 (26.8)	365 (35.2)
Don't know	200 (6.6)	17 (6.9)	46 (5.5)	21 (2.0)

If I get a smallpox vaccination, I will be protected against monkeypox

	General population	Savanta GBMSM	Grindr	Meta
Strongly disagree	100 (3.3)	9 (3.6)	27 (3.2)	6 (0.6)
Disagree	385 (12.6)	19 (7.7)	71 (8.5)	64 (6.2)
Neither agree nor disagree	927 (30.4)	52 (21.1)	158 (19.0)	139 (13.4)

Agree	953 (31.2)	102 (41.3)	391 (47.1)	599 (57.8)
Strongly agree	250 (8.2)	29 (11.7)	117 (14.1)	190 (18.3)
Don't know	435 (14.3)	36 (14.6)	67 (8.1)	38 (3.7)

A smallpox vaccination could give me smallpox

	General population	Savanta GBMSM	Grindr	Meta
Strongly disagree	614 (20.1)	70 (28.3)	400 (48.1)	668 (64.5)
Disagree	931 (30.5)	65 (26.3)	277 (33.3)	264 (25.5)
Neither agree nor disagree	662 (21.7)	38 (15.4)	69 (8.3)	51 (4.9)
Agree	392 (12.9)	32 (13.0)	27 (3.2)	17 (1.6)
Strongly agree	136 (4.5)	9 (3.6)	8 (1.0)	6 (0.6)
Don't know	315 (10.3)	33 (13.4)	50 (6.0)	30 (2.9)

I might regret getting the smallpox vaccination if I later experienced side effects from it

	General population	Savanta GBMSM	Grindr	Meta
Strongly disagree	420 (13.8)	48 (19.4)	259 (31.2)	410 (39.6)
Disagree	825 (27.0)	74 (30.0)	350 (42.1)	421 (40.6)
Neither agree nor disagree	710 (23.3)	43 (17.4)	88 (10.6)	121 (11.7)
Agree	655 (21.5)	50 (20.2)	89 (10.7)	61 (5.9)
Strongly agree	268 (8.8)	19 (7.7)	31 (3.7)	17 (1.6)
Don't know	172 (5.6)	13 (5.3)	14 (1.7)	6 (0.6)

I am already immune to monkeypox

	General population	Savanta GBMSM	Grindr	Meta
Strongly disagree	676 (22.2)	77 (31.2)	269 (32.4)	366 (35.3)
Disagree	800 (26.2)	51 (20.6)	213 (25.6)	251 (24.2)
Neither agree nor disagree	532 (17.4)	30 (12.1)	106 (12.8)	132 (12.7)
Agree	228 (7.5)	24 (9.7)	57 (6.9)	99 (9.6)
Strongly agree	138 (4.5)	13 (5.3)	26 (3.1)	44 (4.2)
Don't know	676 (22.2)	52 (21.1)	160 (19.3)	144 (13.9)

I would be worried about experiencing side effects from a smallpox vaccination

	General population	Savanta GBMSM	Grindr	Meta
Strongly disagree	392 (12.9)	44 (17.8)	246 (29.6)	364 (35.1)
Disagree	793 (26.0)	72 (29.1)	294 (35.4)	407 (39.3)
Neither agree nor disagree	646 (21.2)	41 (16.6)	105 (12.6)	136 (13.1)
Agree	819 (26.9)	54 (21.9)	146 (17.6)	109 (10.5)
Strongly agree	278 (9.1)	28 (11.3)	23 (2.8)	18 (1.7)
Don't know	122 (4.0)	8 (3.2)	17 (2.0)	2 (0.2)

Smith LE, *et al. BMJ Open* 2023; 13:e070882. doi: 10.1136/bmjopen-2022-070882

I would be worried that having a smallpox vaccine might make me infectious to others

	General population	Savanta GBMSM	Grindr	Meta
Strongly disagree	653 (21.4)	74 (30.0)	454 (54.6)	693 (66.9)
Disagree	1050 (34.4)	72 (29.1)	280 (33.7)	274 (26.4)
Neither agree nor disagree	605 (19.8)	33 (13.4)	46 (5.5)	32 (3.1)
Agree	378 (12.4)	31 (12.6)	18 (2.2)	15 (1.4)
Strongly agree	159 (5.2)	18 (7.3)	5 (0.6)	7 (0.7)
Don't know	205 (6.7)	19 (7.7)	28 (3.4)	15 (1.4)

People who are likely to come into high-risk contact with monkeypox should have a smallpox vaccine

	General population	Savanta GBMSM	Grindr	Meta
Strongly disagree	63 (2.1)	5 (2.0)	13 (1.6)	9 (0.9)
Disagree	158 (5.2)	15 (6.1)	8 (1.0)	5 (0.5)
Neither agree nor disagree	554 (18.2)	32 (13)	57 (6.9)	32 (3.1)
Agree	1134 (37.2)	89 (36)	256 (30.8)	234 (22.6)
Strongly agree	873 (28.6)	89 (36)	465 (56)	745 (71.9)
Don't know	268 (8.8)	17 (6.9)	32 (3.9)	11 (1.1)

New screen

ASK ALL

Q21 To the best of your knowledge, have you received a smallpox vaccine in 2022?

SINGLE CODE

Answer Options

	General population	Savanta GBMSM	Grindr	Meta
Yes	162 (5.3)	26 (10.5)	265 (31.9)	422 (40.7)
No. I was offered the vaccine, but didn't want to have it	116 (3.8)	9 (3.6)	11 (1.3)	16 (1.5)
No, I haven't been offered the vaccine	2609 (85.5)	202 (81.8)	536 (64.5)	587 (56.7)
Don't know / can't remember	163 (5.3)	10 (4.0)	19 (2.3)	11 (1.1)

New screen

ASK IF Q21=2,3,4

Q22 If you were advised by public health officials to have a smallpox vaccine **after coming into high-risk contact with someone who has monkeypox**, how likely would you be to have one?

SINGLE CODE

Answer Options

	General population, n=2888	Savanta GBMSM, n=221	Grindr, n=566	Meta, n=614
Definitely would not	115 (4.0)	6 (2.7)	16 (2.8)	10 (1.6)
Probably would not	139 (4.8)	15 (6.8)	13 (2.3)	4 (0.7)
Not sure	497 (17.2)	22 (10.0)	17 (3.0)	14 (2.3)
Probably would	814 (28.2)	48 (21.7)	95 (16.8)	51 (8.3)
Definitely would	1323 (45.8)	130 (58.8)	425 (75.1)	535 (87.1)

‡This question was not asked to people who had already had the smallpox vaccine in 2022, therefore total *n*s are different.

New screen

ASK IF Q21=2,3,4

Q23 Some people who have been in contact with someone who has monkeypox are being asked to self-isolate for 21 days. If having a smallpox vaccine means that you would have to self-isolate for less time, how likely would you be to get vaccinated?

SINGLE CODE

Answer Options

	General population, n=2888	Savanta GBMSM, n=221	Grindr, n=566	Meta, n=614
Definitely would not	114 (3.9)	5 (2.3)	17 (3.0)	9 (1.5)
Probably would not	128 (4.4)	9 (4.1)	17 (3.0)	8 (1.3)
Not sure	487 (16.9)	29 (13.1)	31 (5.5)	19 (3.1)
Probably would	822 (28.5)	45 (20.4)	92 (16.3)	55 (9.0)
Definitely would	1337 (46.3)	133 (60.2)	409 (72.3)	523 (85.2)

‡This question was not asked to people who had already had the smallpox vaccine in 2022, therefore total *n*s are different.

New screen

The smallpox vaccine is considered effective in preventing monkeypox or reducing the severity of monkeypox. Some people who are more likely to come into contact with monkeypox are being offered this vaccine.

ASK IF Q21=2,3,4

Q24 If you were offered a smallpox vaccine, how likely would you be to have one?

SINGLE CODE

Answer Options

	General population,	Savanta GBMSM,	Grindr, n=566	Meta, n=614

	n=2888	n=221		
Definitely would not	111 (3.8)	4 (1.8)	20 (3.5)	12 (2.0)
Probably would not	157 (5.4)	12 (5.4)	14 (2.5)	5 (0.8)
Not sure	518 (17.9)	21 (9.5)	20 (3.5)	21 (3.4)
Probably would	851 (29.5)	64 (29.0)	95 (16.8)	64 (10.4)
Definitely would	1251 (43.3)	120 (54.3)	417 (73.7)	512 (83.4)

‡This question was not asked to people who had already had the smallpox vaccine in 2022, therefore total *n*s are different.

New screen

ASK ALL
Q25. Do you have any further thoughts you would like to share with us about monkeypox, self-isolation, vaccines or anything else to do with monkeypox?

If you do not have anything to add, please put "None".

Type your answer below
OPEN END – require at least one alphabetic/numeric character

New screen

Sociodemographic variables

Now we are going to ask some questions about you. This survey is anonymous. This means that nobody can link you to your answers.

New screen

ASK ALL
D1A In the past month, **have you personally**:
Please select one option for each answer

Gone without enough food to eat

	General population	Savanta GBMSM	Grindr	Meta
Never	2006 (65.8)	171 (69.2)	670 (80.6)	938 (90.5)
Sometimes	663 (21.7)	48 (19.4)	121 (14.6)	85 (8.2)
Often	300 (9.8)	21 (8.5)	28 (3.4)	8 (0.8)
Don't know	63 (2.1)	7 (2.8)	7 (0.8)	3 (0.3)
Prefer not to say	18 (0.6)	0 (0.0)	5 (0.6)	2 (0.2)

Gone without an income

	General population	Savanta GBMSM	Grindr	Meta
Never	2014 (66)	172 (69.6)	653 (78.6)	890 (85.9)

Sometimes	600 (19.7)	43 (17.4)	105 (12.6)	106 (10.2)
Often	335 (11.0)	24 (9.7)	60 (7.2)	34 (3.3)
Don't know	69 (2.3)	6 (2.4)	2 (0.2)	2 (0.2)
Prefer not to say	32 (1.0)	2 (0.8)	11 (1.3)	4 (0.4)

Gone without fuel for heating or to cook food

	General population	Savanta GBMSM	Grindr	Meta
Never	2232 (73.2)	179 (72.5)	730 (87.8)	978 (94.4)
Sometimes	474 (15.5)	38 (15.4)	72 (8.7)	48 (4.6)
Often	255 (8.4)	25 (10.1)	22 (2.6)	6 (0.6)
Don't know	71 (2.3)	2 (0.8)	1 (0.1)	2 (0.2)
Prefer not to say	18 (0.6)	3 (1.2)	6 (0.7)	2 (0.2)

New screen

ASK ALL
D1B Last month, how difficult was it for you to cover your expenses and pay all your bills?
SINGLE CODE

	General population	Savanta GBMSM	Grindr	Meta
Not at all difficult	980 (32.1)	93 (37.7)	421 (50.7)	675 (65.2)
Somewhat difficult	1148 (37.6)	86 (34.8)	275 (33.1)	271 (26.2)
Very difficult	380 (12.5)	29 (11.7)	53 (6.4)	42 (4.1)
Extremely difficult	407 (13.3)	32 (13)	50 (6.0)	25 (2.4)
Don't know	55 (1.8)	1 (0.4)	2 (0.2)	2 (0.2)
Not applicable, I don't pay the bills in my household	53 (1.7)	5 (2.0)	22 (2.6)	10 (1.0)
Prefer not to say	27 (0.9)	1 (0.4)	8 (1)	11 (1.1)

New screen

ASK ALL
D2 How many people currently live in your household?

Pl*ease include yourself and all adults and children – including those not related to you*
SINGLE CODE

	General population	Savanta GBMSM	Grindr	Meta
I live alone	663 (21.7)	101 (40.9)	358 (43.1)	380 (36.7)
2	1104 (36.2)	90 (36.4)	313 (37.7)	531 (51.3)

Smith LE, *et al. BMJ Open* 2023; 13:e070882. doi: 10.1136/bmjopen-2022-070882

3 – 4	1055 (34.6)	42 (17.0)	131 (15.8)	103 (9.9)
5 – 6	204 (6.7)	13 (5.3)	25 (3.0)	15 (1.4)
7 +	24 (0.8)	1 (0.4)	4 (0.5)	7 (0.7)

New screen

ASK ALL
D3 Are you the parent / guardian of any dependent children?

Dependent children are those aged under 18 living in your household.
SINGLE CODE

	General population	Savanta GBMSM	Grindr	Meta
Yes, I have one or more dependent children	975 (32.0)	37 (15.0)	35 (4.2)	22 (2.1)
No, I don’t have any dependent children	2075 (68.0)	210 (85.0)	796 (95.8)	1014 (97.9)

New screen

ASK ALL
D4. Have you ever provided voluntary care for a family member or friend who needs support due to old age, physical illness, disability, mental health problems, or addiction?
Please select one option
SINGLE CODE

	General population	Savanta GBMSM	Grindr	Meta
Yes, I currently do this	623 (20.4)	45 (18.2)	158 (19.0)	155 (15.0)
I have previously provided care for a friend/family member, but no longer do	897 (29.4)	68 (27.5)	223 (26.8)	315 (30.4)
I have never provided care for a family member/friend in this way	1424 (46.7)	121 (49)	422 (50.8)	541 (52.2)
Prefer not to say	106 (3.5)	13 (5.3)	28 (3.4)	25 (2.4)

New screen

ASK ALL
D5 Do you currently have any pets that live in your home?
MULTI CODE

	General population	Savanta GBMSM	Grindr	Meta
Yes, I have a dog or cat	1534 (50.3)	117 (47.4)	286 (34.4)	336 (32.4)
Yes, I have a rabbit, ferret, or rodent (e.g. rat, mouse, chinchilla, guinea pig)	195 (6.4)	7 (2.8)	7 (0.8)	10 (1.0)
Yes, I have another type of animal (e.g. reptile, fish, bird)	232 (7.6)	17 (6.9)	43 (5.2)	51 (4.9)
No, I don't have a pet [EXCLUSIVE]	1311 (43.0)	118 (47.8)	516 (62.1)	667 (64.4)

†Participants could select more than one item for this question, therefore column totals add to more than 100%.

New screen

ASK ALL
D6 What is your employment status?
SINGLE CODE

	General population	Savanta GBMSM	Grindr	Meta
Full time paid job (31+ hours)	1163 (38.1)	120 (48.6)	529 (63.7)	626 (60.4)
Part time paid job (<31 hours)	471 (15.4)	21 (8.5)	58 (7.0)	90 (8.7)
Doing paid work on a self-employed basis or within your own business	102 (3.3)	9 (3.6)	82 (9.9)	115 (11.1)
Student / On a government training programme (Nation Traineeship/Modern Apprenticeship)	93 (3.0)	7 (2.8)	35 (4.2)	32 (3.1)
Out of work	120 (3.9)	7 (2.8)	31 (3.7)	19 (1.8)
Looking after home / Homemaker	184 (6.0)	4 (1.6)	3 (0.4)	5 (0.5)
Retired	707 (23.2)	53 (21.5)	57 (6.9)	106 (10.2)
Disabled OR Long-term sick	155 (5.1)	21 (8.5)	25 (3.0)	28 (2.7)
Unpaid work for a business, community or voluntary organisation	27 (0.9)	1 (0.4)	6 (0.7)	11 (1.1)
Prefer not to say	28 (0.9)	4 (1.6)	5 (0.6)	4 (0.4)

New screen

ASK IF D6= CODES 1,2, 3, 4, or 9

Smith LE, *et al. BMJ Open* 2023; 13:e070882. doi: 10.1136/bmjopen-2022-070882

D6A Are you a **frontline health or social care worker**? Please include any voluntary work

A frontline health or social care worker is someone who is directly involved in the care of patients or residents in long-stay care facilities (e.g. a care home), who has face-to-face contact with patients or clients, or who works in a laboratory, pathology or mortuary.

SINGLE CODE
FIX ORDER

	General population, n=1856	Savanta GBMSM, n=158	Grindr, n=710	Meta, n=874
Yes	355 (19.1)	23 (14.6)	94 (13.2)	125 (14.3)
No	1474 (79.4)	135 (85.4)	613 (86.3)	747 (85.5)
Prefer not to say	27 (1.5)	0 (0.0)	3 (0.4)	2 (0.2)

‡This question was only asked to those who reported that they were in full-time, part-time, self-employment, a student, or did unpaid or voluntary work, therefore total *n*s are different.

New screen

ASK IF D6= CODES 1, 2 OR 3
D6B If you needed to, are you able to work from home?
SINGLE CODE

	General population, n=1736	Savanta GBMSM, n=150	Grindr, n=669	Meta, n=831
Yes – full time	632 (36.4)	52 (34.7)	209 (31.2)	294 (35.4)
Yes – most of the time	260 (15.0)	26 (17.3)	136 (20.3)	196 (23.6)
Yes – but I have to go into my place of work once or twice a week	142 (8.2)	13 (8.7)	67 (10.0)	111 (13.4)
No – I cannot do my job from home	702 (40.4)	59 (39.3)	257 (38.4)	230 (27.7)

‡This question was only asked to those who reported that they were in full-time, part-time, or self-employment, therefore total *n*s are different.

New screen

ASK ALL
D7 What is the highest level of educational qualification you have received?
SINGLE CODE

	General population	Savanta GBMSM	Grindr	Meta
PhD/Doctorate	43 (1.4)	7 (2.8)	41 (4.9)	79 (7.6)

Smith LE, *et al. BMJ Open* 2023; 13:e070882. doi: 10.1136/bmjopen-2022-070882

Masters	213 (7.0)	22 (8.9)	204 (24.5)	325 (31.4)
Bachelor's Degree or equivalent (Such as a NVQ level 5)	729 (23.9)	59 (23.9)	291 (35.0)	379 (36.6)
Higher education (Such as a HND or a NVQ level 4)	348 (11.4)	29 (11.7)	106 (12.8)	89 (8.6)
A level or equivalent (Such as Scottish Highers or NVQ level 3)	666 (21.8)	58 (23.5)	110 (13.2)	88 (8.5)
GCSE and below (Such as O level or an RSA Diploma)	747 (24.5)	53 (21.5)	57 (6.9)	63 (6.1)
Other qualifications (Such as NVQ level 1)	119 (3.9)	12 (4.9)	11 (1.3)	11 (1.1)
No qualifications	185 (6.1)	7 (2.8)	11 (1.3)	2 (0.2)

New screen

ASK ALL
D8 Which of the following categories would best describe your ethnicity?
SINGLE CODE

	General population	Savanta GBMSM	Grindr	Meta
White				
British/English/Welsh/Scottish/Northern Irish	2649 (86.9)	214 (86.6)	629 (75.7)	799 (77.1)
Irish	38 (1.2)	5 (2.0)	26 (3.1)	31 (3.0)
Gypsy, Traveller or Irish Traveller	7 (0.2)	0 (0.0)	1 (0.1)	1 (0.1)
Any other White background	71 (2.3)	13 (5.3)	90 (10.8)	131 (12.6)
Mixed/ Multiple ethnic groups				
White and Black Caribbean	18 (0.6)	1 (0.4)	7 (0.8)	4 (0.4)
White and Black African	11 (0.4)	4 (1.6)	1 (0.1)	3 (0.3)
White and Asian	24 (0.8)	1 (0.4)	3 (0.4)	9 (0.9)
Any other Mixed/ Multiple ethnic background	16 (0.5)	1 (0.4)	12 (1.4)	17 (1.6)
Asian or Asian British				
Indian	41 (1.3)	4 (1.6)	20 (2.4)	1 (0.1)
Pakistani	50 (1.6)	0 (0.0)	2 (0.2)	0 (0.0)
Bangladeshi	12 (0.4)	0 (0.0)	1 (0.1)	1 (0.1)
Chinese	14 (0.5)	1 (0.4)	7 (0.8)	7 (0.7)
Any other Asian background	12 (0.4)	0 (0.0)	4 (0.5)	5 (0.5)
Black or Black British				

Smith LE, *et al. BMJ Open* 2023; 13:e070882. doi: 10.1136/bmjopen-2022-070882

African	26 (0.9)	1 (0.4)	5 (0.6)	2 (0.2)
Caribbean	32 (1.0)	0 (0.0)	8 (1.0)	4 (0.4)
Any other Black/ African/ Caribbean background	3 (0.1)	1 (0.4)	1 (0.1)	1 (0.1)
Other ethnic group				
Arab	7 (0.2)	0 (0.0)	3 (0.4)	1 (0.1)
Other	9 (0.3)	1 (0.4)	6 (0.7)	8 (0.8)
Don't know / Prefer not to say				
Don't know	2 (0.1)	0 (0.0)	0 (0.0)	1 (0.1)
Prefer not to say	8 (0.3)	0 (0.0)	5 (0.6)	10 (1.0)

New screen

ASK ALL

D9 Do you, or anyone else in your household have any long-standing illness, disability or infirmity?

Please select all that apply

MULTICODE

	General population	Savanta GBMSM	Grindr	Meta
Yes, I do	843 (27.6)	80 (32.4)	212 (25.5)	305 (29.4)
Yes, another household member does	391 (12.8)	21 (8.5)	88 (10.6)	115 (11.1)
No	1879 (61.6)	149 (60.3)	538 (64.7)	634 (61.2)
Prefer not to say	62 (2)	4 (1.6)	10 (1.2)	14 (1.4)

†Participants could select more than one item for this question, therefore column totals add to more than 100%.

New screen

ASK IF D9=1 (Yes, I do)

D9A Do you have any of the following health conditions?

Please select all that apply

MULTICODE

FIX ORDER

	General population, n=843	Savanta GBMSM, n=80	Grindr, n=212	Meta, n=305
Diabetes	184 (21.8)	21 (26.3)	38 (17.9)	49 (16.1)
Any type of cancer	57 (6.8)	4 (5.0)	10 (4.7)	14 (4.6)
Heart conditions	116 (13.8)	8 (10.0)	24 (11.3)	42 (13.8)
Respiratory conditions	191 (22.7)	15 (18.8)	27 (12.7)	59 (19.3)

Smith LE, *et al. BMJ Open* 2023; 13:e070882. doi: 10.1136/bmjopen-2022-070882

(e.g. chronic obstructive pulmonary disease [COPD], asthma)				
Mental health	317 (37.6)	34 (42.5)	84 (39.6)	102 (33.4)
A skin condition (e.g. atopic dermatitis / eczema)	114 (13.5)	9 (11.3)	25 (11.8)	46 (15.1)
A condition that makes you much more likely to get infections (e.g. SCID, homozygous sickle cell)	20 (2.4)	0 (0.0)	7 (3.3)	7 (2.3)
A weakened immune system	110 (13)	8 (10.0)	24 (11.3)	38 (12.5)
Are taking medicine that weakens your immune system (e.g. steroid tablets, chemotherapy, or antiretroviral medications)	62 (7.4)	4 (5.0)	15 (7.1)	24 (7.9)
HIV/AIDS	6 (0.7)	12 (15.0)	52 (24.5)	117 (38.4)
Other, please specify	183 (21.7)	11 (13.8)	48 (22.6)	60 (19.7)
Prefer not to say	46 (5.5)	5 (6.3)	11 (5.2)	8 (2.6)

†Participants could select more than one item for this question, therefore column totals add to more than 100%.

‡This question was only asked to those who reported that had a chronic illness, therefore total *n*s are different.

New screen

ASK ALL

D9B Are you pregnant?

SINGLE CODE

	General population	Savanta GBMSM	Grindr	Meta
Yes	38 (1.2)	1 (0.4)	0 (0.0)	0 (0.0)
No	3001 (98.4)	245 (99.2)	830 (99.9)	1035 (99.9)
Prefer not to say	11 (0.4)	1 (0.4)	1 (0.1)	1 (0.1)

New screen

ASK ALL

D9C Have you ever taken PrEP (pre-exposure prophylaxis) for HIV?

SINGLE CODE

	General population	Savanta GBMSM	Grindr	Meta
Yes	42 (1.4)	28 (11.3)	402 (48.4)	487 (47.0)
No	2993 (98.1)	216 (87.4)	427 (51.4)	547 (52.8)
Prefer not to say	15 (0.5)	3 (1.2)	2 (0.2)	2 (0.2)

New screen

ASK ALL

D10 To the best of your knowledge, have you received a vaccine for…

Hepatitis A

	General population	Savanta GBMSM	Grindr	Meta
Yes	531 (17.4)	87 (35.2)	549 (66.1)	773 (74.6)
No. I was offered the vaccine, but didn't want to have it	128 (4.2)	12 (4.9)	7 (0.8)	4 (0.4)
No, I haven't been offered the vaccine	1579 (51.8)	94 (38.1)	129 (15.5)	125 (12.1)
Don't know / can't remember	812 (26.6)	54 (21.9)	146 (17.6)	134 (12.9)

Smallpox (before 2022)

	General population	Savanta GBMSM	Grindr	Meta
Yes	512 (16.8)	51 (20.6)	144 (17.3)	218 (21)
No. I was offered the vaccine, but didn't want to have it	117 (3.8)	12 (4.9)	5 (0.6)	7 (0.7)
No, I haven't been offered the vaccine	1666 (54.6)	133 (53.8)	477 (57.4)	595 (57.4)
Don't know / can't remember	755 (24.8)	51 (20.6)	205 (24.7)	216 (20.8)

Covid-19 (two doses or more)

	General population	Savanta GBMSM	Grindr	Meta
Yes	2557 (83.8)	218 (88.3)	796 (95.8)	1023 (98.7)
No. I was offered the vaccine, but didn't want to have it	242 (7.9)	16 (6.5)	27 (3.2)	11 (1.1)
No, I haven't been offered the vaccine	189 (6.2)	9 (3.6)	5 (0.6)	0 (0.0)
Don't know / can't remember	62 (2.0)	4 (1.6)	3 (0.4)	2 (0.2)

New screen

Smith LE, *et al. BMJ Open* 2023; 13:e070882. doi: 10.1136/bmjopen-2022-070882

ASK ALL
D11 What is your marital status?
SINGLE CODE

	General population	Savanta GBMSM	Grindr	Meta
Single, never married	741 (24.3)	123 (49.8)	470 (56.6)	402 (38.8)
Married / civil partnership	1382 (45.3)	62 (25.1)	149 (17.9)	290 (28)
Separated	69 (2.3)	3 (1.2)	29 (3.5)	21 (2.0)
Divorced	319 (10.5)	14 (5.7)	57 (6.9)	39 (3.8)
Widowed	136 (4.5)	1 (0.4)	5 (0.6)	24 (2.3)
Partnered / in a relationship	393 (12.9)	43 (17.4)	111 (13.4)	251 (24.2)
Prefer not to say	10 (0.3)	1 (0.4)	10 (1.2)	9 (0.9)

New screen

The following questions are sensitive. You don't need to answer if you don't want to. If you don't want to answer, please select "prefer not to say".

New screen

ASK ALL
D12A How many **male** sexual partners have you had…?

By "sexual partner" we mean any form of genital contact, including touching, kissing, and intercourse.

Please give an approximate number if you are unsure. If you have not had any male sexual partners in this time, please put 0.
Type your answer below

		General population	Savanta GBMSM	Grindr	Meta
In the last 3 weeks (21 days)	OPEN TEXT ENTRY – number [cap at 100]	N=2418, M=0.6, SD=3.6, range 0 to 100.	N=210, M=1.1, SD=2.0, range 0 to 14.	N=787, M=2.9, SD=5.2, range 0 to 75.	N=982, M=2.5, SD=4.7, range 0 to 100.
In the last 3 months (90 days)	OPEN TEXT ENTRY – number	N=2418, M=0.8, SD=8.9, range 0 to 400.	N=210, M=2.8, SD=7.0, range 0 to 50.	N=787, M=9.1, SD=23.8, range 0 to 400.	N=982, M=8.9, SD=26.2, range 0 to 400.

	[cap at 400]				
Prefer not to say		632 (20.7)	37 (15.0)	44 (5.3)	54 (5.2)

New screen

ASK ALL

D12B How many **female** sexual partners have you had…?

By "sexual partner" we mean any form of genital contact, including touching, kissing, and intercourse.

Please give an approximate number if you are unsure. If you have not had any female sexual partners in this time, please put 0.

Type your answer below

		General population	Savanta GBMSM	Grindr	Meta
In the last 3 weeks (21 days)	OPEN TEXT ENTRY – number [cap at 100]	N=2442, M=0.4, SD=3.1, range 0 to 100.	N=207, M=0.2, SD=0.8, range 0 to 100.	N=792, M=0.1, SD=0.6, range 0 to 10.	N=992, M=0.0, SD=0.4, range 0 to 9.
In the last 3 months (90 days)	OPEN TEXT ENTRY – number [cap at 400]	N=2442, M=0.8, SD=9.2, range 0 to 400.	N=207, M=2.2, SD=27.8, range 0 to 400.	N=792, M=0.2, SD=1.6, range 0 to 30.	N=992, M=0.1, SD=1.0, range 0 to 28.
Prefer not to say		608 (19.9)	40 (16.2)	39 (4.7)	44 (4.2)

New screen

Thanks for completing the survey. If you would like any more information on monkeypox, please see https://www.nhs.uk/conditions/monkeypox/.

Please press the Finish button to complete the survey.
Many thanks for completing this survey, your views are extremely important to us.

If you have any further comments or feedback, please use the box below.
