# Supplementary material for: Did mpox knowledge, attitudes and beliefs affect intended behaviour in the general population and men who are gay, bisexual and who have sex with men? An online cross-sectional survey in the UK
Source: BMJ Open. 2023 Oct 12;13(10):e070882. doi: 10.1136/bmjopen-2022-070882 (PMC10583036; doi:10.1136/bmjopen-2022-070882)
Supplement: Supplementary data [file bmjopen-2022-070882supp002.pdf]

Supplementary materials 2. Data preparation

Some participants (3.8%, n=196) did not provide their postcode and were therefore missing data about region and index of multiple deprivation. While individual items about financial hardship had small amounts of missing data (2.0% to 3.8%, n=105 to 197), as items were summed to give a scale, missingness on the resulting scale would have been higher (7.9%, n=410). There were no apparent patterns to the missing data. We used multiple imputation to impute missing values for region, index of multiple deprivation and financial hardship items. Socio-demographic variables (age, occupation of highest earner, number of people in household, having a dependent child, employment status, education, ethnicity, marital status, and region, index of multiple deprivation and financial hardship where available) were used to predict missing values using a Markov Chain Monte Carlo (MCMC) method using linear regressions with ten iterations, to give five imputations.

We computed a single variable to indicate perceived susceptibility and severity from three items. For these items, "don't know" was coded as the mid-point on the scale. We then multiplied responses together (range 1 to 125) and took the cubic root to give a scale from 1 (lowest perceived susceptibility and severity) to 5 (highest perceived susceptibility and severity). We also asked participants how much they agreed that they were already immune to mpox. This item was recoded into a binary item to show perceived immunity to mpox (strongly agree and agree, vs neither agree nor disagree, disagree, strongly disagree, and don't know).

To create a single variable for perceived knowledge, we used three items (Cronbach's α=0.74). Responses for these items were given on a five-point scale from "strongly disagree" to "strongly agree" We recoded each item as a binary item (strongly agree and agree [1], vs neither agree nor disagree, disagree, strongly disagree, and don't know [0]), then summed responses to give a scale from 0 (lowest perceived knowledge) to 3 (highest perceived knowledge).

For knowledge about mpox symptoms, for each symptom selected, we coded participants as being correct (1, symptom listed on NHS mpox website) or incorrect (0, symptom not listed on NHS mpox website). Scores were then summed to give a scale from 0 (no selected symptoms were mpox symptoms) to 4 (all selected symptoms were mpox symptoms); responses of "don't know" were coded as 0. A single variable denoting understanding of transmission was computed. It is unknown whether mpox can be caught from pet animals

Smith LE, *et al. BMJ Open* 2023; 13:e070882. doi: 10.1136/bmjopen-2022-070882

(but pet animals can catch mpox from humans (1)), therefore we did not include this item in analyses. We coded answers as correct (1) or incorrect (0; answers of "don't know" coded as incorrect), and summed items to give a score from 0 (lowest knowledge about transmission) to 6 (highest knowledge about transmission).

To reduce the number of variables included in regression models, we used dimension reduction techniques. We conducted principal components analysis, using direct oblimin rotation as items may have been correlated, on items potentially associated with self-isolation (ten items), help seeking (six items), and vaccination (eight items) separately. The number of factors was determined using a scree plot. The item with the highest loading on to a component was then included in regression analyses. Results of the principal components analyses are reported in the supplementary materials 3.

References

1. Seang S, Burrel S, Todesco E, Leducq V, Monsel G, Le Pluart D, et al. Evidence of human-to-dog transmission of monkeypox virus. The Lancet. 2022;400(10353):658-9.

Smith LE, *et al. BMJ Open* 2023; 13:e070882. doi: 10.1136/bmjopen-2022-070882
