# Supplementary material for: Did mpox knowledge, attitudes and beliefs affect intended behaviour in the general population and men who are gay, bisexual and who have sex with men? An online cross-sectional survey in the UK
Source: BMJ Open. 2023 Oct 12;13(10):e070882. doi: 10.1136/bmjopen-2022-070882 (PMC10583036; doi:10.1136/bmjopen-2022-070882)
Supplement: Supplementary data [file bmjopen-2022-070882supp003.pdf]

Supplementary materials 3. Results of principal components analyses.

Self-isolation

A scree plot suggested three components underlying factors potentially associated with self-isolation. Table 1 shows loadings of items onto each component.

Table 1. Loadings of items measuring factors potentially associated with self-isolation onto components identified (only loadings over ± .4 are shown).

Item	Component 1 loading	Component 2 loading	Component 3 loading
If I had mpox symptoms, I wouldn't want to tell anyone as I don't want to self-isolate*		-.864	
If I had mpox symptoms, I wouldn't want to tell anyone so that others don't have to self-isolate		-.853	
Most people would self-isolate if they were told to*			.810
I have the support I need to self-isolate for 21 days			.729
An effective way to prevent the spread of mpox is for people who have tested positive to self-isolate		.544	.426
If I had to self-isolate because I had tested positive for mpox...I would lose touch with my friends and relatives	.627		
If I had to self-isolate because I had tested positive for mpox...it would have a severe impact on my family's wellbeing	.684		
If I had to self-isolate because I had tested positive for mpox...it would have a negative impact on how much money I have	.754		
If I had to self-isolate because I had tested positive for mpox...it would have a negative impact on my work*	.789		
If I had to self-isolate because I had tested positive for mpox...I would miss out on events and activities that I want to attend	.646		

Rotation method: oblimin with Kaiser normalization. Rotation converged in 10 iterations.
*Included in regression analyses

Help seeking

A scree plot suggested two components underlying factors potentially associated with seeking help immediately. Table 2 shows loadings of items onto each component.

Table 2. Loadings of items measuring factors potentially associated with self-isolation onto components identified (only loadings over ± .4 are shown).

Item	Component 1 loading	Component 2 loading
I wouldn't want to know the results of a mpox test		-.526
I would be worried what my friends or family would think about me if they thought I had mpox *	.867	
I would be worried about how colleagues / my employer would react if they thought I had mpox	.845	
I don't want to have a mpox test result on my medical record	.727	
An effective way to prevent the spread of mpox is for people who have symptoms to contact healthcare services		.772
I would be willing to contact a sexual health clinic if I thought I had mpox symptoms or had come into contact with someone who had mpox *		.784

Rotation method: oblimin with Kaiser normalization. Rotation converged in 2 iterations.
*Included in regression analyses

Smith LE, *et al. BMJ Open* 2023; 13:e070882. doi: 10.1136/bmjopen-2022-070882

Vaccination

A scree plot suggested two components underlying factors potentially associated with vaccination. Table 3 shows loadings of items onto each component.

Table 3. Loadings of items measuring factors potentially associated with vaccination onto components identified (only loadings over ± .4 are shown).

Item	Component 1 loading	Component 2 loading
In general, vaccination is a good thing		.627
Most people like me will get a smallpox vaccination if advised		.737
If I get a smallpox vaccination, I will be protected against mpox *		.746
A smallpox vaccination could give me smallpox	.801	
I might regret getting the smallpox vaccination if I later experienced side effects from it	.789	
I would be worried about experiencing side effects from a smallpox vaccination	.793	
I would be worried that having a smallpox vaccine might make me infectious to others*	.843	
People who are likely to come into high-risk contact with mpox should have a smallpox vaccine		.744

Rotation method: oblimin with Kaiser normalization. Rotation converged in 4 iterations.
*Included in regression analyses

Smith LE, *et al. BMJ Open* 2023; 13:e070882. doi: 10.1136/bmjopen-2022-070882
