# Supplementary material for: Did mpox knowledge, attitudes and beliefs affect intended behaviour in the general population and men who are gay, bisexual and who have sex with men? An online cross-sectional survey in the UK
Source: BMJ Open. 2023 Oct 12;13(10):e070882. doi: 10.1136/bmjopen-2022-070882 (PMC10583036; doi:10.1136/bmjopen-2022-070882)
Supplement: Supplementary data [file bmjopen-2022-070882supp004.pdf]

Supplementary materials 4. Influence of motivational messages on behavioural outcomes.

Table 1. Influence of motivational messaging on behavioural outcomes, in the general population sample.

			General population, n (%)				
			Risk + perceived necessity/efficacy, n=762	Risk + perceived benefits, n=768	Risk + perceived costs, n=762	Control, n=758	p-value
**Self-isolation for 21 days**	Sum of intention if case and high-risk contact	2 (lowest intention) to 10 (highest intention)	M=8.0, SD=2.2	M=7.9, SD=2.2	M=7.8, SD=2.2	M=8.1, SD=2.1	0.07
**Help seeking**	Any action that involves contacting a healthcare professional (by phone or in person)	Would not seek help or would seek help, but would "wait and see" for a day or two to see if symptoms resolved	362 (47.5)	413 (53.8)	382 (50.1)	391 (51.6)	0.10
		Would seek help immediately	400 (52.5)	355 (46.2)	380 (49.9)	367 (48.4)	
**Contact behaviour**	Have sexual contact with others†	Less than, same as, or more than normal	125 (20.5)	146 (22.6)	148 (23.8)	148 (24.2)	0.41
		Completely stop	485 (79.5)	501 (77.4)	473 (76.2)	464 (75.8)	
**Contact sharing**	Sexual contacts	1 (definitely would not) to 5 (definitely would)	N=748, M=4.2, SD=1.2	N=756, M=4.2, SD=1.2	N=746, M=4.2, SD=1.1	N=747, M=4.1, SD=1.1	0.98
**Vaccination intention**	Smallpox vaccine "if advised"	1 (definitely would not) to 5 (definitely would)	N=723, M=4.0, SD=1.1	N=726, M=4.0, SD=1.1	N=721, M=4.0, SD=1.1	N=718, M=4.0, SD=1.1	0.81

†Answers of "don't know", "prefer not to say" and "not applicable, I wouldn't do this anyway" were coded as missing, therefore total *n* was substantially lower (n=2490).

Table 2. Influence of motivational messaging on behavioural outcomes, by GBMSM sample.

			**Savanta GBMSM, n (%)**			**Grindr, n (%)**			**Meta, n (%)**		
			Motivational message, n=115	Control, n=132	p-value	Motivational message, n=404	Control, n=427	p-value	Motivational message, n=529	Control, n=507	p-value
**Self-isolation for 21 days**	2 (lowest intention) to 10 (highest intention)		M=7.7, SD=2.4	M=8.0, SD=2.1	0.22	M=7.2, SD=2.4	M=7.3, SD=2.5	0.74	M=7.1, SD=2.3	M=7.5, SD=2.2	0.005
**Help seeking**	Any action that involves contacting a healthcare professional (by phone or in person)	Would not seek help or would seek help, but would "wait and see" for a day or two to see if symptoms resolved	59 (51.3)	66 (50.0)	0.84	189 (46.8)	199 (46.6)	0.96	187 (35.3)	204 (40.2)	0.10
		Would seek help immediately	56 (48.7)	66 (50.0)		215 (53.2)	228 (53.4)		342 (64.7)	303 (59.8)	
**Contact behaviour**	Have sexual contact with others†	Less than, same as, or more than normal	26 (26.5)	19 (16.1)	0.06	28 (7.3)	41 (10.1)	0.17	38 (7.5)	31 (6.4)	0.50
		Completely stop	72 (73.5)	99 (83.9)		355 (92.7)	366 (89.9)		469 (92.5)	453 (93.6)	
**Contact sharing**	Sexual contacts	1 (definitely would not) to 5 (definitely would)	N=113, M=4.0, SD=1.2	N=130, M=4.1, SD=1.2	0.54	N=402, M=4.1, SD=1.2	N=421, M=4.1, SD=1.2	0.72	N=526, M=4.1, SD=1.2	N=505, M=4.2, SD=1.1	0.04
**Vaccination intention**	Smallpox vaccine "if advised"‡	1 (definitely would not) to 5 (definitely would)	N=103, M=4.3, SD=1.0	N=118, M=4.2, SD=1.0	0.36	N=282, M=4.6, SD=0.9	N=284, M=4.5, SD=1.0	0.18	N=304, M=4.7, SD=0.9	N=310, M=4.8, SD=0.6	0.03

†Answers of "don't know", "prefer not to say" and "not applicable, I wouldn't do this anyway" were coded as missing, therefore total *n*s were substantially lower (Savanta GBMSM, n=216; Grindr, n=790; Meta, n=991).

‡Not asked to people who reported that they had had the smallpox vaccine in 2022, therefore total *n*s were substantially lower (Savanta GBMSM, n=221; Grindr, n=566; Meta, n=614).

Smith LE, *et al. BMJ Open* 2023; 13:e070882. doi: 10.1136/bmjopen-2022-070882
