# Supplementary material for: Did mpox knowledge, attitudes and beliefs affect intended behaviour in the general population and men who are gay, bisexual and who have sex with men? An online cross-sectional survey in the UK
Source: BMJ Open. 2023 Oct 12;13(10):e070882. doi: 10.1136/bmjopen-2022-070882 (PMC10583036; doi:10.1136/bmjopen-2022-070882)
Supplement: Supplementary data [file bmjopen-2022-070882supp005.pdf]

Supplementary materials 5. Full results of regressions with intention to seek help immediately.

Table 1. Associations between intending to seek help immediately and socio-demographic characteristics and motivational message, by sample. Variables were entered into the logistic regression model in blocks (block 1: socio-demographic variables and motivational message, block 2: psychological factors, block 3: help-seeking specific factors). Results for block 3, using pooled estimates are reported.

Participant characteristics	Level	General population		Grindr	
		aOR (95% CI)	*p*-value	aOR (95% CI)	*p*-value
Gender	Male (including trans man)	Ref	-	-	-
	Female (including trans woman)	1.10 (0.93 to 1.30)	0.25	-	-
Sexual orientation	Straight or heterosexual	Ref	-	-	-
	Gay, lesbian, bisexual, or queer	1.15 (0.84 to 1.58)	0.39	-	-
Age	Range 18 to 98 years	1.012 (1.006 to 1.019)	<0.001*	1.02 (1.01 to 1.04)	0.006
	Quadratic term, $(\text{age} – \text{mean})^2$	1.0002 (0.9999 to 1.0005)	0.24	1.0001 (0.9992 to 1.0011)	0.80
Region	Midlands (East and West)	Ref	-	Ref	-
	South (East, West, East of England)	1.10 (0.87 to 1.40)	0.40	1.01 (0.50 to 2.04)	0.97
	North (East, West, Yorkshire and the Humber)	0.90 (0.71 to 1.15)	0.41	1.53 (0.75 to 3.13)	0.24
	London	0.90 (0.65 to 1.25)	0.54	1.15 (0.59 to 2.26)	0.67
	Devolved nations (Scotland, Wales, and Northern Ireland)	0.83 (0.62 to 1.13)	0.24	1.04 (0.47 to 2.28)	0.93
Dependent child in household	No	Ref	-	Ref	-
	Yes	0.96 (0.78 to 1.17)	0.68	1.58 (0.71 to 3.51)	0.26
Employment status	Not working	Ref	-	Ref	-
	Working	1.12 (0.87 to 1.43)	0.39	0.98 (0.57 to 1.68)	0.94
Frontline health or social care worker	No	Ref	-	Ref	-
	Yes	1.05 (0.81 to 1.36)	0.73	1.91 (1.15 to 3.18)	0.01
Need to leave home for work	Do not need to leave home for work	Ref	-	Ref	-
	Need to leave home for work	1.05 (0.84 to 1.31)	0.65	0.94 (0.64 to 1.37)	0.75
Education	GCSE/vocational/A-level/No formal qualifications	Ref	-	Ref	-
	Degree or higher (Bachelors, Masters, PhD)	0.87 (0.72 to 1.04)	0.12	0.94 (0.66 to 1.34)	0.74
Ethnicity	White British	Ref	-	Ref	-
	White other	1.26 (0.82 to 1.92)	0.29	1.03 (0.65 to 1.65)	0.89
	Black, Asian, other minoritized ethnicity	0.90 (0.66 to 1.22)	0.48	0.95 (0.55 to 1.63)	0.85
Marital status	Not partnered	Ref	-	Ref	-
	Partnered	1.09 (0.87 to 1.36)	0.44	0.81 (0.53 to 1.22)	0.31
Live alone	Live with someone else	Ref	-	Ref	-
	Live alone	1.10 (0.85 to 1.41)	0.48	0.79 (0.55 to 1.15)	0.23
Own chronic illness	None	Ref	-	Ref	-
	Present	1.11 (0.92 to 1.34)	0.28	1.28 (0.88 to 1.88)	0.20
Ever taken PrEP for HIV	No	-	-	Ref	-
	Yes	-	-	0.89 (0.62 to 1.28)	0.54
Vaccinated for smallpox in 2022	Not vaccinated	-	-	Ref	-
	Vaccinated	-	-	0.85 (0.57 to 1.27)	0.43
Index of multiple	Deciles (1st = most deprived, 10th	1.00 (0.97 to 1.03)	0.93	1.05 (0.98 to 1.13)	0.16

deprivation	= least deprived)				
Socio-economic grade	ABC1	Ref	-	Ref	-
	C2DE	0.94 (0.79 to 1.13)	0.53	0.66 (0.41 to 1.07)	0.09
Financial hardship	4 (lowest hardship) to 13 (most hardship)	0.96 (0.92 to 1.00)	0.07	0.99 (0.90 to 1.09)	0.87
Total number of sexual partners (male and female) in last three weeks	0	Ref	-	-	-
	1	1.09 (0.88 to 1.34)	0.45	-	-
	2 to 4	1.09 (0.73 to 1.62)	0.68	-	-
	5 or more	0.78 (0.38 to 1.64)	0.52	-	-
	Prefer not to say	1.09 (0.86 to 1.37)	0.49	-	-
Number of male sexual partners in last three weeks	0	-	-	Ref	-
	1	-	-	1.01 (0.63 to 1.62)	0.95
	2 to 4	-	-	1.01 (0.66 to 1.56)	0.95
	5 to 9	-	-	0.84 (0.47 to 1.49)	0.55
	10 or more	-	-	1.06 (0.51 to 2.17)	0.88
	Prefer not to say	-	-	0.93 (0.43 to 2.03)	0.86
Motivational message	Perceived risk of illness and necessity and efficacy of the response	1.16 (0.93 to 1.44)	0.19	-	-
	Perceived risk of illness and benefits of the response	0.96 (0.77 to 1.20)	0.71	-	-
	Perceived risk of illness and low perceived costs of response	1.05 (0.84 to 1.31)	0.68	-	-
	Control	Ref	-	-	-
Motivational message	All motivational components	-	-	0.86 (0.63 to 1.17)	0.34
	Control	-	-	Ref	-

*$p \leq 0.001$

Table 2. Associations between intending to seek help immediately and psychological and contextual factors, by sample. Variables were entered into the logistic regression model in blocks (block 1: socio-demographic variables and motivational message, block 2: psychological factors, block 3: help-seeking specific factors). Results for block 3, using pooled estimates are reported.

Factor	Level	General population aOR (95% CI)	*p*-value	Grindr aOR (95% CI)	*p*-value
Amount heard about mpox	I have not seen or heard anything (1) to I have seen or heard a lot (3)	1.05 (0.87 to 1.25)	0.63	0.96 (0.67 to 1.37)	0.83
Worry about mpox	Not at all worried (1) to extremely worried (4)	1.05 (0.90 to 1.23)	0.52	1.17 (0.84 to 1.63)	0.36
Perceived risk of mpox to oneself	No risk at all (1) to very high risk (5)	0.85 (0.74 to 0.98)	0.02	0.90 (0.72 to 1.14)	0.38
Perceived risk of mpox to people in UK	No risk at all (1) to very high risk (5)	1.04 (0.90 to 1.19)	0.62	1.37 (1.07 to 1.76)	0.01
Perceived susceptibility and severity	Lowest (1) to highest (5)	1.07 (0.94 to 1.23)	0.32	1.03 (0.77 to 1.36)	0.86
I am already immune to mpox	Strongly disagree, disagree, neither agree nor disagree, don't know	Ref	-	Ref	-
	Strongly agree and agree	0.95 (0.72 to 1.26)	0.73	0.69 (0.40 to 1.19)	0.18
People who catch mpox usually make a full recovery, even if they do not receive any treatment	Strongly disagree (1) to strongly agree (5)	0.87 (0.78 to 0.96)	0.006	0.84 (0.69 to 1.03)	0.09
My personal behaviour has an impact on how mpox spreads	Strongly disagree (1) to strongly agree (5)	1.05 (0.98 to 1.13)	0.19	1.07 (0.92 to 1.25)	0.37
My life has been negatively affected by changes made in response to the mpox outbreak	Strongly disagree (1) to strongly agree (5)	1.00 (0.91 to 1.10)	0.99	1.00 (0.85 to 1.17)	0.99
The risks of mpox are being exaggerated	Strongly disagree (1) to strongly agree (5)	0.83 (0.76 to 0.91)	<0.001*	0.94 (0.77 to 1.13)	0.48
Mpox is only a risk to men who are gay, bisexual or have sex with men	Strongly disagree (1) to strongly agree (5)	0.95 (0.88 to 1.03)	0.22	0.85 (0.72 to 1.01)	0.06
Perceived knowledge	Lowest (0) to highest (3)	0.97 (0.89 to 1.06)	0.54	0.99 (0.80 to 1.23)	0.93
Knowledge of mpox symptoms	Identified no symptoms (0) to identified four symptoms (4)	1.03 (0.96 to 1.09)	0.44	1.13 (0.98 to 1.30)	0.09
Knowledge of mpox transmission	Lowest (0) to highest (6)	0.95 (0.90 to 1.01)	0.12	1.03 (0.89 to 1.20)	0.69
I would be worried what my friends or family would think about me if they thought I had mpox	Strongly disagree (1) to strongly agree (5)	0.94 (0.88 to 1.00)	0.06	0.85 (0.75 to 0.97)	0.01
I would be willing to contact a sexual health clinic if I thought I had mpox symptoms or had come into contact with someone who had mpox	Strongly disagree (1) to strongly agree (5)	1.25 (1.16 to 1.34)	<.001*	1.57 (1.29 to 1.91)	<.001*

*$p \leq 0.001$
