# Supplementary material for: Did mpox knowledge, attitudes and beliefs affect intended behaviour in the general population and men who are gay, bisexual and who have sex with men? An online cross-sectional survey in the UK
Source: BMJ Open. 2023 Oct 12;13(10):e070882. doi: 10.1136/bmjopen-2022-070882 (PMC10583036; doi:10.1136/bmjopen-2022-070882)
Supplement: Supplementary data [file bmjopen-2022-070882supp006.pdf]

## Supplementary materials 6. Full results of regressions with smallpox vaccination.

Table 1. Associations between vaccine uptake (general population sample: actual and intended, GBMSM: actual, actual and intended) and socio-demographic characteristics and motivational message, by sample. Variables were entered into the logistic regression model in blocks (block 1: socio-demographic variables and motivational message, block 2: psychological factors, block 3: vaccination specific factors). Results for block 3, using pooled estimates are reported.

| Participant characteristics | Level | General population | | Grindr | | | |
|---|---|---|---|---|---|---|---|
| | | Vaccinated in 2022 or would be vaccinated if advised | | Vaccinated in 2022 | | Vaccinated in 2022 or would be vaccinated if advised | |
| | | aOR (95% CI) | *p*-value | aOR (95% CI) | *p*-value | aOR (95% CI) | *p*-value |
| Gender | Male (including trans man) | Ref | - | - | - | - | - |
| | Female (including trans woman) | 0.93 (0.75 to 1.13) | 0.46 | - | - | - | - |
| Sexual orientation | Straight or heterosexual | Ref | - | - | - | - | - |
| | Gay, lesbian, bisexual, or queer | 1.26 (0.85 to 1.88) | 0.26 | - | - | - | - |
| Age | Range 18 to 98 years | 1.015 (1.007 to 1.023) | <0.001* | 1.00 (0.98 to 1.02) | 0.81 | 0.98 (0.94 to 1.02) | 0.30 |
| | Quadratic term, $(\text{age} - \text{mean})^2$ | 1.0007 (1.0002 to 1.0011) | 0.002 | 1.000 (0.998 to 1.001) | 0.48 | 0.999 (0.997 to 1.001) | 0.29 |
| Region | Midlands (East and West) | Ref | - | Ref | - | Ref | - |
| | South (East, West, East of England) | 0.82 (0.61 to 1.11) | 0.20 | 0.93 (0.43 to 2.03) | 0.86 | 1.05 (0.22 to 5.12) | 0.95 |
| | North (East, West, Yorkshire and the Humber) | 0.77 (0.57 to 1.04) | 0.09 | 1.21 (0.51 to 2.87) | 0.67 | 0.48 (0.09 to 2.61) | 0.40 |
| | London | 0.79 (0.54 to 1.17) | 0.24 | 2.30 (1.04 to 5.11) | 0.04 | 0.68 (0.13 to 3.64) | 0.65 |
| | Devolved nations (Scotland, Wales, and Northern Ireland) | 0.64 (0.44 to 0.93) | 0.02 | 0.82 (0.31 to 2.13) | 0.68 | 1.91 (0.23 to 16.11) | 0.55 |
| Dependent child in household | No | Ref | - | Ref | - | Ref | - |
| | Yes | 0.85 (0.67 to 1.08) | 0.19 | 1.35 (0.49 to 3.71) | 0.56 | 1.11 (0.09 to 13.72) | 0.94 |
| Employment status | Not working | Ref | - | Ref | - | Ref | - |
| | Working | 1.34 (0.98 to 1.83) | 0.07 | 1.22 (0.61 to 2.45) | 0.57 | 2.06 (0.42 to 10.12) | 0.37 |
| Frontline health or social care worker | No | Ref | - | Ref | - | Ref | - |
| | Yes | 1.46 (1.07 to 1.99) | 0.02 | 0.41 (0.20 to 0.81) | 0.01 | 5.38 (0.90 to 32.19) | 0.07 |
| Need to leave home for work | Do not need to leave home for work | Ref | - | Ref | - | Ref | - |
| | Need to leave home for work | 0.56 (0.43 to 0.73) | <0.001* | 0.99 (0.61 to 1.60) | 0.96 | 0.28 (0.08 to 1.06) | 0.06 |
| Education | GCSE/vocational/A-level/No formal qualifications | Ref | - | Ref | - | Ref | - |
| | Degree or higher (Bachelors, Masters, PhD) | 1.18 (0.94 to 1.47) | 0.16 | 0.78 (0.49 to 1.26) | 0.31 | 1.56 (0.58 to 4.19) | 0.38 |
| Ethnicity | White British | Ref | - | Ref | - | Ref | - |



Smith LE, *et al. BMJ Open* 2023; 13:e070882. doi: 10.1136/bmjopen-2022-070882

| | | | | | | | |
|---|---|---|---|---|---|---|---|
| | White other | 0.53 (0.33 to 0.85) | 0.009 | 1.27 (0.72 to 2.26) | 0.40 | 0.59 (0.15 to 2.26) | 0.44 |
| | Black, Asian, other minoritized ethnicity | 0.81 (0.57 to 1.15) | 0.24 | 1.52 (0.78 to 2.99) | 0.22 | 0.41 (0.10 to 1.69) | 0.22 |
| Marital status | Not partnered | Ref | - | Ref | - | Ref | - |
| | Partnered | 1.02 (0.79 to 1.33) | 0.86 | 0.74 (0.43 to 1.25) | 0.26 | 2.35 (0.66 to 8.33) | 0.19 |
| Live alone | Live with someone else | Ref | - | Ref | - | Ref | - |
| | Live alone | 0.94 (0.69 to 1.27) | 0.68 | 1.30 (0.81 to 2.09) | 0.28 | 1.09 (0.40 to 2.96) | 0.86 |
| Own chronic illness | None | Ref | - | Ref | - | Ref | - |
| | Present | 1.07 (0.85 to 1.36) | 0.57 | 1.67 (1.02 to 2.74) | 0.04 | 1.12 (0.39 to 3.25) | 0.83 |
| Ever taken PrEP for HIV | No | - | - | Ref | - | Ref | - |
| | Yes | - | - | 8.95 (5.61 to 14.28) | <0.001* | 3.41 (1.11 to 10.46) | 0.03 |
| Index of multiple deprivation | Deciles (1st = most deprived, 10th = least deprived) | 1.00 (0.96 to 1.04) | 0.89 | 0.95 (0.86 to 1.05) | 0.28 | 0.94 (0.78 to 1.13) | 0.52 |
| Socio-economic grade | ABC1 | Ref | - | Ref | - | Ref | - |
| | C2DE | 0.83 (0.67 to 1.02) | 0.08 | 1.53 (0.82 to 2.87) | 0.19 | 1.39 (0.41 to 4.67) | 0.60 |
| Financial hardship | 4 (lowest hardship) to 13 (most hardship) | 0.95 (0.90 to 0.99) | 0.02 | 1.03 (0.91 to 1.18) | 0.62 | 0.95 (0.73 to 1.24) | 0.71 |
| Total number of sexual partners (male and female) in last three weeks | 0 | Ref | - | - | - | - | - |
| | 1 | 1.05 (0.80 to 1.37) | 0.72 | - | - | - | - |
| | 2 to 4 | 1.33 (0.81 to 2.18) | 0.26 | - | - | - | - |
| | 5 or more | 1.62 (0.64 to 4.09) | 0.30 | - | - | - | - |
| | Prefer not to say | 0.70 (0.54 to 0.93) | 0.01 | - | - | - | - |
| Number of male sexual partners in last three weeks | 0 | - | - | Ref | - | Ref | - |
| | 1 | - | - | 1.33 (0.69 to 2.57) | 0.40 | 1.12 (0.33 to 3.77) | 0.85 |
| | 2 to 4 | - | - | 1.70 (0.96 to 3.03) | 0.07 | 1.78 (0.56 to 5.65) | 0.33 |
| | 5 to 9 | - | - | 2.40 (1.17 to 4.95) | 0.02 | 2.14 (0.34 to 13.33) | 0.42 |
| | 10 or more | - | - | 1.53 (0.65 to 3.62) | 0.33 | 3.80 (0.28 to 52.03) | 0.32 |
| | Prefer not to say | - | - | 1.21 (0.39 to 3.73) | 0.74 | 0.72 (0.15 to 3.47) | 0.68 |
| Motivational message | Perceived risk of illness and necessity and efficacy of the response | 0.92 (0.70 to 1.20) | 0.53 | - | - | - | - |
| | Perceived risk of illness and benefits of the response | 1.15 (0.87 to 1.50) | 0.32 | - | - | - | - |
| | Perceived risk of illness and low perceived costs of response | 1.02 (0.78 to 1.34) | 0.89 | - | - | - | - |
| | Control | Ref | - | - | - | - | - |
| Motivational message | All motivational components | - | - | 0.91 (0.61 to 1.35) | 0.63 | 1.02 (0.43 to 2.39) | 0.97 |
| | Control | - | - | Ref | - | Ref | - |

*$p \leq 0.001$



Smith LE, *et al. BMJ Open* 2023; 13:e070882. doi: 10.1136/bmjopen-2022-070882

Table 2. Associations between vaccine uptake (general population sample: actual and intended, GBMSM: actual, actual and intended) and psychological and contextual factors, by sample. Variables were entered into the logistic regression model in blocks (block 1: socio-demographic variables and motivational message, block 2: psychological factors, block 3: vaccination specific factors). Results for block 3, using pooled estimates are reported.

| Factor | Level | General population<br>Vaccinated in 2022 or would be vaccinated if advised | | Grindr<br>Vaccinated in 2022 | | Vaccinated in 2022 or would be vaccinated if advised | |
|---|---|---|---|---|---|---|---|
| | | aOR (95% CI) | *p*-value | aOR (95% CI) | *p*-value | aOR (95% CI) | *p*-value |
| Amount heard about mpox | I have not seen or heard anything (1) to I have seen or heard a lot (3) | 0.93 (0.75 to 1.15) | 0.50 | 1.74 (1.09 to 2.78) | 0.02 | 0.56 (0.21 to 1.55) | 0.27 |
| Worry about mpox | Not at all worried (1) to extremely worried (4) | 1.49 (1.24 to 1.80) | <0.001* | 1.00 (0.66 to 1.52) | 0.99 | 4.33 (1.69 to 11.07) | 0.002 |
| Perceived risk of mpox to oneself | No risk at all (1) to very high risk (5) | 0.99 (0.84 to 1.17) | 0.92 | 1.25 (0.93 to 1.67) | 0.13 | 1.19 (0.63 to 2.27) | 0.59 |
| Perceived risk of mpox to people in UK | No risk at all (1) to very high risk (5) | 1.19 (1.00 to 1.40) | 0.05 | 0.85 (0.62 to 1.16) | 0.31 | 0.93 (0.43 to 2.00) | 0.85 |
| Perceived susceptibility and severity | Lowest (1) to highest (5) | 1.31 (1.11 to 1.55) | 0.001* | 0.85 (0.60 to 1.21) | 0.37 | 0.90 (0.40 to 2.05) | 0.81 |
| I am already immune to mpox | Strongly disagree, disagree, neither agree nor disagree, don't know | Ref | - | Ref | - | Ref | - |
| | Strongly agree and agree | 1.30 (0.93 to 1.83) | 0.12 | 8.83 (4.52 to 17.22) | <0.001* | 1.30 (0.27 to 6.36) | 0.74 |
| People who catch mpox usually make a full recovery, even if they do not receive any treatment | Strongly disagree (1) to strongly agree (5) | 1.01 (0.89 to 1.14) | 0.90 | 1.20 (0.94 to 1.53) | 0.15 | 0.51 (0.27 to 0.94) | 0.03 |
| My personal behaviour has an impact on how mpox spreads | Strongly disagree (1) to strongly agree (5) | 1.00 (0.91 to 1.09) | 0.99 | 0.99 (0.80 to 1.22) | 0.92 | 0.94 (0.63 to 1.39) | 0.74 |
| My life has been negatively affected by changes made in response to the mpox outbreak | Strongly disagree (1) to strongly agree (5) | 0.93 (0.84 to 1.04) | 0.23 | 0.96 (0.79 to 1.17) | 0.70 | 0.97 (0.63 to 1.51) | 0.90 |
| The risks of mpox are being exaggerated | Strongly disagree (1) to strongly agree (5) | 0.76 (0.68 to 0.85) | <0.001* | 0.78 (0.61 to 1.00) | 0.05 | 0.45 (0.27 to 0.75) | 0.002 |
| Mpox is only a risk to men who are gay, bisexual or have sex with men | Strongly disagree (1) to strongly agree (5) | 0.97 (0.88 to 1.07) | 0.56 | 0.84 (0.67 to 1.04) | 0.11 | 1.05 (0.66 to 1.65) | 0.85 |
| Perceived knowledge | Lowest (0) to highest (3) | 1.03 (0.92 to 1.14) | 0.64 | 1.25 (0.93 to 1.67) | 0.14 | 0.78 (0.43 to 1.43) | 0.42 |
| Knowledge of mpox symptoms | Identified no symptoms (0) to identified four symptoms (4) | 1.02 (0.94 to 1.10) | 0.65 | 1.01 (0.83 to 1.21) | 0.95 | 0.96 (0.67 to 1.38) | 0.82 |
| Knowledge of mpox transmission | Lowest (0) to highest (6) | 1.04 (0.96 to 1.11) | 0.34 | 0.98 (0.80 to 1.19) | 0.82 | 1.05 (0.71 to 1.55) | 0.82 |
| If I get a smallpox vaccination, I will be protected against mpox | Strongly disagree (1) to strongly agree (5) | 1.44 (1.29 to 1.61) | <0.001* | 1.33 (1.06 to 1.66) | 0.01 | 3.25 (1.97 to 5.35) | <0.001* |



Smith LE, *et al. BMJ Open* 2023; 13:e070882. doi: 10.1136/bmjopen-2022-070882

| | | | | | | | |
|---|---|---|---|---|---|---|---|
| I would be worried that having a smallpox vaccine might make me infectious to others | Strongly disagree (1) to strongly agree (5) | 0.64 (0.58 to 0.71) | <0.001* | 0.63 (0.46 to 0.87) | 0.005 | 0.44 (0.26 to 0.76) | 0.003 |

*$p \leq 0.001$



Smith LE, *et al. BMJ Open* 2023; 13:e070882. doi: 10.1136/bmjopen-2022-070882
