# Supplementary material for: Did mpox knowledge, attitudes and beliefs affect intended behaviour in the general population and men who are gay, bisexual and who have sex with men? An online cross-sectional survey in the UK
Source: BMJ Open. 2023 Oct 12;13(10):e070882. doi: 10.1136/bmjopen-2022-070882 (PMC10583036; doi:10.1136/bmjopen-2022-070882)
Supplement: Author's manuscript [file bmjopen-2022-070882.draft_revisions.pdf]

BMJ Open is committed to open peer review. As part of this commitment we make the peer review history of every article we publish publicly available.

When an article is published we post the peer reviewers' comments and the authors' responses online. We also post the versions of the paper that were used during peer review. These are the versions that the peer review comments apply to.

The versions of the paper that follow are the versions that were submitted during the peer review process. They are not the versions of record or the final published versions. They should not be cited or distributed as the published version of this manuscript.

BMJ Open is an open access journal and the full, final, typeset and author-corrected version of record of the manuscript is available on our site with no access controls, subscription charges or pay-per-view fees (http://bmjopen.bmj.com).

If you have any questions on BMJ Open's open peer review process please email info.bmjopen@bmj.com

BMJ Open

Mpox knowledge, attitudes, beliefs, and intended behaviour in the general population and men who are gay, bisexual, and who have sex with men

Journal:	*BMJ Open*
Manuscript ID	bmjopen-2022-070882
Article Type:	Original research
Date Submitted by the Author:	12-Dec-2022

Complete List of Authors:	Smith, Louise; King's College London<br>Potts, Henry; University College London, Institute of Health Informatics<br>Brainard, Julii; University of East Anglia, Norwich Medical School<br>May, Tom; University of Bristol<br>Oliver, Isabel; UK Health Security Agency<br>Amlôt, Richard; UK Health Security Agency<br>Yardley, Lucy; University of Bristol<br>Rubin, G James; King's College London

Keywords:	Public health < INFECTIOUS DISEASES, PUBLIC HEALTH, SEXUAL MEDICINE

SCHOLARONE™
Manuscripts

I, the Submitting Author has the right to grant and does grant on behalf of all authors of the Work (as defined in the below author licence), an exclusive licence and/or a non-exclusive licence for contributions from authors who are: i) UK Crown employees; ii) where BMJ has agreed a CC-BY licence shall apply, and/or iii) in accordance with the terms applicable for US Federal Government officers or employees acting as part of their official duties; on a worldwide, perpetual, irrevocable, royalty-free basis to BMJ Publishing Group Ltd ("BMJ") its licensees and where the relevant Journal is co-owned by BMJ to the co-owners of the Journal, to publish the Work in this journal and any other BMJ products and to exploit all rights, as set out in our licence.

The Submitting Author accepts and understands that any supply made under these terms is made by BMJ to the Submitting Author unless you are acting as an employee on behalf of your employer or a postgraduate student of an affiliated institution which is paying any applicable article publishing charge ("APC") for Open Access articles. Where the Submitting Author wishes to make the Work available on an Open Access basis (and intends to pay the relevant APC), the terms of reuse of such Open Access shall be governed by a Creative Commons licence – details of these licences and which Creative Commons licence will apply to this Work are set out in our licence referred to above.

Other than as permitted in any relevant BMJ Author's Self Archiving Policies, I confirm this Work has not been accepted for publication elsewhere, is not being considered for publication elsewhere and does not duplicate material already published. I confirm all authors consent to publication of this Work and authorise the granting of this licence.

For peer review only

Mpox knowledge, attitudes, beliefs, and intended behaviour in the general population and men who are gay, bisexual, and who have sex with men

Louise E Smith (0000-0002-1277-2564),[1,2] PhD, Henry WW Potts (0000-0002-6200-8804),[3] PhD, Julii Brainard (0000-0002-5272-7995),[2,4] PhD, Tom May (0000-0003-3077-523X),[5,6,7] Isabel Oliver (0000-0002-6106-1734),[5,9] PhD, Richard Amlôt (0000-0003-3481-6588),[2,5,9] PhD, Lucy Yardley (0000-0002-3853-883X),[5,7,8] PhD, G James Rubin,[1,2] PhD

1 King's College London, Institute of Psychiatry, Psychology and Neuroscience

2 NIHR Health Protection Research Unit in Emergency Preparedness and Response

3 University College London, Institute of Health Informatics

4 University of East Anglia, Norwich Medical School

5 NIHR Health Protection Research Unit in Behavioural Science and Evaluation, University of Bristol

6 University of Bristol, Bristol Medical School

7 University of Bristol, School of Psychological Science

8 University of Southampton, School of Psychology

9 UK Health Security Agency, Chief Scientific Officer's Group

Corresponding author: Louise E Smith, Research Fellow. Department of Psychological Medicine, King's College London, Weston Education Centre, Cutcombe Road, London, SE5 9RJ, UK. Email: louise.e.smith@kcl.ac.uk

Henry WW Potts, Professor of Health Informatics. Institute of Health Informatics, 222 Euston Road, London, NW1 2DA, UK.

For peer review only - http://bmjopen.bmj.com/site/about/guidelines.xhtml

Julii Brainard, Senior Research Associate. Norwich Medical School, University of East Anglia, Norwich, Norfolk, NR4 7TJ, UK.

Tom May, Research Fellow, NIHR Health Protection Research Unit in Behavioural Science and Evaluation, Bristol Medical School, University of Bristol, Bristol, BS8 1TU, UK.

Isabel Oliver, Chief Scientific Officer, UK Health Security Agency, 17 Smith Square, London, SW1P 3HX, UK.

Richard Amlôt, Interim Deputy Director Behavioural Science and Insights, UK Health Security Agency, 17 Smith Square, London, SW1P 3HX, UK.

Lucy Yardley, Professor of Health Psychology. School of Psychological Science, University of Bristol, 12A Priory Rd, Bristol BS8 1TU, UK.

G James Rubin, Director, NIHR Health Protection Research Unit in Emergency Preparedness and Response, King's College London, Weston Education Centre, Cutcombe Road, London, SE5 9RJ, UK.

For peer review only

ABSTRACT

Objectives: To investigate rates of mpox beliefs, knowledge, and intended behaviours in the general population and in gay, bisexual or other men who have sex with men (GBMSM), and factors associated with intended behaviours. To test the impact of motivational messages (vs a factual control) on intended behaviours.

Design: Cross-sectional online survey including a nested randomised controlled trial.

Setting: Data collected 5 September to 6 October 2022.

Participants: Participants were aged 18 years and over and lived in the UK (general population). In addition, GBMSM were male, and gay, bisexual or had sex with men. The general population sample was recruited through a market research company. GBMSM were recruited through a market research company, the dating app Grindr, and targeted adverts on Meta (Facebook and Instagram).

Main outcome measures: Intention to self-isolate, seek medical help, stop all sexual contact, share details of recent sexual contacts, and accept vaccination.

Results: Socio-demographic characteristics differed by sample. There was no effect of very brief motivational messaging on behavioural intentions. Respondents from Grindr and Meta were more likely to intend to seek help immediately, completely stop sexual behaviour and be vaccinated or intend to be vaccinated, but being less likely to intend to self-isolate (*p*s<0.001). In the general population sample, intending to carry out protective behaviours was generally associated with being female, older, having less financial hardship, greater worry, higher perceived risk to others, and higher perceived susceptibility to and severity of mpox (*p*s<0.001). There were fewer associations with behaviours in the Grindr sample, possibly due to reduced power.

Conclusions: GBMSM were more likely to intend to enact protective behaviours, except for self-isolation. This may reflect targeted public health efforts and engagement with this group. Associations with socio-economic factors suggests that providing financial support may encourage people to engage with protective behaviours.

Word count: 298/300

Key words: adherence; self-isolation; symptoms; testing; mpox; psychological factors

For peer review only - http://bmjopen.bmj.com/site/about/guidelines.xhtml

STRENGTHS AND LIMITATIONS

- Anonymous cross-sectional survey in large samples of the general population and men who are gay, bisexual, or have sex with men (recruited from a market research company, the dating app Grindr, and targeted adverts on Meta [Facebook and Instagram]).
- Data collection occurred over a short period (5 September to 6 October 2022) during the mpox outbreak.
- Responses may have been affected by social desirability or recall bias, although the anonymous nature of the survey should mitigate this somewhat.
- Socio-demographic characteristics differed by sample. Participants recruited from Grindr and Meta were more likely to be working, highly educated, of higher socio-economic grade, and have less financial hardship.
- We measured behavioural intentions. Rates of engagement with behaviours may be lower. Factors associated with intentions should still be valid.

For peer review only

For peer review only - http://bmjopen.bmj.com/site/about/guidelines.xhtml

INTRODUCTION

Mpox (also known as monkeypox) is an orthopox virus that causes fever, headache, exhaustion, swollen glands and aches (joint, muscle, back), followed by a rash with blisters.(1) It spreads from person to person through touching clothing, bedding or towels used by someone with mpox rash, touching mpox skin blisters or scabs, and through the coughs or sneezes of someone with mpox rash (droplet transmission).(1) Since May 2022, there has been a multi-country outbreak of mpox in non-endemic countries.(2) Estimates from the World Health Organization (WHO; data up to 1 December 2022), indicate that there have been over 81,000 cases in 110 countries, resulting in 59 deaths.(3) Most cases have been in men who are gay, bisexual, or have sex with men, with close human skin-to skin contact (including sexual) being the primary driver of transmission.(4) The UK is the sixth country most affected by this mpox outbreak with 3725 cases.(3) Within the UK, most cases have been identified in England (with 69% of English cases in London). Almost all (99%) cases were men; English cases had a median age of 37 years.(5) The peak of the epidemic was seen in June and July 2022, with case numbers falling since the end of July.(6)

In the UK, people who thought they might have mpox were asked to call a sexual health clinic, to stay at home (self-isolate) and avoid close contact with other people.(1) Suspected cases were tested for mpox, and confirmed cases were asked to self-isolate for up to 21 days and engage with contact tracing.(7) Cases, their close contacts, and those most likely to be exposed to mpox were advised to be vaccinated with modified vaccinia Ankara vaccine – which offers cross-protection – to reduce transmission and prevent severe illness.(8, 9) While similar public health actions are now familiar to the public as a result of the COVID-19 pandemic, research conducted during the pandemic indicates that engagement with uptake of testing and self-isolation was sub-optimal in some groups.(10-12)

Various theories have guided research into the psychological factors associated with the uptake of health behaviours. One such theory is the Protection Motivation Theory (PMT), which states that people's intention to carry out a protective behaviour is influenced by their appraisal of the threat (perceived susceptibility to and severity of, e.g. mpox) and the coping response (perceived effectiveness of and self-efficacy for, e.g. testing, self-isolation, contact tracing, vaccination).(13) During the COVID-19 pandemic, psychological, and socio-demographic factors associated with testing and self-isolating included: higher perceived risk of COVID-19, knowledge of transmission modes, higher perceived effectiveness of protective behaviours, believing that your behaviour had an impact on transmission, and

For peer review only - http://bmjopen.bmj.com/site/about/guidelines.xhtml

belief that others in the same position would also self-isolate, being female, and having less financial hardship.(10-12, 14) Generally speaking, engagement with protective behaviours was associated with being older, apart from uptake of lateral flow testing, which was higher in younger people.(10, 11) Factors associated with COVID-19 vaccine uptake (completed and intended) included perceiving vaccination to be safe and necessary, perceiving COVID-19 to be more severe, and thinking that others would also be vaccinated.(15-18) Perception of side effects is one of the most common reasons given for refusing vaccination.(19) Historically, smallpox vaccines have been associated with severe adverse effects.(8) While vaccines currently licensed are associated with fewer severe adverse effects, this may affect people's intention to be vaccinated.

At the time of writing, few scientific studies have investigated behaviour during the 2022 mpox epidemic. While most have investigated vaccination acceptability, few have investigated engagement with a contact tracing system. Those studies that have been done suggest that knowledge of transmission modes is incomplete.(20-22) Greater agreement with vaccination for mpox was associated with perceiving the virus as more dangerous and virulent and higher worry about mpox in a survey of members of the Saudi Arabian general public.(23) In Dutch GBMSM, willingness to accept a vaccine was associated with being more worried about getting mpox, perceiving a higher risk of mpox, perceiving mpox to be more severe, thinking that vaccination is important, thinking that the vaccine was effective, and greater social norms for vaccination.(24) Another study also conducted in the Netherlands found that vaccination intention in GBMSM was associated with higher worry about mpox, knowing someone who had mpox, and being single but dating or in an open/polyamorous relationship.(25) This study also investigated self-isolation intention for 21 days, finding that higher intentions were associated with thinking that mpox had more problematic consequences, and lower education.(25) A study conducted in the UK found that agreement with self-isolation was associated with not having completed a higher education degree, not being employed, and identifying as having a disability.(26) There was no difference in agreement between GBMSM and those who were not GBMSM. Further research is needed to investigate how psychological factors may affect engagement with public health measures (isolating, testing, contact tracing, vaccination) put in place to control the spread of the mpox outbreak in the UK.

Official communications are vital during new and emerging outbreaks, and serve to inform the public about the threat, the public health response, and behaviours that people should

For peer review only - http://bmjopen.bmj.com/site/about/guidelines.xhtml

engage with in order to protect themselves and others.(27) Messages based on theories of health behaviours, such as the PMT,(13) may therefore increase engagement with protective behaviours. For example, research suggests that COVID-19 vaccination intention increased when communications emphasised the safety and effectiveness of the vaccine and using social norms interventions (e.g. asking people to “join the millions” being vaccinated).(28) While findings relating to messages emphasising the benefits of vaccination to oneself and others were mixed, there is some evidence that the influence of these messages may be most evident in strongly hesitant groups.

In this study, we recruited a general population sample and three GBMSM samples from: a market research company, Grindr, and Meta (Facebook and Instagram). We investigated knowledge, attitudes and beliefs about mpox, and intentions for key behaviours that could prevent the spread of mpox (self-isolation, help seeking [as the advised route into testing], sexual contact behaviour when symptomatic, contact sharing, vaccination). We used an experimental approach to investigate the impact of different brief communication approaches (promoting perceived susceptibility to and severity of illness / necessity and efficacy of the response / benefits of the response / low perceived costs of response) on intended behaviour. Psychological and socio-demographic factors associated with intended uptake of behaviours were also investigated.

METHOD

Design

Online cross-sectional survey conducted by Savanta, a Market Research Society (MRS) Company Partner. Data were collected between 5 September and 6 October 2022.

Participants

Eligibility criteria for the general population sample were living in the UK and being aged 18 years or over. For the GBMSM samples, additional criteria were being a man and identifying as being gay, bisexual, or having sex with men.

Recruitment for the general population sample used quota sampling, a standard opinion polling method that allows for rapid data collection. Members of Savanta’s specialist research panel (n=150,000 across proprietary panels; people who have signed up to complete online surveys) were sent the survey link. Quota sampling uses pre-determined targets, based on pre-selected socio-demographic characteristics (quotas) that match the national population.

For peer review only - http://bmjopen.bmj.com/site/about/guidelines.xhtml

Participants who belong to a quota that has already been filled are prevented from completing the survey. Therefore, response rate is not an accurate measure of response bias in quota samples. For this study, quotas were based on age, gender, socio-economic grade and Government Office Region, and reflected targets based on 2020 mid-year estimates.(29)

We recruited three GBMSM samples (Savanta, Grindr, Meta [Facebook and Instagram]). A "boost" sample of 250 GBMSM was recruited by Savanta, using the same quota sampling (excluding gender as all participants were male). We also recruited for the GBMSM sample through a push-notification inbox advert on Grindr (1.1 million adverts delivered, 24,933 opened, and 4288 clicks) and using targeted adverts on Meta (308,472 adverts shown, 108,865 adverts seen, and 3675 clicks). No quotas were placed on these samples.

Study materials

Full study materials are in supplementary materials 1. Items were based on previously validated measures,(30, 31) and items used in previous surveys by our group.(11, 14, 32, 33)

Outcome measures

Self-isolation intention was measured using two items, asking participants to imagine that they were contacted by public health officials and told that they needed to self-isolate for 21 days because they had mpox, and because they had come into high-risk contact with a case. Responses were given on a five-point scale from "definitely would not" to "definitely would". The order of items was randomised between participants to mitigate potential order effects. As answers on both items were highly correlated across all respondents (r = 0.77, p = 0.001), we combined responses to give a nine-point scale (2 to 10; summing responses "definitely would not" = 1 to "definitely would" = 5; higher number indicates greater intention).

To measure help-seeking intention, participants were asked to imagine that they developed an unexplained rash with blisters and learned that they had come into contact with a mpox case. They were then asked what they would do, from a list of ten behaviours including waiting to see if they got better, contacting healthcare services, letting people you had been in recent close contact know, and searching for information. Responses for each item were given on the same five-point "definitely would not" to "definitely would" scale. We created a binary variable, coding participants as intending to seek help immediately if they answered "probably" or "definitely would" to any help seeking where they would encounter a health professional (trying to book an appointment with a general practitioner, visiting a

For peer review only - http://bmjopen.bmj.com/site/about/guidelines.xhtml

pharmacist/chemist, going to Accident & Emergency or another NHS service, calling NHS111 or 999, visiting a walk-in sexual health clinic, or calling a sexual health clinic), and did not select “wait a day or two to see if they get better or clear up on their own” vs did not select any help-seeking behaviour where they would encounter a health professional, or selected a help-seeking behaviour but also stated that they would “wait a day or two to see…”.

Participants were then asked about their intended contact behaviour in the same scenario, being asked “in the following 21 days, realistically how much” they would come into skin-to skin contact with others, have sexual contact, have sex without using a condom, go to a crowded place, help or provide care for a vulnerable person, and go to a public place where they may come into physical contact with someone else. For each item, participants responded “I would completely stop doing this”, “less than normal”, “same as normal”, “more than normal”, “not applicable, I wouldn’t do this anyway”, “don’t know” or “prefer not to say”. We focused our analyses on the item asking about sexual contact (from kissing to intercourse) with other people, recoding it into a binary item (“I would completely stop doing this” vs would do this less than, same as, or more than usual). Answers of “not applicable, I wouldn’t do this anyway”, “don’t know”, and “prefer not to say” were coded as missing.

We measured intention to share details of close contacts by asking participants to indicate how likely they were, if asked by public health officials, to share contact details of every person who had been in their home, they had had sexual contact with, they had skin-to-skin contact with, and that they had shared bedding, towels or clothes with in the last seven days, and every place they had had sexual contact with someone. Responses were given on a five-point “definitely would not” (1) to “definitely would” (5) scale. We used the most recent official guidance on contact tracing available at the start of data analysis to select the item most relevant to contact tracing efforts (identifying every person you had sexual contact with in the last seven days).(7)

Participants were asked if they had received a smallpox vaccine in 2022. Those who indicated they had not had a vaccine were asked about their vaccination intention. We asked participants how likely they would be to have a smallpox vaccine if they were offered one. Responses were given on a five-point “definitely would not” (1) to “definitely would” (5) scale.

For peer review only - http://bmjopen.bmj.com/site/about/guidelines.xhtml

Motivational messaging

Very brief motivational messages were constructed based on components of the PMT.(13) The general population sample were randomised to one of four groups, and were shown messages about: 1) perceived risk of illness and necessity and efficacy of the response, 2) perceived risk of illness plus benefits of the response, 3) perceived risk of illness plus low perceived costs of response, and 4) a control message of similar length giving factual details about the mpox outbreak. Messages are shown in supplementary materials 1. Messages to promote perceived risk of illness (i.e. susceptibility to and severity of mpox) were included in all motivational messages, as we hypothesised that perceiving a risk is necessary before choosing to adopt a behaviour to mitigate that risk. Due to anticipated smaller sample sizes, the GBMSM samples were randomised to one of two messages. The first included all motivational components, whereas the second, a control message, gave factual information about the mpox outbreak.

Psychological factors

We asked participants how much they had seen or heard about mpox, how worried they were about mpox, and how much risk they thought mpox posed to people in the UK and themselves personally. For these items, answers of "don't know" were coded as missing.

Participants were also asked about their perceived susceptibility to mpox (two items: would be likely to come into contact with a case, would be likely to catch mpox if in contact with a case) and perceived severity of mpox. We asked participants how much they agreed that: their personal behaviour had an impact on the spread of mpox; their life had been negatively affected by changes made in response to the mpox outbreak; the risks of mpox were being exaggerated; people who catch mpox usually make a full recovery without treatment; and that mpox is only a risk to men who are gay, bisexual, or have sex with men. For perceptions such as these, with no right or wrong answers, we recoded answers of "don't know" as the mid-point on the scale.

To measure perceived knowledge, we asked participants three items about whether they had a good idea how people catch mpox, they knew the main symptoms of mpox, and they thought it would be easy to tell if someone had mpox. Knowledge about mpox symptoms was measured using a question asking participants to identify the main symptoms of mpox from a list of fifteen taken from the NHS mpox website (1) and common, non-specific symptoms. Participants could select up to four symptoms. Understanding of transmission was measured using seven items, asking about contact and droplet transmission (adapted from (30), and

For peer review only - http://bmjopen.bmj.com/site/about/guidelines.xhtml

other modes of transmission as specified by the World Health Organization (WHO) website (34)). For factual questions such as these, we coded "don't know" as missing.

Behaviour-specific perceptions were also investigated. We used a series of ten items to measure factors that may be associated with self-isolation, including perceived effectiveness, social norms, having the necessary support, and impact on social connectedness, family wellbeing, and finances. Factors that may affect intention to seek help were measured by six items asking participants to what extent they agreed that they would not want to know the results of a mpox test, they would be worried what their friends, family, or employers thought of them if they had mpox, not wanting to have a mpox test on their medical record, testing is an effective way to prevent the spread of mpox, and being willing to contact a sexual health clinic if they thought they had mpox symptoms or had come into contact with a case. Vaccination attitudes were measured by eight items, asking about general vaccine attitudes, perceived social norms, perceived effectiveness of vaccination, worry about vaccine side-effects, that the vaccine could make you infectious to others, and thinking that those who come into high-risk contact with mpox should be vaccinated. Responses of "don't know" were recoded to the mid-point of the scale.

Socio-demographic characteristics

Participants were asked to report their age, gender,(35) sexual orientation,(36) socio-economic grade,(37) financial hardship (adapted from (38)), employment status, highest level of education, ethnicity, marital status, how many people lived in their household, whether they were the parent or guardian of any dependent children, and if they had any pets. Those who were employed were asked if they were a frontline health or social care worker and if they needed to leave home for work. For these items, we coded participants who were not employed as not being a frontline health or social care worker (prefer not to say coded as missing) and not needing to leave home for work, respectively. Participants were asked for their full postcode, from which region and indices of multiple deprivation were determined.(39)

We asked participants if they or a household member had a chronic illness, whether they were pregnant, had ever taken pre-exposure prophylaxis (PrEP) for HIV, and for their vaccination status for smallpox (in 2022, and before 2022), hepatitis A, and COVID-19 (two doses or more).

For peer review only - http://bmjopen.bmj.com/site/about/guidelines.xhtml

For peer review only

Participants were also asked how many male and female sexual partners they had had in the last 3 weeks and last 3 months.

Ethics

Ethical approval for this study was given by the King's College London Psychiatry, Nursing and Midwifery Research Ethics Panel (reference number: LRS/DP-21/22-32287). Participants gave informed consent before beginning survey materials.

Patient and public involvement

To ensure the research aims and study information were appropriate, members of the public were involved in the development of the funding application and survey materials. For the funding application, six people gave feedback on the initial proposal resulting in changes to aims of the study and terminology used. For the survey materials, four lay people (two GBMSM) gave feedback on the questionnaire and motivational messages, resulting in changes to survey items and messages to improve clarity, validity, and readability of statements.

Power

A sample size of 3000 allows a 95% confidence interval of plus or minus 1.8% for the prevalence estimate for a survey item with a prevalence of around 50% (sample size of 250 gives a confidence interval of plus or minus 6.2%; sample size of 1000 gives a confidence interval of plus or minus 3.1%).

For multiple linear regression analyses, a sample of 830 allowed over 99% power to detect small effect sizes (f=0.10) at p=0.001 (43 predictors). For logistic regression analyses, a sample of 830 allowed over 99% power to detect small effect sizes (OR=1.68 (40)) at p=0.001 (42.5% self-isolation, 18.0% requesting a test, 79.1% sharing details of close contacts (11)).

Analysis

Information about data preparation is reported in supplementary materials 2.

We tested whether socio-demographic characteristics of GBMSM samples were different depending on the recruitment method (Savanta, Grindr, Meta). Due to significant differences, further analyses were conducted in each sample separately.

For peer review only - http://bmjopen.bmj.com/site/about/guidelines.xhtml

First, we tested the influence of motivational messages on outcomes using χ^2 tests (binary outcomes), one-way ANOVAs (general population sample, continuous outcomes), or t-tests (GBMSM, continuous outcomes). For vaccination, we investigated the influence of motivational message on intention to be vaccinated if advised (excluding people already vaccinated).

Next, we investigated psychological and contextual factors associated with intended behaviours (self-isolation, help seeking, sexual contact, sharing details of contacts, vaccination). To minimise analyses conducted, we investigated one variable per outcome, except for vaccination (GBMSM sample investigated two outcomes). For vaccination, as smallpox vaccine uptake in GBMSM was high, we used two binary outcomes: vaccination uptake in 2022 (vaccinated vs not vaccinated), and a computed variable indicating vaccine intention and uptake (vaccinated or intend to be vaccinated ["definitely" or "probably would"] vs not vaccinated and do not intend to be vaccinated ["not sure", "probably would not", "definitely would not"]). For the general population sample, we used only the computed variable. We conducted regressions (binary logistic for binary outcomes, linear for continuous outcomes) in the general population sample and Grindr sample (the target GBMSM sample who were actively seeking new partners; Savanta sample excluded due to small numbers, Meta sample excluded as they differed significantly from the general population).

We entered variables into regressions in blocks. In the first block, we entered socio-demographic characteristics: gender (general population sample only, male / female), sexual orientation (general population sample only, straight or heterosexual / gay, lesbian, bisexual, or queer), age, region, having a dependent child in household (no / yes), employment status (working / not working), education (GCSE, vocational, A-level, no formal qualifications / degree or higher), ethnicity (White British / White other / Black, Asian, other minoritized ethnicity), marital status, living alone, having a chronic illness oneself (none / present), index of multiple deprivation (deciles), socio-economic grade (ABC1 / C2DE), financial hardship, and motivational message. In the Grindr sample, we also included smallpox vaccination status in 2022 (except for vaccine uptake outcome) and ever having taken PrEP for HIV.

In the second block, we entered psychological and contextual factors: self-reported knowledge, knowledge of mpox symptoms, knowledge of mpox transmission, amount heard about mpox, worry about mpox, perceived risk of mpox (to oneself and to people in the UK), perceived susceptibility and severity of mpox, thinking that you are immune to mpox, that

For peer review only - http://bmjopen.bmj.com/site/about/guidelines.xhtml

your personal behaviour has an impact on how mpox spreads, that your life has been negatively affected by changes made in response to the mpox outbreak, that the risks of mpox are being exaggerated, that mpox is only a risk to men who are gay, bisexual or have sex with men, and that people who catch mpox usually make a full recovery even without treatment.

For self-isolation, help seeking and vaccination, a third block was also added, which included specific factors potentially associated with individual outcomes. Items were chosen through principal component analyses (see supplementary materials 3).

All analyses were carried out in SPSS 28. Data are unweighted.

Many comparisons were investigated in regression models (n=40 to n=43, based on outcome). Therefore, we applied a conservative Bonferroni correction and only report as significant results with $p \leq 0.001$ to reduce the risk of Type I errors.

RESULTS

Top-line results for all survey materials, by sample, are shown in supplementary materials 1.

For regression analyses, we report imputed values. Results using imputed values were compared with non-imputed data. There were no substantial differences in results with and without imputed values.

Participant characteristics

There were significant differences in participant characteristics by sampling method. Most notably, participants recruited from Grindr and Meta were more likely to be working, need to leave home for work, more highly educated, higher socio-economic grade, and have less financial hardship (Table 1). In the general population sample, participants were mostly female (57%), white British (87%), with a mean age of 49 years.

Table 1. Participant characteristics, by recruitment method.

		General population, n=3050	GBMSM samples			
			Savanta GBMSM, n=247	Grindr, n=831	Meta, n=1036	p-value
Gender	Male (including trans man)	1289 (42.4)	247 (100.0)	831 (100.0)	1036 (100.0)	-
	Female (including trans woman)	1736 (57.1)	-	-	-	-
	Non-binary	17 (0.6)	-	-	-	-
Sexual orientation	Straight or heterosexual	2795 (92.7)	-	-	-	-
	Gay, lesbian, bisexual, or queer	221 (7.3)	247 (100.0)	831 (100.0)	1036 (100.0)	-

For peer review only - http://bmjopen.bmj.com/site/about/guidelines.xhtml

Age	Range 18 to 98 years	M=48.6, SD=17.4	M=47.1, SD=16.5	M=44.2, SD=12.5	M=47.6, SD=11.9	<0.001*
Region†	Midlands (East and West)	522 (17.1)	39 (15.8)	73 (8.8)	85 (8.2)	<0.001*
	South (East, West, East of England)	992 (32.5)	70 (28.3)	207 (24.9)	279 (26.9)	
	North (East, West, Yorkshire and the Humber)	774 (25.4)	58 (23.5)	119 (14.3)	155 (15.0)	
	London	308 (10.1)	46 (18.6)	195 (23.5)	379 (36.6)	
	Devolved nations (Scotland, Wales, and Northern Ireland)	454 (14.9)	34 (13.8)	95 (11.4)	84 (8.1)	
	Not specified	0 (0.0)	0 (0.0)	142 (17.1)	54 (5.2)	
Dependent child in household	No	2075 (68.0)	210 (85.0)	796 (95.8)	1014 (97.9)	<0.001*
	Yes	975 (32.0)	37 (15.0)	35 (4.2)	22 (2.1)	
Employment status	Not working	1286 (42.6)	93 (38.3)	157 (19.0)	201 (19.5)	<0.001*
	Working	1736 (57.4)	150 (61.7)	669 (81.0)	831 (80.5)	
Frontline health or social care worker	No	2668 (88.3)	224 (90.7)	734 (88.6)	909 (87.9)	0.66
	Yes	355 (11.7)	23 (9.3)	94 (11.4)	125 (12.1)	
Need to leave home for work	Do not need to leave home for work	1946 (63.8)	149 (60.3)	371 (44.6)	499 (48.2)	<0.001*
	Need to leave home for work	1104 (36.2)	98 (39.7)	460 (55.4)	537 (51.8)	
Education	GCSE/vocational/A-level/No formal qualifications	2065 (67.7)	159 (64.4)	295 (35.5)	253 (24.4)	<0.001*
	Degree or higher (Bachelors, Masters, PhD)	985 (32.3)	88 (35.6)	536 (64.5)	783 (75.6)	
Ethnicity	White British	2649 (87.1)	214 (86.6)	629 (76.2)	799 (78.0)	<0.001*
	White other	116 (3.8)	18 (7.3)	117 (14.2)	163 (15.9)	
	Black, Asian, other minoritized ethnicity	275 (9.0)	15 (6.1)	80 (9.7)	63 (6.1)	
Marital status	Not partnered	1265 (41.6)	141 (57.3)	561 (68.3)	486 (47.3)	<0.001*
	Partnered	1775 (58.4)	105 (42.7)	260 (31.7)	541 (52.7)	
Live alone	Live with someone else	2387 (78.3)	146 (59.1)	473 (56.9)	656 (63.3)	0.02
	Live alone	663 (21.7)	101 (40.9)	358 (43.1)	380 (36.7)	
Own chronic illness	None	2207 (72.4)	167 (67.6)	619 (74.5)	731 (70.6)	0.05
	Present	843 (27.6)	80 (32.4)	212 (25.5)	305 (29.4)	
Household member chronic illness	None	2659 (87.2)	226 (91.5)	743 (89.4)	921 (88.9)	0.49
	Present	391 (12.8)	21 (8.5)	88 (10.6)	115 (11.1)	
Ever taken PrEP for HIV	No	2993 (98.6)	216 (88.5)	427 (51.5)	547 (52.9)	<0.001*
	Yes	42 (1.4)	28 (11.5)	402 (48.5)	487 (47.1)	
Vaccinated for smallpox in 2022	Not vaccinated	2888 (94.7)	221 (89.5)	566 (68.1)	614 (59.3)	<0.001*
	Vaccinated	162 (5.3)	26 (10.5)	265 (31.9)	422 (40.7)	
Index of multiple deprivation†	Deciles (1st = most deprived, 10th = least deprived)	M=4.5, SD=2.9	M=4.4, SD=3.0	N=689, M=4.7, SD=2.8	N=982, M=4.9, SD=2.7	<0.001*
Socio-economic grade	ABC1	1796 (58.9)	169 (68.4)	694 (83.5)	944 (91.1)	<0.001*
	C2DE	1254 (41.1)	78 (31.6)	137 (16.5)	92 (8.9)	
Financial hardship†	4 (lowest hardship) to 13 (most hardship)	N=2743, M=6.1, SD=2.3	N=223, M=5.9, SD=2.4	N=784, M=5.2, SD=1.8	N=1004, M=4.7, SD=1.3	<0.001*

*$p \leq 0.001$
†Using original (not imputed) data.

Motivational messaging

There was no effect of motivational messaging on outcomes, except for in the sample recruited from Meta (see supplementary materials 4). In this sample, those receiving the

For peer review only - http://bmjopen.bmj.com/site/about/guidelines.xhtml

motivational message were less likely to intend to self-isolate for 21 days ($t(1034) = -2.81$, $p=0.005$; motivational message, n=529, M=7.1, SD=2.3; control message, n=507, M=7.5, SD=2.2), share details of all recent sexual contacts ($t(1029) = -2.05$, $p=0.04$; motivational message, n=526, M=4.1, SD=1.2; control message, n=505, M=4.2, SD=1.1), and be vaccinated for smallpox if advised ($t(612) = -2.21$, $p=0.03$; motivational message, n=304, M=4.7, SD=0.9; control message, n=310, M=4.8, SD=0.6).

Self-isolation

Rates of intended self-isolation were higher when imagining you were a case than a high-risk contact. Three-quarters of the general population sample intended to self-isolate for 21 days if they were to develop mpox (75.2%, 95% CI 73.7% to 76.7%, n=2294; Table 2). However, only 68.9% (95% CI 67.2% to 70.5%, n=2100) intended to self-isolate if they were to come into contact with a case. Intention to self-isolate was lower in Grindr and Meta samples.

For peer review only

Table 2. Main behavioural outcomes, by sample.

			**N (%, 95% CI)**				
			General population, total n=3050	Savanta GBMSM, total n=247	Grindr, total n=831	Meta, total n=1036	p-value
**Self-isolation for 21 days**	If "you have" mpox	Probably or definitely would not, or not sure	756 (24.8, 23.3-26.3)	53 (21.5, 16.3-26.6)	246 (29.6, 26.5-32.7)	275 (26.5, 23.9-29.2)	-
		Probably or definitely would	2294 (75.2, 73.7-76.7)	194 (78.5, 73.4-83.7)	585 (70.4, 67.3-73.5)	761 (73.5, 70.8-76.1)	
	If "you have come into high-risk contact with someone who has" mpox	Probably or definitely would not, or not sure	950 (31.1, 29.5-32.8)	77 (31.2, 25.4-37.0)	371 (44.6, 41.3-48.0)	491 (47.4, 44.3-50.4)	-
		Probably or definitely would	2100 (68.9, 67.2-70.5)	170 (68.8, 63.0-74.6)	460 (55.4, 52-58.7)	545 (52.6, 49.6-55.7)	
	Sum of intention if case and high-risk contact	2 (lowest intention) to 10 (highest intention)	M=8.0, SD=2.2	M=7.9, SD=2.3	M=7.2, SD=2.4	M=7.3, SD=2.3	<0.001*
**Help seeking**	Any action that involves contacting a healthcare professional (by phone or in person)	Would not seek help or would "wait and see" for a day or two to see if symptoms resolved	1548 (50.8, 49.0-52.5)	125 (50.6, 44.3-56.9)	388 (46.7, 43.3-50.1)	391 (37.7, 34.8-40.7)	<0.001*
		Would seek help immediately	1502 (49.2, 47.5-51.0)	122 (49.4, 43.1-55.7)	443 (53.3, 49.9-56.7)	645 (62.3, 59.3-65.2)	
**Sexual contact behaviour †**	Have sexual contact with others	Would not completely stop	567 (22.8, 21.1-24.4)	45 (20.8, 15.4-26.3)	69 (8.7, 6.8-10.7)	69 (7.0, 5.4-8.6)	<0.001*
		Completely stop	1923 (77.2, 75.6-78.9)	171 (79.2, 73.7-84.6)	721 (91.3, 89.3-93.2)	922 (93.0, 91.4-94.6)	
**Contact sharing**	Sexual contacts	Probably or definitely would not, or not sure	697 (23.3, 21.7-24.8)	60 (24.7, 19.2-30.2)	167 (20.3, 17.5-23.0)	204 (19.8, 17.4-22.2)	-
		Probably or definitely would	2300 (76.7, 75.2-78.3)	183 (75.3, 69.8-80.8)	656 (79.7, 77-82.5)	827 (80.2, 77.8-82.6)	

For peer review only - http://bmjopen.bmj.com/site/about/guidelines.xhtml

	Scale	1 (definitely would not) to 5 (definitely would)	N=2997, M=4.2, SD=1.2	N=243, M=4.1, SD=1.2	N=823, M=4.1, SD=1.2	N=1031, M=4.2, SD=1.1	0.57
**Vaccination intention**	Vaccinated in 2022	No or not sure	2888 (94.7, 93.9-95.5)	221 (89.5, 85.6-93.3)	566 (68.1, 64.9-71.3)	614 (59.3, 56.3-62.3)	<0.001*
		Yes	162 (5.3, 4.5-6.1)	26 (10.5, 6.7-14.4)	265 (31.9, 28.7-35.1)	422 (40.7, 37.7-43.7)	
	Vaccinated in 2022 or would be vaccinated if advised	Not vaccinated and probably or definitely would not be vaccinated or not sure	786 (25.8, 24.2-27.3)	37 (15.0, 10.5-19.5)	54 (6.5, 4.8-8.2)	38 (3.7, 2.5-4.8)	<0.001*
		Vaccinated or probably or definitely would be vaccinated	2264 (74.2, 72.7-75.8)	210 (85.0, 80.5-89.5)	777 (93.5, 91.8-95.2)	998 (96.3, 95.2-97.5)	

*$p \leq 0.001$
†Answers of "don't know", "prefer not to say" and "not applicable, I wouldn't do this anyway" were coded as missing, therefore total *n*s are substantially lower (general population, n=2490; Savanta GBMSM, n=216; Grindr, n=790; Meta, n=991).

In the general population, intention to self-isolate was associated with: less financial hardship, being more worried about mpox, perceiving a bigger threat of mpox to people in the UK, greater perceived susceptibility and severity of mpox, and greater perceived social norms for self-isolation (Tables 3 and 4). Not intending to self-isolate was associated with agreeing that if you had mpox symptoms, you would not want to tell anyone as you did not want to self-isolate, believing the risks of mpox were being exaggerated, and that if you had to self-isolate due to mpox it would have a negative impact on your work (Table 4). In the Grindr sample, self-isolation intention was associated with greater perceived social norms (Table 4). Not intending to self-isolate was associated with agreeing that if you had mpox symptoms, you would not want to tell anyone as you did not want to self-isolate, and that if you had to self-isolate due to mpox it would have a negative impact on your work (Table 4).

Table 3. Associations between intending to self-isolate and socio-demographic characteristics and motivational message, by sample. A higher score indicates greater intention to self-isolate. Variables were entered into the linear regression model in blocks (block 1: socio-demographic variables and motivational message, block 2: psychological factors, block 3: isolation-specific beliefs). Results for block 3, using pooled estimates are reported.

Participant characteristics	Level	General population		Grindr	
		B (95% CI)	*p*-value	*B* (95% CI)	*p*-value
Gender	Male (including trans man)	Ref	-	-	-
	Female (including trans woman)	0.15 (0.01 to 0.30)	0.03	-	-
Sexual orientation	Straight or heterosexual	Ref	-	-	-
	Gay, lesbian, bisexual, or queer	0.06 (-0.21 to 0.33)	0.67	-	-
Age	Range 18 to 98 years	0.01 (0.00 to 0.01)	0.06	0.00 (-0.01 to 0.02)	0.66

For peer review only - http://bmjopen.bmj.com/site/about/guidelines.xhtml

	Quadratic term, (age – mean)^2	0.0002 (-0.0001 to 0.0005)	0.17	0.001 (0.000 to 0.002)	0.02
Region	Midlands (East and West)	Ref	-	Ref	-
	South (East, West, East of England)	-0.19 (-0.39 to 0.01)	0.07	0.02 (-0.51 to 0.55)	0.95
	North (East, West, Yorkshire and the Humber)	-0.07 (-0.28 to 0.13)	0.49	0.06 (-0.53 to 0.64)	0.85
	London	-0.11 (-0.39 to 0.16)	0.42	-0.01 (-0.59 to 0.57)	0.98
	Devolved nations (Scotland, Wales, and Northern Ireland)	-0.04 (-0.30 to 0.22)	0.78	0.48 (-0.20 to 1.16)	0.17
Dependent child in household	No	Ref	-	Ref	-
	Yes	-0.15 (-0.32 to 0.03)	0.10	-0.42 (-1.13 to 0.29)	0.25
Employment status	Not working	Ref	-	Ref	-
	Working	0.09 (-0.12 to 0.31)	0.39	-0.13 (-0.61 to 0.34)	0.58
Frontline health or social care worker	No	Ref	-	Ref	-
	Yes	0.08 (-0.14 to 0.30)	0.47	0.68 (0.24 to 1.13)	0.003
Need to leave home for work	Do not need to leave home for work	Ref	-	Ref	-
	Need to leave home for work	-0.26 (-0.45 to -0.07)	0.007	0.14 (-0.22 to 0.51)	0.44
Education	GCSE/vocational/A-level/No formal qualifications	Ref	-	Ref	-
	Degree or higher (Bachelors, Masters, PhD)	0.02 (-0.13 to 0.18)	0.77	-0.16 (-0.48 to 0.16)	0.31
Ethnicity	White British	Ref	-	Ref	-
	White other	-0.23 (-0.59 to 0.13)	0.21	-0.33 (-0.75 to 0.09)	0.12
	Black, Asian, other minoritized ethnicity	0.11 (-0.15 to 0.37)	0.40	-0.03 (-0.52 to 0.46)	0.91
Marital status	Not partnered	Ref	-	Ref	-
	Partnered	0.15 (-0.04 to 0.34)	0.12	0.13 (-0.24 to 0.49)	0.50
Live alone	Live with someone else	Ref	-	Ref	-
	Live alone	0.06 (-0.15 to 0.28)	0.56	-0.07 (-0.40 to 0.26)	0.69
Own chronic illness	None	Ref	-	Ref	-
	Present	0.21 (0.05 to 0.38)	0.009	0.10 (-0.24 to 0.44)	0.56
Ever taken PrEP for HIV	No	-	-	Ref	-
	Yes	-	-	-0.11 (-0.43 to 0.22)	0.51
Vaccinated for smallpox in 2022	Not vaccinated	-	-	Ref	-
	Vaccinated	-	-	0.03 (-0.32 to 0.39)	0.86
Index of multiple deprivation	Deciles (1st = most deprived, 10th = least deprived)	-0.01 (-0.04 to 0.02)	0.48	0.02 (-0.05 to 0.08)	0.66
Socio-economic grade	ABC1	Ref	-	Ref	-
	C2DE	-0.09 (-0.24 to 0.06)	0.24	-0.03 (-0.46 to 0.39)	0.88
Financial hardship	4 (lowest hardship) to 13 (most hardship)	-0.08 (-0.12 to -0.05)	<0.001*	-0.02 (-0.11 to 0.07)	0.70
Total number of sexual partners (male and female) in last three weeks	0	Ref	-	-	-
	1	-0.04 (-0.23 to 0.14)	0.65	-	-
	2 to 4	-0.25 (-0.59 to 0.10)	0.16	-	-
	5 or more	-0.09 (-0.69 to 0.52)	0.77	-	-
	Prefer not to say	-0.22 (-0.42 to -0.02)	0.03	-	-
Number of male sexual partners in last three weeks	0	-	-	Ref	-
	1	-	-	-0.50 (-0.92 to -0.08)	0.02
	2 to 4	-	-	-0.09 (-0.47 to 0.30)	0.65
	5 to 9	-	-	-0.48 (-1.00 to 0.03)	0.07
	10 or more	-	-	-0.32 (-0.96 to 0.33)	0.34
	Prefer not to say	-	-	0.83 (0.12 to 1.53)	0.02
Motivational message	Perceived risk of illness and necessity and efficacy of the response	-0.06 (-0.25 to 0.12)	0.50	-	-
	Perceived risk of illness and benefits of the response	-0.09 (-0.28 to 0.09)	0.33	-	-

For peer review only - http://bmjopen.bmj.com/site/about/guidelines.xhtml

	Perceived risk of illness and low perceived costs of response	-0.19 (-0.38 to 0.00)	0.05	-	-
	Control	Ref	-	-	-
Motivational message	All motivational components	-	-	-0.10 (-0.37 to 0.18)	0.48
	Control	-	-	Ref	-

**p*≤0.001

Table 4. Associations between intending to self-isolate and psychological factors and isolation-specific beliefs, by sample. A higher score indicates greater intention to self-isolate. Variables were entered into the linear regression model in blocks (block 1: socio-demographic variables and motivational message, block 2: psychological factors, block 3: isolation-specific beliefs). Results for block 3, using pooled estimates are reported.

Factor	Level	General population *B* (95% CI)	General population *p*-value	Grindr *B* (95% CI)	Grindr *p*-value
Amount heard about mpox	I have not seen or heard anything (1) to I have seen or heard a lot (3)	0.09 (-0.07 to 0.24)	0.26	-0.09 (-0.41 to 0.23)	0.57
Worry about mpox	Not at all worried (1) to extremely worried (4)	0.26 (0.13 to 0.39)	<0.001*	0.41 (0.12 to 0.71)	0.006
Perceived risk of mpox to oneself	No risk at all (1) to very high risk (5)	-0.05 (-0.17 to 0.06)	0.39	-0.25 (-0.46 to -0.04)	0.02
Perceived risk of mpox to people in UK	No risk at all (1) to very high risk (5)	0.19 (0.07 to 0.31)	0.001*	0.030 (-0.20 to 0.25)	0.82
Perceived susceptibility and severity	Lowest (1) to highest (5)	0.03 (0.19 to 0.42)	<0.001*	0.12 (-0.13 to 0.37)	0.36
I am already immune to mpox	Strongly disagree, disagree, neither agree nor disagree, don't know	Ref	-	Ref	-
	Strongly agree and agree	0.27 (0.03 to 0.52)	0.03	-0.12 (-0.61 to 0.36)	0.62
People who catch mpox usually make a full recovery, even if they do not receive any treatment	Strongly disagree (1) to strongly agree (5)	-0.05 (-0.14 to 0.03)	0.23	-0.26 (-0.43 to -0.08)	0.004
My personal behaviour has an impact on how mpox spreads	Strongly disagree (1) to strongly agree (5)	0.09 (0.03 to 0.15)	0.004	0.08 (-0.06 to 0.22)	0.26
My life has been negatively affected by changes made in response to the mpox outbreak	Strongly disagree (1) to strongly agree (5)	-0.01 (-0.09 to 0.07)	0.89	-0.03 (-0.17 to 0.11)	0.70
The risks of mpox are being exaggerated	Strongly disagree (1) to strongly agree (5)	-0.19 (-0.27 to -0.11)	<0.001*	-0.07 (-0.24 to 0.09)	0.39
Mpox is only a risk to men who are gay, bisexual or have sex with men	Strongly disagree (1) to strongly agree (5)	-0.02 (-0.08 to 0.05)	0.58	-0.17 (-0.32 to -0.02)	0.03
Perceived knowledge	Lowest (0) to highest (3)	-0.01 (-0.08 to 0.07)	0.88	-0.18 (-0.37 to 0.02)	0.08
Knowledge of mpox symptoms	Identified no symptoms (0) to identified four symptoms (4)	0.08 (0.03 to 0.14)	0.003	0.06 (-0.06 to 0.19)	0.31
Knowledge of mpox transmission	Lowest (0) to highest (6)	0.02 (-0.03 to 0.07)	0.42	0.04 (-0.10 to 0.17)	0.59
If I had mpox symptoms, I wouldn't want to tell anyone as I don't want to self-isolate	Strongly disagree (1) to strongly agree (5)	-0.66 (-0.73 to -0.60)	<0.001*	-1.01 (-1.15 to -0.87)	<0.001*
Most people would self-isolate if they were told to	Strongly disagree (1) to strongly agree (5)	0.34 (0.28 to 0.41)	<0.001*	0.41 (0.28 to 0.54)	<0.001*
If I had to self-isolate because I had tested positive for mpox...it would have a negative impact on my work	Strongly disagree (1) to strongly agree (5)	-0.15 (-0.21 to -0.09)	<0.001*	-0.34 (-0.46 to -0.21)	<0.001*

**p*≤0.001

For peer review only - http://bmjopen.bmj.com/site/about/guidelines.xhtml

Help seeking

Approximately half of participants in the general population, Savanta GBMSM, and Grindr samples indicated that they would seek help immediately (49.2% to 53.3%; Table 2). Intention to seek help immediately was higher in the Meta sample (62.3%, 95% CI 59.3 to 65.2, n=645).

In the general population, intention to seek help immediately was associated with being older (aOR 1.012, 95% CI 1.006 to 1.019, p<0.001), disagreeing that the risks of mpox are being exaggerated (aOR 0.83, 95% CI 0.76 to 0.91, p<0.001), and being willing to contact a sexual health clinic if you thought you had mpox symptoms or had been in contact with a case (aOR 1.25, 95% CI 1.16 to 1.34, p<0.001; supplementary materials 5). In the Grindr sample, intention to seek help immediately was associated with being willing to contact a sexual health clinic if you thought you had mpox symptoms or had been in contact with a case (aOR 1.57, 95% CI 1.29 to 1.91, p<0.001; supplementary materials 5).

Sexual contact behaviour when symptomatic

In the general population and Savanta GBMSM, 77.2% (95% CI 75.6% to 78.9%, n=1923) and 79.2% (73.7% to 84.6%, n=171) intended to completely stop sexual contact if they were to develop an unexplained rash with blisters and learn that they had come into contact with a mpox case (Table 2). Rates of intending to completely stop sexual contact were significantly higher in Grindr (91.3%, 95% CI 89.3% to 93.2%, n=721) and Meta samples (93.0%, 95% CI 91.4% to 94.6%, n=922). The number of days that participants would wait before resuming sexual contact from the start of their symptoms was also higher in Grindr and Meta samples than in general population and Savanta GBMSM samples (F(3,3900)=29.0, p<0.001; general population: n=2021, M=15.7, SD=15.3; Savanta GBMSM: n=190, M=16.2, SD=14.4; Grindr: n=746, M=20.4, SD=15.8; Meta: n=947, M=20.0, SD=12.3).

In the general population, intending to completely stop any sexual contact if symptomatic was associated with being female, older, less financial hardship, and being more knowledgeable about mpox transmission (Tables 5 and 6). Not intending to completely stop sexual behaviour was associated with preferring not to say how many recent sexual partners you had had, thinking that you were already immune to mpox, that your life had been negatively affected by changes made in response to the mpox outbreak, and that the risks of

For peer review only - http://bmjopen.bmj.com/site/about/guidelines.xhtml

mpox were being exaggerated (Tables 5 and 6). No associations reached significance (Bonferroni corrected) in the Grindr sample.

Table 5. Associations between intending to completely stop any sexual contact and socio-demographic characteristics and motivational message, by sample. Variables were entered into the logistic regression model in blocks (block 1: socio-demographic variables and motivational message, block 2: psychological factors). Results for block 2, using pooled estimates are reported.

Participant characteristics	Level	General population aOR (95% CI)	General population *p*-value	Grindr aOR (95% CI)	Grindr *p*-value
Gender	Male (including trans man)	Ref	-	-	-
	Female (including trans woman)	1.90 (1.48 to 2.45)	<0.001*	-	-
Sexual orientation	Straight or heterosexual	Ref	-	-	-
	Gay, lesbian, bisexual, or queer	0.91 (0.58 to 1.41)	0.67	-	-
Age	Range 18 to 98 years	1.04 (1.03 to 1.05)	<0.001*	1.01 (0.98 to 1.04)	0.53
	Quadratic term, (age – mean)^2	1.000 (0.999 to 1.001)	0.87	1.000 (0.998 to 1.001)	0.71
Region	Midlands (East and West)	Ref	-	Ref	-
	South (East, West, East of England)	1.08 (0.76 to 1.54)	0.67	1.18 (0.38 to 3.66)	0.77
	North (East, West, Yorkshire and the Humber)	1.14 (0.79 to 1.66)	0.49	1.48 (0.35 to 6.21)	0.58
	London	1.17 (0.73 to 1.86)	0.52	0.94 (0.27 to 3.25)	0.92
	Devolved nations (Scotland, Wales, and Northern Ireland)	1.26 (0.79 to 2.01)	0.33	1.26 (0.31 to 5.15)	0.74
Dependent child in household	No	Ref	-	Ref	-
	Yes	0.97 (0.72 to 1.29)	0.82	0.37 (0.11 to 1.28)	0.12
Employment status	Not working	Ref	-	Ref	-
	Working	1.22 (0.90 to 1.66)	0.20	0.92 (0.39 to 2.18)	0.85
Frontline health or social care worker	No	Ref	-	Ref	-
	Yes	0.97 (0.67 to 1.39)	0.86	1.77 (0.67 to 4.65)	0.25
Education	GCSE/vocational/A-level/No formal qualifications	Ref	-	Ref	-
	Degree or higher (Bachelors, Masters, PhD)	0.96 (0.73 to 1.27)	0.79	1.87 (0.97 to 3.59)	0.06
Ethnicity	White British	Ref	-	Ref	-
	White other	0.47 (0.26 to 0.84)	0.01	0.84 (0.35 to 2.06)	0.71
	Black, Asian, other minoritized ethnicity	0.85 (0.56 to 1.27)	0.42	0.36 (0.15 to 0.85)	0.02
Marital status	Not partnered	Ref	-	Ref	-
	Partnered	0.90 (0.65 to 1.24)	0.50	1.11 (0.51 to 2.41)	0.79
Live alone	Live with someone else	Ref	-	Ref	-
	Live alone	1.16 (0.77 to 1.73)	0.48	1.12 (0.56 to 2.23)	0.75
Own chronic illness	None	Ref	-	Ref	-
	Present	0.97 (0.72 to 1.31)	0.85	1.10 (0.53 to 2.27)	0.80
Ever taken PrEP for HIV	No	-	-	Ref	-
	Yes	-	-	1.36 (0.69 to 2.71)	0.37
Vaccinated for smallpox in 2022	Not vaccinated	-	-	Ref	-
	Vaccinated	-	-	0.71 (0.33 to 1.50)	0.37
Index of multiple deprivation	Deciles (1st = most deprived, 10th = least deprived)	1.00 (0.96 to 1.06)	0.85	1.01 (0.86 to 1.19)	0.88
Socio-economic grade	ABC1	Ref	-	Ref	-
	C2DE	1.13 (0.86 to 1.49)	0.39	4.15 (1.45 to 11.85)	0.008
Financial hardship	4 (lowest hardship) to 13 (most hardship)	0.85 (0.80 to 0.90)	<0.001*	0.89 (0.74 to 1.07)	0.21

For peer review only - http://bmjopen.bmj.com/site/about/guidelines.xhtml

Total number of sexual partners (male and female) in last three weeks	0	Ref	-	-	-
	1	0.83 (0.59 to 1.18)	0.30	-	-
	2 to 4	0.68 (0.39 to 1.18)	0.17	-	-
	5 or more	0.37 (0.14 to 0.96)	0.04	-	-
	Prefer not to say	0.45 (0.32 to 0.64)	<0.001*	-	-
Number of male sexual partners in last three weeks	0	-	-	Ref	-
	1	-	-	0.52 (0.18 to 1.46)	0.21
	2 to 4	-	-	0.38 (0.14 to 0.98)	0.05
	5 to 9	-	-	0.21 (0.07 to 0.65)	0.007
	10 or more	-	-	0.20 (0.06 to 0.72)	0.01
	Prefer not to say	-	-	0.30 (0.08 to 1.11)	0.07
Motivational message	Perceived risk of illness and necessity and efficacy of the response	1.37 (0.97 to 1.93)	0.08	-	-
	Perceived risk of illness and benefits of the response	1.07 (0.76 to 1.49)	0.70	-	-
	Perceived risk of illness and low perceived costs of response	1.10 (0.79 to 1.55)	0.57	-	-
	Control	Ref	-	-	-
Motivational message	All motivational components	-	-	1.43 (0.80 to 2.54)	0.23
	Control	-	-	Ref	-

*$p \leq 0.001$

Table 6. Associations between intending to completely stop any sexual contact and psychological and contextual factors, by sample. Variables were entered into the logistic regression model in blocks (block 1: socio-demographic variables and motivational message, block 2: psychological factors). Results for block 2, using pooled estimates are reported.

Factor	Level	General population aOR (95% CI)	*p*-value	Grindr aOR (95% CI)	*p*-value
Amount heard about mpox	I have not seen or heard anything (1) to I have seen or heard a lot (3)	1.42 (1.08 to 1.86)	0.01	1.38 (0.70 to 2.72)	0.35
Worry about mpox	Not at all worried (1) to extremely worried (4)	1.11 (0.88 to 1.40)	0.36	1.51 (0.82 to 2.77)	0.19
Perceived risk of mpox to oneself	No risk at all (1) to very high risk (5)	0.83 (0.68 to 1.02)	0.08	0.93 (0.60 to 1.44)	0.75
Perceived risk of mpox to people in UK	No risk at all (1) to very high risk (5)	1.10 (0.90 to 1.34)	0.35	0.99 (0.64 to 1.53)	0.95
Perceived susceptibility and severity	Lowest (1) to highest (5)	1.27 (1.03 to 1.56)	0.03	1.18 (0.72 to 1.94)	0.51
I am already immune to mpox	Strongly disagree, disagree, neither agree nor disagree, don't know	Ref	-	Ref	-
	Strongly agree and agree	0.37 (0.26 to 0.53)	<0.001*	0.78 (0.32 to 1.90)	0.59
People who catch mpox usually make a full recovery, even if they do not receive any treatment	Strongly disagree (1) to strongly agree (5)	0.87 (0.74 to 1.01)	0.08	0.66 (0.45 to 0.97)	0.04
My personal behaviour has an impact on how mpox spreads	Strongly disagree (1) to strongly agree (5)	1.03 (0.92 to 1.16)	0.57	1.42 (1.08 to 1.87)	0.01
My life has been negatively affected by changes made in response to the mpox outbreak	Strongly disagree (1) to strongly agree (5)	0.70 (0.62 to 0.80)	<0.001*	0.73 (0.55 to 0.97)	0.03
The risks of mpox are being exaggerated	Strongly disagree (1) to strongly agree (5)	0.77 (0.67 to 0.88)	<0.001*	0.81 (0.58 to 1.11)	0.18

For peer review only

For peer review only - http://bmjopen.bmj.com/site/about/guidelines.xhtml

mpox is only a risk to men who are gay, bisexual or have sex with men	Strongly disagree (1) to strongly agree (5)	0.87 (0.77 to 0.97)	0.02	0.77 (0.58 to 1.03)	0.08
Perceived knowledge	Lowest (0) to highest (3)	1.00 (0.88 to 1.15)	0.94	0.86 (0.58 to 1.28)	0.46
Knowledge of mpox symptoms	Identified no symptoms (0) to identified four symptoms (4)	1.14 (1.03 to 1.26)	0.01	1.29 (1.00 to 1.66)	0.05
Knowledge of mpox transmission	Lowest (0) to highest (6)	1.18 (1.08 to 1.29)	<0.001*	1.31 (1.00 to 1.73)	0.05

*$p \leq 0.001$

Sharing details of contacts

There was no difference in intention to share details of all sexual partners in the last seven days between samples (Table 2), with 75.3% to 80.2% saying that they probably or definitely would.

In the general population sample, intention to share details of every sexual contact in the last seven days was associated with being female, older, less financial hardship, higher perceived susceptibility to and severity of mpox, and higher knowledge about modes of mpox transmission (Tables 7 and 8). Not intending to share details of every recent sexual contact was associated with preferring not to say how many recent sexual partners you had had, thinking that your life had been negatively affected by changes made in response to the mpox outbreak, and thinking that the risks of mpox had been exaggerated (Tables 7 and 8). No associations reached our threshold for significance in the Grindr sample.

Table 7. Associations between intending to share details of every sexual contact in the last seven days and socio-demographic characteristics and motivational message, by sample. A higher score indicates greater intention to share details. Variables were entered into the linear regression model in blocks (block 1: socio-demographic variables and motivational message, block 2: psychological factors). Results for block 2, using pooled estimates are reported.

Participant characteristics	Level	General population		Grindr	
		B (95% CI)	*p*-value	*B* (95% CI)	*p*-value
Gender	Male (including trans man)	Ref	-	-	-
	Female (including trans woman)	0.16 (0.07 to 0.24)	<0.001*	-	-
Sexual orientation	Straight or heterosexual	Ref	-	-	-
	Gay, lesbian, bisexual, or queer	0.12 (-0.05 to 0.28)	0.16	-	-
Age	Range 18 to 98 years	0.008 (0.005 to 0.011)	<0.001*	0.003 (-0.005 to 0.011)	0.46
	Quadratic term, $(\text{age} - \text{mean})^2$	-0.00017 (-0.00034 to -0.00001)	0.04	0.0003 (-0.0002 to 0.0008)	0.19
Region	Midlands (East and West)	Ref	-	Ref	-
	South (East, West, East of England)	-0.04 (-0.16 to 0.08)	0.50	0.08 (-0.23 to 0.39)	0.60

For peer review only - http://bmjopen.bmj.com/site/about/guidelines.xhtml

	North (East, West, Yorkshire and the Humber)	-0.07 (-0.19 to 0.06)	0.29	0.31 (-0.05 to 0.68)	0.09
	London	0.05 (-0.11 to 0.22)	0.53	0.12 (-0.25 to 0.49)	0.52
	Devolved nations (Scotland, Wales, and Northern Ireland)	0.09 (-0.06 to 0.25)	0.24	0.26 (-0.21 to 0.73)	0.27
Dependent child in household	No	Ref	-	Ref	-
	Yes	-0.12 (-0.22 to -0.01)	0.03	-0.22 (-0.64 to 0.20)	0.30
Employment status	Not working	Ref	-	Ref	-
	Working	-0.10 (-0.20 to 0.00)	0.05	-0.01 (-0.26 to 0.23)	0.90
Frontline health or social care worker	No	Ref	-	Ref	-
	Yes	-0.04 (-0.17 to 0.09)	0.57	0.08 (-0.19 to 0.34)	0.57
Education	GCSE/vocational/A-level/No formal qualifications	Ref	-	Ref	-
	Degree or higher (Bachelors, Masters, PhD)	0.00 (-0.09 to 0.09)	0.97	0.13 (-0.06 to 0.32)	0.19
Ethnicity	White British	Ref	-	Ref	-
	White other	-0.29 (-0.51 to -0.08)	0.008	0.00 (-0.25 to 0.25)	0.98
	Black, Asian, other minoritized ethnicity	-0.17 (-0.32 to -0.01)	0.03	0.01 (-0.29 to 0.30)	0.97
Marital status	Not partnered	Ref	-	Ref	-
	Partnered	0.03 (-0.08 to 0.15)	0.55	0.10 (-0.11 to 0.32)	0.35
Live alone	Live with someone else	Ref	-	Ref	-
	Live alone	-0.04 (-0.17 to 0.08)	0.50	0.21 (0.01 to 0.40)	0.04
Own chronic illness	None	Ref	-	Ref	-
	Present	-0.04 (-0.14 to 0.05)	0.40	0.31 (0.11 to 0.51)	0.003
Ever taken PrEP for HIV	No	-	-	Ref	-
	Yes	-	-	0.09 (-0.10 to 0.28)	0.35
Vaccinated for smallpox in 2022	Not vaccinated	-	-	Ref	-
	Vaccinated	-	-	-0.07 (-0.28 to 0.14)	0.50
Index of multiple deprivation	Deciles (1st = most deprived, 10th = least deprived)	0.00 (-0.02 to 0.02)	0.87	0.03 (-0.02 to 0.08)	0.19
Socio-economic grade	ABC1	Ref	-	Ref	-
	C2DE	-0.12 (-0.21 to -0.03)	0.01	0.04 (-0.22 to 0.29)	0.78
Financial hardship	4 (lowest hardship) to 13 (most hardship)	-0.05 (-0.07 to -0.03)	<0.001*	-0.02 (-0.07 to 0.04)	0.53
Total number of sexual partners (male and female) in last three weeks	0	Ref	-	-	-
	1	0.06 (-0.05 to 0.17)	0.28	-	-
	2 to 4	0.07 (-0.14 to 0.27)	0.53	-	-
	5 or more	-0.09 (-0.45 to 0.28)	0.64	-	-
	Prefer not to say	-0.37 (-0.49 to -0.24)	<0.001*	-	-
Number of male sexual partners in last three weeks	0	-	-	Ref	-
	1	-	-	-0.02 (-0.27 to 0.22)	0.84
	2 to 4	-	-	-0.02 (-0.24 to 0.21)	0.90
	5 to 9	-	-	-0.08 (-0.38 to 0.23)	0.62
	10 or more	-	-	-0.58 (-0.96 to -0.20)	0.003
	Prefer not to say	-	-	-0.09 (-0.52 to 0.34)	0.67
Motivational message	Perceived risk of illness and necessity and efficacy of the response	-0.03 (-0.15 to 0.08)	0.55	-	-
	Perceived risk of illness and benefits of the response	0.03 (-0.08 to 0.14)	0.59	-	-
	Perceived risk of illness and low perceived costs of response	-0.01 (-0.12 to 0.10)	0.86	-	-
	Control	Ref	-	-	-
Motivational message	All motivational components	-	-	-0.06 (-0.23 to 0.10)	0.45
	Control	-	-	Ref	-

*$p \leq 0.001$

For peer review only - http://bmjopen.bmj.com/site/about/guidelines.xhtml

Table 8. Associations between intending to share details of every sexual contact in the last seven days and psychological and contextual factors, by sample. A higher score indicates greater intention to share details. Variables were entered into the linear regression model in blocks (block 1: socio-demographic variables and motivational message, block 2: psychological factors). Results for block 2, using pooled estimates are reported.

Factor	Level	General population		Grindr	
		B (95% CI)	*p*-value	*B* (95% CI)	*p*-value
Amount heard about mpox	I have not seen or heard anything (1) to I have seen or heard a lot (3)	-0.04 (-0.13 to 0.05)	0.43	0.01 (-0.18 to 0.20)	0.92
Worry about mpox	Not at all worried (1) to extremely worried (4)	0.08 (0.00 to 0.16)	0.06	0.19 (0.01 to 0.36)	0.03
Perceived risk of mpox to oneself	No risk at all (1) to very high risk (5)	-0.04 (-0.11 to 0.03)	0.27	-0.06 (-0.18 to 0.06)	0.34
Perceived risk of mpox to people in UK	No risk at all (1) to very high risk (5)	0.07 (0.00 to 0.14)	0.06	-0.01 (-0.14 to 0.12)	0.85
Perceived susceptibility and severity	Lowest (1) to highest (5)	0.12 (0.05 to 0.19)	<0.001*	-0.09 (-0.24 to 0.06)	0.24
I am already immune to mpox	Strongly disagree, disagree, neither agree nor disagree, don't know	Ref	-	Ref	-
	Strongly agree and agree	-0.18 (-0.32 to -0.04)	0.01	-0.09 (-0.37 to 0.20)	0.55
People who catch mpox usually make a full recovery, even if they do not receive any treatment	Strongly disagree (1) to strongly agree (5)	-0.01 (-0.06 to 0.04)	0.71	-0.06 (-0.17 to 0.04)	0.23
My personal behaviour has an impact on how mpox spreads	Strongly disagree (1) to strongly agree (5)	0.04 (0.01 to 0.08)	0.03	0.02 (-0.06 to 0.10)	0.64
My life has been negatively affected by changes made in response to the mpox outbreak	Strongly disagree (1) to strongly agree (5)	-0.10 (-0.15 to -0.06)	<0.001*	-0.03 (-0.11 to 0.06)	0.56
The risks of mpox are being exaggerated	Strongly disagree (1) to strongly agree (5)	-0.11 (-0.15 to -0.06)	<0.001*	-0.15 (-0.25 to -0.05)	0.004
Mpox is only a risk to men who are gay, bisexual or have sex with men	Strongly disagree (1) to strongly agree (5)	-0.02 (-0.06 to 0.02)	0.29	-0.07 (-0.16 to 0.01)	0.10
Perceived knowledge	Lowest (0) to highest (3)	0.05 (0.00 to 0.09)	0.04	0.02 (-0.10 to 0.13)	0.78
Knowledge of mpox symptoms	Identified no symptoms (0) to identified four symptoms (4)	0.05 (0.02 to 0.08)	0.004	0.05 (-0.02 to 0.13)	0.14
Knowledge of mpox transmission	Lowest (0) to highest (6)	0.05 (0.02 to 0.08)	<0.001*	0.05 (-0.03 to 0.13)	0.22

*$p \leq 0.001$

Vaccination

Few people had been vaccinated for smallpox in 2022 in the general population sample (5.3%, 95% CI 4.5% to 6.1%, n=162; Table 2). This was significantly higher in GBMSM samples (10.5% to 40.7%). In a measure of actual and intended vaccination, 96.3% (95% CI 95.2% to 97.5%, n=998) of the Meta sample and 93.5% (95% CI 91.8% to 95.2%, n=777) of the Grindr sample were vaccinated or intended to be vaccinated for smallpox if advised. Rates were significantly lower in the general population and Savanta GBMSM samples.

For peer review only - http://bmjopen.bmj.com/site/about/guidelines.xhtml

In the general population, being vaccinated for smallpox in 2022 or intending to be vaccinated if offered a vaccine was associated with being older (aOR 1.015, 95% CI 1.007 to 1.023, $p<0.001$), more worried about mpox (1.49, 95% CI 1.24 to 1.80, $p<0.001$), and perceiving a higher susceptibility to and severity of mpox (1.31, 95% CI 1.11 to 1.55, $p<0.001$; supplementary materials 6). Not intending to be vaccinated was associated with needing to leave home for work (aOR 0.56, 95% CI 0.43 to 0.73, $p<0.001$) and agreeing that the risks of mpox were being exaggerated (aOR 0.76, 95% CI 0.68 to 0.85, $p<0.001$; supplementary materials 6).

In the Grindr sample, vaccination uptake was associated with ever having taken PrEP for HIV (aOR 8.95, 95% CI 5.61 to 14.28, $p<0.001$) and agreeing that you are already immune to mpox (aOR 8.83, 95% CI 4.52 to 17.22, $p<0.001$; supplementary materials 6). When including vaccination intention, being vaccinated for smallpox in 2022 or intending to be vaccinated if offered a vaccine was associated with agreeing that if you got a smallpox vaccine, you would be protected against mpox (aOR 3.25, 95% CI 1.97 to 5.35, $p<0.001$; supplementary materials 6).

DISCUSSION

We investigated mpox attitudes, beliefs, and intended behaviours in a general population sample and three GBMSM samples. Samples differed by socio-demographic characteristics. This was reflected in intended behaviours, with GBMSM recruited from Grindr or Meta being more likely to intend to seek help immediately for mpox symptoms, completely stop sexual contact when symptomatic, and be vaccinated for smallpox if advised. High rates of smallpox vaccination in these samples perhaps reflects that they were mostly educated and working, and thus more likely to be health literate and engaged with services. This was also reflected in knowledge about mpox, with Grindr and Meta samples being more likely to correctly identify mpox transmission modes and the symptoms of mpox. Within the general population, rates of "don't know" answers were high, with 18.4% selecting "don't know" when asked what the main symptoms of mpox were, and 18.7% to 24.2% selecting "don't know" for transmission modes (except for having sex with someone who has mpox, where 13.9% selected "don't know"). This is similar to other surveys of the general population, where 24% of people were not sure if mpox usually spreads by close contact with an infected person.(41)

For peer review only - http://bmjopen.bmj.com/site/about/guidelines.xhtml

There were no effects of motivational messaging on our behavioural outcomes, except for in the Meta sample for intention to self-isolate, share details of all recent sexual contacts, and be vaccinated for smallpox if advised. For these outcomes, intention to engage with protective behaviours was higher in the control group. In practice, however, intentions were very similar (means changed by 0.1 to 0.4) and this is unlikely to represent a meaningful difference. Another study investigating gonorrhoea reinfection in young adults has also found that those in the control group (who received monthly texts reminding them to update their contact details) were less likely to be reinfected than those in the intervention group (who received a series of texts that were educational and used behaviour change techniques).(42) These messages were specifically designed to decrease stigma, and partner numbers were higher in the intervention arm. A systematic review of intervention communications during the COVID-19 pandemic found that messages about the personal and collective benefits of vaccination had mixed effects on vaccination intention, with some suggestions that this approach may be more effective in more strongly hesitant individuals.(28)

Generally, higher intentions were seen in the Grindr and Meta samples, except for self-isolation. However, rates should be interpreted with caution, as intended behaviour does not always translate to enacted behaviour.(43) This was shown to be the case for intended and actual engagement with the UK contact tracing programme during the COVID-19 pandemic.(11) Rates of having already been vaccinated for smallpox in 2022 were especially high (32% Grindr, 41% Meta), again suggesting our sample may have been particularly interested in the topic of the survey, especially given issues with access to vaccination.(44) In the UK, the public health agency (UK Health Security Agency) has been working in conjunction with community-based organisations and charities to raise awareness of mpox and how to prevent the transmission in GBMSM as the population most affected. Higher rates of knowledge and behavioural intentions suggest that this targeted messaging has been effective in increasing knowledge and driving protective behaviours. Evidence suggests that while one-off sexual encounters may make up only a minority of sexual interactions, they could account for a large proportion of mpox transmission.(45, 46) Furthermore, if encounters are anonymous, this will affect contact tracing efforts. Therefore, very high rates of intending to completely stop sexual contact with others if symptomatic (91% to 93% in Grindr and Meta samples respectively) are encouraging.

It is notable that 19% (Grindr, Meta) to 30% of people (general population, Savanta GBMSM) agreed that is it best to avoid physical contact with GBMSM because of the mpox

For peer review only - http://bmjopen.bmj.com/site/about/guidelines.xhtml

outbreak, perhaps also reflecting a change in behaviour in this population. Recent decreases in some sexually transmitted infections in GBMSM, and the decline in mpox transmission in England, suggest this may be the case.(6)

Within the general population, women, people who were older, and those with lower financial hardship were more likely to intend to carry out protective behaviours. This pattern was also widely seen during the COVID-19 pandemic (11, 47, 48) and previous outbreaks.(49) This highlights the importance of considering health equity issues to ensure effective outbreak control. Offering financial support for protective behaviours, especially those that may affect people's ability to earn an income such as self-isolation, is likely to increase engagement, especially for those in lower income settings. Few socio-demographic characteristics were associated with outcomes in the Grindr sample. This could be because the Grindr sample differed from the general population sample on key characteristics (more educated, higher socio-economic grade, less financial hardship), or be a function of the way analyses were conducted with all variables being entered into a single regression model. The Grindr sample, being smaller, also had about half the statistical power of the general population sample.

Intention to engage in protective behaviours in the general population was also associated with psychological factors such as greater worry about mpox, perceived risk of mpox to others (but there was little evidence of an association with perceived risk to oneself), perceived susceptibility and severity of mpox, and greater knowledge about transmission. This pattern of results was also seen during the COVID-19 and aH1N1 influenza pandemics.(12, 47, 50-52) COVID-19 vaccination intention was also associated with perceived risk to others, but not to oneself.(33) Few associations were significant in the Grindr sample. In addition to the decreased statistical power, this may also reflect the different context and experience that participants in this group had. While for our general population sample the questions about mpox risk were somewhat hypothetical, the Grindr sample were GBMSM and thus more likely to be impacted by mpox (as illustrated by the high vaccination rates). Factors relating to their personal experience of the outbreak may have superseeded any effect of the psychological variables that we assessed. Taken together, these results suggest that clear communications about the level of risk of infection may encourage people to enact protective behaviours when appropriate.

Agreeing that the risks of mpox were being exaggerated was associated with lower intention to engage with protective behaviours. This was also the case in previous pandemics.(47, 53)

For peer review only - http://bmjopen.bmj.com/site/about/guidelines.xhtml

Other psychological factors associated with decreased intention to engage with protective behaviours were believing that you were already immune to mpox and that your life had been negatively affected by changes made in response to mpox. Attending an important event was one of the reasons given for breaking self-isolation during the aH1N1 pandemic.(54) The peak of the mpox outbreak in the UK occurred in July 2022, a period coinciding with the summer holiday and festival season, including Pride events. It is important to be conscious of asking people to self-isolate or quarantine over periods encompassing key events that may be happening.

Behaviour-specific beliefs were also associated with intentions. For self-isolation, greater intention was associated with greater social norms (thinking that others would also self-isolate). Greater social norms were associated with fewer outings during lockdown (14) and increased vaccination uptake and intention (in oneself and one's child) (15, 55) during the COVID-19 pandemic. Social norms were also associated with enacting protective behaviours, including self-isolation, during previous outbreaks.(49, 56) Lower self-isolation intention was associated with thinking that self-isolation would have a negative impact on your work. In this study, as in previous research,(11) the main reasons for not being able to self-isolate were needing to go out for essentials (food / medicines), for a walk or some other exercise, and for work. In the UK, people with suspected mpox were directed to call sexual health services, likely explaining this association. Some people may have found this stigmatising as mpox is not a sexually transmitted infection.(57) Stigma surrounding having other sexually transmitted infections is associated with not seeking help.(58) In our study, immediate help seeking was associated with being willing to contact a sexual health clinic. These findings suggest that widening recommended points of contact with health services for suspected mpox cases or contacts may be beneficial. Taken together, results suggest that communications emphasising that others are also engaging with the protective behaviour and recommending non-stigmatising routes into health systems may improve engagement with self-isolation and help seeking.

In the Grindr sample, having been vaccinated for smallpox in 2022 was associated with having ever taken PrEP for HIV. This is probably due to how the vaccine was rolled out in the UK. GBMSM identified by sexual health services as being at highest risk of exposure – using markers similar to those used to assess eligibility for PrEP – were invited to be vaccinated.(59) Being vaccinated was also strongly associated with thinking that you were already immune to mpox in this survey. As this is a cross-sectional survey, we cannot infer

For peer review only - http://bmjopen.bmj.com/site/about/guidelines.xhtml

the direction of results, but it is likely that respondents believed they were immune to mpox because of their recent vaccination. This is interesting given limited information about the effectiveness of the vaccine at that time.

Strengths of the study include the collection of data from four large samples, including the general population and the population most affected by the recent mpox outbreak (GBMSM). We would also caution readers about several caveats for this study, including that participant socio-demographic characteristics differed by the recruitment method. Participants recruited through Grindr and Meta were more likely to be highly educated, higher socio-economic grade, and have less financial hardship. High rates of smallpox vaccination in these samples suggest that these groups are likely to be interested in and have personal experience relating to the outbreak and may have been more likely to engage with protective behaviours. We do not know if survey respondents are representative in terms of their beliefs, knowledge and intended behaviours with reference to the general population sample, GBMSM generally, and GBMSM who use Grindr and Meta. While overall rates of beliefs, knowledge and intended behaviours may be affected by sampling, associations within the data are likely to hold true.(60)

Other limitations relate to the survey and statistical measures used. The use of a cross-sectional survey means that answers may have been influenced by social desirability and recall bias, although the anonymity of a written survey may have mitigated these effects to some degree. Rates of intended behaviour are often higher than enacted behaviours.(11, 43) Therefore, rates of intended behaviours should be taken with caution. Our motivational messages were very brief and only repeated once, before measuring intended behaviours and behaviour-specific attitudes. A fully developed public communications campaign where people are exposed to co-produced and repeated messages may have more influence. There were high rates of actual and intended vaccination in the Grindr sample (94%). This may have affected our ability to detect associations. Variables were entered into regression models in blocks, with results reported for the final block entered (essentially all variables entered together). Therefore, we investigated the independent effect of a variable, accounting for all other variables included.

This study investigated mpox beliefs, knowledge and intended behaviours in a general population sample and in GBMSM (those most affected by the current outbreak). Intended uptake of protective behaviours differed by behaviour. GBMSM generally had higher intention to engage with protective behaviours, apart from self-isolation. This may have been

For peer review only - http://bmjopen.bmj.com/site/about/guidelines.xhtml

a function of sampling. Higher knowledge about mpox symptoms and transmission in GBMSM samples suggests that the public health messaging carried out by multiple stakeholders including the UK Health Security Agency, charities and via grass-roots community efforts has been successful. There was no impact of additional motivational messaging on intended uptake of protective behaviours. Associations between increased financial hardship and lower intention to enact protective behaviours suggest that providing financial support to those affected in future outbreaks may increase uptake.

For peer review only

For peer review only - http://bmjopen.bmj.com/site/about/guidelines.xhtml

FUNDING SOURCES

LS, JB, RA and GJR are supported by the National Institute for Health and Care Research Health Protection Research Unit (NIHR HPRU) in Emergency Preparedness and Response, a partnership between the UK Health Security Agency, King's College London and the University of East Anglia. Additionally, IO, LY, TM and RA are funded by the NIHR HPRU in Behavioural Science and Evaluation, a partnership between UKHSA and the University of Bristol. The views expressed are those of the authors and not necessarily those of the NIHR, UKHSA, or the Department of Health and Social Care. The study was funded by NIHR. For the purpose of open access, the author has applied a Creative Commons Attribution (CC BY) licence] to any Author Accepted Manuscript version arising.

DATA SHARING STATEMENT

Anonymised data are available online.(61)

AUTHOR CONTRIBUTION STATEMENT

All authors conceptualised the study and contributed to survey materials. LS completed analyses with guidance from HWWP, JB and GJR. LS wrote the first draft of the manuscript. All authors contributed to, and approved, the final manuscript. LS is guarantor. The corresponding author attests that all listed authors meet authorship criteria and that no others meeting the criteria have been omitted.

DISSEMINATION DECLARATION

Dissemination of survey results to participants is not possible due to the anonymous nature of data collection.

COMPETING INTERESTS STATEMENT

HWWP has received additional salary support from Public Health England and NHS England and receives funding from the National Institute of Health Research. HWWP receives consultancy fees to his employer from Ipsos MORI and has a PhD student who works at and has fees paid by AstraZeneca. GJR has acted as an expert witness in an unrelated case involving Bayer PLC, supported by LS. IO and RA are employees of UKHSA.

For peer review only

For peer review only - http://bmjopen.bmj.com/site/about/guidelines.xhtml

ACKNOWLEDGEMENTS

We would like to thank the Terrence Higgins Trust for their support with participant recruitment, and Savanta for hosting the survey. We would also like to thank study participants, for taking their time to complete the survey.

ETHICS

Ethical approval for this study was given by the King's College London Psychiatry, Nursing and Midwifery Research Ethics Panel (reference number: LRS/DP-21/22-32287). Participants gave informed consent before beginning survey materials.

For peer review only

REFERENCES

1. NHS. Monkeypox. 2022 [updated 1 June 2022, accessed 7 June 2022]. Available from: https://www.nhs.uk/conditions/monkeypox/.

2. World Health Organization. Multi-country monkeypox outbreak in non-endemic countries: situation update. 2022 [updated 4 June 2022, accessed 7 June 2022]. Available from: https://www.who.int/emergencies/disease-outbreak-news/item/2022-DON390.

3. World Health Organization. 2022 Monkeypox Outbreak: Global Trends 2022 [updated 1 December 2022, accessed 1 December 2022]. Available from: https://worldhealthorg.shinyapps.io/mpx_global/.

4. UK Health Security Agency. Monkeypox cases confirmed in England – latest updates. 2022 [updated 7 June 2022, accessed 7 June 2022]. Available from: https://www.gov.uk/government/news/monkeypox-cases-confirmed-in-england-latest-updates.

5. UK Health Security Agency. Monkeypox outbreak: epidemiological overview, 29 November 2022. 2022 [updated 29 November 2022, accessed 1 December 2022]. Available from: https://www.gov.uk/government/publications/monkeypox-outbreak-epidemiological-overview/monkeypox-outbreak-epidemiological-overview-29-november-2022.

6. UK Health Security Agency. Investigation into monkeypox outbreak in England: technical briefing 8. 2022 [updated 23 September 2022, accessed 31 October 2022]. Available from: https://www.gov.uk/government/publications/monkeypox-outbreak-technical-briefings/investigation-into-monkeypox-outbreak-in-england-technical-briefing-8#part-2-epidemiology-update.

7. UK Health Security Agency. Monkeypox contact tracing guidance: classification of contacts and follow-up advice for non-HCID strains of monkeypox. 2022 [updated 27 September 2022, accessed 6 October 2022]. Available from: https://assets.publishing.service.gov.uk/government/uploads/system/uploads/attachment_data/file/1106691/monkeypox-contact-tracing-classification-and-vaccination-matrix-version-13-27-sept-2022.pdf.

8. UK Health Security Agency. Recommendations for the use of pre and post exposure vaccination during a monkeypox incident (updated May 2022 v6.6). 21 May 2022. Available from:

For peer review only - http://bmjopen.bmj.com/site/about/guidelines.xhtml

https://assets.publishing.service.gov.uk/government/uploads/system/uploads/attachment_data/file/1077678/Recommendations-for-use-of-pre-and-post-exposure-vaccination-during-a-monkeypox-incident.pdf.

9. UKHSA finds vaccination offers strong protection against monkeypox [press release]. 22 November 2022. Available from: https://www.gov.uk/government/news/ukhsa-finds-vaccination-offers-strong-protection-against-monkeypox.

10. Smith LE, Potts HW, Amlot R, Fear NT, Michie S, Rubin GJ. Who is engaging with lateral flow testing for COVID-19 in the UK? The COVID-19 Rapid Survey of Adherence to Interventions and Responses (CORSAIR) study. BMJ Open. 2022;12(2):e058060.

11. Smith LE, Potts HWW, Amlot R, Fear NT, Michie S, Rubin GJ. Adherence to the test, trace, and isolate system in the UK: results from 37 nationally representative surveys. BMJ. 2021;372:n608.

12. Smith LE, Potts HWW, Amlot R, Fear NT, Michie S, Rubin GJ. Intention to adhere to test, trace, and isolate during the COVID-19 pandemic (the COVID-19 Rapid Survey of Adherence to Interventions and Responses study). Br J Health Psychol. 2021.

13. Rogers RW. A Protection Motivation Theory of Fear Appeals and Attitude Change. J Psychol. 1975;91(1):93-114.

14. Smith LE, Amlot R, Lambert H, Oliver I, Robin C, Yardley L, et al. Factors associated with adherence to self-isolation and lockdown measures in the UK: a cross-sectional survey. Public Health. 2020;187:41-52.

15. Smith LE, Sim J, Cutts M, Dasch H, Amlôt R, Sevdalis N, et al. Psychosocial factors affecting COVID-19 vaccine uptake in the UK: a prospective cohort study (CoVAccS – wave 3). medRxiv. 2022:2022.03.25.22272954.

16. Sherman SM, Sim J, Cutts M, Dasch H, Amlot R, Rubin GJ, et al. COVID-19 vaccination acceptability in the UK at the start of the vaccination programme: a nationally representative cross-sectional survey (CoVAccS - wave 2). Public Health. 2022;202:1-9.

17. Paul E, Steptoe A, Fancourt D. Attitudes towards vaccines and intention to vaccinate against COVID-19: Implications for public health communications. Lancet Reg Health Eur. 2021;1:100012.

For peer review only - http://bmjopen.bmj.com/site/about/guidelines.xhtml

18. Butter S, McGlinchey E, Berry E, Armour C. Psychological, social, and situational factors associated with COVID-19 vaccination intentions: A study of UK key workers and non-key workers. Br J Health Psychol. 2022;27(1):13-29.

19. Bish A, Yardley L, Nicoll A, Michie S. Factors associated with uptake of vaccination against pandemic influenza: a systematic review. Vaccine. 2011;29(38):6472-84.

20. Malik AA, Winters MS, Omer SB. Attitudes of the US general public towards Monkeypox. medRxiv. 2022:2022.06.20.22276527.

21. Temsah M-H, Aljamaan F, Alenezi S, Abouammoh N, Alhasan K, Dasuqi SA, et al. Comparison of Monkeypox disease knowledge and perception among the healthcare workers versus the general population during the first month of the WHO emerging infectious disease alert. medRxiv. 2022:2022.10.17.22281183.

22. Paparini S, Whitacre R, Smuk M, Thornhill J, Mwendera C, Strachan S, et al. Public understanding, awareness, and response to monkeypox virus outbreak: A cross-sectional survey of the most affected communities in the United Kingdom during the 2022 public health emergency. medRxiv. 2022:2022.08.25.22279207.

23. Temsah M-H, Aljamaan F, Alenezi S, Alhasan K, Saddik B, Al-Barag A, et al. Monkeypox caused less worry than COVID-19 among the general population during the first month of the WHO Monkeypox alert: Experience from Saudi Arabia. Travel Medicine and Infectious Disease. 2022;49:102426.

24. Dukers-Muijrers NH, Evers Y, Widdershoven V, Davidovich U, Adam PC, Op de Coul EL, et al. Monkeypox vaccination willingness, determinants, and communication needs in gay, bisexual, and other men who have sex with men, in the context of limited vaccine availability in the Netherlands (Dutch MPX-survey). medRxiv. 2022:2022.10.11.22280965.

25. Wang H, Abreu de Paulo KJI, Gültzow T, Zimmermann HML, Jonas KJ. Monkeypox self-diagnosis abilities, determinants of vaccination and self-isolation intention after diagnosis among MSM, the Netherlands, July 2022. Eurosurveillance. 2022;27(33):2200603.

26. Paparini S, Whitacre R, Smuk M, Thornhill J, Mwendera C, Strachan S, et al. Public understanding, awareness, and response to monkeypox virus outbreak: A cross-sectional survey of the most affected communities in the United Kingdom during the 2022 public health emergency. Personal communication.

For peer review only

For peer review only - http://bmjopen.bmj.com/site/about/guidelines.xhtml

27. Kapur GB, Bezek S, Dyal J. Effective communication during disasters : making use of technology, media, and human resources. Oakville: Apple Academic Press,; 2017.

28. Batteux E, Mills F, Jones LF, Symons C, Weston D. The Effectiveness of Interventions for Increasing COVID-19 Vaccine Uptake: A Systematic Review. Vaccines. 2022;10(3):386.

29. Office for National Statistics. Mid-Year Population Estimates, UK, June 2020. 2021 [updated 25 June 2021, accessed 14 October 2022]. Available from: https://www.ons.gov.uk/peoplepopulationandcommunity/populationandmigration/populationestimates/datasets/populationestimatesforukenglandandwalesscotlandandnorthernireland.

30. Rubin GJ, Amlôt R, Page L, Pearce J, Wessely S. Assessing Perceptions AbouT Hazardous Substances (PATHS): The PATHS questionnaire. Journal of Health Psychology. 2013;18(8):1100-13.

31. Rubin GJ, Bakhshi S, Amlôt R, Fear N, Potts HWW, Michie S. The design of a survey questionnaire to measure perceptions and behaviour during an influenza pandemic: the Flu TElephone Survey Template (FluTEST). Health Services and Delivery Research. 2014;2(41).

32. Smith LE, Potts HWW, Amlot R, Fear NT, Michie S, Rubin GJ. Holding a stigmatizing attitude at the start of the COVID-19 outbreak: A cross-sectional survey. Br J Health Psychol. 2021.

33. Sherman SM, Smith LE, Sim J, Amlot R, Cutts M, Dasch H, et al. COVID-19 vaccination intention in the UK: results from the COVID-19 vaccination acceptability study (CoVAccS), a nationally representative cross-sectional survey. Hum Vaccin Immunother. 2021;17(6):1612-21.

34. World Health Organization. Monkeypox. 2022 [updated 19 May 2022, accessed 6 June 2022]. Available from: https://www.who.int/news-room/fact-sheets/detail/monkeypox.

35. UK Health Security Agency. GUMCAD STI Surveillance System: Data specification and technical guidance. 2021. Available from: https://assets.publishing.service.gov.uk/government/uploads/system/uploads/attachment_data/file/1032965/GUMCAD_data_specification_and_technical_guidance_2021.pdf.

36. Office for National Statistics. Sexual orientation question development for Census 2021. No date. Available from:

For peer review only - http://bmjopen.bmj.com/site/about/guidelines.xhtml

https://www.ons.gov.uk/census/censustransformationprogramme/questiondevelopment/sexualorientationquestiondevelopmentforcensus2021#:~:text=Census%202021%20will%20be%20the,at%20the%20sexual%20orientation%20question.

37. Ipsos MediaCT. Social Grade: A Classification Tool. 2009. Available from: https://www.ipsos.com/sites/default/files/publication/6800-03/MediaCT_thoughtpiece_Social_Grade_July09_V3_WEB.pdf.

38. Organisation for Economic Co-operation and Development. Supplementary Questions: Optional Survey Questions for the OECD INFE Financial Literacy Core Questionnaire. 2012. Available from: https://www.oecd.org/finance/financial-education/49878153.pdf

39. Ministry of Housing Communities & Local Government. English indices of deprivation 2019. 2019 [updated 26 September 2019, accessed 17 October 2022]. Available from: https://www.gov.uk/government/statistics/english-indices-of-deprivation-2019.

40. Chen H, Cohen P, Chen S. How Big is a Big Odds Ratio? Interpreting the Magnitudes of Odds Ratios in Epidemiological Studies. Communications in Statistics - Simulation and Computation. 2010;39(4):860-4.

41. Annenberg Public Policy Center. Survey: 1 in 5 Americans Fear Getting Monkeypox but Many Know Little About It. 2022 [updated 29 July 2022, accessed 31 October 2022]. Available from: https://www.asc.upenn.edu/news-events/news/survey-1-5-americans-fear-getting-monkeypox-many-know-little-about-it.

42. Free C, Palmer MJ, McCarthy OL, Jerome L, Berendes S, Knight M, et al. Effectiveness of a behavioural intervention delivered by text messages (safetxt) on sexually transmitted reinfections in people aged 16-24 years: randomised controlled trial. BMJ. 2022;378:e070351.

43. Sniehotta FF, Scholz U, Schwarzer R. Bridging the intention–behaviour gap: Planning, self-efficacy, and action control in the adoption and maintenance of physical exercise. Psychology & Health. 2005;20(2):143-60.

44. Josh Parry. Monkeypox: Lack of vaccines sparks pause in UK rollout. BBC News Online. 2022 [updated 15 August 2022, accessed 11 November 2022]. Available from: https://www.bbc.co.uk/news/uk-62551821.

For peer review only - http://bmjopen.bmj.com/site/about/guidelines.xhtml

45. Spicknall IH, Pollock ED, Clay PA, Oster AM, Charniga K, Masters N, et al. Modeling the Impact of Sexual Networks in the Transmission of Monkeypox virus Among Gay, Bisexual, and Other Men Who Have Sex with Men - United States, 2022. MMWR Morb Mortal Wkly Rep. 2022;71(35):1131-5.

46. Endo A, Murayama H, Abbott S, Ratnayake R, Pearson CAB, Edmunds WJ, et al. Heavy-tailed sexual contact networks and the epidemiology of monkeypox outbreak in non-endemic regions, May 2022. medRxiv. 2022:2022.06.13.22276353.

47. Smith LE, Potts HWW, Amlôt R, Fear NT, Michie S, Rubin GJ. Patterns of social mixing in England changed in line with restrictions during the COVID-19 pandemic (September 2020 to April 2022). Scientific Reports. 2022;12(1):10436.

48. Wright L, Steptoe A, Fancourt D. Patterns of compliance with COVID-19 preventive behaviours: a latent class analysis of 20 000 UK adults. J Epidemiol Community Health. 2022;76(3):247-53.

49. Bish A, Michie S. Demographic and attitudinal determinants of protective behaviours during a pandemic: A review. British Journal of Health Psychology. 2010;15(4):797-824.

50. Smith LE, Potts HWW, Amlot R, Fear NT, Michie S, Rubin GJ. Worry and behaviour at the start of the COVID-19 outbreak: Results from three UK surveys (the COVID-19 rapid survey of Adherence to Interventions and responses [CORSAIR] study). Prev Med Rep. 2022;25:101686.

51. Dryhurst S, Schneider CR, Kerr J, Freeman ALJ, Recchia G, van der Bles AM, et al. Risk perceptions of COVID-19 around the world. Journal of Risk Research. 2020;23(7-8):994-1006.

52. Rubin GJ, Potts HW, Michie S. The impact of communications about swine flu (influenza A H1N1v) on public responses to the outbreak: results from 36 national telephone surveys in the UK. Health Technol Assess. 2010;14(34):183-266.

53. Rubin GJ, Amlôt R, Page L, Wessely S. Public perceptions, anxiety, and behaviour change in relation to the swine flu outbreak: cross sectional telephone survey. BMJ. 2009;339:b2651.

54. Teh B, Olsen K, Black J, Cheng AC, Aboltins C, Bull K, et al. Impact of swine influenza and quarantine measures on patients and households during the H1N1/09 pandemic. Scandinavian Journal of Infectious Diseases. 2012;44(4):289-96.

For peer review only - http://bmjopen.bmj.com/site/about/guidelines.xhtml

55. Smith LE, Sherman SM, Sim J, Amlôt R, Cutts M, Dasch H, et al. Parents' intention to vaccinate their child for COVID-19: a cross-sectional survey (CoVAccS – wave 3). medRxiv. 2022:2022.05.20.22275350.

56. Webster RK, Brooks SK, Smith LE, Woodland L, Wessely S, Rubin GJ. How to improve adherence with quarantine: rapid review of the evidence. Public Health. 2020;182:163-9.

57. März JW, Holm S, Biller-Andorno N. Monkeypox, stigma and public health. The Lancet Regional Health – Europe. 2022;23.

58. Stangl AL, Pliakas T, Izazola-Licea JA, Ayala G, Beattie TS, Ferguson L, et al. Removing the societal and legal impediments to the HIV response: An evidence-based framework for 2025 and beyond. PLOS ONE. 2022;17(2):e0264249.

59. Joint Committee on Vaccination and Immunisation. JCVI statement on vaccine dose prioritisation in response to the monkeypox outbreak. 2022 [updated 23 September 2022, accessed 11 November 2022]. Available from: https://www.gov.uk/government/publications/monkeypox-outbreak-jcvi-statement-on-vaccine-dose-prioritisation-september-2022/jcvi-statement-on-vaccine-dose-prioritisation-in-response-to-the-monkeypox-outbreak.

60. Kohler U. Possible Uses of Nonprobability Sampling for the Social Sciences. Survey Methods: Insights from the Field. 2019.

61. Smith LE. Mpox: Investigating public perceptions, understanding, and behaviours. 2022 [updated 7 December 2022, accessed 7 December 2022]. Available from: https://osf.io/n7p4c/.

For peer review only - http://bmjopen.bmj.com/site/about/guidelines.xhtml

Supplementary materials 1. Full survey materials and top-line results for general population and GBMSM samples separately

For categorical data, n (%) are shown. Percentages are column percentages. Where totals do not add up to 100%, it is due to rounding errors.

For continuous data, n (where different to total sample), mean (M), standard deviation (SD), and range are shown.

Total *ns* are: General population, n=3050; Savanta GBMSM, n=247; Grindr, n=831; Meta [Facebook and Instagram], n=1036.

Screening questions

ASK ALL
Postcode What is your full postcode?

We will only use your postcode to allocate you to groups based on your location (e.g. region). Your full postcode will not be passed on to the research team.

Type your answer below
OPEN END

OPEN TEXT ENTRY
Prefer not to say

New screen

ASK ALL
Gender What is your gender?
SINGLE CODE

	General population	Savanta GBMSM	Grindr	Meta
Male (including trans man)	1282 (42.0)	247 (100.0)	827 (99.5)	1030 (99.4)
Female (including trans woman)	1729 (56.7)	0 (0.0)	0 (0.0)	0 (0.0)
Non-binary	17 (0.6)	0 (0.0)	0 (0.0)	0 (0.0)
Other, please state	15 (0.5)	0 (0.0)	4 (0.5)	6 (0.6)
Prefer not to say	7 (0.2)	0 (0.0)	0 (0.0)	0 (0.0)

For peer review only - http://bmjopen.bmj.com/site/about/guidelines.xhtml

** For main analyses, where possible, text answers from "other, please state" were used to assign participants to a group. Therefore, numbers presented in the main manuscript may differ slightly.*

New screen

ASK ALL
SexBirth Is your gender the same as your gender assigned at birth?
SINGLE CODE

	General population	Savanta GBMSM	Grindr	Meta
Yes	3022 (99.1)	238 (96.4)	828 (99.6)	1013 (97.8)
No	22 (0.7)	9 (3.6)	2 (0.2)	18 (1.7)
Prefer not to say	6 (0.2)	0 (0.0)	1 (0.1)	5 (0.5)

New screen

ASK ALL
Age How old are you (in years)?
Type your answer below
NUMERIC - OPEN END

	General population	Savanta GBMSM	Grindr	Meta
OPEN TEXT ENTRY – number [cap at 110]	M=48.6, SD=17.4, range 18 to 98.	M=47.1, SD=16.5, range 18 to 77.	M=44.2, SD=12.5, range 18 to 82.	M=47.6, SD=11.9, range 18 to 79.

SCREEN OUT if under 18 years.

New screen

ASK ALL
Sexuality Which of the following best describes your sexual orientation?
SINGLE CODE, RANDOMISE

	General population	Savanta GBMSM	Grindr	Meta
Straight or heterosexual	2795 (91.6)	0 (0.0)	0 (0.0)	0 (0.0)
Gay or lesbian	78 (2.6)	230 (93.1)	706 (85.0)	967 (93.3)
Bisexual	135 (4.4)	16 (6.5)	118 (14.2)	55 (5.3)
Other sexual orientation, please state [ANCHOR]	18 (0.6)	1 (0.4)	7 (0.8)	14 (1.4)
Prefer not to say	24 (0.8)	0 (0.0)	0 (0.0)	0 (0.0)

For peer review only - http://bmjopen.bmj.com/site/about/guidelines.xhtml

[ANCHOR]				

** For main analyses, where possible, text answers from "other, please state" were used to assign participants to a group. Therefore, numbers presented in the main manuscript may differ slightly.*

New screen

ASK ALL

SEG Which of the following best describes the profession of the chief income earner in your household?

SINGLE CODE

	General population	Savanta GBMSM	Grindr	Meta
**High managerial, administrative or professional** e.g. doctor, lawyer, medium / large company director (50+ people)	193 (6.3)	28 (11.3)	144 (17.3)	253 (24.4)
**Intermediate managerial, administrative or professional** e.g. teacher, manager, accountant	608 (19.9)	57 (23.1)	308 (37.1)	381 (36.8)
**Supervisor, administrative or professional** e.g. police officer, nurse, secretary, self-employed	628 (20.6)	51 (20.6)	175 (21.1)	195 (18.8)
**Skilled manual worker** e.g. mechanic, plumber, electrician, lorry driver, train driver	395 (13.0)	17 (6.9)	42 (5.1)	18 (1.7)
**Semi-skilled or unskilled manual worker** e.g. waiter, factory worker, receptionist, labourer	328 (10.8)	18 (7.3)	45 (5.4)	31 (3.0)
**House-wife / house-husband**	76 (2.5)	3 (1.2)	4 (0.5)	0.0 (0.0)
**Unemployed**	176 (5.8)	24 (9.7)	27 (3.2)	26 (2.5)
**Student**	52 (1.7)	5 (2.0)	30 (3.6)	32 (3.1)
**Retired on a state pension**	215 (7.0)	9 (3.6)	15 (1.8)	13 (1.3)
**Retired on a private pension**	379 (12.4)	35 (14.2)	41 (4.9)	87 (8.4)

For peer review only - http://bmjopen.bmj.com/site/about/guidelines.xhtml

New screen

ASK ALL
QC This is a quality control question.

What colour is grass usually?

SINGLE CODE, RANDOMISE ORDER

	General population	Savanta GBMSM	Grindr	Meta
Purple	0 (0.0)	0 (0.0)	0 (0.0)	0 (0.0)
Pink	0 (0.0)	0 (0.0)	0 (0.0)	0 (0.0)
Orange	0 (0.0)	0 (0.0)	0 (0.0)	0 (0.0)
Green	3050 (100.0)	247 (100.0)	831 (100.0)	1036 (100.0)
White	0 (0.0)	0 (0.0)	0 (0.0)	0 (0.0)
Black	0 (0.0)	0 (0.0)	0 (0.0)	0 (0.0)

SCREEN OUT if do not select "green".

Beliefs and attitudes

New screen

There is an infection called "monkeypox" that has affected some people in the UK. We are interested to know your personal opinion about monkeypox, based on what you currently know.

For each question, please select the answer that reflects your opinion about monkeypox. Do not worry if you do not know what the best answer might be or if you are not at all familiar with monkeypox. We only ask that you try to give your answer based on what you think you know or what you would honestly decide to do in the situations described. If you are really not sure, please answer "don't know".

New screen

Q1. Before today, how much have you seen or heard about monkeypox?

	General population	Savanta GBMSM	Grindr	Meta
I have not seen or heard anything	117 (3.8)	6 (2.4)	7 (0.8)	2 (0.2)
I have seen or heard a little	2164 (71.0)	136 (55.1)	385 (46.3)	287 (27.7)
I have seen or heard a lot	759 (24.9)	103 (41.7)	439 (52.8)	747 (72.1)
Don't know	10 (0.3)	2 (0.8)	0 (0.0)	0 (0.0)

New screen

For peer review only - http://bmjopen.bmj.com/site/about/guidelines.xhtml

ASK ALL

Q2. Overall, how worried are you about monkeypox?

SINGLE CODE

	General population	Savanta GBMSM	Grindr	Meta
Not at all worried	635 (20.8)	40 (16.2)	77 (9.3)	47 (4.5)
Not very worried	1763 (57.8)	125 (50.6)	469 (56.4)	500 (48.3)
Very worried	452 (14.8)	61 (24.7)	234 (28.2)	410 (39.6)
Extremely worried	129 (4.2)	13 (5.3)	38 (4.6)	72 (6.9)
Don't know	71 (2.3)	8 (3.2)	13 (1.6)	7 (0.7)

New screen

ASK ALL

Q3. How much risk do you think monkeypox currently poses to:

Please select one option for each answer

RANDOMISE order of presentation of statements

People in the UK?

	General population	Savanta GBMSM	Grindr	Meta
No risk at all	106 (3.5)	9 (3.6)	11 (1.3)	3 (0.3)
Low risk	1537 (50.4)	102 (41.3)	357 (43.0)	427 (41.2)
Medium risk	1058 (34.7)	97 (39.3)	337 (40.6)	426 (41.1)
High risk	186 (6.1)	28 (11.3)	92 (11.1)	153 (14.8)
Very high risk	75 (2.5)	6 (2.4)	17 (2.0)	22 (2.1)
Don't know	88 (2.9)	5 (2.0)	17 (2.0)	5 (0.5)

You personally?

	General population	Savanta GBMSM	Grindr	Meta
No risk at all	700 (23.0)	48 (19.4)	42 (5.1)	44 (4.2)
Low risk	1619 (53.1)	96 (38.9)	291 (35.0)	266 (25.7)
Medium risk	441 (14.5)	71 (28.7)	303 (36.5)	332 (32.0)
High risk	136 (4.5)	15 (6.1)	148 (17.8)	299 (28.9)
Very high risk	58 (1.9)	13 (5.3)	37 (4.5)	93 (9.0)
Don't know	96 (3.1)	4 (1.6)	10 (1.2)	2 (0.2)

New screen

ASK ALL

Q4. How much, if at all, have you seen or heard about monkeypox? From:

The news (TV, online news websites or apps, printed newspapers, or radio)

	General population	Savanta GBMSM	Grindr	Meta

For peer review only - http://bmjopen.bmj.com/site/about/guidelines.xhtml

Not at all	210 (6.9)	14 (5.7)	61 (7.3)	54 (5.2)
A little	2125 (69.7)	149 (60.3)	533 (64.1)	652 (62.9)
A lot	693 (22.7)	82 (33.2)	235 (28.3)	330 (31.9)
Don't know	22 (0.7)	2 (0.8)	2 (0.2)	0 (0.0)

Official websites or helplines (e.g. NHS, GOV.UK), or an NHS GP practice, clinic or hospital

	General population	Savanta GBMSM	Grindr	Meta
Not at all	1547 (50.7)	98 (39.7)	198 (23.8)	188 (18.1)
A little	1113 (36.5)	97 (39.3)	450 (54.2)	601 (58.0)
A lot	316 (10.4)	48 (19.4)	169 (20.3)	241 (23.3)
Don't know	74 (2.4)	4 (1.6)	14 (1.7)	6 (0.6)

Speaking to friends, family, colleagues or other people you know (in person, by phone, text, WhatsApp, email, or in other ways)

	General population	Savanta GBMSM	Grindr	Meta
Not at all	1469 (48.2)	111 (44.9)	263 (31.6)	218 (21.0)
A little	1235 (40.5)	87 (35.2)	388 (46.7)	466 (45.0)
A lot	300 (9.8)	44 (17.8)	175 (21.1)	350 (33.8)
Don't know	46 (1.5)	5 (2.0)	5 (0.6)	2 (0.2)

Workers in community groups, community or faith leaders, charities, or volunteers who help improve the health and wellbeing of others

	General population	Savanta GBMSM	Grindr	Meta
Not at all	2111 (69.2)	165 (66.8)	553 (66.5)	633 (61.1)
A little	633 (20.8)	53 (21.5)	162 (19.5)	253 (24.4)
A lot	220 (7.2)	25 (10.1)	58 (7.0)	113 (10.9)
Don't know	86 (2.8)	4 (1.6)	58 (7.0)	37 (3.6)

Other places, including blogs, social media sites (e.g. Facebook, Twitter, Instagram), online communities or other websites

	General population	Savanta GBMSM	Grindr	Meta
Not at all	1426 (46.8)	100 (40.5)	214 (25.8)	138 (13.3)
A little	1215 (39.8)	100 (40.5)	427 (51.4)	534 (51.5)
A lot	357 (11.7)	43 (17.4)	179 (21.5)	361 (34.8)
Don't know	52 (1.7)	4 (1.6)	11 (1.3)	3 (0.3)

New screen

Q4_other. Please state any other places that you have seen or heard about monkeypox from.
Open ended, text entry
None button

New screen

For peer review only - http://bmjopen.bmj.com/site/about/guidelines.xhtml

For each question, please select the answer that reflects your opinion about monkeypox. Do not worry if you do not know what the best answer might be or if you are not at all familiar with monkeypox. We only ask that you try to give your answer based on what you think you know. If you are really not sure, please answer “don’t know”.

New screen

ASK ALL

Q5. How much do you agree or disagree with the following statements:

RANDOMISE statements

I have a good idea of how people catch monkeypox

	General population	Savanta GBMSM	Grindr	Meta
Strongly disagree	252 (8.2)	14 (5.6)	27 (3.2)	15 (1.4)
Disagree	727 (23.8)	35 (14.1)	46 (5.5)	31 (2.9)
Neither agree nor disagree	590 (19.3)	32 (12.9)	82 (9.8)	57 (5.5)
Agree	1159 (38.0)	121 (48.9)	468 (56.3)	559 (53.9)
Strongly agree	217 (7.1)	41 (16.5)	196 (23.5)	371 (35.8)
Don’t know	105 (3.4)	4 (1.6)	12 (1.4)	3 (0.2)

I know what the main symptoms of monkeypox are

	General population	Savanta GBMSM	Grindr	Meta
Strongly disagree	263 (8.6)	16 (6.5)	38 (4.6)	17 (1.6)
Disagree	820 (26.9)	44 (17.8)	100 (12.0)	60 (5.8)
Neither agree nor disagree	590 (19.3)	41 (16.6)	106 (12.8)	108 (10.4)
Agree	1113 (36.5)	120 (48.6)	493 (59.3)	642 (62.0)
Strongly agree	152 (5.0)	23 (9.3)	80 (9.6)	204 (19.7)
Don’t know	112 (3.7)	3 (1.2)	14 (1.7)	5 (0.5)

It would be easy for me to tell if someone I meet has monkeypox

	General population	Savanta GBMSM	Grindr	Meta
Strongly disagree	485 (15.9)	33 (13.4)	149 (17.9)	205 (19.8)
Disagree	1123 (36.8)	71 (28.7)	369 (44.4)	498 (48.1)
Neither agree nor disagree	663 (21.7)	64 (25.9)	181 (21.8)	209 (20.2)
Agree	504 (16.5)	54 (21.9)	88 (10.6)	92 (8.9)
Strongly agree	119 (3.9)	14 (5.7)	15 (1.8)	15 (1.4)
Don’t know	156 (5.1)	11 (4.5)	29 (3.5)	17 (1.6)

New screen

For peer review only - http://bmjopen.bmj.com/site/about/guidelines.xhtml

ASK ALL

Q6. How much do you agree or disagree with the following statements:

RANDOMISE statements

In the near future, it is likely that some of the people I come into physical contact with (touch) will have monkeypox

	General population	Savanta GBMSM	Grindr	Meta
Strongly disagree	275 (9.0)	13 (5.3)	23 (2.8)	23 (2.2)
Disagree	743 (24.4)	56 (22.7)	144 (17.3)	145 (14.0)
Neither agree nor disagree	912 (29.9)	65 (26.3)	237 (28.5)	238 (23.0)
Agree	632 (20.7)	73 (29.6)	297 (35.7)	470 (45.4)
Strongly agree	112 (3.7)	12 (4.9)	43 (5.2)	118 (11.4)
Don't know	376 (12.3)	28 (11.3)	87 (10.5)	42 (4.1)

If I come into physical contact with (touch) someone who has monkeypox, it is likely that I will catch it

	General population	Savanta GBMSM	Grindr	Meta
Strongly disagree	108 (3.5)	5 (2.0)	20 (2.4)	9 (0.9)
Disagree	467 (15.3)	24 (9.7)	93 (11.2)	145 (14.0)
Neither agree nor disagree	814 (26.7)	59 (23.9)	167 (20.1)	207 (20.0)
Agree	944 (31.0)	99 (40.1)	376 (45.2)	497 (48.0)
Strongly agree	207 (6.8)	25 (10.1)	98 (11.8)	135 (13.0)
Don't know	510 (16.7)	35 (14.2)	77 (9.3)	43 (4.2)

Monkeypox would be a serious illness for me

	General population	Savanta GBMSM	Grindr	Meta
Strongly disagree	224 (7.3)	16 (6.5)	30 (3.6)	26 (2.5)
Disagree	546 (17.9)	35 (14.2)	144 (17.3)	160 (15.4)
Neither agree nor disagree	744 (24.4)	58 (23.5)	182 (21.9)	220 (21.2)
Agree	787 (25.8)	85 (34.4)	290 (34.9)	413 (39.9)
Strongly agree	274 (9.0)	28 (11.3)	116 (14.0)	166 (16.0)
Don't know	475 (15.6)	25 (10.1)	69 (8.3)	51 (4.9)

People who catch monkeypox usually make a full recovery, even if they do not receive any treatment

	General population	Savanta GBMSM	Grindr	Meta
Strongly disagree	52 (1.7)	0 (0.0)	11 (1.3)	6 (0.6)
Disagree	264 (8.7)	25 (10.1)	77 (9.3)	62 (6.0)
Neither agree nor disagree	844 (27.7)	56 (22.7)	117 (14.1)	144 (13.9)
Agree	977 (32.0)	96 (38.9)	383 (46.1)	530 (51.2)

For peer review only - http://bmjopen.bmj.com/site/about/guidelines.xhtml

Strongly agree	228 (7.5)	29 (11.7)	102 (12.3)	210 (20.3)
Don't know	685 (22.5)	41 (16.6)	141 (17.0)	84 (8.1)

My personal behaviour has an impact on how monkeypox spreads

	General population	Savanta GBMSM	Grindr	Meta
Strongly disagree	498 (16.3)	24 (9.7)	53 (6.4)	28 (2.7)
Disagree	581 (19.0)	31 (12.6)	78 (9.4)	71 (6.9)
Neither agree nor disagree	572 (18.8)	40 (16.2)	91 (11.0)	67 (6.5)
Agree	834 (27.3)	95 (38.5)	384 (46.2)	552 (53.3)
Strongly agree	278 (9.1)	45 (18.2)	191 (23.0)	312 (30.1)
Don't know	287 (9.4)	12 (4.9)	34 (4.1)	6 (0.6)

My life has been negatively affected by changes made in response to the monkeypox outbreak

	General population	Savanta GBMSM	Grindr	Meta
Strongly disagree	1181 (38.7)	90 (36.4)	167 (20.1)	160 (15.4)
Disagree	981 (32.2)	75 (30.4)	289 (34.8)	300 (29.0)
Neither agree nor disagree	420 (13.8)	37 (15.0)	168 (20.2)	217 (20.9)
Agree	246 (8.1)	24 (9.7)	144 (17.3)	263 (25.4)
Strongly agree	115 (3.8)	12 (4.9)	42 (5.1)	88 (8.5)
Don't know	107 (3.5)	9 (3.6)	21 (2.5)	8 (0.8)

New screen

Below are some claims that some people have made about monkeypox. For each one, please say whether you strongly disagree, disagree, neither agree nor disagree, agree, or strongly agree.

ASK ALL
Q7. How much do you agree or disagree with the following claims:
SINGLE CODE

RANDOMISE statements

The risks of monkeypox are being exaggerated

	General population	Savanta GBMSM	Grindr	Meta
Strongly disagree	170 (5.6)	23 (9.3)	99 (11.9)	175 (16.9)
Disagree	822 (27.0)	87 (35.2)	326 (39.2)	505 (48.7)
Neither agree nor disagree	998 (32.7)	65 (26.3)	221 (26.6)	214 (20.7)
Agree	529 (17.3)	39 (15.8)	89 (10.7)	89 (8.6)
Strongly agree	184 (6.0)	11 (4.5)	34 (4.1)	19 (1.8)
Don't know	347 (11.4)	22 (8.9)	62 (7.5)	34 (3.3)

For peer review only - http://bmjopen.bmj.com/site/about/guidelines.xhtml

Because of the monkeypox outbreak, it is best to avoid physical contact with (touch) men who are gay, bisexual or have sex with men

	General population	Savanta GBMSM	Grindr	Meta
Strongly disagree	422 (13.8)	50 (20.2)	237 (28.5)	294 (28.4)
Disagree	707 (23.2)	64 (25.9)	267 (32.1)	357 (34.5)
Neither agree nor disagree	667 (21.9)	44 (17.8)	147 (17.7)	172 (16.6)
Agree	611 (20.0)	51 (20.6)	132 (15.9)	161 (15.5)
Strongly agree	305 (10.0)	22 (8.9)	22 (2.6)	37 (3.6)
Don't know	338 (11.1)	16 (6.5)	26 (3.1)	15 (1.4)

Because of the monkeypox outbreak, it is best to avoid physical contact with (touch) people from Africa

	General population	Savanta GBMSM	Grindr	Meta
Strongly disagree	462 (15.1)	52 (21.1)	327 (39.4)	431 (41.6)
Disagree	845 (27.7)	69 (27.9)	273 (32.9)	399 (38.5)
Neither agree nor disagree	768 (25.2)	57 (23.1)	118 (14.2)	127 (12.3)
Agree	454 (14.9)	40 (16.2)	59 (7.1)	43 (4.2)
Strongly agree	175 (5.7)	10 (4.0)	14 (1.7)	11 (1.1)
Don't know	346 (11.3)	19 (7.7)	40 (4.8)	25 (2.4)

Monkeypox is only a risk to men who are gay, bisexual or have sex with men

	General population	Savanta GBMSM	Grindr	Meta
Strongly disagree	536 (17.6)	77 (31.2)	375 (45.1)	431 (41.6)
Disagree	909 (29.8)	75 (30.4)	302 (36.3)	449 (43.3)
Neither agree nor disagree	586 (19.2)	35 (14.2)	56 (6.7)	63 (6.1)
Agree	521 (17.1)	30 (12.1)	61 (7.3)	65 (6.3)
Strongly agree	161 (5.3)	15 (6.1)	14 (1.7)	21 (2.0)
Don't know	337 (11.0)	15 (6.1)	23 (2.8)	7 (0.7)

New screen

ASK ALL

Q8. What do you think the main symptoms of monkeypox are?

Please select up to 4

MULTICODE – MAX 4

RANDOMISE

	General population	Savanta GBMSM	Grindr	Meta
High temperature / fever	1081 (35.4)	105 (42.5)	445 (53.5)	632 (61.0)

For peer review only - http://bmjopen.bmj.com/site/about/guidelines.xhtml

Shivering / chills	501 (16.4)	35 (14.2)	151 (18.2)	232 (22.4)
Muscle aches	542 (17.8)	47 (19.0)	249 (30.0)	339 (32.7)
Pain in your arms, legs or joints	385 (12.6)	45 (18.2)	117 (14.1)	170 (16.4)
Backache	104 (3.4)	10 (4.0)	19 (2.3)	23 (2.2)
Headache	426 (14.0)	39 (15.8)	146 (17.6)	181 (17.5)
Exhaustion	320 (10.5)	37 (15)	169 (20.3)	226 (21.8)
Swollen glands	496 (16.3)	68 (27.5)	256 (30.8)	446 (43.1)
Unexplained rash with blisters	1688 (55.3)	147 (59.5)	647 (77.9)	872 (84.2)
Unexplained rash without blisters	632 (20.7)	65 (26.3)	198 (23.8)	257 (24.8)
Runny or blocked nose	127 (4.2)	5 (2.0)	16 (1.9)	10 (1.0)
Dizziness	160 (5.2)	12 (4.9)	23 (2.8)	18 (1.7)
Sore throat	281 (9.2)	30 (12.1)	72 (8.7)	88 (8.5)
Nausea / feeling sick, or vomiting	388 (12.7)	46 (18.6)	68 (8.2)	77 (7.4)
Diarrhoea	182 (6)	17 (6.9)	31 (3.7)	32 (3.1)
Other, please state [ANCHOR]	5 (0.2)	0 (0.0)	9 (1.1)	12 (1.2)
Don't know [ANCHOR, EXCLUSIVE]	562 (18.4)	23 (9.3)	48 (5.8)	25 (2.4)

†Participants could select up to four answers to this question, therefore column totals add to more than 100%.

New screen

ASK ALL
Q9. You can catch monkeypox if:
Please select one option for each answer

RANDOMISE statements

You touch the rash of a person who has monkeypox

	General population	Savanta GBMSM	Grindr	Meta
Strongly disagree	68 (2.2)	3 (1.2)	11 (1.3)	6 (0.6)
Disagree	176 (5.8)	10 (4.0)	23 (2.8)	15 (1.4)
Neither agree nor disagree	516 (16.9)	28 (11.3)	48 (5.8)	38 (3.7)
Agree	1326 (43.5)	112 (45.3)	394 (47.4)	424 (40.9)
Strongly agree	394 (12.9)	65 (26.3)	293 (35.3)	519 (50.1)
Don't know	570 (18.7)	29 (11.7)	62 (7.5)	34 (3.3)

You are coughed or sneezed on by a person who has monkeypox

	General	Savanta GBMSM	Grindr	Meta

For peer review only - http://bmjopen.bmj.com/site/about/guidelines.xhtml

	population			
Strongly disagree	82 (2.7)	17 (6.9)	50 (6.0)	82 (7.9)
Disagree	356 (11.7)	39 (15.8)	191 (23.0)	253 (24.4)
Neither agree nor disagree	634 (20.8)	41 (16.6)	127 (15.3)	160 (15.4)
Agree	1024 (33.6)	87 (35.2)	251 (30.2)	344 (33.2)
Strongly agree	273 (9.0)	22 (8.9)	74 (8.9)	82 (7.9)
Don't know	681 (22.3)	41 (16.6)	138 (16.6)	115 (11.1)

You touch a person who has monkeypox, even if they do not have a rash

	General population	Savanta GBMSM	Grindr	Meta
Strongly disagree	91 (3.0)	4 (1.6)	26 (3.1)	20 (1.9)
Disagree	419 (13.7)	28 (11.3)	139 (16.7)	189 (18.2)
Neither agree nor disagree	634 (20.8)	61 (24.7)	125 (15.0)	198 (19.1)
Agree	932 (30.6)	91 (36.8)	337 (40.6)	410 (39.6)
Strongly agree	237 (7.8)	24 (9.7)	73 (8.8)	95 (9.2)
Don't know	737 (24.2)	39 (15.8)	131 (15.8)	124 (12)

You have sex with someone who has monkeypox

	General population	Savanta GBMSM	Grindr	Meta
Strongly disagree	72 (2.4)	5 (2.0)	12 (1.4)	10 (1.0)
Disagree	99 (3.2)	4 (1.6)	5 (0.6)	7 (0.7)
Neither agree nor disagree	359 (11.8)	17 (6.9)	24 (2.9)	16 (1.5)
Agree	1293 (42.4)	120 (48.6)	387 (46.6)	441 (42.6)
Strongly agree	802 (26.3)	89 (36.0)	377 (45.4)	551 (53.2)
Don't know	425 (13.9)	12 (4.9)	26 (3.1)	11 (1.1)

You come within 1 metre of someone who has monkeypox

	General population	Savanta GBMSM	Grindr	Meta
Strongly disagree	357 (11.7)	59 (23.9)	269 (32.4)	407 (39.3)
Disagree	972 (31.9)	93 (37.7)	379 (45.6)	438 (42.3)
Neither agree nor disagree	598 (19.6)	29 (11.7)	72 (8.7)	84 (8.1)
Agree	358 (11.7)	34 (13.8)	39 (4.7)	50 (4.8)
Strongly agree	131 (4.3)	4 (1.6)	10 (1.2)	11 (1.1)
Don't know	634 (20.8)	28 (11.3)	62 (7.5)	46 (4.4)

You touch something (e.g. towels, bedding or clothing) that has been touched by a person who has monkeypox

	General population	Savanta GBMSM	Grindr	Meta
Strongly disagree	87 (2.9)	7 (2.8)	21 (2.5)	18 (1.7)

For peer review only

Disagree	414 (13.6)	30 (12.1)	85 (10.2)	72 (6.9)
Neither agree nor disagree	653 (21.4)	46 (18.6)	100 (12.0)	89 (8.6)
Agree	961 (31.5)	96 (38.9)	379 (45.6)	585 (56.5)
Strongly agree	248 (8.1)	35 (14.2)	142 (17.1)	204 (19.7)
Don't know	687 (22.5)	33 (13.4)	104 (12.5)	68 (6.6)

You touch a pet animal that has monkeypox

	General population	Savanta GBMSM	Grindr	Meta
Strongly disagree	130 (4.3)	14 (5.7)	61 (7.3)	67 (6.5)
Disagree	469 (15.4)	43 (17.4)	140 (16.8)	151 (14.6)
Neither agree nor disagree	693 (22.7)	57 (23.1)	159 (19.1)	208 (20.1)
Agree	740 (24.3)	62 (25.1)	219 (26.4)	341 (32.9)
Strongly agree	193 (6.3)	24 (9.7)	46 (5.5)	82 (7.9)
Don't know	825 (27.0)	47 (19)	206 (24.8)	187 (18.1)

Intended behaviour

New screen

[For Savanta gen pop sample, randomise people to four groups. Ppts should see EITHER group A OR group B OR group C OR group D.
For Savanta GBMSM, Grindr, Meta samples, randomise people to two groups. Ppts should see EITHER group E OR group F.]

[Savanta gen pop] GROUP A (risk + perceived necessity/efficacy): Monkeypox causes mild illness for most people, but it can be very severe and can even kill some people. Pets may also be able to catch monkeypox. People who have recently been in contact with someone who might have monkeypox and get an unexplained rash should get tested quickly. If they test positive, they should avoid close contact with others to stop monkeypox from spreading further.

[Savanta gen pop] GROUP B (risk + perceived benefits): Monkeypox causes mild illness for most people, but it can be very severe and can even kill some people. Pets may also be able to catch monkeypox. People who test positive for monkeypox can take action to protect their friends, family, and pets from infection.

[Savanta gen pop] GROUP C (risk + perceived costs): Monkeypox causes mild illness for most people, but it can be very severe and can even kill some people. Pets may also be able to catch monkeypox. Imagine that people who have to self-isolate because they test positive for monkeypox will be provided with the practical and financial support that they need to do that.

For peer review only - http://bmjopen.bmj.com/site/about/guidelines.xhtml

[Savanta gen pop] GROUP D (control message): Monkeypox is a rare infection that is mainly found in parts of west or central Africa. Recently, there has been an outbreak of monkeypox in other countries. Since early May, more than 40,000 monkeypox cases have been reported in over 80 countries where the virus is not normally seen, including the UK.

[Savanta GBMSM / Grindr / Meta] GROUP E (all messages): Monkeypox causes mild illness for most people, but it can be very severe and can even kill some people. Pets may also be able to catch monkeypox.

People who have recently been in contact with someone who might have monkeypox and get an unexplained rash should get tested quickly. If they test positive, they should avoid close contact with others to stop monkeypox from spreading further. People who test positive for monkeypox can take action to protect their friends, family, and pets from infection. Imagine that people who have to self-isolate because they test positive for monkeypox will be provided with the practical and financial support that they need to do that.

[Savanta GBMSM / Grindr / Meta] GROUP F (control message): Monkeypox is a rare infection that is mainly found in parts of west or central Africa. Recently, there has been an outbreak of monkeypox in other countries.

There is a new outbreak of monkeypox in the UK. Since early May, more than 40,000 monkeypox cases have been reported in over 80 countries where the virus is not normally seen, including the UK. The current outbreak is the first time that the virus has been passed from person to person in the UK, and so the UK Health Security Agency (UKHSA) is working with partners to put in place guidance and measures that will prevent the spread of infection.

New screen

Self-isolation

People who have monkeypox are being asked to self-isolate for 21 days (3 weeks). They are also being asked to tell public health officials about other people they have been in contact with.

Self-isolation means staying at home and not going to work, school or public areas. It means avoiding close contact with people you live with, spending time in separate rooms as much as possible, and not having visitors to your home.

[Randomise order of Q10 and Q11]

New screen

ASK ALL
Q10. Imagine you are contacted by public health officials after testing and told that **you have monkeypox**. They tell you that you need to self-isolate for 21 days.

Realistically, would you self-isolate for 21 days?
SINGLE CODE

For peer review only - http://bmjopen.bmj.com/site/about/guidelines.xhtml

Answer Options

	General population	Savanta GBMSM	Grindr	Meta
Definitely would not	106 (3.5)	10 (4.0)	56 (6.7)	41 (4.0)
Probably would not	283 (9.3)	24 (9.7)	116 (14.0)	144 (13.9)
Not sure	367 (12.0)	19 (7.7)	74 (8.9)	90 (8.7)
Probably would	799 (26.2)	76 (30.8)	260 (31.3)	327 (31.6)
Definitely would	1495 (49.0)	118 (47.8)	325 (39.1)	434 (41.9)

New screen

ASK ALL

Q11. Imagine you are contacted by public health officials and told that **you have come into high-risk contact with someone who has monkeypox**. They tell you that you need to self-isolate for 21 days.

Realistically, would you self-isolate for 21 days?
SINGLE CODE
Answer Options

	General population	Savanta GBMSM	Grindr	Meta
Definitely would not	126 (4.1)	15 (6.1)	78 (9.4)	70 (6.8)
Probably would not	341 (11.2)	38 (15.4)	169 (20.3)	249 (24.0)
Not sure	483 (15.8)	24 (9.7)	124 (14.9)	172 (16.6)
Probably would	907 (29.7)	77 (31.2)	261 (31.4)	322 (31.1)
Definitely would	1193 (39.1)	93 (37.7)	199 (23.9)	223 (21.5)

New screen

ASK ALL

Q12. What would stop you from being able to self-isolate for 21 days?

Please select all that apply
MULTI CODE, RANDOMISE ORDER
Answer Options

	General population	Savanta GBMSM	Grindr	Meta
I would need to go out to get food, medicines, or other essentials	992 (32.5)	119 (48.2)	477 (57.4)	604 (58.3)
I would need to go out to work	542 (17.8)	52 (21.1)	309 (37.2)	323 (31.2)
I would need to go out to provide help or care to friends or relatives	238 (7.8)	22 (8.9)	53 (6.4)	72 (6.9)
I would need to go out as part of looking after my	396 (13.0)	9 (3.6)	21 (2.5)	15 (1.4)

For peer review only - http://bmjopen.bmj.com/site/about/guidelines.xhtml

children (e.g. to take them to school, or a playground)				
I would need to go out for a walk or some other exercise	765 (25.1)	72 (29.1)	388 (46.7)	550 (53.1)
My home is too small	192 (6.3)	17 (6.9)	103 (12.4)	109 (10.5)
I would be too bored or lonely	344 (11.3)	39 (15.8)	170 (20.5)	242 (23.4)
I don't think it's necessary for me to self-isolate	207 (6.8)	12 (4.9)	54 (6.5)	85 (8.2)
Other, please state	40 (1.3)	3 (1.2)	40 (4.8)	52 (5.0)
Nothing, I would stay at home for 21 days [EXCLUSIVE]	1166 (38.2)	76 (30.8)	153 (18.4)	185 (17.9)

†Participants could select more than one item for this question, therefore column totals add to more than 100%.

New screen

Now we would like you to think about a different scenario.

Help seeking

New screen

Imagine that tomorrow morning **you develop an unexplained rash with blisters**. You also learn that **you have come into contact with someone who might have monkeypox**.

ASK ALL
Q13 If **you** got these symptoms, which of these things would you do?

RANDOMISE order

Wait a day or two to see if they get better or clear up on their own

	General population	Savanta GBMSM	Grindr	Meta
Definitely would not	344 (11.3)	27 (10.9)	130 (15.6)	195 (18.8)
Probably would not	546 (17.9)	47 (19.0)	175 (21.1)	248 (23.9)
Not sure	809 (26.5)	58 (23.5)	145 (17.4)	217 (20.9)
Probably would	1080 (35.4)	92 (37.2)	323 (38.9)	326 (31.5)
Definitely would	271 (8.9)	23 (9.3)	58 (7.0)	50 (4.8)

For peer review only - http://bmjopen.bmj.com/site/about/guidelines.xhtml

Try to book an appointment with a GP

	General population	Savanta GBMSM	Grindr	Meta
Definitely would not	307 (10.1)	28 (11.3)	154 (18.5)	183 (17.7)
Probably would not	409 (13.4)	48 (19.4)	167 (20.1)	295 (28.5)
Not sure	586 (19.2)	40 (16.2)	125 (15.0)	148 (14.3)
Probably would	1032 (33.8)	86 (34.8)	221 (26.6)	222 (21.4)
Definitely would	716 (23.5)	45 (18.2)	164 (19.7)	188 (18.1)

Visit a Pharmacist/Chemist for advice

	General population	Savanta GBMSM	Grindr	Meta
Definitely would not	634 (20.8)	49 (19.8)	284 (34.2)	393 (37.9)
Probably would not	749 (24.6)	69 (27.9)	329 (39.6)	438 (42.3)
Not sure	727 (23.8)	64 (25.9)	97 (11.7)	125 (12.1)
Probably would	701 (23.0)	48 (19.4)	88 (10.6)	63 (6.1)
Definitely would	239 (7.8)	17 (6.9)	33 (4.0)	17 (1.6)

Go to A&E or visit another NHS service such as a walk-in centre or minor injuries unit

	General population	Savanta GBMSM	Grindr	Meta
Definitely would not	776 (25.4)	60 (24.3)	309 (37.2)	436 (42.1)
Probably would not	841 (27.6)	66 (26.7)	274 (33.0)	365 (35.2)
Not sure	682 (22.4)	54 (21.9)	110 (13.2)	113 (10.9)
Probably would	519 (17.0)	52 (21.1)	93 (11.2)	87 (8.4)
Definitely would	232 (7.6)	15 (6.1)	45 (5.4)	35 (3.4)

Call NHS 111 or 999 / ambulance service

	General population	Savanta GBMSM	Grindr	Meta
Definitely would not	246 (8.1)	22 (8.9)	145 (17.4)	185 (17.9)
Probably would not	358 (11.7)	30 (12.1)	153 (18.4)	233 (22.5)
Not sure	556 (18.2)	36 (14.6)	108 (13.0)	118 (11.4)
Probably would	1084 (35.5)	95 (38.5)	243 (29.2)	283 (27.3)
Definitely would	806 (26.4)	64 (25.9)	182 (21.9)	217 (20.9)

Visit a walk-in sexual health service

	General population	Savanta GBMSM	Grindr	Meta
Definitely would not	906 (29.7)	50 (20.2)	164 (19.7)	207 (20.0)
Probably would not	783 (25.7)	56 (22.7)	192 (23.1)	203 (19.6)
Not sure	727 (23.8)	51 (20.6)	125 (15.0)	130 (12.5)

For peer review only - http://bmjopen.bmj.com/site/about/guidelines.xhtml

Probably would	454 (14.9)	65 (26.3)	205 (24.7)	262 (25.3)
Definitely would	180 (5.9)	25 (10.1)	145 (17.4)	234 (22.6)

Call a sexual health service [fix below 6]

	General population	Savanta GBMSM	Grindr	Meta
Definitely would not	692 (22.7)	27 (10.9)	33 (4.0)	25 (2.4)
Probably would not	690 (22.6)	38 (15.4)	68 (8.2)	57 (5.5)
Not sure	697 (22.9)	35 (14.2)	60 (7.2)	60 (5.8)
Probably would	630 (20.7)	87 (35.2)	278 (33.5)	320 (30.9)
Definitely would	341 (11.2)	60 (24.3)	392 (47.2)	574 (55.4)

Let people you have recently been in close contact with know that you have symptoms

	General population	Savanta GBMSM	Grindr	Meta
Definitely would not	81 (2.7)	8 (3.2)	34 (4.1)	32 (3.1)
Probably would not	220 (7.2)	20 (8.1)	75 (9.0)	90 (8.7)
Not sure	495 (16.2)	47 (19.0)	152 (18.3)	177 (17.1)
Probably would	1185 (38.9)	96 (38.9)	298 (35.9)	396 (38.2)
Definitely would	1069 (35)	76 (30.8)	272 (32.7)	341 (32.9)

Search for information from official websites (e.g. NHS, GOV.UK)

	General population	Savanta GBMSM	Grindr	Meta
Definitely would not	73 (2.4)	4 (1.6)	13 (1.6)	9 (0.9)
Probably would not	122 (4.0)	10 (4.0)	26 (3.1)	23 (2.2)
Not sure	325 (10.7)	22 (8.9)	30 (3.6)	24 (2.3)
Probably would	1165 (38.2)	91 (36.8)	281 (33.8)	366 (35.3)
Definitely would	1365 (44.8)	120 (48.6)	481 (57.9)	614 (59.3)

Search for information from other places, including blogs, social media sites (e.g. Facebook, Twitter, Instagram), online communities or other websites [fix below 9]

	General population	Savanta GBMSM	Grindr	Meta
Definitely would not	533 (17.5)	49 (19.8)	144 (17.3)	135 (13.0)
Probably would not	647 (21.2)	54 (21.9)	231 (27.8)	277 (26.7)
Not sure	647 (21.2)	39 (15.8)	113 (13.6)	155 (15.0)
Probably would	789 (25.9)	70 (28.3)	206 (24.8)	301 (29.1)
Definitely would	434 (14.2)	35 (14.2)	137 (16.5)	168 (16.2)

New screen

For peer review only - http://bmjopen.bmj.com/site/about/guidelines.xhtml

Imagine that tomorrow morning **you develop an unexplained rash with blisters**. You also learn that **you have come into contact with someone who might have monkeypox**.

Q13_other. Please state any other things you would do.
Open ended text
Nothing button

Contact behaviour

New screen

Imagine that tomorrow morning **you develop an unexplained rash with blisters**. You also learn that **you have come into contact with someone who might have monkeypox**.

ASK ALL
Q14 If **you** got these symptoms, in the following 21 days, realistically how much, if at all, would you:

RANDOMISE order

Come into skin-to-skin contact with other people

	General population	Savanta GBMSM	Grindr	Meta
I would completely stop doing this	1883 (61.7)	153 (61.9)	648 (78)	858 (82.8)
Less than normal	412 (13.5)	41 (16.6)	127 (15.3)	135 (13.0)
Same as normal	249 (8.2)	21 (8.5)	15 (1.8)	12 (1.2)
More than normal	107 (3.5)	8 (3.2)	3 (0.4)	1 (0.1)
Not applicable, I wouldn't do this anyway	280 (9.2)	16 (6.5)	22 (2.6)	21 (2.0)
Don't know	109 (3.6)	7 (2.8)	15 (1.8)	9 (0.9)
Prefer not to say	10 (0.3)	1 (0.4)	1 (0.1)	0 (0.0)

Have sexual contact (from kissing to intercourse) with other people

	General population	Savanta GBMSM	Grindr	Meta
I would completely stop doing this	1923 (63.0)	171 (69.2)	721 (86.8)	922 (89.0)
Less than normal	238 (7.8)	20 (8.1)	54 (6.5)	61 (5.9)
Same as normal	216 (7.1)	15 (6.1)	14 (1.7)	7 (0.7)
More than normal	113 (3.7)	10 (4.0)	1 (0.1)	1 (0.1)
Not applicable, I wouldn't do this anyway	451 (14.8)	24 (9.7)	27 (3.2)	30 (2.9)
Don't know	93 (3.0)	6 (2.4)	13 (1.6)	13 (1.3)
Prefer not to say	16 (0.5)	1 (0.4)	1 (0.1)	2 (0.2)

Have sex without using a condom

For peer review only - http://bmjopen.bmj.com/site/about/guidelines.xhtml

	General population	Savanta GBMSM	Grindr	Meta
I would completely stop doing this	1729 (56.7)	155 (62.8)	623 (75)	788 (76.1)
Less than normal	146 (4.8)	17 (6.9)	33 (4.0)	37 (3.6)
Same as normal	235 (7.7)	15 (6.1)	27 (3.2)	29 (2.8)
More than normal	124 (4.1)	11 (4.5)	3 (0.4)	2 (0.2)
Not applicable, I wouldn't do this anyway	680 (22.3)	42 (17.0)	123 (14.8)	161 (15.5)
Don't know	111 (3.6)	7 (2.8)	17 (2.0)	14 (1.4)
Prefer not to say	25 (0.8)	0 (0.0)	5 (0.6)	5 (0.5)

Share bedding, towels, or clothes with other people

	General population	Savanta GBMSM	Grindr	Meta
I would completely stop doing this	1839 (60.3)	147 (59.5)	604 (72.7)	807 (77.9)
Less than normal	286 (9.4)	23 (9.3)	62 (7.5)	96 (9.3)
Same as normal	279 (9.1)	21 (8.5)	24 (2.9)	19 (1.8)
More than normal	124 (4.1)	12 (4.9)	2 (0.2)	1 (0.1)
Not applicable, I wouldn't do this anyway	415 (13.6)	41 (16.6)	126 (15.2)	104 (10.0)
Don't know	98 (3.2)	3 (1.2)	12 (1.4)	8 (0.8)
Prefer not to say	9 (0.3)	0 (0.0)	1 (0.1)	1 (0.1)

Go to a crowded place

	General population	Savanta GBMSM	Grindr	Meta
I would completely stop doing this	1872 (61.4)	141 (57.1)	552 (66.4)	663 (64.0)
Less than normal	418 (13.7)	50 (20.2)	183 (22)	297 (28.7)
Same as normal	248 (8.1)	23 (9.3)	57 (6.9)	39 (3.8)
More than normal	123 (4.0)	9 (3.6)	1 (0.1)	1 (0.1)
Not applicable, I wouldn't do this anyway	275 (9.0)	16 (6.5)	25 (3.0)	23 (2.2)
Don't know	103 (3.4)	8 (3.2)	13 (1.6)	12 (1.2)
Prefer not to say	11 (0.4)	0 (0.0)	0 (0.0)	1 (0.1)

Help or provide care for a vulnerable person

	General population	Savanta GBMSM	Grindr	Meta
I would completely stop doing this	1674 (54.9)	131 (53.0)	516 (62.1)	686 (66.2)
Less than normal	304 (10.0)	16 (6.5)	81 (9.7)	81 (7.8)
Same as normal	255 (8.4)	24 (9.7)	23 (2.8)	24 (2.3)
More than normal	160 (5.2)	14 (5.7)	8 (1.0)	6 (0.6)
Not applicable, I wouldn't do this anyway	513 (16.8)	50 (20.2)	181 (21.8)	217 (20.9)
Don't know	134 (4.4)	11 (4.5)	22 (2.6)	20 (1.9)
Prefer not to say	10 (0.3)	1 (0.4)	0 (0.0)	2 (0.2)

For peer review only - http://bmjopen.bmj.com/site/about/guidelines.xhtml

Go to a public place where you may come into physical contact with (touch) someone else

	General population	Savanta GBMSM	Grindr	Meta
I would completely stop doing this	1818 (59.6)	146 (59.1)	525 (63.2)	653 (63.0)
Less than normal	469 (15.4)	51 (20.6)	236 (28.4)	318 (30.7)
Same as normal	249 (8.2)	20 (8.1)	33 (4.0)	24 (2.3)
More than normal	143 (4.7)	9 (3.6)	2 (0.2)	2 (0.2)
Not applicable, I wouldn't do this anyway	259 (8.5)	11 (4.5)	24 (2.9)	24 (2.3)
Don't know	104 (3.4)	9 (3.6)	10 (1.2)	14 (1.4)
Prefer not to say	8 (0.3)	1 (0.4)	1 (0.1)	1 (0.1)

New screen

Imagine that tomorrow morning **you develop an unexplained rash with blisters**. You also learn that **you have come into contact with someone who might have monkeypox**.

Q15 How many days after the start of these symptoms would you:

Please give an approximate number if you are unsure. If you would not do this, please put 0.
Type your answer below

			General population	Savanta GBMSM	Grindr	Meta
Have sexual contact (from kissing to intercourse) with someone else	OPEN TEXT ENTRY – number [cap at 100]	ASK ONLY to those who answer 1-4 for similar question above	N=2021, M=15.7, SD=15.3, range 0 to 100.	N=190, M=16.2, SD=14.4, range 0 to 100.	N=746, M=20.4, SD=15.8, range 0 to 100.	N=947, M=20.0, SD=12.3, range 0 to 100.
Prefer not to say			469 (18.8)	26 (12.0)	44 (5.6)	44 (4.4)
Have sex without using a condom	OPEN TEXT ENTRY – number [cap at 100]	ASK ONLY to those who answer 1-4 for similar question above	N=1807, M=15.7, SD=16.8, range 0 to 100.	N=175, M=16.8, SD=18.9, range 0 to 100.	N=649, M=21.2, SD=19.5, range 0 to 100.	N=821, M=21.3, SD=18.0, range 0 to 100.
Prefer not to say			427 (19.1)	23 (11.6)	37 (5.4)	35 (4.1)

Contact sharing

New screen

For peer review only - http://bmjopen.bmj.com/site/about/guidelines.xhtml

People with suspected or confirmed monkeypox are being asked by public health officials to share contact details of people they have been in close contact with, so that they can be offered appropriate care and advice. Details of contacts are kept completely confidential.

ASK ALL

Q16 If **you** were asked for this information, would you try to share all the contact details of every …

RANDOMISE statements

Person who had been in your home (household members and visitors) in the last seven days

	General population	Savanta GBMSM	Grindr	Meta
Definitely would not	135 (4.4)	14 (5.7)	51 (6.1)	56 (5.4)
Probably would not	168 (5.5)	19 (7.7)	101 (12.2)	118 (11.4)
Not sure	392 (12.9)	30 (12.1)	97 (11.7)	120 (11.6)
Probably would	758 (24.9)	60 (24.3)	247 (29.7)	325 (31.4)
Definitely would	1552 (50.9)	120 (48.6)	331 (39.8)	414 (40.0)
Prefer not to say	45 (1.5)	4 (1.6)	4 (0.5)	3 (0.3)

Person you had sexual contact with (from kissing to intercourse) in the last seven days

	General population	Savanta GBMSM	Grindr	Meta
Definitely would not	169 (5.5)	17 (6.9)	49 (5.9)	54 (5.2)
Probably would not	162 (5.3)	16 (6.5)	59 (7.1)	67 (6.5)
Not sure	366 (12.0)	27 (10.9)	59 (7.1)	83 (8.0)
Probably would	629 (20.6)	56 (22.7)	236 (28.4)	282 (27.2)
Definitely would	1671 (54.8)	127 (51.4)	420 (50.5)	545 (52.6)
Prefer not to say	53 (1.7)	4 (1.6)	8 (1.0)	5 (0.5)

Place you had sexual contact with someone (from kissing to intercourse) in the last seven days

	General population	Savanta GBMSM	Grindr	Meta
Definitely would not	165 (5.4)	17 (6.9)	51 (6.1)	52 (5)
Probably would not	188 (6.2)	18 (7.3)	63 (7.6)	72 (6.9)
Not sure	415 (13.6)	28 (11.3)	72 (8.7)	91 (8.8)
Probably would	655 (21.5)	64 (25.9)	261 (31.4)	306 (29.5)
Definitely would	1559 (51.1)	116 (47)	375 (45.1)	510 (49.2)
Prefer not to say	68 (2.2)	4 (1.6)	9 (1.1)	5 (0.5)

Person you had skin-to-skin contact with (including hugging or kissing) in the last seven days

	General population	Savanta GBMSM	Grindr	Meta
Definitely would not	140 (4.6)	15 (6.1)	45 (5.4)	53 (5.1)

For peer review only - http://bmjopen.bmj.com/site/about/guidelines.xhtml

Probably would not	183 (6.0)	16 (6.5)	74 (8.9)	83 (8.0)
Not sure	352 (11.5)	25 (10.1)	74 (8.9)	116 (11.2)
Probably would	710 (23.3)	65 (26.3)	261 (31.4)	309 (29.8)
Definitely would	1621 (53.1)	122 (49.4)	373 (44.9)	472 (45.6)
Prefer not to say	44 (1.4)	4 (1.6)	4 (0.5)	3 (0.3)

Person you had shared bedding, towels, or clothes with in the last seven days

	General population	Savanta GBMSM	Grindr	Meta
Definitely would not	148 (4.9)	15 (6.1)	48 (5.8)	47 (4.5)
Probably would not	180 (5.9)	16 (6.5)	57 (6.9)	70 (6.8)
Not sure	352 (11.5)	26 (10.5)	69 (8.3)	81 (7.8)
Probably would	746 (24.5)	65 (26.3)	236 (28.4)	282 (27.2)
Definitely would	1574 (51.6)	122 (49.4)	415 (49.9)	551 (53.2)
Prefer not to say	50 (1.6)	3 (1.2)	6 (0.7)	5 (0.5)

Attitudes and beliefs

New screen

ASK ALL

Q17 How much do you agree or disagree with the following statements:

RANDOMISE statements

I wouldn't want to know the results of a monkeypox test

	General population	Savanta GBMSM	Grindr	Meta
Strongly disagree	1057 (34.7)	110 (44.5)	498 (59.9)	690 (66.6)
Disagree	963 (31.6)	63 (25.5)	235 (28.3)	243 (23.5)
Neither agree nor disagree	365 (12.0)	23 (9.3)	29 (3.5)	27 (2.6)
Agree	353 (11.6)	23 (9.3)	27 (3.2)	22 (2.1)
Strongly agree	240 (7.9)	23 (9.3)	35 (4.2)	51 (4.9)
Don't know	72 (2.4)	5 (2)	7 (0.8)	3 (0.3)

I would be worried what my friends or family would think about me if they thought I had monkeypox

	General population	Savanta GBMSM	Grindr	Meta
Strongly disagree	428 (14.0)	35 (14.2)	83 (10.0)	125 (12.1)
Disagree	624 (20.5)	43 (17.4)	114 (13.7)	147 (14.2)
Neither agree nor disagree	627 (20.6)	42 (17.0)	99 (11.9)	104 (10.0)
Agree	865 (28.4)	73 (29.6)	271 (32.6)	325 (31.4)
Strongly agree	402 (13.2)	52 (21.1)	263 (31.6)	333 (32.1)

For peer review only - http://bmjopen.bmj.com/site/about/guidelines.xhtml

Don’t know	104 (3.4)	2 (0.8)	1 (0.1)	2 (0.2)

I would be worried about how colleagues / my employer would react if they thought I had monkeypox

	General population	Savanta GBMSM	Grindr	Meta
Strongly disagree	366 (12)	31 (12.6)	79 (9.5)	99 (9.6)
Disagree	508 (16.7)	40 (16.2)	95 (11.4)	112 (10.8)
Neither agree nor disagree	649 (21.3)	37 (15.0)	82 (9.9)	109 (10.5)
Agree	914 (30.0)	67 (27.1)	249 (30.0)	309 (29.8)
Strongly agree	462 (15.1)	64 (25.9)	316 (38.0)	391 (37.7)
Don’t know	151 (5.0)	8 (3.2)	10 (1.2)	16 (1.5)

I don’t want to have a monkeypox test result on my medical record

	General population	Savanta GBMSM	Grindr	Meta
Strongly disagree	541 (17.7)	50 (20.2)	161 (19.4)	229 (22.1)
Disagree	801 (26.3)	63 (25.5)	193 (23.2)	260 (25.1)
Neither agree nor disagree	837 (27.4)	58 (23.5)	183 (22.0)	205 (19.8)
Agree	494 (16.2)	42 (17.0)	142 (17.1)	151 (14.6)
Strongly agree	257 (8.4)	28 (11.3)	142 (17.1)	171 (16.5)
Don’t know	120 (3.9)	6 (2.4)	10 (1.2)	20 (1.9)

An effective way to prevent the spread of monkeypox is for people who have symptoms to contact healthcare services

	General population	Savanta GBMSM	Grindr	Meta
Strongly disagree	67 (2.2)	7 (2.8)	16 (1.9)	19 (1.8)
Disagree	131 (4.3)	9 (3.6)	25 (3.0)	19 (1.8)
Neither agree nor disagree	459 (15.0)	24 (9.7)	58 (7.0)	64 (6.2)
Agree	1185 (38.9)	88 (35.6)	311 (37.4)	378 (36.5)
Strongly agree	1105 (36.2)	113 (45.7)	408 (49.1)	549 (53)
Don’t know	103 (3.4)	6 (2.4)	13 (1.6)	7 (0.7)

I would be willing to contact a sexual health clinic if I thought I had monkeypox symptoms or had come into contact with someone who had monkeypox

	General population	Savanta GBMSM	Grindr	Meta
Strongly disagree	149 (4.9)	7 (2.8)	21 (2.5)	20 (1.9)
Disagree	291 (9.5)	20 (8.1)	24 (2.9)	17 (1.6)
Neither agree nor disagree	590 (19.3)	28 (11.3)	36 (4.3)	39 (3.8)
Agree	1047 (34.3)	73 (29.6)	260 (31.3)	247 (23.8)
Strongly agree	800 (26.2)	110 (44.5)	481 (57.9)	709 (68.4)
Don’t know	173 (5.7)	9 (3.6)	9 (1.1)	4 (0.4)

For peer review only - http://bmjopen.bmj.com/site/about/guidelines.xhtml

New screen

ASK ALL

Q18 How much do you agree or disagree with the following statements:

RANDOMISE statements

If I had monkeypox symptoms, I wouldn't want to tell anyone as I don't want to self-isolate

	General population	Savanta GBMSM	Grindr	Meta
Strongly disagree	1055 (34.6)	79 (32.0)	291 (35.0)	374 (36.1)
Disagree	949 (31.1)	74 (30.0)	322 (38.7)	414 (40.0)
Neither agree nor disagree	431 (14.1)	33 (13.4)	98 (11.8)	119 (11.5)
Agree	365 (12.0)	32 (13.0)	72 (8.7)	78 (7.5)
Strongly agree	174 (5.7)	21 (8.5)	36 (4.3)	35 (3.4)
Don't know	76 (2.5)	8 (3.2)	12 (1.4)	16 (1.5)

If I had monkeypox symptoms, I wouldn't want to tell anyone so that others don't have to self-isolate

	General population	Savanta GBMSM	Grindr	Meta
Strongly disagree	909 (29.8)	71 (28.7)	260 (31.3)	305 (29.4)
Disagree	991 (32.5)	72 (29.1)	319 (38.4)	407 (39.3)
Neither agree nor disagree	468 (15.3)	37 (15.0)	122 (14.7)	143 (13.8)
Agree	396 (13)	35 (14.2)	86 (10.3)	114 (11.0)
Strongly agree	207 (6.8)	25 (10.1)	33 (4.0)	41 (4.0)
Don't know	79 (2.6)	7 (2.8)	11 (1.3)	26 (2.5)

Most people would self-isolate if they were told to

	General population	Savanta GBMSM	Grindr	Meta
Strongly disagree	132 (4.3)	14 (5.7)	84 (10.1)	90 (8.7)
Disagree	561 (18.4)	42 (17.0)	233 (28.0)	338 (32.6)
Neither agree nor disagree	634 (20.8)	51 (20.6)	180 (21.7)	222 (21.4)
Agree	1127 (37.0)	93 (37.7)	239 (28.8)	311 (30.0)
Strongly agree	461 (15.1)	32 (13.0)	51 (6.1)	36 (3.5)
Don't know	135 (4.4)	15 (6.1)	44 (5.3)	39 (3.8)

I have the support I need to self-isolate for 21 days

	General population	Savanta GBMSM	Grindr	Meta
Strongly disagree	173 (5.7)	26 (10.5)	142 (17.1)	132 (12.7)
Disagree	348 (11.4)	28 (11.3)	185 (22.3)	245 (23.6)
Neither agree nor disagree	458 (15.0)	41 (16.6)	129 (15.5)	127 (12.3)

For peer review only - http://bmjopen.bmj.com/site/about/guidelines.xhtml

Agree	1062 (34.8)	80 (32.4)	226 (27.2)	352 (34.0)
Strongly agree	942 (30.9)	66 (26.7)	133 (16.0)	168 (16.2)
Don't know	67 (2.2)	6 (2.4)	16 (1.9)	12 (1.2)

An effective way to prevent the spread of monkeypox is for people who have tested positive to self-isolate

	General population	Savanta GBMSM	Grindr	Meta
Strongly disagree	51 (1.7)	2 (0.8)	17 (2.0)	21 (2.0)
Disagree	107 (3.5)	13 (5.3)	16 (1.9)	34 (3.3)
Neither agree nor disagree	361 (11.8)	24 (9.7)	45 (5.4)	64 (6.2)
Agree	1018 (33.4)	68 (27.5)	319 (38.4)	362 (34.9)
Strongly agree	1424 (46.7)	132 (53.4)	416 (50.1)	543 (52.4)
Don't know	89 (2.9)	8 (3.2)	18 (2.2)	12 (1.2)

New screen

ASK ALL

Q19 How much do you agree or disagree with the following statements:

If I had to self-isolate because I had tested positive for monkeypox…

RANDOMISE statements

…I would lose touch with my friends and relatives

	General population	Savanta GBMSM	Grindr	Meta
Strongly disagree	567 (18.6)	44 (17.8)	151 (18.2)	180 (17.4)
Disagree	1039 (34.1)	78 (31.6)	320 (38.5)	408 (39.4)
Neither agree nor disagree	614 (20.1)	47 (19.0)	148 (17.8)	180 (17.4)
Agree	539 (17.7)	52 (21.1)	134 (16.1)	200 (19.3)
Strongly agree	225 (7.4)	19 (7.7)	61 (7.3)	65 (6.3)
Don't know	66 (2.2)	7 (2.8)	17 (2)	3 (0.3)

…it would have a severe impact on my family's wellbeing

	General population	Savanta GBMSM	Grindr	Meta
Strongly disagree	420 (13.8)	48 (19.4)	152 (18.3)	181 (17.5)
Disagree	740 (24.3)	58 (23.5)	279 (33.6)	350 (33.8)
Neither agree nor disagree	705 (23.1)	51 (20.6)	159 (19.1)	198 (19.1)
Agree	763 (25.0)	56 (22.7)	148 (17.8)	195 (18.8)
Strongly agree	338 (11.1)	21 (8.5)	81 (9.7)	99 (9.6)
Don't know	84 (2.8)	13 (5.3)	12 (1.4)	13 (1.3)

For peer review only - http://bmjopen.bmj.com/site/about/guidelines.xhtml

…it would have a negative impact on how much money I have

	General population	Savanta GBMSM	Grindr	Meta
Strongly disagree	498 (16.3)	52 (21.1)	132 (15.9)	184 (17.8)
Disagree	791 (25.9)	62 (25.1)	231 (27.8)	357 (34.5)
Neither agree nor disagree	566 (18.6)	27 (10.9)	99 (11.9)	153 (14.8)
Agree	694 (22.8)	56 (22.7)	162 (19.5)	169 (16.3)
Strongly agree	414 (13.6)	43 (17.4)	196 (23.6)	165 (15.9)
Don't know	87 (2.9)	7 (2.8)	11 (1.3)	8 (0.8)

…it would have a negative impact on my work

	General population	Savanta GBMSM	Grindr	Meta
Strongly disagree	545 (17.9)	52 (21.1)	76 (9.1)	98 (9.5)
Disagree	664 (21.8)	38 (15.4)	136 (16.4)	191 (18.4)
Neither agree nor disagree	622 (20.4)	49 (19.8)	100 (12)	136 (13.1)
Agree	697 (22.9)	55 (22.3)	260 (31.3)	325 (31.4)
Strongly agree	405 (13.3)	46 (18.6)	251 (30.2)	264 (25.5)
Don't know	117 (3.8)	7 (2.8)	8 (1.0)	22 (2.1)

…I would miss out on events and activities that I want to attend

	General population	Savanta GBMSM	Grindr	Meta
Strongly disagree	340 (11.1)	32 (13.0)	77 (9.3)	46 (4.4)
Disagree	471 (15.4)	33 (13.4)	106 (12.8)	107 (10.3)
Neither agree nor disagree	607 (19.9)	37 (15.0)	97 (11.7)	117 (11.3)
Agree	1097 (36.0)	84 (34.0)	354 (42.6)	457 (44.1)
Strongly agree	460 (15.1)	55 (22.3)	185 (22.3)	306 (29.5)
Don't know	75 (2.5)	6 (2.4)	12 (1.4)	3 (0.3)

Vaccination

New screen

People who have come into significant contact with someone who has tested positive for monkeypox are being asked to have a smallpox vaccine to reduce the risk of getting seriously ill. As monkeypox is caused by a virus similar to the one that causes smallpox, vaccines designed for smallpox are considered effective in preventing or reducing the severity of monkeypox.

For peer review only - http://bmjopen.bmj.com/site/about/guidelines.xhtml

Like all medicines, this vaccine can cause side effects, but not everyone gets them. The most common side effects are pain and itching at the injection site. Most side effects are mild and clear fully without any treatment within 7 days.

New screen

For each question, please select the answer that reflects your opinion. Do not worry if you do not know what the best answer might be or if you are not at all familiar with monkeypox or vaccination. We only ask that you try to give your answer based on what you think you know or what you would honestly decide to do in the situations described. If you are really not sure, please answer "don't know".

New screen

ASK ALL
Q20 How much do you agree or disagree with the following statements:

RANDOMISE statements

In general, vaccination is a good thing

	General population	Savanta GBMSM	Grindr	Meta
Strongly disagree	65 (2.1)	9 (3.6)	15 (1.8)	6 (0.6)
Disagree	119 (3.9)	4 (1.6)	5 (0.6)	3 (0.3)
Neither agree nor disagree	361 (11.8)	21 (8.5)	33 (4.0)	15 (1.4)
Agree	1019 (33.4)	59 (23.9)	189 (22.7)	142 (13.7)
Strongly agree	1400 (45.9)	148 (59.9)	584 (70.3)	869 (83.9)
Don't know	86 (2.8)	6 (2.4)	5 (0.6)	1 (0.1)

Most people like me will get a smallpox vaccination if advised

	General population	Savanta GBMSM	Grindr	Meta
Strongly disagree	82 (2.7)	5 (2.0)	24 (2.9)	15 (1.4)
Disagree	248 (8.1)	14 (5.7)	41 (4.9)	43 (4.2)
Neither agree nor disagree	533 (17.5)	31 (12.6)	93 (11.2)	122 (11.8)
Agree	1270 (41.6)	111 (44.9)	404 (48.6)	470 (45.4)
Strongly agree	717 (23.5)	69 (27.9)	223 (26.8)	365 (35.2)
Don't know	200 (6.6)	17 (6.9)	46 (5.5)	21 (2.0)

If I get a smallpox vaccination, I will be protected against monkeypox

	General population	Savanta GBMSM	Grindr	Meta
Strongly disagree	100 (3.3)	9 (3.6)	27 (3.2)	6 (0.6)
Disagree	385 (12.6)	19 (7.7)	71 (8.5)	64 (6.2)
Neither agree nor disagree	927 (30.4)	52 (21.1)	158 (19.0)	139 (13.4)

For peer review only - http://bmjopen.bmj.com/site/about/guidelines.xhtml

Agree	953 (31.2)	102 (41.3)	391 (47.1)	599 (57.8)
Strongly agree	250 (8.2)	29 (11.7)	117 (14.1)	190 (18.3)
Don't know	435 (14.3)	36 (14.6)	67 (8.1)	38 (3.7)

A smallpox vaccination could give me smallpox

	General population	Savanta GBMSM	Grindr	Meta
Strongly disagree	614 (20.1)	70 (28.3)	400 (48.1)	668 (64.5)
Disagree	931 (30.5)	65 (26.3)	277 (33.3)	264 (25.5)
Neither agree nor disagree	662 (21.7)	38 (15.4)	69 (8.3)	51 (4.9)
Agree	392 (12.9)	32 (13.0)	27 (3.2)	17 (1.6)
Strongly agree	136 (4.5)	9 (3.6)	8 (1.0)	6 (0.6)
Don't know	315 (10.3)	33 (13.4)	50 (6.0)	30 (2.9)

I might regret getting the smallpox vaccination if I later experienced side effects from it

	General population	Savanta GBMSM	Grindr	Meta
Strongly disagree	420 (13.8)	48 (19.4)	259 (31.2)	410 (39.6)
Disagree	825 (27.0)	74 (30.0)	350 (42.1)	421 (40.6)
Neither agree nor disagree	710 (23.3)	43 (17.4)	88 (10.6)	121 (11.7)
Agree	655 (21.5)	50 (20.2)	89 (10.7)	61 (5.9)
Strongly agree	268 (8.8)	19 (7.7)	31 (3.7)	17 (1.6)
Don't know	172 (5.6)	13 (5.3)	14 (1.7)	6 (0.6)

I am already immune to monkeypox

	General population	Savanta GBMSM	Grindr	Meta
Strongly disagree	676 (22.2)	77 (31.2)	269 (32.4)	366 (35.3)
Disagree	800 (26.2)	51 (20.6)	213 (25.6)	251 (24.2)
Neither agree nor disagree	532 (17.4)	30 (12.1)	106 (12.8)	132 (12.7)
Agree	228 (7.5)	24 (9.7)	57 (6.9)	99 (9.6)
Strongly agree	138 (4.5)	13 (5.3)	26 (3.1)	44 (4.2)
Don't know	676 (22.2)	52 (21.1)	160 (19.3)	144 (13.9)

I would be worried about experiencing side effects from a smallpox vaccination

	General population	Savanta GBMSM	Grindr	Meta
Strongly disagree	392 (12.9)	44 (17.8)	246 (29.6)	364 (35.1)
Disagree	793 (26.0)	72 (29.1)	294 (35.4)	407 (39.3)
Neither agree nor disagree	646 (21.2)	41 (16.6)	105 (12.6)	136 (13.1)
Agree	819 (26.9)	54 (21.9)	146 (17.6)	109 (10.5)
Strongly agree	278 (9.1)	28 (11.3)	23 (2.8)	18 (1.7)
Don't know	122 (4.0)	8 (3.2)	17 (2.0)	2 (0.2)

For peer review only - http://bmjopen.bmj.com/site/about/guidelines.xhtml

For peer review only

I would be worried that having a smallpox vaccine might make me infectious to others

	General population	Savanta GBMSM	Grindr	Meta
Strongly disagree	653 (21.4)	74 (30.0)	454 (54.6)	693 (66.9)
Disagree	1050 (34.4)	72 (29.1)	280 (33.7)	274 (26.4)
Neither agree nor disagree	605 (19.8)	33 (13.4)	46 (5.5)	32 (3.1)
Agree	378 (12.4)	31 (12.6)	18 (2.2)	15 (1.4)
Strongly agree	159 (5.2)	18 (7.3)	5 (0.6)	7 (0.7)
Don’t know	205 (6.7)	19 (7.7)	28 (3.4)	15 (1.4)

People who are likely to come into high-risk contact with monkeypox should have a smallpox vaccine

	General population	Savanta GBMSM	Grindr	Meta
Strongly disagree	63 (2.1)	5 (2.0)	13 (1.6)	9 (0.9)
Disagree	158 (5.2)	15 (6.1)	8 (1.0)	5 (0.5)
Neither agree nor disagree	554 (18.2)	32 (13)	57 (6.9)	32 (3.1)
Agree	1134 (37.2)	89 (36)	256 (30.8)	234 (22.6)
Strongly agree	873 (28.6)	89 (36)	465 (56)	745 (71.9)
Don’t know	268 (8.8)	17 (6.9)	32 (3.9)	11 (1.1)

New screen

ASK ALL

Q21 To the best of your knowledge, have you received a smallpox vaccine in 2022?

SINGLE CODE

Answer Options

	General population	Savanta GBMSM	Grindr	Meta
Yes	162 (5.3)	26 (10.5)	265 (31.9)	422 (40.7)
No. I was offered the vaccine, but didn’t want to have it	116 (3.8)	9 (3.6)	11 (1.3)	16 (1.5)
No, I haven’t been offered the vaccine	2609 (85.5)	202 (81.8)	536 (64.5)	587 (56.7)
Don’t know / can’t remember	163 (5.3)	10 (4.0)	19 (2.3)	11 (1.1)

New screen

ASK IF Q21=2,3,4

Q22 If you were advised by public health officials to have a smallpox vaccine **after coming into high-risk contact with someone who has monkeypox**, how likely would you be to have one?

For peer review only

SINGLE CODE

Answer Options

	General population, n=2888	Savanta GBMSM, n=221	Grindr, n=566	Meta, n=614
Definitely would not	115 (4.0)	6 (2.7)	16 (2.8)	10 (1.6)
Probably would not	139 (4.8)	15 (6.8)	13 (2.3)	4 (0.7)
Not sure	497 (17.2)	22 (10.0)	17 (3.0)	14 (2.3)
Probably would	814 (28.2)	48 (21.7)	95 (16.8)	51 (8.3)
Definitely would	1323 (45.8)	130 (58.8)	425 (75.1)	535 (87.1)

‡This question was not asked to people who had already had the smallpox vaccine in 2022, therefore total *ns* are different.

New screen

ASK IF Q21=2,3,4

Q23 Some people who have been in contact with someone who has monkeypox are being asked to self-isolate for 21 days. If having a smallpox vaccine means that you would have to self-isolate for less time, how likely would you be to get vaccinated?

SINGLE CODE

Answer Options

	General population, n=2888	Savanta GBMSM, n=221	Grindr, n=566	Meta, n=614
Definitely would not	114 (3.9)	5 (2.3)	17 (3.0)	9 (1.5)
Probably would not	128 (4.4)	9 (4.1)	17 (3.0)	8 (1.3)
Not sure	487 (16.9)	29 (13.1)	31 (5.5)	19 (3.1)
Probably would	822 (28.5)	45 (20.4)	92 (16.3)	55 (9.0)
Definitely would	1337 (46.3)	133 (60.2)	409 (72.3)	523 (85.2)

‡This question was not asked to people who had already had the smallpox vaccine in 2022, therefore total *ns* are different.

New screen

The smallpox vaccine is considered effective in preventing monkeypox or reducing the severity of monkeypox. Some people who are more likely to come into contact with monkeypox are being offered this vaccine.

ASK IF Q21=2,3,4

Q24 If you were offered a smallpox vaccine, how likely would you be to have one?

SINGLE CODE

Answer Options

	General population,	Savanta GBMSM,	Grindr, n=566	Meta, n=614

For peer review only - http://bmjopen.bmj.com/site/about/guidelines.xhtml

	n=2888	n=221		
Definitely would not	111 (3.8)	4 (1.8)	20 (3.5)	12 (2.0)
Probably would not	157 (5.4)	12 (5.4)	14 (2.5)	5 (0.8)
Not sure	518 (17.9)	21 (9.5)	20 (3.5)	21 (3.4)
Probably would	851 (29.5)	64 (29.0)	95 (16.8)	64 (10.4)
Definitely would	1251 (43.3)	120 (54.3)	417 (73.7)	512 (83.4)

‡This question was not asked to people who had already had the smallpox vaccine in 2022, therefore total *ns* are different.

New screen

ASK ALL
Q25. Do you have any further thoughts you would like to share with us about monkeypox, self-isolation, vaccines or anything else to do with monkeypox?

If you do not have anything to add, please put "None".

Type your answer below
OPEN END – require at least one alphabetic/numeric character

New screen

Sociodemographic variables

Now we are going to ask some questions about you. This survey is anonymous. This means that nobody can link you to your answers.

New screen

ASK ALL
D1A In the past month, **have you personally**:
Please select one option for each answer

Gone without enough food to eat

	General population	Savanta GBMSM	Grindr	Meta
Never	2006 (65.8)	171 (69.2)	670 (80.6)	938 (90.5)
Sometimes	663 (21.7)	48 (19.4)	121 (14.6)	85 (8.2)
Often	300 (9.8)	21 (8.5)	28 (3.4)	8 (0.8)
Don't know	63 (2.1)	7 (2.8)	7 (0.8)	3 (0.3)
Prefer not to say	18 (0.6)	0 (0.0)	5 (0.6)	2 (0.2)

Gone without an income

	General population	Savanta GBMSM	Grindr	Meta
Never	2014 (66)	172 (69.6)	653 (78.6)	890 (85.9)

For peer review only - http://bmjopen.bmj.com/site/about/guidelines.xhtml

For peer review only

Sometimes	600 (19.7)	43 (17.4)	105 (12.6)	106 (10.2)
Often	335 (11.0)	24 (9.7)	60 (7.2)	34 (3.3)
Don't know	69 (2.3)	6 (2.4)	2 (0.2)	2 (0.2)
Prefer not to say	32 (1.0)	2 (0.8)	11 (1.3)	4 (0.4)

Gone without fuel for heating or to cook food

	General population	Savanta GBMSM	Grindr	Meta
Never	2232 (73.2)	179 (72.5)	730 (87.8)	978 (94.4)
Sometimes	474 (15.5)	38 (15.4)	72 (8.7)	48 (4.6)
Often	255 (8.4)	25 (10.1)	22 (2.6)	6 (0.6)
Don't know	71 (2.3)	2 (0.8)	1 (0.1)	2 (0.2)
Prefer not to say	18 (0.6)	3 (1.2)	6 (0.7)	2 (0.2)

New screen

ASK ALL
D1B Last month, how difficult was it for you to cover your expenses and pay all your bills?
SINGLE CODE

	General population	Savanta GBMSM	Grindr	Meta
Not at all difficult	980 (32.1)	93 (37.7)	421 (50.7)	675 (65.2)
Somewhat difficult	1148 (37.6)	86 (34.8)	275 (33.1)	271 (26.2)
Very difficult	380 (12.5)	29 (11.7)	53 (6.4)	42 (4.1)
Extremely difficult	407 (13.3)	32 (13)	50 (6.0)	25 (2.4)
Don't know	55 (1.8)	1 (0.4)	2 (0.2)	2 (0.2)
Not applicable, I don't pay the bills in my household	53 (1.7)	5 (2.0)	22 (2.6)	10 (1.0)
Prefer not to say	27 (0.9)	1 (0.4)	8 (1)	11 (1.1)

New screen

ASK ALL
D2 How many people currently live in your household?

Please include yourself and all adults and children – including those not related to you
SINGLE CODE

	General population	Savanta GBMSM	Grindr	Meta
I live alone	663 (21.7)	101 (40.9)	358 (43.1)	380 (36.7)
2	1104 (36.2)	90 (36.4)	313 (37.7)	531 (51.3)

For peer review only - http://bmjopen.bmj.com/site/about/guidelines.xhtml

3 – 4	1055 (34.6)	42 (17.0)	131 (15.8)	103 (9.9)
5 – 6	204 (6.7)	13 (5.3)	25 (3.0)	15 (1.4)
7 +	24 (0.8)	1 (0.4)	4 (0.5)	7 (0.7)

New screen

ASK ALL
D3 Are you the parent / guardian of any dependent children?

Dependent children are those aged under 18 living in your household.
SINGLE CODE

	General population	Savanta GBMSM	Grindr	Meta
Yes, I have one or more dependent children	975 (32.0)	37 (15.0)	35 (4.2)	22 (2.1)
No, I don't have any dependent children	2075 (68.0)	210 (85.0)	796 (95.8)	1014 (97.9)

New screen

ASK ALL
D4. Have you ever provided voluntary care for a family member or friend who needs support due to old age, physical illness, disability, mental health problems, or addiction?
Please select one option
SINGLE CODE

	General population	Savanta GBMSM	Grindr	Meta
Yes, I currently do this	623 (20.4)	45 (18.2)	158 (19.0)	155 (15.0)
I have previously provided care for a friend/family member, but no longer do	897 (29.4)	68 (27.5)	223 (26.8)	315 (30.4)
I have never provided care for a family member/friend in this way	1424 (46.7)	121 (49)	422 (50.8)	541 (52.2)
Prefer not to say	106 (3.5)	13 (5.3)	28 (3.4)	25 (2.4)

New screen

ASK ALL
D5 Do you currently have any pets that live in your home?
MULTI CODE

For peer review only - http://bmjopen.bmj.com/site/about/guidelines.xhtml

	General population	Savanta GBMSM	Grindr	Meta
Yes, I have a dog or cat	1534 (50.3)	117 (47.4)	286 (34.4)	336 (32.4)
Yes, I have a rabbit, ferret, or rodent (e.g. rat, mouse, chinchilla, guinea pig)	195 (6.4)	7 (2.8)	7 (0.8)	10 (1.0)
Yes, I have another type of animal (e.g. reptile, fish, bird)	232 (7.6)	17 (6.9)	43 (5.2)	51 (4.9)
No, I don't have a pet [EXCLUSIVE]	1311 (43.0)	118 (47.8)	516 (62.1)	667 (64.4)

†Participants could select more than one item for this question, therefore column totals add to more than 100%.

New screen

ASK ALL

D6 What is your employment status?

SINGLE CODE

	General population	Savanta GBMSM	Grindr	Meta
Full time paid job (31+ hours)	1163 (38.1)	120 (48.6)	529 (63.7)	626 (60.4)
Part time paid job (<31 hours)	471 (15.4)	21 (8.5)	58 (7.0)	90 (8.7)
Doing paid work on a self-employed basis or within your own business	102 (3.3)	9 (3.6)	82 (9.9)	115 (11.1)
Student / On a government training programme (Nation Traineeship/Modern Apprenticeship)	93 (3.0)	7 (2.8)	35 (4.2)	32 (3.1)
Out of work	120 (3.9)	7 (2.8)	31 (3.7)	19 (1.8)
Looking after home / Homemaker	184 (6.0)	4 (1.6)	3 (0.4)	5 (0.5)
Retired	707 (23.2)	53 (21.5)	57 (6.9)	106 (10.2)
Disabled OR Long-term sick	155 (5.1)	21 (8.5)	25 (3.0)	28 (2.7)
Unpaid work for a business, community or voluntary organisation	27 (0.9)	1 (0.4)	6 (0.7)	11 (1.1)
Prefer not to say	28 (0.9)	4 (1.6)	5 (0.6)	4 (0.4)

New screen

ASK IF D6= CODES 1,2, 3, 4, or 9

For peer review only - http://bmjopen.bmj.com/site/about/guidelines.xhtml

D6A Are you a **frontline health or social care worker**? Please include any voluntary work

A frontline health or social care worker is someone who is directly involved in the care of patients or residents in long-stay care facilities (e.g. a care home), who has face-to-face contact with patients or clients, or who works in a laboratory, pathology or mortuary.

SINGLE CODE
FIX ORDER

	General population, n=1856	Savanta GBMSM, n=158	Grindr, n=710	Meta, n=874
Yes	355 (19.1)	23 (14.6)	94 (13.2)	125 (14.3)
No	1474 (79.4)	135 (85.4)	613 (86.3)	747 (85.5)
Prefer not to say	27 (1.5)	0 (0.0)	3 (0.4)	2 (0.2)

‡This question was only asked to those who reported that they were in full-time, part-time, self-employment, a student, or did unpaid or voluntary work, therefore total *n*s are different.

New screen

ASK IF D6= CODES 1, 2 OR 3
D6B If you needed to, are you able to work from home?
SINGLE CODE

	General population, n=1736	Savanta GBMSM, n=150	Grindr, n=669	Meta, n=831
Yes – full time	632 (36.4)	52 (34.7)	209 (31.2)	294 (35.4)
Yes – most of the time	260 (15.0)	26 (17.3)	136 (20.3)	196 (23.6)
Yes – but I have to go into my place of work once or twice a week	142 (8.2)	13 (8.7)	67 (10.0)	111 (13.4)
No – I cannot do my job from home	702 (40.4)	59 (39.3)	257 (38.4)	230 (27.7)

‡This question was only asked to those who reported that they were in full-time, part-time, or self-employment, therefore total *n*s are different.

New screen

ASK ALL
D7 What is the highest level of educational qualification you have received?
SINGLE CODE

	General population	Savanta GBMSM	Grindr	Meta
PhD/Doctorate	43 (1.4)	7 (2.8)	41 (4.9)	79 (7.6)

For peer review only - http://bmjopen.bmj.com/site/about/guidelines.xhtml

Masters	213 (7.0)	22 (8.9)	204 (24.5)	325 (31.4)
Bachelor's Degree or equivalent (Such as a NVQ level 5)	729 (23.9)	59 (23.9)	291 (35.0)	379 (36.6)
Higher education (Such as a HND or a NVQ level 4)	348 (11.4)	29 (11.7)	106 (12.8)	89 (8.6)
A level or equivalent (Such as Scottish Highers or NVQ level 3)	666 (21.8)	58 (23.5)	110 (13.2)	88 (8.5)
GCSE and below (Such as O level or an RSA Diploma)	747 (24.5)	53 (21.5)	57 (6.9)	63 (6.1)
Other qualifications (Such as NVQ level 1)	119 (3.9)	12 (4.9)	11 (1.3)	11 (1.1)
No qualifications	185 (6.1)	7 (2.8)	11 (1.3)	2 (0.2)

New screen

ASK ALL

D8 Which of the following categories would best describe your ethnicity?

SINGLE CODE

	General population	Savanta GBMSM	Grindr	Meta
White				
British/English/Welsh/Scottish/Northern Irish	2649 (86.9)	214 (86.6)	629 (75.7)	799 (77.1)
Irish	38 (1.2)	5 (2.0)	26 (3.1)	31 (3.0)
Gypsy, Traveller or Irish Traveller	7 (0.2)	0 (0.0)	1 (0.1)	1 (0.1)
Any other White background	71 (2.3)	13 (5.3)	90 (10.8)	131 (12.6)
Mixed/ Multiple ethnic groups				
White and Black Caribbean	18 (0.6)	1 (0.4)	7 (0.8)	4 (0.4)
White and Black African	11 (0.4)	4 (1.6)	1 (0.1)	3 (0.3)
White and Asian	24 (0.8)	1 (0.4)	3 (0.4)	9 (0.9)
Any other Mixed/ Multiple ethnic background	16 (0.5)	1 (0.4)	12 (1.4)	17 (1.6)
Asian or Asian British				
Indian	41 (1.3)	4 (1.6)	20 (2.4)	1 (0.1)
Pakistani	50 (1.6)	0 (0.0)	2 (0.2)	0 (0.0)
Bangladeshi	12 (0.4)	0 (0.0)	1 (0.1)	1 (0.1)
Chinese	14 (0.5)	1 (0.4)	7 (0.8)	7 (0.7)
Any other Asian background	12 (0.4)	0 (0.0)	4 (0.5)	5 (0.5)
Black or Black British				

For peer review only - http://bmjopen.bmj.com/site/about/guidelines.xhtml

African	26 (0.9)	1 (0.4)	5 (0.6)	2 (0.2)
Caribbean	32 (1.0)	0 (0.0)	8 (1.0)	4 (0.4)
Any other Black/ African/ Caribbean background	3 (0.1)	1 (0.4)	1 (0.1)	1 (0.1)
Other ethnic group				
Arab	7 (0.2)	0 (0.0)	3 (0.4)	1 (0.1)
Other	9 (0.3)	1 (0.4)	6 (0.7)	8 (0.8)
Don't know / Prefer not to say				
Don't know	2 (0.1)	0 (0.0)	0 (0.0)	1 (0.1)
Prefer not to say	8 (0.3)	0 (0.0)	5 (0.6)	10 (1.0)

New screen

ASK ALL
D9 Do you, or anyone else in your household have any long-standing illness, disability or infirmity?
Please select all that apply
MULTICODE

	General population	Savanta GBMSM	Grindr	Meta
Yes, I do	843 (27.6)	80 (32.4)	212 (25.5)	305 (29.4)
Yes, another household member does	391 (12.8)	21 (8.5)	88 (10.6)	115 (11.1)
No	1879 (61.6)	149 (60.3)	538 (64.7)	634 (61.2)
Prefer not to say	62 (2)	4 (1.6)	10 (1.2)	14 (1.4)

†Participants could select more than one item for this question, therefore column totals add to more than 100%.

New screen

ASK IF D9=1 (Yes, I do)
D9A Do you have any of the following health conditions?
Please select all that apply
MULTICODE
FIX ORDER

	General population, n=843	Savanta GBMSM, n=80	Grindr, n=212	Meta, n=305
Diabetes	184 (21.8)	21 (26.3)	38 (17.9)	49 (16.1)
Any type of cancer	57 (6.8)	4 (5.0)	10 (4.7)	14 (4.6)
Heart conditions	116 (13.8)	8 (10.0)	24 (11.3)	42 (13.8)
Respiratory conditions	191 (22.7)	15 (18.8)	27 (12.7)	59 (19.3)

For peer review only - http://bmjopen.bmj.com/site/about/guidelines.xhtml

(e.g. chronic obstructive pulmonary disease [COPD], asthma)				
Mental health	317 (37.6)	34 (42.5)	84 (39.6)	102 (33.4)
A skin condition (e.g. atopic dermatitis / eczema)	114 (13.5)	9 (11.3)	25 (11.8)	46 (15.1)
A condition that makes you much more likely to get infections (e.g. SCID, homozygous sickle cell)	20 (2.4)	0 (0.0)	7 (3.3)	7 (2.3)
A weakened immune system	110 (13)	8 (10.0)	24 (11.3)	38 (12.5)
Are taking medicine that weakens your immune system (e.g. steroid tablets, chemotherapy, or antiretroviral medications)	62 (7.4)	4 (5.0)	15 (7.1)	24 (7.9)
HIV/AIDS	6 (0.7)	12 (15.0)	52 (24.5)	117 (38.4)
Other, please specify	183 (21.7)	11 (13.8)	48 (22.6)	60 (19.7)
Prefer not to say	46 (5.5)	5 (6.3)	11 (5.2)	8 (2.6)

†Participants could select more than one item for this question, therefore column totals add to more than 100%.

‡This question was only asked to those who reported that had a chronic illness, therefore total *n*s are different.

New screen

ASK ALL

D9B Are you pregnant?

SINGLE CODE

	General population	Savanta GBMSM	Grindr	Meta
Yes	38 (1.2)	1 (0.4)	0 (0.0)	0 (0.0)
No	3001 (98.4)	245 (99.2)	830 (99.9)	1035 (99.9)
Prefer not to say	11 (0.4)	1 (0.4)	1 (0.1)	1 (0.1)

New screen

ASK ALL

D9C Have you ever taken PrEP (pre-exposure prophylaxis) for HIV?

SINGLE CODE

For peer review only - http://bmjopen.bmj.com/site/about/guidelines.xhtml

	General population	Savanta GBMSM	Grindr	Meta
Yes	42 (1.4)	28 (11.3)	402 (48.4)	487 (47.0)
No	2993 (98.1)	216 (87.4)	427 (51.4)	547 (52.8)
Prefer not to say	15 (0.5)	3 (1.2)	2 (0.2)	2 (0.2)

New screen

ASK ALL

D10 To the best of your knowledge, have you received a vaccine for…

Hepatitis A

	General population	Savanta GBMSM	Grindr	Meta
Yes	531 (17.4)	87 (35.2)	549 (66.1)	773 (74.6)
No. I was offered the vaccine, but didn't want to have it	128 (4.2)	12 (4.9)	7 (0.8)	4 (0.4)
No, I haven't been offered the vaccine	1579 (51.8)	94 (38.1)	129 (15.5)	125 (12.1)
Don't know / can't remember	812 (26.6)	54 (21.9)	146 (17.6)	134 (12.9)

Smallpox (before 2022)

	General population	Savanta GBMSM	Grindr	Meta
Yes	512 (16.8)	51 (20.6)	144 (17.3)	218 (21)
No. I was offered the vaccine, but didn't want to have it	117 (3.8)	12 (4.9)	5 (0.6)	7 (0.7)
No, I haven't been offered the vaccine	1666 (54.6)	133 (53.8)	477 (57.4)	595 (57.4)
Don't know / can't remember	755 (24.8)	51 (20.6)	205 (24.7)	216 (20.8)

Covid-19 (two doses or more)

	General population	Savanta GBMSM	Grindr	Meta
Yes	2557 (83.8)	218 (88.3)	796 (95.8)	1023 (98.7)
No. I was offered the vaccine, but didn't want to have it	242 (7.9)	16 (6.5)	27 (3.2)	11 (1.1)
No, I haven't been offered the vaccine	189 (6.2)	9 (3.6)	5 (0.6)	0 (0.0)
Don't know / can't remember	62 (2.0)	4 (1.6)	3 (0.4)	2 (0.2)

New screen

For peer review only - http://bmjopen.bmj.com/site/about/guidelines.xhtml

ASK ALL
D11 What is your marital status?
SINGLE CODE

	General population	Savanta GBMSM	Grindr	Meta
Single, never married	741 (24.3)	123 (49.8)	470 (56.6)	402 (38.8)
Married / civil partnership	1382 (45.3)	62 (25.1)	149 (17.9)	290 (28)
Separated	69 (2.3)	3 (1.2)	29 (3.5)	21 (2.0)
Divorced	319 (10.5)	14 (5.7)	57 (6.9)	39 (3.8)
Widowed	136 (4.5)	1 (0.4)	5 (0.6)	24 (2.3)
Partnered / in a relationship	393 (12.9)	43 (17.4)	111 (13.4)	251 (24.2)
Prefer not to say	10 (0.3)	1 (0.4)	10 (1.2)	9 (0.9)

New screen

The following questions are sensitive. You don't need to answer if you don't want to. If you don't want to answer, please select "prefer not to say".

New screen

ASK ALL
D12A How many **male** sexual partners have you had…?

By "sexual partner" we mean any form of genital contact, including touching, kissing, and intercourse.

Please give an approximate number if you are unsure. If you have not had any male sexual partners in this time, please put 0.
Type your answer below

		General population	Savanta GBMSM	Grindr	Meta
In the last 3 weeks (21 days)	OPEN TEXT ENTRY – number [cap at 100]	N=2418, M=0.6, SD=3.6, range 0 to 100.	N=210, M=1.1, SD=2.0, range 0 to 14.	N=787, M=2.9, SD=5.2, range 0 to 75.	N=982, M=2.5, SD=4.7, range 0 to 100.
In the last 3 months (90 days)	OPEN TEXT ENTRY – number	N=2418, M=0.8, SD=8.9, range 0 to 400.	N=210, M=2.8, SD=7.0, range 0 to 50.	N=787, M=9.1, SD=23.8, range 0 to 400.	N=982, M=8.9, SD=26.2, range 0 to 400.

For peer review only - http://bmjopen.bmj.com/site/about/guidelines.xhtml

	[cap at 400]				
Prefer not to say		632 (20.7)	37 (15.0)	44 (5.3)	54 (5.2)

New screen

ASK ALL
D12B How many **female** sexual partners have you had…?

By "sexual partner" we mean any form of genital contact, including touching, kissing, and intercourse.

Please give an approximate number if you are unsure. If you have not had any female sexual partners in this time, please put 0.
Type your answer below

		General population	Savanta GBMSM	Grindr	Meta
In the last 3 weeks (21 days)	OPEN TEXT ENTRY – number [cap at 100]	N=2442, M=0.4, SD=3.1, range 0 to 100.	N=207, M=0.2, SD=0.8, range 0 to 100.	N=792, M=0.1, SD=0.6, range 0 to 10.	N=992, M=0.0, SD=0.4, range 0 to 9.
In the last 3 months (90 days)	OPEN TEXT ENTRY – number [cap at 400]	N=2442, M=0.8, SD=9.2, range 0 to 400.	N=207, M=2.2, SD=27.8, range 0 to 400.	N=792, M=0.2, SD=1.6, range 0 to 30.	N=992, M=0.1, SD=1.0, range 0 to 28.
Prefer not to say		608 (19.9)	40 (16.2)	39 (4.7)	44 (4.2)

New screen

Thanks for completing the survey. If you would like any more information on monkeypox, please see https://www.nhs.uk/conditions/monkeypox/.

Please press the Finish button to complete the survey.
Many thanks for completing this survey, your views are extremely important to us.

If you have any further comments or feedback, please use the box below.

For peer review only - http://bmjopen.bmj.com/site/about/guidelines.xhtml

Supplementary materials 2. Data preparation

Some participants (3.8%, n=196) did not provide their postcode and were therefore missing data about region and index of multiple deprivation. While individual items about financial hardship had small amounts of missing data (2.0% to 3.8%, n=105 to 197), as items were summed to give a scale, missingness on the resulting scale would have been higher (7.9%, n=410). There were no apparent patterns to the missing data. We used multiple imputation to impute missing values for region, index of multiple deprivation and financial hardship items. Socio-demographic variables (age, occupation of highest earner, number of people in household, having a dependent child, employment status, education, ethnicity, marital status, and region, index of multiple deprivation and financial hardship where available) were used to predict missing values using a Markov Chain Monte Carlo (MCMC) method using linear regressions with ten iterations, to give five imputations.

We computed a single variable to indicate perceived susceptibility and severity from three items. For these items, "don't know" was coded as the mid-point on the scale. We then multiplied responses together (range 1 to 125) and took the cubic root to give a scale from 1 (lowest perceived susceptibility and severity) to 5 (highest perceived susceptibility and severity). We also asked participants how much they agreed that they were already immune to mpox. This item was recoded into a binary item to show perceived immunity to mpox (strongly agree and agree, vs neither agree nor disagree, disagree, strongly disagree, and don't know).

To create a single variable for perceived knowledge, we used three items (Cronbach's α=0.74). Responses for these items were given on a five-point scale from "strongly disagree" to "strongly agree" We recoded each item as a binary item (strongly agree and agree [1], vs neither agree nor disagree, disagree, strongly disagree, and don't know [0]), then summed responses to give a scale from 0 (lowest perceived knowledge) to 3 (highest perceived knowledge).

For knowledge about mpox symptoms, for each symptom selected, we coded participants as being correct (1, symptom listed on NHS mpox website) or incorrect (0, symptom not listed on NHS mpox website). Scores were then summed to give a scale from 0 (no selected symptoms were mpox symptoms) to 4 (all selected symptoms were mpox symptoms); responses of "don't know" were coded as 0. A single variable denoting understanding of transmission was computed. It is unknown whether mpox can be caught from pet animals

For peer review only - http://bmjopen.bmj.com/site/about/guidelines.xhtml

(but pet animals can catch mpox from humans (1)), therefore we did not include this item in analyses. We coded answers as correct (1) or incorrect (0; answers of "don't know" coded as incorrect), and summed items to give a score from 0 (lowest knowledge about transmission) to 6 (highest knowledge about transmission).

To reduce the number of variables included in regression models, we used dimension reduction techniques. We conducted principal components analysis, using direct oblimin rotation as items may have been correlated, on items potentially associated with self-isolation (ten items), help seeking (six items), and vaccination (eight items) separately. The number of factors was determined using a scree plot. The item with the highest loading on to a component was then included in regression analyses. Results of the principal components analyses are reported in the supplementary materials 3.

References

1. Seang S, Burrel S, Todesco E, Leducq V, Monsel G, Le Pluart D, et al. Evidence of human-to-dog transmission of monkeypox virus. The Lancet. 2022;400(10353):658-9.

For peer review only

Supplementary materials 3. Results of principal components analyses.

Self-isolation

A scree plot suggested three components underlying factors potentially associated with self-isolation. Table 1 shows loadings of items onto each component.

Table 1. Loadings of items measuring factors potentially associated with self-isolation onto components identified (only loadings over ± .4 are shown).

Item	Component 1 loading	Component 2 loading	Component 3 loading
If I had mpox symptoms, I wouldn't want to tell anyone as I don't want to self-isolate*		-.864	
If I had mpox symptoms, I wouldn't want to tell anyone so that others don't have to self-isolate		-.853	
Most people would self-isolate if they were told to*			.810
I have the support I need to self-isolate for 21 days			.729
An effective way to prevent the spread of mpox is for people who have tested positive to self-isolate		.544	.426
If I had to self-isolate because I had tested positive for mpox...I would lose touch with my friends and relatives	.627		
If I had to self-isolate because I had tested positive for mpox...it would have a severe impact on my family's wellbeing	.684		
If I had to self-isolate because I had tested positive for mpox...it would have a negative impact on how much money I have	.754		
If I had to self-isolate because I had tested positive for mpox...it would have a negative impact on my work*	.789		
If I had to self-isolate because I had tested positive for mpox...I would miss out on events and activities that I want to attend	.646		

Rotation method: oblimin with Kaiser normalization. Rotation converged in 10 iterations.
*Included in regression analyses

Help seeking

A scree plot suggested two components underlying factors potentially associated with seeking help immediately. Table 2 shows loadings of items onto each component.

Table 2. Loadings of items measuring factors potentially associated with self-isolation onto components identified (only loadings over ± .4 are shown).

Item	Component 1 loading	Component 2 loading
I wouldn't want to know the results of a mpox test		-.526
I would be worried what my friends or family would think about me if they thought I had mpox *	.867	
I would be worried about how colleagues / my employer would react if they thought I had mpox	.845	
I don't want to have a mpox test result on my medical record	.727	
An effective way to prevent the spread of mpox is for people who have symptoms to contact healthcare services		.772
I would be willing to contact a sexual health clinic if I thought I had mpox symptoms or had come into contact with someone who had mpox *		.784

Rotation method: oblimin with Kaiser normalization. Rotation converged in 2 iterations.
*Included in regression analyses

For peer review only - http://bmjopen.bmj.com/site/about/guidelines.xhtml

Vaccination

A scree plot suggested two components underlying factors potentially associated with vaccination. Table 3 shows loadings of items onto each component.

Table 3. Loadings of items measuring factors potentially associated with vaccination onto components identified (only loadings over ± .4 are shown).

Item	Component 1 loading	Component 2 loading
In general, vaccination is a good thing		.627
Most people like me will get a smallpox vaccination if advised		.737
If I get a smallpox vaccination, I will be protected against mpox *		.746
A smallpox vaccination could give me smallpox	.801	
I might regret getting the smallpox vaccination if I later experienced side effects from it	.789	
I would be worried about experiencing side effects from a smallpox vaccination	.793	
I would be worried that having a smallpox vaccine might make me infectious to others*	.843	
People who are likely to come into high-risk contact with mpox should have a smallpox vaccine		.744

Rotation method: oblimin with Kaiser normalization. Rotation converged in 4 iterations.
*Included in regression analyses

For peer review only

For peer review only - http://bmjopen.bmj.com/site/about/guidelines.xhtml

Supplementary materials 4. Influence of motivational messages on behavioural outcomes.

Table 1. Influence of motivational messaging on behavioural outcomes, in the general population sample.

			General population, n (%)				
			Risk + perceived necessity/efficacy, n=762	Risk + perceived benefits, n=768	Risk + perceived costs, n=762	Control, n=758	p-value
**Self-isolation for 21 days**	Sum of intention if case and high-risk contact	2 (lowest intention) to 10 (highest intention)	M=8.0, SD=2.2	M=7.9, SD=2.2	M=7.8, SD=2.2	M=8.1, SD=2.1	0.07
**Help seeking**	Any action that involves contacting a healthcare professional (by phone or in person)	Would not seek help or would seek help, but would "wait and see" for a day or two to see if symptoms resolved	362 (47.5)	413 (53.8)	382 (50.1)	391 (51.6)	0.10
		Would seek help immediately	400 (52.5)	355 (46.2)	380 (49.9)	367 (48.4)	
**Contact behaviour**	Have sexual contact with others†	Less than, same as, or more than normal	125 (20.5)	146 (22.6)	148 (23.8)	148 (24.2)	0.41
		Completely stop	485 (79.5)	501 (77.4)	473 (76.2)	464 (75.8)	
**Contact sharing**	Sexual contacts	1 (definitely would not) to 5 (definitely would)	N=748, M=4.2, SD=1.2	N=756, M=4.2, SD=1.2	N=746, M=4.2, SD=1.1	N=747, M=4.1, SD=1.1	0.98
**Vaccination intention**	Smallpox vaccine "if advised"	1 (definitely would not) to 5 (definitely would)	N=723, M=4.0, SD=1.1	N=726, M=4.0, SD=1.1	N=721, M=4.0, SD=1.1	N=718, M=4.0, SD=1.1	0.81

†Answers of "don't know", "prefer not to say" and "not applicable, I wouldn't do this anyway" were coded as missing, therefore total *n* was substantially lower (n=2490).

For peer review only

Table 2. Influence of motivational messaging on behavioural outcomes, by GBMSM sample.

			Savanta GBMSM, n (%)			Grindr, n (%)			Meta, n (%)		
			Motivational message, n=115	Control, n=132	p-value	Motivational message, n=404	Control, n=427	p-value	Motivational message, n=529	Control, n=507	p-value
**Self-isolation for 21 days**	2 (lowest intention) to 10 (highest intention)		M=7.7, SD=2.4	M=8.0, SD=2.1	0.22	M=7.2, SD=2.4	M=7.3, SD=2.5	0.74	M=7.1, SD=2.3	M=7.5, SD=2.2	0.005
**Help seeking**	Any action that involves contacting a healthcare professional (by phone or in person)	Would not seek help or would seek help, but would "wait and see" for a day or two to see if symptoms resolved	59 (51.3)	66 (50.0)	0.84	189 (46.8)	199 (46.6)	0.96	187 (35.3)	204 (40.2)	0.10
		Would seek help immediately	56 (48.7)	66 (50.0)		215 (53.2)	228 (53.4)		342 (64.7)	303 (59.8)	
**Contact behaviour**	Have sexual contact with others†	Less than, same as, or more than normal	26 (26.5)	19 (16.1)	0.06	28 (7.3)	41 (10.1)	0.17	38 (7.5)	31 (6.4)	0.50
		Completely stop	72 (73.5)	99 (83.9)		355 (92.7)	366 (89.9)		469 (92.5)	453 (93.6)	
**Contact sharing**	Sexual contacts	1 (definitely would not) to 5 (definitely would)	N=113, M=4.0, SD=1.2	N=130, M=4.1, SD=1.2	0.54	N=402, M=4.1, SD=1.2	N=421, M=4.1, SD=1.2	0.72	N=526, M=4.1, SD=1.2	N=505, M=4.2, SD=1.1	0.04
**Vaccination intention**	Smallpox vaccine "if advised"‡	1 (definitely would not) to 5 (definitely would)	N=103, M=4.3, SD=1.0	N=118, M=4.2, SD=1.0	0.36	N=282, M=4.6, SD=0.9	N=284, M=4.5, SD=1.0	0.18	N=304, M=4.7, SD=0.9	N=310, M=4.8, SD=0.6	0.03

†Answers of "don't know", "prefer not to say" and "not applicable, I wouldn't do this anyway" were coded as missing, therefore total *n*s were substantially lower (Savanta GBMSM, n=216; Grindr, n=790; Meta, n=991).
‡Not asked to people who reported that they had had the smallpox vaccine in 2022, therefore total *n*s were substantially lower (Savanta GBMSM, n=221; Grindr, n=566; Meta, n=614).

For peer review only - http://bmjopen.bmj.com/site/about/guidelines.xhtml

Supplementary materials 5. Full results of regressions with intention to seek help immediately.

Table 1. Associations between intending to seek help immediately and socio-demographic characteristics and motivational message, by sample. Variables were entered into the logistic regression model in blocks (block 1: socio-demographic variables and motivational message, block 2: psychological factors, block 3: help-seeking specific factors). Results for block 3, using pooled estimates are reported.

Participant characteristics	Level	General population		Grindr	
		aOR (95% CI)	*p*-value	aOR (95% CI)	*p*-value
Gender	Male (including trans man)	Ref	-	-	-
	Female (including trans woman)	1.10 (0.93 to 1.30)	0.25	-	-
Sexual orientation	Straight or heterosexual	Ref	-	-	-
	Gay, lesbian, bisexual, or queer	1.15 (0.84 to 1.58)	0.39	-	-
Age	Range 18 to 98 years	1.012 (1.006 to 1.019)	<0.001*	1.02 (1.01 to 1.04)	0.006
	Quadratic term, (age – mean)^2	1.0002 (0.9999 to 1.0005)	0.24	1.0001 (0.9992 to 1.0011)	0.80
Region	Midlands (East and West)	Ref	-	Ref	-
	South (East, West, East of England)	1.10 (0.87 to 1.40)	0.40	1.01 (0.50 to 2.04)	0.97
	North (East, West, Yorkshire and the Humber)	0.90 (0.71 to 1.15)	0.41	1.53 (0.75 to 3.13)	0.24
	London	0.90 (0.65 to 1.25)	0.54	1.15 (0.59 to 2.26)	0.67
	Devolved nations (Scotland, Wales, and Northern Ireland)	0.83 (0.62 to 1.13)	0.24	1.04 (0.47 to 2.28)	0.93
Dependent child in household	No	Ref	-	Ref	-
	Yes	0.96 (0.78 to 1.17)	0.68	1.58 (0.71 to 3.51)	0.26
Employment status	Not working	Ref	-	Ref	-
	Working	1.12 (0.87 to 1.43)	0.39	0.98 (0.57 to 1.68)	0.94
Frontline health or social care worker	No	Ref	-	Ref	-
	Yes	1.05 (0.81 to 1.36)	0.73	1.91 (1.15 to 3.18)	0.01
Need to leave home for work	Do not need to leave home for work	Ref	-	Ref	-
	Need to leave home for work	1.05 (0.84 to 1.31)	0.65	0.94 (0.64 to 1.37)	0.75
Education	GCSE/vocational/A-level/No formal qualifications	Ref	-	Ref	-
	Degree or higher (Bachelors, Masters, PhD)	0.87 (0.72 to 1.04)	0.12	0.94 (0.66 to 1.34)	0.74
Ethnicity	White British	Ref	-	Ref	-
	White other	1.26 (0.82 to 1.92)	0.29	1.03 (0.65 to 1.65)	0.89
	Black, Asian, other minoritized ethnicity	0.90 (0.66 to 1.22)	0.48	0.95 (0.55 to 1.63)	0.85
Marital status	Not partnered	Ref	-	Ref	-
	Partnered	1.09 (0.87 to 1.36)	0.44	0.81 (0.53 to 1.22)	0.31
Live alone	Live with someone else	Ref	-	Ref	-
	Live alone	1.10 (0.85 to 1.41)	0.48	0.79 (0.55 to 1.15)	0.23
Own chronic illness	None	Ref	-	Ref	-
	Present	1.11 (0.92 to 1.34)	0.28	1.28 (0.88 to 1.88)	0.20
Ever taken PrEP for HIV	No	-	-	Ref	-
	Yes	-	-	0.89 (0.62 to 1.28)	0.54
Vaccinated for smallpox in 2022	Not vaccinated	-	-	Ref	-
	Vaccinated	-	-	0.85 (0.57 to 1.27)	0.43
Index of multiple	Deciles (1st = most deprived, 10th	1.00 (0.97 to 1.03)	0.93	1.05 (0.98 to 1.13)	0.16

For peer review only

deprivation	= least deprived)				
Socio-economic grade	ABC1	Ref	-	Ref	-
	C2DE	0.94 (0.79 to 1.13)	0.53	0.66 (0.41 to 1.07)	0.09
Financial hardship	4 (lowest hardship) to 13 (most hardship)	0.96 (0.92 to 1.00)	0.07	0.99 (0.90 to 1.09)	0.87
Total number of sexual partners (male and female) in last three weeks	0	Ref	-	-	-
	1	1.09 (0.88 to 1.34)	0.45	-	-
	2 to 4	1.09 (0.73 to 1.62)	0.68	-	-
	5 or more	0.78 (0.38 to 1.64)	0.52	-	-
	Prefer not to say	1.09 (0.86 to 1.37)	0.49	-	-
Number of male sexual partners in last three weeks	0	-	-	Ref	-
	1	-	-	1.01 (0.63 to 1.62)	0.95
	2 to 4	-	-	1.01 (0.66 to 1.56)	0.95
	5 to 9	-	-	0.84 (0.47 to 1.49)	0.55
	10 or more	-	-	1.06 (0.51 to 2.17)	0.88
	Prefer not to say	-	-	0.93 (0.43 to 2.03)	0.86
Motivational message	Perceived risk of illness and necessity and efficacy of the response	1.16 (0.93 to 1.44)	0.19	-	-
	Perceived risk of illness and benefits of the response	0.96 (0.77 to 1.20)	0.71	-	-
	Perceived risk of illness and low perceived costs of response	1.05 (0.84 to 1.31)	0.68	-	-
	Control	Ref	-	-	-
Motivational message	All motivational components	-	-	0.86 (0.63 to 1.17)	0.34
	Control	-	-	Ref	-

*$p \leq 0.001$

For peer review only

Table 2. Associations between intending to seek help immediately and psychological and contextual factors, by sample. Variables were entered into the logistic regression model in blocks (block 1: socio-demographic variables and motivational message, block 2: psychological factors, block 3: help-seeking specific factors). Results for block 3, using pooled estimates are reported.

Factor	Level	General population aOR (95% CI)	*p*-value	Grindr aOR (95% CI)	*p*-value
Amount heard about mpox	I have not seen or heard anything (1) to I have seen or heard a lot (3)	1.05 (0.87 to 1.25)	0.63	0.96 (0.67 to 1.37)	0.83
Worry about mpox	Not at all worried (1) to extremely worried (4)	1.05 (0.90 to 1.23)	0.52	1.17 (0.84 to 1.63)	0.36
Perceived risk of mpox to oneself	No risk at all (1) to very high risk (5)	0.85 (0.74 to 0.98)	0.02	0.90 (0.72 to 1.14)	0.38
Perceived risk of mpox to people in UK	No risk at all (1) to very high risk (5)	1.04 (0.90 to 1.19)	0.62	1.37 (1.07 to 1.76)	0.01
Perceived susceptibility and severity	Lowest (1) to highest (5)	1.07 (0.94 to 1.23)	0.32	1.03 (0.77 to 1.36)	0.86
I am already immune to mpox	Strongly disagree, disagree, neither agree nor disagree, don't know	Ref	-	Ref	-
	Strongly agree and agree	0.95 (0.72 to 1.26)	0.73	0.69 (0.40 to 1.19)	0.18
People who catch mpox usually make a full recovery, even if they do not receive any treatment	Strongly disagree (1) to strongly agree (5)	0.87 (0.78 to 0.96)	0.006	0.84 (0.69 to 1.03)	0.09
My personal behaviour has an impact on how mpox spreads	Strongly disagree (1) to strongly agree (5)	1.05 (0.98 to 1.13)	0.19	1.07 (0.92 to 1.25)	0.37
My life has been negatively affected by changes made in response to the mpox outbreak	Strongly disagree (1) to strongly agree (5)	1.00 (0.91 to 1.10)	0.99	1.00 (0.85 to 1.17)	0.99
The risks of mpox are being exaggerated	Strongly disagree (1) to strongly agree (5)	0.83 (0.76 to 0.91)	<0.001*	0.94 (0.77 to 1.13)	0.48
Mpox is only a risk to men who are gay, bisexual or have sex with men	Strongly disagree (1) to strongly agree (5)	0.95 (0.88 to 1.03)	0.22	0.85 (0.72 to 1.01)	0.06
Perceived knowledge	Lowest (0) to highest (3)	0.97 (0.89 to 1.06)	0.54	0.99 (0.80 to 1.23)	0.93
Knowledge of mpox symptoms	Identified no symptoms (0) to identified four symptoms (4)	1.03 (0.96 to 1.09)	0.44	1.13 (0.98 to 1.30)	0.09
Knowledge of mpox transmission	Lowest (0) to highest (6)	0.95 (0.90 to 1.01)	0.12	1.03 (0.89 to 1.20)	0.69
I would be worried what my friends or family would think about me if they thought I had mpox	Strongly disagree (1) to strongly agree (5)	0.94 (0.88 to 1.00)	0.06	0.85 (0.75 to 0.97)	0.01
I would be willing to contact a sexual health clinic if I thought I had mpox symptoms or had come into contact with someone who had mpox	Strongly disagree (1) to strongly agree (5)	1.25 (1.16 to 1.34)	<.001*	1.57 (1.29 to 1.91)	<.001*

*$p \leq 0.001$

Supplementary materials 6. Full results of regressions with smallpox vaccination.

Table 1. Associations between vaccine uptake (general population sample: actual and intended, GBMSM: actual, actual and intended) and socio-demographic characteristics and motivational message, by sample. Variables were entered into the logistic regression model in blocks (block 1: socio-demographic variables and motivational message, block 2: psychological factors, block 3: vaccination specific factors). Results for block 3, using pooled estimates are reported.

Participant characteristics	Level	General population		Grindr			
		Vaccinated in 2022 or would be vaccinated if advised		Vaccinated in 2022		Vaccinated in 2022 or would be vaccinated if advised	
		aOR (95% CI)	*p*-value	aOR (95% CI)	*p*-value	aOR (95% CI)	*p*-value
Gender	Male (including trans man)	Ref	-	-	-	-	-
	Female (including trans woman)	0.93 (0.75 to 1.13)	0.46	-	-	-	-
Sexual orientation	Straight or heterosexual	Ref	-	-	-	-	-
	Gay, lesbian, bisexual, or queer	1.26 (0.85 to 1.88)	0.26	-	-	-	-
Age	Range 18 to 98 years	1.015 (1.007 to 1.023)	<0.001*	1.00 (0.98 to 1.02)	0.81	0.98 (0.94 to 1.02)	0.30
	Quadratic term, $(age – mean)^2$	1.0007 (1.0002 to 1.0011)	0.002	1.000 (0.998 to 1.001)	0.48	0.999 (0.997 to 1.001)	0.29
Region	Midlands (East and West)	Ref	-	Ref	-	Ref	-
	South (East, West, East of England)	0.82 (0.61 to 1.11)	0.20	0.93 (0.43 to 2.03)	0.86	1.05 (0.22 to 5.12)	0.95
	North (East, West, Yorkshire and the Humber)	0.77 (0.57 to 1.04)	0.09	1.21 (0.51 to 2.87)	0.67	0.48 (0.09 to 2.61)	0.40
	London	0.79 (0.54 to 1.17)	0.24	2.30 (1.04 to 5.11)	0.04	0.68 (0.13 to 3.64)	0.65
	Devolved nations (Scotland, Wales, and Northern Ireland)	0.64 (0.44 to 0.93)	0.02	0.82 (0.31 to 2.13)	0.68	1.91 (0.23 to 16.11)	0.55
Dependent child in household	No	Ref	-	Ref	-	Ref	-
	Yes	0.85 (0.67 to 1.08)	0.19	1.35 (0.49 to 3.71)	0.56	1.11 (0.09 to 13.72)	0.94
Employment status	Not working	Ref	-	Ref	-	Ref	-
	Working	1.34 (0.98 to 1.83)	0.07	1.22 (0.61 to 2.45)	0.57	2.06 (0.42 to 10.12)	0.37
Frontline health or social care worker	No	Ref	-	Ref	-	Ref	-
	Yes	1.46 (1.07 to 1.99)	0.02	0.41 (0.20 to 0.81)	0.01	5.38 (0.90 to 32.19)	0.07
Need to leave home for work	Do not need to leave home for work	Ref	-	Ref	-	Ref	-
	Need to leave home for work	0.56 (0.43 to 0.73)	<0.001*	0.99 (0.61 to 1.60)	0.96	0.28 (0.08 to 1.06)	0.06
Education	GCSE/vocational/A-level/No formal qualifications	Ref	-	Ref	-	Ref	-
	Degree or higher (Bachelors, Masters, PhD)	1.18 (0.94 to 1.47)	0.16	0.78 (0.49 to 1.26)	0.31	1.56 (0.58 to 4.19)	0.38
Ethnicity	White British	Ref	-	Ref	-	Ref	-

For peer review only - http://bmjopen.bmj.com/site/about/guidelines.xhtml

	White other	0.53 (0.33 to 0.85)	0.009	1.27 (0.72 to 2.26)	0.40	0.59 (0.15 to 2.26)	0.44
	Black, Asian, other minoritized ethnicity	0.81 (0.57 to 1.15)	0.24	1.52 (0.78 to 2.99)	0.22	0.41 (0.10 to 1.69)	0.22
Marital status	Not partnered	Ref	-	Ref	-	Ref	-
	Partnered	1.02 (0.79 to 1.33)	0.86	0.74 (0.43 to 1.25)	0.26	2.35 (0.66 to 8.33)	0.19
Live alone	Live with someone else	Ref	-	Ref	-	Ref	-
	Live alone	0.94 (0.69 to 1.27)	0.68	1.30 (0.81 to 2.09)	0.28	1.09 (0.40 to 2.96)	0.86
Own chronic illness	None	Ref	-	Ref	-	Ref	-
	Present	1.07 (0.85 to 1.36)	0.57	1.67 (1.02 to 2.74)	0.04	1.12 (0.39 to 3.25)	0.83
Ever taken PrEP for HIV	No	-	-	Ref	-	Ref	-
	Yes	-	-	8.95 (5.61 to 14.28)	<0.001*	3.41 (1.11 to 10.46)	0.03
Index of multiple deprivation	Deciles (1st = most deprived, 10th = least deprived)	1.00 (0.96 to 1.04)	0.89	0.95 (0.86 to 1.05)	0.28	0.94 (0.78 to 1.13)	0.52
Socio-economic grade	ABC1	Ref	-	Ref	-	Ref	-
	C2DE	0.83 (0.67 to 1.02)	0.08	1.53 (0.82 to 2.87)	0.19	1.39 (0.41 to 4.67)	0.60
Financial hardship	4 (lowest hardship) to 13 (most hardship)	0.95 (0.90 to 0.99)	0.02	1.03 (0.91 to 1.18)	0.62	0.95 (0.73 to 1.24)	0.71
Total number of sexual partners (male and female) in last three weeks	0	Ref	-	-	-	-	-
	1	1.05 (0.80 to 1.37)	0.72	-	-	-	-
	2 to 4	1.33 (0.81 to 2.18)	0.26	-	-	-	-
	5 or more	1.62 (0.64 to 4.09)	0.30	-	-	-	-
	Prefer not to say	0.70 (0.54 to 0.93)	0.01	-	-	-	-
Number of male sexual partners in last three weeks	0	-	-	Ref	-	Ref	-
	1	-	-	1.33 (0.69 to 2.57)	0.40	1.12 (0.33 to 3.77)	0.85
	2 to 4	-	-	1.70 (0.96 to 3.03)	0.07	1.78 (0.56 to 5.65)	0.33
	5 to 9	-	-	2.40 (1.17 to 4.95)	0.02	2.14 (0.34 to 13.33)	0.42
	10 or more	-	-	1.53 (0.65 to 3.62)	0.33	3.80 (0.28 to 52.03)	0.32
	Prefer not to say	-	-	1.21 (0.39 to 3.73)	0.74	0.72 (0.15 to 3.47)	0.68
Motivational message	Perceived risk of illness and necessity and efficacy of the response	0.92 (0.70 to 1.20)	0.53	-	-	-	-
	Perceived risk of illness and benefits of the response	1.15 (0.87 to 1.50)	0.32	-	-	-	-
	Perceived risk of illness and low perceived costs of response	1.02 (0.78 to 1.34)	0.89	-	-	-	-
	Control	Ref	-	-	-	-	-
Motivational message	All motivational components	-	-	0.91 (0.61 to 1.35)	0.63	1.02 (0.43 to 2.39)	0.97
	Control	-	-	Ref	-	Ref	-

*$p \leq 0.001$

For peer review only - http://bmjopen.bmj.com/site/about/guidelines.xhtml

Table 2. Associations between vaccine uptake (general population sample: actual and intended, GBMSM: actual, actual and intended) and psychological and contextual factors, by sample. Variables were entered into the logistic regression model in blocks (block 1: socio-demographic variables and motivational message, block 2: psychological factors, block 3: vaccination specific factors). Results for block 3, using pooled estimates are reported.

Factor	Level	General population<br>Vaccinated in 2022 or would be vaccinated if advised		Grindr<br>Vaccinated in 2022		 Vaccinated in 2022 or would be vaccinated if advised	
		aOR (95% CI)	*p*-value	aOR (95% CI)	*p*-value	aOR (95% CI)	*p*-value
Amount heard about mpox	I have not seen or heard anything (1) to I have seen or heard a lot (3)	0.93 (0.75 to 1.15)	0.50	1.74 (1.09 to 2.78)	0.02	0.56 (0.21 to 1.55)	0.27
Worry about mpox	Not at all worried (1) to extremely worried (4)	1.49 (1.24 to 1.80)	<0.001*	1.00 (0.66 to 1.52)	0.99	4.33 (1.69 to 11.07)	0.002
Perceived risk of mpox to oneself	No risk at all (1) to very high risk (5)	0.99 (0.84 to 1.17)	0.92	1.25 (0.93 to 1.67)	0.13	1.19 (0.63 to 2.27)	0.59
Perceived risk of mpox to people in UK	No risk at all (1) to very high risk (5)	1.19 (1.00 to 1.40)	0.05	0.85 (0.62 to 1.16)	0.31	0.93 (0.43 to 2.00)	0.85
Perceived susceptibility and severity	Lowest (1) to highest (5)	1.31 (1.11 to 1.55)	0.001*	0.85 (0.60 to 1.21)	0.37	0.90 (0.40 to 2.05)	0.81
I am already immune to mpox	Strongly disagree, disagree, neither agree nor disagree, don't know	Ref	-	Ref	-	Ref	-
	Strongly agree and agree	1.30 (0.93 to 1.83)	0.12	8.83 (4.52 to 17.22)	<0.001*	1.30 (0.27 to 6.36)	0.74
People who catch mpox usually make a full recovery, even if they do not receive any treatment	Strongly disagree (1) to strongly agree (5)	1.01 (0.89 to 1.14)	0.90	1.20 (0.94 to 1.53)	0.15	0.51 (0.27 to 0.94)	0.03
My personal behaviour has an impact on how mpox spreads	Strongly disagree (1) to strongly agree (5)	1.00 (0.91 to 1.09)	0.99	0.99 (0.80 to 1.22)	0.92	0.94 (0.63 to 1.39)	0.74
My life has been negatively affected by changes made in response to the mpox outbreak	Strongly disagree (1) to strongly agree (5)	0.93 (0.84 to 1.04)	0.23	0.96 (0.79 to 1.17)	0.70	0.97 (0.63 to 1.51)	0.90
The risks of mpox are being exaggerated	Strongly disagree (1) to strongly agree (5)	0.76 (0.68 to 0.85)	<0.001*	0.78 (0.61 to 1.00)	0.05	0.45 (0.27 to 0.75)	0.002
Mpox is only a risk to men who are gay, bisexual or have sex with men	Strongly disagree (1) to strongly agree (5)	0.97 (0.88 to 1.07)	0.56	0.84 (0.67 to 1.04)	0.11	1.05 (0.66 to 1.65)	0.85
Perceived knowledge	Lowest (0) to highest (3)	1.03 (0.92 to 1.14)	0.64	1.25 (0.93 to 1.67)	0.14	0.78 (0.43 to 1.43)	0.42
Knowledge of mpox symptoms	Identified no symptoms (0) to identified four symptoms (4)	1.02 (0.94 to 1.10)	0.65	1.01 (0.83 to 1.21)	0.95	0.96 (0.67 to 1.38)	0.82
Knowledge of mpox transmission	Lowest (0) to highest (6)	1.04 (0.96 to 1.11)	0.34	0.98 (0.80 to 1.19)	0.82	1.05 (0.71 to 1.55)	0.82
If I get a smallpox vaccination, I will be protected against mpox	Strongly disagree (1) to strongly agree (5)	1.44 (1.29 to 1.61)	<0.001*	1.33 (1.06 to 1.66)	0.01	3.25 (1.97 to 5.35)	<0.001*

For peer review only - http://bmjopen.bmj.com/site/about/guidelines.xhtml

I would be worried that having a smallpox vaccine might make me infectious to others	Strongly disagree (1) to strongly agree (5)	0.64 (0.58 to 0.71)	<0.001*	0.63 (0.46 to 0.87)	0.005	0.44 (0.26 to 0.76)	0.003

*$p \leq 0.001$

For peer review only

STROBE 2007 (v4) Statement—Checklist of items that should be included in reports of *cross-sectional studies*

Section/Topic	Item #	Recommendation	Reported on page #
**Title and abstract**	1	(*a*) Indicate the study's design with a commonly used term in the title or the abstract	3
		(*b*) Provide in the abstract an informative and balanced summary of what was done and what was found	3
**Introduction**			
Background/rationale	2	Explain the scientific background and rationale for the investigation being reported	5-7
Objectives	3	State specific objectives, including any prespecified hypotheses	7
**Methods**			
Study design	4	Present key elements of study design early in the paper	7-8
Setting	5	Describe the setting, locations, and relevant dates, including periods of recruitment, exposure, follow-up, and data collection	7-8
Participants	6	(*a*) Give the eligibility criteria, and the sources and methods of selection of participants	7-8
Variables	7	Clearly define all outcomes, exposures, predictors, potential confounders, and effect modifiers. Give diagnostic criteria, if applicable	8-12, supplementary materials 1
Data sources/ measurement	8*	For each variable of interest, give sources of data and details of methods of assessment (measurement). Describe comparability of assessment methods if there is more than one group	8-12, supplementary materials 1
Bias	9	Describe any efforts to address potential sources of bias	7-14
Study size	10	Explain how the study size was arrived at	7-8, 12
Quantitative variables	11	Explain how quantitative variables were handled in the analyses. If applicable, describe which groupings were chosen and why	12-14, supplementary materials 2
Statistical methods	12	(*a*) Describe all statistical methods, including those used to control for confounding	12-14
		(*b*) Describe any methods used to examine subgroups and interactions	12-14
		(*c*) Explain how missing data were addressed	12-14, supplementary materials 2

For peer review only

		(*d*) If applicable, describe analytical methods taking account of sampling strategy	12-14
		(*e*) Describe any sensitivity analyses	N/A
**Results**			
Participants	13*	(a) Report numbers of individuals at each stage of study—eg numbers potentially eligible, examined for eligibility, confirmed eligible, included in the study, completing follow-up, and analysed	14-15
		(b) Give reasons for non-participation at each stage	N/A
		(c) Consider use of a flow diagram	Considered
Descriptive data	14*	(a) Give characteristics of study participants (eg demographic, clinical, social) and information on exposures and potential confounders	14-15
		(b) Indicate number of participants with missing data for each variable of interest	Supplementary materials 1 and 2
Outcome data	15*	Report numbers of outcome events or summary measures	16-17, 20, 23, 25, supplementary materials 4
Main results	16	(*a*) Give unadjusted estimates and, if applicable, confounder-adjusted estimates and their precision (eg, 95% confidence interval). Make clear which confounders were adjusted for and why they were included	15-26, supplementary materials 5 and 6
		(*b*) Report category boundaries when continuous variables were categorized	12-14, supplementary materials 2
		(*c*) If relevant, consider translating estimates of relative risk into absolute risk for a meaningful time period	N/A
Other analyses	17	Report other analyses done—eg analyses of subgroups and interactions, and sensitivity analyses	N/A
**Discussion**			
Key results	18	Summarise key results with reference to study objectives	26-30
Limitations	19	Discuss limitations of the study, taking into account sources of potential bias or imprecision. Discuss both direction and magnitude of any potential bias	30
Interpretation	20	Give a cautious overall interpretation of results considering objectives, limitations, multiplicity of analyses, results from similar studies, and other relevant evidence	26-31
Generalisability	21	Discuss the generalisability (external validity) of the study results	26-31
**Other information**			

For peer review only

Funding	22	Give the source of funding and the role of the funders for the present study and, if applicable, for the original study on which the present article is based	32

*Give information separately for cases and controls in case-control studies and, if applicable, for exposed and unexposed groups in cohort and cross-sectional studies.

Note: An Explanation and Elaboration article discusses each checklist item and gives methodological background and published examples of transparent reporting. The STROBE checklist is best used in conjunction with this article (freely available on the Web sites of PLoS Medicine at http://www.plosmedicine.org/, Annals of Internal Medicine at http://www.annals.org/, and Epidemiology at http://www.epidem.com/). Information on the STROBE Initiative is available at www.strobe-statement.org.

For peer review only

Did mpox knowledge, attitudes, and beliefs affect intended behaviour in the general population and men who are gay, bisexual, and who have sex with men? An online cross-sectional survey

Journal:	*BMJ Open*
Manuscript ID	bmjopen-2022-070882.R1
Article Type:	Original research
Date Submitted by the Author:	16-May-2023
Complete List of Authors:	Smith, Louise; King's College London<br>Potts, Henry; University College London, Institute of Health Informatics<br>Brainard, Julii; University of East Anglia, Norwich Medical School<br>May, Tom; University of Bristol<br>Oliver, Isabel; UK Health Security Agency<br>Amlôt, Richard; UK Health Security Agency<br>Yardley, Lucy; University of Bristol<br>Rubin, G James; King's College London
**Primary Subject Heading**:	Public health
Secondary Subject Heading:	Health policy
Keywords:	Public health < INFECTIOUS DISEASES, PUBLIC HEALTH, SEXUAL MEDICINE

SCHOLARONE™ Manuscripts

I, the Submitting Author has the right to grant and does grant on behalf of all authors of the Work (as defined in the below author licence), an exclusive licence and/or a non-exclusive licence for contributions from authors who are: i) UK Crown employees; ii) where BMJ has agreed a CC-BY licence shall apply, and/or iii) in accordance with the terms applicable for US Federal Government officers or employees acting as part of their official duties; on a worldwide, perpetual, irrevocable, royalty-free basis to BMJ Publishing Group Ltd ("BMJ") its licensees and where the relevant Journal is co-owned by BMJ to the co-owners of the Journal, to publish the Work in this journal and any other BMJ products and to exploit all rights, as set out in our licence.

The Submitting Author accepts and understands that any supply made under these terms is made by BMJ to the Submitting Author unless you are acting as an employee on behalf of your employer or a postgraduate student of an affiliated institution which is paying any applicable article publishing charge ("APC") for Open Access articles. Where the Submitting Author wishes to make the Work available on an Open Access basis (and intends to pay the relevant APC), the terms of reuse of such Open Access shall be governed by a Creative Commons licence – details of these licences and which Creative Commons licence will apply to this Work are set out in our licence referred to above.

Other than as permitted in any relevant BMJ Author's Self Archiving Policies, I confirm this Work has not been accepted for publication elsewhere, is not being considered for publication elsewhere and does not duplicate material already published. I confirm all authors consent to publication of this Work and authorise the granting of this licence.

For peer review only

Did mpox knowledge, attitudes, and beliefs affect intended behaviour in the general population and men who are gay, bisexual, and who have sex with men? An online cross-sectional survey

For peer review only

Louise E Smith (0000-0002-1277-2564),[1,2] PhD, Henry WW Potts (0000-0002-6200-8804),[3] PhD, Julii Brainard (0000-0002-5272-7995),[2,4] PhD, Tom May (0000-0003-3077-523X),[5,6,7] Isabel Oliver (0000-0002-6106-1734),[5,9] PhD, Richard Amlôt (0000-0003-3481-6588),[2,5,9] PhD, Lucy Yardley (0000-0002-3853-883X),[5,7,8] PhD, G James Rubin,[1,2] PhD

1 King's College London, Institute of Psychiatry, Psychology and Neuroscience

2 NIHR Health Protection Research Unit in Emergency Preparedness and Response

3 University College London, Institute of Health Informatics

4 University of East Anglia, Norwich Medical School

5 NIHR Health Protection Research Unit in Behavioural Science and Evaluation, University of Bristol

6 University of Bristol, Bristol Medical School

7 University of Bristol, School of Psychological Science

8 University of Southampton, School of Psychology

9 UK Health Security Agency, Chief Scientific Officer's Group

Corresponding author: Louise E Smith, Research Fellow. Department of Psychological Medicine, King's College London, Weston Education Centre, Cutcombe Road, London, SE5 9RJ, UK. Email: louise.e.smith@kcl.ac.uk

Henry WW Potts, Professor of Health Informatics. Institute of Health Informatics, 222 Euston Road, London, NW1 2DA, UK.

For peer review only - http://bmjopen.bmj.com/site/about/guidelines.xhtml

Julii Brainard, Senior Research Associate. Norwich Medical School, University of East Anglia, Norwich, Norfolk, NR4 7TJ, UK.

Tom May, Research Fellow, NIHR Health Protection Research Unit in Behavioural Science and Evaluation, Bristol Medical School, University of Bristol, Bristol, BS8 1TU, UK.

Isabel Oliver, Chief Scientific Officer, UK Health Security Agency, 17 Smith Square, London, SW1P 3HX, UK.

Richard Amlôt, Interim Deputy Director Behavioural Science and Insights, UK Health Security Agency, 17 Smith Square, London, SW1P 3HX, UK.

Lucy Yardley, Professor of Health Psychology. School of Psychological Science, University of Bristol, 12A Priory Rd, Bristol BS8 1TU, UK.

G James Rubin, Director, NIHR Health Protection Research Unit in Emergency Preparedness and Response, King's College London, Weston Education Centre, Cutcombe Road, London, SE5 9RJ, UK.

For peer review only

For peer review only - http://bmjopen.bmj.com/site/about/guidelines.xhtml

ABSTRACT

Objectives: To investigate rates of mpox beliefs, knowledge, and intended behaviours in the general population and in gay, bisexual or other men who have sex with men (GBMSM), and factors associated with intended behaviours. To test the impact of motivational messages (vs a factual control) on intended behaviours.

Design: Cross-sectional online survey including a nested randomised controlled trial.

Setting: Data collected 5 September to 6 October 2022.

Participants: Participants were aged 18 years and over and lived in the UK (general population). In addition, GBMSM were male, and gay, bisexual or had sex with men. The general population sample was recruited through a market research company. GBMSM were recruited through a market research company, the dating app Grindr, and targeted adverts on Meta (Facebook and Instagram).

Main outcome measures: Intention to self-isolate, seek medical help, stop all sexual contact, share details of recent sexual contacts, and accept vaccination.

Results: Socio-demographic characteristics differed by sample. There was no effect of very brief motivational messaging on behavioural intentions. Respondents from Grindr and Meta were more likely to intend to seek help immediately, completely stop sexual behaviour and be vaccinated or intend to be vaccinated, but being less likely to intend to self-isolate (*p*s<0.001). In the general population sample, intending to carry out protective behaviours was generally associated with being female, older, having less financial hardship, greater worry, higher perceived risk to others, and higher perceived susceptibility to and severity of mpox (*p*s<0.001). There were fewer associations with behaviours in the Grindr sample, possibly due to reduced power.

Conclusions: GBMSM were more likely to intend to enact protective behaviours, except for self-isolation. This may reflect targeted public health efforts and engagement with this group. Associations with socio-economic factors suggests that providing financial support may encourage people to engage with protective behaviours.

Word count: 298/300

Key words: adherence; self-isolation; symptoms; testing; mpox; psychological factors

For peer review only

STRENGTHS AND LIMITATIONS

- Anonymous cross-sectional survey in large samples of the general population and men who are gay, bisexual, or have sex with men (recruited from a market research company, the dating app Grindr, and targeted adverts on Meta [Facebook and Instagram]).
- Data collection occurred over a short period (5 September to 6 October 2022) during the mpox outbreak.
- Responses may have been affected by social desirability or recall bias, although the anonymous nature of the survey should mitigate this somewhat.
- Socio-demographic characteristics differed by sample. Participants recruited from Grindr and Meta were more likely to be working, highly educated, of higher socio-economic grade, and have less financial hardship.
- We measured behavioural intentions. Rates of engagement with behaviours may be lower. Factors associated with intentions should still be valid.

For peer review only

INTRODUCTION

Mpox (also known as monkeypox) is an orthopox virus that causes fever, headache, exhaustion, swollen glands and aches (joint, muscle, back), followed by a rash with blisters.[1] It spreads from person to person through touching clothing, bedding or towels used by someone with mpox rash, touching mpox skin blisters or scabs, and through the coughs or sneezes of someone with mpox rash (droplet transmission).[1] Since May 2022, there has been a multi-country outbreak of mpox in non-endemic countries.[2] Estimates from the World Health Organization (WHO; data up to 9 May 2023), indicate that there have been over 87,000 cases in 111 countries, resulting in 140 deaths.[3] Most cases have been in men who are gay, bisexual, or have sex with men, with close human skin-to skin contact (including sexual) being the primary driver of transmission.[4] The UK is the eigth country most affected by this mpox outbreak with 3741 cases.[3] Within the UK, most cases have been identified in England (with 69% of English cases in London). Almost all (99%) cases were men; English cases had a median age of 37 years.[5] The peak of the epidemic was seen in June and July 2022, with case numbers falling since the end of July.[6]

In the UK, people who thought they might have mpox were asked to call a sexual health clinic, to stay at home (self-isolate) and avoid close contact with other people.[1] Suspected cases were tested for mpox, and confirmed cases were asked to self-isolate for up to 21 days and engage with contact tracing.[7] Cases, their close contacts, and those most likely to be exposed to mpox were advised to be vaccinated with modified vaccinia Ankara vaccine – which offers cross-protection – to reduce transmission and prevent severe illness.[8, 9] While similar public health actions are now familiar to the public as a result of the COVID-19 pandemic, research conducted during that pandemic indicates that engagement with uptake of testing and self-isolation was sub-optimal in some groups.[10-12]

Various theories have guided research into the psychological factors associated with the uptake of health behaviours. One such theory is the Protection Motivation Theory (PMT), which states that people's intention to carry out a protective behaviour is influenced by their appraisal of the threat (perceived susceptibility to and severity of, e.g. mpox) and the coping response (perceived effectiveness of and self-efficacy for, e.g. testing, self-isolation, contact tracing, vaccination).[13] During the COVID-19 pandemic, psychological, and socio-demographic factors associated with testing and self-isolating included: higher perceived risk of COVID-19, knowledge of transmission modes, higher perceived effectiveness of protective behaviours, believing that your behaviour had an impact on transmission, and

For peer review only - http://bmjopen.bmj.com/site/about/guidelines.xhtml

belief that others in the same position would also self-isolate, being female, and having less financial hardship.[10-12, 14] Generally speaking, engagement with protective behaviours was associated with being older, apart from uptake of lateral flow testing, which was higher in younger people.[10, 11] Factors associated with COVID-19 vaccine uptake (completed and intended) included perceiving vaccination to be safe and necessary, perceiving COVID-19 to be more severe, and thinking that others would also be vaccinated.[15-18] Perception of side effects is one of the most common reasons given for refusing vaccination.[19] Historically, smallpox vaccines have been associated with severe adverse effects.[8] While vaccines currently licensed are associated with fewer severe adverse effects, this may affect people's intention to be vaccinated.

At the time of writing, few scientific studies have investigated behaviour during the 2022 mpox epidemic. While most have investigated vaccination acceptability, few have investigated engagement with a contact tracing system. Those studies that have been done suggest that knowledge of transmission modes is incomplete.[20-22] Greater agreement with vaccination for mpox was associated with perceiving the virus as more dangerous and virulent and higher worry about mpox in a survey of members of the Saudi Arabian general public.[23] In Dutch GBMSM, willingness to accept a vaccine was associated with being more worried about getting mpox, perceiving a higher risk of mpox, perceiving mpox to be more severe, thinking that vaccination is important, thinking that the vaccine was effective, and greater social norms for vaccination.[24] Another study also conducted in the Netherlands found that vaccination intention in GBMSM was associated with higher worry about mpox, knowing someone who had mpox, and being single but dating or in an open/polyamorous relationship.[25] This study also investigated self-isolation intention for 21 days, finding that higher intentions were associated with thinking that mpox had more problematic consequences, and lower education.[25] A study conducted in the UK found that agreement with self-isolation was associated with not having completed a higher education degree, not being employed, and identifying as having a disability.[26] There was no difference in agreement between GBMSM and those who were not GBMSM. Further research is needed to investigate how psychological factors may affect engagement with public health measures (isolating, testing, contact tracing, vaccination) put in place to control the spread of the mpox outbreak in the UK.

Official communications are vital during new and emerging outbreaks, and serve to inform the public about the threat, the public health response, and behaviours that people should

For peer review only - http://bmjopen.bmj.com/site/about/guidelines.xhtml

engage with in order to protect themselves and others.[27] Messages based on theories of health behaviours, such as the PMT,[13] may therefore increase engagement with protective behaviours. For example, research suggests that COVID-19 vaccination intention increased when communications emphasised the safety and effectiveness of the vaccine and using social norms interventions (e.g. asking people to "join the millions" being vaccinated).[28] While findings relating to messages emphasising the benefits of vaccination to oneself and others were mixed, there is some evidence that the influence of these messages may be most evident in strongly hesitant groups.

Decreasing rates of incidence of HIV in England [29] suggest that efforts to promote safer sexual practices in GBMSM have been successful. Existing communication channels and protocols to prevent HIV risk, may have led to increased knowledge and awareness about responsible sexual practices in GBMSM. During the mpox outbreak, concerted efforts were made by public health agencies (e.g. the UK Health Security Agency) to disseminate accurate scientific information about mpox to GBMSM in collaboration with community-based organisations and charities, the dating app Grindr, and organisers of Pride events,[30] building on existing communication channels.[31] Therefore, knowledge and beliefs about mpox may be different in GBMSM and the general population. For example, it is likely that perceived worry and risk are higher in GBMSM – the population most affected by the mpox outbreak – than the general population.

In this study, we recruited a general population sample and three GBMSM samples from: a market research company, Grindr, and Meta (Facebook and Instagram). We investigated knowledge, attitudes and beliefs about mpox, and intentions for key behaviours that could prevent the spread of mpox (self-isolation, help seeking [as the advised route into testing], sexual contact behaviour when symptomatic, contact sharing, vaccination). We used an experimental approach to investigate the impact of different brief communication approaches (promoting perceived susceptibility to and severity of illness / necessity and efficacy of the response / benefits of the response / low perceived costs of response) on intended behaviour. Psychological and socio-demographic factors associated with intended uptake of behaviours were also investigated.

For peer review only - http://bmjopen.bmj.com/site/about/guidelines.xhtml

METHOD

Design

Online cross-sectional survey conducted by Savanta, a Market Research Society (MRS) Company Partner. Data were collected between 5 September and 6 October 2022.

Participants

Eligibility criteria for the general population sample were living in the UK and being aged 18 years or over. For the GBMSM samples, additional criteria were being a man and identifying as being gay, bisexual, or having sex with men.

Recruitment for the general population sample used quota sampling, a standard opinion polling method that allows for rapid data collection. Members of Savanta's specialist research panel (n=150,000 across proprietary panels; people who have signed up to complete online surveys) were sent the survey link. Quota sampling uses pre-determined targets, based on pre-selected socio-demographic characteristics (quotas) that match the national population. Participants who belong to a quota that has already been filled are prevented from completing the survey. Therefore, response rate is not an accurate measure of response bias in quota samples. For this study, quotas were based on age, gender, socio-economic grade and Government Office Region, and reflected targets based on 2020 mid-year estimates.[32]

We recruited three GBMSM samples (Savanta, Grindr, Meta [Facebook and Instagram]). A "boost" sample of 250 GBMSM was recruited by Savanta, using the same quota sampling (excluding gender as all participants were male). We also recruited for the GBMSM sample through a push-notification inbox advert on Grindr (1.1 million adverts delivered, 24,933 opened, and 4288 clicks) and using targeted adverts on Meta (308,472 adverts shown, 108,865 adverts seen, and 3675 clicks). No quotas were placed on these samples.

Study materials

Full study materials are in supplementary materials 1. Items were based on previously validated measures,[33, 34] and items used in previous surveys during the COVID-19 pandemic by our group.[11, 14, 35, 36]

Outcome measures

Self-isolation intention was measured using two items, asking participants to imagine that they were contacted by public health officials and told that they needed to self-isolate for 21

For peer review only - http://bmjopen.bmj.com/site/about/guidelines.xhtml

days because they had mpox, and because they had come into high-risk contact with a case. Responses were given on a five-point scale from "definitely would not" to "definitely would". The order of items was randomised between participants to mitigate potential order effects. As answers on both items were highly correlated across all respondents (r = 0.77, p = 0.001), we combined responses to give a nine-point scale (2 to 10; summing responses "definitely would not" = 1 to "definitely would" = 5; higher number indicates greater intention).

To measure help-seeking intention, participants were asked to imagine that they developed an unexplained rash with blisters and learned that they had come into contact with an mpox case. They were then asked what they would do, from a list of ten behaviours including waiting to see if they got better, contacting healthcare services, letting people you had been in recent close contact know, and searching for information. Responses for each item were given on the same five-point "definitely would not" to "definitely would" scale. We created a binary variable, coding participants as intending to seek help immediately if they answered "probably" or "definitely would" to any help seeking where they would encounter a health professional (trying to book an appointment with a general practitioner, visiting a pharmacist/chemist, going to Accident & Emergency or another NHS service, calling NHS111 or 999, visiting a walk-in sexual health clinic, or calling a sexual health clinic), and did not select "wait a day or two to see if they get better or clear up on their own" vs did not select any help-seeking behaviour where they would encounter a health professional, or selected a help-seeking behaviour but also stated that they would "wait a day or two to see…".

Participants were then asked about their intended contact behaviour in the same scenario, being asked "in the following 21 days, realistically how much" they would come into skin-to skin contact with others, have sexual contact, have sex without using a condom, go to a crowded place, help or provide care for a vulnerable person, and go to a public place where they may come into physical contact with someone else. For each item, participants responded "I would completely stop doing this", "less than normal", "same as normal", "more than normal", "not applicable, I wouldn't do this anyway", "don't know" or "prefer not to say". We focused our analyses on the item asking about sexual contact (from kissing to intercourse) with other people, recoding it into a binary item ("I would completely stop doing this" vs would do this less than, same as, or more than usual). Answers of "not applicable, I wouldn't do this anyway", "don't know", and "prefer not to say" were coded as missing.

For peer review only - http://bmjopen.bmj.com/site/about/guidelines.xhtml

We measured intention to share details of close contacts by asking participants to indicate how likely they were, if asked by public health officials, to share contact details of every person who had been in their home, they had had sexual contact with, they had skin-to-skin contact with, and that they had shared bedding, towels or clothes with in the last seven days, and every place they had had sexual contact with someone. Responses were given on a five-point “definitely would not” (1) to “definitely would” (5) scale. We used the most recent official guidance on contact tracing available at the start of data analysis to select the item most relevant to contact tracing efforts (identifying every person you had sexual contact with in the last seven days).[7]

Participants were asked if they had received a smallpox vaccine in 2022. Those who indicated they had not had a vaccine were asked about their vaccination intention. We asked participants how likely they would be to have a smallpox vaccine if they were offered one. Responses were given on a five-point “definitely would not” (1) to “definitely would” (5) scale.

Motivational messaging

Very brief motivational messages were constructed based on components of the PMT.[13] The general population sample were randomised to one of four groups, and were shown messages about: 1) perceived risk of illness and necessity and efficacy of the response, 2) perceived risk of illness plus benefits of the response, 3) perceived risk of illness plus low perceived costs of response, and 4) a control message of similar length giving factual details about the mpox outbreak. Messages are shown in supplementary materials 1. Messages to promote perceived risk of illness (i.e. susceptibility to and severity of mpox) were included in all motivational messages, as we hypothesised that perceiving a risk is necessary before choosing to adopt a behaviour to mitigate that risk. Due to anticipated smaller sample sizes, the GBMSM samples were randomised to one of two messages. The first included all motivational components, whereas the second, a control message, gave factual information about the mpox outbreak.

Psychological factors

We asked participants how much they had seen or heard about mpox, how worried they were about mpox, and how much risk they thought mpox posed to people in the UK and themselves personally. For these items, answers of “don’t know” were coded as missing.

For peer review only - http://bmjopen.bmj.com/site/about/guidelines.xhtml

Participants were also asked about their perceived susceptibility to mpox (two items: would be likely to come into contact with a case; would be likely to catch mpox if in contact with a case) and perceived severity of mpox. We asked participants how much they agreed that: their personal behaviour had an impact on the spread of mpox; their life had been negatively affected by changes made in response to the mpox outbreak; the risks of mpox were being exaggerated; people who catch mpox usually make a full recovery without treatment; and that mpox is only a risk to men who are gay, bisexual, or have sex with men. For perceptions such as these, with no right or wrong answers, we recoded answers of "don't know" as the mid-point on the scale.

To measure perceived knowledge, we asked participants three items about whether they had a good idea how people catch mpox, they knew the main symptoms of mpox, and they thought it would be easy to tell if someone had mpox. Knowledge about mpox symptoms was measured using a question asking participants to identify the main symptoms of mpox from a list of fifteen taken from the NHS mpox website [1] and common, non-specific symptoms. Participants could select up to four symptoms. Understanding of transmission was measured using seven items, asking about contact and droplet transmission (adapted from [33], and other modes of transmission as specified by the World Health Organization (WHO) website [37]). For factual questions such as these, we coded "don't know" as missing.

Behaviour-specific perceptions were also investigated. We used a series of ten items to measure factors that may be associated with self-isolation, including perceived effectiveness, social norms, having the necessary support, and impact on social connectedness, family wellbeing, and finances. Factors that may affect intention to seek help were measured by six items asking participants to what extent they agreed that they would not want to know the results of a mpox test, they would be worried what their friends, family, or employers thought of them if they had mpox, not wanting to have a mpox test on their medical record, testing is an effective way to prevent the spread of mpox, and being willing to contact a sexual health clinic if they thought they had mpox symptoms or had come into contact with a case. Vaccination attitudes were measured by eight items, asking about general vaccine attitudes, perceived social norms, perceived effectiveness of vaccination, worry about vaccine side-effects, that the vaccine could make you infectious to others, and thinking that those who come into high-risk contact with mpox should be vaccinated. Responses of "don't know" were recoded to the mid-point of the scale.

For peer review only - http://bmjopen.bmj.com/site/about/guidelines.xhtml

Socio-demographic characteristics

Participants were asked to report their age, gender,[38] sexual orientation,[39] socio-economic grade,[40] financial hardship (adapted from [41]), employment status, highest level of education, ethnicity, marital status, how many people lived in their household, whether they were the parent or guardian of any dependent children, and if they had any pets. Those who were employed were asked if they were a frontline health or social care worker and if they needed to leave home for work. For these items, we coded participants who were not employed as not being a frontline health or social care worker (prefer not to say coded as missing) and not needing to leave home for work, respectively. Participants were asked for their full postcode, from which region and indices of multiple deprivation were determined.[42]

We asked participants if they or a household member had a chronic illness, whether they were pregnant, had ever taken pre-exposure prophylaxis (PrEP) for HIV, and for their vaccination status for smallpox (in 2022, and before 2022), hepatitis A, and COVID-19 (two doses or more).

Participants were also asked how many male and female sexual partners they had had in the last 3 weeks and last 3 months.

Ethics

Ethical approval for this study was given by the King's College London Psychiatry, Nursing and Midwifery Research Ethics Panel (reference number: LRS/DP-21/22-32287). Participants gave informed written consent before beginning survey materials.

Patient and public involvement

To ensure the research aims and study information were appropriate, members of the public were involved in the development of the funding application and survey materials. For the funding application, six people gave feedback on the initial proposal resulting in changes to aims of the study and terminology used. For the survey materials, four lay people (two GBMSM) gave feedback on the questionnaire and motivational messages, resulting in changes to survey items and messages to improve clarity, validity, and readability of statements.

For peer review only - http://bmjopen.bmj.com/site/about/guidelines.xhtml

Power

A sample size of 3000 allows a 95% confidence interval of plus or minus 1.8% for the prevalence estimate for a survey item with a prevalence of around 50% (sample size of 250 gives a confidence interval of plus or minus 6.2%; sample size of 1000 gives a confidence interval of plus or minus 3.1%).

For multiple linear regression analyses, a sample of 830 allowed over 99% power to detect small effect sizes (f=0.10) at p=0.001 (43 predictors). For logistic regression analyses, a sample of 830 allowed over 99% power to detect small effect sizes (OR=1.68 [43]) at p=0.001 (42.5% self-isolation, 18.0% requesting a test, 79.1% sharing details of close contacts [11]).

For peer review only

Analysis

Information about data preparation is reported in supplementary materials 2.

We tested whether socio-demographic characteristics of GBMSM samples were different depending on the recruitment method (Savanta, Grindr, Meta). Due to significant differences, further analyses were conducted in each sample separately.

First, we tested the influence of motivational messages on outcomes using χ^2-tests (binary outcomes), one-way ANOVAs (general population sample, continuous outcomes), or t-tests (GBMSM, continuous outcomes). For vaccination, we investigated the influence of motivational message on intention to be vaccinated if advised (excluding people already vaccinated).

Next, we investigated psychological and contextual factors associated with intended behaviours (self-isolation, help seeking, sexual contact, sharing details of contacts, vaccination). To minimise analyses conducted, we investigated one variable per outcome, except for vaccination (GBMSM sample investigated two outcomes). For vaccination, as smallpox vaccine uptake in GBMSM was high, we used two binary outcomes: vaccination uptake in 2022 (vaccinated vs not vaccinated), and a computed variable indicating vaccine intention and uptake (vaccinated or intend to be vaccinated [“definitely” or “probably would”] vs not vaccinated and do not intend to be vaccinated [“not sure”, “probably would not”, “definitely would not”]). For the general population sample, we used only the computed variable. We conducted regressions (binary logistic for binary outcomes, linear for continuous outcomes) in the general population sample and Grindr sample (the target

For peer review only - http://bmjopen.bmj.com/site/about/guidelines.xhtml

GBMSM sample who were actively seeking new partners; Savanta sample excluded due to small numbers; Meta sample excluded as they differed significantly from the general population).

We entered variables into regressions in blocks. In the first block, we entered socio-demographic characteristics: gender (general population sample only, male / female), sexual orientation (general population sample only, straight or heterosexual / gay, lesbian, bisexual, or queer), age, region, having a dependent child in household (no / yes), employment status (working / not working), education (GCSE, vocational, A-level, no formal qualifications / degree or higher), ethnicity (White British / White other / Black, Asian, other minoritized ethnicity), marital status, living alone, having a chronic illness oneself (none / present), index of multiple deprivation (deciles), socio-economic grade (ABC1 / C2DE), financial hardship, and motivational message. In the Grindr sample, we also included smallpox vaccination status in 2022 (except for vaccine uptake outcome) and ever having taken PrEP for HIV.

In the second block, we entered psychological and contextual factors: self-reported knowledge, knowledge of mpox symptoms, knowledge of mpox transmission, amount heard about mpox, worry about mpox, perceived risk of mpox (to oneself and to people in the UK), perceived susceptibility and severity of mpox, thinking that you are immune to mpox, that your personal behaviour has an impact on how mpox spreads, that your life has been negatively affected by changes made in response to the mpox outbreak, that the risks of mpox are being exaggerated, that mpox is only a risk to men who are gay, bisexual or have sex with men, and that people who catch mpox usually make a full recovery even without treatment.

For self-isolation, help seeking and vaccination, a third block was also added, which included specific factors potentially associated with individual outcomes. Items were chosen through principal component analyses (see supplementary materials 3).

All analyses were carried out in SPSS 28. Data are unweighted.

Many comparisons were investigated in regression models (n=40 to n=43, based on outcome). Therefore, we applied a conservative Bonferroni correction and only report as significant results with $p \leq 0.001$ to reduce the risk of Type I errors.

RESULTS

Top-line results for all survey materials, by sample, are shown in supplementary materials 1. Anonymised data are available online.[44]

For peer review only - http://bmjopen.bmj.com/site/about/guidelines.xhtml

For regression analyses, we report imputed values. Results using imputed values were compared with non-imputed data. There were no substantial differences in results with and without imputed values.

Participant characteristics

There were significant differences in participant characteristics by sampling method. Most notably, participants recruited from Grindr and Meta were more likely to be working, need to leave home for work, more highly educated, higher socio-economic grade, and have less financial hardship (Table 1). In the general population sample, participants were mostly female (57%), white British (87%), with a mean age of 49 years.

For peer review only

Table 1. Participant characteristics, by recruitment method.

		General population, n=3050	GBMSM samples			
			Savanta GBMSM, n=247	Grindr, n=831	Meta, n=1036	p-value
Gender	Male (including trans man)	1278 (41.9)	238 (96.4)	828 (99.6)	1013 (97.8)	-
	Gender same as assigned at birth (no or prefer not to say)	11 (0.4)	9 (3.6)	3 (0.04)	23 (2.2)	-
	Female (including trans woman)	1729 (56.7)	-	-	-	-
	Gender same as assigned at birth (no or prefer not to say)	7 (0.02)	-	-	-	-
	Non-binary	8 (0.3)	-	-	-	-
	Gender same as assigned at birth (no or prefer not to say)	9 (0.3)	-	-	-	-
Sexual orientation	Straight or heterosexual	2795 (92.7)	-	-	-	-
	Gay, lesbian, bisexual, or queer	221 (7.3)	247 (100.0)	831 (100.0)	1036 (100.0)	-
Age	Range 18 to 98 years	M=48.6, SD=17.4	M=47.1, SD=16.5	M=44.2, SD=12.5	M=47.6, SD=11.9	<0.001*
Region†	Midlands (East and West)	522 (17.1)	39 (15.8)	73 (8.8)	85 (8.2)	<0.001*
	South (East, West, East of England)	992 (32.5)	70 (28.3)	207 (24.9)	279 (26.9)	
	North (East, West, Yorkshire and the Humber)	774 (25.4)	58 (23.5)	119 (14.3)	155 (15.0)	
	London	308 (10.1)	46 (18.6)	195 (23.5)	379 (36.6)	
	Devolved nations (Scotland, Wales, and Northern Ireland)	454 (14.9)	34 (13.8)	95 (11.4)	84 (8.1)	
	Not specified	0 (0.0)	0 (0.0)	142 (17.1)	54 (5.2)	

Dependent child in household	No	2075 (68.0)	210 (85.0)	796 (95.8)	1014 (97.9)	<0.001*
	Yes	975 (32.0)	37 (15.0)	35 (4.2)	22 (2.1)	
Employment status	Not working	1286 (42.6)	93 (38.3)	157 (19.0)	201 (19.5)	<0.001*
	Working	1736 (57.4)	150 (61.7)	669 (81.0)	831 (80.5)	
Frontline health or social care worker	No	2668 (88.3)	224 (90.7)	734 (88.6)	909 (87.9)	0.66
	Yes	355 (11.7)	23 (9.3)	94 (11.4)	125 (12.1)	
Need to leave home for work	Do not need to leave home for work	1946 (63.8)	149 (60.3)	371 (44.6)	499 (48.2)	<0.001*
	Need to leave home for work	1104 (36.2)	98 (39.7)	460 (55.4)	537 (51.8)	
Education	GCSE/vocational/A-level/No formal qualifications	2065 (67.7)	159 (64.4)	295 (35.5)	253 (24.4)	<0.001*
	Degree or higher (Bachelors, Masters, PhD)	985 (32.3)	88 (35.6)	536 (64.5)	783 (75.6)	
Ethnicity	White British	2649 (87.1)	214 (86.6)	629 (76.2)	799 (78.0)	<0.001*
	White other	116 (3.8)	18 (7.3)	117 (14.2)	163 (15.9)	
	Black, Asian, other minoritized ethnicity	275 (9.0)	15 (6.1)	80 (9.7)	63 (6.1)	
Marital status	Not partnered	1265 (41.6)	141 (57.3)	561 (68.3)	486 (47.3)	<0.001*
	Partnered	1775 (58.4)	105 (42.7)	260 (31.7)	541 (52.7)	
Live alone	Live with someone else	2387 (78.3)	146 (59.1)	473 (56.9)	656 (63.3)	0.02
	Live alone	663 (21.7)	101 (40.9)	358 (43.1)	380 (36.7)	
Own chronic illness	None	2207 (72.4)	167 (67.6)	619 (74.5)	731 (70.6)	0.05
	Present	843 (27.6)	80 (32.4)	212 (25.5)	305 (29.4)	
Household member chronic illness	None	2659 (87.2)	226 (91.5)	743 (89.4)	921 (88.9)	0.49
	Present	391 (12.8)	21 (8.5)	88 (10.6)	115 (11.1)	
Ever taken PrEP for HIV	No	2993 (98.6)	216 (88.5)	427 (51.5)	547 (52.9)	<0.001*
	Yes	42 (1.4)	28 (11.5)	402 (48.5)	487 (47.1)	
Vaccinated for smallpox in 2022	Not vaccinated	2888 (94.7)	221 (89.5)	566 (68.1)	614 (59.3)	<0.001*
	Vaccinated	162 (5.3)	26 (10.5)	265 (31.9)	422 (40.7)	
Index of multiple deprivation†	Deciles (1st = most deprived, 10th = least deprived)	M=4.5, SD=2.9	M=4.4, SD=3.0	N=689, M=4.7, SD=2.8	N=982, M=4.9, SD=2.7	<0.001*
Socio-economic grade	ABC1	1796 (58.9)	169 (68.4)	694 (83.5)	944 (91.1)	<0.001*
	C2DE	1254 (41.1)	78 (31.6)	137 (16.5)	92 (8.9)	
Financial hardship†	4 (lowest hardship) to 13 (most hardship)	N=2743, M=6.1, SD=2.3	N=223, M=5.9, SD=2.4	N=784, M=5.2, SD=1.8	N=1004, M=4.7, SD=1.3	<0.001*

*p≤0.001
†Using original (not imputed) data.

Motivational messaging

There was no effect of motivational messaging on outcomes, except for in the sample recruited from Meta (see supplementary materials 4). In this sample, those receiving the motivational message were less likely to intend to self-isolate for 21 days (t(1034)= -2.81, p=0.005; motivational message, n=529, M=7.1, SD=2.3; control message, n=507, M=7.5, SD=2.2), share details of all recent sexual contacts (t(1029)= -2.05, p=0.04; motivational message, n=526, M=4.1, SD=1.2; control message, n=505, M=4.2, SD=1.1), and be

For peer review only - http://bmjopen.bmj.com/site/about/guidelines.xhtml

vaccinated for smallpox if advised ($t(612)= -2.21$, $p=0.03$; motivational message, n=304, M=4.7, SD=0.9; control message, n=310, M=4.8, SD=0.6).

Self-isolation

Rates of intended self-isolation were higher when imagining you were a case than a high-risk contact. Three-quarters of the general population sample intended to self-isolate for 21 days if they were to develop mpox (75.2%, 95% CI 73.7% to 76.7%, n=2294; Table 2). However, only 68.9% (95% CI 67.2% to 70.5%, n=2100) intended to self-isolate if they were to come into contact with a case. Intention to self-isolate was lower in Grindr and Meta samples.

Table 2. Main behavioural outcomes, by sample.

			**N (%, 95% CI)**				
			General population, total n=3050	Savanta GBMSM, total n=247	Grindr, total n=831	Meta, total n=1036	p-value
**Self-isolation for 21 days**	If "you have" mpox	Probably or definitely would not, or not sure	756 (24.8, 23.3-26.3)	53 (21.5, 16.3-26.6)	246 (29.6, 26.5-32.7)	275 (26.5, 23.9-29.2)	-
		Probably or definitely would	2294 (75.2, 73.7-76.7)	194 (78.5, 73.4-83.7)	585 (70.4, 67.3-73.5)	761 (73.5, 70.8-76.1)	
	If "you have come into high-risk contact with someone who has" mpox	Probably or definitely would not, or not sure	950 (31.1, 29.5-32.8)	77 (31.2, 25.4-37.0)	371 (44.6, 41.3-48.0)	491 (47.4, 44.3-50.4)	-
		Probably or definitely would	2100 (68.9, 67.2-70.5)	170 (68.8, 63.0-74.6)	460 (55.4, 52-58.7)	545 (52.6, 49.6-55.7)	
	Sum of intention if case and high-risk contact	2 (lowest intention) to 10 (highest intention)	M=8.0, SD=2.2	M=7.9, SD=2.3	M=7.2, SD=2.4	M=7.3, SD=2.3	<0.001*
**Help seeking**	Any action that involves contacting a healthcare professional (by phone or in person)	Would not seek help or would "wait and see" for a day or two to see if symptoms resolved	1548 (50.8, 49.0-52.5)	125 (50.6, 44.3-56.9)	388 (46.7, 43.3-50.1)	391 (37.7, 34.8-40.7)	<0.001*
		Would seek help immediately	1502 (49.2, 47.5-51.0)	122 (49.4, 43.1-55.7)	443 (53.3, 49.9-56.7)	645 (62.3, 59.3-65.2)	
**Sexual contact behaviour †**	Have sexual contact with others	Would not completely stop	567 (22.8, 21.1-24.4)	45 (20.8, 15.4-26.3)	69 (8.7, 6.8-10.7)	69 (7.0, 5.4-8.6)	<0.001*
		Completely stop	1923 (77.2, 75.6-78.9)	171 (79.2, 73.7-84.6)	721 (91.3, 89.3-93.2)	922 (93.0, 91.4-94.6)	
**Contact sharing**	Sexual contacts	Probably or definitely would not, or not sure	697 (23.3, 21.7-24.8)	60 (24.7, 19.2-30.2)	167 (20.3, 17.5-23.0)	204 (19.8, 17.4-22.2)	-
		Probably or definitely would	2300 (76.7, 75.2-78.3)	183 (75.3, 69.8-80.8)	656 (79.7, 77-82.5)	827 (80.2, 77.8-82.6)	
	Scale	1 (definitely would not) to 5 (definitely would)	N=2997, M=4.2, SD=1.2	N=243, M=4.1, SD=1.2	N=823, M=4.1, SD=1.2	N=1031, M=4.2, SD=1.1	0.57
**Vaccination intention**	Vaccinated in 2022	No or not sure	2888 (94.7, 93.9-95.5)	221 (89.5, 85.6-93.3)	566 (68.1, 64.9-71.3)	614 (59.3, 56.3-62.3)	<0.001*
		Yes	162 (5.3, 4.5-6.1)	26 (10.5, 6.7-14.4)	265 (31.9, 28.7-35.1)	422 (40.7, 37.7-43.7)	

For peer review only - http://bmjopen.bmj.com/site/about/guidelines.xhtml

	Vaccinated in 2022 or would be vaccinated if advised	Not vaccinated and probably or definitely would not be vaccinated or not sure	786 (25.8, 24.2-27.3)	37 (15.0, 10.5-19.5)	54 (6.5, 4.8-8.2)	38 (3.7, 2.5-4.8)	<0.001*
		Vaccinated or probably or definitely would be vaccinated	2264 (74.2, 72.7-75.8)	210 (85.0, 80.5-89.5)	777 (93.5, 91.8-95.2)	998 (96.3, 95.2-97.5)	

*$p \leq 0.001$

†Answers of "don't know", "prefer not to say" and "not applicable, I wouldn't do this anyway" were coded as missing, therefore total *n*s are substantially lower (general population, n=2490; Savanta GBMSM, n=216; Grindr, n=790; Meta, n=991).

In the general population, intention to self-isolate was associated with: less financial hardship, being more worried about mpox, perceiving a bigger threat of mpox to people in the UK, greater perceived susceptibility and severity of mpox, and greater perceived social norms for self-isolation (Tables 3 and 4). Not intending to self-isolate was associated with agreeing that if you had mpox symptoms, you would not want to tell anyone as you did not want to self-isolate, believing the risks of mpox were being exaggerated, and that if you had to self-isolate due to mpox it would have a negative impact on your work (Table 4). In the Grindr sample, self-isolation intention was associated with greater perceived social norms (Table 4). Not intending to self-isolate was associated with agreeing that if you had mpox symptoms, you would not want to tell anyone as you did not want to self-isolate, and that if you had to self-isolate due to mpox it would have a negative impact on your work (Table 4).

Table 3. Associations between intending to self-isolate and socio-demographic characteristics and motivational message, by sample. A higher score indicates greater intention to self-isolate. Variables were entered into the linear regression model in blocks (block 1: socio-demographic variables and motivational message, block 2: psychological factors, block 3: isolation-specific beliefs). Results for block 3, using pooled estimates are reported.

Participant characteristics	Level	General population		Grindr	
		B (95% CI)	*p*-value	*B* (95% CI)	*p*-value
Gender	Male (including trans man)	Ref	-	-	-
	Female (including trans woman)	0.15 (0.01 to 0.30)	0.03	-	-
Sexual orientation	Straight or heterosexual	Ref	-	-	-
	Gay, lesbian, bisexual, or queer	0.06 (-0.21 to 0.33)	0.67	-	-
Age	Range 18 to 98 years	0.01 (0.00 to 0.01)	0.06	0.00 (-0.01 to 0.02)	0.66
	Quadratic term, (age – mean)^2	0.0002 (-0.0001 to 0.0005)	0.17	0.001 (0.000 to 0.002)	0.02
Region	Midlands (East and West)	Ref	-	Ref	-
	South (East, West, East of England)	-0.19 (-0.39 to 0.01)	0.07	0.02 (-0.51 to 0.55)	0.95
	North (East, West, Yorkshire and the Humber)	-0.07 (-0.28 to 0.13)	0.49	0.06 (-0.53 to 0.64)	0.85
	London	-0.11 (-0.39 to 0.16)	0.42	-0.01 (-0.59 to 0.57)	0.98

For peer review only

For peer review only - http://bmjopen.bmj.com/site/about/guidelines.xhtml

	Devolved nations (Scotland, Wales, and Northern Ireland)	-0.04 (-0.30 to 0.22)	0.78	0.48 (-0.20 to 1.16)	0.17
Dependent child in household	No	Ref	-	Ref	-
	Yes	-0.15 (-0.32 to 0.03)	0.10	-0.42 (-1.13 to 0.29)	0.25
Employment status	Not working	Ref	-	Ref	-
	Working	0.09 (-0.12 to 0.31)	0.39	-0.13 (-0.61 to 0.34)	0.58
Frontline health or social care worker	No	Ref	-	Ref	-
	Yes	0.08 (-0.14 to 0.30)	0.47	0.68 (0.24 to 1.13)	0.003
Need to leave home for work	Do not need to leave home for work	Ref	-	Ref	-
	Need to leave home for work	-0.26 (-0.45 to -0.07)	0.007	0.14 (-0.22 to 0.51)	0.44
Education	GCSE/vocational/A-level/No formal qualifications	Ref	-	Ref	-
	Degree or higher (Bachelors, Masters, PhD)	0.02 (-0.13 to 0.18)	0.77	-0.16 (-0.48 to 0.16)	0.31
Ethnicity	White British	Ref	-	Ref	-
	White other	-0.23 (-0.59 to 0.13)	0.21	-0.33 (-0.75 to 0.09)	0.12
	Black, Asian, other minoritized ethnicity	0.11 (-0.15 to 0.37)	0.40	-0.03 (-0.52 to 0.46)	0.91
Marital status	Not partnered	Ref	-	Ref	-
	Partnered	0.15 (-0.04 to 0.34)	0.12	0.13 (-0.24 to 0.49)	0.50
Live alone	Live with someone else	Ref	-	Ref	-
	Live alone	0.06 (-0.15 to 0.28)	0.56	-0.07 (-0.40 to 0.26)	0.69
Own chronic illness	None	Ref	-	Ref	-
	Present	0.21 (0.05 to 0.38)	0.009	0.10 (-0.24 to 0.44)	0.56
Ever taken PrEP for HIV	No	-	-	Ref	-
	Yes	-	-	-0.11 (-0.43 to 0.22)	0.51
Vaccinated for smallpox in 2022	Not vaccinated	-	-	Ref	-
	Vaccinated	-	-	0.03 (-0.32 to 0.39)	0.86
Index of multiple deprivation	Deciles (1st = most deprived, 10th = least deprived)	-0.01 (-0.04 to 0.02)	0.48	0.02 (-0.05 to 0.08)	0.66
Socio-economic grade	ABC1	Ref	-	Ref	-
	C2DE	-0.09 (-0.24 to 0.06)	0.24	-0.03 (-0.46 to 0.39)	0.88
Financial hardship	4 (lowest hardship) to 13 (most hardship)	-0.08 (-0.12 to -0.05)	<0.001*	-0.02 (-0.11 to 0.07)	0.70
Total number of sexual partners (male and female) in last three weeks	0	Ref	-	-	-
	1	-0.04 (-0.23 to 0.14)	0.65	-	-
	2 to 4	-0.25 (-0.59 to 0.10)	0.16	-	-
	5 or more	-0.09 (-0.69 to 0.52)	0.77	-	-
	Prefer not to say	-0.22 (-0.42 to -0.02)	0.03	-	-
Number of male sexual partners in last three weeks	0	-	-	Ref	-
	1	-	-	-0.50 (-0.92 to -0.08)	0.02
	2 to 4	-	-	-0.09 (-0.47 to 0.30)	0.65
	5 to 9	-	-	-0.48 (-1.00 to 0.03)	0.07
	10 or more	-	-	-0.32 (-0.96 to 0.33)	0.34
	Prefer not to say	-	-	0.83 (0.12 to 1.53)	0.02
Motivational message	Perceived risk of illness and necessity and efficacy of the response	-0.06 (-0.25 to 0.12)	0.50	-	-
	Perceived risk of illness and benefits of the response	-0.09 (-0.28 to 0.09)	0.33	-	-
	Perceived risk of illness and low perceived costs of response	-0.19 (-0.38 to 0.00)	0.05	-	-
	Control	Ref	-	-	-
Motivational message	All motivational components	-	-	-0.10 (-0.37 to 0.18)	0.48
	Control	-	-	Ref	-

*$p \leq 0.001$

For peer review only - http://bmjopen.bmj.com/site/about/guidelines.xhtml

For peer review only

Table 4. Associations between intending to self-isolate and psychological factors and isolation-specific beliefs, by sample. A higher score indicates greater intention to self-isolate. Variables were entered into the linear regression model in blocks (block 1: socio-demographic variables and motivational message, block 2: psychological factors, block 3: isolation-specific beliefs). Results for block 3, using pooled estimates are reported.

Factor	Level	General population *B* (95% CI)	General population *p*-value	Grindr *B* (95% CI)	Grindr *p*-value
Amount heard about mpox	I have not seen or heard anything (1) to I have seen or heard a lot (3)	0.09 (-0.07 to 0.24)	0.26	-0.09 (-0.41 to 0.23)	0.57
Worry about mpox	Not at all worried (1) to extremely worried (4)	0.26 (0.13 to 0.39)	<0.001*	0.41 (0.12 to 0.71)	0.006
Perceived risk of mpox to oneself	No risk at all (1) to very high risk (5)	-0.05 (-0.17 to 0.06)	0.39	-0.25 (-0.46 to -0.04)	0.02
Perceived risk of mpox to people in UK	No risk at all (1) to very high risk (5)	0.19 (0.07 to 0.31)	0.001*	0.030 (-0.20 to 0.25)	0.82
Perceived susceptibility and severity	Lowest (1) to highest (5)	0.03 (0.19 to 0.42)	<0.001*	0.12 (-0.13 to 0.37)	0.36
I am already immune to mpox	Strongly disagree, disagree, neither agree nor disagree, don't know	Ref	-	Ref	-
	Strongly agree and agree	0.27 (0.03 to 0.52)	0.03	-0.12 (-0.61 to 0.36)	0.62
People who catch mpox usually make a full recovery, even if they do not receive any treatment	Strongly disagree (1) to strongly agree (5)	-0.05 (-0.14 to 0.03)	0.23	-0.26 (-0.43 to -0.08)	0.004
My personal behaviour has an impact on how mpox spreads	Strongly disagree (1) to strongly agree (5)	0.09 (0.03 to 0.15)	0.004	0.08 (-0.06 to 0.22)	0.26
My life has been negatively affected by changes made in response to the mpox outbreak	Strongly disagree (1) to strongly agree (5)	-0.01 (-0.09 to 0.07)	0.89	-0.03 (-0.17 to 0.11)	0.70
The risks of mpox are being exaggerated	Strongly disagree (1) to strongly agree (5)	-0.19 (-0.27 to -0.11)	<0.001*	-0.07 (-0.24 to 0.09)	0.39
Mpox is only a risk to men who are gay, bisexual or have sex with men	Strongly disagree (1) to strongly agree (5)	-0.02 (-0.08 to 0.05)	0.58	-0.17 (-0.32 to -0.02)	0.03
Perceived knowledge	Lowest (0) to highest (3)	-0.01 (-0.08 to 0.07)	0.88	-0.18 (-0.37 to 0.02)	0.08
Knowledge of mpox symptoms	Identified no symptoms (0) to identified four symptoms (4)	0.08 (0.03 to 0.14)	0.003	0.06 (-0.06 to 0.19)	0.31
Knowledge of mpox transmission	Lowest (0) to highest (6)	0.02 (-0.03 to 0.07)	0.42	0.04 (-0.10 to 0.17)	0.59
If I had mpox symptoms, I wouldn't want to tell anyone as I don't want to self-isolate	Strongly disagree (1) to strongly agree (5)	-0.66 (-0.73 to -0.60)	<0.001*	-1.01 (-1.15 to -0.87)	<0.001*
Most people would self-isolate if they were told to	Strongly disagree (1) to strongly agree (5)	0.34 (0.28 to 0.41)	<0.001*	0.41 (0.28 to 0.54)	<0.001*
If I had to self-isolate because I had tested positive for mpox...it would have a negative impact on my work	Strongly disagree (1) to strongly agree (5)	-0.15 (-0.21 to -0.09)	<0.001*	-0.34 (-0.46 to -0.21)	<0.001*

*$p \leq 0.001$

Help seeking

Approximately half of participants in the general population, Savanta GBMSM, and Grindr samples indicated that they would seek help immediately (49.2% to 53.3%; Table 2).

For peer review only - http://bmjopen.bmj.com/site/about/guidelines.xhtml

Intention to seek help immediately was higher in the Meta sample (62.3%, 95% CI 59.3 to 65.2, n=645).

In the general population, intention to seek help immediately was associated with being older (aOR 1.012, 95% CI 1.006 to 1.019, *p*<0.001), disagreeing that the risks of mpox are being exaggerated (aOR 0.83, 95% CI 0.76 to 0.91, *p*<0.001), and being willing to contact a sexual health clinic if you thought you had mpox symptoms or had been in contact with a case (aOR 1.25, 95% CI 1.16 to 1.34, *p*<0.001; supplementary materials 5). In the Grindr sample, intention to seek help immediately was associated with being willing to contact a sexual health clinic if you thought you had mpox symptoms or had been in contact with a case (aOR 1.57, 95% CI 1.29 to 1.91, *p*<0.001; supplementary materials 5).

Sexual contact behaviour when symptomatic

In the general population and Savanta GBMSM, 77.2% (95% CI 75.6% to 78.9%, n=1923) and 79.2% (73.7% to 84.6%, n=171) intended to completely stop sexual contact if they were to develop an unexplained rash with blisters and learn that they had come into contact with a mpox case (Table 2). Rates of intending to completely stop sexual contact were significantly higher in Grindr (91.3%, 95% CI 89.3% to 93.2%, n=721) and Meta samples (93.0%, 95% CI 91.4% to 94.6%, n=922). The number of days that participants would wait before resuming sexual contact from the start of their symptoms was also higher in Grindr and Meta samples than in general population and Savanta GBMSM samples ($F(3,3900)=29.0$, $p<0.001$; general population: n=2021, M=15.7, SD=15.3; Savanta GBMSM: n=190, M=16.2, SD=14.4; Grindr: n=746, M=20.4, SD=15.8; Meta: n=947, M=20.0, SD=12.3).

In the general population, intending to completely stop any sexual contact if symptomatic was associated with being female, older, less financial hardship, and being more knowledgeable about mpox transmission (Tables 5 and 6). Not intending to completely stop sexual behaviour was associated with preferring not to say how many recent sexual partners you had had, thinking that you were already immune to mpox, that your life had been negatively affected by changes made in response to the mpox outbreak, and that the risks of mpox were being exaggerated (Tables 5 and 6). No associations reached significance (Bonferroni corrected) in the Grindr sample.

Table 5. Associations between intending to completely stop any sexual contact and socio-demographic characteristics and motivational message, by sample. Variables were entered into the logistic regression model in blocks (block 1: socio-demographic variables and

For peer review only - http://bmjopen.bmj.com/site/about/guidelines.xhtml

motivational message, block 2: psychological factors). Results for block 2, using pooled estimates are reported.

Participant characteristics	Level	General population aOR (95% CI)	General population *p*-value	Grindr aOR (95% CI)	Grindr *p*-value
Gender	Male (including trans man)	Ref	-	-	-
	Female (including trans woman)	1.90 (1.48 to 2.45)	<0.001*	-	-
Sexual orientation	Straight or heterosexual	Ref	-	-	-
	Gay, lesbian, bisexual, or queer	0.91 (0.58 to 1.41)	0.67	-	-
Age	Range 18 to 98 years	1.04 (1.03 to 1.05)	<0.001*	1.01 (0.98 to 1.04)	0.53
	Quadratic term, $(age – mean)^2$	1.000 (0.999 to 1.001)	0.87	1.000 (0.998 to 1.001)	0.71
Region	Midlands (East and West)	Ref	-	Ref	-
	South (East, West, East of England)	1.08 (0.76 to 1.54)	0.67	1.18 (0.38 to 3.66)	0.77
	North (East, West, Yorkshire and the Humber)	1.14 (0.79 to 1.66)	0.49	1.48 (0.35 to 6.21)	0.58
	London	1.17 (0.73 to 1.86)	0.52	0.94 (0.27 to 3.25)	0.92
	Devolved nations (Scotland, Wales, and Northern Ireland)	1.26 (0.79 to 2.01)	0.33	1.26 (0.31 to 5.15)	0.74
Dependent child in household	No	Ref	-	Ref	-
	Yes	0.97 (0.72 to 1.29)	0.82	0.37 (0.11 to 1.28)	0.12
Employment status	Not working	Ref	-	Ref	-
	Working	1.22 (0.90 to 1.66)	0.20	0.92 (0.39 to 2.18)	0.85
Frontline health or social care worker	No	Ref	-	Ref	-
	Yes	0.97 (0.67 to 1.39)	0.86	1.77 (0.67 to 4.65)	0.25
Education	GCSE/vocational/A-level/No formal qualifications	Ref	-	Ref	-
	Degree or higher (Bachelors, Masters, PhD)	0.96 (0.73 to 1.27)	0.79	1.87 (0.97 to 3.59)	0.06
Ethnicity	White British	Ref	-	Ref	-
	White other	0.47 (0.26 to 0.84)	0.01	0.84 (0.35 to 2.06)	0.71
	Black, Asian, other minoritized ethnicity	0.85 (0.56 to 1.27)	0.42	0.36 (0.15 to 0.85)	0.02
Marital status	Not partnered	Ref	-	Ref	-
	Partnered	0.90 (0.65 to 1.24)	0.50	1.11 (0.51 to 2.41)	0.79
Live alone	Live with someone else	Ref	-	Ref	-
	Live alone	1.16 (0.77 to 1.73)	0.48	1.12 (0.56 to 2.23)	0.75
Own chronic illness	None	Ref	-	Ref	-
	Present	0.97 (0.72 to 1.31)	0.85	1.10 (0.53 to 2.27)	0.80
Ever taken PrEP for HIV	No	-	-	Ref	-
	Yes	-	-	1.36 (0.69 to 2.71)	0.37
Vaccinated for smallpox in 2022	Not vaccinated	-	-	Ref	-
	Vaccinated	-	-	0.71 (0.33 to 1.50)	0.37
Index of multiple deprivation	Deciles (1st = most deprived, 10th = least deprived)	1.00 (0.96 to 1.06)	0.85	1.01 (0.86 to 1.19)	0.88
Socio-economic grade	ABC1	Ref	-	Ref	-
	C2DE	1.13 (0.86 to 1.49)	0.39	4.15 (1.45 to 11.85)	0.008
Financial hardship	4 (lowest hardship) to 13 (most hardship)	0.85 (0.80 to 0.90)	<0.001*	0.89 (0.74 to 1.07)	0.21
Total number of sexual partners (male and female) in last three weeks	0	Ref	-	-	-
	1	0.83 (0.59 to 1.18)	0.30	-	-
	2 to 4	0.68 (0.39 to 1.18)	0.17	-	-
	5 or more	0.37 (0.14 to 0.96)	0.04	-	-
	Prefer not to say	0.45 (0.32 to 0.64)	<0.001*	-	-
Number of male sexual partners in last three weeks	0	-	-	Ref	-
	1	-	-	0.52 (0.18 to 1.46)	0.21
	2 to 4	-	-	0.38 (0.14 to 0.98)	0.05
	5 to 9	-	-	0.21 (0.07 to 0.65)	0.007
	10 or more	-	-	0.20 (0.06 to 0.72)	0.01

For peer review only

For peer review only - http://bmjopen.bmj.com/site/about/guidelines.xhtml

	Prefer not to say	-	-	0.30 (0.08 to 1.11)	0.07
Motivational message	Perceived risk of illness and necessity and efficacy of the response	1.37 (0.97 to 1.93)	0.08	-	-
	Perceived risk of illness and benefits of the response	1.07 (0.76 to 1.49)	0.70	-	-
	Perceived risk of illness and low perceived costs of response	1.10 (0.79 to 1.55)	0.57	-	-
	Control	Ref	-	-	-
Motivational message	All motivational components	-	-	1.43 (0.80 to 2.54)	0.23
	Control	-	-	Ref	-

**p*≤0.001

Table 6. Associations between intending to completely stop any sexual contact and psychological and contextual factors, by sample. Variables were entered into the logistic regression model in blocks (block 1: socio-demographic variables and motivational message, block 2: psychological factors). Results for block 2, using pooled estimates are reported.

Factor	Level	General population aOR (95% CI)	*p*-value	Grindr aOR (95% CI)	*p*-value
Amount heard about mpox	I have not seen or heard anything (1) to I have seen or heard a lot (3)	1.42 (1.08 to 1.86)	0.01	1.38 (0.70 to 2.72)	0.35
Worry about mpox	Not at all worried (1) to extremely worried (4)	1.11 (0.88 to 1.40)	0.36	1.51 (0.82 to 2.77)	0.19
Perceived risk of mpox to oneself	No risk at all (1) to very high risk (5)	0.83 (0.68 to 1.02)	0.08	0.93 (0.60 to 1.44)	0.75
Perceived risk of mpox to people in UK	No risk at all (1) to very high risk (5)	1.10 (0.90 to 1.34)	0.35	0.99 (0.64 to 1.53)	0.95
Perceived susceptibility and severity	Lowest (1) to highest (5)	1.27 (1.03 to 1.56)	0.03	1.18 (0.72 to 1.94)	0.51
I am already immune to mpox	Strongly disagree, disagree, neither agree nor disagree, don't know	Ref	-	Ref	-
	Strongly agree and agree	0.37 (0.26 to 0.53)	<0.001*	0.78 (0.32 to 1.90)	0.59
People who catch mpox usually make a full recovery, even if they do not receive any treatment	Strongly disagree (1) to strongly agree (5)	0.87 (0.74 to 1.01)	0.08	0.66 (0.45 to 0.97)	0.04
My personal behaviour has an impact on how mpox spreads	Strongly disagree (1) to strongly agree (5)	1.03 (0.92 to 1.16)	0.57	1.42 (1.08 to 1.87)	0.01
My life has been negatively affected by changes made in response to the mpox outbreak	Strongly disagree (1) to strongly agree (5)	0.70 (0.62 to 0.80)	<0.001*	0.73 (0.55 to 0.97)	0.03
The risks of mpox are being exaggerated	Strongly disagree (1) to strongly agree (5)	0.77 (0.67 to 0.88)	<0.001*	0.81 (0.58 to 1.11)	0.18
mpox is only a risk to men who are gay, bisexual or have sex with men	Strongly disagree (1) to strongly agree (5)	0.87 (0.77 to 0.97)	0.02	0.77 (0.58 to 1.03)	0.08
Perceived knowledge	Lowest (0) to highest (3)	1.00 (0.88 to 1.15)	0.94	0.86 (0.58 to 1.28)	0.46
Knowledge of mpox symptoms	Identified no symptoms (0) to identified four symptoms (4)	1.14 (1.03 to 1.26)	0.01	1.29 (1.00 to 1.66)	0.05
Knowledge of mpox transmission	Lowest (0) to highest (6)	1.18 (1.08 to 1.29)	<0.001*	1.31 (1.00 to 1.73)	0.05

**p*≤0.001

For peer review only - http://bmjopen.bmj.com/site/about/guidelines.xhtml

Sharing details of contacts

There was no difference in intention to share details of all sexual partners in the last seven days between samples (Table 2), with 75.3% to 80.2% saying that they probably or definitely would.

In the general population sample, intention to share details of every sexual contact in the last seven days was associated with being female, older, less financial hardship, higher perceived susceptibility to and severity of mpox, and higher knowledge about modes of mpox transmission (Tables 7 and 8). Not intending to share details of every recent sexual contact was associated with preferring not to say how many recent sexual partners you had had, thinking that your life had been negatively affected by changes made in response to the mpox outbreak, and thinking that the risks of mpox had been exaggerated (Tables 7 and 8). No associations reached our threshold for significance in the Grindr sample.

Table 7. Associations between intending to share details of every sexual contact in the last seven days and socio-demographic characteristics and motivational message, by sample. A higher score indicates greater intention to share details. Variables were entered into the linear regression model in blocks (block 1: socio-demographic variables and motivational message, block 2: psychological factors). Results for block 2, using pooled estimates are reported.

Participant characteristics	Level	General population		Grindr	
		B (95% CI)	*p*-value	*B* (95% CI)	*p*-value
Gender	Male (including trans man)	Ref	-	-	-
	Female (including trans woman)	0.16 (0.07 to 0.24)	<0.001*	-	-
Sexual orientation	Straight or heterosexual	Ref	-	-	-
	Gay, lesbian, bisexual, or queer	0.12 (-0.05 to 0.28)	0.16	-	-
Age	Range 18 to 98 years	0.008 (0.005 to 0.011)	<0.001*	0.003 (-0.005 to 0.011)	0.46
	Quadratic term, (age – mean)^2	-0.00017 (-0.00034 to -0.00001)	0.04	0.0003 (-0.0002 to 0.0008)	0.19
Region	Midlands (East and West)	Ref	-	Ref	-
	South (East, West, East of England)	-0.04 (-0.16 to 0.08)	0.50	0.08 (-0.23 to 0.39)	0.60
	North (East, West, Yorkshire and the Humber)	-0.07 (-0.19 to 0.06)	0.29	0.31 (-0.05 to 0.68)	0.09
	London	0.05 (-0.11 to 0.22)	0.53	0.12 (-0.25 to 0.49)	0.52
	Devolved nations (Scotland, Wales, and Northern Ireland)	0.09 (-0.06 to 0.25)	0.24	0.26 (-0.21 to 0.73)	0.27
Dependent child in household	No	Ref	-	Ref	-
	Yes	-0.12 (-0.22 to -0.01)	0.03	-0.22 (-0.64 to 0.20)	0.30
Employment status	Not working	Ref	-	Ref	-
	Working	-0.10 (-0.20 to 0.00)	0.05	-0.01 (-0.26 to 0.23)	0.90
Frontline health or social care worker	No	Ref	-	Ref	-
	Yes	-0.04 (-0.17 to 0.09)	0.57	0.08 (-0.19 to 0.34)	0.57

For peer review only - http://bmjopen.bmj.com/site/about/guidelines.xhtml

Education	GCSE/vocational/A-level/No formal qualifications	Ref	-	Ref	-
	Degree or higher (Bachelors, Masters, PhD)	0.00 (-0.09 to 0.09)	0.97	0.13 (-0.06 to 0.32)	0.19
Ethnicity	White British	Ref	-	Ref	-
	White other	-0.29 (-0.51 to -0.08)	0.008	0.00 (-0.25 to 0.25)	0.98
	Black, Asian, other minoritized ethnicity	-0.17 (-0.32 to -0.01)	0.03	0.01 (-0.29 to 0.30)	0.97
Marital status	Not partnered	Ref	-	Ref	-
	Partnered	0.03 (-0.08 to 0.15)	0.55	0.10 (-0.11 to 0.32)	0.35
Live alone	Live with someone else	Ref	-	Ref	-
	Live alone	-0.04 (-0.17 to 0.08)	0.50	0.21 (0.01 to 0.40)	0.04
Own chronic illness	None	Ref	-	Ref	-
	Present	-0.04 (-0.14 to 0.05)	0.40	0.31 (0.11 to 0.51)	0.003
Ever taken PrEP for HIV	No	-	-	Ref	-
	Yes	-	-	0.09 (-0.10 to 0.28)	0.35
Vaccinated for smallpox in 2022	Not vaccinated	-	-	Ref	-
	Vaccinated	-	-	-0.07 (-0.28 to 0.14)	0.50
Index of multiple deprivation	Deciles (1st = most deprived, 10th = least deprived)	0.00 (-0.02 to 0.02)	0.87	0.03 (-0.02 to 0.08)	0.19
Socio-economic grade	ABC1	Ref	-	Ref	-
	C2DE	-0.12 (-0.21 to -0.03)	0.01	0.04 (-0.22 to 0.29)	0.78
Financial hardship	4 (lowest hardship) to 13 (most hardship)	-0.05 (-0.07 to -0.03)	<0.001*	-0.02 (-0.07 to 0.04)	0.53
Total number of sexual partners (male and female) in last three weeks	0	Ref	-	-	-
	1	0.06 (-0.05 to 0.17)	0.28	-	-
	2 to 4	0.07 (-0.14 to 0.27)	0.53	-	-
	5 or more	-0.09 (-0.45 to 0.28)	0.64	-	-
	Prefer not to say	-0.37 (-0.49 to -0.24)	<0.001*	-	-
Number of male sexual partners in last three weeks	0	-	-	Ref	-
	1	-	-	-0.02 (-0.27 to 0.22)	0.84
	2 to 4	-	-	-0.02 (-0.24 to 0.21)	0.90
	5 to 9	-	-	-0.08 (-0.38 to 0.23)	0.62
	10 or more	-	-	-0.58 (-0.96 to -0.20)	0.003
	Prefer not to say	-	-	-0.09 (-0.52 to 0.34)	0.67
Motivational message	Perceived risk of illness and necessity and efficacy of the response	-0.03 (-0.15 to 0.08)	0.55	-	-
	Perceived risk of illness and benefits of the response	0.03 (-0.08 to 0.14)	0.59	-	-
	Perceived risk of illness and low perceived costs of response	-0.01 (-0.12 to 0.10)	0.86	-	-
	Control	Ref	-	-	-
Motivational message	All motivational components	-	-	-0.06 (-0.23 to 0.10)	0.45
	Control	-	-	Ref	-

*$p \leq 0.001$

Table 8. Associations between intending to share details of every sexual contact in the last seven days and psychological and contextual factors, by sample. A higher score indicates greater intention to share details. Variables were entered into the linear regression model in blocks (block 1: socio-demographic variables and motivational message, block 2: psychological factors). Results for block 2, using pooled estimates are reported.

Factor	Level	General population		Grindr	
		B (95% CI)	*p*-value	*B* (95% CI)	*p*-value

For peer review only - http://bmjopen.bmj.com/site/about/guidelines.xhtml

Amount heard about mpox	I have not seen or heard anything (1) to I have seen or heard a lot (3)	-0.04 (-0.13 to 0.05)	0.43	0.01 (-0.18 to 0.20)	0.92
Worry about mpox	Not at all worried (1) to extremely worried (4)	0.08 (0.00 to 0.16)	0.06	0.19 (0.01 to 0.36)	0.03
Perceived risk of mpox to oneself	No risk at all (1) to very high risk (5)	-0.04 (-0.11 to 0.03)	0.27	-0.06 (-0.18 to 0.06)	0.34
Perceived risk of mpox to people in UK	No risk at all (1) to very high risk (5)	0.07 (0.00 to 0.14)	0.06	-0.01 (-0.14 to 0.12)	0.85
Perceived susceptibility and severity	Lowest (1) to highest (5)	0.12 (0.05 to 0.19)	<0.001*	-0.09 (-0.24 to 0.06)	0.24
I am already immune to mpox	Strongly disagree, disagree, neither agree nor disagree, don't know	Ref	-	Ref	-
	Strongly agree and agree	-0.18 (-0.32 to -0.04)	0.01	-0.09 (-0.37 to 0.20)	0.55
People who catch mpox usually make a full recovery, even if they do not receive any treatment	Strongly disagree (1) to strongly agree (5)	-0.01 (-0.06 to 0.04)	0.71	-0.06 (-0.17 to 0.04)	0.23
My personal behaviour has an impact on how mpox spreads	Strongly disagree (1) to strongly agree (5)	0.04 (0.01 to 0.08)	0.03	0.02 (-0.06 to 0.10)	0.64
My life has been negatively affected by changes made in response to the mpox outbreak	Strongly disagree (1) to strongly agree (5)	-0.10 (-0.15 to -0.06)	<0.001*	-0.03 (-0.11 to 0.06)	0.56
The risks of mpox are being exaggerated	Strongly disagree (1) to strongly agree (5)	-0.11 (-0.15 to -0.06)	<0.001*	-0.15 (-0.25 to -0.05)	0.004
Mpox is only a risk to men who are gay, bisexual or have sex with men	Strongly disagree (1) to strongly agree (5)	-0.02 (-0.06 to 0.02)	0.29	-0.07 (-0.16 to 0.01)	0.10
Perceived knowledge	Lowest (0) to highest (3)	0.05 (0.00 to 0.09)	0.04	0.02 (-0.10 to 0.13)	0.78
Knowledge of mpox symptoms	Identified no symptoms (0) to identified four symptoms (4)	0.05 (0.02 to 0.08)	0.004	0.05 (-0.02 to 0.13)	0.14
Knowledge of mpox transmission	Lowest (0) to highest (6)	0.05 (0.02 to 0.08)	<0.001*	0.05 (-0.03 to 0.13)	0.22

*$p \leq 0.001$

Vaccination

Few people had been vaccinated for smallpox in 2022 in the general population sample (5.3%, 95% CI 4.5% to 6.1%, n=162; Table 2). This was significantly higher in GBMSM samples (10.5% to 40.7%). In a measure of actual and intended vaccination, 96.3% (95% CI 95.2% to 97.5%, n=998) of the Meta sample and 93.5% (95% CI 91.8% to 95.2%, n=777) of the Grindr sample were vaccinated or intended to be vaccinated for smallpox if advised. Rates were significantly lower in the general population and Savanta GBMSM samples.

In the general population, being vaccinated for smallpox in 2022 or intending to be vaccinated if offered a vaccine was associated with being older (aOR 1.015, 95% CI 1.007 to 1.023, $p<0.001$), more worried about mpox (1.49, 95% CI 1.24 to 1.80, $p<0.001$), and perceiving a higher susceptibility to and severity of mpox (1.31, 95% CI 1.11 to 1.55, $p<0.001$; supplementary materials 6). Not intending to be vaccinated was associated with needing to leave home for work (aOR 0.56, 95% CI 0.43 to 0.73, $p<0.001$) and agreeing that

For peer review only - http://bmjopen.bmj.com/site/about/guidelines.xhtml

the risks of mpox were being exaggerated (aOR 0.76, 95% CI 0.68 to 0.85, p<0.001; supplementary materials 6).

In the Grindr sample, vaccination uptake was associated with ever having taken PrEP for HIV (aOR 8.95, 95% CI 5.61 to 14.28, p<0.001) and agreeing that you are already immune to mpox (aOR 8.83, 95% CI 4.52 to 17.22, p<0.001; supplementary materials 6). When including vaccination intention, being vaccinated for smallpox in 2022 or intending to be vaccinated if offered a vaccine was associated with agreeing that if you got a smallpox vaccine, you would be protected against mpox (aOR 3.25, 95% CI 1.97 to 5.35, p<0.001; supplementary materials 6).

DISCUSSION

We investigated mpox attitudes, beliefs, and intended behaviours in a general population sample and three GBMSM samples. Samples differed by socio-demographic characteristics. This was reflected in intended behaviours, with GBMSM recruited from Grindr or Meta being more likely to intend to seek help immediately for mpox symptoms, completely stop sexual contact when symptomatic, and be vaccinated for smallpox if advised. High rates of smallpox vaccination in these samples perhaps reflects that they were mostly educated and working, and thus more likely to be health literate and engaged with services. This was also reflected in knowledge about mpox, with Grindr and Meta samples being more likely to correctly identify mpox transmission modes and the symptoms of mpox. Within the general population, rates of "don't know" answers were high, with 18.4% selecting "don't know" when asked what the main symptoms of mpox were, and 18.7% to 24.2% selecting "don't know" for transmission modes (except for having sex with someone who has mpox, where 13.9% selected "don't know"). This is similar to other surveys of the general population, where 24% of people were not sure if mpox usually spreads by close contact with an infected person.[45]

There were no effects of motivational messaging on our behavioural outcomes, except for in the Meta sample for intention to self-isolate, share details of all recent sexual contacts, and be vaccinated for smallpox if advised. For these outcomes, intention to engage with protective behaviours was higher in the control group. In practice, however, intentions were very similar (means changed by 0.1 to 0.4) and this is unlikely to represent a meaningful difference. Another study investigating gonorrhoea reinfection in young adults has also found that those in the control group (who received monthly texts reminding them to update their contact

For peer review only - http://bmjopen.bmj.com/site/about/guidelines.xhtml

details) were less likely to be reinfected than those in the intervention group (who received a series of texts that were educational and used behaviour change techniques).[46] These messages were specifically designed to decrease stigma, and partner numbers were higher in the intervention arm. A systematic review of intervention communications during the COVID-19 pandemic found that messages about the personal and collective benefits of vaccination had mixed effects on vaccination intention, with some suggestions that this approach may be more effective in more strongly hesitant individuals.[28] These results highlight the need for empirical testing of messages on behavioural outcomes, before widespread use in public health.

Generally, higher intentions were seen in the Grindr and Meta samples, except for self-isolation. However, rates should be interpreted with caution, as intended behaviour does not always translate to enacted behaviour.[47] This was shown to be the case for intended and actual engagement with the UK contact tracing programme during the COVID-19 pandemic.[11] Rates of having already been vaccinated for smallpox in 2022 were especially high (32% Grindr, 41% Meta), again suggesting our sample may have been particularly interested in the topic of the survey, especially given issues with access to vaccination.[48] Evidence suggests that while one-off sexual encounters may make up only a minority of sexual interactions, they could account for a large proportion of mpox transmission.[49, 50] Furthermore, if encounters are anonymous, this will affect contact tracing efforts. Therefore, very high rates of intending to completely stop sexual contact with others if symptomatic (91% to 93% in Grindr and Meta samples respectively) are encouraging. In the UK, the public health agency (UK Health Security Agency) has been working in conjunction with community-based organisations and charities to raise awareness of mpox and how to prevent the transmission in GBMSM as the population most affected. Higher rates of knowledge and behavioural intentions suggest that this targeted messaging has been effective in increasing knowledge and driving protective behaviours. Public health efforts in future outbreaks should use a similar model to increase knowledge about transmission and engagement with protective behaviours in affected populations.[31]

It is notable that 19% (Grindr, Meta) to 30% of people (general population, Savanta GBMSM) agreed that is it best to avoid physical contact with GBMSM because of the mpox outbreak, perhaps also reflecting a change in behaviour in this population. Recent decreases in some sexually transmitted infections in GBMSM, and the decline in mpox transmission in England, suggest this may be the case.[6]

For peer review only - http://bmjopen.bmj.com/site/about/guidelines.xhtml

Within the general population, women, people who were older, and those with lower financial hardship were more likely to intend to carry out protective behaviours. This pattern was also widely seen during the COVID-19 pandemic [11, 51, 52] and previous outbreaks.[53] This highlights the importance of considering health equity issues to ensure effective outbreak control and has implications for policymakers. Offering financial support for protective behaviours, especially those that may affect people's ability to earn an income such as self-isolation, is likely to increase engagement, especially for those in lower income settings. Few socio-demographic characteristics were associated with outcomes in the Grindr sample. This could be because the Grindr sample differed from the general population sample on key characteristics (more educated, higher socio-economic grade, less financial hardship), or be a function of the way analyses were conducted with all variables being entered into a single regression model. The Grindr sample, being smaller, also had about half the statistical power of the general population sample.

Intention to engage in protective behaviours in the general population was also associated with psychological factors such as greater worry about mpox, perceived risk of mpox to others (but there was little evidence of an association with perceived risk to oneself), perceived susceptibility and severity of mpox, and greater knowledge about transmission. This pattern of results was also seen during the COVID-19 and aH1N1 influenza pandemics.[12, 51, 54-56] COVID-19 vaccination intention was also associated with perceived risk to others, but not to oneself.[36] Few associations were significant in the Grindr sample. In addition to the decreased statistical power, this may also reflect the different context and experience that participants in this group had. While for our general population sample the questions about mpox risk were somewhat hypothetical, the Grindr sample were more likely to be impacted by mpox (as illustrated by the high vaccination rates). Factors relating to their personal experience of the outbreak may have superseded any effect of the psychological variables that we assessed. Taken together, these results suggest that clear communications about the level of risk of infection may encourage people to enact protective behaviours when appropriate.

Agreeing that the risks of mpox were being exaggerated was associated with lower intention to engage with protective behaviours. This was also the case in previous pandemics.[51, 57] Other psychological factors associated with decreased intention to engage with protective behaviours were believing that you were already immune to mpox and that your life had been negatively affected by changes made in response to mpox. Attending an important event was

For peer review only - http://bmjopen.bmj.com/site/about/guidelines.xhtml

one of the reasons given for breaking self-isolation during the aH1N1 pandemic.[58] The peak of the mpox outbreak in the UK occurred in July 2022, a period coinciding with the summer holiday and festival season, including Pride events. It is important to be conscious of asking people to self-isolate or quarantine over periods encompassing large public events that may be happening.

Behaviour-specific beliefs were also associated with intentions. For self-isolation, greater intention was associated with greater social norms (thinking that others would also self-isolate). Greater social norms were associated with fewer outings during lockdown [14] and increased vaccination uptake and intention (in oneself and one's child) [15, 59] during the COVID-19 pandemic. Social norms were also associated with enacting protective behaviours, including self-isolation, during previous outbreaks.[53, 60] Lower self-isolation intention was associated with thinking that self-isolation would have a negative impact on your work. In this study, as in previous research,[11] the main reasons for not being able to self-isolate were needing to go out for essentials (food / medicines), for a walk or some other exercise, and for work. Results suggest that communications emphasising that others are also engaging with the protective behaviour may improve engagement with self-isolation. Research investigating the relative importance of different psychological factors and specific needs in behavioural decisions could contribute to the design of more effective communications.

In the UK, people with suspected mpox were directed to call sexual health services. Some people may have found this stigmatising as mpox is not a sexually transmitted infection.[61] Stigma surrounding having other sexually transmitted infections is associated with not seeking help.[62] In our study, immediate help seeking was associated with being willing to contact a sexual health clinic. These findings suggest that widening recommended points of contact with health services to include non-stigmatising routes for suspected mpox cases or contacts may be beneficial and could have increased help seeking.

In the Grindr sample, having been vaccinated for smallpox in 2022 was associated with having ever taken PrEP for HIV. This is probably due to how the vaccine was rolled out in the UK. GBMSM identified by sexual health services as being at highest risk of exposure – using markers similar to those used to assess eligibility for PrEP – were invited to be vaccinated.[63] Being vaccinated was also strongly associated with thinking that you were already immune to mpox in this survey. As this is a cross-sectional survey, we cannot infer the direction of results, but it is likely that respondents believed they were immune to mpox

For peer review only - http://bmjopen.bmj.com/site/about/guidelines.xhtml

because of their recent vaccination. This is interesting given limited information about the effectiveness of the vaccine at that time.

Strengths of the study include the collection of data from four large samples, including the general population and the population most affected by the recent mpox outbreak (GBMSM). We would also caution readers about several caveats for this study, including that participant socio-demographic characteristics differed by the recruitment method. Participants recruited through Grindr and Meta were more likely to be highly educated, higher socio-economic grade, and have less financial hardship. We are unsure whether this is representative of the socio-demographic characteristics of active Grindr and Meta users as we were unable to find, access, or interpret published statistics of the socio-demographic profile of users.[64] Where published, most statistics outline only age and gender, rather than the additional characteristics we were interested in.[65] High rates of smallpox vaccination in these samples suggest that these groups are likely to be interested in and have personal experience relating to the outbreak and may have been more likely to engage with protective behaviours. We do not know if survey respondents are representative in terms of their beliefs, knowledge and intended behaviours with reference to the general population sample, GBMSM generally, and GBMSM who use Grindr and Meta. While overall rates of beliefs, knowledge and intended behaviours may be affected by sampling and should be taken with caution, associations within the data are likely to hold true.[66]

Other limitations relate to the survey and statistical measures used. The use of a cross-sectional survey means that answers may have been influenced by social desirability and recall bias, although the anonymity of a written survey may have mitigated these effects to some degree. Rates of intended behaviour are often higher than enacted behaviours.[11, 47] Therefore, rates of intended behaviours should be taken with caution. Our motivational messages were very brief and only repeated once, before measuring intended behaviours and behaviour-specific attitudes. A fully developed public communications campaign where people are exposed to co-produced and repeated messages may have more influence. There were high rates of actual and intended vaccination in the Grindr sample (94%). This may have affected our ability to detect associations. Variables were entered into regression models in blocks, with results reported for the final block entered (essentially all variables entered together). Therefore, we investigated the independent effect of a variable, accounting for all other variables included.

For peer review only - http://bmjopen.bmj.com/site/about/guidelines.xhtml

This study investigated mpox beliefs, knowledge and intended behaviours in a general population sample and in GBMSM (those most affected by the current outbreak). Intended uptake of protective behaviours differed by behaviour. GBMSM generally had higher intention to engage with protective behaviours, apart from self-isolation. This may have been a function of sampling. Higher knowledge about mpox symptoms and transmission in GBMSM samples suggests that the public health messaging carried out by multiple stakeholders including the UK Health Security Agency, charities and via grass-roots community efforts has been successful and a similar model should be used in future outbreaks. There was no impact of additional motivational messaging on intended uptake of protective behaviours. Associations between increased financial hardship and lower intention to enact protective behaviours suggest that providing financial support to those affected in future outbreaks may increase uptake.

For peer review only - http://bmjopen.bmj.com/site/about/guidelines.xhtml

FUNDING SOURCES

LS, JB, RA and GJR are supported by the National Institute for Health Research Health Protection Research Unit (NIHR HPRU) in Emergency Preparedness and Response, a partnership between the UK Health Security Agency, King's College London and the University of East Anglia. Additionally, IO, LY, TM and RA are funded by the NIHR HPRU in Behavioural Science and Evaluation, a partnership between UKHSA and the University of Bristol. The views expressed are those of the authors and not necessarily those of the NIHR, UKHSA, or the Department of Health and Social Care. The study was funded by NIHR. For the purpose of open access, the author has applied a Creative Commons Attribution (CC BY) licence] to any Author Accepted Manuscript version arising.

DATA SHARING STATEMENT

Anonymised data are available online.

AUTHOR CONTRIBUTION STATEMENT

LS, HWWP, JB, TM, IO, RA, LY and GJR conceptualised the study and contributed to survey materials. LS completed analyses with guidance from HWWP, JB and GJR. LS wrote the first draft of the manuscript. LS, HWWP, JB, TM, IO, RA, LY and GJR contributed to, and approved, the final manuscript. LS is guarantor. The corresponding author attests that all listed authors meet authorship criteria and that no others meeting the criteria have been omitted.

DISSEMINATION DECLARATION

Dissemination of survey results to participants is not possible due to the anonymous nature of data collection.

COMPETING INTERESTS STATEMENT

HWWP has received additional salary support from Public Health England and NHS England and receives funding from the National Institute of Health Research. HWWP receives consultancy fees to his employer from Ipsos MORI and has a PhD student who works at and has fees paid by AstraZeneca. IO and RA are employees of UKHSA.

For peer review only

For peer review only - http://bmjopen.bmj.com/site/about/guidelines.xhtml

ACKNOWLEDGEMENTS

We would like to thank the Terrence Higgins Trust for their support with participant recruitment, and Savanta for hosting the survey. We would also like to thank study participants, for taking their time to complete the survey.

For peer review only

For peer review only - http://bmjopen.bmj.com/site/about/guidelines.xhtml

ETHICS

Ethical approval for this study was given by the King's College London Psychiatry, Nursing and Midwifery Research Ethics Panel (reference number: LRS/DP-21/22-32287). Participants gave informed written consent before beginning survey materials.

For peer review only

For peer review only - http://bmjopen.bmj.com/site/about/guidelines.xhtml

REFERENCES

1. NHS. Monkeypox. 2022 [updated 1 June 2022, accessed 7 June 2022]. Available from: https://www.nhs.uk/conditions/monkeypox/.

2. World Health Organization. Multi-country monkeypox outbreak in non-endemic countries: situation update. 2022 [updated 4 June 2022, accessed 7 June 2022]. Available from: https://www.who.int/emergencies/disease-outbreak-news/item/2022-DON390.

3. World Health Organization. 2022-23 Mpox (Monkeypox) Outbreak: Global Trends 2023 [updated 11 May 2023, accessed 12 May 2023]. Available from: https://worldhealthorg.shinyapps.io/mpx_global/#9_Useful_links_and_documentation.

4. UK Health Security Agency. Monkeypox cases confirmed in England – latest updates. 2022 [updated 7 June 2022, accessed 7 June 2022]. Available from: https://www.gov.uk/government/news/monkeypox-cases-confirmed-in-england-latest-updates.

5. UK Health Security Agency. Monkeypox outbreak: epidemiological overview, 29 November 2022. 2022 [updated 29 November 2022, accessed 1 December 2022]. Available from: https://www.gov.uk/government/publications/monkeypox-outbreak-epidemiological-overview/monkeypox-outbreak-epidemiological-overview-29-november-2022.

6. UK Health Security Agency. Investigation into monkeypox outbreak in England: technical briefing 8. 2022 [updated 23 September 2022, accessed 31 October 2022]. Available from: https://www.gov.uk/government/publications/monkeypox-outbreak-technical-briefings/investigation-into-monkeypox-outbreak-in-england-technical-briefing-8#part-2-epidemiology-update.

7. UK Health Security Agency. Monkeypox contact tracing guidance: classification of contacts and follow-up advice for non-HCID strains of monkeypox. 2022 [updated 27 September 2022, accessed 6 October 2022]. Available from: https://assets.publishing.service.gov.uk/government/uploads/system/uploads/attachment_data/file/1106691/monkeypox-contact-tracing-classification-and-vaccination-matrix-version-13-27-sept-2022.pdf.

8. UK Health Security Agency. Recommendations for the use of pre and post exposure vaccination during a monkeypox incident (updated May 2022 v6.6). 21 May 2022. Available from:

For peer review only - http://bmjopen.bmj.com/site/about/guidelines.xhtml

https://assets.publishing.service.gov.uk/government/uploads/system/uploads/attachment_data/file/1077678/Recommendations-for-use-of-pre-and-post-exposure-vaccination-during-a-monkeypox-incident.pdf.

9. UKHSA finds vaccination offers strong protection against monkeypox [press release]. 22 November 2022. Available from: https://www.gov.uk/government/news/ukhsa-finds-vaccination-offers-strong-protection-against-monkeypox.

10. Smith LE, Potts HW, Amlot R, Fear NT, Michie S, Rubin GJ. Who is engaging with lateral flow testing for COVID-19 in the UK? The COVID-19 Rapid Survey of Adherence to Interventions and Responses (CORSAIR) study. BMJ Open. 2022;12(2):e058060.

11. Smith LE, Potts HWW, Amlot R, Fear NT, Michie S, Rubin GJ. Adherence to the test, trace, and isolate system in the UK: results from 37 nationally representative surveys. BMJ. 2021;372:n608.

12. Smith LE, Potts HWW, Amlot R, Fear NT, Michie S, Rubin GJ. Intention to adhere to test, trace, and isolate during the COVID-19 pandemic (the COVID-19 Rapid Survey of Adherence to Interventions and Responses study). Br J Health Psychol. 2021.

13. Rogers RW. A Protection Motivation Theory of Fear Appeals and Attitude Change. J Psychol. 1975;91(1):93-114.

14. Smith LE, Amlot R, Lambert H, Oliver I, Robin C, Yardley L, et al. Factors associated with adherence to self-isolation and lockdown measures in the UK: a cross-sectional survey. Public Health. 2020;187:41-52.

15. Smith LE, Sim J, Cutts M, Dasch H, Amlôt R, Sevdalis N, et al. Psychosocial factors affecting COVID-19 vaccine uptake in the UK: A prospective cohort study (CoVAccS – Wave 3). Vaccine: X. 2023;13:100276.

16. Sherman SM, Sim J, Cutts M, Dasch H, Amlot R, Rubin GJ, et al. COVID-19 vaccination acceptability in the UK at the start of the vaccination programme: a nationally representative cross-sectional survey (CoVAccS - wave 2). Public Health. 2022;202:1-9.

17. Paul E, Steptoe A, Fancourt D. Attitudes towards vaccines and intention to vaccinate against COVID-19: Implications for public health communications. Lancet Reg Health Eur. 2021;1:100012.

For peer review only - http://bmjopen.bmj.com/site/about/guidelines.xhtml

18. Butter S, McGlinchey E, Berry E, Armour C. Psychological, social, and situational factors associated with COVID-19 vaccination intentions: A study of UK key workers and non-key workers. Br J Health Psychol. 2022;27(1):13-29.

19. Bish A, Yardley L, Nicoll A, Michie S. Factors associated with uptake of vaccination against pandemic influenza: a systematic review. Vaccine. 2011;29(38):6472-84.

20. Malik AA, Winters MS, Omer SB. Attitudes of the US general public towards Monkeypox. medRxiv. 2022:2022.06.20.22276527.

21. Temsah MH, Aljamaan F, Alenezi S, Abouammoh N, Alhasan K, Dasuqi SA, et al. Monkeypox Disease (MPOX) Perceptions among Healthcare Workers versus General Population during the First Month of the WHO Alert: Cross-Sectional Survey in Saudi Arabia. Vaccines (Basel). 2022;10(12).

22. Paparini S, Whitacre R, Smuk M, Thornhill J, Mwendera C, Strachan S, et al. Public understanding and awareness of and response to monkeypox virus outbreak: A cross-sectional survey of the most affected communities in the United Kingdom during the 2022 public health emergency. HIV Medicine. 2023;24(5):544-57.

23. Temsah M-H, Aljamaan F, Alenezi S, Alhasan K, Saddik B, Al-Barag A, et al. Monkeypox caused less worry than COVID-19 among the general population during the first month of the WHO Monkeypox alert: Experience from Saudi Arabia. Travel Medicine and Infectious Disease. 2022;49:102426.

24. Dukers-Muijrers NH, Evers Y, Widdershoven V, Davidovich U, Adam PC, Op de Coul EL, et al. Mpox vaccination willingness, determinants, and communication needs in gay, bisexual, and other men who have sex with men, in the context of limited vaccine availability in the Netherlands (Dutch Mpox-survey). Frontiers in Public Health. 2023;10(1058807).

25. Wang H, Abreu de Paulo KJI, Gültzow T, Zimmermann HML, Jonas KJ. Monkeypox self-diagnosis abilities, determinants of vaccination and self-isolation intention after diagnosis among MSM, the Netherlands, July 2022. Eurosurveillance. 2022;27(33):2200603.

26. Paparini S, Whitacre R, Smuk M, Thornhill J, Mwendera C, Strachan S, et al. Public understanding, awareness, and response to monkeypox virus outbreak: A cross-sectional survey of the most affected communities in the United Kingdom during the 2022 public health emergency. Personal communication.

For peer review only - http://bmjopen.bmj.com/site/about/guidelines.xhtml

27. Kapur GB, Bezek S, Dyal J. Effective communication during disasters : making use of technology, media, and human resources. Oakville: Apple Academic Press,; 2017.

28. Batteux E, Mills F, Jones LF, Symons C, Weston D. The Effectiveness of Interventions for Increasing COVID-19 Vaccine Uptake: A Systematic Review. Vaccines. 2022;10(3):386.

29. Hanum N, Cambiano V, Sewell J, Rodger AJ, Nwokolo N, Asboe D, et al. Trends in HIV incidence between 2013–2019 and association of baseline factors with subsequent incident HIV among gay, bisexual, and other men who have sex with men attending sexual health clinics in England: A prospective cohort study. PLOS Medicine. 2021;18(6):e1003677.

30. UKHSA latest findings into monkeypox outbreak [press release]. 1 June 2022. Available from: https://www.gov.uk/government/news/ukhsa-latest-findings-into-monkeypox-outbreak.

31. WHO Regional Office for Europe. Holding mass and large gathering events during the multi-country mpox outbreak in the WHO European Region: Lessons identified for future mass gathering preparedness; meeting report, 22 February 2023. Copenhagen; 2023.

32. Office for National Statistics. Mid-Year Population Estimates, UK, June 2020. 2021 [updated 25 June 2021, accessed 14 October 2022]. Available from: https://www.ons.gov.uk/peoplepopulationandcommunity/populationandmigration/populationestimates/datasets/populationestimatesforukenglandandwalesscotlandandnorthernireland.

33. Rubin GJ, Amlôt R, Page L, Pearce J, Wessely S. Assessing Perceptions AbouT Hazardous Substances (PATHS): The PATHS questionnaire. Journal of Health Psychology. 2013;18(8):1100-13.

34. Rubin GJ, Bakhshi S, Amlôt R, Fear N, Potts HWW, Michie S. The design of a survey questionnaire to measure perceptions and behaviour during an influenza pandemic: the Flu TElephone Survey Template (FluTEST). Health Services and Delivery Research. 2014;2(41).

35. Smith LE, Potts HWW, Amlot R, Fear NT, Michie S, Rubin GJ. Holding a stigmatizing attitude at the start of the COVID-19 outbreak: A cross-sectional survey. Br J Health Psychol. 2021.

For peer review only - http://bmjopen.bmj.com/site/about/guidelines.xhtml

36. Sherman SM, Smith LE, Sim J, Amlot R, Cutts M, Dasch H, et al. COVID-19 vaccination intention in the UK: results from the COVID-19 vaccination acceptability study (CoVAccS), a nationally representative cross-sectional survey. Hum Vaccin Immunother. 2021;17(6):1612-21.

37. World Health Organization. Monkeypox. 2022 [updated 19 May 2022, accessed 6 June 2022]. Available from: https://www.who.int/news-room/fact-sheets/detail/monkeypox.

38. UK Health Security Agency. GUMCAD STI Surveillance System: Data specification and technical guidance. 2021. Available from: https://assets.publishing.service.gov.uk/government/uploads/system/uploads/attachment_data/file/1032965/GUMCAD_data_specification_and_technical_guidance_2021.pdf.

39. Office for National Statistics. Sexual orientation question development for Census 2021. No date. Available from: https://www.ons.gov.uk/census/censustransformationprogramme/questiondevelopment/sexualorientationquestiondevelopmentforcensus2021#:~:text=Census%202021%20will%20be%20the,at%20the%20sexual%20orientation%20question.

40. Ipsos MediaCT. Social Grade: A Classification Tool. 2009. Available from: https://www.ipsos.com/sites/default/files/publication/6800-03/MediaCT_thoughtpiece_Social_Grade_July09_V3_WEB.pdf.

41. Organisation for Economic Co-operation and Development. Supplementary Questions: Optional Survey Questions for the OECD INFE Financial Literacy Core Questionnaire. 2012. Available from: https://www.oecd.org/finance/financial-education/49878153.pdf.

42. Ministry of Housing Communities & Local Government. English indices of deprivation 2019. 2019 [updated 26 September 2019, accessed 17 October 2022]. Available from: https://www.gov.uk/government/statistics/english-indices-of-deprivation-2019.

43. Chen H, Cohen P, Chen S. How Big is a Big Odds Ratio? Interpreting the Magnitudes of Odds Ratios in Epidemiological Studies. Communications in Statistics - Simulation and Computation. 2010;39(4):860-4.

[dataset] 44. Smith LE, Yardley L, Rubin J, May T. Mpox: Investigating public perceptions, understanding, and behaviours; Quantitative survey. Open Science Framework, 15 May 2023. https://doi.org/10.17605/OSF.IO/AD7YJ.

For peer review only - http://bmjopen.bmj.com/site/about/guidelines.xhtml

45. Annenberg Public Policy Center. Survey: 1 in 5 Americans Fear Getting Monkeypox but Many Know Little About It. 2022 [updated 29 July 2022, accessed 31 October 2022]. Available from: https://www.asc.upenn.edu/news-events/news/survey-1-5-americans-fear-getting-monkeypox-many-know-little-about-it.

46. Free C, Palmer MJ, McCarthy OL, Jerome L, Berendes S, Knight M, et al. Effectiveness of a behavioural intervention delivered by text messages (safetxt) on sexually transmitted reinfections in people aged 16-24 years: randomised controlled trial. BMJ. 2022;378:e070351.

47. Sniehotta FF, Scholz U, Schwarzer R. Bridging the intention–behaviour gap: Planning, self-efficacy, and action control in the adoption and maintenance of physical exercise. Psychology & Health. 2005;20(2):143-60.

48. Josh Parry. Monkeypox: Lack of vaccines sparks pause in UK rollout. BBC News Online. 2022 [updated 15 August 2022, accessed 11 November 2022]. Available from: https://www.bbc.co.uk/news/uk-62551821.

49. Spicknall IH, Pollock ED, Clay PA, Oster AM, Charniga K, Masters N, et al. Modeling the Impact of Sexual Networks in the Transmission of Monkeypox virus Among Gay, Bisexual, and Other Men Who Have Sex with Men - United States, 2022. MMWR Morb Mortal Wkly Rep. 2022;71(35):1131-5.

50. Endo A, Murayama H, Abbott S, Ratnayake R, Pearson CAB, Edmunds WJ, et al. Heavy-tailed sexual contact networks and monkeypox epidemiology in the global outbreak, 2022. Science. 2022;378(6615):90-4.

51. Smith LE, Potts HWW, Amlôt R, Fear NT, Michie S, Rubin GJ. Patterns of social mixing in England changed in line with restrictions during the COVID-19 pandemic (September 2020 to April 2022). Scientific Reports. 2022;12(1):10436.

52. Wright L, Steptoe A, Fancourt D. Patterns of compliance with COVID-19 preventive behaviours: a latent class analysis of 20 000 UK adults. J Epidemiol Community Health. 2022;76(3):247-53.

53. Bish A, Michie S. Demographic and attitudinal determinants of protective behaviours during a pandemic: A review. British Journal of Health Psychology. 2010;15(4):797-824.

54. Smith LE, Potts HWW, Amlot R, Fear NT, Michie S, Rubin GJ. Worry and behaviour at the start of the COVID-19 outbreak: Results from three UK surveys (the COVID-19 rapid

For peer review only

For peer review only - http://bmjopen.bmj.com/site/about/guidelines.xhtml

survey of Adherence to Interventions and responses [CORSAIR] study). Prev Med Rep. 2022;25:101686.

55. Dryhurst S, Schneider CR, Kerr J, Freeman ALJ, Recchia G, van der Bles AM, et al. Risk perceptions of COVID-19 around the world. Journal of Risk Research. 2020;23(7-8):994-1006.

56. Rubin GJ, Potts HW, Michie S. The impact of communications about swine flu (influenza A H1N1v) on public responses to the outbreak: results from 36 national telephone surveys in the UK. Health Technol Assess. 2010;14(34):183-266.

57. Rubin GJ, Amlôt R, Page L, Wessely S. Public perceptions, anxiety, and behaviour change in relation to the swine flu outbreak: cross sectional telephone survey. BMJ. 2009;339:b2651.

58. Teh B, Olsen K, Black J, Cheng AC, Aboltins C, Bull K, et al. Impact of swine influenza and quarantine measures on patients and households during the H1N1/09 pandemic. Scandinavian Journal of Infectious Diseases. 2012;44(4):289-96.

59. Smith LE, Sherman SM, Sim J, Amlôt R, Cutts M, Dasch H, et al. Parents' intention to vaccinate their child for COVID-19: A mixed-methods study (CoVAccS-wave 3). PLoS One. 2022;17(12):e0279285.

60. Webster RK, Brooks SK, Smith LE, Woodland L, Wessely S, Rubin GJ. How to improve adherence with quarantine: rapid review of the evidence. Public Health. 2020;182:163-9.

61. März JW, Holm S, Biller-Andorno N. Monkeypox, stigma and public health. The Lancet Regional Health – Europe. 2022;23.

62. Stangl AL, Pliakas T, Izazola-Licea JA, Ayala G, Beattie TS, Ferguson L, et al. Removing the societal and legal impediments to the HIV response: An evidence-based framework for 2025 and beyond. PLOS ONE. 2022;17(2):e0264249.

63. Joint Committee on Vaccination and Immunisation. JCVI statement on vaccine dose prioritisation in response to the monkeypox outbreak. 2022 [updated 23 September 2022, accessed 11 November 2022]. Available from: https://www.gov.uk/government/publications/monkeypox-outbreak-jcvi-statement-on-vaccine-dose-prioritisation-september-2022/jcvi-statement-on-vaccine-dose-prioritisation-in-response-to-the-monkeypox-outbreak.

For peer review only

64. Kunst A. Grindr brand profile in the UK 2022: Statista; 2022 [updated 13 December 2022, accessed 24 April 2023]. Available from: https://www.statista.com/forecasts/1335156/grindr-dating-brand-profile-in-the-uk.

65. Dixon S. Meta audiences in the United Kingdom 2023, by age and gender: Statista; 2023 [updated 24 February 2023, accessed 24 April 2023]. Available from: https://www.statista.com/statistics/1315413/uk-meta-audiences-by-age-and-gender/.

66. Kohler U. Possible Uses of Nonprobability Sampling for the Social Sciences. Survey Methods: Insights from the Field. 2019.

For peer review only

Supplementary materials 1. Full survey materials and top-line results for general population and GBMSM samples separately

For categorical data, n (%) are shown. Percentages are column percentages. Where totals do not add up to 100%, it is due to rounding errors.

For continuous data, n (where different to total sample), mean (M), standard deviation (SD), and range are shown.

Total *ns* are: General population, n=3050; Savanta GBMSM, n=247; Grindr, n=831; Meta [Facebook and Instagram], n=1036.

For peer review only

Screening questions

ASK ALL
Postcode What is your full postcode?

We will only use your postcode to allocate you to groups based on your location (e.g. region). Your full postcode will not be passed on to the research team.

Type your answer below
OPEN END

OPEN TEXT ENTRY
Prefer not to say

New screen

ASK ALL
Gender What is your gender?
SINGLE CODE

	General population	Savanta GBMSM	Grindr	Meta
Male (including trans man)	1282 (42.0)	247 (100.0)	827 (99.5)	1030 (99.4)
Female (including trans woman)	1729 (56.7)	0 (0.0)	0 (0.0)	0 (0.0)
Non-binary	17 (0.6)	0 (0.0)	0 (0.0)	0 (0.0)
Other, please state	15 (0.5)	0 (0.0)	4 (0.5)	6 (0.6)
Prefer not to say	7 (0.2)	0 (0.0)	0 (0.0)	0 (0.0)

** For main analyses, where possible, text answers from "other, please state" were used to assign participants to a group. Therefore, numbers presented in the main manuscript may differ slightly.*

New screen

ASK ALL
SexBirth Is your gender the same as your gender assigned at birth?
SINGLE CODE

	General population	Savanta GBMSM	Grindr	Meta
Yes	3022 (99.1)	238 (96.4)	828 (99.6)	1013 (97.8)
No	22 (0.7)	9 (3.6)	2 (0.2)	18 (1.7)
Prefer not to say	6 (0.2)	0 (0.0)	1 (0.1)	5 (0.5)

New screen

ASK ALL
Age How old are you (in years)?
Type your answer below
NUMERIC - OPEN END

	General population	Savanta GBMSM	Grindr	Meta
OPEN TEXT ENTRY – number [cap at 110]	M=48.6, SD=17.4, range 18 to 98.	M=47.1, SD=16.5, range 18 to 77.	M=44.2, SD=12.5, range 18 to 82.	M=47.6, SD=11.9, range 18 to 79.

SCREEN OUT if under 18 years.

New screen

ASK ALL
Sexuality Which of the following best describes your sexual orientation?
SINGLE CODE, RANDOMISE

	General population	Savanta GBMSM	Grindr	Meta
Straight or heterosexual	2795 (91.6)	0 (0.0)	0 (0.0)	0 (0.0)
Gay or lesbian	78 (2.6)	230 (93.1)	706 (85.0)	967 (93.3)
Bisexual	135 (4.4)	16 (6.5)	118 (14.2)	55 (5.3)
Other sexual orientation, please state [ANCHOR]	18 (0.6)	1 (0.4)	7 (0.8)	14 (1.4)
Prefer not to say	24 (0.8)	0 (0.0)	0 (0.0)	0 (0.0)

For peer review only - http://bmjopen.bmj.com/site/about/guidelines.xhtml

[ANCHOR]				

** For main analyses, where possible, text answers from "other, please state" were used to assign participants to a group. Therefore, numbers presented in the main manuscript may differ slightly.*

New screen

ASK ALL

SEG Which of the following best describes the profession of the chief income earner in your household?

SINGLE CODE

	General population	Savanta GBMSM	Grindr	Meta
**High managerial, administrative or professional** e.g. doctor, lawyer, medium / large company director (50+ people)	193 (6.3)	28 (11.3)	144 (17.3)	253 (24.4)
**Intermediate managerial, administrative or professional** e.g. teacher, manager, accountant	608 (19.9)	57 (23.1)	308 (37.1)	381 (36.8)
**Supervisor, administrative or professional** e.g. police officer, nurse, secretary, self-employed	628 (20.6)	51 (20.6)	175 (21.1)	195 (18.8)
**Skilled manual worker** e.g. mechanic, plumber, electrician, lorry driver, train driver	395 (13.0)	17 (6.9)	42 (5.1)	18 (1.7)
**Semi-skilled or unskilled manual worker** e.g. waiter, factory worker, receptionist, labourer	328 (10.8)	18 (7.3)	45 (5.4)	31 (3.0)
**House-wife / house-husband**	76 (2.5)	3 (1.2)	4 (0.5)	0.0 (0.0)
**Unemployed**	176 (5.8)	24 (9.7)	27 (3.2)	26 (2.5)
**Student**	52 (1.7)	5 (2.0)	30 (3.6)	32 (3.1)
**Retired on a state pension**	215 (7.0)	9 (3.6)	15 (1.8)	13 (1.3)
**Retired on a private pension**	379 (12.4)	35 (14.2)	41 (4.9)	87 (8.4)

For peer review only - http://bmjopen.bmj.com/site/about/guidelines.xhtml

New screen

ASK ALL
QC This is a quality control question.

What colour is grass usually?

SINGLE CODE, RANDOMISE ORDER

	General population	Savanta GBMSM	Grindr	Meta
Purple	0 (0.0)	0 (0.0)	0 (0.0)	0 (0.0)
Pink	0 (0.0)	0 (0.0)	0 (0.0)	0 (0.0)
Orange	0 (0.0)	0 (0.0)	0 (0.0)	0 (0.0)
Green	3050 (100.0)	247 (100.0)	831 (100.0)	1036 (100.0)
White	0 (0.0)	0 (0.0)	0 (0.0)	0 (0.0)
Black	0 (0.0)	0 (0.0)	0 (0.0)	0 (0.0)

SCREEN OUT if do not select "green".

Beliefs and attitudes

New screen

There is an infection called "monkeypox" that has affected some people in the UK. We are interested to know your personal opinion about monkeypox, based on what you currently know.

For each question, please select the answer that reflects your opinion about monkeypox. Do not worry if you do not know what the best answer might be or if you are not at all familiar with monkeypox. We only ask that you try to give your answer based on what you think you know or what you would honestly decide to do in the situations described. If you are really not sure, please answer "don't know".

New screen

Q1. Before today, how much have you seen or heard about monkeypox?

	General population	Savanta GBMSM	Grindr	Meta
I have not seen or heard anything	117 (3.8)	6 (2.4)	7 (0.8)	2 (0.2)
I have seen or heard a little	2164 (71.0)	136 (55.1)	385 (46.3)	287 (27.7)
I have seen or heard a lot	759 (24.9)	103 (41.7)	439 (52.8)	747 (72.1)
Don't know	10 (0.3)	2 (0.8)	0 (0.0)	0 (0.0)

New screen

For peer review only - http://bmjopen.bmj.com/site/about/guidelines.xhtml

ASK ALL
Q2. Overall, how worried are you about monkeypox?
SINGLE CODE

	General population	Savanta GBMSM	Grindr	Meta
Not at all worried	635 (20.8)	40 (16.2)	77 (9.3)	47 (4.5)
Not very worried	1763 (57.8)	125 (50.6)	469 (56.4)	500 (48.3)
Very worried	452 (14.8)	61 (24.7)	234 (28.2)	410 (39.6)
Extremely worried	129 (4.2)	13 (5.3)	38 (4.6)	72 (6.9)
Don't know	71 (2.3)	8 (3.2)	13 (1.6)	7 (0.7)

New screen

ASK ALL
Q3. How much risk do you think monkeypox currently poses to:
Please select one option for each answer
RANDOMISE order of presentation of statements

People in the UK?

	General population	Savanta GBMSM	Grindr	Meta
No risk at all	106 (3.5)	9 (3.6)	11 (1.3)	3 (0.3)
Low risk	1537 (50.4)	102 (41.3)	357 (43.0)	427 (41.2)
Medium risk	1058 (34.7)	97 (39.3)	337 (40.6)	426 (41.1)
High risk	186 (6.1)	28 (11.3)	92 (11.1)	153 (14.8)
Very high risk	75 (2.5)	6 (2.4)	17 (2.0)	22 (2.1)
Don't know	88 (2.9)	5 (2.0)	17 (2.0)	5 (0.5)

You personally?

	General population	Savanta GBMSM	Grindr	Meta
No risk at all	700 (23.0)	48 (19.4)	42 (5.1)	44 (4.2)
Low risk	1619 (53.1)	96 (38.9)	291 (35.0)	266 (25.7)
Medium risk	441 (14.5)	71 (28.7)	303 (36.5)	332 (32.0)
High risk	136 (4.5)	15 (6.1)	148 (17.8)	299 (28.9)
Very high risk	58 (1.9)	13 (5.3)	37 (4.5)	93 (9.0)
Don't know	96 (3.1)	4 (1.6)	10 (1.2)	2 (0.2)

New screen

ASK ALL
Q4. How much, if at all, have you seen or heard about monkeypox? From:

The news (TV, online news websites or apps, printed newspapers, or radio)

	General population	Savanta GBMSM	Grindr	Meta

For peer review only - http://bmjopen.bmj.com/site/about/guidelines.xhtml

Not at all	210 (6.9)	14 (5.7)	61 (7.3)	54 (5.2)
A little	2125 (69.7)	149 (60.3)	533 (64.1)	652 (62.9)
A lot	693 (22.7)	82 (33.2)	235 (28.3)	330 (31.9)
Don't know	22 (0.7)	2 (0.8)	2 (0.2)	0 (0.0)

Official websites or helplines (e.g. NHS, GOV.UK), or an NHS GP practice, clinic or hospital

	General population	Savanta GBMSM	Grindr	Meta
Not at all	1547 (50.7)	98 (39.7)	198 (23.8)	188 (18.1)
A little	1113 (36.5)	97 (39.3)	450 (54.2)	601 (58.0)
A lot	316 (10.4)	48 (19.4)	169 (20.3)	241 (23.3)
Don't know	74 (2.4)	4 (1.6)	14 (1.7)	6 (0.6)

Speaking to friends, family, colleagues or other people you know (in person, by phone, text, WhatsApp, email, or in other ways)

	General population	Savanta GBMSM	Grindr	Meta
Not at all	1469 (48.2)	111 (44.9)	263 (31.6)	218 (21.0)
A little	1235 (40.5)	87 (35.2)	388 (46.7)	466 (45.0)
A lot	300 (9.8)	44 (17.8)	175 (21.1)	350 (33.8)
Don't know	46 (1.5)	5 (2.0)	5 (0.6)	2 (0.2)

Workers in community groups, community or faith leaders, charities, or volunteers who help improve the health and wellbeing of others

	General population	Savanta GBMSM	Grindr	Meta
Not at all	2111 (69.2)	165 (66.8)	553 (66.5)	633 (61.1)
A little	633 (20.8)	53 (21.5)	162 (19.5)	253 (24.4)
A lot	220 (7.2)	25 (10.1)	58 (7.0)	113 (10.9)
Don't know	86 (2.8)	4 (1.6)	58 (7.0)	37 (3.6)

Other places, including blogs, social media sites (e.g. Facebook, Twitter, Instagram), online communities or other websites

	General population	Savanta GBMSM	Grindr	Meta
Not at all	1426 (46.8)	100 (40.5)	214 (25.8)	138 (13.3)
A little	1215 (39.8)	100 (40.5)	427 (51.4)	534 (51.5)
A lot	357 (11.7)	43 (17.4)	179 (21.5)	361 (34.8)
Don't know	52 (1.7)	4 (1.6)	11 (1.3)	3 (0.3)

New screen

Q4_other. Please state any other places that you have seen or heard about monkeypox from.

Open ended, text entry

None button

New screen

For peer review only - http://bmjopen.bmj.com/site/about/guidelines.xhtml

For each question, please select the answer that reflects your opinion about monkeypox. Do not worry if you do not know what the best answer might be or if you are not at all familiar with monkeypox. We only ask that you try to give your answer based on what you think you know. If you are really not sure, please answer “don’t know”.

New screen

ASK ALL

Q5. How much do you agree or disagree with the following statements:

RANDOMISE statements

I have a good idea of how people catch monkeypox

	General population	Savanta GBMSM	Grindr	Meta
Strongly disagree	252 (8.2)	14 (5.6)	27 (3.2)	15 (1.4)
Disagree	727 (23.8)	35 (14.1)	46 (5.5)	31 (2.9)
Neither agree nor disagree	590 (19.3)	32 (12.9)	82 (9.8)	57 (5.5)
Agree	1159 (38.0)	121 (48.9)	468 (56.3)	559 (53.9)
Strongly agree	217 (7.1)	41 (16.5)	196 (23.5)	371 (35.8)
Don’t know	105 (3.4)	4 (1.6)	12 (1.4)	3 (0.2)

I know what the main symptoms of monkeypox are

	General population	Savanta GBMSM	Grindr	Meta
Strongly disagree	263 (8.6)	16 (6.5)	38 (4.6)	17 (1.6)
Disagree	820 (26.9)	44 (17.8)	100 (12.0)	60 (5.8)
Neither agree nor disagree	590 (19.3)	41 (16.6)	106 (12.8)	108 (10.4)
Agree	1113 (36.5)	120 (48.6)	493 (59.3)	642 (62.0)
Strongly agree	152 (5.0)	23 (9.3)	80 (9.6)	204 (19.7)
Don’t know	112 (3.7)	3 (1.2)	14 (1.7)	5 (0.5)

It would be easy for me to tell if someone I meet has monkeypox

	General population	Savanta GBMSM	Grindr	Meta
Strongly disagree	485 (15.9)	33 (13.4)	149 (17.9)	205 (19.8)
Disagree	1123 (36.8)	71 (28.7)	369 (44.4)	498 (48.1)
Neither agree nor disagree	663 (21.7)	64 (25.9)	181 (21.8)	209 (20.2)
Agree	504 (16.5)	54 (21.9)	88 (10.6)	92 (8.9)
Strongly agree	119 (3.9)	14 (5.7)	15 (1.8)	15 (1.4)
Don’t know	156 (5.1)	11 (4.5)	29 (3.5)	17 (1.6)

New screen

For peer review only - http://bmjopen.bmj.com/site/about/guidelines.xhtml

ASK ALL

Q6. How much do you agree or disagree with the following statements:

RANDOMISE statements

In the near future, it is likely that some of the people I come into physical contact with (touch) will have monkeypox

	General population	Savanta GBMSM	Grindr	Meta
Strongly disagree	275 (9.0)	13 (5.3)	23 (2.8)	23 (2.2)
Disagree	743 (24.4)	56 (22.7)	144 (17.3)	145 (14.0)
Neither agree nor disagree	912 (29.9)	65 (26.3)	237 (28.5)	238 (23.0)
Agree	632 (20.7)	73 (29.6)	297 (35.7)	470 (45.4)
Strongly agree	112 (3.7)	12 (4.9)	43 (5.2)	118 (11.4)
Don't know	376 (12.3)	28 (11.3)	87 (10.5)	42 (4.1)

If I come into physical contact with (touch) someone who has monkeypox, it is likely that I will catch it

	General population	Savanta GBMSM	Grindr	Meta
Strongly disagree	108 (3.5)	5 (2.0)	20 (2.4)	9 (0.9)
Disagree	467 (15.3)	24 (9.7)	93 (11.2)	145 (14.0)
Neither agree nor disagree	814 (26.7)	59 (23.9)	167 (20.1)	207 (20.0)
Agree	944 (31.0)	99 (40.1)	376 (45.2)	497 (48.0)
Strongly agree	207 (6.8)	25 (10.1)	98 (11.8)	135 (13.0)
Don't know	510 (16.7)	35 (14.2)	77 (9.3)	43 (4.2)

Monkeypox would be a serious illness for me

	General population	Savanta GBMSM	Grindr	Meta
Strongly disagree	224 (7.3)	16 (6.5)	30 (3.6)	26 (2.5)
Disagree	546 (17.9)	35 (14.2)	144 (17.3)	160 (15.4)
Neither agree nor disagree	744 (24.4)	58 (23.5)	182 (21.9)	220 (21.2)
Agree	787 (25.8)	85 (34.4)	290 (34.9)	413 (39.9)
Strongly agree	274 (9.0)	28 (11.3)	116 (14.0)	166 (16.0)
Don't know	475 (15.6)	25 (10.1)	69 (8.3)	51 (4.9)

People who catch monkeypox usually make a full recovery, even if they do not receive any treatment

	General population	Savanta GBMSM	Grindr	Meta
Strongly disagree	52 (1.7)	0 (0.0)	11 (1.3)	6 (0.6)
Disagree	264 (8.7)	25 (10.1)	77 (9.3)	62 (6.0)
Neither agree nor disagree	844 (27.7)	56 (22.7)	117 (14.1)	144 (13.9)
Agree	977 (32.0)	96 (38.9)	383 (46.1)	530 (51.2)

For peer review only - http://bmjopen.bmj.com/site/about/guidelines.xhtml

Strongly agree	228 (7.5)	29 (11.7)	102 (12.3)	210 (20.3)
Don't know	685 (22.5)	41 (16.6)	141 (17.0)	84 (8.1)

My personal behaviour has an impact on how monkeypox spreads

	General population	Savanta GBMSM	Grindr	Meta
Strongly disagree	498 (16.3)	24 (9.7)	53 (6.4)	28 (2.7)
Disagree	581 (19.0)	31 (12.6)	78 (9.4)	71 (6.9)
Neither agree nor disagree	572 (18.8)	40 (16.2)	91 (11.0)	67 (6.5)
Agree	834 (27.3)	95 (38.5)	384 (46.2)	552 (53.3)
Strongly agree	278 (9.1)	45 (18.2)	191 (23.0)	312 (30.1)
Don't know	287 (9.4)	12 (4.9)	34 (4.1)	6 (0.6)

My life has been negatively affected by changes made in response to the monkeypox outbreak

	General population	Savanta GBMSM	Grindr	Meta
Strongly disagree	1181 (38.7)	90 (36.4)	167 (20.1)	160 (15.4)
Disagree	981 (32.2)	75 (30.4)	289 (34.8)	300 (29.0)
Neither agree nor disagree	420 (13.8)	37 (15.0)	168 (20.2)	217 (20.9)
Agree	246 (8.1)	24 (9.7)	144 (17.3)	263 (25.4)
Strongly agree	115 (3.8)	12 (4.9)	42 (5.1)	88 (8.5)
Don't know	107 (3.5)	9 (3.6)	21 (2.5)	8 (0.8)

New screen

Below are some claims that some people have made about monkeypox. For each one, please say whether you strongly disagree, disagree, neither agree nor disagree, agree, or strongly agree.

ASK ALL

Q7. How much do you agree or disagree with the following claims:

SINGLE CODE

RANDOMISE statements

The risks of monkeypox are being exaggerated

	General population	Savanta GBMSM	Grindr	Meta
Strongly disagree	170 (5.6)	23 (9.3)	99 (11.9)	175 (16.9)
Disagree	822 (27.0)	87 (35.2)	326 (39.2)	505 (48.7)
Neither agree nor disagree	998 (32.7)	65 (26.3)	221 (26.6)	214 (20.7)
Agree	529 (17.3)	39 (15.8)	89 (10.7)	89 (8.6)
Strongly agree	184 (6.0)	11 (4.5)	34 (4.1)	19 (1.8)
Don't know	347 (11.4)	22 (8.9)	62 (7.5)	34 (3.3)

For peer review only - http://bmjopen.bmj.com/site/about/guidelines.xhtml

Because of the monkeypox outbreak, it is best to avoid physical contact with (touch) men who are gay, bisexual or have sex with men

	General population	Savanta GBMSM	Grindr	Meta
Strongly disagree	422 (13.8)	50 (20.2)	237 (28.5)	294 (28.4)
Disagree	707 (23.2)	64 (25.9)	267 (32.1)	357 (34.5)
Neither agree nor disagree	667 (21.9)	44 (17.8)	147 (17.7)	172 (16.6)
Agree	611 (20.0)	51 (20.6)	132 (15.9)	161 (15.5)
Strongly agree	305 (10.0)	22 (8.9)	22 (2.6)	37 (3.6)
Don't know	338 (11.1)	16 (6.5)	26 (3.1)	15 (1.4)

Because of the monkeypox outbreak, it is best to avoid physical contact with (touch) people from Africa

	General population	Savanta GBMSM	Grindr	Meta
Strongly disagree	462 (15.1)	52 (21.1)	327 (39.4)	431 (41.6)
Disagree	845 (27.7)	69 (27.9)	273 (32.9)	399 (38.5)
Neither agree nor disagree	768 (25.2)	57 (23.1)	118 (14.2)	127 (12.3)
Agree	454 (14.9)	40 (16.2)	59 (7.1)	43 (4.2)
Strongly agree	175 (5.7)	10 (4.0)	14 (1.7)	11 (1.1)
Don't know	346 (11.3)	19 (7.7)	40 (4.8)	25 (2.4)

Monkeypox is only a risk to men who are gay, bisexual or have sex with men

	General population	Savanta GBMSM	Grindr	Meta
Strongly disagree	536 (17.6)	77 (31.2)	375 (45.1)	431 (41.6)
Disagree	909 (29.8)	75 (30.4)	302 (36.3)	449 (43.3)
Neither agree nor disagree	586 (19.2)	35 (14.2)	56 (6.7)	63 (6.1)
Agree	521 (17.1)	30 (12.1)	61 (7.3)	65 (6.3)
Strongly agree	161 (5.3)	15 (6.1)	14 (1.7)	21 (2.0)
Don't know	337 (11.0)	15 (6.1)	23 (2.8)	7 (0.7)

New screen

ASK ALL

Q8. What do you think the main symptoms of monkeypox are?

Please select up to 4

MULTICODE – MAX 4

RANDOMISE

	General population	Savanta GBMSM	Grindr	Meta
High temperature / fever	1081 (35.4)	105 (42.5)	445 (53.5)	632 (61.0)

For peer review only - http://bmjopen.bmj.com/site/about/guidelines.xhtml

Shivering / chills	501 (16.4)	35 (14.2)	151 (18.2)	232 (22.4)
Muscle aches	542 (17.8)	47 (19.0)	249 (30.0)	339 (32.7)
Pain in your arms, legs or joints	385 (12.6)	45 (18.2)	117 (14.1)	170 (16.4)
Backache	104 (3.4)	10 (4.0)	19 (2.3)	23 (2.2)
Headache	426 (14.0)	39 (15.8)	146 (17.6)	181 (17.5)
Exhaustion	320 (10.5)	37 (15)	169 (20.3)	226 (21.8)
Swollen glands	496 (16.3)	68 (27.5)	256 (30.8)	446 (43.1)
Unexplained rash with blisters	1688 (55.3)	147 (59.5)	647 (77.9)	872 (84.2)
Unexplained rash without blisters	632 (20.7)	65 (26.3)	198 (23.8)	257 (24.8)
Runny or blocked nose	127 (4.2)	5 (2.0)	16 (1.9)	10 (1.0)
Dizziness	160 (5.2)	12 (4.9)	23 (2.8)	18 (1.7)
Sore throat	281 (9.2)	30 (12.1)	72 (8.7)	88 (8.5)
Nausea / feeling sick, or vomiting	388 (12.7)	46 (18.6)	68 (8.2)	77 (7.4)
Diarrhoea	182 (6)	17 (6.9)	31 (3.7)	32 (3.1)
Other, please state [ANCHOR]	5 (0.2)	0 (0.0)	9 (1.1)	12 (1.2)
Don't know [ANCHOR, EXCLUSIVE]	562 (18.4)	23 (9.3)	48 (5.8)	25 (2.4)

†Participants could select up to four answers to this question, therefore column totals add to more than 100%.

New screen

ASK ALL

Q9. You can catch monkeypox if:

Please select one option for each answer

RANDOMISE statements

You touch the rash of a person who has monkeypox

	General population	Savanta GBMSM	Grindr	Meta
Strongly disagree	68 (2.2)	3 (1.2)	11 (1.3)	6 (0.6)
Disagree	176 (5.8)	10 (4.0)	23 (2.8)	15 (1.4)
Neither agree nor disagree	516 (16.9)	28 (11.3)	48 (5.8)	38 (3.7)
Agree	1326 (43.5)	112 (45.3)	394 (47.4)	424 (40.9)
Strongly agree	394 (12.9)	65 (26.3)	293 (35.3)	519 (50.1)
Don’t know	570 (18.7)	29 (11.7)	62 (7.5)	34 (3.3)

You are coughed or sneezed on by a person who has monkeypox

	General	Savanta GBMSM	Grindr	Meta

For peer review only - http://bmjopen.bmj.com/site/about/guidelines.xhtml

	population			
Strongly disagree	82 (2.7)	17 (6.9)	50 (6.0)	82 (7.9)
Disagree	356 (11.7)	39 (15.8)	191 (23.0)	253 (24.4)
Neither agree nor disagree	634 (20.8)	41 (16.6)	127 (15.3)	160 (15.4)
Agree	1024 (33.6)	87 (35.2)	251 (30.2)	344 (33.2)
Strongly agree	273 (9.0)	22 (8.9)	74 (8.9)	82 (7.9)
Don't know	681 (22.3)	41 (16.6)	138 (16.6)	115 (11.1)

You touch a person who has monkeypox, even if they do not have a rash

	General population	Savanta GBMSM	Grindr	Meta
Strongly disagree	91 (3.0)	4 (1.6)	26 (3.1)	20 (1.9)
Disagree	419 (13.7)	28 (11.3)	139 (16.7)	189 (18.2)
Neither agree nor disagree	634 (20.8)	61 (24.7)	125 (15.0)	198 (19.1)
Agree	932 (30.6)	91 (36.8)	337 (40.6)	410 (39.6)
Strongly agree	237 (7.8)	24 (9.7)	73 (8.8)	95 (9.2)
Don't know	737 (24.2)	39 (15.8)	131 (15.8)	124 (12)

You have sex with someone who has monkeypox

	General population	Savanta GBMSM	Grindr	Meta
Strongly disagree	72 (2.4)	5 (2.0)	12 (1.4)	10 (1.0)
Disagree	99 (3.2)	4 (1.6)	5 (0.6)	7 (0.7)
Neither agree nor disagree	359 (11.8)	17 (6.9)	24 (2.9)	16 (1.5)
Agree	1293 (42.4)	120 (48.6)	387 (46.6)	441 (42.6)
Strongly agree	802 (26.3)	89 (36.0)	377 (45.4)	551 (53.2)
Don't know	425 (13.9)	12 (4.9)	26 (3.1)	11 (1.1)

You come within 1 metre of someone who has monkeypox

	General population	Savanta GBMSM	Grindr	Meta
Strongly disagree	357 (11.7)	59 (23.9)	269 (32.4)	407 (39.3)
Disagree	972 (31.9)	93 (37.7)	379 (45.6)	438 (42.3)
Neither agree nor disagree	598 (19.6)	29 (11.7)	72 (8.7)	84 (8.1)
Agree	358 (11.7)	34 (13.8)	39 (4.7)	50 (4.8)
Strongly agree	131 (4.3)	4 (1.6)	10 (1.2)	11 (1.1)
Don't know	634 (20.8)	28 (11.3)	62 (7.5)	46 (4.4)

You touch something (e.g. towels, bedding or clothing) that has been touched by a person who has monkeypox

	General population	Savanta GBMSM	Grindr	Meta
Strongly disagree	87 (2.9)	7 (2.8)	21 (2.5)	18 (1.7)

For peer review only - http://bmjopen.bmj.com/site/about/guidelines.xhtml

Disagree	414 (13.6)	30 (12.1)	85 (10.2)	72 (6.9)
Neither agree nor disagree	653 (21.4)	46 (18.6)	100 (12.0)	89 (8.6)
Agree	961 (31.5)	96 (38.9)	379 (45.6)	585 (56.5)
Strongly agree	248 (8.1)	35 (14.2)	142 (17.1)	204 (19.7)
Don't know	687 (22.5)	33 (13.4)	104 (12.5)	68 (6.6)

You touch a pet animal that has monkeypox

	General population	Savanta GBMSM	Grindr	Meta
Strongly disagree	130 (4.3)	14 (5.7)	61 (7.3)	67 (6.5)
Disagree	469 (15.4)	43 (17.4)	140 (16.8)	151 (14.6)
Neither agree nor disagree	693 (22.7)	57 (23.1)	159 (19.1)	208 (20.1)
Agree	740 (24.3)	62 (25.1)	219 (26.4)	341 (32.9)
Strongly agree	193 (6.3)	24 (9.7)	46 (5.5)	82 (7.9)
Don't know	825 (27.0)	47 (19)	206 (24.8)	187 (18.1)

Intended behaviour

Motivational messaging

New screen

[For Savanta gen pop sample, randomise people to four groups. Ppts should see EITHER group A OR group B OR group C OR group D.
For Savanta GBMSM, Grindr, Meta samples, randomise people to two groups. Ppts should see EITHER group E OR group F.]

[Savanta gen pop] GROUP A (risk + perceived necessity/efficacy): Monkeypox causes mild illness for most people, but it can be very severe and can even kill some people. Pets may also be able to catch monkeypox. People who have recently been in contact with someone who might have monkeypox and get an unexplained rash should get tested quickly. If they test positive, they should avoid close contact with others to stop monkeypox from spreading further.

[Savanta gen pop] GROUP B (risk + perceived benefits): Monkeypox causes mild illness for most people, but it can be very severe and can even kill some people. Pets may also be able to catch monkeypox. People who test positive for monkeypox can take action to protect their friends, family, and pets from infection.

[Savanta gen pop] GROUP C (risk + perceived costs): Monkeypox causes mild illness for most people, but it can be very severe and can even kill some people. Pets may also be able to catch monkeypox. Imagine that people who have to self-isolate because they test positive for monkeypox will be provided with the practical and financial support that they need to do that.

For peer review only - http://bmjopen.bmj.com/site/about/guidelines.xhtml

[Savanta gen pop] GROUP D (control message): Monkeypox is a rare infection that is mainly found in parts of west or central Africa. Recently, there has been an outbreak of monkeypox in other countries. Since early May, more than 40,000 monkeypox cases have been reported in over 80 countries where the virus is not normally seen, including the UK.

[Savanta GBMSM / Grindr / Meta] GROUP E (all messages): Monkeypox causes mild illness for most people, but it can be very severe and can even kill some people. Pets may also be able to catch monkeypox.

People who have recently been in contact with someone who might have monkeypox and get an unexplained rash should get tested quickly. If they test positive, they should avoid close contact with others to stop monkeypox from spreading further. People who test positive for monkeypox can take action to protect their friends, family, and pets from infection. Imagine that people who have to self-isolate because they test positive for monkeypox will be provided with the practical and financial support that they need to do that.

[Savanta GBMSM / Grindr / Meta] GROUP F (control message): Monkeypox is a rare infection that is mainly found in parts of west or central Africa. Recently, there has been an outbreak of monkeypox in other countries.

There is a new outbreak of monkeypox in the UK. Since early May, more than 40,000 monkeypox cases have been reported in over 80 countries where the virus is not normally seen, including the UK. The current outbreak is the first time that the virus has been passed from person to person in the UK, and so the UK Health Security Agency (UKHSA) is working with partners to put in place guidance and measures that will prevent the spread of infection.

New screen

Behaviours

Self-isolation

People who have monkeypox are being asked to self-isolate for 21 days (3 weeks). They are also being asked to tell public health officials about other people they have been in contact with.

Self-isolation means staying at home and not going to work, school or public areas. It means avoiding close contact with people you live with, spending time in separate rooms as much as possible, and not having visitors to your home.

[Randomise order of Q10 and Q11]

New screen

ASK ALL
Q10. Imagine you are contacted by public health officials after testing and told that **you have monkeypox**. They tell you that you need to self-isolate for 21 days.

For peer review only - http://bmjopen.bmj.com/site/about/guidelines.xhtml

Realistically, would you self-isolate for 21 days?
SINGLE CODE
Answer Options

	General population	Savanta GBMSM	Grindr	Meta
Definitely would not	106 (3.5)	10 (4.0)	56 (6.7)	41 (4.0)
Probably would not	283 (9.3)	24 (9.7)	116 (14.0)	144 (13.9)
Not sure	367 (12.0)	19 (7.7)	74 (8.9)	90 (8.7)
Probably would	799 (26.2)	76 (30.8)	260 (31.3)	327 (31.6)
Definitely would	1495 (49.0)	118 (47.8)	325 (39.1)	434 (41.9)

New screen

ASK ALL
Q11. Imagine you are contacted by public health officials and told that **you have come into high-risk contact with someone who has monkeypox**. They tell you that you need to self-isolate for 21 days.

Realistically, would you self-isolate for 21 days?
SINGLE CODE
Answer Options

	General population	Savanta GBMSM	Grindr	Meta
Definitely would not	126 (4.1)	15 (6.1)	78 (9.4)	70 (6.8)
Probably would not	341 (11.2)	38 (15.4)	169 (20.3)	249 (24.0)
Not sure	483 (15.8)	24 (9.7)	124 (14.9)	172 (16.6)
Probably would	907 (29.7)	77 (31.2)	261 (31.4)	322 (31.1)
Definitely would	1193 (39.1)	93 (37.7)	199 (23.9)	223 (21.5)

New screen

ASK ALL
Q12. What would stop you from being able to self-isolate for 21 days?

Please select all that apply
MULTI CODE, RANDOMISE ORDER
Answer Options

	General population	Savanta GBMSM	Grindr	Meta
I would need to go out to get food, medicines, or other essentials	992 (32.5)	119 (48.2)	477 (57.4)	604 (58.3)
I would need to go out to work	542 (17.8)	52 (21.1)	309 (37.2)	323 (31.2)
I would need to go out to provide help or care to friends or relatives	238 (7.8)	22 (8.9)	53 (6.4)	72 (6.9)

For peer review only - http://bmjopen.bmj.com/site/about/guidelines.xhtml

I would need to go out as part of looking after my children (e.g. to take them to school, or a playground)	396 (13.0)	9 (3.6)	21 (2.5)	15 (1.4)
I would need to go out for a walk or some other exercise	765 (25.1)	72 (29.1)	388 (46.7)	550 (53.1)
My home is too small	192 (6.3)	17 (6.9)	103 (12.4)	109 (10.5)
I would be too bored or lonely	344 (11.3)	39 (15.8)	170 (20.5)	242 (23.4)
I don't think it's necessary for me to self-isolate	207 (6.8)	12 (4.9)	54 (6.5)	85 (8.2)
Other, please state	40 (1.3)	3 (1.2)	40 (4.8)	52 (5.0)
Nothing, I would stay at home for 21 days [EXCLUSIVE]	1166 (38.2)	76 (30.8)	153 (18.4)	185 (17.9)

†Participants could select more than one item for this question, therefore column totals add to more than 100%.

New screen

Now we would like you to think about a different scenario.

Help seeking

New screen

Imagine that tomorrow morning **you develop an unexplained rash with blisters**. You also learn that **you have come into contact with someone who might have monkeypox**.

ASK ALL
Q13 If **you** got these symptoms, which of these things would you do?

RANDOMISE order

Wait a day or two to see if they get better or clear up on their own

	General population	Savanta GBMSM	Grindr	Meta
Definitely would not	344 (11.3)	27 (10.9)	130 (15.6)	195 (18.8)
Probably would not	546 (17.9)	47 (19.0)	175 (21.1)	248 (23.9)
Not sure	809 (26.5)	58 (23.5)	145 (17.4)	217 (20.9)
Probably would	1080 (35.4)	92 (37.2)	323 (38.9)	326 (31.5)

For peer review only - http://bmjopen.bmj.com/site/about/guidelines.xhtml

Definitely would	271 (8.9)	23 (9.3)	58 (7.0)	50 (4.8)

Try to book an appointment with a GP

	General population	Savanta GBMSM	Grindr	Meta
Definitely would not	307 (10.1)	28 (11.3)	154 (18.5)	183 (17.7)
Probably would not	409 (13.4)	48 (19.4)	167 (20.1)	295 (28.5)
Not sure	586 (19.2)	40 (16.2)	125 (15.0)	148 (14.3)
Probably would	1032 (33.8)	86 (34.8)	221 (26.6)	222 (21.4)
Definitely would	716 (23.5)	45 (18.2)	164 (19.7)	188 (18.1)

Visit a Pharmacist/Chemist for advice

	General population	Savanta GBMSM	Grindr	Meta
Definitely would not	634 (20.8)	49 (19.8)	284 (34.2)	393 (37.9)
Probably would not	749 (24.6)	69 (27.9)	329 (39.6)	438 (42.3)
Not sure	727 (23.8)	64 (25.9)	97 (11.7)	125 (12.1)
Probably would	701 (23.0)	48 (19.4)	88 (10.6)	63 (6.1)
Definitely would	239 (7.8)	17 (6.9)	33 (4.0)	17 (1.6)

Go to A&E or visit another NHS service such as a walk-in centre or minor injuries unit

	General population	Savanta GBMSM	Grindr	Meta
Definitely would not	776 (25.4)	60 (24.3)	309 (37.2)	436 (42.1)
Probably would not	841 (27.6)	66 (26.7)	274 (33.0)	365 (35.2)
Not sure	682 (22.4)	54 (21.9)	110 (13.2)	113 (10.9)
Probably would	519 (17.0)	52 (21.1)	93 (11.2)	87 (8.4)
Definitely would	232 (7.6)	15 (6.1)	45 (5.4)	35 (3.4)

Call NHS 111 or 999 / ambulance service

	General population	Savanta GBMSM	Grindr	Meta
Definitely would not	246 (8.1)	22 (8.9)	145 (17.4)	185 (17.9)
Probably would not	358 (11.7)	30 (12.1)	153 (18.4)	233 (22.5)
Not sure	556 (18.2)	36 (14.6)	108 (13.0)	118 (11.4)
Probably would	1084 (35.5)	95 (38.5)	243 (29.2)	283 (27.3)
Definitely would	806 (26.4)	64 (25.9)	182 (21.9)	217 (20.9)

Visit a walk-in sexual health service

	General population	Savanta GBMSM	Grindr	Meta
Definitely would not	906 (29.7)	50 (20.2)	164 (19.7)	207 (20.0)

For peer review only

Probably would not	783 (25.7)	56 (22.7)	192 (23.1)	203 (19.6)
Not sure	727 (23.8)	51 (20.6)	125 (15.0)	130 (12.5)
Probably would	454 (14.9)	65 (26.3)	205 (24.7)	262 (25.3)
Definitely would	180 (5.9)	25 (10.1)	145 (17.4)	234 (22.6)

Call a sexual health service [fix below 6]

	General population	Savanta GBMSM	Grindr	Meta
Definitely would not	692 (22.7)	27 (10.9)	33 (4.0)	25 (2.4)
Probably would not	690 (22.6)	38 (15.4)	68 (8.2)	57 (5.5)
Not sure	697 (22.9)	35 (14.2)	60 (7.2)	60 (5.8)
Probably would	630 (20.7)	87 (35.2)	278 (33.5)	320 (30.9)
Definitely would	341 (11.2)	60 (24.3)	392 (47.2)	574 (55.4)

Let people you have recently been in close contact with know that you have symptoms

	General population	Savanta GBMSM	Grindr	Meta
Definitely would not	81 (2.7)	8 (3.2)	34 (4.1)	32 (3.1)
Probably would not	220 (7.2)	20 (8.1)	75 (9.0)	90 (8.7)
Not sure	495 (16.2)	47 (19.0)	152 (18.3)	177 (17.1)
Probably would	1185 (38.9)	96 (38.9)	298 (35.9)	396 (38.2)
Definitely would	1069 (35)	76 (30.8)	272 (32.7)	341 (32.9)

Search for information from official websites (e.g. NHS, GOV.UK)

	General population	Savanta GBMSM	Grindr	Meta
Definitely would not	73 (2.4)	4 (1.6)	13 (1.6)	9 (0.9)
Probably would not	122 (4.0)	10 (4.0)	26 (3.1)	23 (2.2)
Not sure	325 (10.7)	22 (8.9)	30 (3.6)	24 (2.3)
Probably would	1165 (38.2)	91 (36.8)	281 (33.8)	366 (35.3)
Definitely would	1365 (44.8)	120 (48.6)	481 (57.9)	614 (59.3)

Search for information from other places, including blogs, social media sites (e.g. Facebook, Twitter, Instagram), online communities or other websites [fix below 9]

	General population	Savanta GBMSM	Grindr	Meta
Definitely would not	533 (17.5)	49 (19.8)	144 (17.3)	135 (13.0)
Probably would not	647 (21.2)	54 (21.9)	231 (27.8)	277 (26.7)
Not sure	647 (21.2)	39 (15.8)	113 (13.6)	155 (15.0)
Probably would	789 (25.9)	70 (28.3)	206 (24.8)	301 (29.1)
Definitely would	434 (14.2)	35 (14.2)	137 (16.5)	168 (16.2)

For peer review only - http://bmjopen.bmj.com/site/about/guidelines.xhtml

New screen

Imagine that tomorrow morning **you develop an unexplained rash with blisters**. You also learn that **you have come into contact with someone who might have monkeypox**.

Q13_other. Please state any other things you would do.
Open ended text
Nothing button

Contact behaviour

New screen

Imagine that tomorrow morning **you develop an unexplained rash with blisters**. You also learn that **you have come into contact with someone who might have monkeypox**.

ASK ALL
Q14 If **you** got these symptoms, in the following 21 days, realistically how much, if at all, would you:

RANDOMISE order

Come into skin-to-skin contact with other people

	General population	Savanta GBMSM	Grindr	Meta
I would completely stop doing this	1883 (61.7)	153 (61.9)	648 (78)	858 (82.8)
Less than normal	412 (13.5)	41 (16.6)	127 (15.3)	135 (13.0)
Same as normal	249 (8.2)	21 (8.5)	15 (1.8)	12 (1.2)
More than normal	107 (3.5)	8 (3.2)	3 (0.4)	1 (0.1)
Not applicable, I wouldn't do this anyway	280 (9.2)	16 (6.5)	22 (2.6)	21 (2.0)
Don't know	109 (3.6)	7 (2.8)	15 (1.8)	9 (0.9)
Prefer not to say	10 (0.3)	1 (0.4)	1 (0.1)	0 (0.0)

Have sexual contact (from kissing to intercourse) with other people

	General population	Savanta GBMSM	Grindr	Meta
I would completely stop doing this	1923 (63.0)	171 (69.2)	721 (86.8)	922 (89.0)
Less than normal	238 (7.8)	20 (8.1)	54 (6.5)	61 (5.9)
Same as normal	216 (7.1)	15 (6.1)	14 (1.7)	7 (0.7)
More than normal	113 (3.7)	10 (4.0)	1 (0.1)	1 (0.1)
Not applicable, I wouldn't do this anyway	451 (14.8)	24 (9.7)	27 (3.2)	30 (2.9)
Don't know	93 (3.0)	6 (2.4)	13 (1.6)	13 (1.3)
Prefer not to say	16 (0.5)	1 (0.4)	1 (0.1)	2 (0.2)

For peer review only - http://bmjopen.bmj.com/site/about/guidelines.xhtml

Have sex without using a condom

	General population	Savanta GBMSM	Grindr	Meta
I would completely stop doing this	1729 (56.7)	155 (62.8)	623 (75)	788 (76.1)
Less than normal	146 (4.8)	17 (6.9)	33 (4.0)	37 (3.6)
Same as normal	235 (7.7)	15 (6.1)	27 (3.2)	29 (2.8)
More than normal	124 (4.1)	11 (4.5)	3 (0.4)	2 (0.2)
Not applicable, I wouldn't do this anyway	680 (22.3)	42 (17.0)	123 (14.8)	161 (15.5)
Don't know	111 (3.6)	7 (2.8)	17 (2.0)	14 (1.4)
Prefer not to say	25 (0.8)	0 (0.0)	5 (0.6)	5 (0.5)

Share bedding, towels, or clothes with other people

	General population	Savanta GBMSM	Grindr	Meta
I would completely stop doing this	1839 (60.3)	147 (59.5)	604 (72.7)	807 (77.9)
Less than normal	286 (9.4)	23 (9.3)	62 (7.5)	96 (9.3)
Same as normal	279 (9.1)	21 (8.5)	24 (2.9)	19 (1.8)
More than normal	124 (4.1)	12 (4.9)	2 (0.2)	1 (0.1)
Not applicable, I wouldn't do this anyway	415 (13.6)	41 (16.6)	126 (15.2)	104 (10.0)
Don't know	98 (3.2)	3 (1.2)	12 (1.4)	8 (0.8)
Prefer not to say	9 (0.3)	0 (0.0)	1 (0.1)	1 (0.1)

Go to a crowded place

	General population	Savanta GBMSM	Grindr	Meta
I would completely stop doing this	1872 (61.4)	141 (57.1)	552 (66.4)	663 (64.0)
Less than normal	418 (13.7)	50 (20.2)	183 (22)	297 (28.7)
Same as normal	248 (8.1)	23 (9.3)	57 (6.9)	39 (3.8)
More than normal	123 (4.0)	9 (3.6)	1 (0.1)	1 (0.1)
Not applicable, I wouldn't do this anyway	275 (9.0)	16 (6.5)	25 (3.0)	23 (2.2)
Don't know	103 (3.4)	8 (3.2)	13 (1.6)	12 (1.2)
Prefer not to say	11 (0.4)	0 (0.0)	0 (0.0)	1 (0.1)

Help or provide care for a vulnerable person

	General population	Savanta GBMSM	Grindr	Meta
I would completely stop doing this	1674 (54.9)	131 (53.0)	516 (62.1)	686 (66.2)
Less than normal	304 (10.0)	16 (6.5)	81 (9.7)	81 (7.8)
Same as normal	255 (8.4)	24 (9.7)	23 (2.8)	24 (2.3)
More than normal	160 (5.2)	14 (5.7)	8 (1.0)	6 (0.6)
Not applicable, I wouldn't do this anyway	513 (16.8)	50 (20.2)	181 (21.8)	217 (20.9)

For peer review only - http://bmjopen.bmj.com/site/about/guidelines.xhtml

Don't know	134 (4.4)	11 (4.5)	22 (2.6)	20 (1.9)
Prefer not to say	10 (0.3)	1 (0.4)	0 (0.0)	2 (0.2)

Go to a public place where you may come into physical contact with (touch) someone else

	General population	Savanta GBMSM	Grindr	Meta
I would completely stop doing this	1818 (59.6)	146 (59.1)	525 (63.2)	653 (63.0)
Less than normal	469 (15.4)	51 (20.6)	236 (28.4)	318 (30.7)
Same as normal	249 (8.2)	20 (8.1)	33 (4.0)	24 (2.3)
More than normal	143 (4.7)	9 (3.6)	2 (0.2)	2 (0.2)
Not applicable, I wouldn't do this anyway	259 (8.5)	11 (4.5)	24 (2.9)	24 (2.3)
Don't know	104 (3.4)	9 (3.6)	10 (1.2)	14 (1.4)
Prefer not to say	8 (0.3)	1 (0.4)	1 (0.1)	1 (0.1)

New screen

Imagine that tomorrow morning **you develop an unexplained rash with blisters**. You also learn that **you have come into contact with someone who might have monkeypox**.

Q15 How many days after the start of these symptoms would you:

Please give an approximate number if you are unsure. If you would not do this, please put 0.
Type your answer below

			General population	Savanta GBMSM	Grindr	Meta
Have sexual contact (from kissing to intercourse) with someone else	OPEN TEXT ENTRY – number [cap at 100]	ASK ONLY to those who answer 1-4 for similar question above	N=2021, M=15.7, SD=15.3, range 0 to 100.	N=190, M=16.2, SD=14.4, range 0 to 100.	N=746, M=20.4, SD=15.8, range 0 to 100.	N=947, M=20.0, SD=12.3, range 0 to 100.
Prefer not to say			469 (18.8)	26 (12.0)	44 (5.6)	44 (4.4)
Have sex without using a condom	OPEN TEXT ENTRY – number [cap at 100]	ASK ONLY to those who answer 1-4 for similar question above	N=1807, M=15.7, SD=16.8, range 0 to 100.	N=175, M=16.8, SD=18.9, range 0 to 100.	N=649, M=21.2, SD=19.5, range 0 to 100.	N=821, M=21.3, SD=18.0, range 0 to 100.
Prefer not to say			427 (19.1)	23 (11.6)	37 (5.4)	35 (4.1)

Contact sharing

For peer review only - http://bmjopen.bmj.com/site/about/guidelines.xhtml

New screen

People with suspected or confirmed monkeypox are being asked by public health officials to share contact details of people they have been in close contact with, so that they can be offered appropriate care and advice. Details of contacts are kept completely confidential.

ASK ALL

Q16 If **you** were asked for this information, would you try to share all the contact details of every …

RANDOMISE statements

Person who had been in your home (household members and visitors) in the last seven days

	General population	Savanta GBMSM	Grindr	Meta
Definitely would not	135 (4.4)	14 (5.7)	51 (6.1)	56 (5.4)
Probably would not	168 (5.5)	19 (7.7)	101 (12.2)	118 (11.4)
Not sure	392 (12.9)	30 (12.1)	97 (11.7)	120 (11.6)
Probably would	758 (24.9)	60 (24.3)	247 (29.7)	325 (31.4)
Definitely would	1552 (50.9)	120 (48.6)	331 (39.8)	414 (40.0)
Prefer not to say	45 (1.5)	4 (1.6)	4 (0.5)	3 (0.3)

Person you had sexual contact with (from kissing to intercourse) in the last seven days

	General population	Savanta GBMSM	Grindr	Meta
Definitely would not	169 (5.5)	17 (6.9)	49 (5.9)	54 (5.2)
Probably would not	162 (5.3)	16 (6.5)	59 (7.1)	67 (6.5)
Not sure	366 (12.0)	27 (10.9)	59 (7.1)	83 (8.0)
Probably would	629 (20.6)	56 (22.7)	236 (28.4)	282 (27.2)
Definitely would	1671 (54.8)	127 (51.4)	420 (50.5)	545 (52.6)
Prefer not to say	53 (1.7)	4 (1.6)	8 (1.0)	5 (0.5)

Place you had sexual contact with someone (from kissing to intercourse) in the last seven days

	General population	Savanta GBMSM	Grindr	Meta
Definitely would not	165 (5.4)	17 (6.9)	51 (6.1)	52 (5)
Probably would not	188 (6.2)	18 (7.3)	63 (7.6)	72 (6.9)
Not sure	415 (13.6)	28 (11.3)	72 (8.7)	91 (8.8)
Probably would	655 (21.5)	64 (25.9)	261 (31.4)	306 (29.5)
Definitely would	1559 (51.1)	116 (47)	375 (45.1)	510 (49.2)
Prefer not to say	68 (2.2)	4 (1.6)	9 (1.1)	5 (0.5)

Person you had skin-to-skin contact with (including hugging or kissing) in the last seven days

	General	Savanta	Grindr	Meta

For peer review only - http://bmjopen.bmj.com/site/about/guidelines.xhtml

	population	GBMSM		
Definitely would not	140 (4.6)	15 (6.1)	45 (5.4)	53 (5.1)
Probably would not	183 (6.0)	16 (6.5)	74 (8.9)	83 (8.0)
Not sure	352 (11.5)	25 (10.1)	74 (8.9)	116 (11.2)
Probably would	710 (23.3)	65 (26.3)	261 (31.4)	309 (29.8)
Definitely would	1621 (53.1)	122 (49.4)	373 (44.9)	472 (45.6)
Prefer not to say	44 (1.4)	4 (1.6)	4 (0.5)	3 (0.3)

Person you had shared bedding, towels, or clothes with in the last seven days

	General population	Savanta GBMSM	Grindr	Meta
Definitely would not	148 (4.9)	15 (6.1)	48 (5.8)	47 (4.5)
Probably would not	180 (5.9)	16 (6.5)	57 (6.9)	70 (6.8)
Not sure	352 (11.5)	26 (10.5)	69 (8.3)	81 (7.8)
Probably would	746 (24.5)	65 (26.3)	236 (28.4)	282 (27.2)
Definitely would	1574 (51.6)	122 (49.4)	415 (49.9)	551 (53.2)
Prefer not to say	50 (1.6)	3 (1.2)	6 (0.7)	5 (0.5)

Attitudes and beliefs

New screen

ASK ALL

Q17 How much do you agree or disagree with the following statements:

RANDOMISE statements

I wouldn't want to know the results of a monkeypox test

	General population	Savanta GBMSM	Grindr	Meta
Strongly disagree	1057 (34.7)	110 (44.5)	498 (59.9)	690 (66.6)
Disagree	963 (31.6)	63 (25.5)	235 (28.3)	243 (23.5)
Neither agree nor disagree	365 (12.0)	23 (9.3)	29 (3.5)	27 (2.6)
Agree	353 (11.6)	23 (9.3)	27 (3.2)	22 (2.1)
Strongly agree	240 (7.9)	23 (9.3)	35 (4.2)	51 (4.9)
Don't know	72 (2.4)	5 (2)	7 (0.8)	3 (0.3)

I would be worried what my friends or family would think about me if they thought I had monkeypox

	General population	Savanta GBMSM	Grindr	Meta
Strongly disagree	428 (14.0)	35 (14.2)	83 (10.0)	125 (12.1)
Disagree	624 (20.5)	43 (17.4)	114 (13.7)	147 (14.2)
Neither agree nor disagree	627 (20.6)	42 (17.0)	99 (11.9)	104 (10.0)

For peer review only - http://bmjopen.bmj.com/site/about/guidelines.xhtml

Agree	865 (28.4)	73 (29.6)	271 (32.6)	325 (31.4)
Strongly agree	402 (13.2)	52 (21.1)	263 (31.6)	333 (32.1)
Don't know	104 (3.4)	2 (0.8)	1 (0.1)	2 (0.2)

I would be worried about how colleagues / my employer would react if they thought I had monkeypox

	General population	Savanta GBMSM	Grindr	Meta
Strongly disagree	366 (12)	31 (12.6)	79 (9.5)	99 (9.6)
Disagree	508 (16.7)	40 (16.2)	95 (11.4)	112 (10.8)
Neither agree nor disagree	649 (21.3)	37 (15.0)	82 (9.9)	109 (10.5)
Agree	914 (30.0)	67 (27.1)	249 (30.0)	309 (29.8)
Strongly agree	462 (15.1)	64 (25.9)	316 (38.0)	391 (37.7)
Don't know	151 (5.0)	8 (3.2)	10 (1.2)	16 (1.5)

I don't want to have a monkeypox test result on my medical record

	General population	Savanta GBMSM	Grindr	Meta
Strongly disagree	541 (17.7)	50 (20.2)	161 (19.4)	229 (22.1)
Disagree	801 (26.3)	63 (25.5)	193 (23.2)	260 (25.1)
Neither agree nor disagree	837 (27.4)	58 (23.5)	183 (22.0)	205 (19.8)
Agree	494 (16.2)	42 (17.0)	142 (17.1)	151 (14.6)
Strongly agree	257 (8.4)	28 (11.3)	142 (17.1)	171 (16.5)
Don't know	120 (3.9)	6 (2.4)	10 (1.2)	20 (1.9)

An effective way to prevent the spread of monkeypox is for people who have symptoms to contact healthcare services

	General population	Savanta GBMSM	Grindr	Meta
Strongly disagree	67 (2.2)	7 (2.8)	16 (1.9)	19 (1.8)
Disagree	131 (4.3)	9 (3.6)	25 (3.0)	19 (1.8)
Neither agree nor disagree	459 (15.0)	24 (9.7)	58 (7.0)	64 (6.2)
Agree	1185 (38.9)	88 (35.6)	311 (37.4)	378 (36.5)
Strongly agree	1105 (36.2)	113 (45.7)	408 (49.1)	549 (53)
Don't know	103 (3.4)	6 (2.4)	13 (1.6)	7 (0.7)

I would be willing to contact a sexual health clinic if I thought I had monkeypox symptoms or had come into contact with someone who had monkeypox

	General population	Savanta GBMSM	Grindr	Meta
Strongly disagree	149 (4.9)	7 (2.8)	21 (2.5)	20 (1.9)
Disagree	291 (9.5)	20 (8.1)	24 (2.9)	17 (1.6)
Neither agree nor disagree	590 (19.3)	28 (11.3)	36 (4.3)	39 (3.8)
Agree	1047 (34.3)	73 (29.6)	260 (31.3)	247 (23.8)

For peer review only - http://bmjopen.bmj.com/site/about/guidelines.xhtml

Strongly agree	800 (26.2)	110 (44.5)	481 (57.9)	709 (68.4)
Don't know	173 (5.7)	9 (3.6)	9 (1.1)	4 (0.4)

New screen

ASK ALL

Q18 How much do you agree or disagree with the following statements:

RANDOMISE statements

If I had monkeypox symptoms, I wouldn't want to tell anyone as I don't want to self-isolate

	General population	Savanta GBMSM	Grindr	Meta
Strongly disagree	1055 (34.6)	79 (32.0)	291 (35.0)	374 (36.1)
Disagree	949 (31.1)	74 (30.0)	322 (38.7)	414 (40.0)
Neither agree nor disagree	431 (14.1)	33 (13.4)	98 (11.8)	119 (11.5)
Agree	365 (12.0)	32 (13.0)	72 (8.7)	78 (7.5)
Strongly agree	174 (5.7)	21 (8.5)	36 (4.3)	35 (3.4)
Don't know	76 (2.5)	8 (3.2)	12 (1.4)	16 (1.5)

If I had monkeypox symptoms, I wouldn't want to tell anyone so that others don't have to self-isolate

	General population	Savanta GBMSM	Grindr	Meta
Strongly disagree	909 (29.8)	71 (28.7)	260 (31.3)	305 (29.4)
Disagree	991 (32.5)	72 (29.1)	319 (38.4)	407 (39.3)
Neither agree nor disagree	468 (15.3)	37 (15.0)	122 (14.7)	143 (13.8)
Agree	396 (13)	35 (14.2)	86 (10.3)	114 (11.0)
Strongly agree	207 (6.8)	25 (10.1)	33 (4.0)	41 (4.0)
Don't know	79 (2.6)	7 (2.8)	11 (1.3)	26 (2.5)

Most people would self-isolate if they were told to

	General population	Savanta GBMSM	Grindr	Meta
Strongly disagree	132 (4.3)	14 (5.7)	84 (10.1)	90 (8.7)
Disagree	561 (18.4)	42 (17.0)	233 (28.0)	338 (32.6)
Neither agree nor disagree	634 (20.8)	51 (20.6)	180 (21.7)	222 (21.4)
Agree	1127 (37.0)	93 (37.7)	239 (28.8)	311 (30.0)
Strongly agree	461 (15.1)	32 (13.0)	51 (6.1)	36 (3.5)
Don't know	135 (4.4)	15 (6.1)	44 (5.3)	39 (3.8)

I have the support I need to self-isolate for 21 days

	General population	Savanta GBMSM	Grindr	Meta
Strongly disagree	173 (5.7)	26 (10.5)	142 (17.1)	132 (12.7)

For peer review only - http://bmjopen.bmj.com/site/about/guidelines.xhtml

Disagree	348 (11.4)	28 (11.3)	185 (22.3)	245 (23.6)
Neither agree nor disagree	458 (15.0)	41 (16.6)	129 (15.5)	127 (12.3)
Agree	1062 (34.8)	80 (32.4)	226 (27.2)	352 (34.0)
Strongly agree	942 (30.9)	66 (26.7)	133 (16.0)	168 (16.2)
Don't know	67 (2.2)	6 (2.4)	16 (1.9)	12 (1.2)

An effective way to prevent the spread of monkeypox is for people who have tested positive to self-isolate

	General population	Savanta GBMSM	Grindr	Meta
Strongly disagree	51 (1.7)	2 (0.8)	17 (2.0)	21 (2.0)
Disagree	107 (3.5)	13 (5.3)	16 (1.9)	34 (3.3)
Neither agree nor disagree	361 (11.8)	24 (9.7)	45 (5.4)	64 (6.2)
Agree	1018 (33.4)	68 (27.5)	319 (38.4)	362 (34.9)
Strongly agree	1424 (46.7)	132 (53.4)	416 (50.1)	543 (52.4)
Don't know	89 (2.9)	8 (3.2)	18 (2.2)	12 (1.2)

New screen

ASK ALL

Q19 How much do you agree or disagree with the following statements:

If I had to self-isolate because I had tested positive for monkeypox…

RANDOMISE statements

…I would lose touch with my friends and relatives

	General population	Savanta GBMSM	Grindr	Meta
Strongly disagree	567 (18.6)	44 (17.8)	151 (18.2)	180 (17.4)
Disagree	1039 (34.1)	78 (31.6)	320 (38.5)	408 (39.4)
Neither agree nor disagree	614 (20.1)	47 (19.0)	148 (17.8)	180 (17.4)
Agree	539 (17.7)	52 (21.1)	134 (16.1)	200 (19.3)
Strongly agree	225 (7.4)	19 (7.7)	61 (7.3)	65 (6.3)
Don't know	66 (2.2)	7 (2.8)	17 (2)	3 (0.3)

…it would have a severe impact on my family's wellbeing

	General population	Savanta GBMSM	Grindr	Meta
Strongly disagree	420 (13.8)	48 (19.4)	152 (18.3)	181 (17.5)
Disagree	740 (24.3)	58 (23.5)	279 (33.6)	350 (33.8)
Neither agree nor disagree	705 (23.1)	51 (20.6)	159 (19.1)	198 (19.1)
Agree	763 (25.0)	56 (22.7)	148 (17.8)	195 (18.8)

For peer review only - http://bmjopen.bmj.com/site/about/guidelines.xhtml

Strongly agree	338 (11.1)	21 (8.5)	81 (9.7)	99 (9.6)
Don't know	84 (2.8)	13 (5.3)	12 (1.4)	13 (1.3)

…it would have a negative impact on how much money I have

	General population	Savanta GBMSM	Grindr	Meta
Strongly disagree	498 (16.3)	52 (21.1)	132 (15.9)	184 (17.8)
Disagree	791 (25.9)	62 (25.1)	231 (27.8)	357 (34.5)
Neither agree nor disagree	566 (18.6)	27 (10.9)	99 (11.9)	153 (14.8)
Agree	694 (22.8)	56 (22.7)	162 (19.5)	169 (16.3)
Strongly agree	414 (13.6)	43 (17.4)	196 (23.6)	165 (15.9)
Don't know	87 (2.9)	7 (2.8)	11 (1.3)	8 (0.8)

…it would have a negative impact on my work

	General population	Savanta GBMSM	Grindr	Meta
Strongly disagree	545 (17.9)	52 (21.1)	76 (9.1)	98 (9.5)
Disagree	664 (21.8)	38 (15.4)	136 (16.4)	191 (18.4)
Neither agree nor disagree	622 (20.4)	49 (19.8)	100 (12)	136 (13.1)
Agree	697 (22.9)	55 (22.3)	260 (31.3)	325 (31.4)
Strongly agree	405 (13.3)	46 (18.6)	251 (30.2)	264 (25.5)
Don't know	117 (3.8)	7 (2.8)	8 (1.0)	22 (2.1)

…I would miss out on events and activities that I want to attend

	General population	Savanta GBMSM	Grindr	Meta
Strongly disagree	340 (11.1)	32 (13.0)	77 (9.3)	46 (4.4)
Disagree	471 (15.4)	33 (13.4)	106 (12.8)	107 (10.3)
Neither agree nor disagree	607 (19.9)	37 (15.0)	97 (11.7)	117 (11.3)
Agree	1097 (36.0)	84 (34.0)	354 (42.6)	457 (44.1)
Strongly agree	460 (15.1)	55 (22.3)	185 (22.3)	306 (29.5)
Don't know	75 (2.5)	6 (2.4)	12 (1.4)	3 (0.3)

Vaccination

New screen

People who have come into significant contact with someone who has tested positive for monkeypox are being asked to have a smallpox vaccine to reduce the risk of getting seriously ill. As monkeypox is caused by a virus similar to the one that causes smallpox, vaccines designed for smallpox are considered effective in preventing or reducing the severity of monkeypox.

For peer review only - http://bmjopen.bmj.com/site/about/guidelines.xhtml

Like all medicines, this vaccine can cause side effects, but not everyone gets them. The most common side effects are pain and itching at the injection site. Most side effects are mild and clear fully without any treatment within 7 days.

New screen

For each question, please select the answer that reflects your opinion. Do not worry if you do not know what the best answer might be or if you are not at all familiar with monkeypox or vaccination. We only ask that you try to give your answer based on what you think you know or what you would honestly decide to do in the situations described. If you are really not sure, please answer "don't know".

New screen

ASK ALL
Q20 How much do you agree or disagree with the following statements:

RANDOMISE statements

In general, vaccination is a good thing

	General population	Savanta GBMSM	Grindr	Meta
Strongly disagree	65 (2.1)	9 (3.6)	15 (1.8)	6 (0.6)
Disagree	119 (3.9)	4 (1.6)	5 (0.6)	3 (0.3)
Neither agree nor disagree	361 (11.8)	21 (8.5)	33 (4.0)	15 (1.4)
Agree	1019 (33.4)	59 (23.9)	189 (22.7)	142 (13.7)
Strongly agree	1400 (45.9)	148 (59.9)	584 (70.3)	869 (83.9)
Don't know	86 (2.8)	6 (2.4)	5 (0.6)	1 (0.1)

Most people like me will get a smallpox vaccination if advised

	General population	Savanta GBMSM	Grindr	Meta
Strongly disagree	82 (2.7)	5 (2.0)	24 (2.9)	15 (1.4)
Disagree	248 (8.1)	14 (5.7)	41 (4.9)	43 (4.2)
Neither agree nor disagree	533 (17.5)	31 (12.6)	93 (11.2)	122 (11.8)
Agree	1270 (41.6)	111 (44.9)	404 (48.6)	470 (45.4)
Strongly agree	717 (23.5)	69 (27.9)	223 (26.8)	365 (35.2)
Don't know	200 (6.6)	17 (6.9)	46 (5.5)	21 (2.0)

If I get a smallpox vaccination, I will be protected against monkeypox

	General population	Savanta GBMSM	Grindr	Meta
Strongly disagree	100 (3.3)	9 (3.6)	27 (3.2)	6 (0.6)
Disagree	385 (12.6)	19 (7.7)	71 (8.5)	64 (6.2)
Neither agree nor disagree	927 (30.4)	52 (21.1)	158 (19.0)	139 (13.4)

For peer review only - http://bmjopen.bmj.com/site/about/guidelines.xhtml

Agree	953 (31.2)	102 (41.3)	391 (47.1)	599 (57.8)
Strongly agree	250 (8.2)	29 (11.7)	117 (14.1)	190 (18.3)
Don't know	435 (14.3)	36 (14.6)	67 (8.1)	38 (3.7)

A smallpox vaccination could give me smallpox

	General population	Savanta GBMSM	Grindr	Meta
Strongly disagree	614 (20.1)	70 (28.3)	400 (48.1)	668 (64.5)
Disagree	931 (30.5)	65 (26.3)	277 (33.3)	264 (25.5)
Neither agree nor disagree	662 (21.7)	38 (15.4)	69 (8.3)	51 (4.9)
Agree	392 (12.9)	32 (13.0)	27 (3.2)	17 (1.6)
Strongly agree	136 (4.5)	9 (3.6)	8 (1.0)	6 (0.6)
Don't know	315 (10.3)	33 (13.4)	50 (6.0)	30 (2.9)

I might regret getting the smallpox vaccination if I later experienced side effects from it

	General population	Savanta GBMSM	Grindr	Meta
Strongly disagree	420 (13.8)	48 (19.4)	259 (31.2)	410 (39.6)
Disagree	825 (27.0)	74 (30.0)	350 (42.1)	421 (40.6)
Neither agree nor disagree	710 (23.3)	43 (17.4)	88 (10.6)	121 (11.7)
Agree	655 (21.5)	50 (20.2)	89 (10.7)	61 (5.9)
Strongly agree	268 (8.8)	19 (7.7)	31 (3.7)	17 (1.6)
Don't know	172 (5.6)	13 (5.3)	14 (1.7)	6 (0.6)

I am already immune to monkeypox

	General population	Savanta GBMSM	Grindr	Meta
Strongly disagree	676 (22.2)	77 (31.2)	269 (32.4)	366 (35.3)
Disagree	800 (26.2)	51 (20.6)	213 (25.6)	251 (24.2)
Neither agree nor disagree	532 (17.4)	30 (12.1)	106 (12.8)	132 (12.7)
Agree	228 (7.5)	24 (9.7)	57 (6.9)	99 (9.6)
Strongly agree	138 (4.5)	13 (5.3)	26 (3.1)	44 (4.2)
Don't know	676 (22.2)	52 (21.1)	160 (19.3)	144 (13.9)

I would be worried about experiencing side effects from a smallpox vaccination

	General population	Savanta GBMSM	Grindr	Meta
Strongly disagree	392 (12.9)	44 (17.8)	246 (29.6)	364 (35.1)
Disagree	793 (26.0)	72 (29.1)	294 (35.4)	407 (39.3)
Neither agree nor disagree	646 (21.2)	41 (16.6)	105 (12.6)	136 (13.1)
Agree	819 (26.9)	54 (21.9)	146 (17.6)	109 (10.5)
Strongly agree	278 (9.1)	28 (11.3)	23 (2.8)	18 (1.7)
Don't know	122 (4.0)	8 (3.2)	17 (2.0)	2 (0.2)

For peer review only - http://bmjopen.bmj.com/site/about/guidelines.xhtml

I would be worried that having a smallpox vaccine might make me infectious to others

	General population	Savanta GBMSM	Grindr	Meta
Strongly disagree	653 (21.4)	74 (30.0)	454 (54.6)	693 (66.9)
Disagree	1050 (34.4)	72 (29.1)	280 (33.7)	274 (26.4)
Neither agree nor disagree	605 (19.8)	33 (13.4)	46 (5.5)	32 (3.1)
Agree	378 (12.4)	31 (12.6)	18 (2.2)	15 (1.4)
Strongly agree	159 (5.2)	18 (7.3)	5 (0.6)	7 (0.7)
Don't know	205 (6.7)	19 (7.7)	28 (3.4)	15 (1.4)

People who are likely to come into high-risk contact with monkeypox should have a smallpox vaccine

	General population	Savanta GBMSM	Grindr	Meta
Strongly disagree	63 (2.1)	5 (2.0)	13 (1.6)	9 (0.9)
Disagree	158 (5.2)	15 (6.1)	8 (1.0)	5 (0.5)
Neither agree nor disagree	554 (18.2)	32 (13)	57 (6.9)	32 (3.1)
Agree	1134 (37.2)	89 (36)	256 (30.8)	234 (22.6)
Strongly agree	873 (28.6)	89 (36)	465 (56)	745 (71.9)
Don't know	268 (8.8)	17 (6.9)	32 (3.9)	11 (1.1)

New screen

ASK ALL

Q21 To the best of your knowledge, have you received a smallpox vaccine in 2022?

SINGLE CODE

Answer Options

	General population	Savanta GBMSM	Grindr	Meta
Yes	162 (5.3)	26 (10.5)	265 (31.9)	422 (40.7)
No. I was offered the vaccine, but didn't want to have it	116 (3.8)	9 (3.6)	11 (1.3)	16 (1.5)
No, I haven't been offered the vaccine	2609 (85.5)	202 (81.8)	536 (64.5)	587 (56.7)
Don't know / can't remember	163 (5.3)	10 (4.0)	19 (2.3)	11 (1.1)

New screen

ASK IF Q21=2,3,4

Q22 If you were advised by public health officials to have a smallpox vaccine **after coming into high-risk contact with someone who has monkeypox**, how likely would you be to have one?

For peer review only - http://bmjopen.bmj.com/site/about/guidelines.xhtml

SINGLE CODE

Answer Options

	General population, n=2888	Savanta GBMSM, n=221	Grindr, n=566	Meta, n=614
Definitely would not	115 (4.0)	6 (2.7)	16 (2.8)	10 (1.6)
Probably would not	139 (4.8)	15 (6.8)	13 (2.3)	4 (0.7)
Not sure	497 (17.2)	22 (10.0)	17 (3.0)	14 (2.3)
Probably would	814 (28.2)	48 (21.7)	95 (16.8)	51 (8.3)
Definitely would	1323 (45.8)	130 (58.8)	425 (75.1)	535 (87.1)

‡This question was not asked to people who had already had the smallpox vaccine in 2022, therefore total *n*s are different.

New screen

ASK IF Q21=2,3,4

Q23 Some people who have been in contact with someone who has monkeypox are being asked to self-isolate for 21 days. If having a smallpox vaccine means that you would have to self-isolate for less time, how likely would you be to get vaccinated?

SINGLE CODE

Answer Options

	General population, n=2888	Savanta GBMSM, n=221	Grindr, n=566	Meta, n=614
Definitely would not	114 (3.9)	5 (2.3)	17 (3.0)	9 (1.5)
Probably would not	128 (4.4)	9 (4.1)	17 (3.0)	8 (1.3)
Not sure	487 (16.9)	29 (13.1)	31 (5.5)	19 (3.1)
Probably would	822 (28.5)	45 (20.4)	92 (16.3)	55 (9.0)
Definitely would	1337 (46.3)	133 (60.2)	409 (72.3)	523 (85.2)

‡This question was not asked to people who had already had the smallpox vaccine in 2022, therefore total *n*s are different.

New screen

The smallpox vaccine is considered effective in preventing monkeypox or reducing the severity of monkeypox. Some people who are more likely to come into contact with monkeypox are being offered this vaccine.

ASK IF Q21=2,3,4

Q24 If you were offered a smallpox vaccine, how likely would you be to have one?

SINGLE CODE

Answer Options

	General population,	Savanta GBMSM,	Grindr, n=566	Meta, n=614

For peer review only - http://bmjopen.bmj.com/site/about/guidelines.xhtml

	n=2888	n=221		
Definitely would not	111 (3.8)	4 (1.8)	20 (3.5)	12 (2.0)
Probably would not	157 (5.4)	12 (5.4)	14 (2.5)	5 (0.8)
Not sure	518 (17.9)	21 (9.5)	20 (3.5)	21 (3.4)
Probably would	851 (29.5)	64 (29.0)	95 (16.8)	64 (10.4)
Definitely would	1251 (43.3)	120 (54.3)	417 (73.7)	512 (83.4)

‡This question was not asked to people who had already had the smallpox vaccine in 2022, therefore total *n*s are different.

New screen

ASK ALL
Q25. Do you have any further thoughts you would like to share with us about monkeypox, self-isolation, vaccines or anything else to do with monkeypox?

If you do not have anything to add, please put "None".

Type your answer below
OPEN END – require at least one alphabetic/numeric character

New screen

Sociodemographic variables

Now we are going to ask some questions about you. This survey is anonymous. This means that nobody can link you to your answers.

New screen

ASK ALL
D1A In the past month, **have you personally**:
Please select one option for each answer

Gone without enough food to eat

	General population	Savanta GBMSM	Grindr	Meta
Never	2006 (65.8)	171 (69.2)	670 (80.6)	938 (90.5)
Sometimes	663 (21.7)	48 (19.4)	121 (14.6)	85 (8.2)
Often	300 (9.8)	21 (8.5)	28 (3.4)	8 (0.8)
Don't know	63 (2.1)	7 (2.8)	7 (0.8)	3 (0.3)
Prefer not to say	18 (0.6)	0 (0.0)	5 (0.6)	2 (0.2)

Gone without an income

	General population	Savanta GBMSM	Grindr	Meta
Never	2014 (66)	172 (69.6)	653 (78.6)	890 (85.9)

For peer review only - http://bmjopen.bmj.com/site/about/guidelines.xhtml

Sometimes	600 (19.7)	43 (17.4)	105 (12.6)	106 (10.2)
Often	335 (11.0)	24 (9.7)	60 (7.2)	34 (3.3)
Don't know	69 (2.3)	6 (2.4)	2 (0.2)	2 (0.2)
Prefer not to say	32 (1.0)	2 (0.8)	11 (1.3)	4 (0.4)

Gone without fuel for heating or to cook food

	General population	Savanta GBMSM	Grindr	Meta
Never	2232 (73.2)	179 (72.5)	730 (87.8)	978 (94.4)
Sometimes	474 (15.5)	38 (15.4)	72 (8.7)	48 (4.6)
Often	255 (8.4)	25 (10.1)	22 (2.6)	6 (0.6)
Don't know	71 (2.3)	2 (0.8)	1 (0.1)	2 (0.2)
Prefer not to say	18 (0.6)	3 (1.2)	6 (0.7)	2 (0.2)

New screen

ASK ALL

D1B Last month, how difficult was it for you to cover your expenses and pay all your bills?

SINGLE CODE

	General population	Savanta GBMSM	Grindr	Meta
Not at all difficult	980 (32.1)	93 (37.7)	421 (50.7)	675 (65.2)
Somewhat difficult	1148 (37.6)	86 (34.8)	275 (33.1)	271 (26.2)
Very difficult	380 (12.5)	29 (11.7)	53 (6.4)	42 (4.1)
Extremely difficult	407 (13.3)	32 (13)	50 (6.0)	25 (2.4)
Don't know	55 (1.8)	1 (0.4)	2 (0.2)	2 (0.2)
Not applicable, I don't pay the bills in my household	53 (1.7)	5 (2.0)	22 (2.6)	10 (1.0)
Prefer not to say	27 (0.9)	1 (0.4)	8 (1)	11 (1.1)

New screen

ASK ALL

D2 How many people currently live in your household?

Please include yourself and all adults and children – including those not related to you

SINGLE CODE

	General population	Savanta GBMSM	Grindr	Meta
I live alone	663 (21.7)	101 (40.9)	358 (43.1)	380 (36.7)
2	1104 (36.2)	90 (36.4)	313 (37.7)	531 (51.3)

For peer review only - http://bmjopen.bmj.com/site/about/guidelines.xhtml

3 – 4	1055 (34.6)	42 (17.0)	131 (15.8)	103 (9.9)
5 – 6	204 (6.7)	13 (5.3)	25 (3.0)	15 (1.4)
7 +	24 (0.8)	1 (0.4)	4 (0.5)	7 (0.7)

New screen

ASK ALL
D3 Are you the parent / guardian of any dependent children?

Dependent children are those aged under 18 living in your household.
SINGLE CODE

	General population	Savanta GBMSM	Grindr	Meta
Yes, I have one or more dependent children	975 (32.0)	37 (15.0)	35 (4.2)	22 (2.1)
No, I don't have any dependent children	2075 (68.0)	210 (85.0)	796 (95.8)	1014 (97.9)

New screen

ASK ALL
D4. Have you ever provided voluntary care for a family member or friend who needs support due to old age, physical illness, disability, mental health problems, or addiction?
Please select one option
SINGLE CODE

	General population	Savanta GBMSM	Grindr	Meta
Yes, I currently do this	623 (20.4)	45 (18.2)	158 (19.0)	155 (15.0)
I have previously provided care for a friend/family member, but no longer do	897 (29.4)	68 (27.5)	223 (26.8)	315 (30.4)
I have never provided care for a family member/friend in this way	1424 (46.7)	121 (49)	422 (50.8)	541 (52.2)
Prefer not to say	106 (3.5)	13 (5.3)	28 (3.4)	25 (2.4)

New screen

ASK ALL
D5 Do you currently have any pets that live in your home?
MULTI CODE

For peer review only - http://bmjopen.bmj.com/site/about/guidelines.xhtml

	General population	Savanta GBMSM	Grindr	Meta
Yes, I have a dog or cat	1534 (50.3)	117 (47.4)	286 (34.4)	336 (32.4)
Yes, I have a rabbit, ferret, or rodent (e.g. rat, mouse, chinchilla, guinea pig)	195 (6.4)	7 (2.8)	7 (0.8)	10 (1.0)
Yes, I have another type of animal (e.g. reptile, fish, bird)	232 (7.6)	17 (6.9)	43 (5.2)	51 (4.9)
No, I don't have a pet [EXCLUSIVE]	1311 (43.0)	118 (47.8)	516 (62.1)	667 (64.4)

†Participants could select more than one item for this question, therefore column totals add to more than 100%.

New screen

ASK ALL

D6 What is your employment status?

SINGLE CODE

	General population	Savanta GBMSM	Grindr	Meta
Full time paid job (31+ hours)	1163 (38.1)	120 (48.6)	529 (63.7)	626 (60.4)
Part time paid job (<31 hours)	471 (15.4)	21 (8.5)	58 (7.0)	90 (8.7)
Doing paid work on a self-employed basis or within your own business	102 (3.3)	9 (3.6)	82 (9.9)	115 (11.1)
Student / On a government training programme (Nation Traineeship/Modern Apprenticeship)	93 (3.0)	7 (2.8)	35 (4.2)	32 (3.1)
Out of work	120 (3.9)	7 (2.8)	31 (3.7)	19 (1.8)
Looking after home / Homemaker	184 (6.0)	4 (1.6)	3 (0.4)	5 (0.5)
Retired	707 (23.2)	53 (21.5)	57 (6.9)	106 (10.2)
Disabled OR Long-term sick	155 (5.1)	21 (8.5)	25 (3.0)	28 (2.7)
Unpaid work for a business, community or voluntary organisation	27 (0.9)	1 (0.4)	6 (0.7)	11 (1.1)
Prefer not to say	28 (0.9)	4 (1.6)	5 (0.6)	4 (0.4)

New screen

ASK IF D6= CODES 1,2, 3, 4, or 9

For peer review only - http://bmjopen.bmj.com/site/about/guidelines.xhtml

D6A Are you a **frontline health or social care worker**? Please include any voluntary work

A frontline health or social care worker is someone who is directly involved in the care of patients or residents in long-stay care facilities (e.g. a care home), who has face-to-face contact with patients or clients, or who works in a laboratory, pathology or mortuary.

SINGLE CODE
FIX ORDER

	General population, n=1856	Savanta GBMSM, n=158	Grindr, n=710	Meta, n=874
Yes	355 (19.1)	23 (14.6)	94 (13.2)	125 (14.3)
No	1474 (79.4)	135 (85.4)	613 (86.3)	747 (85.5)
Prefer not to say	27 (1.5)	0 (0.0)	3 (0.4)	2 (0.2)

‡This question was only asked to those who reported that they were in full-time, part-time, self-employment, a student, or did unpaid or voluntary work, therefore total *n*s are different.

New screen

ASK IF D6= CODES 1, 2 OR 3
D6B If you needed to, are you able to work from home?
SINGLE CODE

	General population, n=1736	Savanta GBMSM, n=150	Grindr, n=669	Meta, n=831
Yes – full time	632 (36.4)	52 (34.7)	209 (31.2)	294 (35.4)
Yes – most of the time	260 (15.0)	26 (17.3)	136 (20.3)	196 (23.6)
Yes – but I have to go into my place of work once or twice a week	142 (8.2)	13 (8.7)	67 (10.0)	111 (13.4)
No – I cannot do my job from home	702 (40.4)	59 (39.3)	257 (38.4)	230 (27.7)

‡This question was only asked to those who reported that they were in full-time, part-time, or self-employment, therefore total *n*s are different.

New screen

ASK ALL
D7 What is the highest level of educational qualification you have received?
SINGLE CODE

	General population	Savanta GBMSM	Grindr	Meta
PhD/Doctorate	43 (1.4)	7 (2.8)	41 (4.9)	79 (7.6)

For peer review only - http://bmjopen.bmj.com/site/about/guidelines.xhtml

Masters	213 (7.0)	22 (8.9)	204 (24.5)	325 (31.4)
Bachelor's Degree or equivalent (Such as a NVQ level 5)	729 (23.9)	59 (23.9)	291 (35.0)	379 (36.6)
Higher education (Such as a HND or a NVQ level 4)	348 (11.4)	29 (11.7)	106 (12.8)	89 (8.6)
A level or equivalent (Such as Scottish Highers or NVQ level 3)	666 (21.8)	58 (23.5)	110 (13.2)	88 (8.5)
GCSE and below (Such as O level or an RSA Diploma)	747 (24.5)	53 (21.5)	57 (6.9)	63 (6.1)
Other qualifications (Such as NVQ level 1)	119 (3.9)	12 (4.9)	11 (1.3)	11 (1.1)
No qualifications	185 (6.1)	7 (2.8)	11 (1.3)	2 (0.2)

New screen

ASK ALL

D8 Which of the following categories would best describe your ethnicity?

SINGLE CODE

	General population	Savanta GBMSM	Grindr	Meta
White				
British/English/Welsh/Scottish/Northern Irish	2649 (86.9)	214 (86.6)	629 (75.7)	799 (77.1)
Irish	38 (1.2)	5 (2.0)	26 (3.1)	31 (3.0)
Gypsy, Traveller or Irish Traveller	7 (0.2)	0 (0.0)	1 (0.1)	1 (0.1)
Any other White background	71 (2.3)	13 (5.3)	90 (10.8)	131 (12.6)
Mixed/ Multiple ethnic groups				
White and Black Caribbean	18 (0.6)	1 (0.4)	7 (0.8)	4 (0.4)
White and Black African	11 (0.4)	4 (1.6)	1 (0.1)	3 (0.3)
White and Asian	24 (0.8)	1 (0.4)	3 (0.4)	9 (0.9)
Any other Mixed/ Multiple ethnic background	16 (0.5)	1 (0.4)	12 (1.4)	17 (1.6)
Asian or Asian British				
Indian	41 (1.3)	4 (1.6)	20 (2.4)	1 (0.1)
Pakistani	50 (1.6)	0 (0.0)	2 (0.2)	0 (0.0)
Bangladeshi	12 (0.4)	0 (0.0)	1 (0.1)	1 (0.1)
Chinese	14 (0.5)	1 (0.4)	7 (0.8)	7 (0.7)
Any other Asian background	12 (0.4)	0 (0.0)	4 (0.5)	5 (0.5)
Black or Black British				

For peer review only - http://bmjopen.bmj.com/site/about/guidelines.xhtml

African	26 (0.9)	1 (0.4)	5 (0.6)	2 (0.2)
Caribbean	32 (1.0)	0 (0.0)	8 (1.0)	4 (0.4)
Any other Black/ African/ Caribbean background	3 (0.1)	1 (0.4)	1 (0.1)	1 (0.1)
Other ethnic group				
Arab	7 (0.2)	0 (0.0)	3 (0.4)	1 (0.1)
Other	9 (0.3)	1 (0.4)	6 (0.7)	8 (0.8)
Don't know / Prefer not to say				
Don't know	2 (0.1)	0 (0.0)	0 (0.0)	1 (0.1)
Prefer not to say	8 (0.3)	0 (0.0)	5 (0.6)	10 (1.0)

New screen

ASK ALL

D9 Do you, or anyone else in your household have any long-standing illness, disability or infirmity?

Please select all that apply

MULTICODE

	General population	Savanta GBMSM	Grindr	Meta
Yes, I do	843 (27.6)	80 (32.4)	212 (25.5)	305 (29.4)
Yes, another household member does	391 (12.8)	21 (8.5)	88 (10.6)	115 (11.1)
No	1879 (61.6)	149 (60.3)	538 (64.7)	634 (61.2)
Prefer not to say	62 (2)	4 (1.6)	10 (1.2)	14 (1.4)

†Participants could select more than one item for this question, therefore column totals add to more than 100%.

New screen

ASK IF D9=1 (Yes, I do)

D9A Do you have any of the following health conditions?

Please select all that apply

MULTICODE

FIX ORDER

	General population, n=843	Savanta GBMSM, n=80	Grindr, n=212	Meta, n=305
Diabetes	184 (21.8)	21 (26.3)	38 (17.9)	49 (16.1)
Any type of cancer	57 (6.8)	4 (5.0)	10 (4.7)	14 (4.6)
Heart conditions	116 (13.8)	8 (10.0)	24 (11.3)	42 (13.8)
Respiratory conditions	191 (22.7)	15 (18.8)	27 (12.7)	59 (19.3)

For peer review only - http://bmjopen.bmj.com/site/about/guidelines.xhtml

(e.g. chronic obstructive pulmonary disease [COPD], asthma)				
Mental health	317 (37.6)	34 (42.5)	84 (39.6)	102 (33.4)
A skin condition (e.g. atopic dermatitis / eczema)	114 (13.5)	9 (11.3)	25 (11.8)	46 (15.1)
A condition that makes you much more likely to get infections (e.g. SCID, homozygous sickle cell)	20 (2.4)	0 (0.0)	7 (3.3)	7 (2.3)
A weakened immune system	110 (13)	8 (10.0)	24 (11.3)	38 (12.5)
Are taking medicine that weakens your immune system (e.g. steroid tablets, chemotherapy, or antiretroviral medications)	62 (7.4)	4 (5.0)	15 (7.1)	24 (7.9)
HIV/AIDS	6 (0.7)	12 (15.0)	52 (24.5)	117 (38.4)
Other, please specify	183 (21.7)	11 (13.8)	48 (22.6)	60 (19.7)
Prefer not to say	46 (5.5)	5 (6.3)	11 (5.2)	8 (2.6)

†Participants could select more than one item for this question, therefore column totals add to more than 100%.

‡This question was only asked to those who reported that had a chronic illness, therefore total *n*s are different.

New screen

ASK ALL
D9B Are you pregnant?
SINGLE CODE

	General population	Savanta GBMSM	Grindr	Meta
Yes	38 (1.2)	1 (0.4)	0 (0.0)	0 (0.0)
No	3001 (98.4)	245 (99.2)	830 (99.9)	1035 (99.9)
Prefer not to say	11 (0.4)	1 (0.4)	1 (0.1)	1 (0.1)

New screen

ASK ALL
D9C Have you ever taken PrEP (pre-exposure prophylaxis) for HIV?
SINGLE CODE

For peer review only - http://bmjopen.bmj.com/site/about/guidelines.xhtml

	General population	Savanta GBMSM	Grindr	Meta
Yes	42 (1.4)	28 (11.3)	402 (48.4)	487 (47.0)
No	2993 (98.1)	216 (87.4)	427 (51.4)	547 (52.8)
Prefer not to say	15 (0.5)	3 (1.2)	2 (0.2)	2 (0.2)

New screen

ASK ALL

D10 To the best of your knowledge, have you received a vaccine for…

Hepatitis A

	General population	Savanta GBMSM	Grindr	Meta
Yes	531 (17.4)	87 (35.2)	549 (66.1)	773 (74.6)
No. I was offered the vaccine, but didn't want to have it	128 (4.2)	12 (4.9)	7 (0.8)	4 (0.4)
No, I haven't been offered the vaccine	1579 (51.8)	94 (38.1)	129 (15.5)	125 (12.1)
Don't know / can't remember	812 (26.6)	54 (21.9)	146 (17.6)	134 (12.9)

Smallpox (before 2022)

	General population	Savanta GBMSM	Grindr	Meta
Yes	512 (16.8)	51 (20.6)	144 (17.3)	218 (21)
No. I was offered the vaccine, but didn't want to have it	117 (3.8)	12 (4.9)	5 (0.6)	7 (0.7)
No, I haven't been offered the vaccine	1666 (54.6)	133 (53.8)	477 (57.4)	595 (57.4)
Don't know / can't remember	755 (24.8)	51 (20.6)	205 (24.7)	216 (20.8)

Covid-19 (two doses or more)

	General population	Savanta GBMSM	Grindr	Meta
Yes	2557 (83.8)	218 (88.3)	796 (95.8)	1023 (98.7)
No. I was offered the vaccine, but didn't want to have it	242 (7.9)	16 (6.5)	27 (3.2)	11 (1.1)
No, I haven't been offered the vaccine	189 (6.2)	9 (3.6)	5 (0.6)	0 (0.0)
Don't know / can't remember	62 (2.0)	4 (1.6)	3 (0.4)	2 (0.2)

New screen

For peer review only - http://bmjopen.bmj.com/site/about/guidelines.xhtml

ASK ALL
D11 What is your marital status?
SINGLE CODE

	General population	Savanta GBMSM	Grindr	Meta
Single, never married	741 (24.3)	123 (49.8)	470 (56.6)	402 (38.8)
Married / civil partnership	1382 (45.3)	62 (25.1)	149 (17.9)	290 (28)
Separated	69 (2.3)	3 (1.2)	29 (3.5)	21 (2.0)
Divorced	319 (10.5)	14 (5.7)	57 (6.9)	39 (3.8)
Widowed	136 (4.5)	1 (0.4)	5 (0.6)	24 (2.3)
Partnered / in a relationship	393 (12.9)	43 (17.4)	111 (13.4)	251 (24.2)
Prefer not to say	10 (0.3)	1 (0.4)	10 (1.2)	9 (0.9)

New screen

The following questions are sensitive. You don't need to answer if you don't want to. If you don't want to answer, please select "prefer not to say".

New screen

ASK ALL
D12A How many **male** sexual partners have you had…?

By "sexual partner" we mean any form of genital contact, including touching, kissing, and intercourse.

Please give an approximate number if you are unsure. If you have not had any male sexual partners in this time, please put 0.
Type your answer below

		General population	Savanta GBMSM	Grindr	Meta
In the last 3 weeks (21 days)	OPEN TEXT ENTRY – number [cap at 100]	N=2418, M=0.6, SD=3.6, range 0 to 100.	N=210, M=1.1, SD=2.0, range 0 to 14.	N=787, M=2.9, SD=5.2, range 0 to 75.	N=982, M=2.5, SD=4.7, range 0 to 100.
In the last 3 months (90 days)	OPEN TEXT ENTRY – number	N=2418, M=0.8, SD=8.9, range 0 to 400.	N=210, M=2.8, SD=7.0, range 0 to 50.	N=787, M=9.1, SD=23.8, range 0 to 400.	N=982, M=8.9, SD=26.2, range 0 to 400.

For peer review only - http://bmjopen.bmj.com/site/about/guidelines.xhtml

	[cap at 400]				
Prefer not to say		632 (20.7)	37 (15.0)	44 (5.3)	54 (5.2)

New screen

ASK ALL

D12B How many **female** sexual partners have you had…?

By "sexual partner" we mean any form of genital contact, including touching, kissing, and intercourse.

Please give an approximate number if you are unsure. If you have not had any female sexual partners in this time, please put 0.

Type your answer below

		General population	Savanta GBMSM	Grindr	Meta
In the last 3 weeks (21 days)	OPEN TEXT ENTRY – number [cap at 100]	N=2442, M=0.4, SD=3.1, range 0 to 100.	N=207, M=0.2, SD=0.8, range 0 to 100.	N=792, M=0.1, SD=0.6, range 0 to 10.	N=992, M=0.0, SD=0.4, range 0 to 9.
In the last 3 months (90 days)	OPEN TEXT ENTRY – number [cap at 400]	N=2442, M=0.8, SD=9.2, range 0 to 400.	N=207, M=2.2, SD=27.8, range 0 to 400.	N=792, M=0.2, SD=1.6, range 0 to 30.	N=992, M=0.1, SD=1.0, range 0 to 28.
Prefer not to say		608 (19.9)	40 (16.2)	39 (4.7)	44 (4.2)

New screen

Thanks for completing the survey. If you would like any more information on monkeypox, please see https://www.nhs.uk/conditions/monkeypox/.

Please press the Finish button to complete the survey.
Many thanks for completing this survey, your views are extremely important to us.

If you have any further comments or feedback, please use the box below.

For peer review only - http://bmjopen.bmj.com/site/about/guidelines.xhtml

Supplementary materials 2. Data preparation

Some participants (3.8%, n=196) did not provide their postcode and were therefore missing data about region and index of multiple deprivation. While individual items about financial hardship had small amounts of missing data (2.0% to 3.8%, n=105 to 197), as items were summed to give a scale, missingness on the resulting scale would have been higher (7.9%, n=410). There were no apparent patterns to the missing data. We used multiple imputation to impute missing values for region, index of multiple deprivation and financial hardship items. Socio-demographic variables (age, occupation of highest earner, number of people in household, having a dependent child, employment status, education, ethnicity, marital status, and region, index of multiple deprivation and financial hardship where available) were used to predict missing values using a Markov Chain Monte Carlo (MCMC) method using linear regressions with ten iterations, to give five imputations.

We computed a single variable to indicate perceived susceptibility and severity from three items. For these items, "don't know" was coded as the mid-point on the scale. We then multiplied responses together (range 1 to 125) and took the cubic root to give a scale from 1 (lowest perceived susceptibility and severity) to 5 (highest perceived susceptibility and severity). We also asked participants how much they agreed that they were already immune to mpox. This item was recoded into a binary item to show perceived immunity to mpox (strongly agree and agree, vs neither agree nor disagree, disagree, strongly disagree, and don't know).

To create a single variable for perceived knowledge, we used three items (Cronbach's α=0.74). Responses for these items were given on a five-point scale from "strongly disagree" to "strongly agree" We recoded each item as a binary item (strongly agree and agree [1], vs neither agree nor disagree, disagree, strongly disagree, and don't know [0]), then summed responses to give a scale from 0 (lowest perceived knowledge) to 3 (highest perceived knowledge).

For knowledge about mpox symptoms, for each symptom selected, we coded participants as being correct (1, symptom listed on NHS mpox website) or incorrect (0, symptom not listed on NHS mpox website). Scores were then summed to give a scale from 0 (no selected symptoms were mpox symptoms) to 4 (all selected symptoms were mpox symptoms); responses of "don't know" were coded as 0. A single variable denoting understanding of transmission was computed. It is unknown whether mpox can be caught from pet animals

For peer review only - http://bmjopen.bmj.com/site/about/guidelines.xhtml

(but pet animals can catch mpox from humans (1)), therefore we did not include this item in analyses. We coded answers as correct (1) or incorrect (0; answers of "don't know" coded as incorrect), and summed items to give a score from 0 (lowest knowledge about transmission) to 6 (highest knowledge about transmission).

To reduce the number of variables included in regression models, we used dimension reduction techniques. We conducted principal components analysis, using direct oblimin rotation as items may have been correlated, on items potentially associated with self-isolation (ten items), help seeking (six items), and vaccination (eight items) separately. The number of factors was determined using a scree plot. The item with the highest loading on to a component was then included in regression analyses. Results of the principal components analyses are reported in the supplementary materials 3.

References

1. Seang S, Burrel S, Todesco E, Leducq V, Monsel G, Le Pluart D, et al. Evidence of human-to-dog transmission of monkeypox virus. The Lancet. 2022;400(10353):658-9.

For peer review only

Supplementary materials 3. Results of principal components analyses.

Self-isolation

A scree plot suggested three components underlying factors potentially associated with self-isolation. Table 1 shows loadings of items onto each component.

Table 1. Loadings of items measuring factors potentially associated with self-isolation onto components identified (only loadings over ± .4 are shown).

Item	Component 1 loading	Component 2 loading	Component 3 loading
If I had mpox symptoms, I wouldn't want to tell anyone as I don't want to self-isolate*		-.864	
If I had mpox symptoms, I wouldn't want to tell anyone so that others don't have to self-isolate		-.853	
Most people would self-isolate if they were told to*			.810
I have the support I need to self-isolate for 21 days			.729
An effective way to prevent the spread of mpox is for people who have tested positive to self-isolate		.544	.426
If I had to self-isolate because I had tested positive for mpox...I would lose touch with my friends and relatives	.627		
If I had to self-isolate because I had tested positive for mpox...it would have a severe impact on my family's wellbeing	.684		
If I had to self-isolate because I had tested positive for mpox...it would have a negative impact on how much money I have	.754		
If I had to self-isolate because I had tested positive for mpox...it would have a negative impact on my work*	.789		
If I had to self-isolate because I had tested positive for mpox...I would miss out on events and activities that I want to attend	.646		

Rotation method: oblimin with Kaiser normalization. Rotation converged in 10 iterations.
*Included in regression analyses

Help seeking

A scree plot suggested two components underlying factors potentially associated with seeking help immediately. Table 2 shows loadings of items onto each component.

Table 2. Loadings of items measuring factors potentially associated with self-isolation onto components identified (only loadings over ± .4 are shown).

Item	Component 1 loading	Component 2 loading
I wouldn't want to know the results of a mpox test		-.526
I would be worried what my friends or family would think about me if they thought I had mpox *	.867	
I would be worried about how colleagues / my employer would react if they thought I had mpox	.845	
I don't want to have a mpox test result on my medical record	.727	
An effective way to prevent the spread of mpox is for people who have symptoms to contact healthcare services		.772
I would be willing to contact a sexual health clinic if I thought I had mpox symptoms or had come into contact with someone who had mpox *		.784

Rotation method: oblimin with Kaiser normalization. Rotation converged in 2 iterations.
*Included in regression analyses

For peer review only - http://bmjopen.bmj.com/site/about/guidelines.xhtml

Vaccination

A scree plot suggested two components underlying factors potentially associated with vaccination. Table 3 shows loadings of items onto each component.

Table 3. Loadings of items measuring factors potentially associated with vaccination onto components identified (only loadings over ± .4 are shown).

Item	Component 1 loading	Component 2 loading
In general, vaccination is a good thing		.627
Most people like me will get a smallpox vaccination if advised		.737
If I get a smallpox vaccination, I will be protected against mpox *		.746
A smallpox vaccination could give me smallpox	.801	
I might regret getting the smallpox vaccination if I later experienced side effects from it	.789	
I would be worried about experiencing side effects from a smallpox vaccination	.793	
I would be worried that having a smallpox vaccine might make me infectious to others*	.843	
People who are likely to come into high-risk contact with mpox should have a smallpox vaccine		.744

Rotation method: oblimin with Kaiser normalization. Rotation converged in 4 iterations.
*Included in regression analyses

For peer review only

Supplementary materials 4. Influence of motivational messages on behavioural outcomes.

Table 1. Influence of motivational messaging on behavioural outcomes, in the general population sample.

			General population, n (%)				
			Risk + perceived necessity/efficacy, n=762	Risk + perceived benefits, n=768	Risk + perceived costs, n=762	Control, n=758	p-value
**Self-isolation for 21 days**	Sum of intention if case and high-risk contact	2 (lowest intention) to 10 (highest intention)	M=8.0, SD=2.2	M=7.9, SD=2.2	M=7.8, SD=2.2	M=8.1, SD=2.1	0.07
**Help seeking**	Any action that involves contacting a healthcare professional (by phone or in person)	Would not seek help or would seek help, but would "wait and see" for a day or two to see if symptoms resolved	362 (47.5)	413 (53.8)	382 (50.1)	391 (51.6)	0.10
		Would seek help immediately	400 (52.5)	355 (46.2)	380 (49.9)	367 (48.4)	
**Contact behaviour**	Have sexual contact with others†	Less than, same as, or more than normal	125 (20.5)	146 (22.6)	148 (23.8)	148 (24.2)	0.41
		Completely stop	485 (79.5)	501 (77.4)	473 (76.2)	464 (75.8)	
**Contact sharing**	Sexual contacts	1 (definitely would not) to 5 (definitely would)	N=748, M=4.2, SD=1.2	N=756, M=4.2, SD=1.2	N=746, M=4.2, SD=1.1	N=747, M=4.1, SD=1.1	0.98
**Vaccination intention**	Smallpox vaccine "if advised"	1 (definitely would not) to 5 (definitely would)	N=723, M=4.0, SD=1.1	N=726, M=4.0, SD=1.1	N=721, M=4.0, SD=1.1	N=718, M=4.0, SD=1.1	0.81

†Answers of "don't know", "prefer not to say" and "not applicable, I wouldn't do this anyway" were coded as missing, therefore total *n* was substantially lower (n=2490).

For peer review only

Table 2. Influence of motivational messaging on behavioural outcomes, by GBMSM sample.

			Savanta GBMSM, n (%)			Grindr, n (%)			Meta, n (%)		
			Motivational message, n=115	Control, n=132	p-value	Motivational message, n=404	Control, n=427	p-value	Motivational message, n=529	Control, n=507	p-value
**Self-isolation for 21 days**	2 (lowest intention) to 10 (highest intention)		M=7.7, SD=2.4	M=8.0, SD=2.1	0.22	M=7.2, SD=2.4	M=7.3, SD=2.5	0.74	M=7.1, SD=2.3	M=7.5, SD=2.2	0.005
**Help seeking**	Any action that involves contacting a healthcare professional (by phone or in person)	Would not seek help or would seek help, but would "wait and see" for a day or two to see if symptoms resolved	59 (51.3)	66 (50.0)	0.84	189 (46.8)	199 (46.6)	0.96	187 (35.3)	204 (40.2)	0.10
		Would seek help immediately	56 (48.7)	66 (50.0)		215 (53.2)	228 (53.4)		342 (64.7)	303 (59.8)	
**Contact behaviour**	Have sexual contact with others†	Less than, same as, or more than normal	26 (26.5)	19 (16.1)	0.06	28 (7.3)	41 (10.1)	0.17	38 (7.5)	31 (6.4)	0.50
		Completely stop	72 (73.5)	99 (83.9)		355 (92.7)	366 (89.9)		469 (92.5)	453 (93.6)	
**Contact sharing**	Sexual contacts	1 (definitely would not) to 5 (definitely would)	N=113, M=4.0, SD=1.2	N=130, M=4.1, SD=1.2	0.54	N=402, M=4.1, SD=1.2	N=421, M=4.1, SD=1.2	0.72	N=526, M=4.1, SD=1.2	N=505, M=4.2, SD=1.1	0.04
**Vaccination intention**	Smallpox vaccine "if advised"‡	1 (definitely would not) to 5 (definitely would)	N=103, M=4.3, SD=1.0	N=118, M=4.2, SD=1.0	0.36	N=282, M=4.6, SD=0.9	N=284, M=4.5, SD=1.0	0.18	N=304, M=4.7, SD=0.9	N=310, M=4.8, SD=0.6	0.03

†Answers of "don't know", "prefer not to say" and "not applicable, I wouldn't do this anyway" were coded as missing, therefore total *n*s were substantially lower (Savanta GBMSM, n=216; Grindr, n=790; Meta, n=991).
‡Not asked to people who reported that they had had the smallpox vaccine in 2022, therefore total *n*s were substantially lower (Savanta GBMSM, n=221; Grindr, n=566; Meta, n=614).

For peer review only - http://bmjopen.bmj.com/site/about/guidelines.xhtml

Supplementary materials 5. Full results of regressions with intention to seek help immediately.

Table 1. Associations between intending to seek help immediately and socio-demographic characteristics and motivational message, by sample. Variables were entered into the logistic regression model in blocks (block 1: socio-demographic variables and motivational message, block 2: psychological factors, block 3: help-seeking specific factors). Results for block 3, using pooled estimates are reported.

Participant characteristics	Level	General population		Grindr	
		aOR (95% CI)	*p*-value	aOR (95% CI)	*p*-value
Gender	Male (including trans man)	Ref	-	-	-
	Female (including trans woman)	1.10 (0.93 to 1.30)	0.25	-	-
Sexual orientation	Straight or heterosexual	Ref	-	-	-
	Gay, lesbian, bisexual, or queer	1.15 (0.84 to 1.58)	0.39	-	-
Age	Range 18 to 98 years	1.012 (1.006 to 1.019)	<0.001*	1.02 (1.01 to 1.04)	0.006
	Quadratic term, (age – mean)^2	1.0002 (0.9999 to 1.0005)	0.24	1.0001 (0.9992 to 1.0011)	0.80
Region	Midlands (East and West)	Ref	-	Ref	-
	South (East, West, East of England)	1.10 (0.87 to 1.40)	0.40	1.01 (0.50 to 2.04)	0.97
	North (East, West, Yorkshire and the Humber)	0.90 (0.71 to 1.15)	0.41	1.53 (0.75 to 3.13)	0.24
	London	0.90 (0.65 to 1.25)	0.54	1.15 (0.59 to 2.26)	0.67
	Devolved nations (Scotland, Wales, and Northern Ireland)	0.83 (0.62 to 1.13)	0.24	1.04 (0.47 to 2.28)	0.93
Dependent child in household	No	Ref	-	Ref	-
	Yes	0.96 (0.78 to 1.17)	0.68	1.58 (0.71 to 3.51)	0.26
Employment status	Not working	Ref	-	Ref	-
	Working	1.12 (0.87 to 1.43)	0.39	0.98 (0.57 to 1.68)	0.94
Frontline health or social care worker	No	Ref	-	Ref	-
	Yes	1.05 (0.81 to 1.36)	0.73	1.91 (1.15 to 3.18)	0.01
Need to leave home for work	Do not need to leave home for work	Ref	-	Ref	-
	Need to leave home for work	1.05 (0.84 to 1.31)	0.65	0.94 (0.64 to 1.37)	0.75
Education	GCSE/vocational/A-level/No formal qualifications	Ref	-	Ref	-
	Degree or higher (Bachelors, Masters, PhD)	0.87 (0.72 to 1.04)	0.12	0.94 (0.66 to 1.34)	0.74
Ethnicity	White British	Ref	-	Ref	-
	White other	1.26 (0.82 to 1.92)	0.29	1.03 (0.65 to 1.65)	0.89
	Black, Asian, other minoritized ethnicity	0.90 (0.66 to 1.22)	0.48	0.95 (0.55 to 1.63)	0.85
Marital status	Not partnered	Ref	-	Ref	-
	Partnered	1.09 (0.87 to 1.36)	0.44	0.81 (0.53 to 1.22)	0.31
Live alone	Live with someone else	Ref	-	Ref	-
	Live alone	1.10 (0.85 to 1.41)	0.48	0.79 (0.55 to 1.15)	0.23
Own chronic illness	None	Ref	-	Ref	-
	Present	1.11 (0.92 to 1.34)	0.28	1.28 (0.88 to 1.88)	0.20
Ever taken PrEP for HIV	No	-	-	Ref	-
	Yes	-	-	0.89 (0.62 to 1.28)	0.54
Vaccinated for smallpox in 2022	Not vaccinated	-	-	Ref	-
	Vaccinated	-	-	0.85 (0.57 to 1.27)	0.43
Index of multiple	Deciles (1st = most deprived, 10th	1.00 (0.97 to 1.03)	0.93	1.05 (0.98 to 1.13)	0.16

For peer review only

deprivation	= least deprived)				
Socio-economic grade	ABC1	Ref	-	Ref	-
	C2DE	0.94 (0.79 to 1.13)	0.53	0.66 (0.41 to 1.07)	0.09
Financial hardship	4 (lowest hardship) to 13 (most hardship)	0.96 (0.92 to 1.00)	0.07	0.99 (0.90 to 1.09)	0.87
Total number of sexual partners (male and female) in last three weeks	0	Ref	-	-	-
	1	1.09 (0.88 to 1.34)	0.45	-	-
	2 to 4	1.09 (0.73 to 1.62)	0.68	-	-
	5 or more	0.78 (0.38 to 1.64)	0.52	-	-
	Prefer not to say	1.09 (0.86 to 1.37)	0.49	-	-
Number of male sexual partners in last three weeks	0	-	-	Ref	-
	1	-	-	1.01 (0.63 to 1.62)	0.95
	2 to 4	-	-	1.01 (0.66 to 1.56)	0.95
	5 to 9	-	-	0.84 (0.47 to 1.49)	0.55
	10 or more	-	-	1.06 (0.51 to 2.17)	0.88
	Prefer not to say	-	-	0.93 (0.43 to 2.03)	0.86
Motivational message	Perceived risk of illness and necessity and efficacy of the response	1.16 (0.93 to 1.44)	0.19	-	-
	Perceived risk of illness and benefits of the response	0.96 (0.77 to 1.20)	0.71	-	-
	Perceived risk of illness and low perceived costs of response	1.05 (0.84 to 1.31)	0.68	-	-
	Control	Ref	-	-	-
Motivational message	All motivational components	-	-	0.86 (0.63 to 1.17)	0.34
	Control	-	-	Ref	-

*$p \leq 0.001$

For peer review only

Table 2. Associations between intending to seek help immediately and psychological and contextual factors, by sample. Variables were entered into the logistic regression model in blocks (block 1: socio-demographic variables and motivational message, block 2: psychological factors, block 3: help-seeking specific factors). Results for block 3, using pooled estimates are reported.

Factor	Level	General population aOR (95% CI)	*p*-value	Grindr aOR (95% CI)	*p*-value
Amount heard about mpox	I have not seen or heard anything (1) to I have seen or heard a lot (3)	1.05 (0.87 to 1.25)	0.63	0.96 (0.67 to 1.37)	0.83
Worry about mpox	Not at all worried (1) to extremely worried (4)	1.05 (0.90 to 1.23)	0.52	1.17 (0.84 to 1.63)	0.36
Perceived risk of mpox to oneself	No risk at all (1) to very high risk (5)	0.85 (0.74 to 0.98)	0.02	0.90 (0.72 to 1.14)	0.38
Perceived risk of mpox to people in UK	No risk at all (1) to very high risk (5)	1.04 (0.90 to 1.19)	0.62	1.37 (1.07 to 1.76)	0.01
Perceived susceptibility and severity	Lowest (1) to highest (5)	1.07 (0.94 to 1.23)	0.32	1.03 (0.77 to 1.36)	0.86
I am already immune to mpox	Strongly disagree, disagree, neither agree nor disagree, don't know	Ref	-	Ref	-
	Strongly agree and agree	0.95 (0.72 to 1.26)	0.73	0.69 (0.40 to 1.19)	0.18
People who catch mpox usually make a full recovery, even if they do not receive any treatment	Strongly disagree (1) to strongly agree (5)	0.87 (0.78 to 0.96)	0.006	0.84 (0.69 to 1.03)	0.09
My personal behaviour has an impact on how mpox spreads	Strongly disagree (1) to strongly agree (5)	1.05 (0.98 to 1.13)	0.19	1.07 (0.92 to 1.25)	0.37
My life has been negatively affected by changes made in response to the mpox outbreak	Strongly disagree (1) to strongly agree (5)	1.00 (0.91 to 1.10)	0.99	1.00 (0.85 to 1.17)	0.99
The risks of mpox are being exaggerated	Strongly disagree (1) to strongly agree (5)	0.83 (0.76 to 0.91)	<0.001*	0.94 (0.77 to 1.13)	0.48
Mpox is only a risk to men who are gay, bisexual or have sex with men	Strongly disagree (1) to strongly agree (5)	0.95 (0.88 to 1.03)	0.22	0.85 (0.72 to 1.01)	0.06
Perceived knowledge	Lowest (0) to highest (3)	0.97 (0.89 to 1.06)	0.54	0.99 (0.80 to 1.23)	0.93
Knowledge of mpox symptoms	Identified no symptoms (0) to identified four symptoms (4)	1.03 (0.96 to 1.09)	0.44	1.13 (0.98 to 1.30)	0.09
Knowledge of mpox transmission	Lowest (0) to highest (6)	0.95 (0.90 to 1.01)	0.12	1.03 (0.89 to 1.20)	0.69
I would be worried what my friends or family would think about me if they thought I had mpox	Strongly disagree (1) to strongly agree (5)	0.94 (0.88 to 1.00)	0.06	0.85 (0.75 to 0.97)	0.01
I would be willing to contact a sexual health clinic if I thought I had mpox symptoms or had come into contact with someone who had mpox	Strongly disagree (1) to strongly agree (5)	1.25 (1.16 to 1.34)	<.001*	1.57 (1.29 to 1.91)	<.001*

$^{*}p \leq 0.001$

Supplementary materials 6. Full results of regressions with smallpox vaccination.

Table 1. Associations between vaccine uptake (general population sample: actual and intended, GBMSM: actual, actual and intended) and socio-demographic characteristics and motivational message, by sample. Variables were entered into the logistic regression model in blocks (block 1: socio-demographic variables and motivational message, block 2: psychological factors, block 3: vaccination specific factors). Results for block 3, using pooled estimates are reported.

Participant characteristics	Level	General population		Grindr			
		Vaccinated in 2022 or would be vaccinated if advised		Vaccinated in 2022		Vaccinated in 2022 or would be vaccinated if advised	
		aOR (95% CI)	*p*-value	aOR (95% CI)	*p*-value	aOR (95% CI)	*p*-value
Gender	Male (including trans man)	Ref	-	-	-	-	-
	Female (including trans woman)	0.93 (0.75 to 1.13)	0.46	-	-	-	-
Sexual orientation	Straight or heterosexual	Ref	-	-	-	-	-
	Gay, lesbian, bisexual, or queer	1.26 (0.85 to 1.88)	0.26	-	-	-	-
Age	Range 18 to 98 years	1.015 (1.007 to 1.023)	<0.001*	1.00 (0.98 to 1.02)	0.81	0.98 (0.94 to 1.02)	0.30
	Quadratic term, $(\text{age} - \text{mean})^2$	1.0007 (1.0002 to 1.0011)	0.002	1.000 (0.998 to 1.001)	0.48	0.999 (0.997 to 1.001)	0.29
Region	Midlands (East and West)	Ref	-	Ref	-	Ref	-
	South (East, West, East of England)	0.82 (0.61 to 1.11)	0.20	0.93 (0.43 to 2.03)	0.86	1.05 (0.22 to 5.12)	0.95
	North (East, West, Yorkshire and the Humber)	0.77 (0.57 to 1.04)	0.09	1.21 (0.51 to 2.87)	0.67	0.48 (0.09 to 2.61)	0.40
	London	0.79 (0.54 to 1.17)	0.24	2.30 (1.04 to 5.11)	0.04	0.68 (0.13 to 3.64)	0.65
	Devolved nations (Scotland, Wales, and Northern Ireland)	0.64 (0.44 to 0.93)	0.02	0.82 (0.31 to 2.13)	0.68	1.91 (0.23 to 16.11)	0.55
Dependent child in household	No	Ref	-	Ref	-	Ref	-
	Yes	0.85 (0.67 to 1.08)	0.19	1.35 (0.49 to 3.71)	0.56	1.11 (0.09 to 13.72)	0.94
Employment status	Not working	Ref	-	Ref	-	Ref	-
	Working	1.34 (0.98 to 1.83)	0.07	1.22 (0.61 to 2.45)	0.57	2.06 (0.42 to 10.12)	0.37
Frontline health or social care worker	No	Ref	-	Ref	-	Ref	-
	Yes	1.46 (1.07 to 1.99)	0.02	0.41 (0.20 to 0.81)	0.01	5.38 (0.90 to 32.19)	0.07
Need to leave home for work	Do not need to leave home for work	Ref	-	Ref	-	Ref	-
	Need to leave home for work	0.56 (0.43 to 0.73)	<0.001*	0.99 (0.61 to 1.60)	0.96	0.28 (0.08 to 1.06)	0.06
Education	GCSE/vocational/A-level/No formal qualifications	Ref	-	Ref	-	Ref	-
	Degree or higher (Bachelors, Masters, PhD)	1.18 (0.94 to 1.47)	0.16	0.78 (0.49 to 1.26)	0.31	1.56 (0.58 to 4.19)	0.38
Ethnicity	White British	Ref	-	Ref	-	Ref	-

For peer review only - http://bmjopen.bmj.com/site/about/guidelines.xhtml
For peer review only

	White other	0.53 (0.33 to 0.85)	0.009	1.27 (0.72 to 2.26)	0.40	0.59 (0.15 to 2.26)	0.44
	Black, Asian, other minoritized ethnicity	0.81 (0.57 to 1.15)	0.24	1.52 (0.78 to 2.99)	0.22	0.41 (0.10 to 1.69)	0.22
Marital status	Not partnered	Ref	-	Ref	-	Ref	-
	Partnered	1.02 (0.79 to 1.33)	0.86	0.74 (0.43 to 1.25)	0.26	2.35 (0.66 to 8.33)	0.19
Live alone	Live with someone else	Ref	-	Ref	-	Ref	-
	Live alone	0.94 (0.69 to 1.27)	0.68	1.30 (0.81 to 2.09)	0.28	1.09 (0.40 to 2.96)	0.86
Own chronic illness	None	Ref	-	Ref	-	Ref	-
	Present	1.07 (0.85 to 1.36)	0.57	1.67 (1.02 to 2.74)	0.04	1.12 (0.39 to 3.25)	0.83
Ever taken PrEP for HIV	No	-	-	Ref	-	Ref	-
	Yes	-	-	8.95 (5.61 to 14.28)	<0.001*	3.41 (1.11 to 10.46)	0.03
Index of multiple deprivation	Deciles (1st = most deprived, 10th = least deprived)	1.00 (0.96 to 1.04)	0.89	0.95 (0.86 to 1.05)	0.28	0.94 (0.78 to 1.13)	0.52
Socio-economic grade	ABC1	Ref	-	Ref	-	Ref	-
	C2DE	0.83 (0.67 to 1.02)	0.08	1.53 (0.82 to 2.87)	0.19	1.39 (0.41 to 4.67)	0.60
Financial hardship	4 (lowest hardship) to 13 (most hardship)	0.95 (0.90 to 0.99)	0.02	1.03 (0.91 to 1.18)	0.62	0.95 (0.73 to 1.24)	0.71
Total number of sexual partners (male and female) in last three weeks	0	Ref	-	-	-	-	-
	1	1.05 (0.80 to 1.37)	0.72	-	-	-	-
	2 to 4	1.33 (0.81 to 2.18)	0.26	-	-	-	-
	5 or more	1.62 (0.64 to 4.09)	0.30	-	-	-	-
	Prefer not to say	0.70 (0.54 to 0.93)	0.01	-	-	-	-
Number of male sexual partners in last three weeks	0	-	-	Ref	-	Ref	-
	1	-	-	1.33 (0.69 to 2.57)	0.40	1.12 (0.33 to 3.77)	0.85
	2 to 4	-	-	1.70 (0.96 to 3.03)	0.07	1.78 (0.56 to 5.65)	0.33
	5 to 9	-	-	2.40 (1.17 to 4.95)	0.02	2.14 (0.34 to 13.33)	0.42
	10 or more	-	-	1.53 (0.65 to 3.62)	0.33	3.80 (0.28 to 52.03)	0.32
	Prefer not to say	-	-	1.21 (0.39 to 3.73)	0.74	0.72 (0.15 to 3.47)	0.68
Motivational message	Perceived risk of illness and necessity and efficacy of the response	0.92 (0.70 to 1.20)	0.53	-	-	-	-
	Perceived risk of illness and benefits of the response	1.15 (0.87 to 1.50)	0.32	-	-	-	-
	Perceived risk of illness and low perceived costs of response	1.02 (0.78 to 1.34)	0.89	-	-	-	-
	Control	Ref	-	-	-	-	-
Motivational message	All motivational components	-	-	0.91 (0.61 to 1.35)	0.63	1.02 (0.43 to 2.39)	0.97
	Control	-	-	Ref	-	Ref	-

*$p \leq 0.001$

For peer review only

Table 2. Associations between vaccine uptake (general population sample: actual and intended, GBMSM: actual, actual and intended) and psychological and contextual factors, by sample. Variables were entered into the logistic regression model in blocks (block 1: socio-demographic variables and motivational message, block 2: psychological factors, block 3: vaccination specific factors). Results for block 3, using pooled estimates are reported.

Factor	Level	General population<br>Vaccinated in 2022 or would be vaccinated if advised		Grindr<br>Vaccinated in 2022		Vaccinated in 2022 or would be vaccinated if advised	
		aOR (95% CI)	*p*-value	aOR (95% CI)	*p*-value	aOR (95% CI)	*p*-value
Amount heard about mpox	I have not seen or heard anything (1) to I have seen or heard a lot (3)	0.93 (0.75 to 1.15)	0.50	1.74 (1.09 to 2.78)	0.02	0.56 (0.21 to 1.55)	0.27
Worry about mpox	Not at all worried (1) to extremely worried (4)	1.49 (1.24 to 1.80)	<0.001*	1.00 (0.66 to 1.52)	0.99	4.33 (1.69 to 11.07)	0.002
Perceived risk of mpox to oneself	No risk at all (1) to very high risk (5)	0.99 (0.84 to 1.17)	0.92	1.25 (0.93 to 1.67)	0.13	1.19 (0.63 to 2.27)	0.59
Perceived risk of mpox to people in UK	No risk at all (1) to very high risk (5)	1.19 (1.00 to 1.40)	0.05	0.85 (0.62 to 1.16)	0.31	0.93 (0.43 to 2.00)	0.85
Perceived susceptibility and severity	Lowest (1) to highest (5)	1.31 (1.11 to 1.55)	0.001*	0.85 (0.60 to 1.21)	0.37	0.90 (0.40 to 2.05)	0.81
I am already immune to mpox	Strongly disagree, disagree, neither agree nor disagree, don't know	Ref	-	Ref	-	Ref	-
	Strongly agree and agree	1.30 (0.93 to 1.83)	0.12	8.83 (4.52 to 17.22)	<0.001*	1.30 (0.27 to 6.36)	0.74
People who catch mpox usually make a full recovery, even if they do not receive any treatment	Strongly disagree (1) to strongly agree (5)	1.01 (0.89 to 1.14)	0.90	1.20 (0.94 to 1.53)	0.15	0.51 (0.27 to 0.94)	0.03
My personal behaviour has an impact on how mpox spreads	Strongly disagree (1) to strongly agree (5)	1.00 (0.91 to 1.09)	0.99	0.99 (0.80 to 1.22)	0.92	0.94 (0.63 to 1.39)	0.74
My life has been negatively affected by changes made in response to the mpox outbreak	Strongly disagree (1) to strongly agree (5)	0.93 (0.84 to 1.04)	0.23	0.96 (0.79 to 1.17)	0.70	0.97 (0.63 to 1.51)	0.90
The risks of mpox are being exaggerated	Strongly disagree (1) to strongly agree (5)	0.76 (0.68 to 0.85)	<0.001*	0.78 (0.61 to 1.00)	0.05	0.45 (0.27 to 0.75)	0.002
Mpox is only a risk to men who are gay, bisexual or have sex with men	Strongly disagree (1) to strongly agree (5)	0.97 (0.88 to 1.07)	0.56	0.84 (0.67 to 1.04)	0.11	1.05 (0.66 to 1.65)	0.85
Perceived knowledge	Lowest (0) to highest (3)	1.03 (0.92 to 1.14)	0.64	1.25 (0.93 to 1.67)	0.14	0.78 (0.43 to 1.43)	0.42
Knowledge of mpox symptoms	Identified no symptoms (0) to identified four symptoms (4)	1.02 (0.94 to 1.10)	0.65	1.01 (0.83 to 1.21)	0.95	0.96 (0.67 to 1.38)	0.82
Knowledge of mpox transmission	Lowest (0) to highest (6)	1.04 (0.96 to 1.11)	0.34	0.98 (0.80 to 1.19)	0.82	1.05 (0.71 to 1.55)	0.82
If I get a smallpox vaccination, I will be protected against mpox	Strongly disagree (1) to strongly agree (5)	1.44 (1.29 to 1.61)	<0.001*	1.33 (1.06 to 1.66)	0.01	3.25 (1.97 to 5.35)	<0.001*

For peer review only - http://bmjopen.bmj.com/site/about/guidelines.xhtml

I would be worried that having a smallpox vaccine might make me infectious to others	Strongly disagree (1) to strongly agree (5)	0.64 (0.58 to 0.71)	<0.001*	0.63 (0.46 to 0.87)	0.005	0.44 (0.26 to 0.76)	0.003

*$p \leq 0.001$

For peer review only

STROBE 2007 (v4) Statement—Checklist of items that should be included in reports of *cross-sectional studies*

Section/Topic	Item #	Recommendation	Reported on page #
**Title and abstract**	1	(*a*) Indicate the study's design with a commonly used term in the title or the abstract	3
		(*b*) Provide in the abstract an informative and balanced summary of what was done and what was found	3
**Introduction**			
Background/rationale	2	Explain the scientific background and rationale for the investigation being reported	5-7
Objectives	3	State specific objectives, including any prespecified hypotheses	7
**Methods**			
Study design	4	Present key elements of study design early in the paper	7-8
Setting	5	Describe the setting, locations, and relevant dates, including periods of recruitment, exposure, follow-up, and data collection	7-8
Participants	6	(*a*) Give the eligibility criteria, and the sources and methods of selection of participants	7-8
Variables	7	Clearly define all outcomes, exposures, predictors, potential confounders, and effect modifiers. Give diagnostic criteria, if applicable	8-12, supplementary materials 1
Data sources/ measurement	8*	For each variable of interest, give sources of data and details of methods of assessment (measurement). Describe comparability of assessment methods if there is more than one group	8-12, supplementary materials 1
Bias	9	Describe any efforts to address potential sources of bias	7-14
Study size	10	Explain how the study size was arrived at	7-8, 12
Quantitative variables	11	Explain how quantitative variables were handled in the analyses. If applicable, describe which groupings were chosen and why	12-14, supplementary materials 2
Statistical methods	12	(*a*) Describe all statistical methods, including those used to control for confounding	12-14
		(*b*) Describe any methods used to examine subgroups and interactions	12-14
		(*c*) Explain how missing data were addressed	12-14, supplementary materials 2

For peer review only

		(*d*) If applicable, describe analytical methods taking account of sampling strategy	12-14
		(*e*) Describe any sensitivity analyses	N/A
**Results**			
Participants	13*	(a) Report numbers of individuals at each stage of study—eg numbers potentially eligible, examined for eligibility, confirmed eligible, included in the study, completing follow-up, and analysed	14-15
		(b) Give reasons for non-participation at each stage	N/A
		(c) Consider use of a flow diagram	Considered
Descriptive data	14*	(a) Give characteristics of study participants (eg demographic, clinical, social) and information on exposures and potential confounders	14-15
		(b) Indicate number of participants with missing data for each variable of interest	Supplementary materials 1 and 2
Outcome data	15*	Report numbers of outcome events or summary measures	16-17, 20, 23, 25, supplementary materials 4
Main results	16	(*a*) Give unadjusted estimates and, if applicable, confounder-adjusted estimates and their precision (eg, 95% confidence interval). Make clear which confounders were adjusted for and why they were included	15-26, supplementary materials 5 and 6
		(*b*) Report category boundaries when continuous variables were categorized	12-14, supplementary materials 2
		(*c*) If relevant, consider translating estimates of relative risk into absolute risk for a meaningful time period	N/A
Other analyses	17	Report other analyses done—eg analyses of subgroups and interactions, and sensitivity analyses	N/A
**Discussion**			
Key results	18	Summarise key results with reference to study objectives	26-30
Limitations	19	Discuss limitations of the study, taking into account sources of potential bias or imprecision. Discuss both direction and magnitude of any potential bias	30
Interpretation	20	Give a cautious overall interpretation of results considering objectives, limitations, multiplicity of analyses, results from similar studies, and other relevant evidence	26-31
Generalisability	21	Discuss the generalisability (external validity) of the study results	26-31
**Other information**			

For peer review only

Funding	22	Give the source of funding and the role of the funders for the present study and, if applicable, for the original study on which the present article is based	32

*Give information separately for cases and controls in case-control studies and, if applicable, for exposed and unexposed groups in cohort and cross-sectional studies.

Note: An Explanation and Elaboration article discusses each checklist item and gives methodological background and published examples of transparent reporting. The STROBE checklist is best used in conjunction with this article (freely available on the Web sites of PLoS Medicine at http://www.plosmedicine.org/, Annals of Internal Medicine at http://www.annals.org/, and Epidemiology at http://www.epidem.com/). Information on the STROBE Initiative is available at www.strobe-statement.org.

For peer review only

BMJ Open

Did mpox knowledge, attitudes, and beliefs affect intended behaviour in the general population and men who are gay, bisexual, and who have sex with men? An online cross-sectional survey in the UK

Journal:	*BMJ Open*
Manuscript ID	bmjopen-2022-070882.R2
Article Type:	Original research
Date Submitted by the Author:	04-Sep-2023
Complete List of Authors:	Smith, Louise; King's College London<br>Potts, Henry; University College London, Institute of Health Informatics<br>Brainard, Julii; University of East Anglia, Norwich Medical School<br>May, Tom; University of Bristol<br>Oliver, Isabel; UK Health Security Agency<br>Amlôt, Richard; UK Health Security Agency<br>Yardley, Lucy; University of Bristol<br>Rubin, G James; King's College London
**Primary Subject Heading**:	Public health
Secondary Subject Heading:	Health policy
Keywords:	Public health < INFECTIOUS DISEASES, PUBLIC HEALTH, SEXUAL MEDICINE

SCHOLARONE™
Manuscripts

BMJ

I, the Submitting Author has the right to grant and does grant on behalf of all authors of the Work (as defined in the below author licence), an exclusive licence and/or a non-exclusive licence for contributions from authors who are: i) UK Crown employees; ii) where BMJ has agreed a CC-BY licence shall apply, and/or iii) in accordance with the terms applicable for US Federal Government officers or employees acting as part of their official duties; on a worldwide, perpetual, irrevocable, royalty-free basis to BMJ Publishing Group Ltd ("BMJ") its licensees and where the relevant Journal is co-owned by BMJ to the co-owners of the Journal, to publish the Work in this journal and any other BMJ products and to exploit all rights, as set out in our licence.

The Submitting Author accepts and understands that any supply made under these terms is made by BMJ to the Submitting Author unless you are acting as an employee on behalf of your employer or a postgraduate student of an affiliated institution which is paying any applicable article publishing charge ("APC") for Open Access articles. Where the Submitting Author wishes to make the Work available on an Open Access basis (and intends to pay the relevant APC), the terms of reuse of such Open Access shall be governed by a Creative Commons licence – details of these licences and which Creative Commons licence will apply to this Work are set out in our licence referred to above.

Other than as permitted in any relevant BMJ Author's Self Archiving Policies, I confirm this Work has not been accepted for publication elsewhere, is not being considered for publication elsewhere and does not duplicate material already published. I confirm all authors consent to publication of this Work and authorise the granting of this licence.

For peer review only

Did mpox knowledge, attitudes, and beliefs affect intended behaviour in the general population and men who are gay, bisexual, and who have sex with men? An online cross-sectional survey in the UK

For peer review only

Louise E Smith (0000-0002-1277-2564),[1,2] PhD, Henry WW Potts (0000-0002-6200-8804),[3] PhD, Julii Brainard (0000-0002-5272-7995),[2,4] PhD, Tom May (0000-0003-3077-523X),[5,6,7] Isabel Oliver (0000-0002-6106-1734),[5,9] PhD, Richard Amlôt (0000-0003-3481-6588),[2,5,9] PhD, Lucy Yardley (0000-0002-3853-883X),[5,7,8] PhD, G James Rubin,[1,2] PhD

1 King's College London, Institute of Psychiatry, Psychology and Neuroscience

2 NIHR Health Protection Research Unit in Emergency Preparedness and Response

3 University College London, Institute of Health Informatics

4 University of East Anglia, Norwich Medical School

5 NIHR Health Protection Research Unit in Behavioural Science and Evaluation, University of Bristol

6 University of Bristol, Bristol Medical School

7 University of Bristol, School of Psychological Science

8 University of Southampton, School of Psychology

9 UK Health Security Agency, Chief Scientific Officer's Group

Corresponding author: Louise E Smith, Research Fellow. Department of Psychological Medicine, King's College London, Weston Education Centre, Cutcombe Road, London, SE5 9RJ, UK. Email: louise.e.smith@kcl.ac.uk

For peer review only - http://bmjopen.bmj.com/site/about/guidelines.xhtml

Henry WW Potts, Professor of Health Informatics. Institute of Health Informatics, 222 Euston Road, London, NW1 2DA, UK.

Julii Brainard, Senior Research Associate. Norwich Medical School, University of East Anglia, Norwich, Norfolk, NR4 7TJ, UK.

Tom May, Research Fellow, NIHR Health Protection Research Unit in Behavioural Science and Evaluation, Bristol Medical School, University of Bristol, Bristol, BS8 1TU, UK.

Isabel Oliver, Chief Scientific Officer, UK Health Security Agency, 17 Smith Square, London, SW1P 3HX, UK.

Richard Amlôt, Interim Deputy Director Behavioural Science and Insights, UK Health Security Agency, 17 Smith Square, London, SW1P 3HX, UK.

Lucy Yardley, Professor of Health Psychology. School of Psychological Science, University of Bristol, 12A Priory Rd, Bristol BS8 1TU, UK.

G James Rubin, Director, NIHR Health Protection Research Unit in Emergency Preparedness and Response, King's College London, Weston Education Centre, Cutcombe Road, London, SE5 9RJ, UK.

For peer review only

ABSTRACT

Objectives: To investigate rates of mpox beliefs, knowledge, and intended behaviours in the general population and in gay, bisexual or other men who have sex with men (GBMSM), and factors associated with intended behaviours. To test the impact of motivational messages (vs a factual control) on intended behaviours.

Design: Cross-sectional online survey including a nested randomised controlled trial.

Setting: Data collected 5 September to 6 October 2022.

Participants: Participants were aged 18 years and over and lived in the UK (general population). In addition, GBMSM were male, and gay, bisexual or had sex with men. The general population sample was recruited through a market research company. GBMSM were recruited through a market research company, the dating app Grindr, and targeted adverts on Meta (Facebook and Instagram).

Main outcome measures: Intention to self-isolate, seek medical help, stop all sexual contact, share details of recent sexual contacts, and accept vaccination.

Results: Socio-demographic characteristics differed by sample. There was no effect of very brief motivational messaging on behavioural intentions. Respondents from Grindr and Meta were more likely to intend to seek help immediately, completely stop sexual behaviour and be vaccinated or intend to be vaccinated, but being less likely to intend to self-isolate (*p*s<0.001). In the general population sample, intending to carry out protective behaviours was generally associated with being female, older, having less financial hardship, greater worry, higher perceived risk to others, and higher perceived susceptibility to and severity of mpox (*p*s<0.001). There were fewer associations with behaviours in the Grindr sample, possibly due to reduced power.

Conclusions: GBMSM were more likely to intend to enact protective behaviours, except for self-isolation. This may reflect targeted public health efforts and engagement with this group. Associations with socio-economic factors suggests that providing financial support may encourage people to engage with protective behaviours.

Word count: 298/300

Key words: adherence; self-isolation; symptoms; testing; mpox; psychological factors

For peer review only

For peer review only - http://bmjopen.bmj.com/site/about/guidelines.xhtml

STRENGTHS AND LIMITATIONS OF THIS STUDY

- A strength of this study is that it collected data from four large samples, including the general population and men who are gay, bisexual, or have sex with men.
- Data collection occurred over a short period (5 September to 6 October 2022) during the mpox outbreak.
- Limitations include that responses may have been affected by social desirability or recall bias, although the anonymous nature of the survey should mitigate this somewhat.
- Socio-demographic characteristics differed by sample, with participants recruited from Grindr and Meta being more likely to be working, highly educated, of higher socio-economic grade, and have less financial hardship.
- We measured behavioural intentions which may differ from actual behaviours, but factors associated with intentions should still be validly identified.

For peer review only

For peer review only - http://bmjopen.bmj.com/site/about/guidelines.xhtml

INTRODUCTION

Mpox (also known as monkeypox) is an orthopox virus that causes fever, headache, exhaustion, swollen glands and aches (joint, muscle, back), followed by a rash with blisters.[1] It spreads from person to person through touching clothing, bedding or towels used by someone with mpox rash, touching mpox skin blisters or scabs, and through the coughs or sneezes of someone with mpox rash (droplet transmission).[1] Since May 2022, there has been a multi-country outbreak of mpox in non-endemic countries.[2] Estimates from the World Health Organization (WHO; data up to 9 August 2023), indicate that there have been over 89,000 cases in 113 countries, resulting in 152 deaths.[3] Most cases have been in men who are gay, bisexual, or have sex with men, with close human skin-to skin contact (including sexual) being the primary driver of transmission.[4] The UK is the eighth country most affected by this mpox outbreak with 3771 cases.[3] Within the UK, most cases have been identified in England (with 69% of English cases in London). Almost all (99%) cases were men; English cases had a median age of 36 years.[5] The peak of the epidemic was seen in June and July 2022, with case numbers falling since the end of July.[6]

In the UK, people who thought they might have mpox were asked to call a sexual health clinic, to stay at home (self-isolate) and avoid close contact with other people.[1] Suspected cases were tested for mpox, and confirmed cases were asked to self-isolate for up to 21 days and engage with contact tracing.[7] Cases, their close contacts, and those most likely to be exposed to mpox were advised to be vaccinated with modified vaccinia Ankara vaccine – which offers cross-protection – to reduce transmission and prevent severe illness.[8, 9] While similar public health actions are now familiar to the public as a result of the COVID-19 pandemic, research conducted during that pandemic indicates that engagement with uptake of testing and self-isolation was sub-optimal in some groups.[10-12]

Various theories have guided research into the psychological factors associated with the uptake of health behaviours. One such theory is the Protection Motivation Theory (PMT), which states that people's intention to carry out a protective behaviour is influenced by their appraisal of the threat (perceived susceptibility to and severity of, e.g. mpox) and the coping response (perceived effectiveness of and self-efficacy for, e.g. testing, self-isolation, contact tracing, vaccination).[13] During the COVID-19 pandemic, psychological, and socio-demographic factors associated with testing and self-isolating included: higher perceived risk of COVID-19, knowledge of transmission modes, higher perceived effectiveness of protective behaviours, believing that your behaviour had an impact on transmission, and

For peer review only - http://bmjopen.bmj.com/site/about/guidelines.xhtml

belief that others in the same position would also self-isolate, being female, and having less financial hardship.[10-12, 14] Generally speaking, engagement with protective behaviours was associated with being older, apart from uptake of lateral flow testing, which was higher in younger people.[10, 11] Factors associated with COVID-19 vaccine uptake (completed and intended) included perceiving vaccination to be safe and necessary, perceiving COVID-19 to be more severe, and thinking that others would also be vaccinated.[15-18] Perception of side effects is one of the most common reasons given for refusing vaccination.[19] Historically, smallpox vaccines have been associated with severe adverse effects.[8] While vaccines currently licensed are associated with fewer severe adverse effects, this may affect people's intention to be vaccinated.

At the time of writing, few scientific studies have investigated behaviour during the 2022 mpox epidemic. While most have investigated vaccination acceptability, few have investigated engagement with a contact tracing system. Those studies that have been done suggest that knowledge of transmission modes is incomplete.[20-22] Greater agreement with vaccination for mpox was associated with perceiving the virus as more dangerous and virulent and higher worry about mpox in a survey of members of the Saudi Arabian general public.[23] In Dutch GBMSM, willingness to accept a vaccine was associated with being more worried about getting mpox, perceiving a higher risk of mpox, perceiving mpox to be more severe, thinking that vaccination is important, thinking that the vaccine was effective, and greater social norms for vaccination.[24] Another study also conducted in the Netherlands found that vaccination intention in GBMSM was associated with higher worry about mpox, knowing someone who had mpox, and being single but dating or in an open/polyamorous relationship.[25] This study also investigated self-isolation intention for 21 days, finding that higher intentions were associated with thinking that mpox had more problematic consequences, and lower education.[25] A study conducted in the UK found that agreement with self-isolation was associated with not having completed a higher education degree, not being employed, and identifying as having a disability.[26] There was no difference in agreement between GBMSM and those who were not GBMSM. Further research is needed to investigate how psychological factors may affect engagement with public health measures (isolating, testing, contact tracing, vaccination) put in place to control the spread of the mpox outbreak in the UK.

Official communications are vital during new and emerging outbreaks, and serve to inform the public about the threat, the public health response, and behaviours that people should

For peer review only - http://bmjopen.bmj.com/site/about/guidelines.xhtml

engage with in order to protect themselves and others.[27] Messages based on theories of health behaviours, such as the PMT,[13] may therefore increase engagement with protective behaviours. For example, research suggests that COVID-19 vaccination intention increased when communications emphasised the safety and effectiveness of the vaccine and using social norms interventions (e.g. asking people to "join the millions" being vaccinated).[28] While findings relating to messages emphasising the benefits of vaccination to oneself and others were mixed, there is some evidence that the influence of these messages may be most evident in strongly hesitant groups.

Decreasing rates of incidence of HIV in England [29] suggest that efforts to promote safer sexual practices in GBMSM have been successful. Existing communication channels and protocols to prevent HIV risk, may have led to increased knowledge and awareness about responsible sexual practices in GBMSM. During the mpox outbreak, concerted efforts were made by public health agencies (e.g. the UK Health Security Agency) to disseminate accurate scientific information about mpox to GBMSM in collaboration with community-based organisations and charities, the dating app Grindr, and organisers of Pride events,[30] building on existing communication channels.[31] Therefore, knowledge and beliefs about mpox may be different in GBMSM and the general population. For example, it is likely that perceived worry and risk are higher in GBMSM – the population most affected by the mpox outbreak – than the general population.

There are few quantitative studies investigating mpox knowledge, attitudes, and beliefs in the UK during the 2022 outbreak. Studies investigating the effectiveness of messages that could be used in official communications are particularly important. Socio-demographic characteristics associated with intended uptake of protective behaviours can help to identify groups that may benefit from increased messaging and support to adhere to public health guidelines. Psychological factors, such as knowledge, attitudes, and beliefs, that are associated with intention to enact protective behaviours can provide insights into potentially modifiable factors that could be included in official messaging. Collecting this information forms the start of an evidence base for policy decisions. In this study, we recruited a general population sample and three GBMSM samples from: a market research company, Grindr, and Meta (Facebook and Instagram). We investigated knowledge, attitudes and beliefs about mpox, and intentions for key behaviours that could prevent the spread of mpox (self-isolation, help seeking [as the advised route into testing], sexual contact behaviour when symptomatic, contact sharing, vaccination). We used an experimental approach to investigate

For peer review only - http://bmjopen.bmj.com/site/about/guidelines.xhtml

the impact of different brief communication approaches (promoting perceived susceptibility to and severity of illness / necessity and efficacy of the response / benefits of the response / low perceived costs of response) on intended behaviour. Psychological and socio-demographic factors associated with intended uptake of behaviours were also investigated.

METHOD

Design

Online cross-sectional survey conducted by Savanta, a Market Research Society (MRS) Company Partner. Data were collected between 5 September and 6 October 2022.

Participants

Eligibility criteria for the general population sample were living in the UK and being aged 18 years or over. For the GBMSM samples, additional criteria were being a man and identifying as being gay, bisexual, or having sex with men.

Recruitment for the general population sample used quota sampling, a standard opinion polling method that allows for rapid data collection. Members of Savanta's specialist research panel (n=150,000 across proprietary panels; people who have signed up to complete online surveys) were sent the survey link. Quota sampling uses pre-determined targets, based on pre-selected socio-demographic characteristics (quotas) that match the national population. Participants who belong to a quota that has already been filled are prevented from completing the survey. Therefore, response rate is not an accurate measure of response bias in quota samples. For this study, quotas were based on age, gender, socio-economic grade and Government Office Region, and reflected targets based on 2020 mid-year estimates.[32]

We recruited three GBMSM samples (Savanta, Grindr, Meta [Facebook and Instagram]). A "boost" sample of 250 GBMSM was recruited by Savanta, using the same quota sampling (excluding gender as all participants were male). We also recruited for the GBMSM sample through a push-notification inbox advert on Grindr (1.1 million adverts delivered, 24,933 opened, and 4288 clicks) and using targeted adverts on Meta (308,472 adverts shown, 108,865 adverts seen, and 3675 clicks). No quotas were placed on these samples.

For peer review only - http://bmjopen.bmj.com/site/about/guidelines.xhtml

Study materials

Full study materials are in supplementary materials 1. Items were based on previously validated measures,[33, 34] and items used in previous surveys during the COVID-19 pandemic by our group.[11, 14, 35, 36]

Outcome measures

Self-isolation intention was measured using two items, asking participants to imagine that they were contacted by public health officials and told that they needed to self-isolate for 21 days because they had mpox, and because they had come into high-risk contact with a case. Responses were given on a five-point scale from "definitely would not" to "definitely would". The order of items was randomised between participants to mitigate potential order effects. As answers on both items were highly correlated across all respondents ($r = 0.77$, $p = 0.001$), we combined responses to give a nine-point scale (2 to 10; summing responses "definitely would not" = 1 to "definitely would" = 5; higher number indicates greater intention).

To measure help-seeking intention, participants were asked to imagine that they developed an unexplained rash with blisters and learned that they had come into contact with an mpox case. They were then asked what they would do, from a list of ten behaviours including waiting to see if they got better, contacting healthcare services, letting people you had been in recent close contact know, and searching for information. Responses for each item were given on the same five-point "definitely would not" to "definitely would" scale. We created a binary variable, coding participants as intending to seek help immediately if they answered "probably" or "definitely would" to any help seeking where they would encounter a health professional (trying to book an appointment with a general practitioner, visiting a pharmacist/chemist, going to Accident & Emergency or another NHS service, calling NHS111 or 999, visiting a walk-in sexual health clinic, or calling a sexual health clinic), and did not select "wait a day or two to see if they get better or clear up on their own" vs did not select any help-seeking behaviour where they would encounter a health professional, or selected a help-seeking behaviour but also stated that they would "wait a day or two to see…".

Participants were then asked about their intended contact behaviour in the same scenario, being asked "in the following 21 days, realistically how much" they would come into skin-to skin contact with others, have sexual contact, have sex without using a condom, go to a

For peer review only - http://bmjopen.bmj.com/site/about/guidelines.xhtml

crowded place, help or provide care for a vulnerable person, and go to a public place where they may come into physical contact with someone else. For each item, participants responded “I would completely stop doing this”, “less than normal”, “same as normal”, “more than normal”, “not applicable, I wouldn’t do this anyway”, “don’t know” or “prefer not to say”. We focused our analyses on the item asking about sexual contact (from kissing to intercourse) with other people, recoding it into a binary item (“I would completely stop doing this” vs would do this less than, same as, or more than usual). Answers of “not applicable, I wouldn’t do this anyway”, “don’t know”, and “prefer not to say” were coded as missing.

We measured intention to share details of close contacts by asking participants to indicate how likely they were, if asked by public health officials, to share contact details of every person who had been in their home, they had had sexual contact with, they had skin-to-skin contact with, and that they had shared bedding, towels or clothes with in the last seven days, and every place they had had sexual contact with someone. Responses were given on a five-point “definitely would not” (1) to “definitely would” (5) scale. We used the most recent official guidance on contact tracing available at the start of data analysis to select the item most relevant to contact tracing efforts (identifying every person you had sexual contact with in the last seven days).[7]

Participants were asked if they had received a smallpox vaccine in 2022. Those who indicated they had not had a vaccine were asked about their vaccination intention. We asked participants how likely they would be to have a smallpox vaccine if they were offered one. Responses were given on a five-point “definitely would not” (1) to “definitely would” (5) scale.

Motivational messaging

Very brief motivational messages were constructed based on components of the PMT.[13] The general population sample were randomised to one of four groups, and were shown messages about: 1) perceived risk of illness and necessity and efficacy of the response, 2) perceived risk of illness plus benefits of the response, 3) perceived risk of illness plus low perceived costs of response, and 4) a control message of similar length giving factual details about the mpox outbreak. Messages are shown in supplementary materials 1. Messages to promote perceived risk of illness (i.e. susceptibility to and severity of mpox) were included in all motivational messages, as we hypothesised that perceiving a risk is necessary before choosing to adopt a behaviour to mitigate that risk. Due to anticipated smaller sample sizes, the GBMSM samples were randomised to one of two messages. The first included all

For peer review only - http://bmjopen.bmj.com/site/about/guidelines.xhtml

motivational components, whereas the second, a control message, gave factual information about the mpox outbreak.

Psychological factors

We asked participants how much they had seen or heard about mpox, how worried they were about mpox, and how much risk they thought mpox posed to people in the UK and themselves personally. For these items, answers of "don't know" were coded as missing.

Participants were also asked about their perceived susceptibility to mpox (two items: would be likely to come into contact with a case; would be likely to catch mpox if in contact with a case) and perceived severity of mpox. We asked participants how much they agreed that: their personal behaviour had an impact on the spread of mpox; their life had been negatively affected by changes made in response to the mpox outbreak; the risks of mpox were being exaggerated; people who catch mpox usually make a full recovery without treatment; and that mpox is only a risk to men who are gay, bisexual, or have sex with men. For perceptions such as these, with no right or wrong answers, we recoded answers of "don't know" as the mid-point on the scale.

To measure perceived knowledge, we asked participants three items about whether they had a good idea how people catch mpox, they knew the main symptoms of mpox, and they thought it would be easy to tell if someone had mpox. Knowledge about mpox symptoms was measured using a question asking participants to identify the main symptoms of mpox from a list of fifteen taken from the NHS mpox website [1] and common, non-specific symptoms. Participants could select up to four symptoms. Understanding of transmission was measured using seven items, asking about contact and droplet transmission (adapted from [33], and other modes of transmission as specified by the World Health Organization (WHO) website [37]). For factual questions such as these, we coded "don't know" as missing.

Behaviour-specific perceptions were also investigated. We used a series of ten items to measure factors that may be associated with self-isolation, including perceived effectiveness, social norms, having the necessary support, and impact on social connectedness, family wellbeing, and finances. Factors that may affect intention to seek help were measured by six items asking participants to what extent they agreed that they would not want to know the results of a mpox test, they would be worried what their friends, family, or employers thought of them if they had mpox, not wanting to have a mpox test on their medical record, testing is an effective way to prevent the spread of mpox, and being willing to contact a sexual health

For peer review only

For peer review only - http://bmjopen.bmj.com/site/about/guidelines.xhtml

clinic if they thought they had mpox symptoms or had come into contact with a case. Vaccination attitudes were measured by eight items, asking about general vaccine attitudes, perceived social norms, perceived effectiveness of vaccination, worry about vaccine side-effects, that the vaccine could make you infectious to others, and thinking that those who come into high-risk contact with mpox should be vaccinated. Responses of "don't know" were recoded to the mid-point of the scale.

Socio-demographic characteristics

Participants were asked to report their age, gender,[38] sexual orientation,[39] socio-economic grade,[40] financial hardship (adapted from [41]), employment status, highest level of education, ethnicity, marital status, how many people lived in their household, whether they were the parent or guardian of any dependent children, and if they had any pets. Questions asking about gender and the categorisations used were based on those used by the Genitourinary Medicine Clinic Activity Dataset (GUMCAD) sexually transmitted infections (STI) surveillance system in England.[38] Those who were employed were asked if they were a frontline health or social care worker and if they needed to leave home for work. For these items, we coded participants who were not employed as not being a frontline health or social care worker (prefer not to say coded as missing) and not needing to leave home for work, respectively. Participants were asked for their full postcode, from which region and indices of multiple deprivation were determined.[42]

We asked participants if they or a household member had a chronic illness, whether they were pregnant, had ever taken pre-exposure prophylaxis (PrEP) for HIV, and for their vaccination status for smallpox (in 2022, and before 2022), hepatitis A, and COVID-19 (two doses or more).

Participants were also asked how many male and female sexual partners they had had in the last 3 weeks and last 3 months.

Ethics

Ethical approval for this study was given by the King's College London Psychiatry, Nursing and Midwifery Research Ethics Panel (reference number: LRS/DP-21/22-32287). Participants gave informed written consent before beginning survey materials.

For peer review only - http://bmjopen.bmj.com/site/about/guidelines.xhtml

Patient and public involvement

To ensure the research aims and study information were appropriate, members of the public were involved in the development of the funding application and survey materials. For the funding application, six people gave feedback on the initial proposal resulting in changes to aims of the study and terminology used. For the survey materials, four lay people (two GBMSM) gave feedback on the questionnaire and motivational messages, resulting in changes to survey items and messages to improve clarity, validity, and readability of statements.

Power

A sample size of 3000 allows a 95% confidence interval of plus or minus 1.8% for the prevalence estimate for a survey item with a prevalence of around 50% (sample size of 250 gives a confidence interval of plus or minus 6.2%; sample size of 1000 gives a confidence interval of plus or minus 3.1%).

For multiple linear regression analyses, a sample of 830 allowed over 99% power to detect small effect sizes (f=0.10) at p=0.001 (43 predictors). For logistic regression analyses, a sample of 830 allowed over 99% power to detect small effect sizes (OR=1.68 [43]) at p=0.001 (42.5% self-isolation, 18.0% requesting a test, 79.1% sharing details of close contacts [11]).

Analysis

Information about data preparation is reported in supplementary materials 2.

We tested whether socio-demographic characteristics of GBMSM samples were different depending on the recruitment method (Savanta, Grindr, Meta). Due to significant differences, further analyses were conducted in each sample separately.

First, we tested the influence of motivational messages on outcomes using χ^2-tests (binary outcomes), one-way ANOVAs (general population sample, continuous outcomes), or t-tests (GBMSM, continuous outcomes). For vaccination, we investigated the influence of motivational message on intention to be vaccinated if advised (excluding people already vaccinated).

Next, we investigated psychological and contextual factors associated with intended behaviours (self-isolation, help seeking, sexual contact, sharing details of contacts, vaccination). To minimise analyses conducted, we investigated one variable per outcome,

For peer review only - http://bmjopen.bmj.com/site/about/guidelines.xhtml

except for vaccination (GBMSM sample investigated two outcomes). For vaccination, as smallpox vaccine uptake in GBMSM was high, we used two binary outcomes: vaccination uptake in 2022 (vaccinated vs not vaccinated), and a computed variable indicating vaccine intention and uptake (vaccinated or intend to be vaccinated [“definitely” or “probably would”] vs not vaccinated and do not intend to be vaccinated [“not sure”, “probably would not”, “definitely would not”]). For the general population sample, we used only the computed variable. We conducted regressions (binary logistic for binary outcomes, linear for continuous outcomes) in the general population sample and Grindr sample (the target GBMSM sample who were actively seeking new partners; Savanta sample excluded due to small numbers; Meta sample excluded as they differed significantly from the general population).

We entered variables into regressions in blocks. In the first block, we entered socio-demographic characteristics: gender (general population sample only, male / female), sexual orientation (general population sample only, straight or heterosexual / gay, lesbian, bisexual, or queer), age, region, having a dependent child in household (no / yes), employment status (working / not working), education (GCSE, vocational, A-level, no formal qualifications / degree or higher), ethnicity (White British / White other / Black, Asian, other minoritized ethnicity), marital status, living alone, having a chronic illness oneself (none / present), index of multiple deprivation (deciles), socio-economic grade (ABC1 / C2DE), financial hardship, and motivational message. In the Grindr sample, we also included smallpox vaccination status in 2022 (except for vaccine uptake outcome) and ever having taken PrEP for HIV.

In the second block, we entered psychological and contextual factors: self-reported knowledge, knowledge of mpox symptoms, knowledge of mpox transmission, amount heard about mpox, worry about mpox, perceived risk of mpox (to oneself and to people in the UK), perceived susceptibility and severity of mpox, thinking that you are immune to mpox, that your personal behaviour has an impact on how mpox spreads, that your life has been negatively affected by changes made in response to the mpox outbreak, that the risks of mpox are being exaggerated, that mpox is only a risk to men who are gay, bisexual or have sex with men, and that people who catch mpox usually make a full recovery even without treatment.

For self-isolation, help seeking and vaccination, a third block was also added, which included specific factors potentially associated with individual outcomes. Items were chosen through principal component analyses (see supplementary materials 3).

For peer review only - http://bmjopen.bmj.com/site/about/guidelines.xhtml

All analyses were carried out in SPSS 28. Data are unweighted.

Many comparisons were investigated in regression models (n=40 to n=43, based on outcome). Therefore, we applied a conservative Bonferroni correction and only report as significant results with $p \leq 0.001$ to reduce the risk of Type I errors.

RESULTS

Top-line results for all survey materials, by sample, are shown in supplementary materials 1. Anonymised data are available online.[44]

For regression analyses, we report imputed values. Results using imputed values were compared with non-imputed data. There were no substantial differences in results with and without imputed values.

Participant characteristics

There were significant differences in participant characteristics by sampling method. Most notably, participants recruited from Grindr and Meta were more likely to be working, need to leave home for work, more highly educated, higher socio-economic grade, and have less financial hardship (Table 1). In the general population sample, participants were mostly female (57%), white British (87%), with a mean age of 49 years.

For peer review only

Table 1. Participant characteristics, by recruitment method.

		General population, n=3050	GBMSM samples			
			Savanta GBMSM, n=247	Grindr, n=831	Meta, n=1036	p-value
Gender	Male (including trans man)	1278 (41.9)	238 (96.4)	828 (99.6)	1013 (97.8)	-
	Gender same as assigned at birth (no or prefer not to say)	11 (0.4)	9 (3.6)	3 (0.04)	23 (2.2)	-
	Female (including trans woman)	1729 (56.7)	-	-	-	-
	Gender same as assigned at birth (no or prefer not to say)	7 (0.02)	-	-	-	-
	Non-binary	8 (0.3)	-	-	-	-
	Gender same as assigned at birth (no or prefer not to say)	9 (0.3)	-	-	-	-
Sexual orientation	Straight or heterosexual	2795 (92.7)	-	-	-	-
	Gay, lesbian, bisexual, or queer	221 (7.3)	247 (100.0)	831 (100.0)	1036 (100.0)	-
Age	Range 18 to 98 years	M=48.6, SD=17.4	M=47.1, SD=16.5	M=44.2, SD=12.5	M=47.6, SD=11.9	<0.001*
Region†	Midlands (East and West)	522 (17.1)	39 (15.8)	73 (8.8)	85 (8.2)	<0.001*
	South (East, West, East of England)	992 (32.5)	70 (28.3)	207 (24.9)	279 (26.9)	
	North (East, West, Yorkshire and the Humber)	774 (25.4)	58 (23.5)	119 (14.3)	155 (15.0)	
	London	308 (10.1)	46 (18.6)	195 (23.5)	379 (36.6)	
	Devolved nations (Scotland, Wales, and Northern Ireland)	454 (14.9)	34 (13.8)	95 (11.4)	84 (8.1)	
	Not specified	0 (0.0)	0 (0.0)	142 (17.1)	54 (5.2)	
Dependent child in household	No	2075 (68.0)	210 (85.0)	796 (95.8)	1014 (97.9)	<0.001*
	Yes	975 (32.0)	37 (15.0)	35 (4.2)	22 (2.1)	
Employment status	Not working	1286 (42.6)	93 (38.3)	157 (19.0)	201 (19.5)	<0.001*
	Working	1736 (57.4)	150 (61.7)	669 (81.0)	831 (80.5)	
Frontline health or social care worker	No	2668 (88.3)	224 (90.7)	734 (88.6)	909 (87.9)	0.66
	Yes	355 (11.7)	23 (9.3)	94 (11.4)	125 (12.1)	
Need to leave home for work	Do not need to leave home for work	1946 (63.8)	149 (60.3)	371 (44.6)	499 (48.2)	<0.001*
	Need to leave home for work	1104 (36.2)	98 (39.7)	460 (55.4)	537 (51.8)	
Education	GCSE/vocational/A-level/No formal qualifications	2065 (67.7)	159 (64.4)	295 (35.5)	253 (24.4)	<0.001*
	Degree or higher (Bachelors, Masters, PhD)	985 (32.3)	88 (35.6)	536 (64.5)	783 (75.6)	
Ethnicity	White British	2649 (87.1)	214 (86.6)	629 (76.2)	799 (78.0)	<0.001*
	White other	116 (3.8)	18 (7.3)	117 (14.2)	163 (15.9)	

For peer review only - http://bmjopen.bmj.com/site/about/guidelines.xhtml

For peer review only

	Black, Asian, other minoritized ethnicity	275 (9.0)	15 (6.1)	80 (9.7)	63 (6.1)	
Marital status	Not partnered<br>Partnered	1265 (41.6)<br>1775 (58.4)	141 (57.3)<br>105 (42.7)	561 (68.3)<br>260 (31.7)	486 (47.3)<br>541 (52.7)	<0.001*
Live alone	Live with someone else<br>Live alone	2387 (78.3)<br>663 (21.7)	146 (59.1)<br>101 (40.9)	473 (56.9)<br>358 (43.1)	656 (63.3)<br>380 (36.7)	0.02
Own chronic illness	None<br>Present	2207 (72.4)<br>843 (27.6)	167 (67.6)<br>80 (32.4)	619 (74.5)<br>212 (25.5)	731 (70.6)<br>305 (29.4)	0.05
Household member chronic illness	None<br>Present	2659 (87.2)<br>391 (12.8)	226 (91.5)<br>21 (8.5)	743 (89.4)<br>88 (10.6)	921 (88.9)<br>115 (11.1)	0.49
Ever taken PrEP for HIV	No<br>Yes	2993 (98.6)<br>42 (1.4)	216 (88.5)<br>28 (11.5)	427 (51.5)<br>402 (48.5)	547 (52.9)<br>487 (47.1)	<0.001*
Vaccinated for smallpox in 2022	Not vaccinated<br>Vaccinated	2888 (94.7)<br>162 (5.3)	221 (89.5)<br>26 (10.5)	566 (68.1)<br>265 (31.9)	614 (59.3)<br>422 (40.7)	<0.001*
Index of multiple deprivation†	Deciles (1st = most deprived, 10th = least deprived)	M=4.5, SD=2.9	M=4.4, SD=3.0	N=689, M=4.7, SD=2.8	N=982, M=4.9, SD=2.7	<0.001*
Socio-economic grade	ABC1<br>C2DE	1796 (58.9)<br>1254 (41.1)	169 (68.4)<br>78 (31.6)	694 (83.5)<br>137 (16.5)	944 (91.1)<br>92 (8.9)	<0.001*
Financial hardship†	4 (lowest hardship) to 13 (most hardship)	N=2743, M=6.1, SD=2.3	N=223, M=5.9, SD=2.4	N=784, M=5.2, SD=1.8	N=1004, M=4.7, SD=1.3	<0.001*

*$p \leq 0.001$
†Using original (not imputed) data.

For peer review only

For peer review only - http://bmjopen.bmj.com/site/about/guidelines.xhtml

Motivational messaging

There was no effect of motivational messaging on outcomes, except for in the sample recruited from Meta (see supplementary materials 4). In this sample, those receiving the motivational message were less likely to intend to self-isolate for 21 days (t(1034)= -2.81, p=0.005; motivational message, n=529, M=7.1, SD=2.3; control message, n=507, M=7.5, SD=2.2), share details of all recent sexual contacts (t(1029)= -2.05, p=0.04; motivational message, n=526, M=4.1, SD=1.2; control message, n=505, M=4.2, SD=1.1), and be vaccinated for smallpox if advised (t(612)= -2.21, p=0.03; motivational message, n=304, M=4.7, SD=0.9; control message, n=310, M=4.8, SD=0.6).

Self-isolation

Rates of intended self-isolation were higher when imagining you were a case than a high-risk contact. Three-quarters of the general population sample intended to self-isolate for 21 days if they were to develop mpox (75.2%, 95% CI 73.7% to 76.7%, n=2294; Table 2). However, only 68.9% (95% CI 67.2% to 70.5%, n=2100) intended to self-isolate if they were to come into contact with a case. Intention to self-isolate was lower in Grindr and Meta samples.

For peer review only

Table 2. Main behavioural outcomes, by sample.

			N (%, 95% CI) General population, total n=3050	Savanta GBMSM, total n=247	Grindr, total n=831	Meta, total n=1036	p-value
**Self-isolation for 21 days**	If "you have" mpox	Probably or definitely would not, or not sure	756 (24.8, 23.3-26.3)	53 (21.5, 16.3-26.6)	246 (29.6, 26.5-32.7)	275 (26.5, 23.9-29.2)	-
		Probably or definitely would	2294 (75.2, 73.7-76.7)	194 (78.5, 73.4-83.7)	585 (70.4, 67.3-73.5)	761 (73.5, 70.8-76.1)	
	If "you have come into high-risk contact with someone who has" mpox	Probably or definitely would not, or not sure	950 (31.1, 29.5-32.8)	77 (31.2, 25.4-37.0)	371 (44.6, 41.3-48.0)	491 (47.4, 44.3-50.4)	-
		Probably or definitely would	2100 (68.9, 67.2-70.5)	170 (68.8, 63.0-74.6)	460 (55.4, 52-58.7)	545 (52.6, 49.6-55.7)	
	Sum of intention if case and high-risk contact	2 (lowest intention) to 10 (highest intention)	M=8.0, SD=2.2	M=7.9, SD=2.3	M=7.2, SD=2.4	M=7.3, SD=2.3	<0.001*
**Help seeking**	Any action that involves contacting a healthcare professional (by phone or in person)	Would not seek help or would "wait and see" for a day or two to see if symptoms resolved	1548 (50.8, 49.0-52.5)	125 (50.6, 44.3-56.9)	388 (46.7, 43.3-50.1)	391 (37.7, 34.8-40.7)	<0.001*
		Would seek help immediately	1502 (49.2, 47.5-51.0)	122 (49.4, 43.1-55.7)	443 (53.3, 49.9-56.7)	645 (62.3, 59.3-65.2)	
**Sexual contact behaviour †**	Have sexual contact with others	Would not completely stop	567 (22.8, 21.1-24.4)	45 (20.8, 15.4-26.3)	69 (8.7, 6.8-10.7)	69 (7.0, 5.4-8.6)	<0.001*
		Completely stop	1923 (77.2, 75.6-78.9)	171 (79.2, 73.7-84.6)	721 (91.3, 89.3-93.2)	922 (93.0, 91.4-94.6)	
**Contact sharing**	Sexual contacts	Probably or definitely would not, or not sure	697 (23.3, 21.7-24.8)	60 (24.7, 19.2-30.2)	167 (20.3, 17.5-23.0)	204 (19.8, 17.4-22.2)	-
		Probably or definitely would	2300 (76.7, 75.2-78.3)	183 (75.3, 69.8-80.8)	656 (79.7, 77-82.5)	827 (80.2, 77.8-82.6)	
	Scale	1 (definitely would not) to 5 (definitely would)	N=2997, M=4.2, SD=1.2	N=243, M=4.1, SD=1.2	N=823, M=4.1, SD=1.2	N=1031, M=4.2, SD=1.1	0.57
**Vaccination intention**	Vaccinated in 2022	No or not sure	2888 (94.7, 93.9-95.5)	221 (89.5, 85.6-93.3)	566 (68.1, 64.9-71.3)	614 (59.3, 56.3-62.3)	<0.001*
		Yes	162 (5.3, 4.5-6.1)	26 (10.5, 6.7-14.4)	265 (31.9, 28.7-35.1)	422 (40.7, 37.7-43.7)	
	Vaccinated in 2022 or would be vaccinated if advised	Not vaccinated and probably or definitely would not be vaccinated or not sure	786 (25.8, 24.2-27.3)	37 (15.0, 10.5-19.5)	54 (6.5, 4.8-8.2)	38 (3.7, 2.5-4.8)	<0.001*
		Vaccinated or probably or definitely would be vaccinated	2264 (74.2, 72.7-75.8)	210 (85.0, 80.5-89.5)	777 (93.5, 91.8-95.2)	998 (96.3, 95.2-97.5)	

*$p \leq 0.001$
†Answers of "don't know", "prefer not to say" and "not applicable, I wouldn't do this anyway" were coded as missing, therefore total *n*s are substantially lower (general population, n=2490; Savanta GBMSM, n=216; Grindr, n=790; Meta, n=991).

For peer review only - http://bmjopen.bmj.com/site/about/guidelines.xhtml

In the general population, intention to self-isolate was associated with: less financial hardship, being more worried about mpox, perceiving a bigger threat of mpox to people in the UK, greater perceived susceptibility and severity of mpox, and greater perceived social norms for self-isolation (Tables 3 and 4). Not intending to self-isolate was associated with agreeing that if you had mpox symptoms, you would not want to tell anyone as you did not want to self-isolate, believing the risks of mpox were being exaggerated, and that if you had to self-isolate due to mpox it would have a negative impact on your work (Table 4). In the Grindr sample, self-isolation intention was associated with greater perceived social norms (Table 4). Not intending to self-isolate was associated with agreeing that if you had mpox symptoms, you would not want to tell anyone as you did not want to self-isolate, and that if you had to self-isolate due to mpox it would have a negative impact on your work (Table 4).

For peer review only

Table 3. Associations between intending to self-isolate and socio-demographic characteristics and motivational message, by sample. A higher score indicates greater intention to self-isolate. Variables were entered into the linear regression model in blocks (block 1: socio-demographic variables and motivational message, block 2: psychological factors, block 3: isolation-specific beliefs). Results for block 3, using pooled estimates are reported.

Participant characteristics	Level	General population *B* (95% CI)	General population *p*-value	Grindr *B* (95% CI)	Grindr *p*-value
Gender	Male (including trans man)	Ref	-	-	-
	Female (including trans woman)	0.15 (0.01 to 0.30)	0.03	-	-
Sexual orientation	Straight or heterosexual	Ref	-	-	-
	Gay, lesbian, bisexual, or queer	0.06 (-0.21 to 0.33)	0.67	-	-
Age	Range 18 to 98 years	0.01 (0.00 to 0.01)	0.06	0.00 (-0.01 to 0.02)	0.66
	Quadratic term, (age – mean)^2	0.0002 (-0.0001 to 0.0005)	0.17	0.001 (0.000 to 0.002)	0.02
Region	Midlands (East and West)	Ref	-	Ref	-
	South (East, West, East of England)	-0.19 (-0.39 to 0.01)	0.07	0.02 (-0.51 to 0.55)	0.95
	North (East, West, Yorkshire and the Humber)	-0.07 (-0.28 to 0.13)	0.49	0.06 (-0.53 to 0.64)	0.85
	London	-0.11 (-0.39 to 0.16)	0.42	-0.01 (-0.59 to 0.57)	0.98
	Devolved nations (Scotland, Wales, and Northern Ireland)	-0.04 (-0.30 to 0.22)	0.78	0.48 (-0.20 to 1.16)	0.17
Dependent child in household	No	Ref	-	Ref	-
	Yes	-0.15 (-0.32 to 0.03)	0.10	-0.42 (-1.13 to 0.29)	0.25
Employment status	Not working	Ref	-	Ref	-
	Working	0.09 (-0.12 to 0.31)	0.39	-0.13 (-0.61 to 0.34)	0.58
Frontline health or social care worker	No	Ref	-	Ref	-
	Yes	0.08 (-0.14 to 0.30)	0.47	0.68 (0.24 to 1.13)	0.003
Need to leave home for work	Do not need to leave home for work	Ref	-	Ref	-
	Need to leave home for work	-0.26 (-0.45 to -0.07)	0.007	0.14 (-0.22 to 0.51)	0.44
Education	GCSE/vocational/A-level/No formal qualifications	Ref	-	Ref	-
	Degree or higher (Bachelors, Masters, PhD)	0.02 (-0.13 to 0.18)	0.77	-0.16 (-0.48 to 0.16)	0.31
Ethnicity	White British	Ref	-	Ref	-
	White other	-0.23 (-0.59 to 0.13)	0.21	-0.33 (-0.75 to 0.09)	0.12
	Black, Asian, other minoritized ethnicity	0.11 (-0.15 to 0.37)	0.40	-0.03 (-0.52 to 0.46)	0.91
Marital status	Not partnered	Ref	-	Ref	-
	Partnered	0.15 (-0.04 to 0.34)	0.12	0.13 (-0.24 to 0.49)	0.50
Live alone	Live with someone else	Ref	-	Ref	-
	Live alone	0.06 (-0.15 to 0.28)	0.56	-0.07 (-0.40 to 0.26)	0.69
Own chronic illness	None	Ref	-	Ref	-
	Present	0.21 (0.05 to 0.38)	0.009	0.10 (-0.24 to 0.44)	0.56
Ever taken PrEP for HIV	No	-	-	Ref	-
	Yes	-	-	-0.11 (-0.43 to 0.22)	0.51
Vaccinated for smallpox in 2022	Not vaccinated	-	-	Ref	-
	Vaccinated	-	-	0.03 (-0.32 to 0.39)	0.86
Index of multiple deprivation	Deciles (1st = most deprived, 10th = least deprived)	-0.01 (-0.04 to 0.02)	0.48	0.02 (-0.05 to 0.08)	0.66
Socio-economic grade	ABC1	Ref	-	Ref	-
	C2DE	-0.09 (-0.24 to 0.06)	0.24	-0.03 (-0.46 to 0.39)	0.88

For peer review only - http://bmjopen.bmj.com/site/about/guidelines.xhtml

Financial hardship	4 (lowest hardship) to 13 (most hardship)	-0.08 (-0.12 to -0.05)	<0.001*	-0.02 (-0.11 to 0.07)	0.70
Total number of sexual partners (male and female) in last three weeks	0	Ref	-	-	-
	1	-0.04 (-0.23 to 0.14)	0.65	-	-
	2 to 4	-0.25 (-0.59 to 0.10)	0.16	-	-
	5 or more	-0.09 (-0.69 to 0.52)	0.77	-	-
	Prefer not to say	-0.22 (-0.42 to -0.02)	0.03	-	-
Number of male sexual partners in last three weeks	0	-	-	Ref	-
	1	-	-	-0.50 (-0.92 to -0.08)	0.02
	2 to 4	-	-	-0.09 (-0.47 to 0.30)	0.65
	5 to 9	-	-	-0.48 (-1.00 to 0.03)	0.07
	10 or more	-	-	-0.32 (-0.96 to 0.33)	0.34
	Prefer not to say	-	-	0.83 (0.12 to 1.53)	0.02
Motivational message	Perceived risk of illness and necessity and efficacy of the response	-0.06 (-0.25 to 0.12)	0.50	-	-
	Perceived risk of illness and benefits of the response	-0.09 (-0.28 to 0.09)	0.33	-	-
	Perceived risk of illness and low perceived costs of response	-0.19 (-0.38 to 0.00)	0.05	-	-
	Control	Ref	-	-	-
Motivational message	All motivational components	-	-	-0.10 (-0.37 to 0.18)	0.48
	Control	-	-	Ref	-

*$p \leq 0.001$

For peer review only

Table 4. Associations between intending to self-isolate and psychological factors and isolation-specific beliefs, by sample. A higher score indicates greater intention to self-isolate. Variables were entered into the linear regression model in blocks (block 1: socio-demographic variables and motivational message, block 2: psychological factors, block 3: isolation-specific beliefs). Results for block 3, using pooled estimates are reported.

Factor	Level	General population		Grindr	
		B (95% CI)	*p*-value	*B* (95% CI)	*p*-value
Amount heard about mpox	I have not seen or heard anything (1) to I have seen or heard a lot (3)	0.09 (-0.07 to 0.24)	0.26	-0.09 (-0.41 to 0.23)	0.57
Worry about mpox	Not at all worried (1) to extremely worried (4)	0.26 (0.13 to 0.39)	<0.001*	0.41 (0.12 to 0.71)	0.006
Perceived risk of mpox to oneself	No risk at all (1) to very high risk (5)	-0.05 (-0.17 to 0.06)	0.39	-0.25 (-0.46 to -0.04)	0.02
Perceived risk of mpox to people in UK	No risk at all (1) to very high risk (5)	0.19 (0.07 to 0.31)	0.001*	0.030 (-0.20 to 0.25)	0.82
Perceived susceptibility and severity	Lowest (1) to highest (5)	0.03 (0.19 to 0.42)	<0.001*	0.12 (-0.13 to 0.37)	0.36
I am already immune to mpox	Strongly disagree, disagree, neither agree nor disagree, don't know	Ref	-	Ref	-
	Strongly agree and agree	0.27 (0.03 to 0.52)	0.03	-0.12 (-0.61 to 0.36)	0.62
People who catch mpox usually make a full recovery, even if they do not receive any treatment	Strongly disagree (1) to strongly agree (5)	-0.05 (-0.14 to 0.03)	0.23	-0.26 (-0.43 to -0.08)	0.004
My personal behaviour has an impact on how mpox spreads	Strongly disagree (1) to strongly agree (5)	0.09 (0.03 to 0.15)	0.004	0.08 (-0.06 to 0.22)	0.26
My life has been negatively affected by changes made in response to the mpox outbreak	Strongly disagree (1) to strongly agree (5)	-0.01 (-0.09 to 0.07)	0.89	-0.03 (-0.17 to 0.11)	0.70
The risks of mpox are being exaggerated	Strongly disagree (1) to strongly agree (5)	-0.19 (-0.27 to -0.11)	<0.001*	-0.07 (-0.24 to 0.09)	0.39
Mpox is only a risk to men who are gay, bisexual or have sex with men	Strongly disagree (1) to strongly agree (5)	-0.02 (-0.08 to 0.05)	0.58	-0.17 (-0.32 to -0.02)	0.03
Perceived knowledge	Lowest (0) to highest (3)	-0.01 (-0.08 to 0.07)	0.88	-0.18 (-0.37 to 0.02)	0.08
Knowledge of mpox symptoms	Identified no symptoms (0) to identified four symptoms (4)	0.08 (0.03 to 0.14)	0.003	0.06 (-0.06 to 0.19)	0.31
Knowledge of mpox transmission	Lowest (0) to highest (6)	0.02 (-0.03 to 0.07)	0.42	0.04 (-0.10 to 0.17)	0.59
If I had mpox symptoms, I wouldn't want to tell anyone as I don't want to self-isolate	Strongly disagree (1) to strongly agree (5)	-0.66 (-0.73 to -0.60)	<0.001*	-1.01 (-1.15 to -0.87)	<0.001*
Most people would self-isolate if they were told to	Strongly disagree (1) to strongly agree (5)	0.34 (0.28 to 0.41)	<0.001*	0.41 (0.28 to 0.54)	<0.001*
If I had to self-isolate because I had tested positive for mpox...it would have a negative impact on my work	Strongly disagree (1) to strongly agree (5)	-0.15 (-0.21 to -0.09)	<0.001*	-0.34 (-0.46 to -0.21)	<0.001*

*$p \leq 0.001$

For peer review only

For peer review only - http://bmjopen.bmj.com/site/about/guidelines.xhtml

Help seeking

Approximately half of participants in the general population, Savanta GBMSM, and Grindr samples indicated that they would seek help immediately (49.2% to 53.3%; Table 2). Intention to seek help immediately was higher in the Meta sample (62.3%, 95% CI 59.3 to 65.2, n=645).

In the general population, intention to seek help immediately was associated with being older (aOR 1.012, 95% CI 1.006 to 1.019, $p<0.001$), disagreeing that the risks of mpox are being exaggerated (aOR 0.83, 95% CI 0.76 to 0.91, $p<0.001$), and being willing to contact a sexual health clinic if you thought you had mpox symptoms or had been in contact with a case (aOR 1.25, 95% CI 1.16 to 1.34, $p<0.001$; supplementary materials 5). In the Grindr sample, intention to seek help immediately was associated with being willing to contact a sexual health clinic if you thought you had mpox symptoms or had been in contact with a case (aOR 1.57, 95% CI 1.29 to 1.91, $p<0.001$; supplementary materials 5).

Sexual contact behaviour when symptomatic

In the general population and Savanta GBMSM, 77.2% (95% CI 75.6% to 78.9%, n=1923) and 79.2% (73.7% to 84.6%, n=171) intended to completely stop sexual contact if they were to develop an unexplained rash with blisters and learn that they had come into contact with a mpox case (Table 2). Rates of intending to completely stop sexual contact were significantly higher in Grindr (91.3%, 95% CI 89.3% to 93.2%, n=721) and Meta samples (93.0%, 95% CI 91.4% to 94.6%, n=922). The number of days that participants would wait before resuming sexual contact from the start of their symptoms was also higher in Grindr and Meta samples than in general population and Savanta GBMSM samples ($F(3,3900)=29.0$, $p<0.001$; general population: n=2021, M=15.7, SD=15.3; Savanta GBMSM: n=190, M=16.2, SD=14.4; Grindr: n=746, M=20.4, SD=15.8; Meta: n=947, M=20.0, SD=12.3).

In the general population, intending to completely stop any sexual contact if symptomatic was associated with being female, older, less financial hardship, and being more knowledgeable about mpox transmission (Tables 5 and 6). Not intending to completely stop sexual behaviour was associated with preferring not to say how many recent sexual partners you had had, thinking that you were already immune to mpox, that your life had been negatively affected by changes made in response to the mpox outbreak, and that the risks of mpox were being exaggerated (Tables 5 and 6). No associations reached significance (Bonferroni corrected) in the Grindr sample.

For peer review only - http://bmjopen.bmj.com/site/about/guidelines.xhtml

Table 5. Associations between intending to completely stop any sexual contact and socio-demographic characteristics and motivational message, by sample. Variables were entered into the logistic regression model in blocks (block 1: socio-demographic variables and motivational message, block 2: psychological factors). Results for block 2, using pooled estimates are reported.

Participant characteristics	Level	General population		Grindr	
		aOR (95% CI)	*p*-value	aOR (95% CI)	*p*-value
Gender	Male (including trans man)	Ref	-	-	-
	Female (including trans woman)	1.90 (1.48 to 2.45)	<0.001*	-	-
Sexual orientation	Straight or heterosexual	Ref	-	-	-
	Gay, lesbian, bisexual, or queer	0.91 (0.58 to 1.41)	0.67	-	-
Age	Range 18 to 98 years	1.04 (1.03 to 1.05)	<0.001*	1.01 (0.98 to 1.04)	0.53
	Quadratic term, $(age - mean)^2$	1.000 (0.999 to 1.001)	0.87	1.000 (0.998 to 1.001)	0.71
Region	Midlands (East and West)	Ref	-	Ref	-
	South (East, West, East of England)	1.08 (0.76 to 1.54)	0.67	1.18 (0.38 to 3.66)	0.77
	North (East, West, Yorkshire and the Humber)	1.14 (0.79 to 1.66)	0.49	1.48 (0.35 to 6.21)	0.58
	London	1.17 (0.73 to 1.86)	0.52	0.94 (0.27 to 3.25)	0.92
	Devolved nations (Scotland, Wales, and Northern Ireland)	1.26 (0.79 to 2.01)	0.33	1.26 (0.31 to 5.15)	0.74
Dependent child in household	No	Ref	-	Ref	-
	Yes	0.97 (0.72 to 1.29)	0.82	0.37 (0.11 to 1.28)	0.12
Employment status	Not working	Ref	-	Ref	-
	Working	1.22 (0.90 to 1.66)	0.20	0.92 (0.39 to 2.18)	0.85
Frontline health or social care worker	No	Ref	-	Ref	-
	Yes	0.97 (0.67 to 1.39)	0.86	1.77 (0.67 to 4.65)	0.25
Education	GCSE/vocational/A-level/No formal qualifications	Ref	-	Ref	-
	Degree or higher (Bachelors, Masters, PhD)	0.96 (0.73 to 1.27)	0.79	1.87 (0.97 to 3.59)	0.06
Ethnicity	White British	Ref	-	Ref	-
	White other	0.47 (0.26 to 0.84)	0.01	0.84 (0.35 to 2.06)	0.71
	Black, Asian, other minoritized ethnicity	0.85 (0.56 to 1.27)	0.42	0.36 (0.15 to 0.85)	0.02
Marital status	Not partnered	Ref	-	Ref	-
	Partnered	0.90 (0.65 to 1.24)	0.50	1.11 (0.51 to 2.41)	0.79
Live alone	Live with someone else	Ref	-	Ref	-
	Live alone	1.16 (0.77 to 1.73)	0.48	1.12 (0.56 to 2.23)	0.75
Own chronic illness	None	Ref	-	Ref	-
	Present	0.97 (0.72 to 1.31)	0.85	1.10 (0.53 to 2.27)	0.80
Ever taken PrEP for HIV	No	-	-	Ref	-
	Yes	-	-	1.36 (0.69 to 2.71)	0.37
Vaccinated for smallpox in 2022	Not vaccinated	-	-	Ref	-
	Vaccinated	-	-	0.71 (0.33 to 1.50)	0.37
Index of multiple deprivation	Deciles (1st = most deprived, 10th = least deprived)	1.00 (0.96 to 1.06)	0.85	1.01 (0.86 to 1.19)	0.88
Socio-economic grade	ABC1	Ref	-	Ref	-
	C2DE	1.13 (0.86 to 1.49)	0.39	4.15 (1.45 to 11.85)	0.008
Financial hardship	4 (lowest hardship) to 13 (most hardship)	0.85 (0.80 to 0.90)	<0.001*	0.89 (0.74 to 1.07)	0.21
Total number of sexual partners (male and female) in last three weeks	0	Ref	-	-	-
	1	0.83 (0.59 to 1.18)	0.30	-	-
	2 to 4	0.68 (0.39 to 1.18)	0.17	-	-
	5 or more	0.37 (0.14 to 0.96)	0.04	-	-

For peer review only - http://bmjopen.bmj.com/site/about/guidelines.xhtml

	Prefer not to say	0.45 (0.32 to 0.64)	<0.001*	-	-
Number of male sexual partners in last three weeks	0	-	-	Ref	-
	1	-	-	0.52 (0.18 to 1.46)	0.21
	2 to 4	-	-	0.38 (0.14 to 0.98)	0.05
	5 to 9	-	-	0.21 (0.07 to 0.65)	0.007
	10 or more	-	-	0.20 (0.06 to 0.72)	0.01
	Prefer not to say	-	-	0.30 (0.08 to 1.11)	0.07
Motivational message	Perceived risk of illness and necessity and efficacy of the response	1.37 (0.97 to 1.93)	0.08	-	-
	Perceived risk of illness and benefits of the response	1.07 (0.76 to 1.49)	0.70	-	-
	Perceived risk of illness and low perceived costs of response	1.10 (0.79 to 1.55)	0.57	-	-
	Control	Ref	-	-	-
Motivational message	All motivational components	-	-	1.43 (0.80 to 2.54)	0.23
	Control	-	-	Ref	-

*$p \leq 0.001$

For peer review only

Table 6. Associations between intending to completely stop any sexual contact and psychological and contextual factors, by sample. Variables were entered into the logistic regression model in blocks (block 1: socio-demographic variables and motivational message, block 2: psychological factors). Results for block 2, using pooled estimates are reported.

Factor	Level	General population aOR (95% CI)	*p*-value	Grindr aOR (95% CI)	*p*-value
Amount heard about mpox	I have not seen or heard anything (1) to I have seen or heard a lot (3)	1.42 (1.08 to 1.86)	0.01	1.38 (0.70 to 2.72)	0.35
Worry about mpox	Not at all worried (1) to extremely worried (4)	1.11 (0.88 to 1.40)	0.36	1.51 (0.82 to 2.77)	0.19
Perceived risk of mpox to oneself	No risk at all (1) to very high risk (5)	0.83 (0.68 to 1.02)	0.08	0.93 (0.60 to 1.44)	0.75
Perceived risk of mpox to people in UK	No risk at all (1) to very high risk (5)	1.10 (0.90 to 1.34)	0.35	0.99 (0.64 to 1.53)	0.95
Perceived susceptibility and severity	Lowest (1) to highest (5)	1.27 (1.03 to 1.56)	0.03	1.18 (0.72 to 1.94)	0.51
I am already immune to mpox	Strongly disagree, disagree, neither agree nor disagree, don't know	Ref	-	Ref	-
	Strongly agree and agree	0.37 (0.26 to 0.53)	<0.001*	0.78 (0.32 to 1.90)	0.59
People who catch mpox usually make a full recovery, even if they do not receive any treatment	Strongly disagree (1) to strongly agree (5)	0.87 (0.74 to 1.01)	0.08	0.66 (0.45 to 0.97)	0.04
My personal behaviour has an impact on how mpox spreads	Strongly disagree (1) to strongly agree (5)	1.03 (0.92 to 1.16)	0.57	1.42 (1.08 to 1.87)	0.01
My life has been negatively affected by changes made in response to the mpox outbreak	Strongly disagree (1) to strongly agree (5)	0.70 (0.62 to 0.80)	<0.001*	0.73 (0.55 to 0.97)	0.03
The risks of mpox are being exaggerated	Strongly disagree (1) to strongly agree (5)	0.77 (0.67 to 0.88)	<0.001*	0.81 (0.58 to 1.11)	0.18
mpox is only a risk to men who are gay, bisexual or have sex with men	Strongly disagree (1) to strongly agree (5)	0.87 (0.77 to 0.97)	0.02	0.77 (0.58 to 1.03)	0.08
Perceived knowledge	Lowest (0) to highest (3)	1.00 (0.88 to 1.15)	0.94	0.86 (0.58 to 1.28)	0.46
Knowledge of mpox symptoms	Identified no symptoms (0) to identified four symptoms (4)	1.14 (1.03 to 1.26)	0.01	1.29 (1.00 to 1.66)	0.05
Knowledge of mpox transmission	Lowest (0) to highest (6)	1.18 (1.08 to 1.29)	<0.001*	1.31 (1.00 to 1.73)	0.05

*$p \leq 0.001$

For peer review only - http://bmjopen.bmj.com/site/about/guidelines.xhtml

Sharing details of contacts

There was no difference in intention to share details of all sexual partners in the last seven days between samples (Table 2), with 75.3% to 80.2% saying that they probably or definitely would.

In the general population sample, intention to share details of every sexual contact in the last seven days was associated with being female, older, less financial hardship, higher perceived susceptibility to and severity of mpox, and higher knowledge about modes of mpox transmission (Tables 7 and 8). Not intending to share details of every recent sexual contact was associated with preferring not to say how many recent sexual partners you had had, thinking that your life had been negatively affected by changes made in response to the mpox outbreak, and thinking that the risks of mpox had been exaggerated (Tables 7 and 8). No associations reached our threshold for significance in the Grindr sample.

For peer review only - http://bmjopen.bmj.com/site/about/guidelines.xhtml

Table 7. Associations between intending to share details of every sexual contact in the last seven days and socio-demographic characteristics and motivational message, by sample. A higher score indicates greater intention to share details. Variables were entered into the linear regression model in blocks (block 1: socio-demographic variables and motivational message, block 2: psychological factors). Results for block 2, using pooled estimates are reported.

Participant characteristics	Level	General population		Grindr	
		B (95% CI)	*p*-value	*B* (95% CI)	*p*-value
Gender	Male (including trans man)	Ref	-	-	-
	Female (including trans woman)	0.16 (0.07 to 0.24)	<0.001*	-	-
Sexual orientation	Straight or heterosexual	Ref	-	-	-
	Gay, lesbian, bisexual, or queer	0.12 (-0.05 to 0.28)	0.16	-	-
Age	Range 18 to 98 years	0.008 (0.005 to 0.011)	<0.001*	0.003 (-0.005 to 0.011)	0.46
	Quadratic term, (age – mean)^2	-0.00017 (-0.00034 to -0.00001)	0.04	0.0003 (-0.0002 to 0.0008)	0.19
Region	Midlands (East and West)	Ref	-	Ref	-
	South (East, West, East of England)	-0.04 (-0.16 to 0.08)	0.50	0.08 (-0.23 to 0.39)	0.60
	North (East, West, Yorkshire and the Humber)	-0.07 (-0.19 to 0.06)	0.29	0.31 (-0.05 to 0.68)	0.09
	London	0.05 (-0.11 to 0.22)	0.53	0.12 (-0.25 to 0.49)	0.52
	Devolved nations (Scotland, Wales, and Northern Ireland)	0.09 (-0.06 to 0.25)	0.24	0.26 (-0.21 to 0.73)	0.27
Dependent child in household	No	Ref	-	Ref	-
	Yes	-0.12 (-0.22 to -0.01)	0.03	-0.22 (-0.64 to 0.20)	0.30
Employment status	Not working	Ref	-	Ref	-
	Working	-0.10 (-0.20 to 0.00)	0.05	-0.01 (-0.26 to 0.23)	0.90
Frontline health or social care worker	No	Ref	-	Ref	-
	Yes	-0.04 (-0.17 to 0.09)	0.57	0.08 (-0.19 to 0.34)	0.57
Education	GCSE/vocational/A-level/No formal qualifications	Ref	-	Ref	-
	Degree or higher (Bachelors, Masters, PhD)	0.00 (-0.09 to 0.09)	0.97	0.13 (-0.06 to 0.32)	0.19
Ethnicity	White British	Ref	-	Ref	-
	White other	-0.29 (-0.51 to -0.08)	0.008	0.00 (-0.25 to 0.25)	0.98
	Black, Asian, other minoritized ethnicity	-0.17 (-0.32 to -0.01)	0.03	0.01 (-0.29 to 0.30)	0.97
Marital status	Not partnered	Ref	-	Ref	-
	Partnered	0.03 (-0.08 to 0.15)	0.55	0.10 (-0.11 to 0.32)	0.35
Live alone	Live with someone else	Ref	-	Ref	-
	Live alone	-0.04 (-0.17 to 0.08)	0.50	0.21 (0.01 to 0.40)	0.04
Own chronic illness	None	Ref	-	Ref	-
	Present	-0.04 (-0.14 to 0.05)	0.40	0.31 (0.11 to 0.51)	0.003
Ever taken PrEP for HIV	No	-	-	Ref	-
	Yes	-	-	0.09 (-0.10 to 0.28)	0.35
Vaccinated for smallpox in 2022	Not vaccinated	-	-	Ref	-
	Vaccinated	-	-	-0.07 (-0.28 to 0.14)	0.50
Index of multiple deprivation	Deciles (1st = most deprived, 10th = least deprived)	0.00 (-0.02 to 0.02)	0.87	0.03 (-0.02 to 0.08)	0.19
Socio-economic grade	ABC1	Ref	-	Ref	-
	C2DE	-0.12 (-0.21 to -0.03)	0.01	0.04 (-0.22 to 0.29)	0.78

For peer review only - http://bmjopen.bmj.com/site/about/guidelines.xhtml

Financial hardship	4 (lowest hardship) to 13 (most hardship)	-0.05 (-0.07 to -0.03)	<0.001*	-0.02 (-0.07 to 0.04)	0.53
Total number of sexual partners (male and female) in last three weeks	0	Ref	-	-	-
	1	0.06 (-0.05 to 0.17)	0.28	-	-
	2 to 4	0.07 (-0.14 to 0.27)	0.53	-	-
	5 or more	-0.09 (-0.45 to 0.28)	0.64	-	-
	Prefer not to say	-0.37 (-0.49 to -0.24)	<0.001*	-	-
Number of male sexual partners in last three weeks	0	-	-	Ref	-
	1	-	-	-0.02 (-0.27 to 0.22)	0.84
	2 to 4	-	-	-0.02 (-0.24 to 0.21)	0.90
	5 to 9	-	-	-0.08 (-0.38 to 0.23)	0.62
	10 or more	-	-	-0.58 (-0.96 to -0.20)	0.003
	Prefer not to say	-	-	-0.09 (-0.52 to 0.34)	0.67
Motivational message	Perceived risk of illness and necessity and efficacy of the response	-0.03 (-0.15 to 0.08)	0.55	-	-
	Perceived risk of illness and benefits of the response	0.03 (-0.08 to 0.14)	0.59	-	-
	Perceived risk of illness and low perceived costs of response	-0.01 (-0.12 to 0.10)	0.86	-	-
	Control	Ref	-	-	-
Motivational message	All motivational components	-	-	-0.06 (-0.23 to 0.10)	0.45
	Control	-	-	Ref	-

*$p \leq 0.001$

For peer review only

For peer review only - http://bmjopen.bmj.com/site/about/guidelines.xhtml

Table 8. Associations between intending to share details of every sexual contact in the last seven days and psychological and contextual factors, by sample. A higher score indicates greater intention to share details. Variables were entered into the linear regression model in blocks (block 1: socio-demographic variables and motivational message, block 2: psychological factors). Results for block 2, using pooled estimates are reported.

Factor	Level	General population *B* (95% CI)	*p*-value	Grindr *B* (95% CI)	*p*-value
Amount heard about mpox	I have not seen or heard anything (1) to I have seen or heard a lot (3)	-0.04 (-0.13 to 0.05)	0.43	0.01 (-0.18 to 0.20)	0.92
Worry about mpox	Not at all worried (1) to extremely worried (4)	0.08 (0.00 to 0.16)	0.06	0.19 (0.01 to 0.36)	0.03
Perceived risk of mpox to oneself	No risk at all (1) to very high risk (5)	-0.04 (-0.11 to 0.03)	0.27	-0.06 (-0.18 to 0.06)	0.34
Perceived risk of mpox to people in UK	No risk at all (1) to very high risk (5)	0.07 (0.00 to 0.14)	0.06	-0.01 (-0.14 to 0.12)	0.85
Perceived susceptibility and severity	Lowest (1) to highest (5)	0.12 (0.05 to 0.19)	<0.001*	-0.09 (-0.24 to 0.06)	0.24
I am already immune to mpox	Strongly disagree, disagree, neither agree nor disagree, don't know	Ref	-	Ref	-
	Strongly agree and agree	-0.18 (-0.32 to -0.04)	0.01	-0.09 (-0.37 to 0.20)	0.55
People who catch mpox usually make a full recovery, even if they do not receive any treatment	Strongly disagree (1) to strongly agree (5)	-0.01 (-0.06 to 0.04)	0.71	-0.06 (-0.17 to 0.04)	0.23
My personal behaviour has an impact on how mpox spreads	Strongly disagree (1) to strongly agree (5)	0.04 (0.01 to 0.08)	0.03	0.02 (-0.06 to 0.10)	0.64
My life has been negatively affected by changes made in response to the mpox outbreak	Strongly disagree (1) to strongly agree (5)	-0.10 (-0.15 to -0.06)	<0.001*	-0.03 (-0.11 to 0.06)	0.56
The risks of mpox are being exaggerated	Strongly disagree (1) to strongly agree (5)	-0.11 (-0.15 to -0.06)	<0.001*	-0.15 (-0.25 to -0.05)	0.004
Mpox is only a risk to men who are gay, bisexual or have sex with men	Strongly disagree (1) to strongly agree (5)	-0.02 (-0.06 to 0.02)	0.29	-0.07 (-0.16 to 0.01)	0.10
Perceived knowledge	Lowest (0) to highest (3)	0.05 (0.00 to 0.09)	0.04	0.02 (-0.10 to 0.13)	0.78
Knowledge of mpox symptoms	Identified no symptoms (0) to identified four symptoms (4)	0.05 (0.02 to 0.08)	0.004	0.05 (-0.02 to 0.13)	0.14
Knowledge of mpox transmission	Lowest (0) to highest (6)	0.05 (0.02 to 0.08)	<0.001*	0.05 (-0.03 to 0.13)	0.22

*$p \leq 0.001$

For peer review only - http://bmjopen.bmj.com/site/about/guidelines.xhtml

Vaccination

Few people had been vaccinated for smallpox in 2022 in the general population sample (5.3%, 95% CI 4.5% to 6.1%, n=162; Table 2). This was significantly higher in GBMSM samples (10.5% to 40.7%). In a measure of actual and intended vaccination, 96.3% (95% CI 95.2% to 97.5%, n=998) of the Meta sample and 93.5% (95% CI 91.8% to 95.2%, n=777) of the Grindr sample were vaccinated or intended to be vaccinated for smallpox if advised. Rates were significantly lower in the general population and Savanta GBMSM samples.

In the general population, being vaccinated for smallpox in 2022 or intending to be vaccinated if offered a vaccine was associated with being older (aOR 1.015, 95% CI 1.007 to 1.023, p<0.001), more worried about mpox (1.49, 95% CI 1.24 to 1.80, p<0.001), and perceiving a higher susceptibility to and severity of mpox (1.31, 95% CI 1.11 to 1.55, p<0.001; supplementary materials 6). Not intending to be vaccinated was associated with needing to leave home for work (aOR 0.56, 95% CI 0.43 to 0.73, p<0.001) and agreeing that the risks of mpox were being exaggerated (aOR 0.76, 95% CI 0.68 to 0.85, p<0.001; supplementary materials 6).

In the Grindr sample, vaccination uptake was associated with ever having taken PrEP for HIV (aOR 8.95, 95% CI 5.61 to 14.28, p<0.001) and agreeing that you are already immune to mpox (aOR 8.83, 95% CI 4.52 to 17.22, p<0.001; supplementary materials 6). When including vaccination intention, being vaccinated for smallpox in 2022 or intending to be vaccinated if offered a vaccine was associated with agreeing that if you got a smallpox vaccine, you would be protected against mpox (aOR 3.25, 95% CI 1.97 to 5.35, p<0.001; supplementary materials 6).

DISCUSSION

We investigated mpox attitudes, beliefs, and intended behaviours in a general population sample and three GBMSM samples. Samples differed by socio-demographic characteristics. This was reflected in intended behaviours, with GBMSM recruited from Grindr or Meta being more likely to intend to seek help immediately for mpox symptoms, completely stop sexual contact when symptomatic, and be vaccinated for smallpox if advised. High rates of smallpox vaccination in these samples perhaps reflects that they were mostly educated and working, and thus more likely to be health literate and engaged with services. This was also reflected in knowledge about mpox, with Grindr and Meta samples being more likely to correctly identify mpox transmission modes and the symptoms of mpox. Within the general

For peer review only - http://bmjopen.bmj.com/site/about/guidelines.xhtml

population, rates of "don't know" answers were high, with 18.4% selecting "don't know" when asked what the main symptoms of mpox were, and 18.7% to 24.2% selecting "don't know" for transmission modes (except for having sex with someone who has mpox, where 13.9% selected "don't know"). This is similar to other surveys of the general population, where 24% of people were not sure if mpox usually spreads by close contact with an infected person.[45]

There were no effects of motivational messaging on our behavioural outcomes, except for in the Meta sample for intention to self-isolate, share details of all recent sexual contacts, and be vaccinated for smallpox if advised. For these outcomes, intention to engage with protective behaviours was higher in the control group. In practice, however, intentions were very similar (means changed by 0.1 to 0.4) and this is unlikely to represent a meaningful difference. Another study investigating gonorrhoea reinfection in young adults has also found that those in the control group (who received monthly texts reminding them to update their contact details) were less likely to be reinfected than those in the intervention group (who received a series of texts that were educational and used behaviour change techniques).[46] These messages were specifically designed to decrease stigma, and partner numbers were higher in the intervention arm. A systematic review of intervention communications during the COVID-19 pandemic found that messages about the personal and collective benefits of vaccination had mixed effects on vaccination intention, with some suggestions that this approach may be more effective in more strongly hesitant individuals.[28] These results highlight the need for empirical testing of messages on behavioural outcomes, before widespread use in public health.

Generally, higher intentions were seen in the Grindr and Meta samples, except for self-isolation. However, rates should be interpreted with caution, as intended behaviour does not always translate to enacted behaviour.[47] This was shown to be the case for intended and actual engagement with the UK contact tracing programme during the COVID-19 pandemic.[11] Rates of having already been vaccinated for smallpox in 2022 were especially high (32% Grindr, 41% Meta), again suggesting our sample may have been particularly interested in the topic of the survey, especially given issues with access to vaccination.[48] Evidence suggests that while one-off sexual encounters may make up only a minority of sexual interactions, they could account for a large proportion of mpox transmission.[49, 50] Furthermore, if encounters are anonymous, this will affect contact tracing efforts. Therefore, very high rates of intending to completely stop sexual contact with others if symptomatic

For peer review only - http://bmjopen.bmj.com/site/about/guidelines.xhtml

(91% to 93% in Grindr and Meta samples respectively) are encouraging. In the UK, the public health agency (UK Health Security Agency) has been working in conjunction with community-based organisations and charities to raise awareness of mpox and how to prevent the transmission in GBMSM as the population most affected. Higher rates of knowledge and behavioural intentions suggest that this targeted messaging has been effective in increasing knowledge and driving protective behaviours. However, efforts must be taken to ensure that messaging is not stigmatising.[51] Public health efforts in future outbreaks should use a similar model to increase knowledge about transmission and engagement with protective behaviours in affected populations.[31]

It is notable that 19% (Grindr, Meta) to 30% of people (general population, Savanta GBMSM) agreed that is it best to avoid physical contact with GBMSM because of the mpox outbreak, perhaps also reflecting a change in behaviour in this population. Recent decreases in some sexually transmitted infections in GBMSM, and the decline in mpox transmission in England, suggest this may be the case.[6] A study conducted in the USA of men who have sex with men and transgender women found that 56% of participants reported changing their sexual behaviour due to the mpox outbreak, with most participants limiting the number of their sexual partners; 25% of the sample became abstinent or avoided having any type of sex.[52]

Within the general population, women, people who were older, and those with lower financial hardship were more likely to intend to carry out protective behaviours. This pattern was also widely seen during the COVID-19 pandemic [11, 53, 54] and previous outbreaks.[55] This highlights the importance of considering health equity issues to ensure effective outbreak control and has implications for policymakers. Offering financial support for protective behaviours, especially those that may affect people's ability to earn an income such as self-isolation, is likely to increase engagement, especially for those in lower income settings. Few socio-demographic characteristics were associated with outcomes in the Grindr sample. This could be because the Grindr sample differed from the general population sample on key characteristics (more educated, higher socio-economic grade, less financial hardship), or be a function of the way analyses were conducted with all variables being entered into a single regression model. The Grindr sample, being smaller, also had about half the statistical power of the general population sample.

Intention to engage in protective behaviours in the general population was also associated with psychological factors such as greater worry about mpox, perceived risk of mpox to

For peer review only - http://bmjopen.bmj.com/site/about/guidelines.xhtml

others (but there was little evidence of an association with perceived risk to oneself), perceived susceptibility and severity of mpox, and greater knowledge about transmission. This pattern of results was also seen during the COVID-19 and aH1N1 influenza pandemics.[12, 53, 56-58] COVID-19 vaccination intention was also associated with perceived risk to others, but not to oneself.[36] Few associations were significant in the Grindr sample. In addition to the decreased statistical power, this may also reflect the different context and experience that participants in this group had. While for our general population sample the questions about mpox risk were somewhat hypothetical, the Grindr sample were more likely to be impacted by mpox (as illustrated by the high vaccination rates). Factors relating to their personal experience of the outbreak may have superseded any effect of the psychological variables that we assessed. Taken together, these results suggest that clear communications about the level of risk of infection may encourage people to enact protective behaviours when appropriate.

Agreeing that the risks of mpox were being exaggerated was associated with lower intention to engage with protective behaviours. This was also the case in previous pandemics.[53, 59] Other psychological factors associated with decreased intention to engage with protective behaviours were believing that you were already immune to mpox and that your life had been negatively affected by changes made in response to mpox. Attending an important event was one of the reasons given for breaking self-isolation during the aH1N1 pandemic.[60] The peak of the mpox outbreak in the UK occurred in July 2022, a period coinciding with the summer holiday and festival season, including Pride events. It is important to be conscious of asking people to self-isolate or quarantine over periods encompassing large public events that may be happening.

Behaviour-specific beliefs were also associated with intentions. For self-isolation, greater intention was associated with greater social norms (thinking that others would also self-isolate). Greater social norms were associated with fewer outings during lockdown [14] and increased vaccination uptake and intention (in oneself and one's child) [15, 61] during the COVID-19 pandemic. Social norms were also associated with enacting protective behaviours, including self-isolation, during previous outbreaks.[55, 62] Lower self-isolation intention was associated with thinking that self-isolation would have a negative impact on your work. In this study, as in previous research,[11] the main reasons for not being able to self-isolate were needing to go out for essentials (food / medicines), for a walk or some other exercise, and for work. Results suggest that communications emphasising that others are also engaging with

For peer review only - http://bmjopen.bmj.com/site/about/guidelines.xhtml

the protective behaviour may improve engagement with self-isolation. Research investigating the relative importance of different psychological factors and specific needs in behavioural decisions could contribute to the design of more effective communications.

In the UK, people with suspected mpox were directed to call sexual health services. Some people may have found this stigmatising as mpox is not a sexually transmitted infection.[63] Stigma surrounding having other sexually transmitted infections is associated with not seeking help.[64] In our study, immediate help seeking was associated with being willing to contact a sexual health clinic. These findings suggest that widening recommended points of contact with health services to include non-stigmatising routes for suspected mpox cases or contacts may be beneficial and could have increased help seeking.

In the Grindr sample, having been vaccinated for smallpox in 2022 was associated with having ever taken PrEP for HIV. This is probably due to how the vaccine was rolled out in the UK. GBMSM identified by sexual health services as being at highest risk of exposure – using markers similar to those used to assess eligibility for PrEP – were invited to be vaccinated.[65] Being vaccinated was also strongly associated with thinking that you were already immune to mpox in this survey. As this is a cross-sectional survey, we cannot infer the direction of results, but it is likely that respondents believed they were immune to mpox because of their recent vaccination. This is interesting given limited information about the effectiveness of the vaccine at that time.

Strengths of the study include the collection of data from four large samples, including the general population and the population most affected by the recent mpox outbreak (GBMSM). We would also caution readers about several caveats for this study, including that participant socio-demographic characteristics differed by the recruitment method. Participants recruited through Grindr and Meta were more likely to be highly educated, higher socio-economic grade, and have less financial hardship. We are unsure whether this is representative of the socio-demographic characteristics of active Grindr and Meta users as we were unable to find, access, or interpret published statistics of the socio-demographic profile of users.[66] Where published, most statistics outline only age and gender, rather than the additional characteristics we were interested in.[67] High rates of smallpox vaccination in these samples suggest that these groups are likely to be interested in and have personal experience relating to the outbreak and may have been more likely to engage with protective behaviours. We do not know if survey respondents are representative in terms of their beliefs, knowledge and intended behaviours with reference to the general population sample, GBMSM generally, and

For peer review only - http://bmjopen.bmj.com/site/about/guidelines.xhtml

GBMSM who use Grindr and Meta. While overall rates of beliefs, knowledge and intended behaviours may be affected by sampling and should be taken with caution, associations within the data are likely to hold true.[68]

Other limitations relate to the survey and statistical measures used. The use of a cross-sectional survey means that answers may have been influenced by social desirability and recall bias, although the anonymity of a written survey may have mitigated these effects to some degree. Rates of intended behaviour are often higher than enacted behaviours.[11, 47] Therefore, rates of intended behaviours should be taken with caution. Our motivational messages were very brief and only repeated once, before measuring intended behaviours and behaviour-specific attitudes. A fully developed public communications campaign where people are exposed to co-produced and repeated messages may have more influence. There were high rates of actual and intended vaccination in the Grindr sample (94%). This may have affected our ability to detect associations. Variables were entered into regression models in blocks, with results reported for the final block entered (essentially all variables entered together). Therefore, we investigated the independent effect of a variable, accounting for all other variables included.

This study investigated mpox beliefs, knowledge and intended behaviours in a general population sample and in GBMSM (those most affected by the current outbreak). Intended uptake of protective behaviours differed by behaviour. GBMSM generally had higher intention to engage with protective behaviours, apart from self-isolation. This may have been a function of sampling. Higher knowledge about mpox symptoms and transmission in GBMSM samples suggests that the public health messaging carried out by multiple stakeholders including the UK Health Security Agency, charities and via grass-roots community efforts has been successful and a similar model should be used in future outbreaks. There was no impact of additional motivational messaging on intended uptake of protective behaviours. Associations between increased financial hardship and lower intention to enact protective behaviours suggest that providing financial support to those affected in future outbreaks may increase uptake.

For peer review only - http://bmjopen.bmj.com/site/about/guidelines.xhtml

FUNDING SOURCES

LS, JB, RA and GJR are supported by the National Institute for Health and Care Research Health Protection Research Unit (NIHR HPRU) in Emergency Preparedness and Response, a partnership between the UK Health Security Agency, King's College London and the University of East Anglia. Additionally, IO, LY, TM and RA are funded by the NIHR HPRU in Behavioural Science and Evaluation, a partnership between UKHSA and the University of Bristol. The views expressed are those of the authors and not necessarily those of the NIHR, UKHSA, or the Department of Health and Social Care. The study was funded by NIHR. For the purpose of open access, the author has applied a Creative Commons Attribution (CC BY) licence to any Author Accepted Manuscript version arising.

DATA SHARING STATEMENT

Anonymised data are available online.

AUTHOR CONTRIBUTION STATEMENT

LS, HWWP, JB, TM, IO, RA, LY and GJR conceptualised the study and contributed to survey materials. LS completed analyses with guidance from HWWP, JB and GJR. LS wrote the first draft of the manuscript. LS, HWWP, JB, TM, IO, RA, LY and GJR contributed to, and approved, the final manuscript. LS is guarantor. The corresponding author attests that all listed authors meet authorship criteria and that no others meeting the criteria have been omitted.

DISSEMINATION DECLARATION

Dissemination of survey results to participants is not possible due to the anonymous nature of data collection.

COMPETING INTERESTS STATEMENT

HWWP has received additional salary support from Public Health England and NHS England and receives funding from the National Institute of Health Research. HWWP receives consultancy fees to his employer from Ipsos MORI and has a PhD student who works at and has fees paid by AstraZeneca. IO and RA are employees of UKHSA.

For peer review only - http://bmjopen.bmj.com/site/about/guidelines.xhtml

ACKNOWLEDGEMENTS

We would like to thank the Terrence Higgins Trust for their support with participant recruitment, and Savanta for hosting the survey. We would also like to thank study participants, for taking their time to complete the survey.

For peer review only

ETHICS

Ethical approval for this study was given by the King's College London Psychiatry, Nursing and Midwifery Research Ethics Panel (reference number: LRS/DP-21/22-32287). Participants gave informed written consent before beginning survey materials.

For peer review only

For peer review only - http://bmjopen.bmj.com/site/about/guidelines.xhtml

REFERENCES

1. NHS. Monkeypox. 2022 [updated 1 June 2022, accessed 7 June 2022]. Available from: https://www.nhs.uk/conditions/monkeypox/.

2. World Health Organization. Multi-country monkeypox outbreak in non-endemic countries: situation update. 2022 [updated 4 June 2022, accessed 7 June 2022]. Available from: https://www.who.int/emergencies/disease-outbreak-news/item/2022-DON390.

3. World Health Organization. Multi-country outbreak of mpox. 2023 [updated 14 August 2023, accessed 24 August 2023]. Available from: https://www.who.int/publications/m/item/multi-country-outbreak-of-mpox--external-situation-report-27---14-august-2023.

4. UK Health Security Agency. Monkeypox cases confirmed in England – latest updates. 2022 [updated 7 June 2022, accessed 7 June 2022]. Available from: https://www.gov.uk/government/news/monkeypox-cases-confirmed-in-england-latest-updates.

5. UK Health Security Agency. Mpox (monkeypox) outbreak: epidemiological overview, 20 December 2022. 2023 [updated 23 August 2023, accessed 24 August 2023]. Available from: https://www.gov.uk/government/publications/monkeypox-outbreak-epidemiological-overview/mpox-monkeypox-outbreak-epidemiological-overview-20-december-2022.

6. UK Health Security Agency. Investigation into monkeypox outbreak in England: technical briefing 8. 2022 [updated 23 September 2022, accessed 31 October 2022]. Available from: https://www.gov.uk/government/publications/monkeypox-outbreak-technical-briefings/investigation-into-monkeypox-outbreak-in-england-technical-briefing-8#part-2-epidemiology-update.

7. UK Health Security Agency. Monkeypox contact tracing guidance: classification of contacts and follow-up advice for non-HCID strains of monkeypox. 2022 [updated 27 September 2022, accessed 6 October 2022]. Available from: https://assets.publishing.service.gov.uk/government/uploads/system/uploads/attachment_data/file/1106691/monkeypox-contact-tracing-classification-and-vaccination-matrix-version-13-27-sept-2022.pdf.

For peer review only - http://bmjopen.bmj.com/site/about/guidelines.xhtml

8. UK Health Security Agency. Recommendations for the use of pre and post exposure vaccination during a monkeypox incident (updated May 2022 v6.6). 21 May 2022. Available from: https://assets.publishing.service.gov.uk/government/uploads/system/uploads/attachment_data/file/1077678/Recommendations-for-use-of-pre-and-post-exposure-vaccination-during-a-monkeypox-incident.pdf.

9. UKHSA finds vaccination offers strong protection against monkeypox [press release]. 22 November 2022. Available from: https://www.gov.uk/government/news/ukhsa-finds-vaccination-offers-strong-protection-against-monkeypox.

10. Smith LE, Potts HW, Amlot R, Fear NT, Michie S, Rubin GJ. Who is engaging with lateral flow testing for COVID-19 in the UK? The COVID-19 Rapid Survey of Adherence to Interventions and Responses (CORSAIR) study. BMJ Open. 2022;12(2):e058060.

11. Smith LE, Potts HWW, Amlot R, Fear NT, Michie S, Rubin GJ. Adherence to the test, trace, and isolate system in the UK: results from 37 nationally representative surveys. BMJ. 2021;372:n608.

12. Smith LE, Potts HWW, Amlot R, Fear NT, Michie S, Rubin GJ. Intention to adhere to test, trace, and isolate during the COVID-19 pandemic (the COVID-19 Rapid Survey of Adherence to Interventions and Responses study). Br J Health Psychol. 2021.

13. Rogers RW. A Protection Motivation Theory of Fear Appeals and Attitude Change. J Psychol. 1975;91(1):93-114.

14. Smith LE, Amlot R, Lambert H, Oliver I, Robin C, Yardley L, et al. Factors associated with adherence to self-isolation and lockdown measures in the UK: a cross-sectional survey. Public Health. 2020;187:41-52.

15. Smith LE, Sim J, Cutts M, Dasch H, Amlôt R, Sevdalis N, et al. Psychosocial factors affecting COVID-19 vaccine uptake in the UK: A prospective cohort study (CoVAccS – Wave 3). Vaccine: X. 2023;13:100276.

16. Sherman SM, Sim J, Cutts M, Dasch H, Amlot R, Rubin GJ, et al. COVID-19 vaccination acceptability in the UK at the start of the vaccination programme: a nationally representative cross-sectional survey (CoVAccS - wave 2). Public Health. 2022;202:1-9.

For peer review only - http://bmjopen.bmj.com/site/about/guidelines.xhtml

17. Paul E, Steptoe A, Fancourt D. Attitudes towards vaccines and intention to vaccinate against COVID-19: Implications for public health communications. Lancet Reg Health Eur. 2021;1:100012.

18. Butter S, McGlinchey E, Berry E, Armour C. Psychological, social, and situational factors associated with COVID-19 vaccination intentions: A study of UK key workers and non-key workers. Br J Health Psychol. 2022;27(1):13-29.

19. Bish A, Yardley L, Nicoll A, Michie S. Factors associated with uptake of vaccination against pandemic influenza: a systematic review. Vaccine. 2011;29(38):6472-84.

20. Malik AA, Winters MS, Omer SB. Attitudes of the US general public towards Monkeypox. medRxiv. 2022:2022.06.20.22276527.

21. Temsah MH, Aljamaan F, Alenezi S, Abouammoh N, Alhasan K, Dasuqi SA, et al. Monkeypox Disease (MPOX) Perceptions among Healthcare Workers versus General Population during the First Month of the WHO Alert: Cross-Sectional Survey in Saudi Arabia. Vaccines (Basel). 2022;10(12).

22. Paparini S, Whitacre R, Smuk M, Thornhill J, Mwendera C, Strachan S, et al. Public understanding and awareness of and response to monkeypox virus outbreak: A cross-sectional survey of the most affected communities in the United Kingdom during the 2022 public health emergency. HIV Medicine. 2023;24(5):544-57.

23. Temsah M-H, Aljamaan F, Alenezi S, Alhasan K, Saddik B, Al-Barag A, et al. Monkeypox caused less worry than COVID-19 among the general population during the first month of the WHO Monkeypox alert: Experience from Saudi Arabia. Travel Medicine and Infectious Disease. 2022;49:102426.

24. Dukers-Muijrers NH, Evers Y, Widdershoven V, Davidovich U, Adam PC, Op de Coul EL, et al. Mpox vaccination willingness, determinants, and communication needs in gay, bisexual, and other men who have sex with men, in the context of limited vaccine availability in the Netherlands (Dutch Mpox-survey). Frontiers in Public Health. 2023;10(1058807).

25. Wang H, Abreu de Paulo KJI, Gültzow T, Zimmermann HML, Jonas KJ. Monkeypox self-diagnosis abilities, determinants of vaccination and self-isolation intention after diagnosis among MSM, the Netherlands, July 2022. Eurosurveillance. 2022;27(33):2200603.

For peer review only - http://bmjopen.bmj.com/site/about/guidelines.xhtml

26. Paparini S, Whitacre R, Smuk M, Thornhill J, Mwendera C, Strachan S, et al. Public understanding, awareness, and response to monkeypox virus outbreak: A cross-sectional survey of the most affected communities in the United Kingdom during the 2022 public health emergency. Personal communication.

27. Kapur GB, Bezek S, Dyal J. Effective communication during disasters : making use of technology, media, and human resources. Oakville: Apple Academic Press,; 2017.

28. Batteux E, Mills F, Jones LF, Symons C, Weston D. The Effectiveness of Interventions for Increasing COVID-19 Vaccine Uptake: A Systematic Review. Vaccines. 2022;10(3):386.

29. Hanum N, Cambiano V, Sewell J, Rodger AJ, Nwokolo N, Asboe D, et al. Trends in HIV incidence between 2013–2019 and association of baseline factors with subsequent incident HIV among gay, bisexual, and other men who have sex with men attending sexual health clinics in England: A prospective cohort study. PLOS Medicine. 2021;18(6):e1003677.

30. UKHSA latest findings into monkeypox outbreak [press release]. 1 June 2022. Available from: https://www.gov.uk/government/news/ukhsa-latest-findings-into-monkeypox-outbreak.

31. WHO Regional Office for Europe. Holding mass and large gathering events during the multi-country mpox outbreak in the WHO European Region: Lessons identified for future mass gathering preparedness; meeting report, 22 February 2023. Copenhagen; 2023.

32. Office for National Statistics. Mid-Year Population Estimates, UK, June 2020. 2021 [updated 25 June 2021, accessed 14 October 2022]. Available from: https://www.ons.gov.uk/peoplepopulationandcommunity/populationandmigration/populationestimates/datasets/populationestimatesforukenglandandwalesscotlandandnorthernireland.

33. Rubin GJ, Amlôt R, Page L, Pearce J, Wessely S. Assessing Perceptions AbouT Hazardous Substances (PATHS): The PATHS questionnaire. Journal of Health Psychology. 2013;18(8):1100-13.

34. Rubin GJ, Bakhshi S, Amlôt R, Fear N, Potts HWW, Michie S. The design of a survey questionnaire to measure perceptions and behaviour during an influenza pandemic: the Flu TElephone Survey Template (FluTEST). Health Services and Delivery Research. 2014;2(41).

For peer review only - http://bmjopen.bmj.com/site/about/guidelines.xhtml

35. Smith LE, Potts HWW, Amlot R, Fear NT, Michie S, Rubin GJ. Holding a stigmatizing attitude at the start of the COVID-19 outbreak: A cross-sectional survey. Br J Health Psychol. 2021.

36. Sherman SM, Smith LE, Sim J, Amlot R, Cutts M, Dasch H, et al. COVID-19 vaccination intention in the UK: results from the COVID-19 vaccination acceptability study (CoVAccS), a nationally representative cross-sectional survey. Hum Vaccin Immunother. 2021;17(6):1612-21.

37. World Health Organization. Monkeypox. 2022 [updated 19 May 2022, accessed 6 June 2022]. Available from: https://www.who.int/news-room/fact-sheets/detail/monkeypox.

38. UK Health Security Agency. GUMCAD STI Surveillance System: Data specification and technical guidance. 2021. Available from: https://assets.publishing.service.gov.uk/government/uploads/system/uploads/attachment_data/file/1032965/GUMCAD_data_specification_and_technical_guidance_2021.pdf.

39. Office for National Statistics. Sexual orientation question development for Census 2021. No date. Available from: https://www.ons.gov.uk/census/censustransformationprogramme/questiondevelopment/sexualorientationquestiondevelopmentforcensus2021#:~:text=Census%202021%20will%20be%20the,at%20the%20sexual%20orientation%20question.

40. Ipsos MediaCT. Social Grade: A Classification Tool. 2009. Available from: https://www.ipsos.com/sites/default/files/publication/6800-03/MediaCT_thoughtpiece_Social_Grade_July09_V3_WEB.pdf.

41. Organisation for Economic Co-operation and Development. Supplementary Questions: Optional Survey Questions for the OECD INFE Financial Literacy Core Questionnaire. 2012. Available from: https://www.oecd.org/finance/financial-education/49878153.pdf.

42. Ministry of Housing Communities & Local Government. English indices of deprivation 2019. 2019 [updated 26 September 2019, accessed 17 October 2022]. Available from: https://www.gov.uk/government/statistics/english-indices-of-deprivation-2019.

43. Chen H, Cohen P, Chen S. How Big is a Big Odds Ratio? Interpreting the Magnitudes of Odds Ratios in Epidemiological Studies. Communications in Statistics - Simulation and Computation. 2010;39(4):860-4.

For peer review only - http://bmjopen.bmj.com/site/about/guidelines.xhtml

[dataset] 44. Smith LE, Yardley L, Rubin J, May T. Mpox: Investigating public perceptions, understanding, and behaviours; Quantitative survey. Open Science Framework, 15 May 2023. https://doi.org/10.17605/OSF.IO/AD7YJ.

45. Annenberg Public Policy Center. Survey: 1 in 5 Americans Fear Getting Monkeypox but Many Know Little About It. 2022 [updated 29 July 2022, accessed 31 October 2022]. Available from: https://www.asc.upenn.edu/news-events/news/survey-1-5-americans-fear-getting-monkeypox-many-know-little-about-it.

46. Free C, Palmer MJ, McCarthy OL, Jerome L, Berendes S, Knight M, et al. Effectiveness of a behavioural intervention delivered by text messages (safetxt) on sexually transmitted reinfections in people aged 16-24 years: randomised controlled trial. BMJ. 2022;378:e070351.

47. Sniehotta FF, Scholz U, Schwarzer R. Bridging the intention–behaviour gap: Planning, self-efficacy, and action control in the adoption and maintenance of physical exercise. Psychology & Health. 2005;20(2):143-60.

48. Josh Parry. Monkeypox: Lack of vaccines sparks pause in UK rollout. BBC News Online. 2022 [updated 15 August 2022, accessed 11 November 2022]. Available from: https://www.bbc.co.uk/news/uk-62551821.

49. Spicknall IH, Pollock ED, Clay PA, Oster AM, Charniga K, Masters N, et al. Modeling the Impact of Sexual Networks in the Transmission of Monkeypox virus Among Gay, Bisexual, and Other Men Who Have Sex with Men - United States, 2022. MMWR Morb Mortal Wkly Rep. 2022;71(35):1131-5.

50. Endo A, Murayama H, Abbott S, Ratnayake R, Pearson CAB, Edmunds WJ, et al. Heavy-tailed sexual contact networks and monkeypox epidemiology in the global outbreak, 2022. Science. 2022;378(6615):90-4.

51. Owens, C. and R.D. Hubach. An Exploratory Study of the Mpox Media Consumption, Attitudes, and Preferences of Sexual and Gender Minority People Assigned Male at Birth in the United States. LGBT Health, 2023. 10(5), 401-407.

52. Hubach, R.D. and C. Owens. Findings on the Monkeypox Exposure Mitigation Strategies Employed by Men Who Have Sex with Men and Transgender Women in the United States. Archives of Sexual Behavior, 2022. 51(8), 3653-3658.

For peer review only - http://bmjopen.bmj.com/site/about/guidelines.xhtml

53. Smith LE, Potts HWW, Amlôt R, Fear NT, Michie S, Rubin GJ. Patterns of social mixing in England changed in line with restrictions during the COVID-19 pandemic (September 2020 to April 2022). Scientific Reports. 2022;12(1):10436.

54. Wright L, Steptoe A, Fancourt D. Patterns of compliance with COVID-19 preventive behaviours: a latent class analysis of 20 000 UK adults. J Epidemiol Community Health. 2022;76(3):247-53.

55. Bish A, Michie S. Demographic and attitudinal determinants of protective behaviours during a pandemic: A review. British Journal of Health Psychology. 2010;15(4):797-824.

56. Smith LE, Potts HWW, Amlot R, Fear NT, Michie S, Rubin GJ. Worry and behaviour at the start of the COVID-19 outbreak: Results from three UK surveys (the COVID-19 rapid survey of Adherence to Interventions and responses [CORSAIR] study). Prev Med Rep. 2022;25:101686.

57. Dryhurst S, Schneider CR, Kerr J, Freeman ALJ, Recchia G, van der Bles AM, et al. Risk perceptions of COVID-19 around the world. Journal of Risk Research. 2020;23(7-8):994-1006.

58. Rubin GJ, Potts HW, Michie S. The impact of communications about swine flu (influenza A H1N1v) on public responses to the outbreak: results from 36 national telephone surveys in the UK. Health Technol Assess. 2010;14(34):183-266.

59. Rubin GJ, Amlôt R, Page L, Wessely S. Public perceptions, anxiety, and behaviour change in relation to the swine flu outbreak: cross sectional telephone survey. BMJ. 2009;339:b2651.

60. Teh B, Olsen K, Black J, Cheng AC, Aboltins C, Bull K, et al. Impact of swine influenza and quarantine measures on patients and households during the H1N1/09 pandemic. Scandinavian Journal of Infectious Diseases. 2012;44(4):289-96.

61. Smith LE, Sherman SM, Sim J, Amlôt R, Cutts M, Dasch H, et al. Parents' intention to vaccinate their child for COVID-19: A mixed-methods study (CoVAccS-wave 3). PLoS One. 2022;17(12):e0279285.

62. Webster RK, Brooks SK, Smith LE, Woodland L, Wessely S, Rubin GJ. How to improve adherence with quarantine: rapid review of the evidence. Public Health. 2020;182:163-9.

For peer review only - http://bmjopen.bmj.com/site/about/guidelines.xhtml

63. März JW, Holm S, Biller-Andorno N. Monkeypox, stigma and public health. The Lancet Regional Health – Europe. 2022;23.

64. Stangl AL, Pliakas T, Izazola-Licea JA, Ayala G, Beattie TS, Ferguson L, et al. Removing the societal and legal impediments to the HIV response: An evidence-based framework for 2025 and beyond. PLOS ONE. 2022;17(2):e0264249.

65. Joint Committee on Vaccination and Immunisation. JCVI statement on vaccine dose prioritisation in response to the monkeypox outbreak. 2022 [updated 23 September 2022, accessed 11 November 2022]. Available from: https://www.gov.uk/government/publications/monkeypox-outbreak-jcvi-statement-on-vaccine-dose-prioritisation-september-2022/jcvi-statement-on-vaccine-dose-prioritisation-in-response-to-the-monkeypox-outbreak.

66. Kunst A. Grindr brand profile in the UK 2022: Statista; 2022 [updated 13 December 2022, accessed 24 April 2023]. Available from: https://www.statista.com/forecasts/1335156/grindr-dating-brand-profile-in-the-uk.

67. Dixon S. Meta audiences in the United Kingdom 2023, by age and gender: Statista; 2023 [updated 24 February 2023, accessed 24 April 2023]. Available from: https://www.statista.com/statistics/1315413/uk-meta-audiences-by-age-and-gender/.

68. Kohler U. Possible Uses of Nonprobability Sampling for the Social Sciences. Survey Methods: Insights from the Field. 2019.

For peer review only

For peer review only - http://bmjopen.bmj.com/site/about/guidelines.xhtml

Supplementary materials 1. Full survey materials and top-line results for general population and GBMSM samples separately

For categorical data, n (%) are shown. Percentages are column percentages. Where totals do not add up to 100%, it is due to rounding errors.

For continuous data, n (where different to total sample), mean (M), standard deviation (SD), and range are shown.

Total *ns* are: General population, n=3050; Savanta GBMSM, n=247; Grindr, n=831; Meta [Facebook and Instagram], n=1036.

For peer review only

Screening questions

ASK ALL
Postcode What is your full postcode?

We will only use your postcode to allocate you to groups based on your location (e.g. region). Your full postcode will not be passed on to the research team.

Type your answer below
OPEN END

OPEN TEXT ENTRY
Prefer not to say

New screen

ASK ALL
Gender What is your gender?
SINGLE CODE

	General population	Savanta GBMSM	Grindr	Meta
Male (including trans man)	1282 (42.0)	247 (100.0)	827 (99.5)	1030 (99.4)
Female (including trans woman)	1729 (56.7)	0 (0.0)	0 (0.0)	0 (0.0)
Non-binary	17 (0.6)	0 (0.0)	0 (0.0)	0 (0.0)
Other, please state	15 (0.5)	0 (0.0)	4 (0.5)	6 (0.6)
Prefer not to say	7 (0.2)	0 (0.0)	0 (0.0)	0 (0.0)

** For main analyses, where possible, text answers from "other, please state" were used to assign participants to a group. Therefore, numbers presented in the main manuscript may differ slightly.*

New screen

ASK ALL
SexBirth Is your gender the same as your gender assigned at birth?
SINGLE CODE

	General population	Savanta GBMSM	Grindr	Meta
Yes	3022 (99.1)	238 (96.4)	828 (99.6)	1013 (97.8)
No	22 (0.7)	9 (3.6)	2 (0.2)	18 (1.7)
Prefer not to say	6 (0.2)	0 (0.0)	1 (0.1)	5 (0.5)

New screen

ASK ALL
Age How old are you (in years)?
Type your answer below
NUMERIC - OPEN END

	General population	Savanta GBMSM	Grindr	Meta
OPEN TEXT ENTRY – number [cap at 110]	M=48.6, SD=17.4, range 18 to 98.	M=47.1, SD=16.5, range 18 to 77.	M=44.2, SD=12.5, range 18 to 82.	M=47.6, SD=11.9, range 18 to 79.

SCREEN OUT if under 18 years.

New screen

ASK ALL
Sexuality Which of the following best describes your sexual orientation?
SINGLE CODE, RANDOMISE

	General population	Savanta GBMSM	Grindr	Meta
Straight or heterosexual	2795 (91.6)	0 (0.0)	0 (0.0)	0 (0.0)
Gay or lesbian	78 (2.6)	230 (93.1)	706 (85.0)	967 (93.3)
Bisexual	135 (4.4)	16 (6.5)	118 (14.2)	55 (5.3)
Other sexual orientation, please state [ANCHOR]	18 (0.6)	1 (0.4)	7 (0.8)	14 (1.4)
Prefer not to say	24 (0.8)	0 (0.0)	0 (0.0)	0 (0.0)

For peer review only - http://bmjopen.bmj.com/site/about/guidelines.xhtml

[ANCHOR]				

** For main analyses, where possible, text answers from "other, please state" were used to assign participants to a group. Therefore, numbers presented in the main manuscript may differ slightly.*

New screen

ASK ALL

SEG Which of the following best describes the profession of the chief income earner in your household?

SINGLE CODE

	General population	Savanta GBMSM	Grindr	Meta
**High managerial, administrative or professional** e.g. doctor, lawyer, medium / large company director (50+ people)	193 (6.3)	28 (11.3)	144 (17.3)	253 (24.4)
**Intermediate managerial, administrative or professional** e.g. teacher, manager, accountant	608 (19.9)	57 (23.1)	308 (37.1)	381 (36.8)
**Supervisor, administrative or professional** e.g. police officer, nurse, secretary, self-employed	628 (20.6)	51 (20.6)	175 (21.1)	195 (18.8)
**Skilled manual worker** e.g. mechanic, plumber, electrician, lorry driver, train driver	395 (13.0)	17 (6.9)	42 (5.1)	18 (1.7)
**Semi-skilled or unskilled manual worker** e.g. waiter, factory worker, receptionist, labourer	328 (10.8)	18 (7.3)	45 (5.4)	31 (3.0)
**House-wife / house-husband**	76 (2.5)	3 (1.2)	4 (0.5)	0.0 (0.0)
**Unemployed**	176 (5.8)	24 (9.7)	27 (3.2)	26 (2.5)
**Student**	52 (1.7)	5 (2.0)	30 (3.6)	32 (3.1)
**Retired on a state pension**	215 (7.0)	9 (3.6)	15 (1.8)	13 (1.3)
**Retired on a private pension**	379 (12.4)	35 (14.2)	41 (4.9)	87 (8.4)

For peer review only - http://bmjopen.bmj.com/site/about/guidelines.xhtml

For peer review only

New screen

ASK ALL
QC This is a quality control question.

What colour is grass usually?

SINGLE CODE, RANDOMISE ORDER

	General population	Savanta GBMSM	Grindr	Meta
Purple	0 (0.0)	0 (0.0)	0 (0.0)	0 (0.0)
Pink	0 (0.0)	0 (0.0)	0 (0.0)	0 (0.0)
Orange	0 (0.0)	0 (0.0)	0 (0.0)	0 (0.0)
Green	3050 (100.0)	247 (100.0)	831 (100.0)	1036 (100.0)
White	0 (0.0)	0 (0.0)	0 (0.0)	0 (0.0)
Black	0 (0.0)	0 (0.0)	0 (0.0)	0 (0.0)

SCREEN OUT if do not select "green".

Beliefs and attitudes

New screen

There is an infection called "monkeypox" that has affected some people in the UK. We are interested to know your personal opinion about monkeypox, based on what you currently know.

For each question, please select the answer that reflects your opinion about monkeypox. Do not worry if you do not know what the best answer might be or if you are not at all familiar with monkeypox. We only ask that you try to give your answer based on what you think you know or what you would honestly decide to do in the situations described. If you are really not sure, please answer "don't know".

New screen

Q1. Before today, how much have you seen or heard about monkeypox?

	General population	Savanta GBMSM	Grindr	Meta
I have not seen or heard anything	117 (3.8)	6 (2.4)	7 (0.8)	2 (0.2)
I have seen or heard a little	2164 (71.0)	136 (55.1)	385 (46.3)	287 (27.7)
I have seen or heard a lot	759 (24.9)	103 (41.7)	439 (52.8)	747 (72.1)
Don't know	10 (0.3)	2 (0.8)	0 (0.0)	0 (0.0)

New screen

For peer review only - http://bmjopen.bmj.com/site/about/guidelines.xhtml

ASK ALL
Q2. Overall, how worried are you about monkeypox?
SINGLE CODE

	General population	Savanta GBMSM	Grindr	Meta
Not at all worried	635 (20.8)	40 (16.2)	77 (9.3)	47 (4.5)
Not very worried	1763 (57.8)	125 (50.6)	469 (56.4)	500 (48.3)
Very worried	452 (14.8)	61 (24.7)	234 (28.2)	410 (39.6)
Extremely worried	129 (4.2)	13 (5.3)	38 (4.6)	72 (6.9)
Don't know	71 (2.3)	8 (3.2)	13 (1.6)	7 (0.7)

New screen

ASK ALL
Q3. How much risk do you think monkeypox currently poses to:
Please select one option for each answer
RANDOMISE order of presentation of statements

People in the UK?

	General population	Savanta GBMSM	Grindr	Meta
No risk at all	106 (3.5)	9 (3.6)	11 (1.3)	3 (0.3)
Low risk	1537 (50.4)	102 (41.3)	357 (43.0)	427 (41.2)
Medium risk	1058 (34.7)	97 (39.3)	337 (40.6)	426 (41.1)
High risk	186 (6.1)	28 (11.3)	92 (11.1)	153 (14.8)
Very high risk	75 (2.5)	6 (2.4)	17 (2.0)	22 (2.1)
Don't know	88 (2.9)	5 (2.0)	17 (2.0)	5 (0.5)

You personally?

	General population	Savanta GBMSM	Grindr	Meta
No risk at all	700 (23.0)	48 (19.4)	42 (5.1)	44 (4.2)
Low risk	1619 (53.1)	96 (38.9)	291 (35.0)	266 (25.7)
Medium risk	441 (14.5)	71 (28.7)	303 (36.5)	332 (32.0)
High risk	136 (4.5)	15 (6.1)	148 (17.8)	299 (28.9)
Very high risk	58 (1.9)	13 (5.3)	37 (4.5)	93 (9.0)
Don't know	96 (3.1)	4 (1.6)	10 (1.2)	2 (0.2)

New screen

ASK ALL
Q4. How much, if at all, have you seen or heard about monkeypox? From:

The news (TV, online news websites or apps, printed newspapers, or radio)

	General population	Savanta GBMSM	Grindr	Meta

For peer review only - http://bmjopen.bmj.com/site/about/guidelines.xhtml

Not at all	210 (6.9)	14 (5.7)	61 (7.3)	54 (5.2)
A little	2125 (69.7)	149 (60.3)	533 (64.1)	652 (62.9)
A lot	693 (22.7)	82 (33.2)	235 (28.3)	330 (31.9)
Don't know	22 (0.7)	2 (0.8)	2 (0.2)	0 (0.0)

Official websites or helplines (e.g. NHS, GOV.UK), or an NHS GP practice, clinic or hospital

	General population	Savanta GBMSM	Grindr	Meta
Not at all	1547 (50.7)	98 (39.7)	198 (23.8)	188 (18.1)
A little	1113 (36.5)	97 (39.3)	450 (54.2)	601 (58.0)
A lot	316 (10.4)	48 (19.4)	169 (20.3)	241 (23.3)
Don't know	74 (2.4)	4 (1.6)	14 (1.7)	6 (0.6)

Speaking to friends, family, colleagues or other people you know (in person, by phone, text, WhatsApp, email, or in other ways)

	General population	Savanta GBMSM	Grindr	Meta
Not at all	1469 (48.2)	111 (44.9)	263 (31.6)	218 (21.0)
A little	1235 (40.5)	87 (35.2)	388 (46.7)	466 (45.0)
A lot	300 (9.8)	44 (17.8)	175 (21.1)	350 (33.8)
Don't know	46 (1.5)	5 (2.0)	5 (0.6)	2 (0.2)

Workers in community groups, community or faith leaders, charities, or volunteers who help improve the health and wellbeing of others

	General population	Savanta GBMSM	Grindr	Meta
Not at all	2111 (69.2)	165 (66.8)	553 (66.5)	633 (61.1)
A little	633 (20.8)	53 (21.5)	162 (19.5)	253 (24.4)
A lot	220 (7.2)	25 (10.1)	58 (7.0)	113 (10.9)
Don't know	86 (2.8)	4 (1.6)	58 (7.0)	37 (3.6)

Other places, including blogs, social media sites (e.g. Facebook, Twitter, Instagram), online communities or other websites

	General population	Savanta GBMSM	Grindr	Meta
Not at all	1426 (46.8)	100 (40.5)	214 (25.8)	138 (13.3)
A little	1215 (39.8)	100 (40.5)	427 (51.4)	534 (51.5)
A lot	357 (11.7)	43 (17.4)	179 (21.5)	361 (34.8)
Don't know	52 (1.7)	4 (1.6)	11 (1.3)	3 (0.3)

New screen

Q4_other. Please state any other places that you have seen or heard about monkeypox from.

Open ended, text entry

None button

New screen

For peer review only - http://bmjopen.bmj.com/site/about/guidelines.xhtml

For each question, please select the answer that reflects your opinion about monkeypox. Do not worry if you do not know what the best answer might be or if you are not at all familiar with monkeypox. We only ask that you try to give your answer based on what you think you know. If you are really not sure, please answer “don’t know”.

New screen

ASK ALL

Q5. How much do you agree or disagree with the following statements:

RANDOMISE statements

I have a good idea of how people catch monkeypox

	General population	Savanta GBMSM	Grindr	Meta
Strongly disagree	252 (8.2)	14 (5.6)	27 (3.2)	15 (1.4)
Disagree	727 (23.8)	35 (14.1)	46 (5.5)	31 (2.9)
Neither agree nor disagree	590 (19.3)	32 (12.9)	82 (9.8)	57 (5.5)
Agree	1159 (38.0)	121 (48.9)	468 (56.3)	559 (53.9)
Strongly agree	217 (7.1)	41 (16.5)	196 (23.5)	371 (35.8)
Don’t know	105 (3.4)	4 (1.6)	12 (1.4)	3 (0.2)

I know what the main symptoms of monkeypox are

	General population	Savanta GBMSM	Grindr	Meta
Strongly disagree	263 (8.6)	16 (6.5)	38 (4.6)	17 (1.6)
Disagree	820 (26.9)	44 (17.8)	100 (12.0)	60 (5.8)
Neither agree nor disagree	590 (19.3)	41 (16.6)	106 (12.8)	108 (10.4)
Agree	1113 (36.5)	120 (48.6)	493 (59.3)	642 (62.0)
Strongly agree	152 (5.0)	23 (9.3)	80 (9.6)	204 (19.7)
Don’t know	112 (3.7)	3 (1.2)	14 (1.7)	5 (0.5)

It would be easy for me to tell if someone I meet has monkeypox

	General population	Savanta GBMSM	Grindr	Meta
Strongly disagree	485 (15.9)	33 (13.4)	149 (17.9)	205 (19.8)
Disagree	1123 (36.8)	71 (28.7)	369 (44.4)	498 (48.1)
Neither agree nor disagree	663 (21.7)	64 (25.9)	181 (21.8)	209 (20.2)
Agree	504 (16.5)	54 (21.9)	88 (10.6)	92 (8.9)
Strongly agree	119 (3.9)	14 (5.7)	15 (1.8)	15 (1.4)
Don’t know	156 (5.1)	11 (4.5)	29 (3.5)	17 (1.6)

New screen

For peer review only - http://bmjopen.bmj.com/site/about/guidelines.xhtml

ASK ALL

Q6. How much do you agree or disagree with the following statements:

RANDOMISE statements

In the near future, it is likely that some of the people I come into physical contact with (touch) will have monkeypox

	General population	Savanta GBMSM	Grindr	Meta
Strongly disagree	275 (9.0)	13 (5.3)	23 (2.8)	23 (2.2)
Disagree	743 (24.4)	56 (22.7)	144 (17.3)	145 (14.0)
Neither agree nor disagree	912 (29.9)	65 (26.3)	237 (28.5)	238 (23.0)
Agree	632 (20.7)	73 (29.6)	297 (35.7)	470 (45.4)
Strongly agree	112 (3.7)	12 (4.9)	43 (5.2)	118 (11.4)
Don't know	376 (12.3)	28 (11.3)	87 (10.5)	42 (4.1)

If I come into physical contact with (touch) someone who has monkeypox, it is likely that I will catch it

	General population	Savanta GBMSM	Grindr	Meta
Strongly disagree	108 (3.5)	5 (2.0)	20 (2.4)	9 (0.9)
Disagree	467 (15.3)	24 (9.7)	93 (11.2)	145 (14.0)
Neither agree nor disagree	814 (26.7)	59 (23.9)	167 (20.1)	207 (20.0)
Agree	944 (31.0)	99 (40.1)	376 (45.2)	497 (48.0)
Strongly agree	207 (6.8)	25 (10.1)	98 (11.8)	135 (13.0)
Don't know	510 (16.7)	35 (14.2)	77 (9.3)	43 (4.2)

Monkeypox would be a serious illness for me

	General population	Savanta GBMSM	Grindr	Meta
Strongly disagree	224 (7.3)	16 (6.5)	30 (3.6)	26 (2.5)
Disagree	546 (17.9)	35 (14.2)	144 (17.3)	160 (15.4)
Neither agree nor disagree	744 (24.4)	58 (23.5)	182 (21.9)	220 (21.2)
Agree	787 (25.8)	85 (34.4)	290 (34.9)	413 (39.9)
Strongly agree	274 (9.0)	28 (11.3)	116 (14.0)	166 (16.0)
Don't know	475 (15.6)	25 (10.1)	69 (8.3)	51 (4.9)

People who catch monkeypox usually make a full recovery, even if they do not receive any treatment

	General population	Savanta GBMSM	Grindr	Meta
Strongly disagree	52 (1.7)	0 (0.0)	11 (1.3)	6 (0.6)
Disagree	264 (8.7)	25 (10.1)	77 (9.3)	62 (6.0)
Neither agree nor disagree	844 (27.7)	56 (22.7)	117 (14.1)	144 (13.9)
Agree	977 (32.0)	96 (38.9)	383 (46.1)	530 (51.2)

For peer review only - http://bmjopen.bmj.com/site/about/guidelines.xhtml

Strongly agree	228 (7.5)	29 (11.7)	102 (12.3)	210 (20.3)
Don't know	685 (22.5)	41 (16.6)	141 (17.0)	84 (8.1)

My personal behaviour has an impact on how monkeypox spreads

	General population	Savanta GBMSM	Grindr	Meta
Strongly disagree	498 (16.3)	24 (9.7)	53 (6.4)	28 (2.7)
Disagree	581 (19.0)	31 (12.6)	78 (9.4)	71 (6.9)
Neither agree nor disagree	572 (18.8)	40 (16.2)	91 (11.0)	67 (6.5)
Agree	834 (27.3)	95 (38.5)	384 (46.2)	552 (53.3)
Strongly agree	278 (9.1)	45 (18.2)	191 (23.0)	312 (30.1)
Don't know	287 (9.4)	12 (4.9)	34 (4.1)	6 (0.6)

My life has been negatively affected by changes made in response to the monkeypox outbreak

	General population	Savanta GBMSM	Grindr	Meta
Strongly disagree	1181 (38.7)	90 (36.4)	167 (20.1)	160 (15.4)
Disagree	981 (32.2)	75 (30.4)	289 (34.8)	300 (29.0)
Neither agree nor disagree	420 (13.8)	37 (15.0)	168 (20.2)	217 (20.9)
Agree	246 (8.1)	24 (9.7)	144 (17.3)	263 (25.4)
Strongly agree	115 (3.8)	12 (4.9)	42 (5.1)	88 (8.5)
Don't know	107 (3.5)	9 (3.6)	21 (2.5)	8 (0.8)

New screen

Below are some claims that some people have made about monkeypox. For each one, please say whether you strongly disagree, disagree, neither agree nor disagree, agree, or strongly agree.

ASK ALL

Q7. How much do you agree or disagree with the following claims:

SINGLE CODE

RANDOMISE statements

The risks of monkeypox are being exaggerated

	General population	Savanta GBMSM	Grindr	Meta
Strongly disagree	170 (5.6)	23 (9.3)	99 (11.9)	175 (16.9)
Disagree	822 (27.0)	87 (35.2)	326 (39.2)	505 (48.7)
Neither agree nor disagree	998 (32.7)	65 (26.3)	221 (26.6)	214 (20.7)
Agree	529 (17.3)	39 (15.8)	89 (10.7)	89 (8.6)
Strongly agree	184 (6.0)	11 (4.5)	34 (4.1)	19 (1.8)
Don't know	347 (11.4)	22 (8.9)	62 (7.5)	34 (3.3)

For peer review only - http://bmjopen.bmj.com/site/about/guidelines.xhtml

Because of the monkeypox outbreak, it is best to avoid physical contact with (touch) men who are gay, bisexual or have sex with men

	General population	Savanta GBMSM	Grindr	Meta
Strongly disagree	422 (13.8)	50 (20.2)	237 (28.5)	294 (28.4)
Disagree	707 (23.2)	64 (25.9)	267 (32.1)	357 (34.5)
Neither agree nor disagree	667 (21.9)	44 (17.8)	147 (17.7)	172 (16.6)
Agree	611 (20.0)	51 (20.6)	132 (15.9)	161 (15.5)
Strongly agree	305 (10.0)	22 (8.9)	22 (2.6)	37 (3.6)
Don't know	338 (11.1)	16 (6.5)	26 (3.1)	15 (1.4)

Because of the monkeypox outbreak, it is best to avoid physical contact with (touch) people from Africa

	General population	Savanta GBMSM	Grindr	Meta
Strongly disagree	462 (15.1)	52 (21.1)	327 (39.4)	431 (41.6)
Disagree	845 (27.7)	69 (27.9)	273 (32.9)	399 (38.5)
Neither agree nor disagree	768 (25.2)	57 (23.1)	118 (14.2)	127 (12.3)
Agree	454 (14.9)	40 (16.2)	59 (7.1)	43 (4.2)
Strongly agree	175 (5.7)	10 (4.0)	14 (1.7)	11 (1.1)
Don't know	346 (11.3)	19 (7.7)	40 (4.8)	25 (2.4)

Monkeypox is only a risk to men who are gay, bisexual or have sex with men

	General population	Savanta GBMSM	Grindr	Meta
Strongly disagree	536 (17.6)	77 (31.2)	375 (45.1)	431 (41.6)
Disagree	909 (29.8)	75 (30.4)	302 (36.3)	449 (43.3)
Neither agree nor disagree	586 (19.2)	35 (14.2)	56 (6.7)	63 (6.1)
Agree	521 (17.1)	30 (12.1)	61 (7.3)	65 (6.3)
Strongly agree	161 (5.3)	15 (6.1)	14 (1.7)	21 (2.0)
Don't know	337 (11.0)	15 (6.1)	23 (2.8)	7 (0.7)

New screen

ASK ALL

Q8. What do you think the main symptoms of monkeypox are?

Please select up to 4

MULTICODE – MAX 4

RANDOMISE

	General population	Savanta GBMSM	Grindr	Meta
High temperature / fever	1081 (35.4)	105 (42.5)	445 (53.5)	632 (61.0)

For peer review only - http://bmjopen.bmj.com/site/about/guidelines.xhtml

Shivering / chills	501 (16.4)	35 (14.2)	151 (18.2)	232 (22.4)
Muscle aches	542 (17.8)	47 (19.0)	249 (30.0)	339 (32.7)
Pain in your arms, legs or joints	385 (12.6)	45 (18.2)	117 (14.1)	170 (16.4)
Backache	104 (3.4)	10 (4.0)	19 (2.3)	23 (2.2)
Headache	426 (14.0)	39 (15.8)	146 (17.6)	181 (17.5)
Exhaustion	320 (10.5)	37 (15)	169 (20.3)	226 (21.8)
Swollen glands	496 (16.3)	68 (27.5)	256 (30.8)	446 (43.1)
Unexplained rash with blisters	1688 (55.3)	147 (59.5)	647 (77.9)	872 (84.2)
Unexplained rash without blisters	632 (20.7)	65 (26.3)	198 (23.8)	257 (24.8)
Runny or blocked nose	127 (4.2)	5 (2.0)	16 (1.9)	10 (1.0)
Dizziness	160 (5.2)	12 (4.9)	23 (2.8)	18 (1.7)
Sore throat	281 (9.2)	30 (12.1)	72 (8.7)	88 (8.5)
Nausea / feeling sick, or vomiting	388 (12.7)	46 (18.6)	68 (8.2)	77 (7.4)
Diarrhoea	182 (6)	17 (6.9)	31 (3.7)	32 (3.1)
Other, please state [ANCHOR]	5 (0.2)	0 (0.0)	9 (1.1)	12 (1.2)
Don't know [ANCHOR, EXCLUSIVE]	562 (18.4)	23 (9.3)	48 (5.8)	25 (2.4)

†Participants could select up to four answers to this question, therefore column totals add to more than 100%.

New screen

ASK ALL

Q9. You can catch monkeypox if:

Please select one option for each answer

RANDOMISE statements

You touch the rash of a person who has monkeypox

	General population	Savanta GBMSM	Grindr	Meta
Strongly disagree	68 (2.2)	3 (1.2)	11 (1.3)	6 (0.6)
Disagree	176 (5.8)	10 (4.0)	23 (2.8)	15 (1.4)
Neither agree nor disagree	516 (16.9)	28 (11.3)	48 (5.8)	38 (3.7)
Agree	1326 (43.5)	112 (45.3)	394 (47.4)	424 (40.9)
Strongly agree	394 (12.9)	65 (26.3)	293 (35.3)	519 (50.1)
Don't know	570 (18.7)	29 (11.7)	62 (7.5)	34 (3.3)

You are coughed or sneezed on by a person who has monkeypox

	General	Savanta GBMSM	Grindr	Meta

For peer review only - http://bmjopen.bmj.com/site/about/guidelines.xhtml

	population			
Strongly disagree	82 (2.7)	17 (6.9)	50 (6.0)	82 (7.9)
Disagree	356 (11.7)	39 (15.8)	191 (23.0)	253 (24.4)
Neither agree nor disagree	634 (20.8)	41 (16.6)	127 (15.3)	160 (15.4)
Agree	1024 (33.6)	87 (35.2)	251 (30.2)	344 (33.2)
Strongly agree	273 (9.0)	22 (8.9)	74 (8.9)	82 (7.9)
Don't know	681 (22.3)	41 (16.6)	138 (16.6)	115 (11.1)

You touch a person who has monkeypox, even if they do not have a rash

	General population	Savanta GBMSM	Grindr	Meta
Strongly disagree	91 (3.0)	4 (1.6)	26 (3.1)	20 (1.9)
Disagree	419 (13.7)	28 (11.3)	139 (16.7)	189 (18.2)
Neither agree nor disagree	634 (20.8)	61 (24.7)	125 (15.0)	198 (19.1)
Agree	932 (30.6)	91 (36.8)	337 (40.6)	410 (39.6)
Strongly agree	237 (7.8)	24 (9.7)	73 (8.8)	95 (9.2)
Don't know	737 (24.2)	39 (15.8)	131 (15.8)	124 (12)

You have sex with someone who has monkeypox

	General population	Savanta GBMSM	Grindr	Meta
Strongly disagree	72 (2.4)	5 (2.0)	12 (1.4)	10 (1.0)
Disagree	99 (3.2)	4 (1.6)	5 (0.6)	7 (0.7)
Neither agree nor disagree	359 (11.8)	17 (6.9)	24 (2.9)	16 (1.5)
Agree	1293 (42.4)	120 (48.6)	387 (46.6)	441 (42.6)
Strongly agree	802 (26.3)	89 (36.0)	377 (45.4)	551 (53.2)
Don't know	425 (13.9)	12 (4.9)	26 (3.1)	11 (1.1)

You come within 1 metre of someone who has monkeypox

	General population	Savanta GBMSM	Grindr	Meta
Strongly disagree	357 (11.7)	59 (23.9)	269 (32.4)	407 (39.3)
Disagree	972 (31.9)	93 (37.7)	379 (45.6)	438 (42.3)
Neither agree nor disagree	598 (19.6)	29 (11.7)	72 (8.7)	84 (8.1)
Agree	358 (11.7)	34 (13.8)	39 (4.7)	50 (4.8)
Strongly agree	131 (4.3)	4 (1.6)	10 (1.2)	11 (1.1)
Don't know	634 (20.8)	28 (11.3)	62 (7.5)	46 (4.4)

You touch something (e.g. towels, bedding or clothing) that has been touched by a person who has monkeypox

	General population	Savanta GBMSM	Grindr	Meta
Strongly disagree	87 (2.9)	7 (2.8)	21 (2.5)	18 (1.7)

For peer review only - http://bmjopen.bmj.com/site/about/guidelines.xhtml

Disagree	414 (13.6)	30 (12.1)	85 (10.2)	72 (6.9)
Neither agree nor disagree	653 (21.4)	46 (18.6)	100 (12.0)	89 (8.6)
Agree	961 (31.5)	96 (38.9)	379 (45.6)	585 (56.5)
Strongly agree	248 (8.1)	35 (14.2)	142 (17.1)	204 (19.7)
Don't know	687 (22.5)	33 (13.4)	104 (12.5)	68 (6.6)

You touch a pet animal that has monkeypox

	General population	Savanta GBMSM	Grindr	Meta
Strongly disagree	130 (4.3)	14 (5.7)	61 (7.3)	67 (6.5)
Disagree	469 (15.4)	43 (17.4)	140 (16.8)	151 (14.6)
Neither agree nor disagree	693 (22.7)	57 (23.1)	159 (19.1)	208 (20.1)
Agree	740 (24.3)	62 (25.1)	219 (26.4)	341 (32.9)
Strongly agree	193 (6.3)	24 (9.7)	46 (5.5)	82 (7.9)
Don't know	825 (27.0)	47 (19)	206 (24.8)	187 (18.1)

Intended behaviour

Motivational messaging

New screen

[For Savanta gen pop sample, randomise people to four groups. Ppts should see EITHER group A OR group B OR group C OR group D.
For Savanta GBMSM, Grindr, Meta samples, randomise people to two groups. Ppts should see EITHER group E OR group F.]

[Savanta gen pop] GROUP A (risk + perceived necessity/efficacy): Monkeypox causes mild illness for most people, but it can be very severe and can even kill some people. Pets may also be able to catch monkeypox. People who have recently been in contact with someone who might have monkeypox and get an unexplained rash should get tested quickly. If they test positive, they should avoid close contact with others to stop monkeypox from spreading further.

[Savanta gen pop] GROUP B (risk + perceived benefits): Monkeypox causes mild illness for most people, but it can be very severe and can even kill some people. Pets may also be able to catch monkeypox. People who test positive for monkeypox can take action to protect their friends, family, and pets from infection.

[Savanta gen pop] GROUP C (risk + perceived costs): Monkeypox causes mild illness for most people, but it can be very severe and can even kill some people. Pets may also be able to catch monkeypox. Imagine that people who have to self-isolate because they test positive for monkeypox will be provided with the practical and financial support that they need to do that.

For peer review only - http://bmjopen.bmj.com/site/about/guidelines.xhtml

[Savanta gen pop] GROUP D (control message): Monkeypox is a rare infection that is mainly found in parts of west or central Africa. Recently, there has been an outbreak of monkeypox in other countries. Since early May, more than 40,000 monkeypox cases have been reported in over 80 countries where the virus is not normally seen, including the UK.

[Savanta GBMSM / Grindr / Meta] GROUP E (all messages): Monkeypox causes mild illness for most people, but it can be very severe and can even kill some people. Pets may also be able to catch monkeypox.

People who have recently been in contact with someone who might have monkeypox and get an unexplained rash should get tested quickly. If they test positive, they should avoid close contact with others to stop monkeypox from spreading further. People who test positive for monkeypox can take action to protect their friends, family, and pets from infection. Imagine that people who have to self-isolate because they test positive for monkeypox will be provided with the practical and financial support that they need to do that.

[Savanta GBMSM / Grindr / Meta] GROUP F (control message): Monkeypox is a rare infection that is mainly found in parts of west or central Africa. Recently, there has been an outbreak of monkeypox in other countries.

There is a new outbreak of monkeypox in the UK. Since early May, more than 40,000 monkeypox cases have been reported in over 80 countries where the virus is not normally seen, including the UK. The current outbreak is the first time that the virus has been passed from person to person in the UK, and so the UK Health Security Agency (UKHSA) is working with partners to put in place guidance and measures that will prevent the spread of infection.

New screen

Behaviours

Self-isolation

People who have monkeypox are being asked to self-isolate for 21 days (3 weeks). They are also being asked to tell public health officials about other people they have been in contact with.

Self-isolation means staying at home and not going to work, school or public areas. It means avoiding close contact with people you live with, spending time in separate rooms as much as possible, and not having visitors to your home.

[Randomise order of Q10 and Q11]

New screen

ASK ALL
Q10. Imagine you are contacted by public health officials after testing and told that **you have monkeypox**. They tell you that you need to self-isolate for 21 days.

For peer review only - http://bmjopen.bmj.com/site/about/guidelines.xhtml

Realistically, would you self-isolate for 21 days?
SINGLE CODE
Answer Options

	General population	Savanta GBMSM	Grindr	Meta
Definitely would not	106 (3.5)	10 (4.0)	56 (6.7)	41 (4.0)
Probably would not	283 (9.3)	24 (9.7)	116 (14.0)	144 (13.9)
Not sure	367 (12.0)	19 (7.7)	74 (8.9)	90 (8.7)
Probably would	799 (26.2)	76 (30.8)	260 (31.3)	327 (31.6)
Definitely would	1495 (49.0)	118 (47.8)	325 (39.1)	434 (41.9)

New screen

ASK ALL
Q11. Imagine you are contacted by public health officials and told that **you have come into high-risk contact with someone who has monkeypox**. They tell you that you need to self-isolate for 21 days.

Realistically, would you self-isolate for 21 days?
SINGLE CODE
Answer Options

	General population	Savanta GBMSM	Grindr	Meta
Definitely would not	126 (4.1)	15 (6.1)	78 (9.4)	70 (6.8)
Probably would not	341 (11.2)	38 (15.4)	169 (20.3)	249 (24.0)
Not sure	483 (15.8)	24 (9.7)	124 (14.9)	172 (16.6)
Probably would	907 (29.7)	77 (31.2)	261 (31.4)	322 (31.1)
Definitely would	1193 (39.1)	93 (37.7)	199 (23.9)	223 (21.5)

New screen

ASK ALL
Q12. What would stop you from being able to self-isolate for 21 days?

Please select all that apply
MULTI CODE, RANDOMISE ORDER
Answer Options

	General population	Savanta GBMSM	Grindr	Meta
I would need to go out to get food, medicines, or other essentials	992 (32.5)	119 (48.2)	477 (57.4)	604 (58.3)
I would need to go out to work	542 (17.8)	52 (21.1)	309 (37.2)	323 (31.2)
I would need to go out to provide help or care to friends or relatives	238 (7.8)	22 (8.9)	53 (6.4)	72 (6.9)

For peer review only - http://bmjopen.bmj.com/site/about/guidelines.xhtml

I would need to go out as part of looking after my children (e.g. to take them to school, or a playground)	396 (13.0)	9 (3.6)	21 (2.5)	15 (1.4)
I would need to go out for a walk or some other exercise	765 (25.1)	72 (29.1)	388 (46.7)	550 (53.1)
My home is too small	192 (6.3)	17 (6.9)	103 (12.4)	109 (10.5)
I would be too bored or lonely	344 (11.3)	39 (15.8)	170 (20.5)	242 (23.4)
I don't think it's necessary for me to self-isolate	207 (6.8)	12 (4.9)	54 (6.5)	85 (8.2)
Other, please state	40 (1.3)	3 (1.2)	40 (4.8)	52 (5.0)
Nothing, I would stay at home for 21 days [EXCLUSIVE]	1166 (38.2)	76 (30.8)	153 (18.4)	185 (17.9)

†Participants could select more than one item for this question, therefore column totals add to more than 100%.

New screen

Now we would like you to think about a different scenario.

Help seeking

New screen

Imagine that tomorrow morning **you develop an unexplained rash with blisters**. You also learn that **you have come into contact with someone who might have monkeypox**.

ASK ALL
Q13 If **you** got these symptoms, which of these things would you do?

RANDOMISE order

Wait a day or two to see if they get better or clear up on their own

	General population	Savanta GBMSM	Grindr	Meta
Definitely would not	344 (11.3)	27 (10.9)	130 (15.6)	195 (18.8)
Probably would not	546 (17.9)	47 (19.0)	175 (21.1)	248 (23.9)
Not sure	809 (26.5)	58 (23.5)	145 (17.4)	217 (20.9)
Probably would	1080 (35.4)	92 (37.2)	323 (38.9)	326 (31.5)

For peer review only - http://bmjopen.bmj.com/site/about/guidelines.xhtml

Definitely would	271 (8.9)	23 (9.3)	58 (7.0)	50 (4.8)

Try to book an appointment with a GP

	General population	Savanta GBMSM	Grindr	Meta
Definitely would not	307 (10.1)	28 (11.3)	154 (18.5)	183 (17.7)
Probably would not	409 (13.4)	48 (19.4)	167 (20.1)	295 (28.5)
Not sure	586 (19.2)	40 (16.2)	125 (15.0)	148 (14.3)
Probably would	1032 (33.8)	86 (34.8)	221 (26.6)	222 (21.4)
Definitely would	716 (23.5)	45 (18.2)	164 (19.7)	188 (18.1)

Visit a Pharmacist/Chemist for advice

	General population	Savanta GBMSM	Grindr	Meta
Definitely would not	634 (20.8)	49 (19.8)	284 (34.2)	393 (37.9)
Probably would not	749 (24.6)	69 (27.9)	329 (39.6)	438 (42.3)
Not sure	727 (23.8)	64 (25.9)	97 (11.7)	125 (12.1)
Probably would	701 (23.0)	48 (19.4)	88 (10.6)	63 (6.1)
Definitely would	239 (7.8)	17 (6.9)	33 (4.0)	17 (1.6)

Go to A&E or visit another NHS service such as a walk-in centre or minor injuries unit

	General population	Savanta GBMSM	Grindr	Meta
Definitely would not	776 (25.4)	60 (24.3)	309 (37.2)	436 (42.1)
Probably would not	841 (27.6)	66 (26.7)	274 (33.0)	365 (35.2)
Not sure	682 (22.4)	54 (21.9)	110 (13.2)	113 (10.9)
Probably would	519 (17.0)	52 (21.1)	93 (11.2)	87 (8.4)
Definitely would	232 (7.6)	15 (6.1)	45 (5.4)	35 (3.4)

Call NHS 111 or 999 / ambulance service

	General population	Savanta GBMSM	Grindr	Meta
Definitely would not	246 (8.1)	22 (8.9)	145 (17.4)	185 (17.9)
Probably would not	358 (11.7)	30 (12.1)	153 (18.4)	233 (22.5)
Not sure	556 (18.2)	36 (14.6)	108 (13.0)	118 (11.4)
Probably would	1084 (35.5)	95 (38.5)	243 (29.2)	283 (27.3)
Definitely would	806 (26.4)	64 (25.9)	182 (21.9)	217 (20.9)

Visit a walk-in sexual health service

	General population	Savanta GBMSM	Grindr	Meta
Definitely would not	906 (29.7)	50 (20.2)	164 (19.7)	207 (20.0)

For peer review only - http://bmjopen.bmj.com/site/about/guidelines.xhtml

Probably would not	783 (25.7)	56 (22.7)	192 (23.1)	203 (19.6)
Not sure	727 (23.8)	51 (20.6)	125 (15.0)	130 (12.5)
Probably would	454 (14.9)	65 (26.3)	205 (24.7)	262 (25.3)
Definitely would	180 (5.9)	25 (10.1)	145 (17.4)	234 (22.6)

Call a sexual health service [fix below 6]

	General population	Savanta GBMSM	Grindr	Meta
Definitely would not	692 (22.7)	27 (10.9)	33 (4.0)	25 (2.4)
Probably would not	690 (22.6)	38 (15.4)	68 (8.2)	57 (5.5)
Not sure	697 (22.9)	35 (14.2)	60 (7.2)	60 (5.8)
Probably would	630 (20.7)	87 (35.2)	278 (33.5)	320 (30.9)
Definitely would	341 (11.2)	60 (24.3)	392 (47.2)	574 (55.4)

Let people you have recently been in close contact with know that you have symptoms

	General population	Savanta GBMSM	Grindr	Meta
Definitely would not	81 (2.7)	8 (3.2)	34 (4.1)	32 (3.1)
Probably would not	220 (7.2)	20 (8.1)	75 (9.0)	90 (8.7)
Not sure	495 (16.2)	47 (19.0)	152 (18.3)	177 (17.1)
Probably would	1185 (38.9)	96 (38.9)	298 (35.9)	396 (38.2)
Definitely would	1069 (35)	76 (30.8)	272 (32.7)	341 (32.9)

Search for information from official websites (e.g. NHS, GOV.UK)

	General population	Savanta GBMSM	Grindr	Meta
Definitely would not	73 (2.4)	4 (1.6)	13 (1.6)	9 (0.9)
Probably would not	122 (4.0)	10 (4.0)	26 (3.1)	23 (2.2)
Not sure	325 (10.7)	22 (8.9)	30 (3.6)	24 (2.3)
Probably would	1165 (38.2)	91 (36.8)	281 (33.8)	366 (35.3)
Definitely would	1365 (44.8)	120 (48.6)	481 (57.9)	614 (59.3)

Search for information from other places, including blogs, social media sites (e.g. Facebook, Twitter, Instagram), online communities or other websites [fix below 9]

	General population	Savanta GBMSM	Grindr	Meta
Definitely would not	533 (17.5)	49 (19.8)	144 (17.3)	135 (13.0)
Probably would not	647 (21.2)	54 (21.9)	231 (27.8)	277 (26.7)
Not sure	647 (21.2)	39 (15.8)	113 (13.6)	155 (15.0)
Probably would	789 (25.9)	70 (28.3)	206 (24.8)	301 (29.1)
Definitely would	434 (14.2)	35 (14.2)	137 (16.5)	168 (16.2)

For peer review only - http://bmjopen.bmj.com/site/about/guidelines.xhtml

New screen

Imagine that tomorrow morning **you develop an unexplained rash with blisters**. You also learn that **you have come into contact with someone who might have monkeypox**.

Q13_other. Please state any other things you would do.
Open ended text
Nothing button

Contact behaviour

New screen

Imagine that tomorrow morning **you develop an unexplained rash with blisters**. You also learn that **you have come into contact with someone who might have monkeypox**.

ASK ALL
Q14 If **you** got these symptoms, in the following 21 days, realistically how much, if at all, would you:

RANDOMISE order

Come into skin-to-skin contact with other people

	General population	Savanta GBMSM	Grindr	Meta
I would completely stop doing this	1883 (61.7)	153 (61.9)	648 (78)	858 (82.8)
Less than normal	412 (13.5)	41 (16.6)	127 (15.3)	135 (13.0)
Same as normal	249 (8.2)	21 (8.5)	15 (1.8)	12 (1.2)
More than normal	107 (3.5)	8 (3.2)	3 (0.4)	1 (0.1)
Not applicable, I wouldn't do this anyway	280 (9.2)	16 (6.5)	22 (2.6)	21 (2.0)
Don't know	109 (3.6)	7 (2.8)	15 (1.8)	9 (0.9)
Prefer not to say	10 (0.3)	1 (0.4)	1 (0.1)	0 (0.0)

Have sexual contact (from kissing to intercourse) with other people

	General population	Savanta GBMSM	Grindr	Meta
I would completely stop doing this	1923 (63.0)	171 (69.2)	721 (86.8)	922 (89.0)
Less than normal	238 (7.8)	20 (8.1)	54 (6.5)	61 (5.9)
Same as normal	216 (7.1)	15 (6.1)	14 (1.7)	7 (0.7)
More than normal	113 (3.7)	10 (4.0)	1 (0.1)	1 (0.1)
Not applicable, I wouldn't do this anyway	451 (14.8)	24 (9.7)	27 (3.2)	30 (2.9)
Don't know	93 (3.0)	6 (2.4)	13 (1.6)	13 (1.3)
Prefer not to say	16 (0.5)	1 (0.4)	1 (0.1)	2 (0.2)

For peer review only - http://bmjopen.bmj.com/site/about/guidelines.xhtml

Have sex without using a condom

	General population	Savanta GBMSM	Grindr	Meta
I would completely stop doing this	1729 (56.7)	155 (62.8)	623 (75)	788 (76.1)
Less than normal	146 (4.8)	17 (6.9)	33 (4.0)	37 (3.6)
Same as normal	235 (7.7)	15 (6.1)	27 (3.2)	29 (2.8)
More than normal	124 (4.1)	11 (4.5)	3 (0.4)	2 (0.2)
Not applicable, I wouldn't do this anyway	680 (22.3)	42 (17.0)	123 (14.8)	161 (15.5)
Don't know	111 (3.6)	7 (2.8)	17 (2.0)	14 (1.4)
Prefer not to say	25 (0.8)	0 (0.0)	5 (0.6)	5 (0.5)

Share bedding, towels, or clothes with other people

	General population	Savanta GBMSM	Grindr	Meta
I would completely stop doing this	1839 (60.3)	147 (59.5)	604 (72.7)	807 (77.9)
Less than normal	286 (9.4)	23 (9.3)	62 (7.5)	96 (9.3)
Same as normal	279 (9.1)	21 (8.5)	24 (2.9)	19 (1.8)
More than normal	124 (4.1)	12 (4.9)	2 (0.2)	1 (0.1)
Not applicable, I wouldn't do this anyway	415 (13.6)	41 (16.6)	126 (15.2)	104 (10.0)
Don't know	98 (3.2)	3 (1.2)	12 (1.4)	8 (0.8)
Prefer not to say	9 (0.3)	0 (0.0)	1 (0.1)	1 (0.1)

Go to a crowded place

	General population	Savanta GBMSM	Grindr	Meta
I would completely stop doing this	1872 (61.4)	141 (57.1)	552 (66.4)	663 (64.0)
Less than normal	418 (13.7)	50 (20.2)	183 (22)	297 (28.7)
Same as normal	248 (8.1)	23 (9.3)	57 (6.9)	39 (3.8)
More than normal	123 (4.0)	9 (3.6)	1 (0.1)	1 (0.1)
Not applicable, I wouldn't do this anyway	275 (9.0)	16 (6.5)	25 (3.0)	23 (2.2)
Don't know	103 (3.4)	8 (3.2)	13 (1.6)	12 (1.2)
Prefer not to say	11 (0.4)	0 (0.0)	0 (0.0)	1 (0.1)

Help or provide care for a vulnerable person

	General population	Savanta GBMSM	Grindr	Meta
I would completely stop doing this	1674 (54.9)	131 (53.0)	516 (62.1)	686 (66.2)
Less than normal	304 (10.0)	16 (6.5)	81 (9.7)	81 (7.8)
Same as normal	255 (8.4)	24 (9.7)	23 (2.8)	24 (2.3)
More than normal	160 (5.2)	14 (5.7)	8 (1.0)	6 (0.6)
Not applicable, I wouldn't do this anyway	513 (16.8)	50 (20.2)	181 (21.8)	217 (20.9)

For peer review only - http://bmjopen.bmj.com/site/about/guidelines.xhtml

Don't know	134 (4.4)	11 (4.5)	22 (2.6)	20 (1.9)
Prefer not to say	10 (0.3)	1 (0.4)	0 (0.0)	2 (0.2)

Go to a public place where you may come into physical contact with (touch) someone else

	General population	Savanta GBMSM	Grindr	Meta
I would completely stop doing this	1818 (59.6)	146 (59.1)	525 (63.2)	653 (63.0)
Less than normal	469 (15.4)	51 (20.6)	236 (28.4)	318 (30.7)
Same as normal	249 (8.2)	20 (8.1)	33 (4.0)	24 (2.3)
More than normal	143 (4.7)	9 (3.6)	2 (0.2)	2 (0.2)
Not applicable, I wouldn't do this anyway	259 (8.5)	11 (4.5)	24 (2.9)	24 (2.3)
Don't know	104 (3.4)	9 (3.6)	10 (1.2)	14 (1.4)
Prefer not to say	8 (0.3)	1 (0.4)	1 (0.1)	1 (0.1)

New screen

Imagine that tomorrow morning **you develop an unexplained rash with blisters**. You also learn that **you have come into contact with someone who might have monkeypox**.

Q15 How many days after the start of these symptoms would you:

Please give an approximate number if you are unsure. If you would not do this, please put 0.
Type your answer below

			General population	Savanta GBMSM	Grindr	Meta
Have sexual contact (from kissing to intercourse) with someone else	OPEN TEXT ENTRY – number [cap at 100]	ASK ONLY to those who answer 1-4 for similar question above	N=2021, M=15.7, SD=15.3, range 0 to 100.	N=190, M=16.2, SD=14.4, range 0 to 100.	N=746, M=20.4, SD=15.8, range 0 to 100.	N=947, M=20.0, SD=12.3, range 0 to 100.
Prefer not to say			469 (18.8)	26 (12.0)	44 (5.6)	44 (4.4)
Have sex without using a condom	OPEN TEXT ENTRY – number [cap at 100]	ASK ONLY to those who answer 1-4 for similar question above	N=1807, M=15.7, SD=16.8, range 0 to 100.	N=175, M=16.8, SD=18.9, range 0 to 100.	N=649, M=21.2, SD=19.5, range 0 to 100.	N=821, M=21.3, SD=18.0, range 0 to 100.
Prefer not to say			427 (19.1)	23 (11.6)	37 (5.4)	35 (4.1)

Contact sharing

For peer review only - http://bmjopen.bmj.com/site/about/guidelines.xhtml

New screen

People with suspected or confirmed monkeypox are being asked by public health officials to share contact details of people they have been in close contact with, so that they can be offered appropriate care and advice. Details of contacts are kept completely confidential.

ASK ALL

Q16 If **you** were asked for this information, would you try to share all the contact details of every …

RANDOMISE statements

Person who had been in your home (household members and visitors) in the last seven days

	General population	Savanta GBMSM	Grindr	Meta
Definitely would not	135 (4.4)	14 (5.7)	51 (6.1)	56 (5.4)
Probably would not	168 (5.5)	19 (7.7)	101 (12.2)	118 (11.4)
Not sure	392 (12.9)	30 (12.1)	97 (11.7)	120 (11.6)
Probably would	758 (24.9)	60 (24.3)	247 (29.7)	325 (31.4)
Definitely would	1552 (50.9)	120 (48.6)	331 (39.8)	414 (40.0)
Prefer not to say	45 (1.5)	4 (1.6)	4 (0.5)	3 (0.3)

Person you had sexual contact with (from kissing to intercourse) in the last seven days

	General population	Savanta GBMSM	Grindr	Meta
Definitely would not	169 (5.5)	17 (6.9)	49 (5.9)	54 (5.2)
Probably would not	162 (5.3)	16 (6.5)	59 (7.1)	67 (6.5)
Not sure	366 (12.0)	27 (10.9)	59 (7.1)	83 (8.0)
Probably would	629 (20.6)	56 (22.7)	236 (28.4)	282 (27.2)
Definitely would	1671 (54.8)	127 (51.4)	420 (50.5)	545 (52.6)
Prefer not to say	53 (1.7)	4 (1.6)	8 (1.0)	5 (0.5)

Place you had sexual contact with someone (from kissing to intercourse) in the last seven days

	General population	Savanta GBMSM	Grindr	Meta
Definitely would not	165 (5.4)	17 (6.9)	51 (6.1)	52 (5)
Probably would not	188 (6.2)	18 (7.3)	63 (7.6)	72 (6.9)
Not sure	415 (13.6)	28 (11.3)	72 (8.7)	91 (8.8)
Probably would	655 (21.5)	64 (25.9)	261 (31.4)	306 (29.5)
Definitely would	1559 (51.1)	116 (47)	375 (45.1)	510 (49.2)
Prefer not to say	68 (2.2)	4 (1.6)	9 (1.1)	5 (0.5)

Person you had skin-to-skin contact with (including hugging or kissing) in the last seven days

	General	Savanta	Grindr	Meta

For peer review only - http://bmjopen.bmj.com/site/about/guidelines.xhtml

	population	GBMSM		
Definitely would not	140 (4.6)	15 (6.1)	45 (5.4)	53 (5.1)
Probably would not	183 (6.0)	16 (6.5)	74 (8.9)	83 (8.0)
Not sure	352 (11.5)	25 (10.1)	74 (8.9)	116 (11.2)
Probably would	710 (23.3)	65 (26.3)	261 (31.4)	309 (29.8)
Definitely would	1621 (53.1)	122 (49.4)	373 (44.9)	472 (45.6)
Prefer not to say	44 (1.4)	4 (1.6)	4 (0.5)	3 (0.3)

Person you had shared bedding, towels, or clothes with in the last seven days

	General population	Savanta GBMSM	Grindr	Meta
Definitely would not	148 (4.9)	15 (6.1)	48 (5.8)	47 (4.5)
Probably would not	180 (5.9)	16 (6.5)	57 (6.9)	70 (6.8)
Not sure	352 (11.5)	26 (10.5)	69 (8.3)	81 (7.8)
Probably would	746 (24.5)	65 (26.3)	236 (28.4)	282 (27.2)
Definitely would	1574 (51.6)	122 (49.4)	415 (49.9)	551 (53.2)
Prefer not to say	50 (1.6)	3 (1.2)	6 (0.7)	5 (0.5)

Attitudes and beliefs

New screen

ASK ALL

Q17 How much do you agree or disagree with the following statements:

RANDOMISE statements

I wouldn't want to know the results of a monkeypox test

	General population	Savanta GBMSM	Grindr	Meta
Strongly disagree	1057 (34.7)	110 (44.5)	498 (59.9)	690 (66.6)
Disagree	963 (31.6)	63 (25.5)	235 (28.3)	243 (23.5)
Neither agree nor disagree	365 (12.0)	23 (9.3)	29 (3.5)	27 (2.6)
Agree	353 (11.6)	23 (9.3)	27 (3.2)	22 (2.1)
Strongly agree	240 (7.9)	23 (9.3)	35 (4.2)	51 (4.9)
Don't know	72 (2.4)	5 (2)	7 (0.8)	3 (0.3)

I would be worried what my friends or family would think about me if they thought I had monkeypox

	General population	Savanta GBMSM	Grindr	Meta
Strongly disagree	428 (14.0)	35 (14.2)	83 (10.0)	125 (12.1)
Disagree	624 (20.5)	43 (17.4)	114 (13.7)	147 (14.2)
Neither agree nor disagree	627 (20.6)	42 (17.0)	99 (11.9)	104 (10.0)

For peer review only - http://bmjopen.bmj.com/site/about/guidelines.xhtml

Agree	865 (28.4)	73 (29.6)	271 (32.6)	325 (31.4)
Strongly agree	402 (13.2)	52 (21.1)	263 (31.6)	333 (32.1)
Don't know	104 (3.4)	2 (0.8)	1 (0.1)	2 (0.2)

I would be worried about how colleagues / my employer would react if they thought I had monkeypox

	General population	Savanta GBMSM	Grindr	Meta
Strongly disagree	366 (12)	31 (12.6)	79 (9.5)	99 (9.6)
Disagree	508 (16.7)	40 (16.2)	95 (11.4)	112 (10.8)
Neither agree nor disagree	649 (21.3)	37 (15.0)	82 (9.9)	109 (10.5)
Agree	914 (30.0)	67 (27.1)	249 (30.0)	309 (29.8)
Strongly agree	462 (15.1)	64 (25.9)	316 (38.0)	391 (37.7)
Don't know	151 (5.0)	8 (3.2)	10 (1.2)	16 (1.5)

I don't want to have a monkeypox test result on my medical record

	General population	Savanta GBMSM	Grindr	Meta
Strongly disagree	541 (17.7)	50 (20.2)	161 (19.4)	229 (22.1)
Disagree	801 (26.3)	63 (25.5)	193 (23.2)	260 (25.1)
Neither agree nor disagree	837 (27.4)	58 (23.5)	183 (22.0)	205 (19.8)
Agree	494 (16.2)	42 (17.0)	142 (17.1)	151 (14.6)
Strongly agree	257 (8.4)	28 (11.3)	142 (17.1)	171 (16.5)
Don't know	120 (3.9)	6 (2.4)	10 (1.2)	20 (1.9)

An effective way to prevent the spread of monkeypox is for people who have symptoms to contact healthcare services

	General population	Savanta GBMSM	Grindr	Meta
Strongly disagree	67 (2.2)	7 (2.8)	16 (1.9)	19 (1.8)
Disagree	131 (4.3)	9 (3.6)	25 (3.0)	19 (1.8)
Neither agree nor disagree	459 (15.0)	24 (9.7)	58 (7.0)	64 (6.2)
Agree	1185 (38.9)	88 (35.6)	311 (37.4)	378 (36.5)
Strongly agree	1105 (36.2)	113 (45.7)	408 (49.1)	549 (53)
Don't know	103 (3.4)	6 (2.4)	13 (1.6)	7 (0.7)

I would be willing to contact a sexual health clinic if I thought I had monkeypox symptoms or had come into contact with someone who had monkeypox

	General population	Savanta GBMSM	Grindr	Meta
Strongly disagree	149 (4.9)	7 (2.8)	21 (2.5)	20 (1.9)
Disagree	291 (9.5)	20 (8.1)	24 (2.9)	17 (1.6)
Neither agree nor disagree	590 (19.3)	28 (11.3)	36 (4.3)	39 (3.8)
Agree	1047 (34.3)	73 (29.6)	260 (31.3)	247 (23.8)

For peer review only - http://bmjopen.bmj.com/site/about/guidelines.xhtml

Strongly agree	800 (26.2)	110 (44.5)	481 (57.9)	709 (68.4)
Don't know	173 (5.7)	9 (3.6)	9 (1.1)	4 (0.4)

New screen

ASK ALL
Q18 How much do you agree or disagree with the following statements:

RANDOMISE statements

If I had monkeypox symptoms, I wouldn't want to tell anyone as I don't want to self-isolate

	General population	Savanta GBMSM	Grindr	Meta
Strongly disagree	1055 (34.6)	79 (32.0)	291 (35.0)	374 (36.1)
Disagree	949 (31.1)	74 (30.0)	322 (38.7)	414 (40.0)
Neither agree nor disagree	431 (14.1)	33 (13.4)	98 (11.8)	119 (11.5)
Agree	365 (12.0)	32 (13.0)	72 (8.7)	78 (7.5)
Strongly agree	174 (5.7)	21 (8.5)	36 (4.3)	35 (3.4)
Don't know	76 (2.5)	8 (3.2)	12 (1.4)	16 (1.5)

If I had monkeypox symptoms, I wouldn't want to tell anyone so that others don't have to self-isolate

	General population	Savanta GBMSM	Grindr	Meta
Strongly disagree	909 (29.8)	71 (28.7)	260 (31.3)	305 (29.4)
Disagree	991 (32.5)	72 (29.1)	319 (38.4)	407 (39.3)
Neither agree nor disagree	468 (15.3)	37 (15.0)	122 (14.7)	143 (13.8)
Agree	396 (13)	35 (14.2)	86 (10.3)	114 (11.0)
Strongly agree	207 (6.8)	25 (10.1)	33 (4.0)	41 (4.0)
Don't know	79 (2.6)	7 (2.8)	11 (1.3)	26 (2.5)

Most people would self-isolate if they were told to

	General population	Savanta GBMSM	Grindr	Meta
Strongly disagree	132 (4.3)	14 (5.7)	84 (10.1)	90 (8.7)
Disagree	561 (18.4)	42 (17.0)	233 (28.0)	338 (32.6)
Neither agree nor disagree	634 (20.8)	51 (20.6)	180 (21.7)	222 (21.4)
Agree	1127 (37.0)	93 (37.7)	239 (28.8)	311 (30.0)
Strongly agree	461 (15.1)	32 (13.0)	51 (6.1)	36 (3.5)
Don't know	135 (4.4)	15 (6.1)	44 (5.3)	39 (3.8)

I have the support I need to self-isolate for 21 days

	General population	Savanta GBMSM	Grindr	Meta
Strongly disagree	173 (5.7)	26 (10.5)	142 (17.1)	132 (12.7)

For peer review only - http://bmjopen.bmj.com/site/about/guidelines.xhtml

Disagree	348 (11.4)	28 (11.3)	185 (22.3)	245 (23.6)
Neither agree nor disagree	458 (15.0)	41 (16.6)	129 (15.5)	127 (12.3)
Agree	1062 (34.8)	80 (32.4)	226 (27.2)	352 (34.0)
Strongly agree	942 (30.9)	66 (26.7)	133 (16.0)	168 (16.2)
Don't know	67 (2.2)	6 (2.4)	16 (1.9)	12 (1.2)

An effective way to prevent the spread of monkeypox is for people who have tested positive to self-isolate

	General population	Savanta GBMSM	Grindr	Meta
Strongly disagree	51 (1.7)	2 (0.8)	17 (2.0)	21 (2.0)
Disagree	107 (3.5)	13 (5.3)	16 (1.9)	34 (3.3)
Neither agree nor disagree	361 (11.8)	24 (9.7)	45 (5.4)	64 (6.2)
Agree	1018 (33.4)	68 (27.5)	319 (38.4)	362 (34.9)
Strongly agree	1424 (46.7)	132 (53.4)	416 (50.1)	543 (52.4)
Don't know	89 (2.9)	8 (3.2)	18 (2.2)	12 (1.2)

New screen

ASK ALL

Q19 How much do you agree or disagree with the following statements:

If I had to self-isolate because I had tested positive for monkeypox…

RANDOMISE statements

…I would lose touch with my friends and relatives

	General population	Savanta GBMSM	Grindr	Meta
Strongly disagree	567 (18.6)	44 (17.8)	151 (18.2)	180 (17.4)
Disagree	1039 (34.1)	78 (31.6)	320 (38.5)	408 (39.4)
Neither agree nor disagree	614 (20.1)	47 (19.0)	148 (17.8)	180 (17.4)
Agree	539 (17.7)	52 (21.1)	134 (16.1)	200 (19.3)
Strongly agree	225 (7.4)	19 (7.7)	61 (7.3)	65 (6.3)
Don't know	66 (2.2)	7 (2.8)	17 (2)	3 (0.3)

…it would have a severe impact on my family's wellbeing

	General population	Savanta GBMSM	Grindr	Meta
Strongly disagree	420 (13.8)	48 (19.4)	152 (18.3)	181 (17.5)
Disagree	740 (24.3)	58 (23.5)	279 (33.6)	350 (33.8)
Neither agree nor disagree	705 (23.1)	51 (20.6)	159 (19.1)	198 (19.1)
Agree	763 (25.0)	56 (22.7)	148 (17.8)	195 (18.8)

For peer review only - http://bmjopen.bmj.com/site/about/guidelines.xhtml

Strongly agree	338 (11.1)	21 (8.5)	81 (9.7)	99 (9.6)
Don't know	84 (2.8)	13 (5.3)	12 (1.4)	13 (1.3)

…it would have a negative impact on how much money I have

	General population	Savanta GBMSM	Grindr	Meta
Strongly disagree	498 (16.3)	52 (21.1)	132 (15.9)	184 (17.8)
Disagree	791 (25.9)	62 (25.1)	231 (27.8)	357 (34.5)
Neither agree nor disagree	566 (18.6)	27 (10.9)	99 (11.9)	153 (14.8)
Agree	694 (22.8)	56 (22.7)	162 (19.5)	169 (16.3)
Strongly agree	414 (13.6)	43 (17.4)	196 (23.6)	165 (15.9)
Don't know	87 (2.9)	7 (2.8)	11 (1.3)	8 (0.8)

…it would have a negative impact on my work

	General population	Savanta GBMSM	Grindr	Meta
Strongly disagree	545 (17.9)	52 (21.1)	76 (9.1)	98 (9.5)
Disagree	664 (21.8)	38 (15.4)	136 (16.4)	191 (18.4)
Neither agree nor disagree	622 (20.4)	49 (19.8)	100 (12)	136 (13.1)
Agree	697 (22.9)	55 (22.3)	260 (31.3)	325 (31.4)
Strongly agree	405 (13.3)	46 (18.6)	251 (30.2)	264 (25.5)
Don't know	117 (3.8)	7 (2.8)	8 (1.0)	22 (2.1)

…I would miss out on events and activities that I want to attend

	General population	Savanta GBMSM	Grindr	Meta
Strongly disagree	340 (11.1)	32 (13.0)	77 (9.3)	46 (4.4)
Disagree	471 (15.4)	33 (13.4)	106 (12.8)	107 (10.3)
Neither agree nor disagree	607 (19.9)	37 (15.0)	97 (11.7)	117 (11.3)
Agree	1097 (36.0)	84 (34.0)	354 (42.6)	457 (44.1)
Strongly agree	460 (15.1)	55 (22.3)	185 (22.3)	306 (29.5)
Don't know	75 (2.5)	6 (2.4)	12 (1.4)	3 (0.3)

Vaccination

New screen

People who have come into significant contact with someone who has tested positive for monkeypox are being asked to have a smallpox vaccine to reduce the risk of getting seriously ill. As monkeypox is caused by a virus similar to the one that causes smallpox, vaccines designed for smallpox are considered effective in preventing or reducing the severity of monkeypox.

For peer review only - http://bmjopen.bmj.com/site/about/guidelines.xhtml

Like all medicines, this vaccine can cause side effects, but not everyone gets them. The most common side effects are pain and itching at the injection site. Most side effects are mild and clear fully without any treatment within 7 days.

New screen

For each question, please select the answer that reflects your opinion. Do not worry if you do not know what the best answer might be or if you are not at all familiar with monkeypox or vaccination. We only ask that you try to give your answer based on what you think you know or what you would honestly decide to do in the situations described. If you are really not sure, please answer "don't know".

New screen

ASK ALL

Q20 How much do you agree or disagree with the following statements:

RANDOMISE statements

In general, vaccination is a good thing

	General population	Savanta GBMSM	Grindr	Meta
Strongly disagree	65 (2.1)	9 (3.6)	15 (1.8)	6 (0.6)
Disagree	119 (3.9)	4 (1.6)	5 (0.6)	3 (0.3)
Neither agree nor disagree	361 (11.8)	21 (8.5)	33 (4.0)	15 (1.4)
Agree	1019 (33.4)	59 (23.9)	189 (22.7)	142 (13.7)
Strongly agree	1400 (45.9)	148 (59.9)	584 (70.3)	869 (83.9)
Don't know	86 (2.8)	6 (2.4)	5 (0.6)	1 (0.1)

Most people like me will get a smallpox vaccination if advised

	General population	Savanta GBMSM	Grindr	Meta
Strongly disagree	82 (2.7)	5 (2.0)	24 (2.9)	15 (1.4)
Disagree	248 (8.1)	14 (5.7)	41 (4.9)	43 (4.2)
Neither agree nor disagree	533 (17.5)	31 (12.6)	93 (11.2)	122 (11.8)
Agree	1270 (41.6)	111 (44.9)	404 (48.6)	470 (45.4)
Strongly agree	717 (23.5)	69 (27.9)	223 (26.8)	365 (35.2)
Don't know	200 (6.6)	17 (6.9)	46 (5.5)	21 (2.0)

If I get a smallpox vaccination, I will be protected against monkeypox

	General population	Savanta GBMSM	Grindr	Meta
Strongly disagree	100 (3.3)	9 (3.6)	27 (3.2)	6 (0.6)
Disagree	385 (12.6)	19 (7.7)	71 (8.5)	64 (6.2)
Neither agree nor disagree	927 (30.4)	52 (21.1)	158 (19.0)	139 (13.4)

For peer review only - http://bmjopen.bmj.com/site/about/guidelines.xhtml

Agree	953 (31.2)	102 (41.3)	391 (47.1)	599 (57.8)
Strongly agree	250 (8.2)	29 (11.7)	117 (14.1)	190 (18.3)
Don't know	435 (14.3)	36 (14.6)	67 (8.1)	38 (3.7)

A smallpox vaccination could give me smallpox

	General population	Savanta GBMSM	Grindr	Meta
Strongly disagree	614 (20.1)	70 (28.3)	400 (48.1)	668 (64.5)
Disagree	931 (30.5)	65 (26.3)	277 (33.3)	264 (25.5)
Neither agree nor disagree	662 (21.7)	38 (15.4)	69 (8.3)	51 (4.9)
Agree	392 (12.9)	32 (13.0)	27 (3.2)	17 (1.6)
Strongly agree	136 (4.5)	9 (3.6)	8 (1.0)	6 (0.6)
Don't know	315 (10.3)	33 (13.4)	50 (6.0)	30 (2.9)

I might regret getting the smallpox vaccination if I later experienced side effects from it

	General population	Savanta GBMSM	Grindr	Meta
Strongly disagree	420 (13.8)	48 (19.4)	259 (31.2)	410 (39.6)
Disagree	825 (27.0)	74 (30.0)	350 (42.1)	421 (40.6)
Neither agree nor disagree	710 (23.3)	43 (17.4)	88 (10.6)	121 (11.7)
Agree	655 (21.5)	50 (20.2)	89 (10.7)	61 (5.9)
Strongly agree	268 (8.8)	19 (7.7)	31 (3.7)	17 (1.6)
Don't know	172 (5.6)	13 (5.3)	14 (1.7)	6 (0.6)

I am already immune to monkeypox

	General population	Savanta GBMSM	Grindr	Meta
Strongly disagree	676 (22.2)	77 (31.2)	269 (32.4)	366 (35.3)
Disagree	800 (26.2)	51 (20.6)	213 (25.6)	251 (24.2)
Neither agree nor disagree	532 (17.4)	30 (12.1)	106 (12.8)	132 (12.7)
Agree	228 (7.5)	24 (9.7)	57 (6.9)	99 (9.6)
Strongly agree	138 (4.5)	13 (5.3)	26 (3.1)	44 (4.2)
Don't know	676 (22.2)	52 (21.1)	160 (19.3)	144 (13.9)

I would be worried about experiencing side effects from a smallpox vaccination

	General population	Savanta GBMSM	Grindr	Meta
Strongly disagree	392 (12.9)	44 (17.8)	246 (29.6)	364 (35.1)
Disagree	793 (26.0)	72 (29.1)	294 (35.4)	407 (39.3)
Neither agree nor disagree	646 (21.2)	41 (16.6)	105 (12.6)	136 (13.1)
Agree	819 (26.9)	54 (21.9)	146 (17.6)	109 (10.5)
Strongly agree	278 (9.1)	28 (11.3)	23 (2.8)	18 (1.7)
Don't know	122 (4.0)	8 (3.2)	17 (2.0)	2 (0.2)

For peer review only - http://bmjopen.bmj.com/site/about/guidelines.xhtml

I would be worried that having a smallpox vaccine might make me infectious to others

	General population	Savanta GBMSM	Grindr	Meta
Strongly disagree	653 (21.4)	74 (30.0)	454 (54.6)	693 (66.9)
Disagree	1050 (34.4)	72 (29.1)	280 (33.7)	274 (26.4)
Neither agree nor disagree	605 (19.8)	33 (13.4)	46 (5.5)	32 (3.1)
Agree	378 (12.4)	31 (12.6)	18 (2.2)	15 (1.4)
Strongly agree	159 (5.2)	18 (7.3)	5 (0.6)	7 (0.7)
Don't know	205 (6.7)	19 (7.7)	28 (3.4)	15 (1.4)

People who are likely to come into high-risk contact with monkeypox should have a smallpox vaccine

	General population	Savanta GBMSM	Grindr	Meta
Strongly disagree	63 (2.1)	5 (2.0)	13 (1.6)	9 (0.9)
Disagree	158 (5.2)	15 (6.1)	8 (1.0)	5 (0.5)
Neither agree nor disagree	554 (18.2)	32 (13)	57 (6.9)	32 (3.1)
Agree	1134 (37.2)	89 (36)	256 (30.8)	234 (22.6)
Strongly agree	873 (28.6)	89 (36)	465 (56)	745 (71.9)
Don't know	268 (8.8)	17 (6.9)	32 (3.9)	11 (1.1)

New screen

ASK ALL

Q21 To the best of your knowledge, have you received a smallpox vaccine in 2022?

SINGLE CODE

Answer Options

	General population	Savanta GBMSM	Grindr	Meta
Yes	162 (5.3)	26 (10.5)	265 (31.9)	422 (40.7)
No. I was offered the vaccine, but didn't want to have it	116 (3.8)	9 (3.6)	11 (1.3)	16 (1.5)
No, I haven't been offered the vaccine	2609 (85.5)	202 (81.8)	536 (64.5)	587 (56.7)
Don't know / can't remember	163 (5.3)	10 (4.0)	19 (2.3)	11 (1.1)

New screen

ASK IF Q21=2,3,4

Q22 If you were advised by public health officials to have a smallpox vaccine **after coming into high-risk contact with someone who has monkeypox**, how likely would you be to have one?

For peer review only - http://bmjopen.bmj.com/site/about/guidelines.xhtml

SINGLE CODE

Answer Options

	General population, n=2888	Savanta GBMSM, n=221	Grindr, n=566	Meta, n=614
Definitely would not	115 (4.0)	6 (2.7)	16 (2.8)	10 (1.6)
Probably would not	139 (4.8)	15 (6.8)	13 (2.3)	4 (0.7)
Not sure	497 (17.2)	22 (10.0)	17 (3.0)	14 (2.3)
Probably would	814 (28.2)	48 (21.7)	95 (16.8)	51 (8.3)
Definitely would	1323 (45.8)	130 (58.8)	425 (75.1)	535 (87.1)

‡This question was not asked to people who had already had the smallpox vaccine in 2022, therefore total *ns* are different.

New screen

ASK IF Q21=2,3,4

Q23 Some people who have been in contact with someone who has monkeypox are being asked to self-isolate for 21 days. If having a smallpox vaccine means that you would have to self-isolate for less time, how likely would you be to get vaccinated?

SINGLE CODE

Answer Options

	General population, n=2888	Savanta GBMSM, n=221	Grindr, n=566	Meta, n=614
Definitely would not	114 (3.9)	5 (2.3)	17 (3.0)	9 (1.5)
Probably would not	128 (4.4)	9 (4.1)	17 (3.0)	8 (1.3)
Not sure	487 (16.9)	29 (13.1)	31 (5.5)	19 (3.1)
Probably would	822 (28.5)	45 (20.4)	92 (16.3)	55 (9.0)
Definitely would	1337 (46.3)	133 (60.2)	409 (72.3)	523 (85.2)

‡This question was not asked to people who had already had the smallpox vaccine in 2022, therefore total *ns* are different.

New screen

The smallpox vaccine is considered effective in preventing monkeypox or reducing the severity of monkeypox. Some people who are more likely to come into contact with monkeypox are being offered this vaccine.

ASK IF Q21=2,3,4

Q24 If you were offered a smallpox vaccine, how likely would you be to have one?

SINGLE CODE

Answer Options

	General population,	Savanta GBMSM,	Grindr, n=566	Meta, n=614

For peer review only - http://bmjopen.bmj.com/site/about/guidelines.xhtml

	n=2888	n=221		
Definitely would not	111 (3.8)	4 (1.8)	20 (3.5)	12 (2.0)
Probably would not	157 (5.4)	12 (5.4)	14 (2.5)	5 (0.8)
Not sure	518 (17.9)	21 (9.5)	20 (3.5)	21 (3.4)
Probably would	851 (29.5)	64 (29.0)	95 (16.8)	64 (10.4)
Definitely would	1251 (43.3)	120 (54.3)	417 (73.7)	512 (83.4)

‡This question was not asked to people who had already had the smallpox vaccine in 2022, therefore total *ns* are different.

New screen

ASK ALL

Q25. Do you have any further thoughts you would like to share with us about monkeypox, self-isolation, vaccines or anything else to do with monkeypox?

If you do not have anything to add, please put "None".

Type your answer below

OPEN END – require at least one alphabetic/numeric character

New screen

Sociodemographic variables

Now we are going to ask some questions about you. This survey is anonymous. This means that nobody can link you to your answers.

New screen

ASK ALL

D1A In the past month, **have you personally**:

Please select one option for each answer

Gone without enough food to eat

	General population	Savanta GBMSM	Grindr	Meta
Never	2006 (65.8)	171 (69.2)	670 (80.6)	938 (90.5)
Sometimes	663 (21.7)	48 (19.4)	121 (14.6)	85 (8.2)
Often	300 (9.8)	21 (8.5)	28 (3.4)	8 (0.8)
Don't know	63 (2.1)	7 (2.8)	7 (0.8)	3 (0.3)
Prefer not to say	18 (0.6)	0 (0.0)	5 (0.6)	2 (0.2)

Gone without an income

	General population	Savanta GBMSM	Grindr	Meta
Never	2014 (66)	172 (69.6)	653 (78.6)	890 (85.9)

For peer review only - http://bmjopen.bmj.com/site/about/guidelines.xhtml

Sometimes	600 (19.7)	43 (17.4)	105 (12.6)	106 (10.2)
Often	335 (11.0)	24 (9.7)	60 (7.2)	34 (3.3)
Don't know	69 (2.3)	6 (2.4)	2 (0.2)	2 (0.2)
Prefer not to say	32 (1.0)	2 (0.8)	11 (1.3)	4 (0.4)

Gone without fuel for heating or to cook food

	General population	Savanta GBMSM	Grindr	Meta
Never	2232 (73.2)	179 (72.5)	730 (87.8)	978 (94.4)
Sometimes	474 (15.5)	38 (15.4)	72 (8.7)	48 (4.6)
Often	255 (8.4)	25 (10.1)	22 (2.6)	6 (0.6)
Don't know	71 (2.3)	2 (0.8)	1 (0.1)	2 (0.2)
Prefer not to say	18 (0.6)	3 (1.2)	6 (0.7)	2 (0.2)

New screen

ASK ALL

D1B Last month, how difficult was it for you to cover your expenses and pay all your bills?

SINGLE CODE

	General population	Savanta GBMSM	Grindr	Meta
Not at all difficult	980 (32.1)	93 (37.7)	421 (50.7)	675 (65.2)
Somewhat difficult	1148 (37.6)	86 (34.8)	275 (33.1)	271 (26.2)
Very difficult	380 (12.5)	29 (11.7)	53 (6.4)	42 (4.1)
Extremely difficult	407 (13.3)	32 (13)	50 (6.0)	25 (2.4)
Don't know	55 (1.8)	1 (0.4)	2 (0.2)	2 (0.2)
Not applicable, I don't pay the bills in my household	53 (1.7)	5 (2.0)	22 (2.6)	10 (1.0)
Prefer not to say	27 (0.9)	1 (0.4)	8 (1)	11 (1.1)

New screen

ASK ALL

D2 How many people currently live in your household?

Please include yourself and all adults and children – including those not related to you

SINGLE CODE

	General population	Savanta GBMSM	Grindr	Meta
I live alone	663 (21.7)	101 (40.9)	358 (43.1)	380 (36.7)
2	1104 (36.2)	90 (36.4)	313 (37.7)	531 (51.3)

For peer review only - http://bmjopen.bmj.com/site/about/guidelines.xhtml

3 – 4	1055 (34.6)	42 (17.0)	131 (15.8)	103 (9.9)
5 – 6	204 (6.7)	13 (5.3)	25 (3.0)	15 (1.4)
7 +	24 (0.8)	1 (0.4)	4 (0.5)	7 (0.7)

New screen

ASK ALL
D3 Are you the parent / guardian of any dependent children?

Dependent children are those aged under 18 living in your household.
SINGLE CODE

	General population	Savanta GBMSM	Grindr	Meta
Yes, I have one or more dependent children	975 (32.0)	37 (15.0)	35 (4.2)	22 (2.1)
No, I don't have any dependent children	2075 (68.0)	210 (85.0)	796 (95.8)	1014 (97.9)

New screen

ASK ALL
D4. Have you ever provided voluntary care for a family member or friend who needs support due to old age, physical illness, disability, mental health problems, or addiction?
Please select one option
SINGLE CODE

	General population	Savanta GBMSM	Grindr	Meta
Yes, I currently do this	623 (20.4)	45 (18.2)	158 (19.0)	155 (15.0)
I have previously provided care for a friend/family member, but no longer do	897 (29.4)	68 (27.5)	223 (26.8)	315 (30.4)
I have never provided care for a family member/friend in this way	1424 (46.7)	121 (49)	422 (50.8)	541 (52.2)
Prefer not to say	106 (3.5)	13 (5.3)	28 (3.4)	25 (2.4)

New screen

ASK ALL
D5 Do you currently have any pets that live in your home?
MULTI CODE

For peer review only - http://bmjopen.bmj.com/site/about/guidelines.xhtml

	General population	Savanta GBMSM	Grindr	Meta
Yes, I have a dog or cat	1534 (50.3)	117 (47.4)	286 (34.4)	336 (32.4)
Yes, I have a rabbit, ferret, or rodent (e.g. rat, mouse, chinchilla, guinea pig)	195 (6.4)	7 (2.8)	7 (0.8)	10 (1.0)
Yes, I have another type of animal (e.g. reptile, fish, bird)	232 (7.6)	17 (6.9)	43 (5.2)	51 (4.9)
No, I don't have a pet [EXCLUSIVE]	1311 (43.0)	118 (47.8)	516 (62.1)	667 (64.4)

†Participants could select more than one item for this question, therefore column totals add to more than 100%.

New screen

ASK ALL

D6 What is your employment status?

SINGLE CODE

	General population	Savanta GBMSM	Grindr	Meta
Full time paid job (31+ hours)	1163 (38.1)	120 (48.6)	529 (63.7)	626 (60.4)
Part time paid job (<31 hours)	471 (15.4)	21 (8.5)	58 (7.0)	90 (8.7)
Doing paid work on a self-employed basis or within your own business	102 (3.3)	9 (3.6)	82 (9.9)	115 (11.1)
Student / On a government training programme (Nation Traineeship/Modern Apprenticeship)	93 (3.0)	7 (2.8)	35 (4.2)	32 (3.1)
Out of work	120 (3.9)	7 (2.8)	31 (3.7)	19 (1.8)
Looking after home / Homemaker	184 (6.0)	4 (1.6)	3 (0.4)	5 (0.5)
Retired	707 (23.2)	53 (21.5)	57 (6.9)	106 (10.2)
Disabled OR Long-term sick	155 (5.1)	21 (8.5)	25 (3.0)	28 (2.7)
Unpaid work for a business, community or voluntary organisation	27 (0.9)	1 (0.4)	6 (0.7)	11 (1.1)
Prefer not to say	28 (0.9)	4 (1.6)	5 (0.6)	4 (0.4)

New screen

ASK IF D6= CODES 1,2, 3, 4, or 9

For peer review only - http://bmjopen.bmj.com/site/about/guidelines.xhtml

D6A Are you a **frontline health or social care worker**? Please include any voluntary work

A frontline health or social care worker is someone who is directly involved in the care of patients or residents in long-stay care facilities (e.g. a care home), who has face-to-face contact with patients or clients, or who works in a laboratory, pathology or mortuary.

SINGLE CODE
FIX ORDER

	General population, n=1856	Savanta GBMSM, n=158	Grindr, n=710	Meta, n=874
Yes	355 (19.1)	23 (14.6)	94 (13.2)	125 (14.3)
No	1474 (79.4)	135 (85.4)	613 (86.3)	747 (85.5)
Prefer not to say	27 (1.5)	0 (0.0)	3 (0.4)	2 (0.2)

‡This question was only asked to those who reported that they were in full-time, part-time, self-employment, a student, or did unpaid or voluntary work, therefore total *n*s are different.

New screen

ASK IF D6= CODES 1, 2 OR 3
D6B If you needed to, are you able to work from home?
SINGLE CODE

	General population, n=1736	Savanta GBMSM, n=150	Grindr, n=669	Meta, n=831
Yes – full time	632 (36.4)	52 (34.7)	209 (31.2)	294 (35.4)
Yes – most of the time	260 (15.0)	26 (17.3)	136 (20.3)	196 (23.6)
Yes – but I have to go into my place of work once or twice a week	142 (8.2)	13 (8.7)	67 (10.0)	111 (13.4)
No – I cannot do my job from home	702 (40.4)	59 (39.3)	257 (38.4)	230 (27.7)

‡This question was only asked to those who reported that they were in full-time, part-time, or self-employment, therefore total *n*s are different.

New screen

ASK ALL
D7 What is the highest level of educational qualification you have received?
SINGLE CODE

	General population	Savanta GBMSM	Grindr	Meta
PhD/Doctorate	43 (1.4)	7 (2.8)	41 (4.9)	79 (7.6)

For peer review only - http://bmjopen.bmj.com/site/about/guidelines.xhtml

Masters	213 (7.0)	22 (8.9)	204 (24.5)	325 (31.4)
Bachelor's Degree or equivalent (Such as a NVQ level 5)	729 (23.9)	59 (23.9)	291 (35.0)	379 (36.6)
Higher education (Such as a HND or a NVQ level 4)	348 (11.4)	29 (11.7)	106 (12.8)	89 (8.6)
A level or equivalent (Such as Scottish Highers or NVQ level 3)	666 (21.8)	58 (23.5)	110 (13.2)	88 (8.5)
GCSE and below (Such as O level or an RSA Diploma)	747 (24.5)	53 (21.5)	57 (6.9)	63 (6.1)
Other qualifications (Such as NVQ level 1)	119 (3.9)	12 (4.9)	11 (1.3)	11 (1.1)
No qualifications	185 (6.1)	7 (2.8)	11 (1.3)	2 (0.2)

New screen

ASK ALL
D8 Which of the following categories would best describe your ethnicity?
SINGLE CODE

	General population	Savanta GBMSM	Grindr	Meta
White				
British/English/Welsh/Scottish/Northern Irish	2649 (86.9)	214 (86.6)	629 (75.7)	799 (77.1)
Irish	38 (1.2)	5 (2.0)	26 (3.1)	31 (3.0)
Gypsy, Traveller or Irish Traveller	7 (0.2)	0 (0.0)	1 (0.1)	1 (0.1)
Any other White background	71 (2.3)	13 (5.3)	90 (10.8)	131 (12.6)
Mixed/ Multiple ethnic groups				
White and Black Caribbean	18 (0.6)	1 (0.4)	7 (0.8)	4 (0.4)
White and Black African	11 (0.4)	4 (1.6)	1 (0.1)	3 (0.3)
White and Asian	24 (0.8)	1 (0.4)	3 (0.4)	9 (0.9)
Any other Mixed/ Multiple ethnic background	16 (0.5)	1 (0.4)	12 (1.4)	17 (1.6)
Asian or Asian British				
Indian	41 (1.3)	4 (1.6)	20 (2.4)	1 (0.1)
Pakistani	50 (1.6)	0 (0.0)	2 (0.2)	0 (0.0)
Bangladeshi	12 (0.4)	0 (0.0)	1 (0.1)	1 (0.1)
Chinese	14 (0.5)	1 (0.4)	7 (0.8)	7 (0.7)
Any other Asian background	12 (0.4)	0 (0.0)	4 (0.5)	5 (0.5)
Black or Black British				

For peer review only - http://bmjopen.bmj.com/site/about/guidelines.xhtml

African	26 (0.9)	1 (0.4)	5 (0.6)	2 (0.2)
Caribbean	32 (1.0)	0 (0.0)	8 (1.0)	4 (0.4)
Any other Black/ African/ Caribbean background	3 (0.1)	1 (0.4)	1 (0.1)	1 (0.1)
Other ethnic group				
Arab	7 (0.2)	0 (0.0)	3 (0.4)	1 (0.1)
Other	9 (0.3)	1 (0.4)	6 (0.7)	8 (0.8)
Don't know / Prefer not to say				
Don't know	2 (0.1)	0 (0.0)	0 (0.0)	1 (0.1)
Prefer not to say	8 (0.3)	0 (0.0)	5 (0.6)	10 (1.0)

New screen

ASK ALL
D9 Do you, or anyone else in your household have any long-standing illness, disability or infirmity?
Please select all that apply
MULTICODE

	General population	Savanta GBMSM	Grindr	Meta
Yes, I do	843 (27.6)	80 (32.4)	212 (25.5)	305 (29.4)
Yes, another household member does	391 (12.8)	21 (8.5)	88 (10.6)	115 (11.1)
No	1879 (61.6)	149 (60.3)	538 (64.7)	634 (61.2)
Prefer not to say	62 (2)	4 (1.6)	10 (1.2)	14 (1.4)

†Participants could select more than one item for this question, therefore column totals add to more than 100%.

New screen

ASK IF D9=1 (Yes, I do)
D9A Do you have any of the following health conditions?
Please select all that apply
MULTICODE
FIX ORDER

	General population, n=843	Savanta GBMSM, n=80	Grindr, n=212	Meta, n=305
Diabetes	184 (21.8)	21 (26.3)	38 (17.9)	49 (16.1)
Any type of cancer	57 (6.8)	4 (5.0)	10 (4.7)	14 (4.6)
Heart conditions	116 (13.8)	8 (10.0)	24 (11.3)	42 (13.8)
Respiratory conditions	191 (22.7)	15 (18.8)	27 (12.7)	59 (19.3)

For peer review only - http://bmjopen.bmj.com/site/about/guidelines.xhtml
For peer review only

(e.g. chronic obstructive pulmonary disease [COPD], asthma)				
Mental health	317 (37.6)	34 (42.5)	84 (39.6)	102 (33.4)
A skin condition (e.g. atopic dermatitis / eczema)	114 (13.5)	9 (11.3)	25 (11.8)	46 (15.1)
A condition that makes you much more likely to get infections (e.g. SCID, homozygous sickle cell)	20 (2.4)	0 (0.0)	7 (3.3)	7 (2.3)
A weakened immune system	110 (13)	8 (10.0)	24 (11.3)	38 (12.5)
Are taking medicine that weakens your immune system (e.g. steroid tablets, chemotherapy, or antiretroviral medications)	62 (7.4)	4 (5.0)	15 (7.1)	24 (7.9)
HIV/AIDS	6 (0.7)	12 (15.0)	52 (24.5)	117 (38.4)
Other, please specify	183 (21.7)	11 (13.8)	48 (22.6)	60 (19.7)
Prefer not to say	46 (5.5)	5 (6.3)	11 (5.2)	8 (2.6)

†Participants could select more than one item for this question, therefore column totals add to more than 100%.

‡This question was only asked to those who reported that had a chronic illness, therefore total *n*s are different.

New screen

ASK ALL

D9B Are you pregnant?

SINGLE CODE

	General population	Savanta GBMSM	Grindr	Meta
Yes	38 (1.2)	1 (0.4)	0 (0.0)	0 (0.0)
No	3001 (98.4)	245 (99.2)	830 (99.9)	1035 (99.9)
Prefer not to say	11 (0.4)	1 (0.4)	1 (0.1)	1 (0.1)

New screen

ASK ALL

D9C Have you ever taken PrEP (pre-exposure prophylaxis) for HIV?

SINGLE CODE

For peer review only - http://bmjopen.bmj.com/site/about/guidelines.xhtml

For peer review only

	General population	Savanta GBMSM	Grindr	Meta
Yes	42 (1.4)	28 (11.3)	402 (48.4)	487 (47.0)
No	2993 (98.1)	216 (87.4)	427 (51.4)	547 (52.8)
Prefer not to say	15 (0.5)	3 (1.2)	2 (0.2)	2 (0.2)

New screen

ASK ALL

D10 To the best of your knowledge, have you received a vaccine for…

Hepatitis A

	General population	Savanta GBMSM	Grindr	Meta
Yes	531 (17.4)	87 (35.2)	549 (66.1)	773 (74.6)
No. I was offered the vaccine, but didn't want to have it	128 (4.2)	12 (4.9)	7 (0.8)	4 (0.4)
No, I haven't been offered the vaccine	1579 (51.8)	94 (38.1)	129 (15.5)	125 (12.1)
Don't know / can't remember	812 (26.6)	54 (21.9)	146 (17.6)	134 (12.9)

Smallpox (before 2022)

	General population	Savanta GBMSM	Grindr	Meta
Yes	512 (16.8)	51 (20.6)	144 (17.3)	218 (21)
No. I was offered the vaccine, but didn't want to have it	117 (3.8)	12 (4.9)	5 (0.6)	7 (0.7)
No, I haven't been offered the vaccine	1666 (54.6)	133 (53.8)	477 (57.4)	595 (57.4)
Don't know / can't remember	755 (24.8)	51 (20.6)	205 (24.7)	216 (20.8)

Covid-19 (two doses or more)

	General population	Savanta GBMSM	Grindr	Meta
Yes	2557 (83.8)	218 (88.3)	796 (95.8)	1023 (98.7)
No. I was offered the vaccine, but didn't want to have it	242 (7.9)	16 (6.5)	27 (3.2)	11 (1.1)
No, I haven't been offered the vaccine	189 (6.2)	9 (3.6)	5 (0.6)	0 (0.0)
Don't know / can't remember	62 (2.0)	4 (1.6)	3 (0.4)	2 (0.2)

New screen

For peer review only - http://bmjopen.bmj.com/site/about/guidelines.xhtml

ASK ALL
D11 What is your marital status?
SINGLE CODE

	General population	Savanta GBMSM	Grindr	Meta
Single, never married	741 (24.3)	123 (49.8)	470 (56.6)	402 (38.8)
Married / civil partnership	1382 (45.3)	62 (25.1)	149 (17.9)	290 (28)
Separated	69 (2.3)	3 (1.2)	29 (3.5)	21 (2.0)
Divorced	319 (10.5)	14 (5.7)	57 (6.9)	39 (3.8)
Widowed	136 (4.5)	1 (0.4)	5 (0.6)	24 (2.3)
Partnered / in a relationship	393 (12.9)	43 (17.4)	111 (13.4)	251 (24.2)
Prefer not to say	10 (0.3)	1 (0.4)	10 (1.2)	9 (0.9)

New screen

The following questions are sensitive. You don't need to answer if you don't want to. If you don't want to answer, please select "prefer not to say".

New screen

ASK ALL
D12A How many **male** sexual partners have you had…?

By "sexual partner" we mean any form of genital contact, including touching, kissing, and intercourse.

Please give an approximate number if you are unsure. If you have not had any male sexual partners in this time, please put 0.
Type your answer below

		General population	Savanta GBMSM	Grindr	Meta
In the last 3 weeks (21 days)	OPEN TEXT ENTRY – number [cap at 100]	N=2418, M=0.6, SD=3.6, range 0 to 100.	N=210, M=1.1, SD=2.0, range 0 to 14.	N=787, M=2.9, SD=5.2, range 0 to 75.	N=982, M=2.5, SD=4.7, range 0 to 100.
In the last 3 months (90 days)	OPEN TEXT ENTRY – number	N=2418, M=0.8, SD=8.9, range 0 to 400.	N=210, M=2.8, SD=7.0, range 0 to 50.	N=787, M=9.1, SD=23.8, range 0 to 400.	N=982, M=8.9, SD=26.2, range 0 to 400.

For peer review only - http://bmjopen.bmj.com/site/about/guidelines.xhtml

	[cap at 400]				
Prefer not to say		632 (20.7)	37 (15.0)	44 (5.3)	54 (5.2)

New screen

ASK ALL

D12B How many **female** sexual partners have you had…?

By "sexual partner" we mean any form of genital contact, including touching, kissing, and intercourse.

Please give an approximate number if you are unsure. If you have not had any female sexual partners in this time, please put 0.

Type your answer below

		General population	Savanta GBMSM	Grindr	Meta
In the last 3 weeks (21 days)	OPEN TEXT ENTRY – number [cap at 100]	N=2442, M=0.4, SD=3.1, range 0 to 100.	N=207, M=0.2, SD=0.8, range 0 to 100.	N=792, M=0.1, SD=0.6, range 0 to 10.	N=992, M=0.0, SD=0.4, range 0 to 9.
In the last 3 months (90 days)	OPEN TEXT ENTRY – number [cap at 400]	N=2442, M=0.8, SD=9.2, range 0 to 400.	N=207, M=2.2, SD=27.8, range 0 to 400.	N=792, M=0.2, SD=1.6, range 0 to 30.	N=992, M=0.1, SD=1.0, range 0 to 28.
Prefer not to say		608 (19.9)	40 (16.2)	39 (4.7)	44 (4.2)

New screen

Thanks for completing the survey. If you would like any more information on monkeypox, please see https://www.nhs.uk/conditions/monkeypox/.

Please press the Finish button to complete the survey.
Many thanks for completing this survey, your views are extremely important to us.

If you have any further comments or feedback, please use the box below.

For peer review only - http://bmjopen.bmj.com/site/about/guidelines.xhtml

Supplementary materials 2. Data preparation

Some participants (3.8%, n=196) did not provide their postcode and were therefore missing data about region and index of multiple deprivation. While individual items about financial hardship had small amounts of missing data (2.0% to 3.8%, n=105 to 197), as items were summed to give a scale, missingness on the resulting scale would have been higher (7.9%, n=410). There were no apparent patterns to the missing data. We used multiple imputation to impute missing values for region, index of multiple deprivation and financial hardship items. Socio-demographic variables (age, occupation of highest earner, number of people in household, having a dependent child, employment status, education, ethnicity, marital status, and region, index of multiple deprivation and financial hardship where available) were used to predict missing values using a Markov Chain Monte Carlo (MCMC) method using linear regressions with ten iterations, to give five imputations.

We computed a single variable to indicate perceived susceptibility and severity from three items. For these items, "don't know" was coded as the mid-point on the scale. We then multiplied responses together (range 1 to 125) and took the cubic root to give a scale from 1 (lowest perceived susceptibility and severity) to 5 (highest perceived susceptibility and severity). We also asked participants how much they agreed that they were already immune to mpox. This item was recoded into a binary item to show perceived immunity to mpox (strongly agree and agree, vs neither agree nor disagree, disagree, strongly disagree, and don't know).

To create a single variable for perceived knowledge, we used three items (Cronbach's α=0.74). Responses for these items were given on a five-point scale from "strongly disagree" to "strongly agree" We recoded each item as a binary item (strongly agree and agree [1], vs neither agree nor disagree, disagree, strongly disagree, and don't know [0]), then summed responses to give a scale from 0 (lowest perceived knowledge) to 3 (highest perceived knowledge).

For knowledge about mpox symptoms, for each symptom selected, we coded participants as being correct (1, symptom listed on NHS mpox website) or incorrect (0, symptom not listed on NHS mpox website). Scores were then summed to give a scale from 0 (no selected symptoms were mpox symptoms) to 4 (all selected symptoms were mpox symptoms); responses of "don't know" were coded as 0. A single variable denoting understanding of transmission was computed. It is unknown whether mpox can be caught from pet animals

For peer review only - http://bmjopen.bmj.com/site/about/guidelines.xhtml

(but pet animals can catch mpox from humans (1)), therefore we did not include this item in analyses. We coded answers as correct (1) or incorrect (0; answers of “don’t know” coded as incorrect), and summed items to give a score from 0 (lowest knowledge about transmission) to 6 (highest knowledge about transmission).

To reduce the number of variables included in regression models, we used dimension reduction techniques. We conducted principal components analysis, using direct oblimin rotation as items may have been correlated, on items potentially associated with self-isolation (ten items), help seeking (six items), and vaccination (eight items) separately. The number of factors was determined using a scree plot. The item with the highest loading on to a component was then included in regression analyses. Results of the principal components analyses are reported in the supplementary materials 3.

References

1. Seang S, Burrel S, Todesco E, Leducq V, Monsel G, Le Pluart D, et al. Evidence of human-to-dog transmission of monkeypox virus. The Lancet. 2022;400(10353):658-9.

For peer review only - http://bmjopen.bmj.com/site/about/guidelines.xhtml

Supplementary materials 3. Results of principal components analyses.

Self-isolation

A scree plot suggested three components underlying factors potentially associated with self-isolation. Table 1 shows loadings of items onto each component.

Table 1. Loadings of items measuring factors potentially associated with self-isolation onto components identified (only loadings over ± .4 are shown).

Item	Component 1 loading	Component 2 loading	Component 3 loading
If I had mpox symptoms, I wouldn't want to tell anyone as I don't want to self-isolate*		-.864	
If I had mpox symptoms, I wouldn't want to tell anyone so that others don't have to self-isolate		-.853	
Most people would self-isolate if they were told to*			.810
I have the support I need to self-isolate for 21 days			.729
An effective way to prevent the spread of mpox is for people who have tested positive to self-isolate		.544	.426
If I had to self-isolate because I had tested positive for mpox...I would lose touch with my friends and relatives	.627		
If I had to self-isolate because I had tested positive for mpox...it would have a severe impact on my family's wellbeing	.684		
If I had to self-isolate because I had tested positive for mpox...it would have a negative impact on how much money I have	.754		
If I had to self-isolate because I had tested positive for mpox...it would have a negative impact on my work*	.789		
If I had to self-isolate because I had tested positive for mpox...I would miss out on events and activities that I want to attend	.646		

Rotation method: oblimin with Kaiser normalization. Rotation converged in 10 iterations.
*Included in regression analyses

Help seeking

A scree plot suggested two components underlying factors potentially associated with seeking help immediately. Table 2 shows loadings of items onto each component.

Table 2. Loadings of items measuring factors potentially associated with self-isolation onto components identified (only loadings over ± .4 are shown).

Item	Component 1 loading	Component 2 loading
I wouldn't want to know the results of a mpox test		-.526
I would be worried what my friends or family would think about me if they thought I had mpox *	.867	
I would be worried about how colleagues / my employer would react if they thought I had mpox	.845	
I don't want to have a mpox test result on my medical record	.727	
An effective way to prevent the spread of mpox is for people who have symptoms to contact healthcare services		.772
I would be willing to contact a sexual health clinic if I thought I had mpox symptoms or had come into contact with someone who had mpox *		.784

Rotation method: oblimin with Kaiser normalization. Rotation converged in 2 iterations.
*Included in regression analyses

For peer review only - http://bmjopen.bmj.com/site/about/guidelines.xhtml

Vaccination

A scree plot suggested two components underlying factors potentially associated with vaccination. Table 3 shows loadings of items onto each component.

Table 3. Loadings of items measuring factors potentially associated with vaccination onto components identified (only loadings over ± .4 are shown).

Item	Component 1 loading	Component 2 loading
In general, vaccination is a good thing		.627
Most people like me will get a smallpox vaccination if advised		.737
If I get a smallpox vaccination, I will be protected against mpox *		.746
A smallpox vaccination could give me smallpox	.801	
I might regret getting the smallpox vaccination if I later experienced side effects from it	.789	
I would be worried about experiencing side effects from a smallpox vaccination	.793	
I would be worried that having a smallpox vaccine might make me infectious to others*	.843	
People who are likely to come into high-risk contact with mpox should have a smallpox vaccine		.744

Rotation method: oblimin with Kaiser normalization. Rotation converged in 4 iterations.
*Included in regression analyses

For peer review only

Supplementary materials 4. Influence of motivational messages on behavioural outcomes.

Table 1. Influence of motivational messaging on behavioural outcomes, in the general population sample.

			General population, n (%)				
			Risk + perceived necessity/efficacy, n=762	Risk + perceived benefits, n=768	Risk + perceived costs, n=762	Control, n=758	p-value
**Self-isolation for 21 days**	Sum of intention if case and high-risk contact	2 (lowest intention) to 10 (highest intention)	M=8.0, SD=2.2	M=7.9, SD=2.2	M=7.8, SD=2.2	M=8.1, SD=2.1	0.07
**Help seeking**	Any action that involves contacting a healthcare professional (by phone or in person)	Would not seek help or would seek help, but would "wait and see" for a day or two to see if symptoms resolved	362 (47.5)	413 (53.8)	382 (50.1)	391 (51.6)	0.10
		Would seek help immediately	400 (52.5)	355 (46.2)	380 (49.9)	367 (48.4)	
**Contact behaviour**	Have sexual contact with others†	Less than, same as, or more than normal	125 (20.5)	146 (22.6)	148 (23.8)	148 (24.2)	0.41
		Completely stop	485 (79.5)	501 (77.4)	473 (76.2)	464 (75.8)	
**Contact sharing**	Sexual contacts	1 (definitely would not) to 5 (definitely would)	N=748, M=4.2, SD=1.2	N=756, M=4.2, SD=1.2	N=746, M=4.2, SD=1.1	N=747, M=4.1, SD=1.1	0.98
**Vaccination intention**	Smallpox vaccine "if advised"	1 (definitely would not) to 5 (definitely would)	N=723, M=4.0, SD=1.1	N=726, M=4.0, SD=1.1	N=721, M=4.0, SD=1.1	N=718, M=4.0, SD=1.1	0.81

†Answers of "don't know", "prefer not to say" and "not applicable, I wouldn't do this anyway" were coded as missing, therefore total *n* was substantially lower (n=2490).

For peer review only

Table 2. Influence of motivational messaging on behavioural outcomes, by GBMSM sample.

			Savanta GBMSM, n (%)			Grindr, n (%)			Meta, n (%)		
			Motivational message, n=115	Control, n=132	p-value	Motivational message, n=404	Control, n=427	p-value	Motivational message, n=529	Control, n=507	p-value
**Self-isolation for 21 days**	2 (lowest intention) to 10 (highest intention)		M=7.7, SD=2.4	M=8.0, SD=2.1	0.22	M=7.2, SD=2.4	M=7.3, SD=2.5	0.74	M=7.1, SD=2.3	M=7.5, SD=2.2	0.005
**Help seeking**	Any action that involves contacting a healthcare professional (by phone or in person)	Would not seek help or would seek help, but would “wait and see” for a day or two to see if symptoms resolved	59 (51.3)	66 (50.0)	0.84	189 (46.8)	199 (46.6)	0.96	187 (35.3)	204 (40.2)	0.10
		Would seek help immediately	56 (48.7)	66 (50.0)		215 (53.2)	228 (53.4)		342 (64.7)	303 (59.8)	
**Contact behaviour**	Have sexual contact with others†	Less than, same as, or more than normal	26 (26.5)	19 (16.1)	0.06	28 (7.3)	41 (10.1)	0.17	38 (7.5)	31 (6.4)	0.50
		Completely stop	72 (73.5)	99 (83.9)		355 (92.7)	366 (89.9)		469 (92.5)	453 (93.6)	
**Contact sharing**	Sexual contacts	1 (definitely would not) to 5 (definitely would)	N=113, M=4.0, SD=1.2	N=130, M=4.1, SD=1.2	0.54	N=402, M=4.1, SD=1.2	N=421, M=4.1, SD=1.2	0.72	N=526, M=4.1, SD=1.2	N=505, M=4.2, SD=1.1	0.04
**Vaccination intention**	Smallpox vaccine “if advised”‡	1 (definitely would not) to 5 (definitely would)	N=103, M=4.3, SD=1.0	N=118, M=4.2, SD=1.0	0.36	N=282, M=4.6, SD=0.9	N=284, M=4.5, SD=1.0	0.18	N=304, M=4.7, SD=0.9	N=310, M=4.8, SD=0.6	0.03

†Answers of “don’t know”, “prefer not to say” and “not applicable, I wouldn’t do this anyway” were coded as missing, therefore total *n*s were substantially lower (Savanta GBMSM, n=216; Grindr, n=790; Meta, n=991).
‡Not asked to people who reported that they had had the smallpox vaccine in 2022, therefore total *n*s were substantially lower (Savanta GBMSM, n=221; Grindr, n=566; Meta, n=614).

For peer review only - http://bmjopen.bmj.com/site/about/guidelines.xhtml

Supplementary materials 5. Full results of regressions with intention to seek help immediately.

Table 1. Associations between intending to seek help immediately and socio-demographic characteristics and motivational message, by sample. Variables were entered into the logistic regression model in blocks (block 1: socio-demographic variables and motivational message, block 2: psychological factors, block 3: help-seeking specific factors). Results for block 3, using pooled estimates are reported.

Participant characteristics	Level	General population		Grindr	
		aOR (95% CI)	*p*-value	aOR (95% CI)	*p*-value
Gender	Male (including trans man)	Ref	-	-	-
	Female (including trans woman)	1.10 (0.93 to 1.30)	0.25	-	-
Sexual orientation	Straight or heterosexual	Ref	-	-	-
	Gay, lesbian, bisexual, or queer	1.15 (0.84 to 1.58)	0.39	-	-
Age	Range 18 to 98 years	1.012 (1.006 to 1.019)	<0.001*	1.02 (1.01 to 1.04)	0.006
	Quadratic term, $(\text{age} - \text{mean})^2$	1.0002 (0.9999 to 1.0005)	0.24	1.0001 (0.9992 to 1.0011)	0.80
Region	Midlands (East and West)	Ref	-	Ref	-
	South (East, West, East of England)	1.10 (0.87 to 1.40)	0.40	1.01 (0.50 to 2.04)	0.97
	North (East, West, Yorkshire and the Humber)	0.90 (0.71 to 1.15)	0.41	1.53 (0.75 to 3.13)	0.24
	London	0.90 (0.65 to 1.25)	0.54	1.15 (0.59 to 2.26)	0.67
	Devolved nations (Scotland, Wales, and Northern Ireland)	0.83 (0.62 to 1.13)	0.24	1.04 (0.47 to 2.28)	0.93
Dependent child in household	No	Ref	-	Ref	-
	Yes	0.96 (0.78 to 1.17)	0.68	1.58 (0.71 to 3.51)	0.26
Employment status	Not working	Ref	-	Ref	-
	Working	1.12 (0.87 to 1.43)	0.39	0.98 (0.57 to 1.68)	0.94
Frontline health or social care worker	No	Ref	-	Ref	-
	Yes	1.05 (0.81 to 1.36)	0.73	1.91 (1.15 to 3.18)	0.01
Need to leave home for work	Do not need to leave home for work	Ref	-	Ref	-
	Need to leave home for work	1.05 (0.84 to 1.31)	0.65	0.94 (0.64 to 1.37)	0.75
Education	GCSE/vocational/A-level/No formal qualifications	Ref	-	Ref	-
	Degree or higher (Bachelors, Masters, PhD)	0.87 (0.72 to 1.04)	0.12	0.94 (0.66 to 1.34)	0.74
Ethnicity	White British	Ref	-	Ref	-
	White other	1.26 (0.82 to 1.92)	0.29	1.03 (0.65 to 1.65)	0.89
	Black, Asian, other minoritized ethnicity	0.90 (0.66 to 1.22)	0.48	0.95 (0.55 to 1.63)	0.85
Marital status	Not partnered	Ref	-	Ref	-
	Partnered	1.09 (0.87 to 1.36)	0.44	0.81 (0.53 to 1.22)	0.31
Live alone	Live with someone else	Ref	-	Ref	-
	Live alone	1.10 (0.85 to 1.41)	0.48	0.79 (0.55 to 1.15)	0.23
Own chronic illness	None	Ref	-	Ref	-
	Present	1.11 (0.92 to 1.34)	0.28	1.28 (0.88 to 1.88)	0.20
Ever taken PrEP for HIV	No	-	-	Ref	-
	Yes	-	-	0.89 (0.62 to 1.28)	0.54
Vaccinated for smallpox in 2022	Not vaccinated	-	-	Ref	-
	Vaccinated	-	-	0.85 (0.57 to 1.27)	0.43
Index of multiple	Deciles (1st = most deprived, 10th	1.00 (0.97 to 1.03)	0.93	1.05 (0.98 to 1.13)	0.16

For peer review only

deprivation	= least deprived)				
Socio-economic grade	ABC1	Ref	-	Ref	-
	C2DE	0.94 (0.79 to 1.13)	0.53	0.66 (0.41 to 1.07)	0.09
Financial hardship	4 (lowest hardship) to 13 (most hardship)	0.96 (0.92 to 1.00)	0.07	0.99 (0.90 to 1.09)	0.87
Total number of sexual partners (male and female) in last three weeks	0	Ref	-	-	-
	1	1.09 (0.88 to 1.34)	0.45	-	-
	2 to 4	1.09 (0.73 to 1.62)	0.68	-	-
	5 or more	0.78 (0.38 to 1.64)	0.52	-	-
	Prefer not to say	1.09 (0.86 to 1.37)	0.49	-	-
Number of male sexual partners in last three weeks	0	-	-	Ref	-
	1	-	-	1.01 (0.63 to 1.62)	0.95
	2 to 4	-	-	1.01 (0.66 to 1.56)	0.95
	5 to 9	-	-	0.84 (0.47 to 1.49)	0.55
	10 or more	-	-	1.06 (0.51 to 2.17)	0.88
	Prefer not to say	-	-	0.93 (0.43 to 2.03)	0.86
Motivational message	Perceived risk of illness and necessity and efficacy of the response	1.16 (0.93 to 1.44)	0.19	-	-
	Perceived risk of illness and benefits of the response	0.96 (0.77 to 1.20)	0.71	-	-
	Perceived risk of illness and low perceived costs of response	1.05 (0.84 to 1.31)	0.68	-	-
	Control	Ref	-	-	-
Motivational message	All motivational components	-	-	0.86 (0.63 to 1.17)	0.34
	Control	-	-	Ref	-

*$p \leq 0.001$

For peer review only

Table 2. Associations between intending to seek help immediately and psychological and contextual factors, by sample. Variables were entered into the logistic regression model in blocks (block 1: socio-demographic variables and motivational message, block 2: psychological factors, block 3: help-seeking specific factors). Results for block 3, using pooled estimates are reported.

Factor	Level	General population aOR (95% CI)	*p*-value	Grindr aOR (95% CI)	*p*-value
Amount heard about mpox	I have not seen or heard anything (1) to I have seen or heard a lot (3)	1.05 (0.87 to 1.25)	0.63	0.96 (0.67 to 1.37)	0.83
Worry about mpox	Not at all worried (1) to extremely worried (4)	1.05 (0.90 to 1.23)	0.52	1.17 (0.84 to 1.63)	0.36
Perceived risk of mpox to oneself	No risk at all (1) to very high risk (5)	0.85 (0.74 to 0.98)	0.02	0.90 (0.72 to 1.14)	0.38
Perceived risk of mpox to people in UK	No risk at all (1) to very high risk (5)	1.04 (0.90 to 1.19)	0.62	1.37 (1.07 to 1.76)	0.01
Perceived susceptibility and severity	Lowest (1) to highest (5)	1.07 (0.94 to 1.23)	0.32	1.03 (0.77 to 1.36)	0.86
I am already immune to mpox	Strongly disagree, disagree, neither agree nor disagree, don't know	Ref	-	Ref	-
	Strongly agree and agree	0.95 (0.72 to 1.26)	0.73	0.69 (0.40 to 1.19)	0.18
People who catch mpox usually make a full recovery, even if they do not receive any treatment	Strongly disagree (1) to strongly agree (5)	0.87 (0.78 to 0.96)	0.006	0.84 (0.69 to 1.03)	0.09
My personal behaviour has an impact on how mpox spreads	Strongly disagree (1) to strongly agree (5)	1.05 (0.98 to 1.13)	0.19	1.07 (0.92 to 1.25)	0.37
My life has been negatively affected by changes made in response to the mpox outbreak	Strongly disagree (1) to strongly agree (5)	1.00 (0.91 to 1.10)	0.99	1.00 (0.85 to 1.17)	0.99
The risks of mpox are being exaggerated	Strongly disagree (1) to strongly agree (5)	0.83 (0.76 to 0.91)	<0.001*	0.94 (0.77 to 1.13)	0.48
Mpox is only a risk to men who are gay, bisexual or have sex with men	Strongly disagree (1) to strongly agree (5)	0.95 (0.88 to 1.03)	0.22	0.85 (0.72 to 1.01)	0.06
Perceived knowledge	Lowest (0) to highest (3)	0.97 (0.89 to 1.06)	0.54	0.99 (0.80 to 1.23)	0.93
Knowledge of mpox symptoms	Identified no symptoms (0) to identified four symptoms (4)	1.03 (0.96 to 1.09)	0.44	1.13 (0.98 to 1.30)	0.09
Knowledge of mpox transmission	Lowest (0) to highest (6)	0.95 (0.90 to 1.01)	0.12	1.03 (0.89 to 1.20)	0.69
I would be worried what my friends or family would think about me if they thought I had mpox	Strongly disagree (1) to strongly agree (5)	0.94 (0.88 to 1.00)	0.06	0.85 (0.75 to 0.97)	0.01
I would be willing to contact a sexual health clinic if I thought I had mpox symptoms or had come into contact with someone who had mpox	Strongly disagree (1) to strongly agree (5)	1.25 (1.16 to 1.34)	<.001*	1.57 (1.29 to 1.91)	<.001*

*$p \leq 0.001$

For peer review only

Supplementary materials 6. Full results of regressions with smallpox vaccination.

Table 1. Associations between vaccine uptake (general population sample: actual and intended, GBMSM: actual, actual and intended) and socio-demographic characteristics and motivational message, by sample. Variables were entered into the logistic regression model in blocks (block 1: socio-demographic variables and motivational message, block 2: psychological factors, block 3: vaccination specific factors). Results for block 3, using pooled estimates are reported.

Participant characteristics	Level	General population		Grindr			
		Vaccinated in 2022 or would be vaccinated if advised		Vaccinated in 2022		Vaccinated in 2022 or would be vaccinated if advised	
		aOR (95% CI)	*p*-value	aOR (95% CI)	*p*-value	aOR (95% CI)	*p*-value
Gender	Male (including trans man)	Ref	-	-	-	-	-
	Female (including trans woman)	0.93 (0.75 to 1.13)	0.46	-	-	-	-
Sexual orientation	Straight or heterosexual	Ref	-	-	-	-	-
	Gay, lesbian, bisexual, or queer	1.26 (0.85 to 1.88)	0.26	-	-	-	-
Age	Range 18 to 98 years	1.015 (1.007 to 1.023)	<0.001*	1.00 (0.98 to 1.02)	0.81	0.98 (0.94 to 1.02)	0.30
	Quadratic term, $(\text{age} - \text{mean})^2$	1.0007 (1.0002 to 1.0011)	0.002	1.000 (0.998 to 1.001)	0.48	0.999 (0.997 to 1.001)	0.29
Region	Midlands (East and West)	Ref	-	Ref	-	Ref	-
	South (East, West, East of England)	0.82 (0.61 to 1.11)	0.20	0.93 (0.43 to 2.03)	0.86	1.05 (0.22 to 5.12)	0.95
	North (East, West, Yorkshire and the Humber)	0.77 (0.57 to 1.04)	0.09	1.21 (0.51 to 2.87)	0.67	0.48 (0.09 to 2.61)	0.40
	London	0.79 (0.54 to 1.17)	0.24	2.30 (1.04 to 5.11)	0.04	0.68 (0.13 to 3.64)	0.65
	Devolved nations (Scotland, Wales, and Northern Ireland)	0.64 (0.44 to 0.93)	0.02	0.82 (0.31 to 2.13)	0.68	1.91 (0.23 to 16.11)	0.55
Dependent child in household	No	Ref	-	Ref	-	Ref	-
	Yes	0.85 (0.67 to 1.08)	0.19	1.35 (0.49 to 3.71)	0.56	1.11 (0.09 to 13.72)	0.94
Employment status	Not working	Ref	-	Ref	-	Ref	-
	Working	1.34 (0.98 to 1.83)	0.07	1.22 (0.61 to 2.45)	0.57	2.06 (0.42 to 10.12)	0.37
Frontline health or social care worker	No	Ref	-	Ref	-	Ref	-
	Yes	1.46 (1.07 to 1.99)	0.02	0.41 (0.20 to 0.81)	0.01	5.38 (0.90 to 32.19)	0.07
Need to leave home for work	Do not need to leave home for work	Ref	-	Ref	-	Ref	-
	Need to leave home for work	0.56 (0.43 to 0.73)	<0.001*	0.99 (0.61 to 1.60)	0.96	0.28 (0.08 to 1.06)	0.06
Education	GCSE/vocational/A-level/No formal qualifications	Ref	-	Ref	-	Ref	-
	Degree or higher (Bachelors, Masters, PhD)	1.18 (0.94 to 1.47)	0.16	0.78 (0.49 to 1.26)	0.31	1.56 (0.58 to 4.19)	0.38
Ethnicity	White British	Ref	-	Ref	-	Ref	-

For peer review only - http://bmjopen.bmj.com/site/about/guidelines.xhtml

	White other	0.53 (0.33 to 0.85)	0.009	1.27 (0.72 to 2.26)	0.40	0.59 (0.15 to 2.26)	0.44
	Black, Asian, other minoritized ethnicity	0.81 (0.57 to 1.15)	0.24	1.52 (0.78 to 2.99)	0.22	0.41 (0.10 to 1.69)	0.22
Marital status	Not partnered	Ref	-	Ref	-	Ref	-
	Partnered	1.02 (0.79 to 1.33)	0.86	0.74 (0.43 to 1.25)	0.26	2.35 (0.66 to 8.33)	0.19
Live alone	Live with someone else	Ref	-	Ref	-	Ref	-
	Live alone	0.94 (0.69 to 1.27)	0.68	1.30 (0.81 to 2.09)	0.28	1.09 (0.40 to 2.96)	0.86
Own chronic illness	None	Ref	-	Ref	-	Ref	-
	Present	1.07 (0.85 to 1.36)	0.57	1.67 (1.02 to 2.74)	0.04	1.12 (0.39 to 3.25)	0.83
Ever taken PrEP for HIV	No	-	-	Ref	-	Ref	-
	Yes	-	-	8.95 (5.61 to 14.28)	<0.001*	3.41 (1.11 to 10.46)	0.03
Index of multiple deprivation	Deciles (1st = most deprived, 10th = least deprived)	1.00 (0.96 to 1.04)	0.89	0.95 (0.86 to 1.05)	0.28	0.94 (0.78 to 1.13)	0.52
Socio-economic grade	ABC1	Ref	-	Ref	-	Ref	-
	C2DE	0.83 (0.67 to 1.02)	0.08	1.53 (0.82 to 2.87)	0.19	1.39 (0.41 to 4.67)	0.60
Financial hardship	4 (lowest hardship) to 13 (most hardship)	0.95 (0.90 to 0.99)	0.02	1.03 (0.91 to 1.18)	0.62	0.95 (0.73 to 1.24)	0.71
Total number of sexual partners (male and female) in last three weeks	0	Ref	-	-	-	-	-
	1	1.05 (0.80 to 1.37)	0.72	-	-	-	-
	2 to 4	1.33 (0.81 to 2.18)	0.26	-	-	-	-
	5 or more	1.62 (0.64 to 4.09)	0.30	-	-	-	-
	Prefer not to say	0.70 (0.54 to 0.93)	0.01	-	-	-	-
Number of male sexual partners in last three weeks	0	-	-	Ref	-	Ref	-
	1	-	-	1.33 (0.69 to 2.57)	0.40	1.12 (0.33 to 3.77)	0.85
	2 to 4	-	-	1.70 (0.96 to 3.03)	0.07	1.78 (0.56 to 5.65)	0.33
	5 to 9	-	-	2.40 (1.17 to 4.95)	0.02	2.14 (0.34 to 13.33)	0.42
	10 or more	-	-	1.53 (0.65 to 3.62)	0.33	3.80 (0.28 to 52.03)	0.32
	Prefer not to say	-	-	1.21 (0.39 to 3.73)	0.74	0.72 (0.15 to 3.47)	0.68
Motivational message	Perceived risk of illness and necessity and efficacy of the response	0.92 (0.70 to 1.20)	0.53	-	-	-	-
	Perceived risk of illness and benefits of the response	1.15 (0.87 to 1.50)	0.32	-	-	-	-
	Perceived risk of illness and low perceived costs of response	1.02 (0.78 to 1.34)	0.89	-	-	-	-
	Control	Ref	-	-	-	-	-
Motivational message	All motivational components	-	-	0.91 (0.61 to 1.35)	0.63	1.02 (0.43 to 2.39)	0.97
	Control	-	-	Ref	-	Ref	-

*$p \leq 0.001$

For peer review only - http://bmjopen.bmj.com/site/about/guidelines.xhtml

Table 2. Associations between vaccine uptake (general population sample: actual and intended, GBMSM: actual, actual and intended) and psychological and contextual factors, by sample. Variables were entered into the logistic regression model in blocks (block 1: socio-demographic variables and motivational message, block 2: psychological factors, block 3: vaccination specific factors). Results for block 3, using pooled estimates are reported.

Factor	Level	General population<br>Vaccinated in 2022 or would be vaccinated if advised		Grindr<br>Vaccinated in 2022		Vaccinated in 2022 or would be vaccinated if advised	
		aOR (95% CI)	*p*-value	aOR (95% CI)	*p*-value	aOR (95% CI)	*p*-value
Amount heard about mpox	I have not seen or heard anything (1) to I have seen or heard a lot (3)	0.93 (0.75 to 1.15)	0.50	1.74 (1.09 to 2.78)	0.02	0.56 (0.21 to 1.55)	0.27
Worry about mpox	Not at all worried (1) to extremely worried (4)	1.49 (1.24 to 1.80)	<0.001*	1.00 (0.66 to 1.52)	0.99	4.33 (1.69 to 11.07)	0.002
Perceived risk of mpox to oneself	No risk at all (1) to very high risk (5)	0.99 (0.84 to 1.17)	0.92	1.25 (0.93 to 1.67)	0.13	1.19 (0.63 to 2.27)	0.59
Perceived risk of mpox to people in UK	No risk at all (1) to very high risk (5)	1.19 (1.00 to 1.40)	0.05	0.85 (0.62 to 1.16)	0.31	0.93 (0.43 to 2.00)	0.85
Perceived susceptibility and severity	Lowest (1) to highest (5)	1.31 (1.11 to 1.55)	0.001*	0.85 (0.60 to 1.21)	0.37	0.90 (0.40 to 2.05)	0.81
I am already immune to mpox	Strongly disagree, disagree, neither agree nor disagree, don’t know	Ref	-	Ref	-	Ref	-
	Strongly agree and agree	1.30 (0.93 to 1.83)	0.12	8.83 (4.52 to 17.22)	<0.001*	1.30 (0.27 to 6.36)	0.74
People who catch mpox usually make a full recovery, even if they do not receive any treatment	Strongly disagree (1) to strongly agree (5)	1.01 (0.89 to 1.14)	0.90	1.20 (0.94 to 1.53)	0.15	0.51 (0.27 to 0.94)	0.03
My personal behaviour has an impact on how mpox spreads	Strongly disagree (1) to strongly agree (5)	1.00 (0.91 to 1.09)	0.99	0.99 (0.80 to 1.22)	0.92	0.94 (0.63 to 1.39)	0.74
My life has been negatively affected by changes made in response to the mpox outbreak	Strongly disagree (1) to strongly agree (5)	0.93 (0.84 to 1.04)	0.23	0.96 (0.79 to 1.17)	0.70	0.97 (0.63 to 1.51)	0.90
The risks of mpox are being exaggerated	Strongly disagree (1) to strongly agree (5)	0.76 (0.68 to 0.85)	<0.001*	0.78 (0.61 to 1.00)	0.05	0.45 (0.27 to 0.75)	0.002
Mpox is only a risk to men who are gay, bisexual or have sex with men	Strongly disagree (1) to strongly agree (5)	0.97 (0.88 to 1.07)	0.56	0.84 (0.67 to 1.04)	0.11	1.05 (0.66 to 1.65)	0.85
Perceived knowledge	Lowest (0) to highest (3)	1.03 (0.92 to 1.14)	0.64	1.25 (0.93 to 1.67)	0.14	0.78 (0.43 to 1.43)	0.42
Knowledge of mpox symptoms	Identified no symptoms (0) to identified four symptoms (4)	1.02 (0.94 to 1.10)	0.65	1.01 (0.83 to 1.21)	0.95	0.96 (0.67 to 1.38)	0.82
Knowledge of mpox transmission	Lowest (0) to highest (6)	1.04 (0.96 to 1.11)	0.34	0.98 (0.80 to 1.19)	0.82	1.05 (0.71 to 1.55)	0.82
If I get a smallpox vaccination, I will be protected against mpox	Strongly disagree (1) to strongly agree (5)	1.44 (1.29 to 1.61)	<0.001*	1.33 (1.06 to 1.66)	0.01	3.25 (1.97 to 5.35)	<0.001*

For peer review only - http://bmjopen.bmj.com/site/about/guidelines.xhtml

I would be worried that having a smallpox vaccine might make me infectious to others	Strongly disagree (1) to strongly agree (5)	0.64 (0.58 to 0.71)	<0.001*	0.63 (0.46 to 0.87)	0.005	0.44 (0.26 to 0.76)	0.003

*$p \leq 0.001$

For peer review only

For peer review only - http://bmjopen.bmj.com/site/about/guidelines.xhtml

STROBE 2007 (v4) Statement—Checklist of items that should be included in reports of *cross-sectional studies*

Section/Topic	Item #	Recommendation	Reported on page #
**Title and abstract**	1	(*a*) Indicate the study's design with a commonly used term in the title or the abstract	3
		(*b*) Provide in the abstract an informative and balanced summary of what was done and what was found	3
**Introduction**			
Background/rationale	2	Explain the scientific background and rationale for the investigation being reported	5-7
Objectives	3	State specific objectives, including any prespecified hypotheses	7
**Methods**			
Study design	4	Present key elements of study design early in the paper	7-8
Setting	5	Describe the setting, locations, and relevant dates, including periods of recruitment, exposure, follow-up, and data collection	7-8
Participants	6	(*a*) Give the eligibility criteria, and the sources and methods of selection of participants	7-8
Variables	7	Clearly define all outcomes, exposures, predictors, potential confounders, and effect modifiers. Give diagnostic criteria, if applicable	8-12, supplementary materials 1
Data sources/ measurement	8*	For each variable of interest, give sources of data and details of methods of assessment (measurement). Describe comparability of assessment methods if there is more than one group	8-12, supplementary materials 1
Bias	9	Describe any efforts to address potential sources of bias	7-14
Study size	10	Explain how the study size was arrived at	7-8, 12
Quantitative variables	11	Explain how quantitative variables were handled in the analyses. If applicable, describe which groupings were chosen and why	12-14, supplementary materials 2
Statistical methods	12	(*a*) Describe all statistical methods, including those used to control for confounding	12-14
		(*b*) Describe any methods used to examine subgroups and interactions	12-14
		(*c*) Explain how missing data were addressed	12-14, supplementary materials 2

For peer review only

		(*d*) If applicable, describe analytical methods taking account of sampling strategy	12-14
		(*e*) Describe any sensitivity analyses	N/A
**Results**			
Participants	13*	(a) Report numbers of individuals at each stage of study—eg numbers potentially eligible, examined for eligibility, confirmed eligible, included in the study, completing follow-up, and analysed	14-15
		(b) Give reasons for non-participation at each stage	N/A
		(c) Consider use of a flow diagram	Considered
Descriptive data	14*	(a) Give characteristics of study participants (eg demographic, clinical, social) and information on exposures and potential confounders	14-15
		(b) Indicate number of participants with missing data for each variable of interest	Supplementary materials 1 and 2
Outcome data	15*	Report numbers of outcome events or summary measures	16-17, 20, 23, 25, supplementary materials 4
Main results	16	(*a*) Give unadjusted estimates and, if applicable, confounder-adjusted estimates and their precision (eg, 95% confidence interval). Make clear which confounders were adjusted for and why they were included	15-26, supplementary materials 5 and 6
		(*b*) Report category boundaries when continuous variables were categorized	12-14, supplementary materials 2
		(*c*) If relevant, consider translating estimates of relative risk into absolute risk for a meaningful time period	N/A
Other analyses	17	Report other analyses done—eg analyses of subgroups and interactions, and sensitivity analyses	N/A
**Discussion**			
Key results	18	Summarise key results with reference to study objectives	26-30
Limitations	19	Discuss limitations of the study, taking into account sources of potential bias or imprecision. Discuss both direction and magnitude of any potential bias	30
Interpretation	20	Give a cautious overall interpretation of results considering objectives, limitations, multiplicity of analyses, results from similar studies, and other relevant evidence	26-31
Generalisability	21	Discuss the generalisability (external validity) of the study results	26-31
**Other information**			

For peer review only

Funding	22	Give the source of funding and the role of the funders for the present study and, if applicable, for the original study on which the present article is based	32

*Give information separately for cases and controls in case-control studies and, if applicable, for exposed and unexposed groups in cohort and cross-sectional studies.

Note: An Explanation and Elaboration article discusses each checklist item and gives methodological background and published examples of transparent reporting. The STROBE checklist is best used in conjunction with this article (freely available on the Web sites of PLoS Medicine at http://www.plosmedicine.org/, Annals of Internal Medicine at http://www.annals.org/, and Epidemiology at http://www.epidem.com/). Information on the STROBE Initiative is available at www.strobe-statement.org.

For peer review only
